# Late-stage benzenoid-to-troponoid skeletal modification of the cephalotanes exemplified by the total synthesis of harringtonolide

**DOI:** 10.1038/s41467-024-48586-6

**Published:** 2024-05-15

**Authors:** Stefan Wiesler, Goh Sennari, Mihai V. Popescu, Kristen E. Gardner, Kazuhiro Aida, Robert S. Paton, Richmond Sarpong

**Affiliations:** 1 Department of Chemistry, University of California, Berkeley, California USA; 2 Ōmura Satoshi Memorial Institute and Graduate School of Infection Control Sciences, Kitasato University, 5-9-1 Shirokane, Minato-ku, Tokyo Japan; 3 Department of Chemistry, Colorado State University, Ft. Collins, Colorado, USA

**Keywords:** Synthetic chemistry methodology, Reaction mechanisms, Natural product synthesis

## Abstract

Skeletal modifications enable elegant and rapid access to various derivatives of a compound that would otherwise be difficult to prepare. They are therefore a powerful tool, especially in the synthesis of natural products or drug discovery, to explore different natural products or to improve the properties of a drug candidate starting from a common intermediate. Inspired by the biosynthesis of the cephalotane natural products, we report here a single-atom insertion into the framework of the benzenoid subfamily, providing access to the troponoid congeners — representing the reverse of the proposed biosynthesis (i.e., a contra-biosynthesis approach). Computational evaluation of our designed transformation prompted us to investigate a Büchner–Curtius–Schlotterbeck reaction of a *p*-quinol methylether, which ultimately results in the synthesis of harringtonolide in two steps from cephanolide A, which we had previously prepared. Additional computational studies reveal that unconventional selectivity outcomes are driven by the choice of a Lewis acid and the nucleophile, which should inform further developments of these types of reactions.

## Abstract (web summary)

Many natural products exist as families of structurally similar molecules, and therefore developing skeletal modifications of common intermediates offers flexible and powerful approaches for target synthesis. Here, the authors report a single-atom insertion into the framework of the benzenoid subfamily, providing access to the troponoid congeners.

## Introduction

**Fig. 1:**
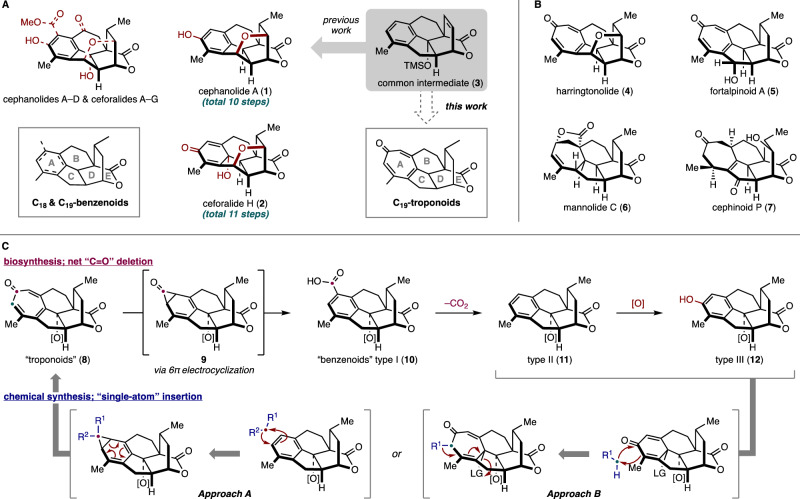
Cephalotane natural products. **A** Benzenoid subfamily and our previous work. **B** Troponoids and non-aromatic seven-membered ring congeners. **C** Biosynthesis of the cephalotanes and our strategy employing a single-atom insertion. [O]: oxidation.

The total synthesis of natural products remains an active area of research in chemical synthesis^1–3^. In cases where many congeners of a family of natural products are targeted for synthesis, it is often more efficient to prepare a late-stage intermediate that can be diversified to access the entire collection^4,5^. In some instances, such late-stage diversification approaches have closely mimicked the biosynthetic pathway to the targeted molecules. For example, congeners of terpenoid secondary metabolites often arise from oxidation or oxygenation reactions that are effected by tailoring P450 enzymes in what has come to be referred to as the oxidase phase^6,7^. This general approach has been adopted to great effect in preparing many terpenoids^8–11^. In our laboratory, we have applied the late-stage diversification approach to the syntheses of members of the longiborneols^12,13^, the phomactins^14,15^, the diterpenoid alkaloids^16,17^, and more recently, cephalotane natural products such as the cephanolides and ceforalides (e.g., **1** and **2**) that were prepared from pentacycle **3** (Fig. 1A)^18,19^.

The cephanolides^20^ and ceforalides^21^ are structurally related to harringtonolide (**4**), first isolated in 1978 from *C. harringtonia* (Fig. 1B)^22^. This natural product has been shown to possess interesting bioactivity, including antiviral and antineoplastic activity^23,24^. The key difference between these structures is that the cephanolides and ceforalides bear an arene A-ring or oxidized variant thereof (hence our reference to these compounds as the benzenoid congeners), whereas **4** possesses a tropone A-ring. Over the last half-decade, a large number of additional troponoids and non-aromatic seven-membered A-ring cephalotane congeners have been isolated^25^, including the fortalpinoids (e.g., **5**)^26^, mannolides (e.g., **6**)^27^, and cephinoids (e.g., **7**)^28^. While syntheses of these latter classes of cephalotanes are beginning to appear^29–31^, harringtonolide remains a popular synthetic target^32–34^. Biosynthetically, it is proposed that the benzenoids might be derived from the troponoid subfamily (e.g., **8**, Fig. 1C) through a 6π electrocyclization to arrive at the corresponding cyclopropanone (**9**), which, following a Baeyer–Villiger type oxidation and aromatization, would give the benzenoid type I framework (**10**). Subsequent decarboxylation and oxidation events would then yield a variety of other congeners bearing the benzenoid type II and III frameworks (**11** and **12**)^20,35^. This proposed cephalotane biosynthesis, which relies on a net one-carbon deletion inspired us to explore a contra-biosynthetic approach employing single-atom insertion to prepare the troponoids from the benzenoid subfamily^36^.

Strategies to achieve such single-atom skeletal edits to access privileged scaffolds continue to emerge and draw the interest of the synthesis community^37^. Because nitrogen-containing heteroaromatics are the most commonly occurring structural motifs in pharmaceuticals and agrochemicals^38,39^, many current methods for skeletal editing have relied on the intrinsic reactivity of aza-heterocycles. In our planned approach, we saw an opportunity to highlight skeletal editing of carbocyclic arenes through ring expansion (and ultimately, also ring contraction) to access families of natural products. The challenge of effecting a skeletal change in complex sp^3^-rich polycyclic structures with multiple functionalities offered opportunities to develop new methods. Here, we report the realization of the benzenoid-to-troponoid conversion of the cephalotanes, culminating in a two-step synthesis of harringtonolide from cephanolide A. Notably, the success of our studies was guided by valuable insights gained through computational analysis of the key ring expansion reaction.

## Results and discussion

**Fig. 2:**
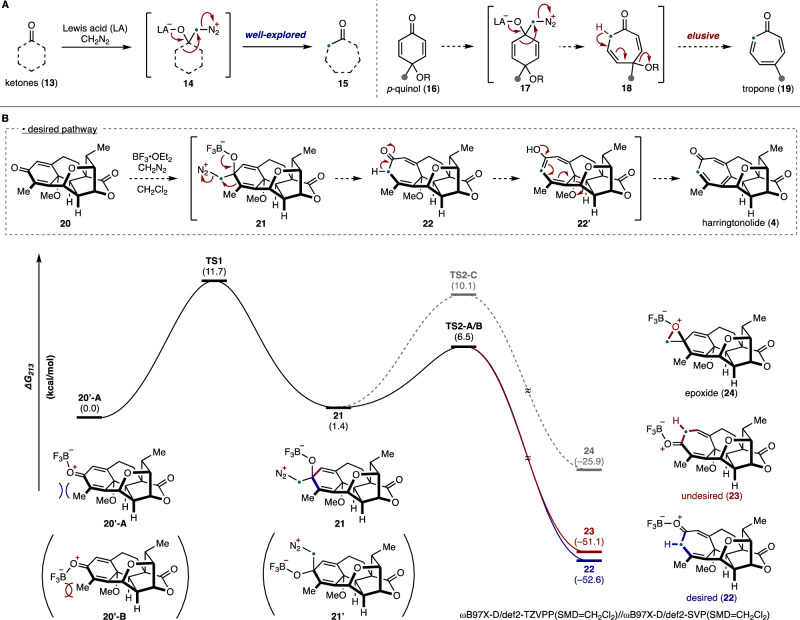
Reaction design for tropone synthesis. **A** Büchner–Curtius–Schlotterbeck reaction and our hypothesis. **B** Computational study performed at the ωB97X-D/def2-TZVPP(SMD=CH_2_Cl_2_)//ωB97X-D/def2-SVP(SMD=CH_2_Cl_2_) level of theory to evaluate the feasibility of tropone formation. LA: Lewis acid, *p-*: para-.

Harringtonolide (**4**) has been synthesized by the groups of Mander^32^, Tang^33^, and Zhai^34^ using highly innovative approaches. In particular, Mander’s approach^32^, which relied on an intramolecular Büchner reaction^40,41^, was highly inspirational to our planned benzenoid-to-troponoid conversion for the synthesis of **4** (Fig. 1C, Approach A). However, this approach was uniformly unsuccessful even following an extensive survey of reaction conditions (Supplementary Table 1)^42–48^. Given the limitations of our attempted intermolecular cycloadditions, we decided to investigate different ring expansion approaches that relied on the reactivity of carbonyl groups (Fig. 1C, Approach B). We were drawn to oxidative dearomatizations of phenols to provide quinols^49^, which could be followed by a Büchner–Curtius–Schlotterbeck (BCS) reaction^50–52^ to afford the desired tropone moiety (Fig. 2A)^53–59^. In general, the BCS reaction has been well-explored and established using saturated ketones^60^. To the best of our knowledge, there were no reports employing *p*-quinol derivatives such as **16** as substrates when we carried out these studies. However, during the review of our work, related studies appeared^61–63^. We postulated that a BCS reaction using **16** would undergo ring expansion to **18** via **17**. An elimination of the alkoxy group in **18** would yield the desired tropone (**19**).

### Reaction design and experimental investigation

To evaluate the feasibility of this tropone synthesis, we have undertaken computational studies as outlined in Fig. 2B^64^. In the context of the synthesis of harringtonolide (**4**), we envisioned using quinol methylether **20**, which would undergo a one-carbon insertion by a BCS ring-expansion via **21**, followed by tautomerization of **22** and loss of methanol (see **22’**) to afford **4**. We first began our calculations at the several levels of theory (Supplementary Fig. 6A) (Quantum chemical calculations were performed with Gaussian 16 rev. C.01 for geometry optimizations and ORCA 5.0.4 for single-point energy corrections; see the Supplementary Information for full computational details and references.)^65–68^ by modeling the reaction of **20** with CH_2_N_2_ in the presence of BF_3_•OEt_2_, which represents one of the most commonly employed conditions for these types of reactions^50^. We theorized that the Lewis acid likely binds to the carbonyl lone-pair of **20** away from the α-Me group, as shown in **20’-A**. At this stage, two diastereoselective additions of CH_2_N_2_ are possible, leading to adducts **21** or **21’**, respectively, in which the convex adduct **21** (via **TS1**; ∆G^‡^ = 11.7 kcal/mol) is marginally favored by 0.6 kcal/mol. The formation of the tropone ring by ring expansion of **21** is energetically feasible via **TS2-A/B** (∆G^‡^ = 6.5 kcal/mol), leading to two constitutional isomers (**22** and **23**). We also found the possibility of intramolecular oxygen replacement via **TS2-C** to give rise to epoxide **24**. Overall, the C–C migration (**TS2**) was calculated to be product-determining, wherein a Curtin–Hammett scenario is one of many possibilities to account for our observations.

**Fig. 3:**
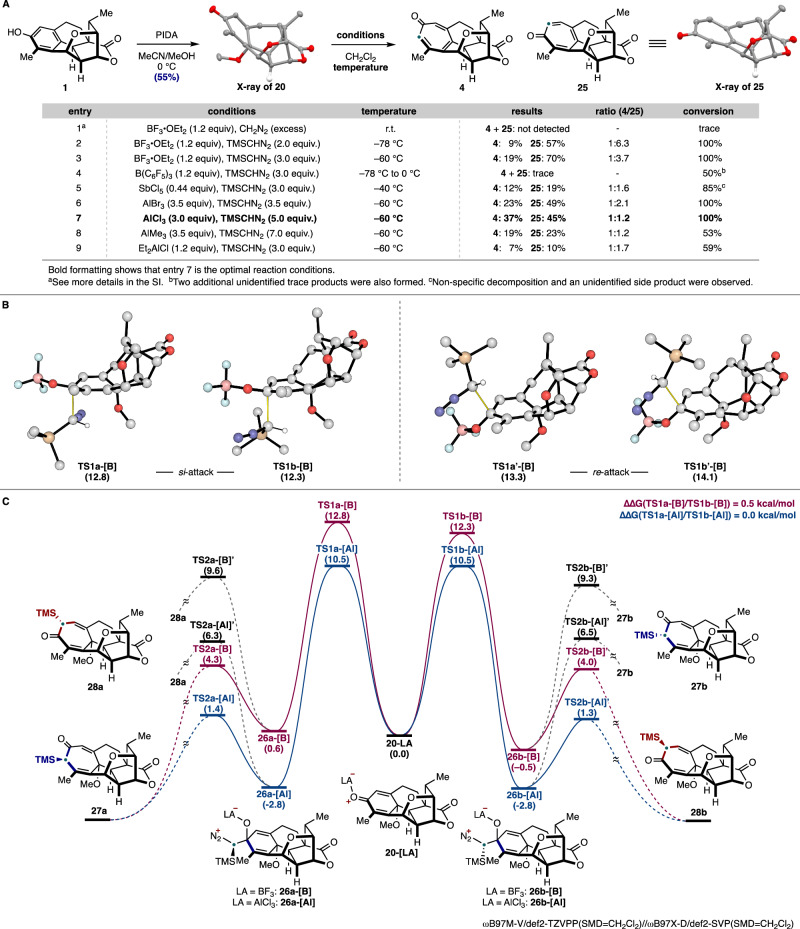
Experimental and computational investigation of the late-stage ring-expansion. **A** Optimization table for the synthesis of harringtonolide. **B** Selectivity for the nucleophilic attack of TMSCHN_2_ on the two prochiral faces of substrate **20-[B]**. **C** Potential energy surface for the reaction between TMSCHN_2_ and **20-[LA]**; All calculations were performed at the ωB97M-V/def2-TZVPP(SMD=CH_2_Cl_2_)//ωB97X-D/def2-SVP(SMD=CH_2_Cl_2_) level of theory. PIDA: phenyliodine(III) diacetate, TMS: trimethylsilyl, TS: transition state.

Given the promising preliminary computational results, we commenced our investigation of the planned BCS reaction by preparing *p*-quinol derivative **20** (Fig. 3A). Treatment of cephanolide A (**1**), which was prepared by a modified 12-step sequence (Supplementary Fig. 1), with Kita oxidative dearomatization conditions^69,70^ afforded **20** in 55% yield. Based on our preliminary calculations, we initially attempted conditions using CH_2_N_2_ in the presence of BF_3_•OEt_2_ for the tropone formation (Table in Fig. 3A). Unfortunately, these conditions were ineffective and led primarily to the recovery of the starting material (entry 1). Likely, CH_2_N_2_ was not nucleophilic enough to react with the carbonyl group of **20** and decomposed under the conditions. Therefore, we turned to other diazomethane equivalents and first examined TMSCHN_2_ (2.0 equiv) in the presence of BF_3_•OEt_2_ (1.2 equiv). To our delight, conducting the reaction at –78 °C yielded tropone **4** but in only 9% isolated yield along with a 57% yield of **25** (a 1:6.3 ratio; entry 2). We also found that using 3.0 equiv of TMSCHN_2_ at –60 °C, **20** was fully consumed to give **4** in 19% yield and **25** in 70% yield (a 1:3.7 ratio; entry 3). To increase the selectivity for the formation of **4**, we then screened a range of Lewis acids (entries 4–9). As a result, we found that AlCl_3_ (3.0 equiv) along with 5.0 equiv of TMSCHN_2_ converted **20** to a 37% yield of **4** and 45% yield of **25** (a 1:1.2 ratio; entry 7). Overall, these conditions proved to be optimal (see Supplementary Tables 2 and 3 for full details). Of note, the conversion of **20** to harringtonolide (**4**) represents the shortest synthesis of this natural product reported to date (14 steps from commercially available material). The selectivity outcome, unexpected based on our preliminary DFT calculations with CH_2_N_2_ (entry 3), as well as the improved ratio obtained using AlCl_3_ (entry 7), led us to undertake additional calculations to gain more insight into the selectivity of this reaction.

### Computational studies

With some experimental results in hand, we performed benchmarking computational studies to rationalize the observed selectivity using a range of computational protocols. Based on our computational benchmarking, we found that ωB97M-V/def2-TZVPP(SMD=CH_2_Cl_2_)//ωB97X-D/def2-SVP(SMD=CH_2_Cl_2_) level of theory most accurately reproduced the empirically observed selectivity. A revised PES has also been calculated for the reaction of **20** with CH_2_N_2_ described in Fig. 2B (Supplementary Fig. 6B). Our calculations showed that the attack of TMSCHN_2_ should occur on the *si*-face of **20-[LA]** — favored by 1 kcal/mol in the case of the BF_3_-activated substrate (Fig. 3B). However, two possible orientations of the attacking nucleophile are possible, leading to either **26a** or **26b**. Rearrangement of **26a** would yield **27a** or **28a**, whereas **26b** would lead to **27b** or **28b**. For the computed scenario with BF_3_•OEt_2_ as the Lewis acid at –60 °C (Fig. 3C), **TS1a-[B]** was found to have a 0.5 kcal/mol higher barrier compared to **TS1b-[B]**. In this case, we believe that the energy difference between **TS1a-[B]** and **TS1b-[B]** accounts for the observed distribution of products, which compares favorably with the empirical observation (i.e., the ratio of **4**/**25** = 1:3.7, which corresponds to a ~ 0.6 kcal/mol difference). With AlCl_3_ as a Lewis acid, there is no difference in stability between **TS1a-[Al]** and **TS1b-[Al]**, consistent with our observed ratio (**4**/**25** = 1:1.2). Overall, these computational results show that in the BCS reaction using TMSCHN_2_, the addition of TMSCHN_2_ (**TS1**) to the Lewis acid-bound *p*-quinol derivative is the selectivity-dictating step.

**Fig. 4:**
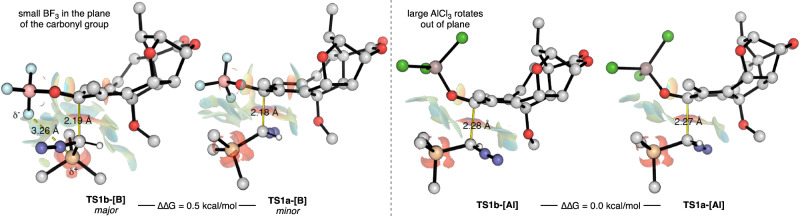
Comparison in the product-determining TSs for reactions mediated by BF_3_•OEt_2_ and AlCl_3_. Effect of the Lewis acid on the product-determining TS of the reaction demonstrated for BF_3_•OEt_2_ and AlCl_3_. The relatively small BF_3_•OEt_2_ (left) lies in the plane of the carbonyl group, leading to TS1b-[B] as the favored TS, whereas the larger Lewis acid AlCl_3_ (right) rotates out of the plane and therefore both TS are equally present. TS: transition state.

To gain deeper insight into the impact of the choice of Lewis acid on the reaction outcome, we conducted a comprehensive analysis of the product-determining TSs for both the BF_3_•OEt_2_ and AlCl_3_-mediated systems (Fig. 4). In the case of the minor pathway via **TS1a-[B]**, we observed a C–C bond distance of 2.18 Å between the nucleophilic carbon of TMSCHN_2_ and the adjacent carbonyl group in an eclipsed orientation. This unexpected, eclipsed orientation of the incoming substituents along the forming C–C bond can be attributed to favorable dispersive interactions between the highly polarizable TMS group and the carbonyl group, as evidenced by the non-covalent interaction (NCI) isosurfaces^71^. In addition, in the case of **TS1b-[B]**, which features a similar C–C bond distance of 2.19 Å, we observed a staggered orientation of the substituents, with the TMS group placed in close proximity to the BF_3_ Lewis acid, which sits in the plane of the carbonyl group. This change in orientation from eclipsed to staggered is driven by the favorable interactions between the partially negatively charged fluoride atoms and the electropositive silicon atom, located within 3.26 Å. As such, the preferential reactivity via **TS1b-[B]** can be attributed to favorable electrostatic and dispersive interactions between the TMS group and the Lewis acid in the case of BF_3_•OEt_2_.

When we conducted a similar analysis on the same two competing TSs (i.e., **TS1a-[Al]** and **TS1b-[Al]**), using AlCl_3_ as the Lewis acid, some significant structural differences emerged. Firstly, the forming C–C bonds between TMSCHN_2_ and the substrate were found to be 0.1 Å longer, which is consistent with earlier TSs, indicating the lower activation energy barriers in this case compared to using BF_3_ (as shown in Fig. 3). However, a more significant change was observed in the orientation of the AlCl_3_ group, which moved out of co-planarity with the carbonyl group due to increased steric demand. This fundamental structural alteration eliminates any favorable interaction between the Lewis acid and the TMS group in **TS1b-[Al]** and promotes the reorientation of the TMS group to a more favorable eclipsed position, similar to that observed in **TS1a-[Al]/[B]**. Consequently, this loss of favorable non-covalent interactions destabilizes **TS1b-[Al]**, resulting, overall, in better selectivity toward the desired product **27**. Finally, in a preliminary study, we have shown that the tropone formation can be extended to other substrates (Supplementary Fig. 2).

In conclusion, we have shown that an oxidative dearomatization and ring expansion starting from cephanolide A accomplishes a benzenoid-to-troponoid ring expansion to afford harringtonolide. To gain insight into the regioselectivity-determining factors in the ring expansion reaction, we have carried out extensive computational studies. These calculations have unveiled the unique effects of the different Lewis acids in establishing secondary interactions with TMSCHN_2_ which significantly affect the regioselectivity by changing the relative energies of the different transition structures. The extension of the ring expansion transformation described here to other quinol derivatives are provided in Supplementary Fig. 2. Future studies will focus on the application of the Büchner–Curtius–Schlotterbeck transformation to other natural product classes.

## Methods

### General considerations

Commercial reagents and solvents were purchased from Fisher Scientific, Acros Organics, Alfa Aesar, and/or Sigma Aldrich, and used without additional purification. Diazomethane (CH_2_N_2_) was generated using an Aldrich® diazomethane-generator with System 45^TM^. MeCN and MeOH were sparged with argon and dried by passing through alumina columns using argon in a Glass Contour solvent purification system. DCM was freshly distilled over calcium hydride under a N_2_ atmosphere before each use. Reaction progress was monitored by thin-layer chromatography (TLC) on Macherey-Nagel TLC plates (60 Å, F254 indicator). TLC plates were visualized by exposure to ultraviolet light (254 nm), and/or stained by submersion in aqueous potassium permanganate solution (KMnO_4_), *p*-anisaldehyde, or phosphomolybdic acid stain and heating with a heat gun. Organic solutions were concentrated under reduced pressure on a Heidolph temperature-controlled rotary evaporator equipped with a dry ice/isopropanol condenser.

### Oxidative dearomatization

To a solution of cephanolide A (**1**) (25.0 mg, 83.8 μmol, 1.0 equiv) in MeCN/MeOH (1:1 v/v, 838 µL, 0.1 M) was added phenyliodine(III) diacetate (PIDA; 32.4 mg, 101 μmol, 1.2 equiv) at 0 °C under a N_2_ atmosphere. After stirring at room temperature for 5 h, the reaction mixture was quenched with sat. aq. NaHCO_3_ (2 mL), diluted with H_2_O (3 mL) and extracted with DCM (3 × 5 mL). The combined organic phase was dried over Na_2_SO_4_ and concentrated in vacuo. The resulting residue was purified by silica gel flash column chromatography (hexanes/EtOAc = 2:1), yielding methyl-ceforalide H (**20**) (15.2 mg, 46.3 μmol, 55%) as a colorless solid.

### Ring-expansion

A flame-dried vial with a magnetic stir bar was transferred to a glovebox and charged with AlCl_3_ (12.2 mg, 91.4 µmol, 3.0 equiv). The vial was sealed with a septa cap and removed from the glove box. The vial was evacuated and backfilled with N_2_ three times and cooled to –60 °C. Freshly distilled DCM (50 µL) was added, and the suspension was stirred at –60 °C for 5 min. A solution of methyl-ceforalide H (**20**) (10.0 mg, 30.5 µmol, 1.0 equiv) in freshly distilled DCM (250 µL) was added and stirred at –60 °C for 10 min to give a grayish suspension. TMS-diazomethane (0.2 M, prepared from a 2.0 M solution in hexanes diluted with freshly distilled DCM, 760 µL, 152 µmol, 5.0 equiv) was added over 2 min resulting in a yellowish solution. The mixture was stirred at –60 °C for 3 h and quenched with sat. aq. NaHCO_3_ (500 µL). The suspension was diluted with H_2_O (2 mL) and extracted with DCM (3 × 3 mL). The combined organic layers were dried over anhydrous Na_2_SO_4_, and the solvent was removed under reduced pressure. The residue was purified by preparative TLC (hexanes/EtOAc = 1:3), yielding harringtonolide (**4**) (3.5 mg, 11.3 µmol, 37%) as a colorless solid and *iso*-harringtonolide (**25**) (4.3 mg, 13.9 µmol, 46%) as a colorless solid.

### Computational methods

The range-separated dispersion corrected ωB97X-D density functional^65^ was used in conjunction with the double-zeta valence polarized def2-SVP basis set^67^, to optimize the geometry of all stationary points. Additional single points energy correction was carried out with the newer generation meta-augmented range separated density functional ωB97M-V^9^ that employs the Vydrov and van Voorhis VV10 dispersion correction^72^, together with the triple-zeta valence polarized def2-TZVPP basis set. The VV10 dispersion corrected family of functionals developed by the Head-Gordon group have been demonstrated to be one of the most robust functionals for assessment of main group thermochemistry and for describing non-covalent interactions (Quantum chemical calculations were performed with Gaussian 16 rev. C.01 for geometry optimizations and ORCA 5.0.4 for single-point energy corrections; see the Supplementary Information for full computational details and references.). All calculations included the integral equation formalism variant of the polarizable continuum model (IEF-PCM), with the SMD solvation model to account for solvation effects (solvent = dichloromethane)^68^. Conformational sampling was performed manually. Gaussian16 version C.01 was employed for all density functional theory (DFT) geometry optimization calculations, using the default ultrafine pruned (99,590) grid for numerical integration of the exchange-correlation functional and its derivatives (Quantum chemical calculations were performed with Gaussian 16 rev. C.01 for geometry optimizations and ORCA 5.0.4 for single-point energy corrections; see the Supplementary Information for full computational details and references.). Single point corrections were carried our using ORCA 5.0.4^73^. Vibrational frequency calculations were used to verify that stationary points were either minima or first-order saddle points on the corresponding potential energy surface. Additional intrinsic reaction coordinate (IRC) calculations were performed to ensure that the transition state structures connected to their appropriate initial and final geometries^74^. The computed thermochemistry data were further corrected following Grimme’s quasi-harmonic (QHA)^75^ model for entropy with a frequency cut-off value of 100.0 cm^−1^ using the GoodVibes^76^ program at 213.15 K (−60° C). In addition, GoodVibes applied 1 M standard concentration corrections to all individual calculations to account for reactions in solution (i.e., change in standard concentration from 1 atm to 1 M)^77^. XYZ coordinate files were also generated using GoodVibes.

### Supplementary information

Supplementary Information

Peer Review File

## Data Availability

The data supporting the findings of this study are available within the paper and its Supplementary Information. Detailed information for reaction conditions, compound characterization data, computational data, and crystallographic data are in the Supplementary Information. The X-ray crystallographic coordinates for structures reported in this study have been deposited at the Cambridge Crystallographic Data Center (CCDC), under deposition numbers 2293695 (**20**) and 2293696 (**25**). These data can be obtained free of charge from The Cambridge Crystallographic Data Center via www.ccdc.cam.ac.uk/data_request/cif. All data are available upon request from the corresponding authors.
