# Supplementary material for: Late-stage benzenoid-to-troponoid skeletal modification of the cephalotanes exemplified by the total synthesis of harringtonolide
Source: Nat Commun. 2024 May 15;15:4125. doi: 10.1038/s41467-024-48586-6 (PMC11096412; doi:10.1038/s41467-024-48586-6)
Supplement: Supplementary file 1 — Supplementary Information [file 41467_2024_48586_MOESM1_ESM.pdf]

Supplementary Information

Late-stage benzenoid-to-troponoid skeletal modification of the cephalotanes exemplified by the total synthesis of harringtonolide

Stefan Wiesler,[1†] Goh Sennari,[1,2†] Mihai V. Popescu,[3] Kristen E. Gardner,[1] Kazuhiro Aida,[1]
Robert S. Paton,[3]* Richmond Sarpong[1]*

[1]Department of Chemistry, University of California, Berkeley, CA 94720, United States
[2]Ōmura Satoshi Memorial Institute, Kitasato University, 5-9-1 Shirokane, Minato-ku, Tokyo 108-8641, Japan
[3]Department of Chemistry, Colorado State University, Ft. Collins, Colorado 80523-1872, United States

Corresponding Authors

*robert.paton@colostate.edu (R.P.)
*rsarpong@berkeley.edu (R.S).

1. General Considerations 1

1-1. Solvents and Reagents 1

1-2. Experimental Procedures 1

1-3. Analytical Instrumentation 1

2. Reaction Investigations 2

2-1. **Supplementary Table 1:** Unsuccessful attempts to achieve intermolecular ring expansion (Approach A) 2

2-2. **Supplementary Table 2:** Initial attempts at the Büchner–Curtius–Schlotterbeck (BCS) reaction using CH_2N_2 3

2-3. **Supplementary Table 3:** Selected optimization of the BCS reaction using $TMSCHN_2$ 4

3. Experimental Procedures and Characterization Data of New Compounds 6

4. Large-Scale Synthesis of Cephanolide A
Supplementary Figure 1: *12-step total synthesis sequence* **.. 9**

5. Additional Data
Supplementary Figure 2: *Tropone synthesis using simple substrates* **............................ 19**

6. Spectral Data Comparison of Natural and Synthetic Harringtonolide 24

7. NMR Spectra Charts **26**

8. Crystallographic Data **40**

8-1. **Supplementary Figure 3:** X-ray structure of Me-ceforalide H (**20**) (CCDC 2293695) 40

8-2. **Supplementary Figure 4:** X-ray structure of *Iso*-harringtonolide (**25**) (CCDC 2293696) 53

9. Computational Studies **65**

9-1. Computational Methods 65

9-2. Benchmarking 66

9-3. Potential Energy Surface Using CH_2N_2 and BF_3•OEt_2 67

9-4. Potential Energy Surface Using $TMSCHN_2$ and $AlCl_3$ 72

9-5. Potential Energy Surface Using $TMSCHN_2$ and BF_3•OEt_2 76

9-6. NCIPLOT of Key Transition States 80

9-7. Lewis Acid Coordination Thermochemistry 81

9-8. Thermochemistry 82

9-9. XXY Coordinates 100

10. References **400**

1. General Considerations

1-1. Solvents and Reagents

Unless noted below, commercial reagents were purchased from Sigma Aldrich, Acros Organics, Fischer Scientific, Matrix Scientific, Chem-Impex, Combi-blocks, TCI, Chemshuttle, Oakwood Chemical, Strem Chemicals, Santa Cruz Biotechnology, Spectrum Chemical and/or Alfa Aesar, and used without additional purification. Solvents were purchased from Fisher Scientific, Acros Organics, Alfa Aesar, and Sigma Aldrich. THF, Et_2O, PhH, PhMe, MeOH, and Et_3N were sparged with argon and dried by passing through alumina columns using argon in a Glass Contour solvent purification system. DCM was freshly distilled over calcium hydride under a N_2 atmosphere prior to each use.

1-2. Experimental Procedures

Unless otherwise noted in the experimental procedures, reactions were carried out in flame- or oven-dried glassware under a positive pressure of N_2 in anhydrous solvents using standard Schlenk techniques. Reaction temperatures above room temperature (20–25 °C) were controlled by an IKA® temperature modulator and monitored using liquid-in-glass thermometers. Diazomethane (CH_2N_2) was generated using an Aldrich® diazomethane-generator with System 45™. Reaction progress was monitored by thin-layer chromatography (TLC) on Macherey-Nagel TLC plates (60 Å, F254 indicator). TLC plates were visualized by exposure to ultraviolet light (254 nm), and/or stained by submersion in aqueous potassium permanganate solution ($KMnO_4$), *p*-anisaldehyde, ceric ammonium molybdate, or phosphomolybdic acid stain and heating with a heat gun. Organic solutions were concentrated under reduced pressure on a Heidolph temperature-controlled rotary evaporator equipped with a dry ice/isopropanol condenser. Flash column chromatography was performed with either glass columns using Silicycle silica gel (40–63 µm particle size) or with a Yamazen Smart Flash EPCLC W-Prep 2XY (dual channel) automated flash chromatography system on prefilled, premium, universal columns using ACS grade solvents. All yields refer to chromatographically and spectroscopically (^1H and ^{13}C NMR) pure material.

1-3. Analytical Instrumentation

^1H NMR and ^{13}C NMR data were recorded on Bruker AVQ-400, AVB-400, NEO-500, AV-600 and AV-700 spectrometers using $CDCl_3$ as a solvent, typically at 20–23 °C. Chemical shifts (δ) are reported in ppm relative to the residual solvent signal (δ 7.26 for ^1H NMR & δ 77.16 for ^{13}C NMR in $CDCl_3$). Data for ^1H and ^{13}C spectroscopy are reported as follows: chemical shift (δ ppm), multiplicity (s = singlet, d = doublet, t = triplet, q = quartet, m = multiplet, br = broad, app = apparent), coupling constant (Hz), integration. Melting points were determined using a MEL-TEMP™ apparatus and are uncorrected. High-resolution mass spectra (HRMS) were obtained from the Mass Spectral Facility at the University of California, Berkeley, on a Finnigan/Thermo LTQ-FT instrument (ESI). Data acquisition and processing were performed using the Xcalibur™ software.

2. Reaction Investigations

2-1. Supplementary Table 1: Unsuccessful attempts to achieve intermolecular ring expansion (Approach A)

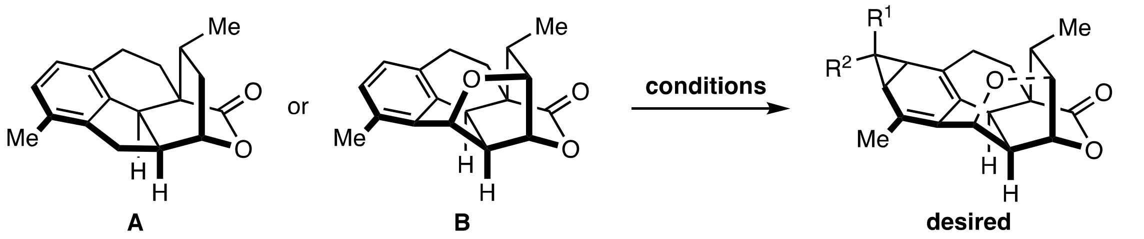

entry	substrate	conditions	conv.
1	**A**	$Rh_2(esp)_2$ (0.05 equiv.), N_2CHCO_2Et (1.0 equiv.), DCE, r.t.	—
2	**A**	$Rh_2(cap)_4$ (0.02 equiv.), N_2CHCO_2Et (3.0 equiv.), DCE, r.t.	trace
3	**A**	$Rh_2(cap)_4$ (1.0 equiv.), N_2CHCO_2Et (3.0 equiv.), DCE, r.t.	trace
4	**A**	$Rh_2(tfa)_4$ (0.05 equiv.), N_2CHCO_2Et (1.0 equiv.), DCE, 60 °C	—
5	**A**	$Rh_2(oct)_4$ (0.05 equiv.), N_2CHCO_2Et (1.0 equiv.), DCE, 80 °C	—
6	**A**	$Cu(acac)_2$ (0.05 equiv.), N_2CHCO_2Et (1.0 equiv.), DCE, 80 °C	—
7	**A**	F_2CBrCO_2Et (3.0 equiv.), K_3PO_4 (3.0 equiv.), MeCN, 90 °C	—
8	**A**	$TMSCF_3$ (4.0 equiv.), NaI (4.0 equiv.), THF, 80 °C	—
9	**B**	N_2CHCO_2Et (1.0 equiv.), DCE, 85 °C	—
10	**B**	$Rh_2(tfa)_4$ (0.05 equiv.), N_2CHCO_2Et (1.0 equiv.), DCE, 40 °C	trace
11	**B**	$Rh_2(oct)_4$ (0.05 equiv.), N_2CHCO_2Et (1.0 equiv.), DCE, 60 °C	—
12	**B**	$Rh_2(esp)_2$ (0.05 equiv.), N_2CHCO_2Et (1.0 equiv.), DCE, 60 °C	—
13	**B**	$Cu(acac)_2$ (0.05 equiv.), N_2CHCO_2Et (1.0 equiv.), DCE, 80 °C	—
14	**B**	F_2CBrCO_2Et (3.0 equiv.), K_3PO_4 (3.0 equiv.), MeCN, 90 °C	trace
15	**B**	$TMSCF_3$ (4.0 equiv.), NaI (4.0 equiv.), THF, 50 °C	—
16	**B**	$TMSCF_3$ (4.0 equiv.), NaI (4.0 equiv.), THF, 120 °C	trace
17	**B**	$TMSCF_3$ (4.0 equiv.), NaI (4.0 equiv.), dioxane, 160 °C	—
18	**B**	TBAT (0.05 equiv.), $TMSCF_3$ (2.5 equiv.), NaI (4.0 equiv.), THF, 120 °C	—
19	**B**	CoTPP (0.05 equiv.), $TMSCF_3$ (4.0 equiv.), NaI (0.2 equiv.), THF, 50 °C	trace
20	**B**	CoTPP (0.05 equiv.), $TMSCF_3$ (4.0 equiv.), NaI (0.2 equiv.), THF, 105 °C	trace
21	**B**	CoTPP (0.05 equiv.), $TMSCF_3$ (4.0 equiv.), NaI (0.2 equiv.), THF, 120 °C	trace
22	**B**	$(MesAcr)BF_4$ (0.05 equiv.), N_2CHCO_2Et (1.0 equiv.), MeCN/TFE, blue LEDs	trace
23	**B**	$(MesAcr)BF_4$ (0.05 equiv.), N_2CHCO_2Et (1.5 equiv.), MeCN/TFE, blue LEDs	complex mixture
24	**B**	$BnNEt_3Cl$ (0.2 equiv.), CCl_3CO_2Na (3.0 equiv.), $CHCl_3$, 90 °C	—

2-2. Supplementary Table 2: Initial attempts at the Büchner–Curtius–Schlotterbeck (BCS) reaction using CH_2N_2

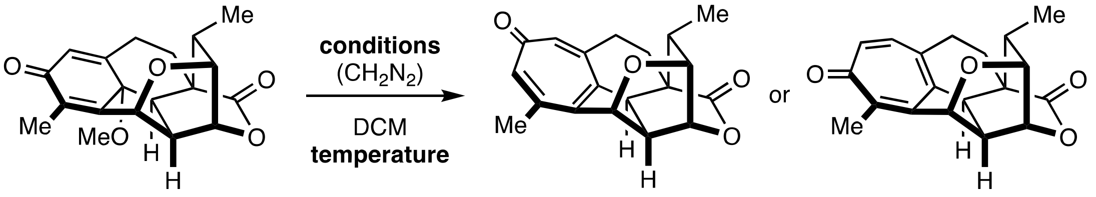

20 **4** **25**

entry	conditions	temperature	conv.	note
1	BF_3•OEt_2 (1.2 equiv.), CH_2N_2 (50 equiv.)[a]	–78 °C	—	—
2	BF_3•OEt_2 (1.2 equiv.), CH_2N_2 (500 equiv.)[a]	–78 °C to r.t.	trace	—
3	BF_3•OEt_2 (1.2 equiv.), CH_2N_2 (500 equiv.)[a]	r.t.	trace	—
4	BF_3•OEt_2 (1.2 equiv.), CH_2N_2 (10 equiv.)[b]	–78 °C to r.t.	trace	transferred with a syringe
5	$AlCl_3$ (3.0 equiv.), CH_2N_2 (5.0 equiv.)[a]	–60 °C	—	—
6	$AlCl_3$ (3.0 equiv.), CH_2N_2 (20 equiv.)[a]	–60 °C	—	—
7	$AlCl_3$ (3.0 equiv.), CH_2N_2 (50 equiv.)[a]	–60 °C	trace	—
8	$AlCl_3$ (3.0 equiv.), CH_2N_2 (50 equiv.)[a]	–60 °C to r.t.	trace	—
9	$AlCl_3$ (3.0 equiv.), CH_2N_2 (5.0 equiv.)[b]	–60 °C	trace	transferred with a syringe
10	$AlCl_3$ (3.0 equiv.), CH_2N_2 (20 equiv.)[b]	–60 °C	trace	transferred with a syringe
11	$AlCl_3$ (3.0 equiv.), CH_2N_2 (50 equiv.)[b]	–60 °C	trace	transferred with a syringe
12	$AlCl_3$ (3.0 equiv.), CH_2N_2 (50 equiv.)[b]	–60 °C to r.t.	trace	transferred with a syringe
13	$AlCl_3$ (3.0 equiv.), CH_2N_2 (5.0 equiv.)[b]	–60 °C	—	transferred with a pipette[c]
14	$AlCl_3$ (3.0 equiv.), CH_2N_2 (20 equiv.)[b]	–60 °C	—	transferred with a pipette[c]
15	$AlCl_3$ (3.0 equiv.), CH_2N_2 (50 equiv.)[b]	–60 °C	trace	transferred with a pipette[c]
16	$AlCl_3$ (3.0 equiv.), CH_2N_2 (50 equiv.)[b]	–60 °C to r.t.	trace	transferred with a pipette[c]

[a]The reaction was performed in a diazomethane generator using Diazald®. [b]Diazomethane was added as a freshly prepared solution in DCM (0.2 M, prepared from Diazald®). [c]The tip of the glass pipette was held in a flame to round it off.

2-3. Supplementary Table 3: Selected optimization of the BCS reaction using $TMSCHN_2$

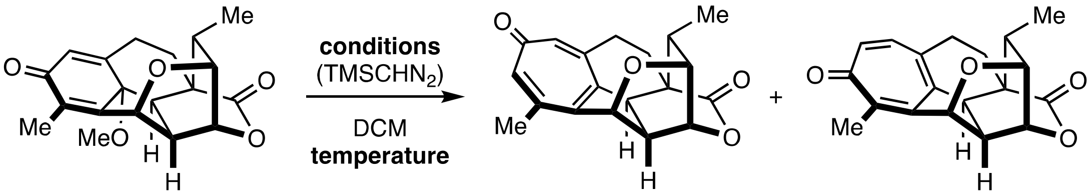

20 **4** **25**

entry	conditions[a]	temperature	4[b]	25[b]	ratio	conv.[c]
1	BF_3•OEt_2 (1.2 equiv.), $TMSCHN_2$ (2.0 equiv.)	–78 °C	9%	57%	1:6.3	100%
2[d]	BF_3•OEt_2 (1.2 equiv.), $TMSCHN_2$ (2.0 equiv.)	–78 °C	trace	12%	—	43%
3[d]	BF_3•OEt_2 (1.2 equiv.), $TMSCHN_2$ (3.0 equiv.)	–78 °C	trace	20%	—	61%
4[e]	BF_3•OEt_2 (1.2 equiv.), $TMSCHN_2$ (3.0 equiv.)	–78 °C	trace	23%	—	59%
5	BF_3•OEt_2 (1.2 equiv.), $TMSCHN_2$ (3.0 equiv.)[f]	–60 °C	19%	70%	1:3.7	100%
6	BF_3•OEt_2 (1.2 equiv.), $TMSCHN_2$ (3.0 equiv.)	–40 °C	13%	45%	1:3.5	100%[g]
7	$B(C_6F_5)_3$ (1.2 equiv.), $TMSCHN_2$ (3.0 equiv.)	–78 °C	—	—	—	ND
8	$B(C_6F_5)_3$ (1.2 equiv.), $TMSCHN_2$ (3.0 equiv.)	–60 °C	trace	trace	—	trace
9	$B(C_6F_5)_3$ (3.0 equiv.), $TMSCHN_2$ (3.0 equiv.)	–60 °C	trace	trace	—	trace
10	$B(C_6F_5)_3$ (1.2 equiv.), $TMSCHN_2$ (6.0 equiv.)	–60 °C	trace	trace	—	trace
11	$B(C_6F_5)_3$ (1.2 equiv.), $TMSCHN_2$ (3.0 equiv.)	–40 °C	trace	trace	1:2.0[h]	trace
12	$B(C_6F_5)_3$ (1.2 equiv.), $TMSCHN_2$ (3.0 equiv.)[i]	–78 to 0 °C	trace	trace	—	50%[j]
13	$Sc(OTf)_3$ (0.5 equiv.), $TMSCHN_2$ (3.0 equiv.)[i]	–78 to 0 °C	—	—	—	ND
14	$Sc(OTf)_3$ (0.5 equiv.), $TMSCHN_2$ (3.0 equiv.)	0 °C	—	—	—	ND
15	$Sc(OTf)_3$ (1.2 equiv.), $TMSCHN_2$ (3.0 equiv.)	0 °C	—	—	—	ND
16	$InBr_3$ (1.5 equiv.), $TMSCHN_2$ (3.0 equiv.)[i]	–78 to 0 °C	—	—	—	ND
17	$CeBr_3$ (1.5 equiv.), $TMSCHN_2$ (3.0 equiv.)[i]	–78 to 0 °C	—	—	—	ND
18	$Zn(OTf)_2$ (1.2 equiv.), $TMSCHN_2$ (3.0 equiv.)[k]	–60 to r.t.	—	—	—	ND
19	$Zn(OTf)_2$ (1.2 equiv.), $TMSCHN_2$ (3.0 equiv.)[i]	0°C to r.t.	—	—	—	ND
20	$Ga(OTf)_3$ (1.2 equiv.), $TMSCHN_2$ (3.0 equiv.)[f]	–60 °C	—	—	—	ND
21	$In(OTf)_3$ (1.2 equiv.), $TMSCHN_2$ (3.0 equiv.)[f]	–60 °C	—	—	—	ND
22	$ZrCl_4$ (1.2 equiv.), $TMSCHN_2$ (3.0 equiv.)	–60 to –40 °C	—	—	—	ND
23	$PhBCl_2$ (1.2 equiv.), $TMSCHN_2$ (3.0 equiv.)[f]	–60 °C	trace	trace	—	trace
24	$SbCl_5$ (0.44 equiv.), $TMSCHN_2$ (3.0 equiv.)	–78 to –50 °C	12%	19%	1:1.6	86%[g]
25	$AlMe_3$ (1.2 equiv.), $TMSCHN_2$ (3.0 equiv.)	–78 to –50 °C	9%	15%	1:1.7	30%
26	$AlMe_3$ (1.2 equiv.), $TMSCHN_2$ (3.0 equiv.)	–78 to –30°C	7%	11%	1:1.6	65%[g]
27	$AlMe_3$ (1.2 equiv.), $TMSCHN_2$ (3.0 equiv.)[l]	–78 to –50 °C	18%	19%	1:1.1	61%[j]

28	$AlMe_3$ (2.5 equiv.), $TMSCHN_2$ (3.0 equiv.)	–78 to –50 °C	15%	20%	1:1.3	42%
29	$AlMe_3$ (3.5 equiv.), $TMSCHN_2$ (3.0 equiv.)	–78 to –50 °C	19%	23%	1:1.2	48%
30	$AlMe_3$ (3.5 equiv.), $TMSCHN_2$ (7.0 equiv.)[i]	–78 to –50 °C	20%	23%	1:1.2	53%
31	$AlBr_3$ (1.2 equiv.), $TMSCHN_2$ (3.0 equiv.)[f]	–60 °C	trace	trace	1:2.2[h]	82%[g]
32	$AlBr_3$ (3.5 equiv.), $TMSCHN_2$ (3.0 equiv.)[f]	–60 °C	23%	49%	1:2.1	100%[g]
33	$AlCl_3$ (1.2 equiv.), $TMSCHN_2$ (3.0 equiv.)	–65 °C	27%	38%	1:1.6	100%
34	$AlCl_3$ (1.2 equiv.), $TMSCHN_2$ (3.0 equiv.)	–50 °C	23%	32%	1:1.4	100%[g]
35	$AlCl_3$ (1.2 equiv.), $TMSCHN_2$ (3.0 equiv.)	–40 °C	16%	29%	1:1.8	100%[g]
36	$AlCl_3$ (3.0 equiv.), $TMSCHN_2$ (3.0 equiv.)	–60 °C	32%	48%	1:1.5	100%
37	$AlCl_3$ (5.0 equiv.), $TMSCHN_2$ (3.0 equiv.)	–60 °C	29%	46%	1:1.6	100%
38	$AlCl_3$ (3.0 equiv.), $TMSCHN_2$ (5.0 equiv.)	–60 °C	**37%**	**46%**	**1:1.2**	100%
39	Et_2AlCl (1.2 equiv.), $TMSCHN_2$ (3.0 equiv.)	–60 °C	7%	10%	1:1.7	59%
40	$Al(OTf)_3$ (1.2 equiv.), $TMSCHN_2$ (3.0 equiv.)	–60 °C	—	—	—	ND

[a]The Lewis acid was pre-stirred with the starting material for 10 min before dropwise addition of $TMSCHN_2$ (0.2 M in hexanes/DCM = 1:9); reaction time: 2 h. [b]Isolated yields. [c]Determined from reisolated starting material (ND: not determined). [d]Rapid addition of $TMSCHN_2$. [e]4 Å MS was used as an additive. [f]reaction time: 1 h. [g]A significant amount of decomposed material was observed. [h]Determined by ^{1}H NMR. [i]Reaction time: 3 h. [j]Additional unidentified trace products were also formed. [k]Reaction time: 5 h. [l]A 2.0 M solution of $TMSCHN_2$ in hexanes was used.

3. Experimental Procedures and Characterization Data of New Compounds

Methyl-ceforalide H (20)

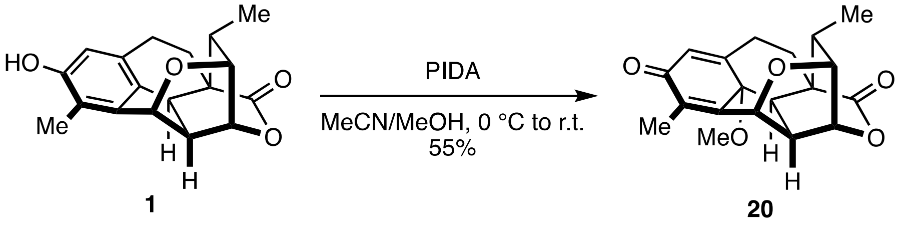

To a solution of **1** (25.0 mg, 83.8 µmol, 1.0 equiv.) in MeCN/MeOH (1:1 v/v, 838 µL) was added phenyliodine(III) diacetate (PIDA; 32.4 mg, 101 µmol, 1.2 equiv.) at 0 °C. After stirring at room temperature for 5 h, the reaction mixture was quenched with sat. aq. $NaHCO_3$ (2 mL), diluted with H_2O (3 mL) and extracted with DCM (3 x 5 mL). The combined organic phase was dried over Na_2SO_4 and concentrated in vacuo. The resulting residue was purified by silica gel flash column chromatography (hexanes/EtOAc = 2:1), yielding methyl-ceforalide H (**20**) (15.2 mg, 46.3 µmol, 55%) as a colorless solid.

Rf-value: 0.54 (hexanes/EtOAc = 1:1)

Melting Point: 181 °C

HRMS (m/z): ESI $[M+Na]^+$ calculated for $C_{19}H_{20}O_5Na$: 351.1203, found: 351.1203.

^{1}H NMR (600 MHz, $CDCl_3$): δ 6.15 (d, *J* = 1.9 Hz, 1H), 5.20 (dd, *J* = 6.0, 1.2 Hz, 1H), 5.01 (t, *J* = 5.9 Hz, 1H), 3.89 (d, *J* = 5.5 Hz, 1H), 3.42 (dt, *J* = 9.1, 6.1 Hz, 1H), 2.97 – 2.89 (m, 4H), 2.68 (d, *J* = 9.1 Hz, 1H), 2.53 (dddd, *J* = 13.3, 11.3, 9.2, 2.0 Hz, 1H), 2.35 (dd, *J* = 13.5, 7.8 Hz, 1H), 2.01 (s, 3H), 1.81 (q, *J* = 7.5 Hz, 1H), 1.62 (ddd, *J* = 14.6, 11.4, 7.6 Hz, 1H), 0.89 (d, *J* = 7.5 Hz, 3H).

^{13}C NMR (151 MHz, $CDCl_3$): δ 188.42, 173.40, 157.29, 156.63, 137.32, 126.66, 80.48, 79.87, 79.40, 77.78, 49.82, 49.72, 42.36, 40.73 (2C), 27.25, 26.78, 16.36, 12.70.

Harringtonolide (4) and *iso*-harringtonolide (25)

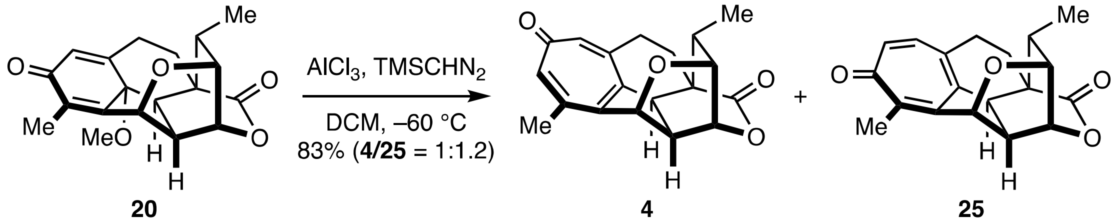

A flame-dried vial with a magnetic stir bar was transferred to a glovebox and charged with $AlCl_3$ (12.2 mg, 91.4 µmol, 3.0 equiv.). The vial was sealed with a septa cap and removed from the glovebox. The vial was evacuated and backfilled with N_2 three times and cooled to –60 °C. Freshly distilled DCM (50 µL) was added, and the suspension was stirred at –60 °C for 5 min. A solution of methyl-ceforalide H (**20**) (10.0 mg, 30.5 µmol, 1.0 equiv.) in freshly distilled DCM (250 µL) was added and stirred at –60 °C for 10 min to give a grayish suspension. TMS-diazomethane (0.2 M, prepared from a 2.0 M solution in hexanes diluted with freshly distilled DCM, 760 µL, 152 µmol, 5.0 equiv.) was added over 2 min resulting in a yellowish solution. The mixture was stirred at –60 °C for 3 h and quenched with sat. aq. $NaHCO_3$ (500 µL). The suspension was diluted with H_2O (2 mL) and extracted with DCM (3 x 3 mL). The combined organic layers were dried over anhydrous Na_2SO_4, and the solvent was removed under reduced pressure. The residue was purified by preparative TLC (hexanes/EtOAc = 1:3), yielding harringtonolide (**4**) (3.5 mg, 11.3 µmol, 37%) as a colorless solid and *iso*-harringtonolide (**25**) (4.3 mg, 13.9 µmol, 46%) as a colorless solid.

Scale-up:

The reaction was also performed on a 100 mg scale to give harringtonolide (**4**) (27.9 mg, 89.9 µmol, 30%) and *iso*-harringtonolide (**25**) (42.3 mg, 136 µmol, 45%) as colorless solids. It is important to note that the ratio of **4**:**25** was very dependent on the rate of addition of $TMSCHN_2$. It was therefore important to maintain an addition time of 2–3 min to avoid forming proportionally more *iso*-harringtonolide (**25**).

Harringtonolide (4)

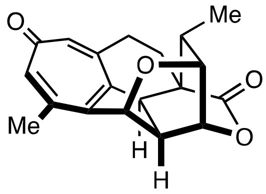

4

Rf-value: 0.13 (hexanes/EtOAc = 1:3)

Melting Point: 258–261 °C (decomposed, turned black)

HRMS (m/z): ESI $[M+H]^+$ calculated for $C_{19}H_{19}O_4$: 311.1278, found: 311.1275.

^{1}**H NMR** (600 MHz, $CDCl_3$): δ 6.96 (s, 1H), 6.89 (t, *J* = 2.1 Hz, 1H), 5.35 (d, *J* = 5.2 Hz, 1H), 5.20 (t, *J* = 5.1 Hz, 1H), 3.98 (d, *J* = 5.7 Hz, 1H), 3.40 – 3.36 (m, 2H), 2.88 – 2.81 (m, 2H), 2.62 (dd, *J* = 14.0, 6.2 Hz, 1H), 2.36 (d, *J* = 1.2 Hz, 3H), 1.76 (q, *J* = 7.6 Hz, 1H), 1.33 – 1.27 (m, 1H), 0.89 (d, *J* = 7.6 Hz, 3H).

^{13}**C NMR** (151 MHz, $CDCl_3$): δ 186.61, 173.62, 145.97, 145.79, 145.10, 143.66, 141.76, 139.41, 86.19, 80.15, 79.88, 50.12, 45.97, 41.95, 40.18, 32.49, 24.00, 22.57, 14.90.

***Iso*-harringtonolide (25)**

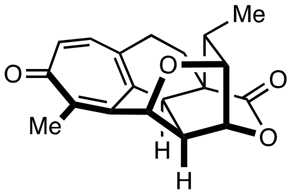

25

Rf-value: 0.27 (hexanes/EtOAc = 1:3)

Melting Point: 254–259 °C (decomposed, turned black)

HRMS (m/z): ESI $[M+H]^+$ calculated for $C_{19}H_{19}O_4$: 311.1278, found: 311.1276.

^{1}**H NMR** (600 MHz, $CDCl_3$): δ 7.02 (d, *J* = 12.1 Hz, 1H), 6.91 (d, *J* = 12.1 Hz, 1H), 5.48 (d, *J* = 5.1 Hz, 1H), 5.22 (t, *J* = 5.7 Hz, 1H), 3.98 (d, *J* = 5.7 Hz, 1H), 3.41 (dt, *J* = 9.6, 5.4 Hz, 1H), 3.06 (d, *J* = 9.6 Hz, 1H), 2.80 – 2.72 (m, 2H), 2.71 – 2.63 (m, 1H), 2.35 (d, *J* = 1.2 Hz, 3H), 1.58 (q, *J* = 7.6 Hz, 1H), 1.38 – 1.27 (m, 1H), 0.87 (d, *J* = 7.6 Hz, 3H).

^{13}**C NMR** (151 MHz, $CDCl_3$): δ 187.53, 173.84, 148.83, 145.89, 145.55, 139.75, 137.88, 137.80, 84.91, 79.99, 78.64, 49.00, 45.89, 42.39, 41.11, 31.21, 21.86, 17.77, 14.73.

4. Large-Scale Synthesis of Cephanolide A

Supplementary Figure 1: 12-step total synthesis sequence

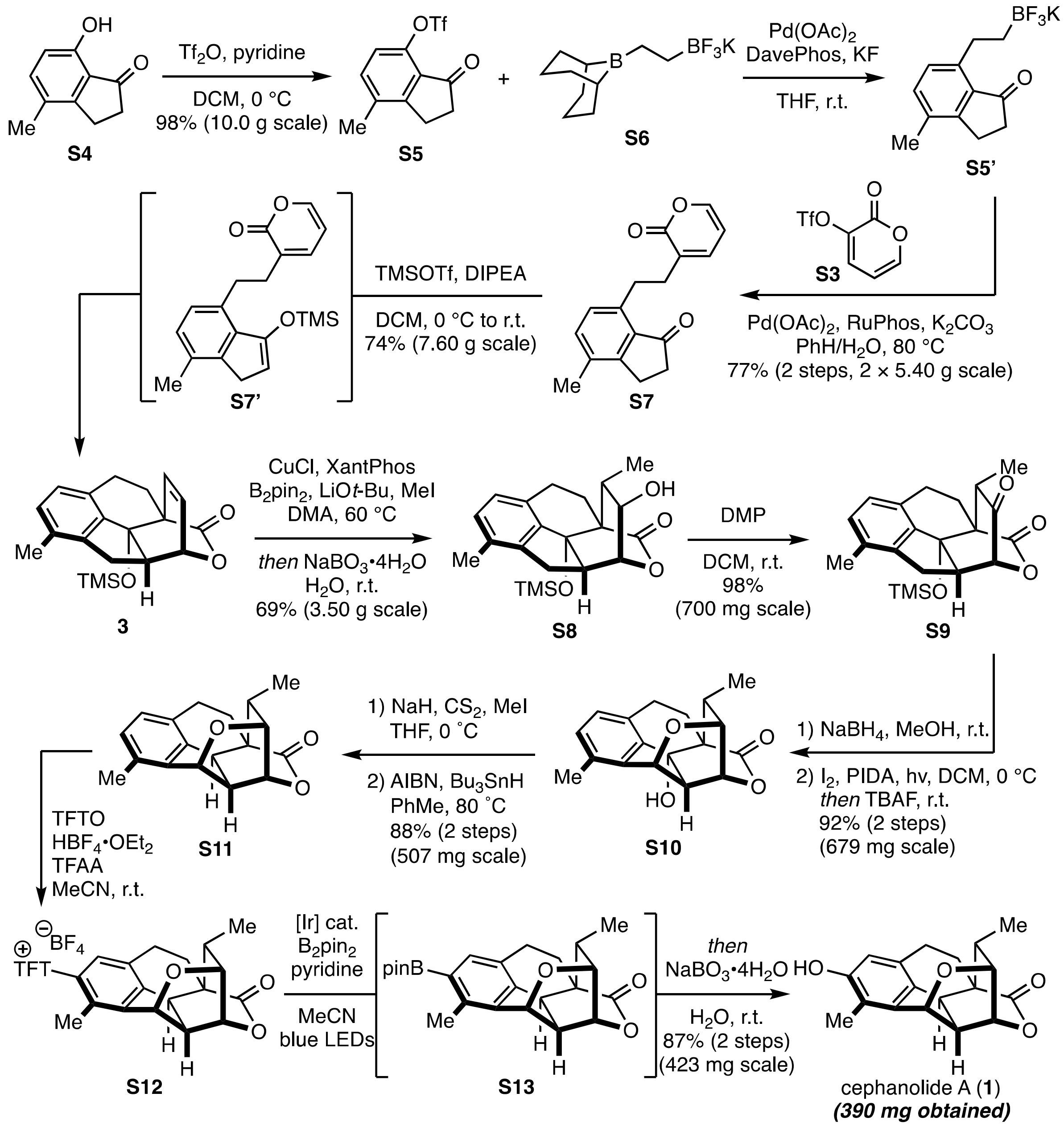

3-Hydroxy pyranone S2

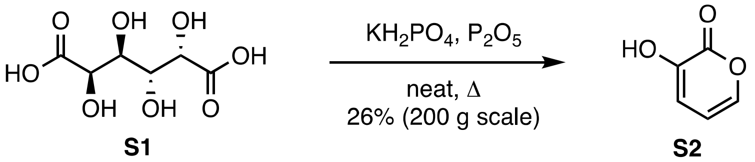

Mucic acid (**S1**) (200 g, 952 mmol, 1.0 equiv.) was thoroughly mixed with KH_2PO_4 (200 g, 1.46 mol, 1.5 equiv.) and P_2O_5 (120 g, 420 mmol, 0.4 equiv.) and ground in a mortar before transferring the heterogenous mixture into a 1 L one-necked round-bottomed flask. A three-necked 1 L round-bottomed flask was connected using a U-shaped glass tube. To the three-necked flask, an open reflux condenser was attached, and the residual joint was sealed with a glass stopper. The three-necked 1 L round-bottomed flask was immersed into a Dewar flask filled with dry ice **(Note: only solid dry ice was used to avoid flammable convection liquids)**. The mixture in the single-necked round-bottomed flask was carefully pyrolyzed with a bunsen flame (by heating from top to bottom) resulting in yellow steam collecting in the condensation flask. After the evolution of yellow steam had ceased (typically 1 h), the apparatus was left to cool to room temperature, before the brown/yellow solid residue in the one-necked 1 L flask was dissolved in 1 L of Et_2O and the acidity adjusted to pH ~6 (as judged using pH-paper) with 1 M KOH. The resulting solution was subjected to continuous liquid/liquid extraction using a Kutscher-Steudl apparatus (containing 1 L of Et_2O in the extraction flask) for 24 h. After this time, the resulting solution was dried over $MgSO_4$, filtered and concentrated *in vacuo*, yielding **S2** (27.5 g, 246 mmol, 26%) as a yellow solid. The analytical data were consistent with the reported values.[1]

Rf-value: 0.22 (PhMe:EtOAc = 5:1; $KMnO_4$)

^{1}H NMR (400 MHz, $CDCl_3$): δ 7.15 (dd, *J* = 5.2, 1.7 Hz, 1H), 6.68 (dd, *J* = 7.1, 1.7 Hz, 1H), 6.51 (s, 1H), 6.21 (dd, *J* = 7.1, 5.2 Hz, 1H).

Pyranone 3-triflate S3

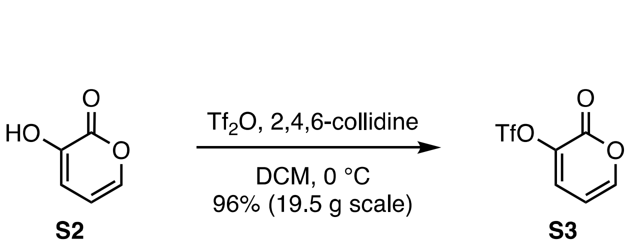

To a solution of **S2** (19.5 g, 174 mmol, 1.0 equiv.) in DCM (500 mL) was added 2,4,6-collidine (25.2 mL, 191 mmol, 1.1 equiv.) and Tf_2O (30.7 mL, 183 mmol, 1.05 equiv.) at 0 °C. After stirring at 0 °C for 50 min, the reaction mixture was quenched with 2 M HCl (500 mL) and extracted with DCM (3 x

400 mL). The combined organic phase was washed with 2 M HCl (1 L) and brine (1 L), dried over Na_2SO_4, filtered and concentrated *in vacuo*. The resulting residue was purified by a short silica plug (eluted with PhMe/EtOAc = 50:1), yielding **S3** (40.8 g, 167 mmol, 96%) as a yellow solid. The analytical data were consistent with the reported values.[2]

Rf-value: 0.41 (PhMe:EtOAc = 5:1; $KMnO_4$)

^{1}H NMR (400 MHz, $CDCl_3$): δ 7.52 (dd, *J* = 5.1, 1.8 Hz, 1H), 7.36 (dd, *J* = 7.2, 1.8 Hz, 1H), 6.32 (dd, *J* = 7.2, 5.2 Hz, 1H).

Indanone 7-triflate S5

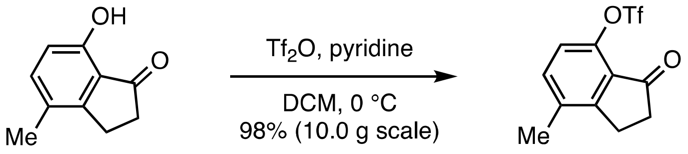

S4 **S5**

To a solution of **S4** (10.0 g, 61.7 mmol, 1.0 equiv.) in DCM (300 mL) was added pyridine (11.6 mL, 142 mmol, 2.3 equiv.) and Tf_2O (11.4 mL, 67.8 mmol, 1.1 equiv.) dropwise at 0 °C. After stirring at 0 °C for 1 h, the reaction mixture was quenched with 2 M HCl (30 mL) and extracted with with DCM (3 x 100 mL). The combined organic phase was washed with 2 M HCl (100 mL) and brine (50 mL), dried over Na_2SO_4, filtered, and concentrated *in vacuo*. The resulting residue was purified by a short silica plug (eluted with PhMe/EtOAc = 50:1), yielding **S5** (17.7 g, 60.2 mmol, 98% yield) as a slightly yellow solid. The analytical data were consistent with the reported values.[3]

Rf-value: 0.26 (PhMe:EtOAc = 5:1; *p*-anisaldehyde)

HRMS (m/z): ESI [M] calculated for $C_{11}H_9O_4SF_3$ $[M]^+$: 294.0174, found $[M]^+$: 294.0175.

^{1}H NMR (400 MHz, $CDCl_3$): δ 7.44 (d, *J* = 8.1 Hz, 1H), 7.09 (d, *J* = 8.1 Hz, 1H), 3.08 – 3.02 (m, 2H), 2.80 – 2.74 (m, 2H), 2.38 (s, 3H).

^{13}C NMR (101 MHz, $CDCl_3$): δ 202.7, 156.4, 143.3, 136.9, 136.2, 128.7, 120.2, 118.8 (q, *J* = 320.7 Hz), 36.6, 24.9, 17.6.

Indanone 7-ethylpyranone S7

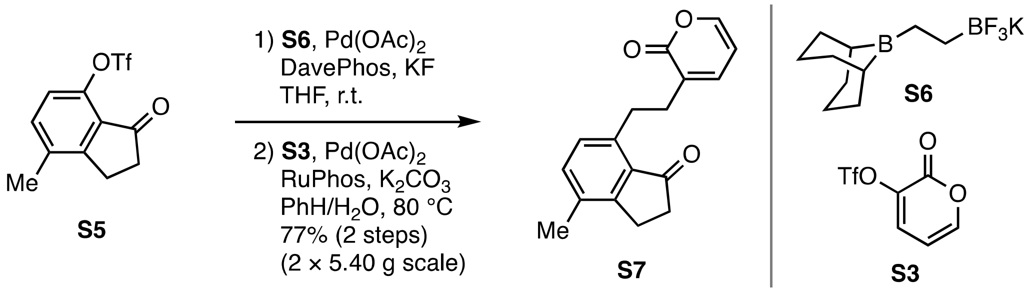

Two 200 mL flasks each containing **S5** (5.40 g, 18.4 mmol, 1.0 equiv.), $Pd(OAc)_2$ (82.6 mg, 368 µmol, 2 mol%), DavePhos (290 mg, 736 µmol, 4 mol%) and KF (3.53 g, 60.7 mmol, 3.3 equiv.) were evacuated and backfilled with N_2 three times. To each mixture was added THF (80.0 mL) at room temperature. The resulting orange suspension was subjected to three freeze-pump-thaw cycles. To each solution was added **S6** (0.5 M, 40.5 mL, 20.2 mmol, 1.1 equiv.) at room temperature *via* a transfer-canula, at which point the solution turned dark brown. After stirring at room temperature for 48 h, the resulting beige suspension was poured into a 1 L Erlenmeyer flask containing Et_2O (500 mL). After stirring for an additional 15 min, the resulting solids were collected by vacuum filtration. The beige solid residue was washed with Et_2O (2 x 200 mL) and dried under vacuum. This crude solid was used in the next reaction without further purification.

To two 500 mL flasks each containing the crude solid, **S3** (8.98 g, 36.8 mmol, 2.0 equiv.), $Pd(OAc)_2$ (207 mg, 920 µmol, 5 mol%), RuPhos (1.03 g, 2.21 mmol, 12 mol%) and K_2CO_3 (7.63 g, 55.2 mmol, 3.0 equiv.) was added PhMe/H_2O (5:1 v/v, 300 mL) at room temperature. After stirring at 80 °C for 48 h, the reaction mixture was allowed to cool down to room temperature, diluted with H_2O (250 mL) and extracted with EtOAc (2 x 300 mL). The combined organic phase was washed with brine (300 mL), dried over $MgSO_4$ and concentrated *in vacuo*. Each crude residue was combined and purified by flash column chromatography (PhMe/EtOAc = 12:1 to 10:1) yielding **S7** (7.60 g, 28.3 mmol, 77% over 2 steps) as an off-white solid. The analytical data were consistent with the reported values.[3]

Rf-value: 0.28 (PhMe:EtOAc = 5:1; *p*-anisaldehyde)

HRMS (m/z): ESI [M] calculated for $C_{17}H_{16}O_3$+Na [M+Na]$^+$: 291.0992, found [M+Na]$^+$: 291.0993.

^{1}H NMR (400 MHz, $CDCl_3$): δ 7.38 (dd, *J* = 5.2, 2.1 Hz, 1H), 7.27 (d, *J* = 7.3 Hz, 1H), 7.18 (dt, *J* = 6.9, 1.4 Hz, 1H), 7.06 (d, *J* = 7.5 Hz, 1H), 6.14 (dd, *J* = 6.5, 5.1 Hz, 1H), 3.28 – 3.24 (m, 2H), 3.00 – 2.97 (m, 2H), 2.74 – 2.70 (m, 2H), 2.70 – 2.67 (m, 2H), 2.31 (s, 3H).

^{13}C NMR (126 MHz, $CDCl_3$): δ 208.2, 163.0, 155.3, 149.4, 139.0, 138.8, 134.8, 133.71, 133.69, 129.8, 128.7, 106.4, 36.8, 31.8, 29.9, 24.4, 17.5.

Cycloadduct 3

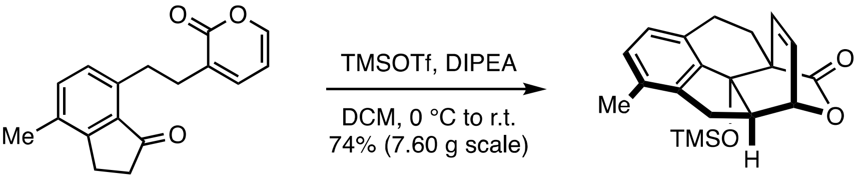

S7 **3**

To a solution of **S7** (7.60 g, 28.3 mmol, 1.0 equiv.) in DCM (300 mL) was added DIPEA (10.9 mL, 62.3 mmol, 2.2 equiv.) and TMSOTf (10.7 mL, 59.4 mmol, 2.1 equiv.) dropwise at 0 °C. After stirring at room temperature for 24 h, the reaction mixture was quenched with sat. aq. $NaHCO_3$ (300 mL) at 0 °C and extracted with DCM (2 x 300 mL). The combined organic phase was washed with brine (500 mL), dried over Na_2SO_4, filtered and concentrated *in vacuo*. The crude residue was purified by a short silica plug (eluted with DCM), yielding **3** (7.09 g, 20.8 mmol, 74%) as a yellow solid. The analytical data were consistent with the reported values.[3]

Rf-value: 0.65 (PhMe:EtOAc = 5:1; *p*-anisaldehyde)

HRMS (m/z): ESI [M] calculated for $C_{20}H_{24}O_3Si$+Na $[M+Na]^+$: 363.1387, found $[M+Na]^+$: 363.1388.

^{1}H NMR (500 MHz, $CDCl_3$): δ 7.00 (d, *J* = 7.5 Hz, 1H), 6.87 (d, *J* = 7.3 Hz, 1H), 6.06 (dd, *J* = 7.7, 4.8 Hz, 1H), 5.46 (dd, *J* = 7.6, 2.1 Hz, 1H), 5.21 (td, *J* = 4.8, 2.1 Hz, 1H), 3.32 (dd, *J* = 17.2, 9.2 Hz, 1H), 3.13 – 3.07 (m, 2H), 2.87 (ddd, *J* = 13.8, 10.9, 4.6 Hz, 1H), 2.75 (ddd, *J* = 17.0, 9.6, 4.6 Hz, 1H), 2.16 (s, 3H), 2.09 (dd, *J* = 17.1, 3.1 Hz, 1H), 2.03 (ddd, *J* = 13.6, 9.5, 3.7 Hz, 1H), -0.13 (s, 9H).

^{13}C NMR (101 MHz, $CDCl_3$): δ 173.3, 142.1, 140.8, 137.2, 132.5, 131.4, 130.9, 128.1, 126.4, 85.4, 77.3, 54.5, 50.4, 32.4, 23.4, 20.7, 18.3, 1.0 (3C).

Methyl alcohol S8

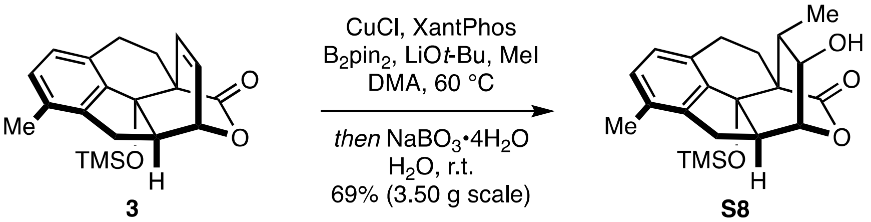

3 **S8**

A 100 mL flask containing CuCl (306 mg, 3.09 mmol, 30 mol%), XantPhos (1.97 g, 3.40 mmol, 33 mol%) and B_2pin_2 (5.23 g, 20.6 mmol, 2.0 equiv.) was evacuated and backfilled with Ar three times. To the flask was added DMA (100 mL) and the resulting solution was cooled to 0 °C. To the mixture was added LiO*t*-Bu (1.0 M in hexanes, 20.6 mL, 20.6 mmol, 2.0 equiv.) dropwise and the resulting

solution was stirred at room temperature for 15 min. To the reaction mixture was added a solution of **3** (3.50 g, 10.3 mmol, 1.0 equiv.) in DMA (10.0 mL), followed by MeI (a few drops, ca. 1.0 equiv.) using a syringe pump. Upon complete addition, the reaction mixture was immediately warmed up to 60 °C, while MeI (19.2 mL, 30.9 mmol, 30.0 equiv.) was continuously added dropwise directly to the stirring reaction solution (avoiding the walls of the flask) over 1.5 h. Upon complete addition, the reaction mixture was stirred at 60 °C for 1 h, and then allowed to cool to room temperature. To the reaction mixture was added H_2O (100 mL) and $NaBO_3$•$4H_2O$ (7.92 g, 51.5 mmol, 5.0 equiv.) at room temperature. After stirring vigorously at room temperature for 2 h, the reaction mixture was quenched with sat. aq. $Na_2S_2O_3$ (300 mL) and extracted with Et_2O (300 mL). The organic phase was washed with brine (300 mL), dried over Na_2SO_4 and concentrated *in vacuo*. The resulting residue was purified by silica gel flash column chromatography (hexanes:EtOAc = 3:1), yielding **S8** (2.63 g, 7.06 mmol, 69%) as a white solid. The analytical data were consistent with the reported values.[4]

Rf-value: 0.50 (hexanes/EtOAc = 1:1, stained with *p*-anisaldehyde)

HRMS (m/z): ESI $[M+Na]^+$ calculated for $C_{21}H_{28}O_4SiNa$: 395.1649, found: 395.1648.

^{1}H NMR (400 MHz, $CDCl_3$): δ 7.08 (d, *J* = 7.5 Hz, 1H), 6.92 (d, *J* = 7.5 Hz, 1H), 4.74 (d, *J* = 5.3 Hz, 1H), 3.55 (d, *J* = 9.4 Hz, 1H), 3.35 (dd, *J* = 17.5, 9.5 Hz, 1H), 3.04 – 2.95 (m, 2H), 2.68 – 2.53 (m, 3H), 2.24 (s, 3H), 1.93 (dd, *J* = 13.9, 8.3 Hz, 1H), 1.28 – 1.19 (m, 1H), 0.86 (d, *J* = 7.6 Hz, 3H), -0.17 (s, 9H).

^{13}C NMR (151 MHz, $CDCl_3$): δ 174.3, 140.1, 139.4, 133.1, 131.9, 131.5, 126.9, 84.6, 84.4, 65.0, 52.8, 48.5, 35.4, 30.3, 22.8, 18.4, 17.8, 10.6, 1.1 (3C).

α-Methyl ketone S9

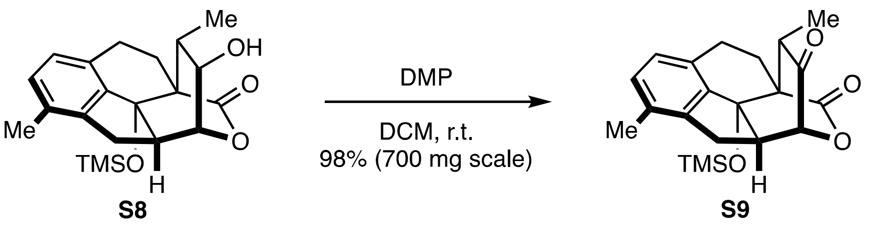

To a solution of **S8** (700 mg, 1.88 mmol) in DCM (47 mL) was added Dess-Martin periodinane (DMP, 3.98 g, 9.39 mmol, 5.0 equiv.) at room temperature. After stirring for 5 h, the reaction mixture was quenched with saturated aq. $Na_2S_2O_3$ (50 mL) and diluted with H_2O (50 mL). The organic layer was separated, and the aqueous layer was extracted with Et_2O (3 x 75 mL). The combined organic layers

were washed with brine (100 mL) and dried over anhydrous Na_2SO_4. The solvent was removed under reduced pressure and the residue was purified by silica gel flash column chromatography (hexanes/EtOAc = 20:1 to 10:1), yielding **S9** (679 mg, 1.83 mmol, 98%) as a colorless solid. The analytical data were consistent with the previously reported values.[1]

Rf-value: 0.50 (hexanes:EtOAc = 4:1; *p*-anisaldehyde)

HRMS (m/z): ESI [M] calculated for $C_{21}H_{26}O_4Si$+Na [M+Na]$^+$: 393.1493, found [M+Na]$^+$: 393.1492.

^{1}H NMR (400 MHz, $CDCl_3$): δ 7.07 (d, *J* = 7.5 Hz, 1H), 6.93 (d, *J* = 7.5 Hz, 1H), 4.64 (d, *J* = 5.7 Hz, 1H), 3.37 (dd, *J* = 17.4, 9.6 Hz, 1H), 3.26 (ddd, *J* = 9.2, 5.7, 2.7 Hz, 1H), 3.05 (dd, *J* = 16.9, 9.9 Hz, 1H), 2.73 – 2.57 (m, 2H), 2.37 (dd, *J* = 17.4, 2.8 Hz, 1H), 2.18 (s, 3H), 1.94 – 1.87 (m, 1H), 1.20 (q, *J* = 7.6 Hz, 1H), 1.03 (d, *J* = 7.6 Hz, 3H), -0.12 (s, 9H).

^{13}C NMR (126 MHz, $CDCl_3$): δ 207.0, 172.0, 140.0, 138.2, 132.7, 132.5, 132.0, 127.1, 84.8, 84.3, 54.0, 49.5, 42.4, 30.0, 22.8, 18.4, 17.5, 12.9, 1.1 (3C).

Hexacyclic alcohol S9

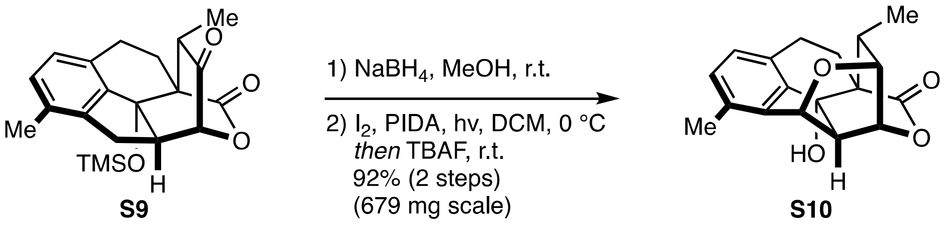

To a solution of **S9** (679 mg, 1.83 mmol) in MeOH (18.3 mL) was added $NaBH_4$ (1.04 g, 27.5 mmol, 1.5 equiv.). After stirring at room temperature for 30 min, the mixture was quenched with acetone (1.00 mL), diluted with sat. aq. $NaHCO_3$ (100 mL), and extracted with EtOAc (100 mL). The organic phase was dried over Na_2SO_4, filtered and concentrated *in vacuo*. This crude material was used in the next reaction without further purification.

To a solution of the crude material in DCM (1.83 mL) was added I_2 (464 mg, 1.83 mmol, 1.0 equiv.) and PIDA (1.30 g, 4.03 mmol, 2.2 equiv.). The reaction mixture was irradiated using a 90 W sunlamp at 0 ˚C using an ice-water Dewar bath for 1 h. To the resulting mixture was added TBAF (1.0 M solution in THF, 9.15 mL, 9.15 mmol, 5.0 equiv.) at room temperature. After stirring at room temperature for 3 h, the reaction mixture was quenched with sat. aq. $Na_2S_2O_3$ (50 mL), diluted with sat. aq. $NaHCO_3$ (50 mL), and extracted with EtOAc (100 mL). The organic phase was dried over Na_2SO_4, filtered and concentrated *in vacuo*. The crude residue was purified by silica gel flash column chromatography

(hexanes/EtOAc = 1:1), yielding **S10** (507 mg, 1.70 µmol, 92% over 2 steps) as a white solid. The analytical data were consistent with the reported values.[1]

Rf-value: 0.45 (hexanes:EtOAc = 1:1; *p*-anisaldehyde)

HRMS (m/z): ESI [M] calculated for $C_{18}H_{18}O_4$+Na $[M+Na]^+$: 321.1097, found $[M+Na]^+$: 321.1098.

^{1}H NMR (400 MHz, $CDCl_3$): δ 7.13 (d, *J* = 7.4 Hz, 1H), 7.06 (dd, *J* = 7.3, 1.1 Hz, 1H), 5.55 (d, *J* = 5.0 Hz, 1H), 5.17 (td, *J* = 5.8, 0.9 Hz, 1H), 3.74 (d, *J* = 5.6 Hz, 1H), 3.31 (t, *J* = 5.4 Hz, 1H), 3.23 (dt, *J* = 15.4, 9.2 Hz, 1H), 2.74 – 2.62 (m, 2H), 2.57 (s, 1H), 2.37 (s, 3H), 1.41 (dt, *J* = 15.2, 8.7 Hz, 1H), 0.73 (d, *J* = 7.6 Hz, 3H), 0.44 (q, *J* = 7.5 Hz, 1H).

^{13}C NMR (126 MHz, $CDCl_3$): δ 172.3, 142.4, 141.5, 134.2, 133.0, 132.4, 128.7, 84.9, 80.8, 79.7, 78.6, 53.0, 52.7, 41.4, 25.5, 20.3, 17.8, 15.2.

Deoxygenated hexacycle S11

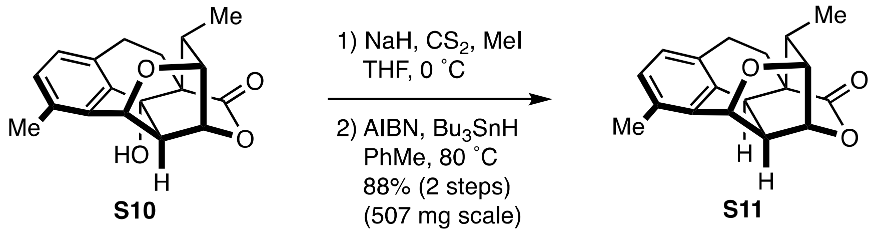

To a solution of **S10** (507 mg, 1.70 mmol) in THF (17.0 mL) was added NaH (60 wt%, 136 mg, 3.40 mmol, 2.0 equiv.) at 0 ˚C. After stirring at 0 °C for 10 min, to the resulting mixture was added CS_2 (1.02 mL, 17.0 mmol, 10.0 equiv.) and MeI (1.06 mL, 17.0 mmol, 10.0 equiv.) at 0 °C. After stirring at 0 °C for 2 h, the reaction mixture was quenched with sat. aq. NH_4Cl (1 0mL) and extracted with EtOAc (50 mL). The organic phase was washed with H_2O (50 mL) and brine (50 mL), dried over Na_2SO_4, filtered, and concentrated *in vacuo*. This crude material was used in the next reaction without further purification.

To a solution of the crude material in PhMe (17.0 mL) was added AIBN (55.8 mg, 0.340 mmol, 20 mol%) and Bu_3SnH (0.916 mL, 3.40 mmol, 2.0 equiv.) at room temperature. After stirring at 80 ˚C for 1 h the reaction mixture was cooled down to room temperature, filtrated through a short plug of silica containing 10% wt/wt K_2CO_3. After the filtrate was concentrated *in vacuo*, the crude residue was purified by silica gel flash column chromatography (hexanes/EtOAc = 4:1), yielding **S11** (423 mg, 1.50 mmol, 88% over 2 steps) as a white solid. The analytical data were consistent with the reported values.[1]

Rf-value: 0.42 (hexanes:EtOAc = 3:1; *p*-anisaldehyde)

HRMS (m/z): ESI [M] calculated for $C_{18}H_{18}O_3$+Na $[M+Na]^+$: 305.1148, found $[M+Na]^+$: 305.1150.

^{1}H NMR (400 MHz, $CDCl_3$): δ 7.09 – 7.04 (m, 2H), 5.58 (d, *J* = 5.0 Hz, 1H), 5.17 (t, *J* = 5.8 Hz, 1H), 3.79 (d, *J* = 5.7 Hz, 1H), 3.57 (dt, *J* = 8.7, 5.5 Hz, 1H), 3.23 (d, *J* = 8.7 Hz, 1H), 2.86 – 2.71 (m, 2H), 2.64 (ddd, *J* = 14.5, 9.1, 1.5 Hz, 1H), 2.40 (s, 3H), 1.33 (ddd, *J* = 14.5, 9.5, 7.9 Hz, 1H), 0.73 (d, *J* = 6.4 Hz, 3H), 0.71 – 0.64 (m, 1H).

^{13}C NMR (126 MHz, $CDCl_3$): δ 174.7, 143.2, 142.2, 133.5, 132.9, 130.5, 127.4, 84.0, 80.3, 79.2, 47.4, 46.8, 43.5, 40.6, 26.7, 23.3, 18.2, 15.1.

Cephanolide A (1)

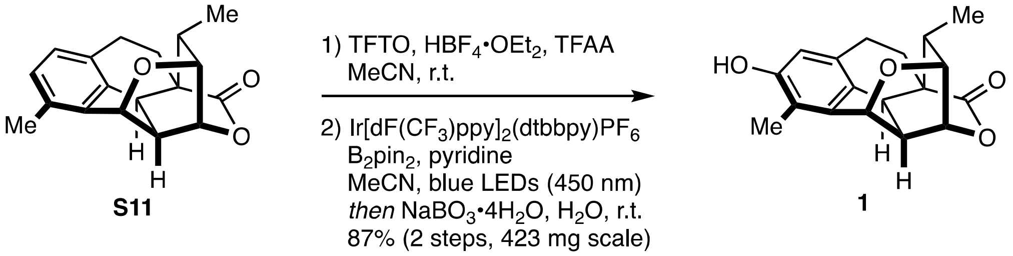

To a solution of **S11** (423 mg, 1.50 mmol) and 2,3,7,8-tetrafluorothianthrene-*S*-oxide (TFTO; 639 mg, 2.10 mmol, 1.4 equiv.) in MeCN (30.0 mL) was added HBF_4•OEt_2 (0.347 mL, 2.55 mmol, 1.7 equiv.), followed by TFAA (0.689 mL, 4.95 mmol, 3.3 equiv.) at 0 °C. After stirring at room temperature for 14 h, the reaction mixture was diluted with H_2O (50 mL) and extracted with DCM (2 x 50 mL). The combined organic phase was washed with sat. aq. $NaBF_4$ (50 mL), dried over Na_2SO_4, filtered, and concentrated *in vacuo*. This crude material was used in the next reaction without further purification.

To a solution the crude material, B_2pin_2 (952 mg, 3.75 mmol, 2.5 equiv.) and Ir[dF(CF_3)ppy]$_2$(dtbbpy)PF_6 (33.7 mg, 30.0 µmol, 2 mol%) in MeCN (10.0 mL) was added pyridine (0.604 mL, 7.50 mmol, 5.0 equiv.) at room temperature. The reaction mixture was sparged with N_2 for 10 min, and then irradiated in a photoreactor at 450 nm for 48 h. To the resulting mixture was added H_2O (10.0 mL) and $NaBO_3$•$4H_2O$ (1.15 g, 7.50 µmol, 5.0 equiv.) at room temperature. After stirring at room temperature for 3 h, the reaction mixture was quenched with sat. aq. $Na_2S_2O_3$ (10 mL), diluted with H_2O (50 mL), and extracted with EtOAc (3 x 50 mL). The combined organic phase was dried over Na_2SO_4, filtered, and concentrated *in vacuo*. The resulting residue was purified by silica gel flash

column chromatography (hexanes/EtOAc = 3:1), yielding **1** (390 mg, 1.31 mmol, 87% over 2 steps) as a white solid. The analytical data were consistent with the reported values.[1]

Rf-value: 0.36 (hexanes:EtOAc = 2:1; *p*-anisaldehyde)

HRMS (m/z): ESI [M] calculated for $C_{18}H_{18}O_4$+Na [M+Na]$^+$: 321.1097, found [M+Na]$^+$: 321.1098.

^{1}H NMR (400 MHz, $CDCl_3$) δ 6.65 (s, 1H), 5.55 (d, *J* = 4.7 Hz, 1H), 5.15 (t, *J* = 5.8 Hz, 1H), 4.90 (s, 1H), 3.79 (d, *J* = 5.6 Hz, 1H), 3.58 (dt, *J* = 10.6, 5.6 Hz, 1H), 3.18 (d, *J* = 8.5 Hz, 1H), 2.83 – 2.75 (m, 1H), 2.69 – 2.58 (m, 2H), 2.28 (s, 3H), 1.33 (dt, *J* = 14.8, 8.9 Hz, 1H), 0.76 – 0.69 (m, 4H).

^{13}C NMR (151 MHz, $CDCl_3$) δ 174.7, 155.1, 144.0, 135.4, 134.5, 118.2, 114.9, 83.9, 80.3, 79.2, 47.7, 46.3, 43.8, 40.5, 26.8, 23.1, 15.1, 11.9.

5. Additional Data

Supplementary Figure 2: Tropone synthesis using simple substrates

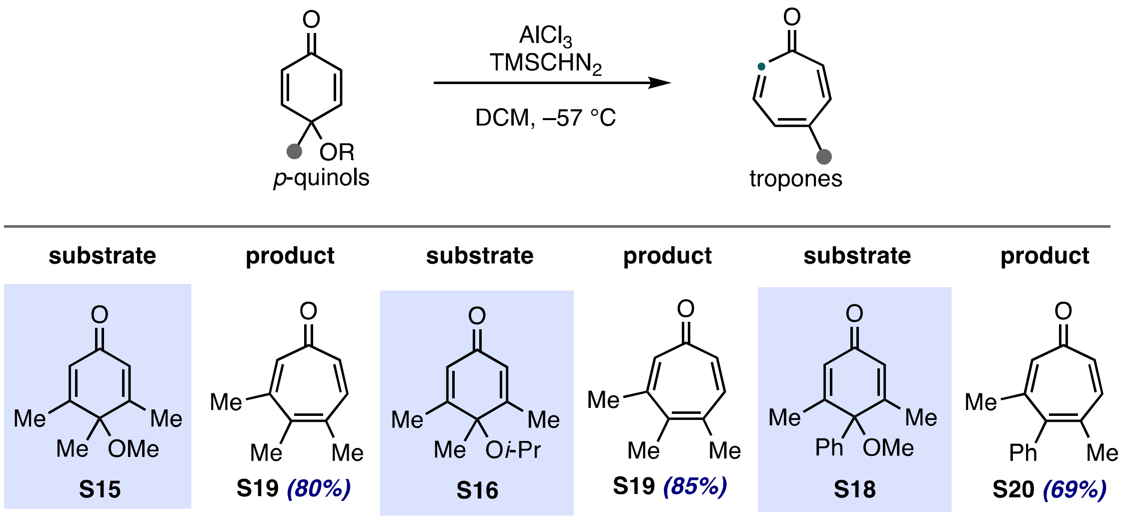

4-Methoxy-3,4,5-trimethylcyclohexa-2,5-dien-1-one (S15)

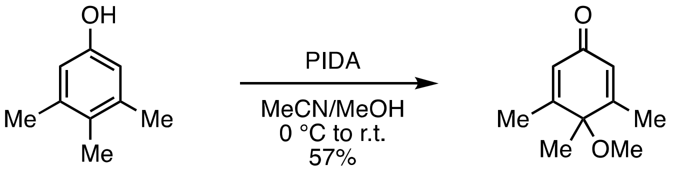

To a solution of **S14** (136 mg, 1.00 mmol) in MeCN/MeOH (1:1 v/v, 10 mL) was added phenyliodine(III) diacetate (PIDA; 387 mg, 1.20 mmol, 1.2 equiv.) at 0 °C. After stirring at room temperature for 6 h, the reaction mixture was quenched with sat. aq. $NaHCO_3$ (10 mL), diluted with H_2O (15 mL) and extracted with DCM (3 x 20 mL). The combined organic phase was dried over Na_2SO_4, filtered, and concentrated in vacuo. The resulting residue was purified by silica gel flash column chromatography (hexanes/EtOAc = 9:1 to 3:1), yielding **S15** (95.1 mg, 572 µmol, 57%) as a colorless solid. ^{1}H NMR data were identical to those reported in the literature.[5]

Rf-value: 0.61 (hexanes:EtOAc = 3:1; *p*-anisaldehyde)

^{1}H NMR (400 MHz, $CDCl_3$): δ 6.14 (s, 1H), 2.92 (s, 1H), 1.97 (s, 2H), 1.36 (s, 1H).

4-Isopropoxy-3,4,5-trimethylcyclohexa-2,5-dien-1-one (S16)

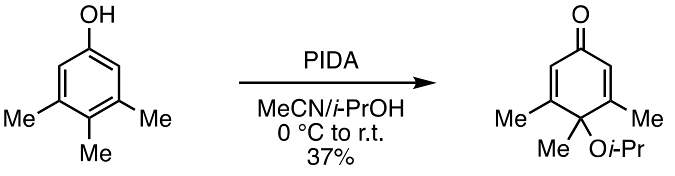

To a solution of **S14** (204 mg, 1.50 mmol) in MeCN/*i*-PrOH (1:1 v/v, 15 mL) was added phenyliodine(III) diacetate (PIDA; 579 mg, 1.80 mmol, 1.2 equiv.) at 0 °C. After stirring at room temperature for 14 h, the reaction mixture was quenched with sat. aq. $NaHCO_3$ (7 mL), diluted with H_2O (10 mL) and extracted with DCM (3 x 15 mL). The combined organic phase was dried over Na_2SO_4, filtered, and concentrated *in vacuo*. The resulting residue was purified by silica gel flash column chromatography (hexanes/EtOAc = 9:1 to 2:1), yielding **S16** (108 mg, 555 µmol, 37%) as a yellow oil.

Rf-value: 0.67 (hexanes:EtOAc = 2:1; *p*-anisaldehyde)

HRMS (m/z): EI $[M]^+$ calculated for $C_{12}H_{18}O_2$: 194.1307, found: 194.1304.

^{1}H NMR (600 MHz, $CDCl_3$) δ 6.14 (s, 2H), 3.42 (hept, *J* = 6.1 Hz, 1H), 2.05 (s, 6H), 1.37 (s, 3H), 1.09 (s, 3H), 1.08 (s, 3H).

^{13}C NMR (151 MHz, $CDCl_3$) δ 185.66, 161.68, 128.63, 76.00, 68.69, 25.22, 24.05, 18.77.

1-Methoxy-2,6-dimethyl-[1,1'-biphenyl]-4(1H)-one (S18)

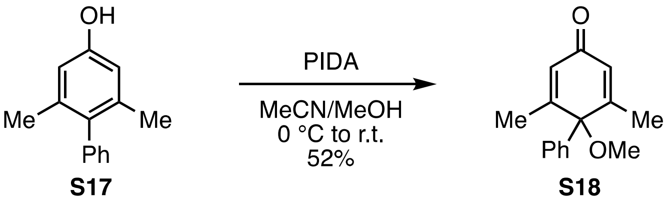

To a solution of **S17** (99.0 mg, 500 µmol) in MeCN/MeOH (1:1 v/v, 12.5 mL) was added phenyliodine(III) diacetate (PIDA; 193 mg, 600 µmol, 1.2 equiv.) at 0 °C. After stirring at room temperature for 1 h, the reaction mixture was quenched with sat. aq. $NaHCO_3$ (7 mL), diluted with H_2O (10 mL) and extracted with DCM (3 x 20 mL). The combined organic phase was dried over Na_2SO_4, filtered, and concentrated *in vacuo*. The resulting residue was purified by silica gel flash column chromatography (hexanes/EtOAc = 9:1 to 3:1), yielding **S18** (59.2 mg, 259 µmol, 52%) as a colorless gum.

Rf-value: 0.33 (hexanes:EtOAc = 3:1; *p*-anisaldehyde)

HRMS (m/z): EI $[M]^+$ calculated for $C_{15}H_{16}O_2$: 228.1150, found: 228.1152.

^{1}H NMR (600 MHz, $CDCl_3$) δ 7.40 – 7.30 (m, 4H), 7.30 – 7.27 (m, 1H), 6.27 (s, 2H), 3.19 (s, 3H), 1.67 (s, 6H).

^{13}C NMR (151 MHz, $CDCl_3$) δ 186.33, 160.17, 138.93, 129.38, 128.64, 127.90, 125.52, 81.24, 51.69, 18.20.

3,4,5-Trimethylcyclohepta-2,4,6-trien-1-one (S19)

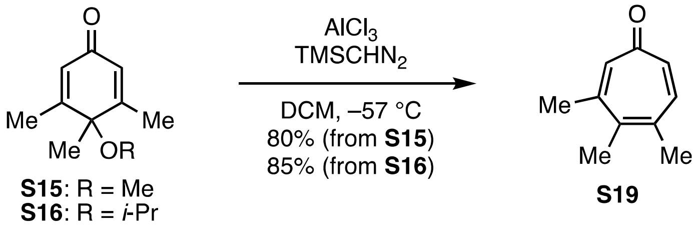

A flame-dried vial with a magnetic stir bar was transferred to a glovebox and charged with $AlCl_3$ (160 mg, 1.20 mmol, 3.0 equiv.). The vial was sealed with a septa cap and removed from the glovebox. The vial was evacuated and backfilled with N_2 three times and cooled to –57 °C (*n*-octane/dry ice bath). Freshly distilled DCM (660 µL) was added, and the suspension was stirred at –57 °C for 5 min. A solution of **S15** (66.5 mg, 400 µmol) in freshly distilled DCM (3.34 mL) was added and stirred at –57 °C for 10 min to give a suspension. $TMSCHN_2$ (0.2 M, prepared from a 2.0 M solution in hexanes diluted with freshly distilled DCM, 10.0 mL, 2.00 mmol, 5.0 equiv.) was added over 2 min. The mixture war stirred at –57 °C for 3 h and quenched with sat. aq. $NaHCO_3$ (5 mL). The suspension was diluted with H_2O (10 mL) and extracted with DCM (3 x 15 mL). The combined organic phase was dried over Na_2SO_4, filtered, and concentrated *in vacuo*. The residue was purified by silica gel flash column chromatography (hexanes/EtOAc = 1:3), yielding **S19** (47.3 mg, 319 µmol, 80%) as a colorless solid.
Analogous to the procedure described above with **S16** as starting material (60.0 mg, 309 µmol, 1.0 equiv.), the product (**S19**) was obtained in 85% yield (39.0 mg, 263 µmol) as a colorless solid.

Rf-value: 0.24 (hexanes:EtOAc = 1:3; *p*-anisaldehyde)

Melting Point: 73–75 °C

HRMS (m/z): EI $[M]^+$ calculated for $C_{10}H_{12}O$: 148.0888, found: 148.0889.

^{1}H NMR (600 MHz, $CDCl_3$) δ 7.04 (d, *J* = 12.3 Hz, 1H), 7.00 (d, *J* = 3.0 Hz, 1H), 6.79 (dd, *J* = 12.4, 3.0 Hz, 1H), 2.30 (s, 6H), 2.17 (s, 3H).

^{13}C NMR (151 MHz, $CDCl_3$) δ 186.60, 148.71, 143.52, 141.47, 140.65, 139.91, 137.74, 27.94, 26.19, 21.04.

3,5-Dimethyl-4-phenylcyclohepta-2,4,6-trien-1-one (S20)

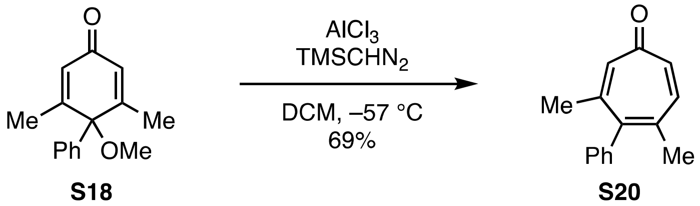

A flame-dried vial with a magnetic stir bar was transferred to a glovebox and charged with $AlCl_3$ (52.6 mg, 394 µmol, 3.0 equiv.). The vial was sealed with a septa cap and removed from the glovebox. The vial was evacuated and backfilled with N_2 three times and cooled to –57 °C (*n*-octane/dry ice bath). Freshly distilled DCM (220 µL) was added, and the suspension was stirred at –57 °C for 5 min. A solution of **S18** (30.0 mg, 131 µmol) in freshly distilled DCM (1.09 mL) was added and stirred at –57 °C for 10 min to give a suspension. $TMSCHN_2$ (0.2 M, prepared from a 2.0 M solution in hexanes diluted with freshly distilled DCM, 3.29 mL, 657 µmol, 5.0 equiv.) was added over 2 min. The mixture war stirred at –57 °C for 3 h and quenched with sat. aq. $NaHCO_3$ (2 mL). The suspension was diluted with H_2O (5 mL) and extracted with DCM (3 x 7 mL). The combined organic layers were dried over anhydrous Na_2SO_4, filtered, and concentrated *in vacuo*. The residue was purified by preparative TLC (hexanes/EtOAc = 1:3), yielding tropone **S20** (19.1 mg, 90.8 µmol, 69%) as a colorless solid.

Rf-value: 0.54 (hexanes:EtOAc = 1:3; *p*-anisaldehyde)

HRMS (m/z): EI $[M]^+$ calculated for $C_{15}H_{14}O$: 210.1045, found: 210.1047.

Melting Point: 102-105 °C

^{1}H NMR (600 MHz, $CDCl_3$) δ 7.48 – 7.44 (m, 2H), 7.40 – 7.34 (m, 1H), 7.19 (d, *J* = 12.3 Hz, 1H), 7.16 (d, *J* = 3.0 Hz, 1H), 7.08 – 7.02 (m, 3H), 1.96 (s, 3H), 1.91 (s, 3H).

^{13}C NMR (151 MHz, $CDCl_3$) δ 186.71, 148.21, 148.12, 142.61, 141.62, 141.01, 140.03, 139.45, 129.29, 127.64, 127.55, 29.23, 26.57.

6. Spectral Data Comparison of Natural and Synthetic Harringtonolide

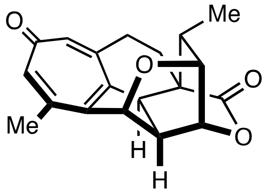

harringtonolide

• ^{1}H NMR

Buta's natural[6] (100 MHz, $CDCl_3$) δ_H	Sun's natural[7] (100 MHz, $CDCl_3$) δ_H	Tang's synthetic[8] (500 MHz, $CDCl_3$) δ_H	Zhai's synthetic[9] (400 MHz, $CDCl_3$) δ_H **$CDCl_3$ = 7.27 as a ref.*	**This work (600 MHz, $CDCl_3$) δ_H *$CDCl_3$ = 7.26 as a ref.***
6.98 (s, 1H)	6.95 (d, *J* = 2 Hz, 1H)	6.98 (s, 1H)	6.97 (s, 1H)	**6.96 (s, 1H)**
6.92 (s, 1H)	6.77 (d, *J* = 2.0 Hz, 1H)	6.91 (s, 1H)	6.90 (s, 1H)	**6.89 (t, *J* = 2.1 Hz, 1H)**
5.47 (m, 1H)	5.35 (q, 1H)	5.36 (m, 1H)	5.36 (t, *J* = 2.8 Hz, 1H)	**5.35 (d, *J* = 5.2 Hz, 1H)**
5.32 (m, 1H)	5.19 (m, 1H)	5.22 (dd, *J* = 5.3, 5.3 Hz, 1H)	5.21 (t, *J* = 5.2 Hz, 1H)	**5.20 (t, *J* = 5.1 Hz, 1H)**
4.00 (m, 1H)	3.98 (d, *J* = 6 Hz, 1H)	4.00 (d, *J* = 5.6 Hz, 1H)	3.99 (d, *J* = 5.6 Hz, 1H)	**3.98 (d, *J* = 5.7 Hz, 1H)**
3.51 (m, 2H)	3.40 (m, 2H)	3.42 (m, 2H)	3.36 – 3.42 (m, 2H)	**3.36 – 3.40 (m, 2H)**
2.70 (m, 3H)	2.85 (m, 1H)	2.87 (m, 1H)	2.82 – 2.91 (m, 2H)	**2.81 – 2.88 (m, 2H)**
	2.75 (m, 1H)	2.83 (m, 1H)		
	2.65 (m, 1H)	2.64 (dd, *J* = 13.7, 6.5 Hz, 1H)	2.60 – 2.66 (m, 1H)	**2.62 (dd, *J* = 14.0, 6.2 Hz, 1H)**
2.36 (s, 3H)	2.37 (s, 3H)	2.38 (s, 3H)	2.37 (d, *J* = 0.8 Hz, 3H)	**2.36 (d, *J* = 1.2 Hz, 3H)**
1.75 (q, 1H)	1.74 (q, *J* = 8 Hz, 1H)	1.77 (q, *J* = 7.5 Hz, 1H)	1.76 (q, *J* = 7.6 Hz, 1H)	**1.76 (q, *J* = 7.6 Hz, 1H)**
1.25 (m, 1H)	1.32 (m, 1H)	1.31 (m, 1H)	1.28 – 1.36 (m, 1H)	**1.27 – 1.33 (m, 1H)**
0.90 (d, 3H)	0.89 (d, *J* = 8 Hz, 3H)	0.91 (d, *J* = 7.6 Hz, 3H)	0.90 (d, *J* = 7.6 Hz, 3H)	**0.89 (d, *J* = 7.6 Hz, 3H)**

• ^{13}C NMR

Buta's natural[6] (25 MHz, $CDCl_3$) δ_C	Sun's natural[7] ($CDCl_3$) δ_C	Tang's synthetic[8] ($CDCl_3$) δ_C	Zhai's synthetic[9] (150 MHz, $CDCl_3$) δ_C **CDCl_3* = 77.0 as a ref.*	**This work (151 MHz, $CDCl_3$) δ_C** ***CDCl_3* = 77.16 as a ref.**
186.4	186.9	186.3	186.5	**186.6**
173.5	173.0	173.3	173.5	**173.6**
145.9	145.9	145.7	145.8	**146.0**
145.7	145.2	-	145.6	**145.8**
145.0	145.0	144.8	145.0	**145.1**
143.6	143.4	-	143.6	**143.7**
141.5	141.5	141.5	141.6	**141.8**
139.1	139.4	139.1	139.3	**139.4**
85.5	86.0	85.9	86.0	**86.2**
80.0	79.6	79.9	80.0	**80.2**
80.0	78.2	79.6	79.7	**79.9**
49.9	49.7	49.8	49.9	**50.1**
43.8	45.5	45.7	45.8	**46.0**
41.7	41.5	41.7	41.8	**42.0**
40.0	39.7	39.9	40.0	**40.2**
32.3	31.8	32.2	32.3	**32.5**
23.8	23.3	23.7	23.9	**24.0**
22.3	22.0	22.3	22.4	**22.6**
14.7	14.2	14.6	14.7	**14.9**

7. NMR Spectra Charts

• Methyl-ceforalide H **20**: ^{1}H NMR ($CDCl_3$)

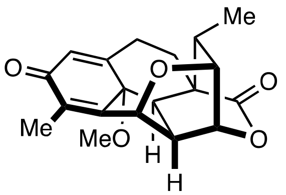

20

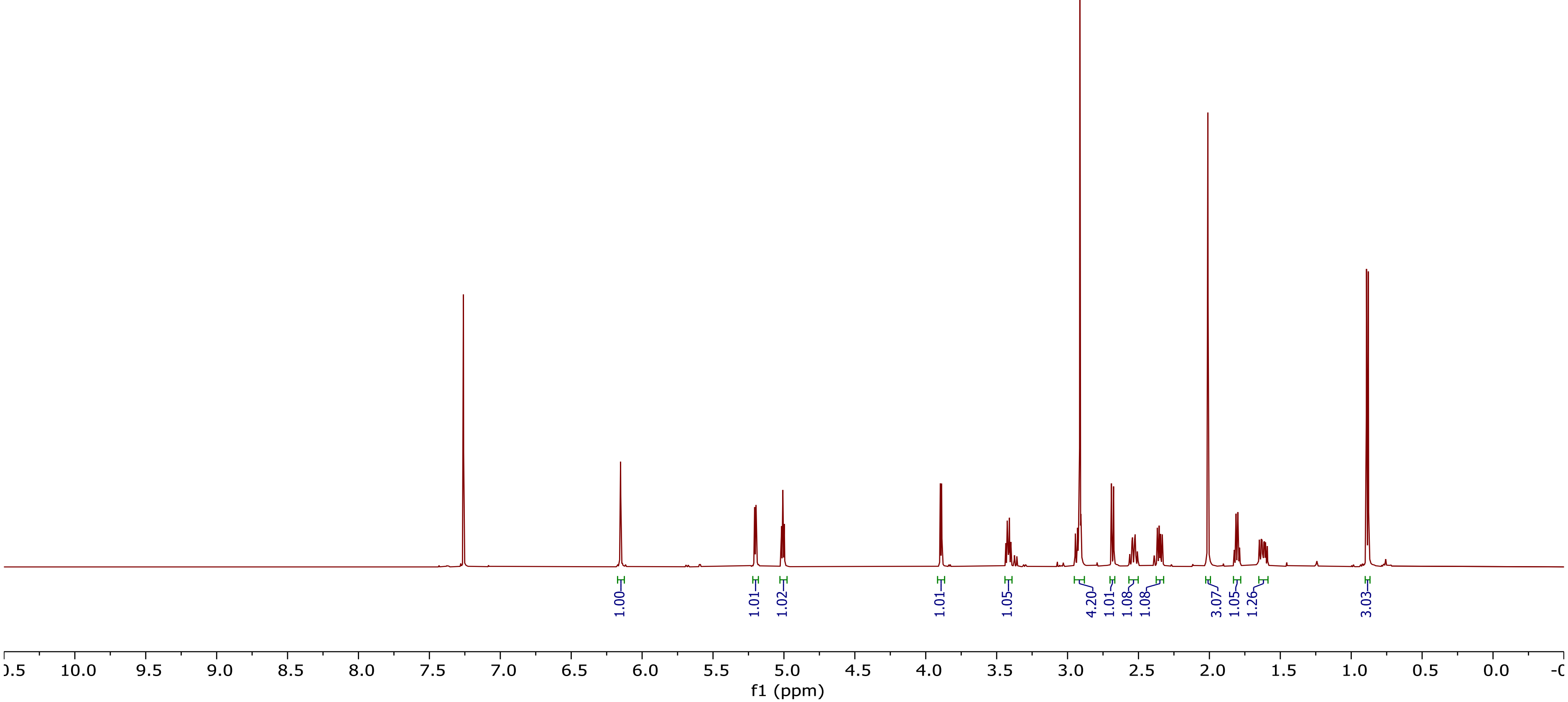

• Methyl-ceforalide H **20**: ^{13}C NMR ($CDCl_3$)

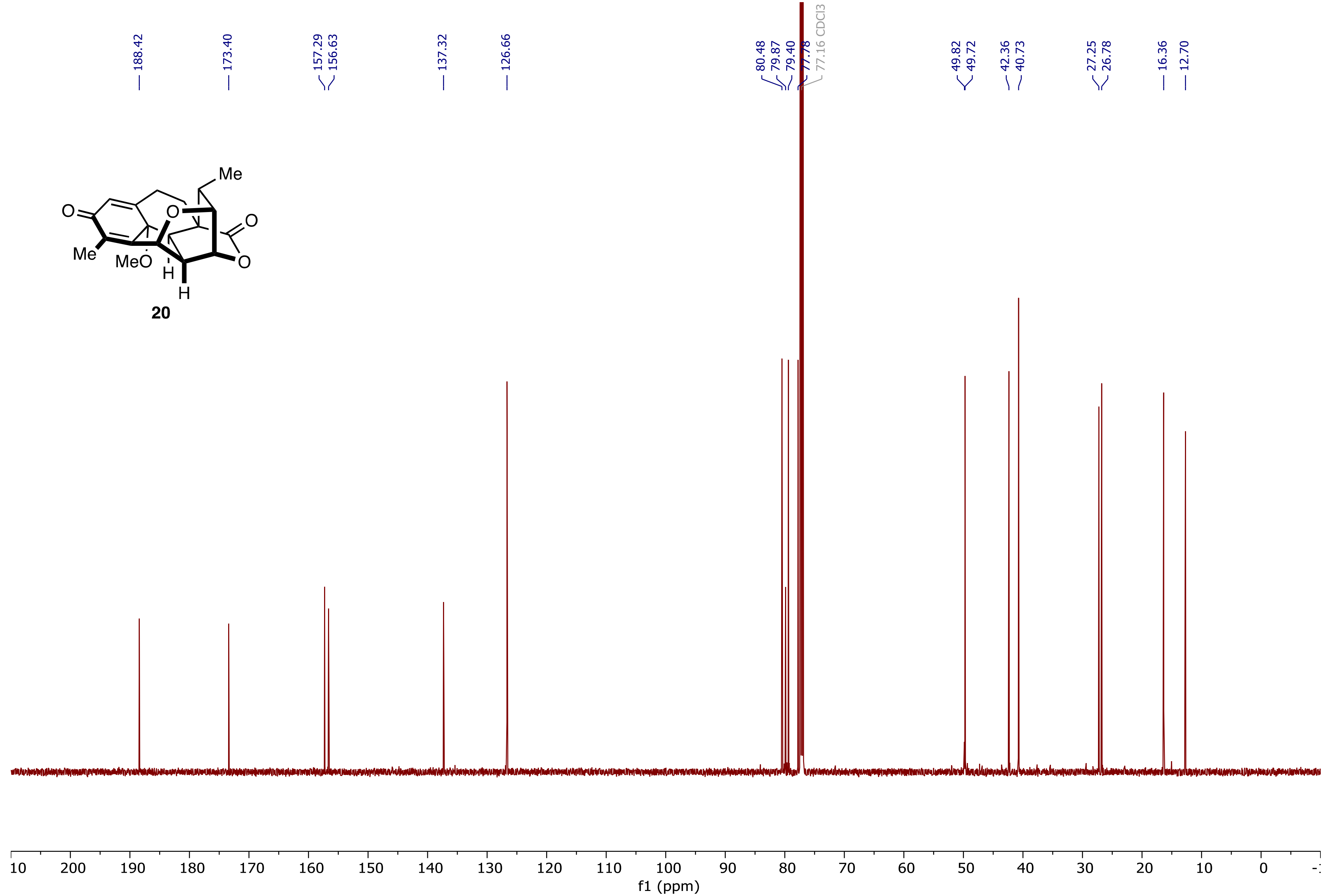

• Harringtonolide (**4**): ^{1}H NMR ($CDCl_3$)

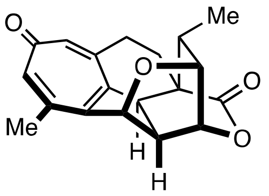

4

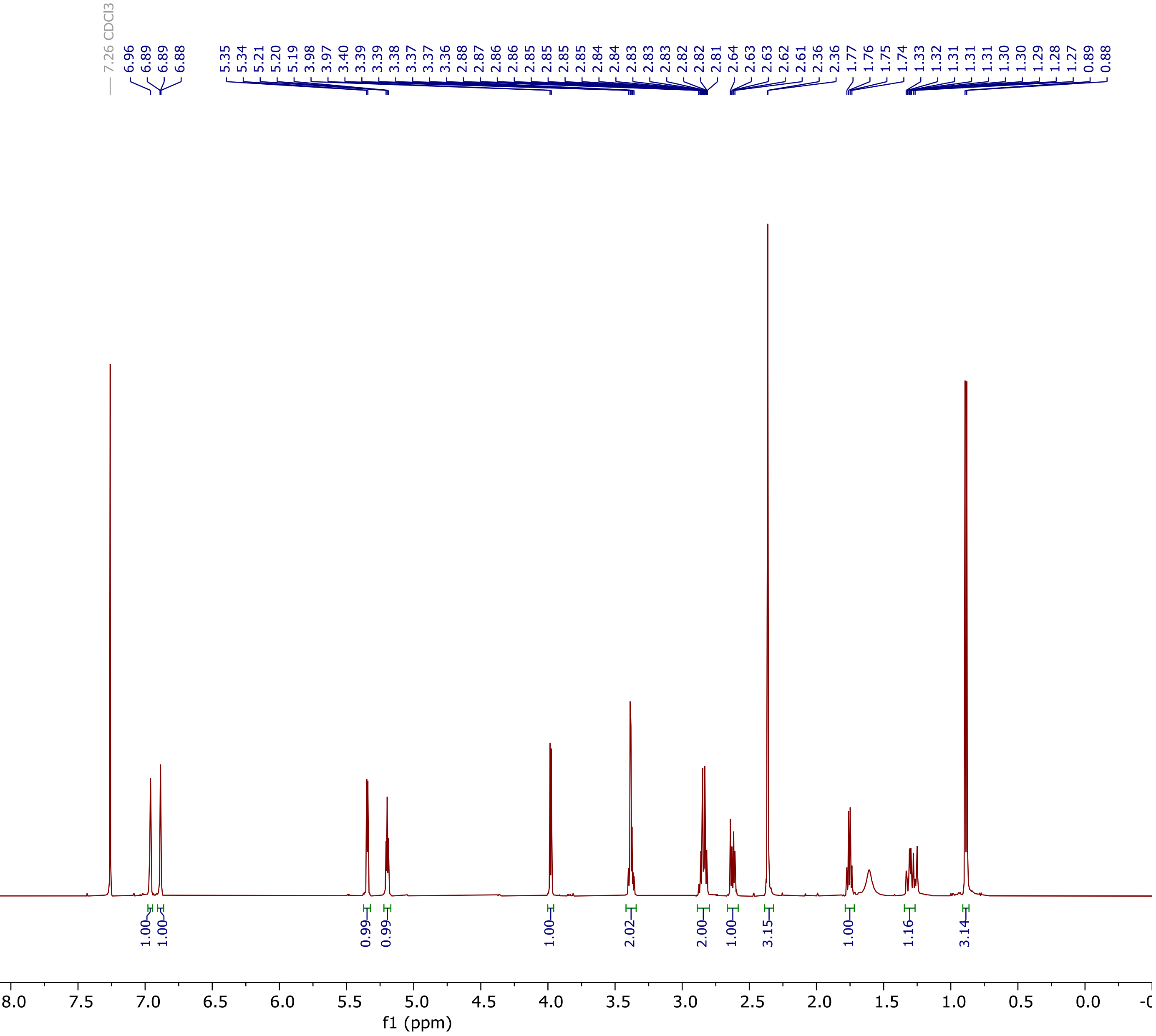

• Harringtonolide (**4**): ^{13}C NMR ($CDCl_3$)

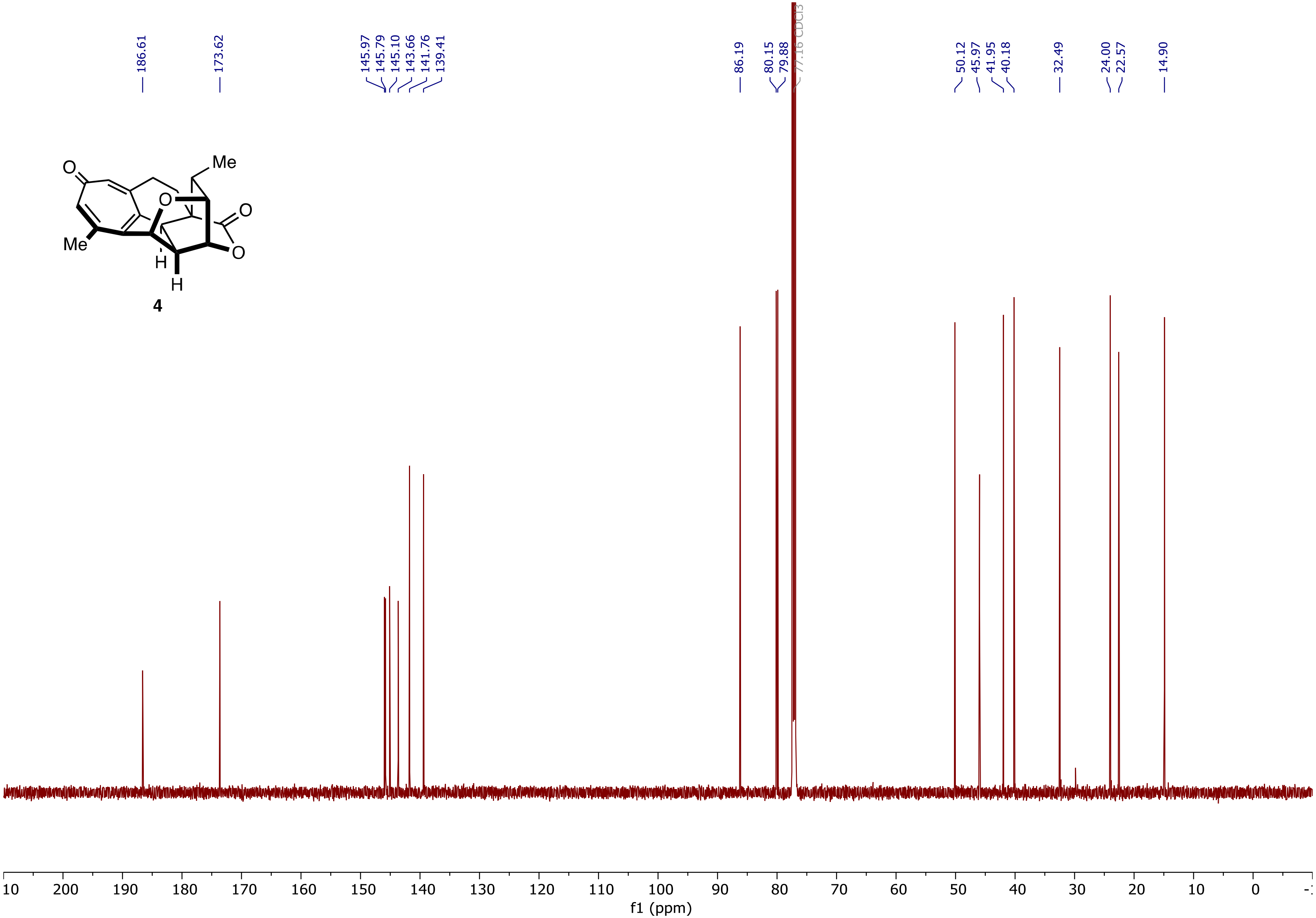

4

• *Iso*-harringtonolide (**25**): ^{1}H NMR ($CDCl_3$)

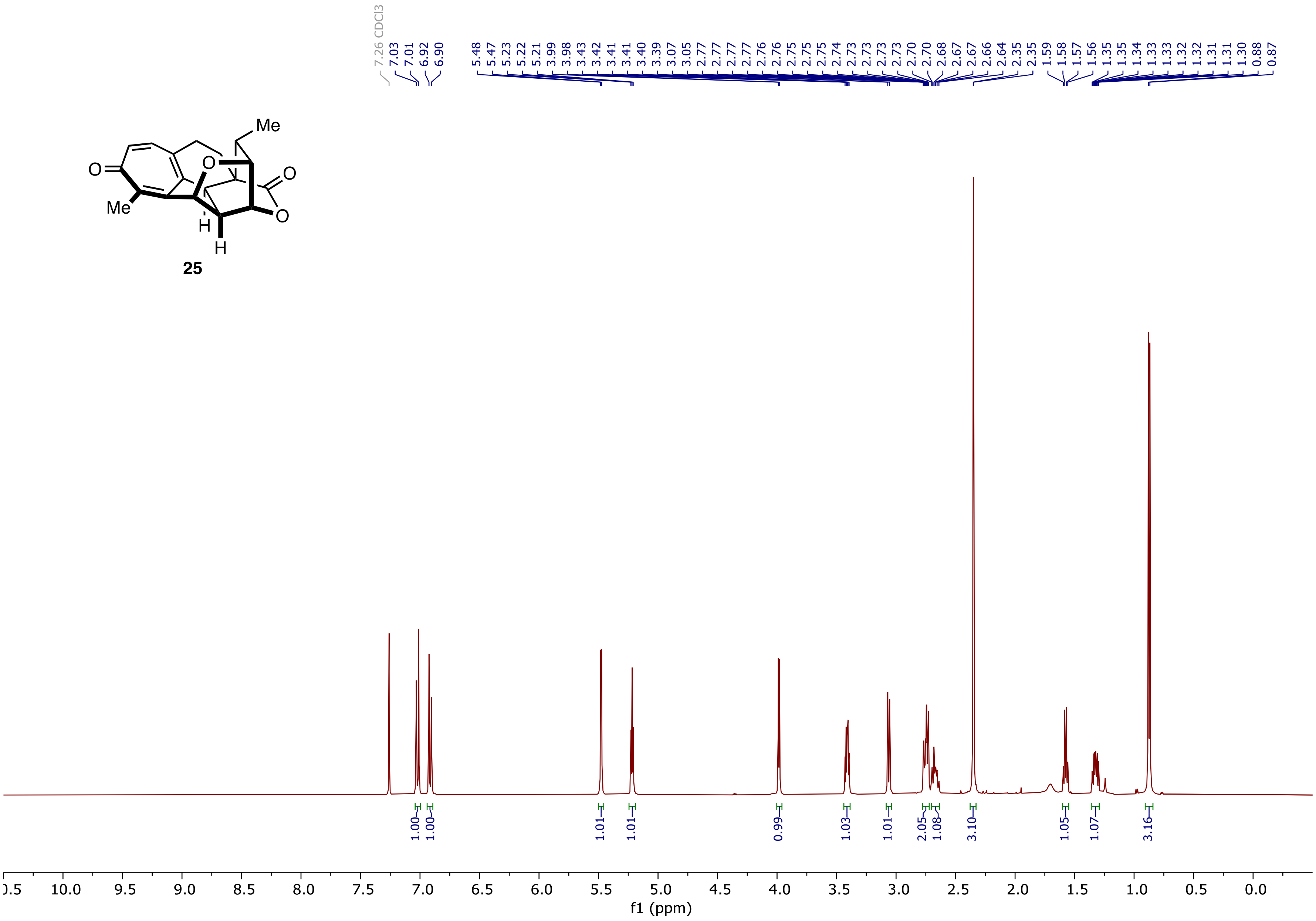

• *Iso*-harringtonolide (**25**): ^{13}C NMR ($CDCl_3$)

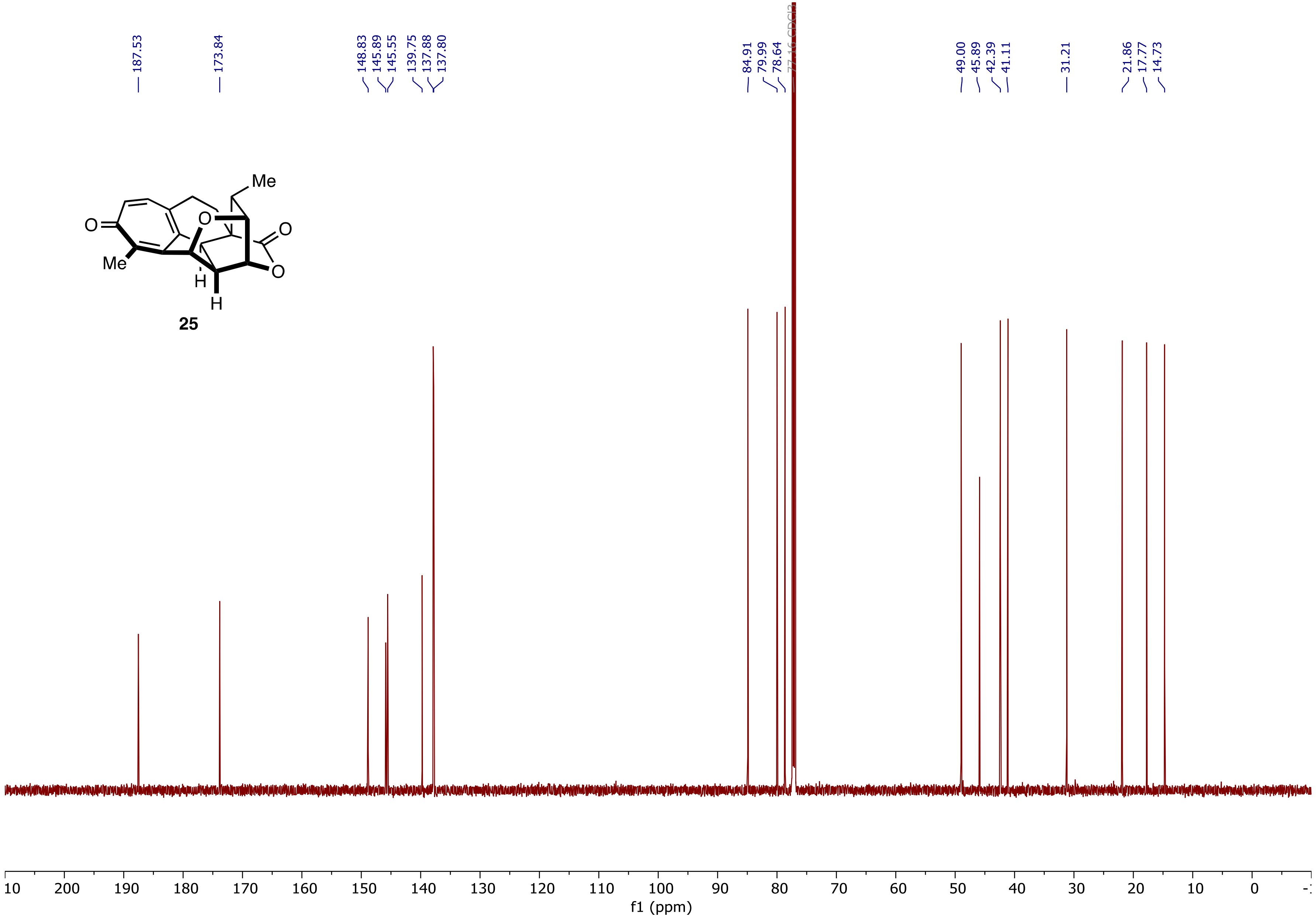

• 4-Isopropoxy-3,4,5-trimethylcyclohexa-2,5-dien-1-one **(S16)**: ^{1}H NMR ($CDCl_3$)

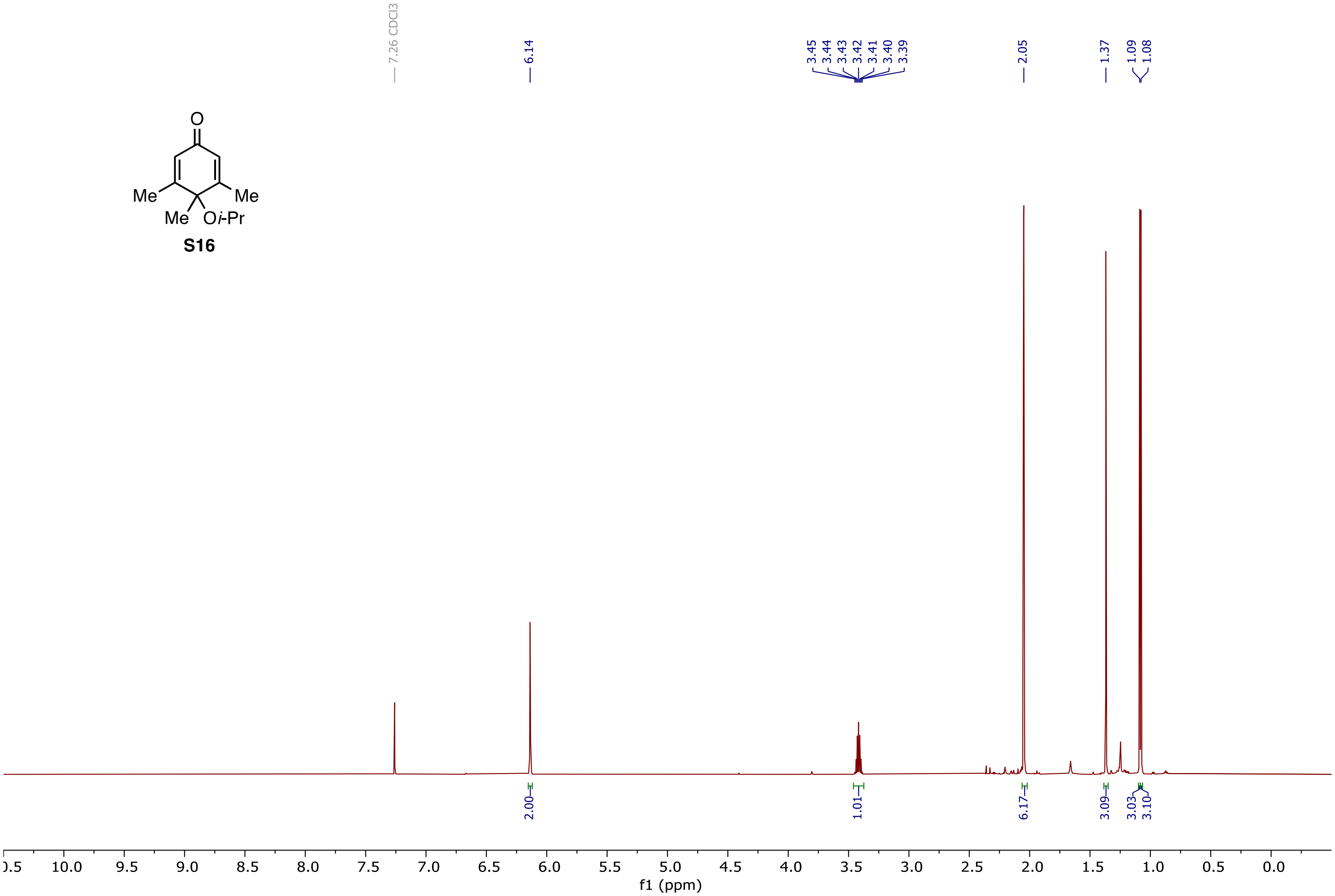

• 4-Isopropoxy-3,4,5-trimethylcyclohexa-2,5-dien-1-one **(S16)**: ^{13}C NMR ($CDCl_3$)

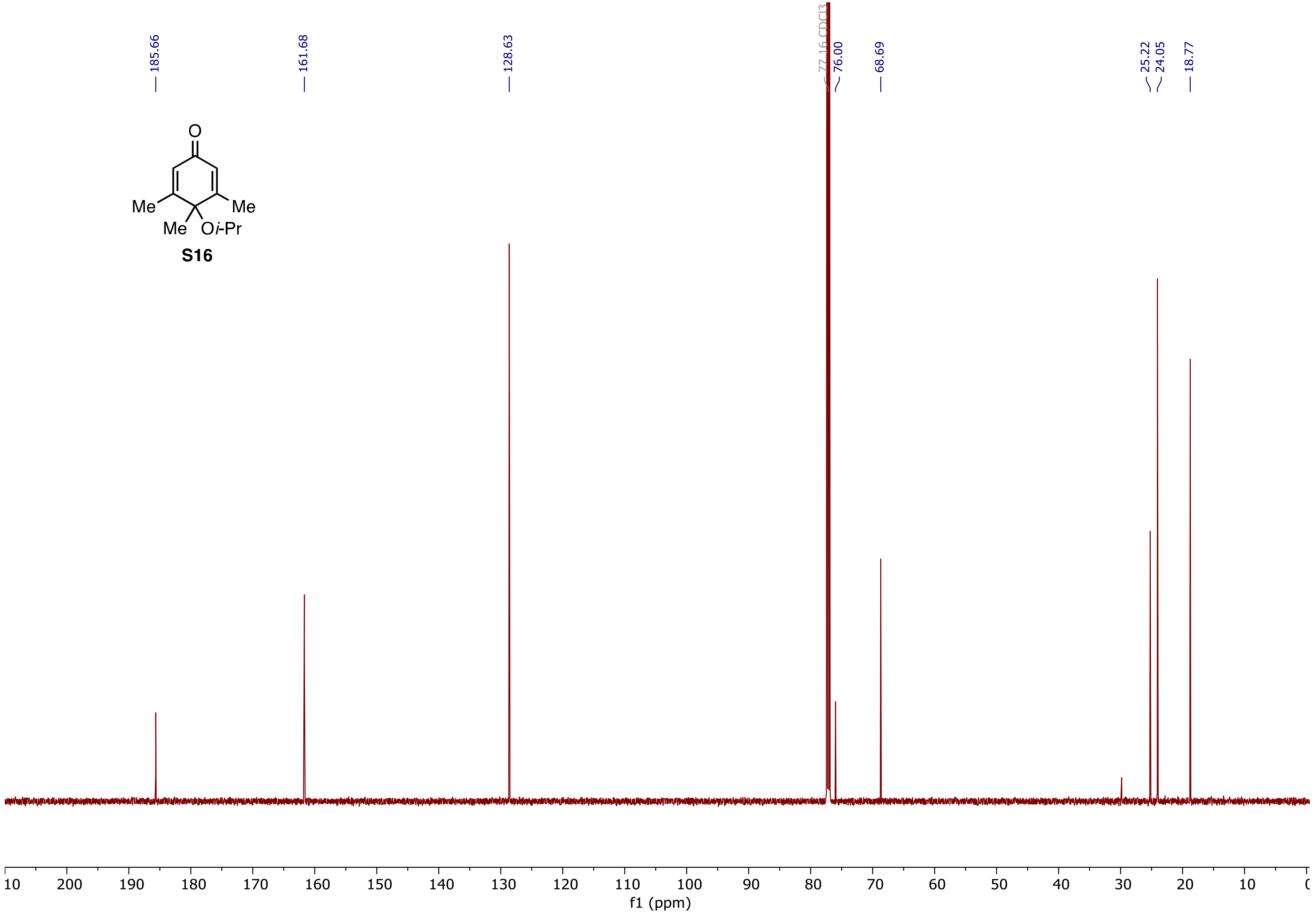

• 1-Methoxy-2,6-dimethyl-[1,1'-biphenyl]-4(1H)-one **(S18)**: ^{1}H NMR ($CDCl_3$)

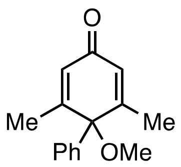

S18

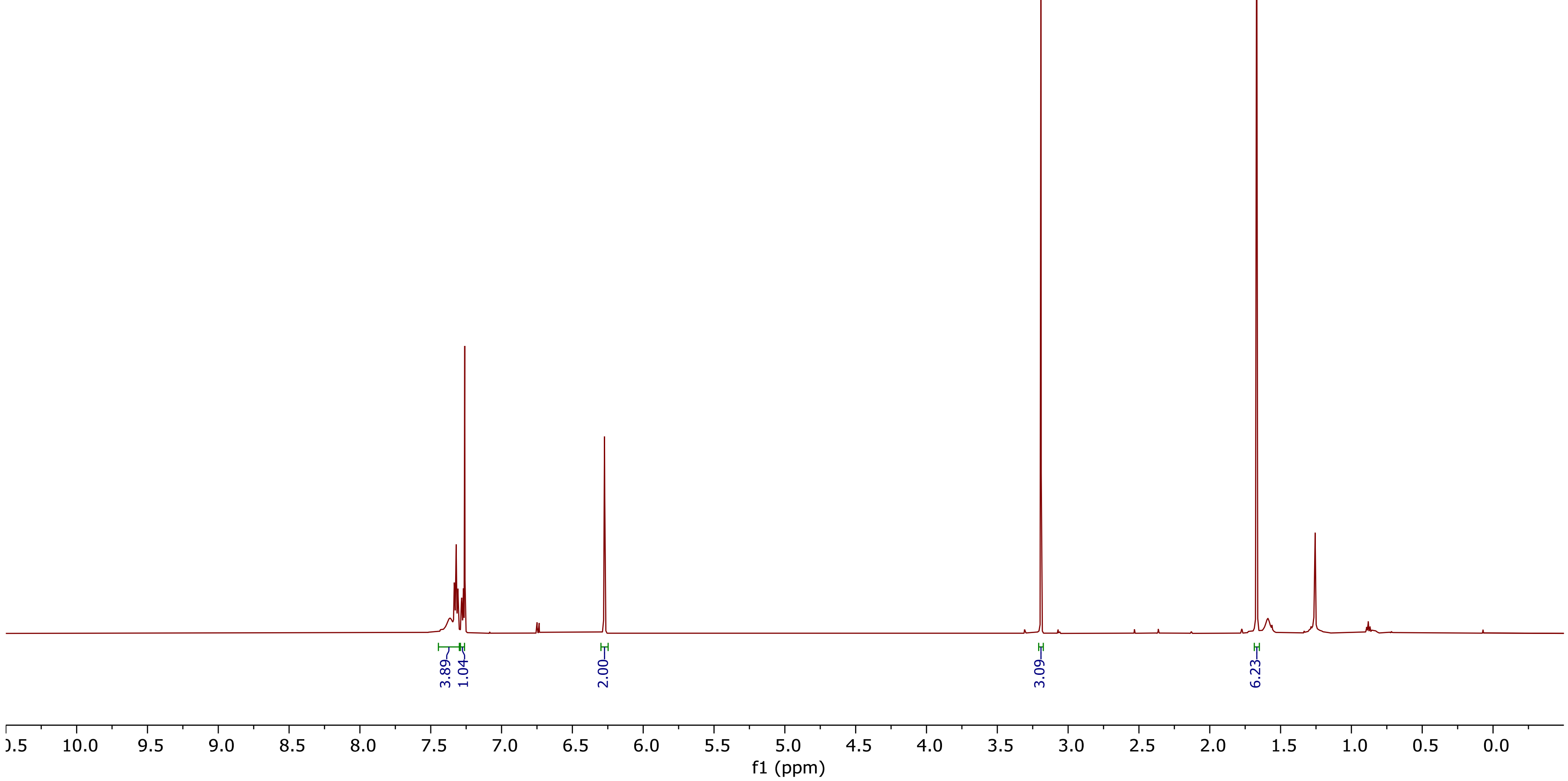

• 1-Methoxy-2,6-dimethyl-[1,1'-biphenyl]-4(1H)-one **(S18)**: ^{13}C NMR ($CDCl_3$)

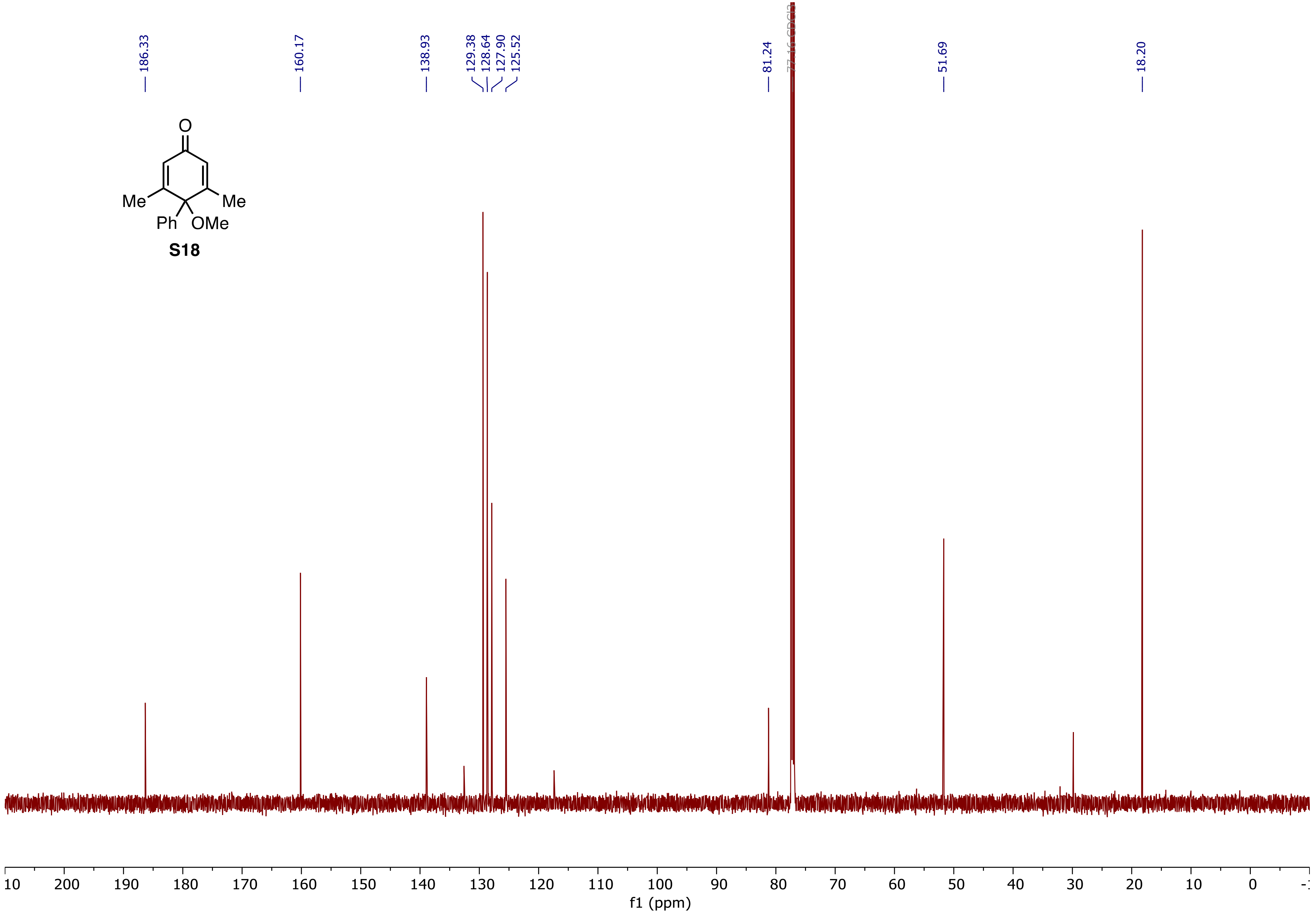

• 3,4,5-Trimethylcyclohepta-2,4,6-trien-1-one **(S19)**: ^{1}H NMR ($CDCl_3$)

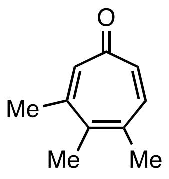

S19

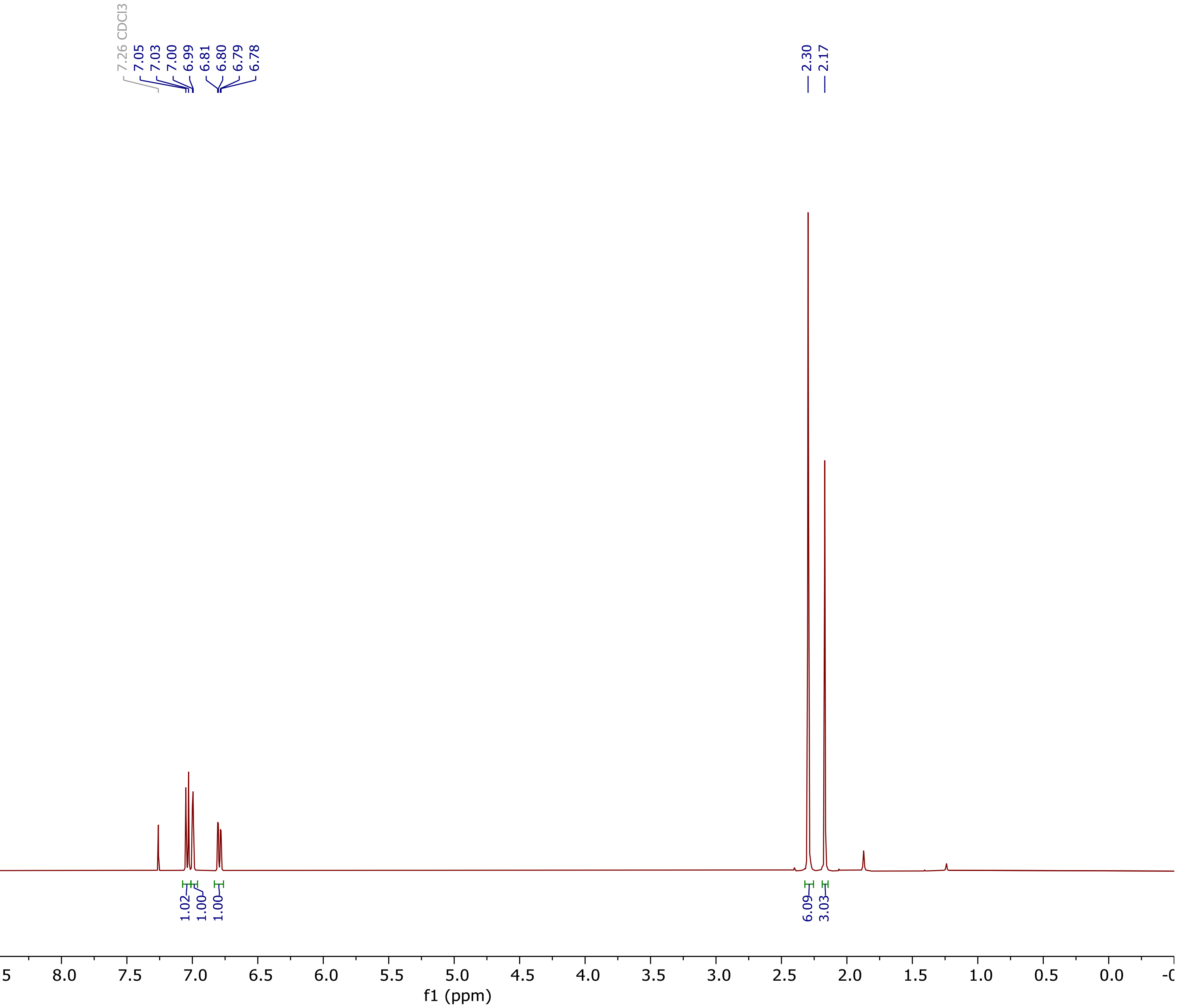

• 3,4,5-Trimethylcyclohepta-2,4,6-trien-1-one **(S19)**: ^{13}C NMR ($CDCl_3$)

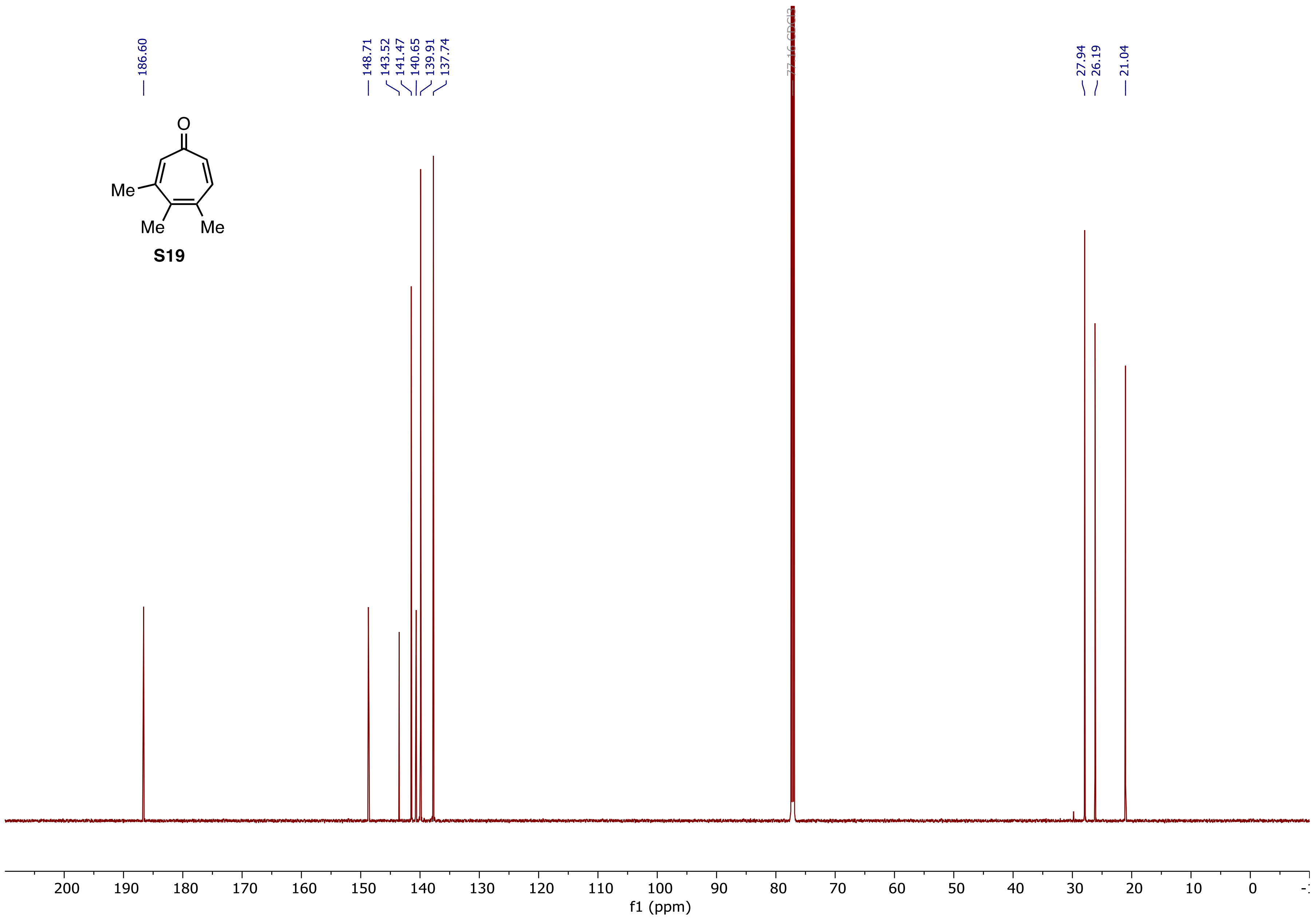

• 3,5-Dimethyl-4-phenylcyclohepta-2,4,6-trien-1-one **(S20)**: ^{1}H NMR ($CDCl_3$)

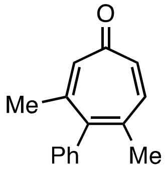

S20

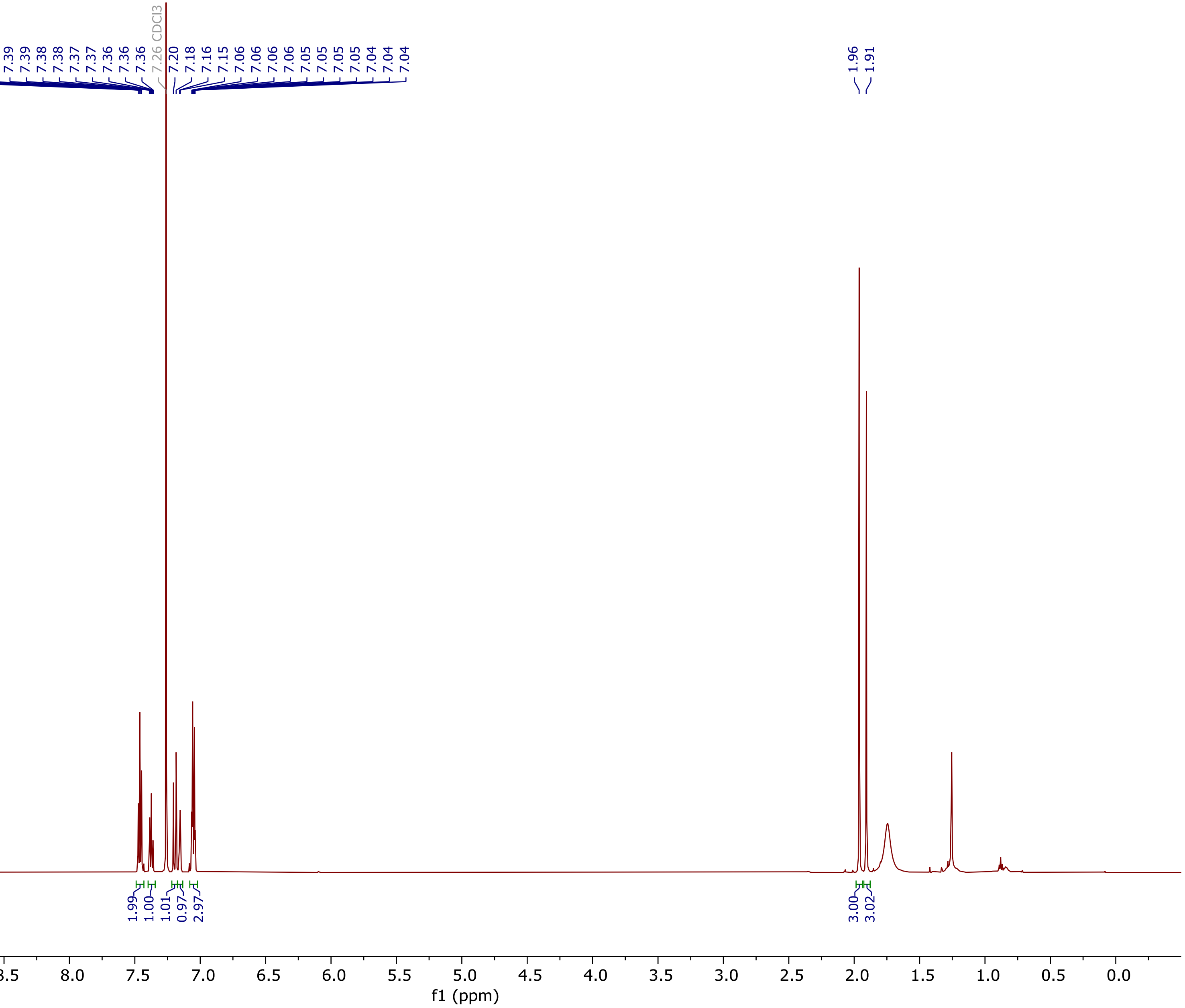

• 3,5-Dimethyl-4-phenylcyclohepta-2,4,6-trien-1-one **(S20)**: ^{13}C NMR ($CDCl_3$)

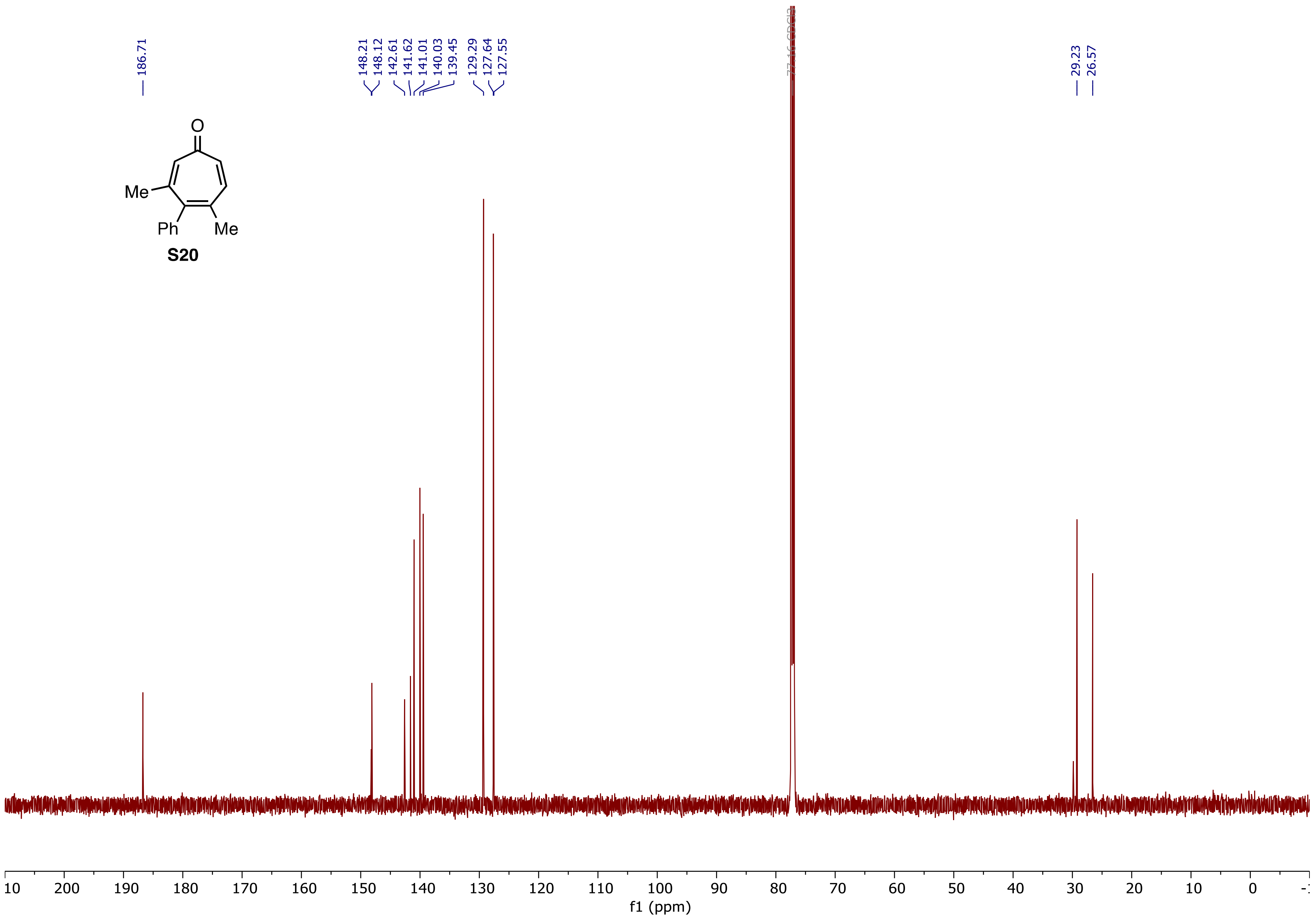

8. Crystallographic Data

8-1. Supplementary Figure 3: X-ray structure of Me-ceforalide H (20) (CCDC 2293695)

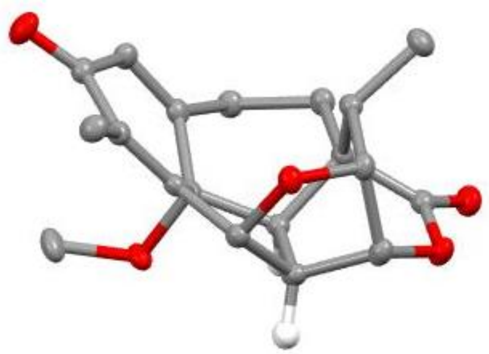

A colorless block 0.33 x 0.18 x 0.16 mm in size was mounted on a Cryoloop with Paratone oil. Data were collected in a nitrogen gas stream at 100(2) K using omega scans. Crystal-to-detector distance was 30.21 mm and exposure time was 0.50 seconds per frame using a scan width of 0.5°. Data collection was 100% complete to 74.000° in θ. A total of 16408 reflections were collected covering the indices -10<=h<=10, -14<=k<=15, -17<=l<=17. 3070 reflections were found to be symmetry independent, with an R_{int} of 0.0285. Indexing and unit cell refinement indicated a primitive, monoclinic lattice. The space group was found to be P 21/c (No. 14). The data were integrated using the CrysAlis^{Pro} 1.172.42.90a software program and scaled using the SCALE3 ABSPACK scaling algorithm. Solution by intrinsic phasing (SHELXT-2015) produced a heavy-atom phasing model consistent with the proposed structure. All non-hydrogen atoms were refined anisotropically by full-matrix least-squares (SHELXL-2014). All hydrogen atoms were placed using a riding model. Their positions were constrained relative to their parent atom using the appropriate HFIX command in SHELXL-2014. A partially occupied water molecule was found to sit in the void space formed by the molecular packing, with this occupancy stabilizing at 11%. Due to the low electron density associated with this water molecule, the hydrogen atoms on it could not be found in the Fourier map. It was deemed more appropriate to leave the hydrogens off the water molecule rather than model them using an HFIX command.

Supplementary Table 4: Crystal data and structure refinement for SWiesler03_Sarpong

Identification code	SWiesler03_Sarpong	
Empirical formula	C19 H20 O5	
Formula weight	328.35	
Temperature	100(2) K	
Wavelength	1.54184 Å	
Crystal system	Monoclinic	
Space group	P 21/c	
Unit cell dimensions	a = 8.64520(10) Å	α= 90°.
	b = 12.1893(2) Å	β= 91.3420(10)°.
	c = 14.2812(2) Å	γ = 90°.
Volume	1504.53(4) Å^3	
Z	4	
Density (calculated)	1.450 Mg/m^3	
Absorption coefficient	0.862 mm^{-1}	
F(000)	696	
Crystal size	0.330 x 0.180 x 0.160 mm^3	
Theta range for data collection	4.770 to 74.496°.	
Index ranges	-10<=h<=10, -14<=k<=15, -17<=l<=17	
Reflections collected	16408	
Independent reflections	3070 [R(int) = 0.0285]	
Completeness to theta = 67.684°	99.9 %	
Absorption correction	Semi-empirical from equivalents	
Max. and min. transmission	1.00000 and 0.85498	
Refinement method	Full-matrix least-squares on F^2	
Data / restraints / parameters	3070 / 0 / 220	
Goodness-of-fit on F^2	1.039	
Final R indices [I>2sigma(I)]	R1 = 0.0356, wR2 = 0.0882	
R indices (all data)	R1 = 0.0374, wR2 = 0.0895	
Extinction coefficient	n/a	
Largest diff. peak and hole	0.282 and -0.195 e.Å^{-3}	

Supplementary Table 5: Atomic coordinates (x 10^4) and equivalent isotropic displacement parameters ($Å^2$x 10^3) for swiesler03_sarpong

U(eq) is defined as one third of the trace of the orthogonalized U^{ij} tensor.

	x	y	z	U(eq)
O(1)	8177(1)	2996(1)	520(1)	24(1)
O(2)	9173(1)	2770(1)	1949(1)	22(1)
O(3)	9853(1)	4835(1)	3734(1)	19(1)
O(4)	6465(1)	8380(1)	3832(1)	26(1)
O(5)	4703(1)	4874(1)	3253(1)	21(1)
C(1)	8414(1)	3384(1)	1285(1)	19(1)
C(2)	9331(1)	3280(1)	2861(1)	20(1)
C(3)	7784(1)	3642(1)	3257(1)	19(1)
C(4)	8263(1)	4556(1)	3957(1)	18(1)
C(5)	10153(1)	4389(1)	2829(1)	19(1)
C(6)	9449(1)	5077(1)	2013(1)	17(1)
C(7)	10651(1)	5254(1)	1260(1)	24(1)
C(8)	7935(1)	4505(1)	1637(1)	17(1)
C(9)	7042(1)	5167(1)	875(1)	20(1)
C(10)	5620(1)	5797(1)	1241(1)	20(1)
C(11)	5957(1)	6181(1)	2219(1)	17(1)
C(12)	6126(1)	7213(1)	2517(1)	19(1)
C(13)	6594(1)	7454(1)	3504(1)	19(1)
C(14)	7354(1)	6557(1)	4056(1)	17(1)
C(15)	8269(1)	6887(1)	4914(1)	21(1)
C(16)	7254(1)	5535(1)	3715(1)	16(1)
C(17)	6184(1)	5256(1)	2906(1)	16(1)
C(18)	6861(1)	4225(1)	2462(1)	17(1)
C(19)	3817(2)	5650(1)	3760(1)	28(1)

Supplementary Table 6: Bond lengths [Å] and angles [°] for swiesler03_sarpong

O(1)-C(1)	1.2030(14)
O(2)-C(1)	1.3638(14)
O(2)-C(2)	1.4465(14)
O(3)-C(5)	1.4312(14)
O(3)-C(4)	1.4581(14)
O(4)-C(13)	1.2277(15)
O(5)-C(19)	1.4257(15)
O(5)-C(17)	1.4599(13)
C(1)-C(8)	1.5174(16)
C(2)-C(5)	1.5283(17)
C(2)-C(3)	1.5298(16)
C(2)-H(2)	1.0000
C(3)-C(18)	1.5453(16)
C(3)-C(4)	1.5467(16)
C(3)-H(3)	1.0000
C(4)-C(16)	1.5134(16)
C(4)-H(4)	1.0000
C(5)-C(6)	1.5497(16)
C(5)-H(5)	1.0000
C(6)-C(7)	1.5284(16)
C(6)-C(8)	1.5669(16)
C(6)-H(6)	1.0000
C(7)-H(7A)	0.9800
C(7)-H(7B)	0.9800
C(7)-H(7C)	0.9800
C(8)-C(9)	1.5463(16)
C(8)-C(18)	1.5545(15)
C(9)-C(10)	1.5504(16)
C(9)-H(9A)	0.9900
C(9)-H(9B)	0.9900
C(10)-C(11)	1.4950(16)
C(10)-H(10A)	0.9900
C(10)-H(10B)	0.9900
C(11)-C(12)	1.3354(17)

C(11)-C(17)	1.5054(16)
C(12)-C(13)	1.4864(16)
C(12)-H(12)	0.9500
C(13)-C(14)	1.4919(16)
C(14)-C(16)	1.3390(16)
C(14)-C(15)	1.4980(16)
C(15)-H(15A)	0.9800
C(15)-H(15B)	0.9800
C(15)-H(15C)	0.9800
C(16)-C(17)	1.5016(15)
C(17)-C(18)	1.5305(15)
C(18)-H(18)	1.0000
C(19)-H(19A)	0.9800
C(19)-H(19B)	0.9800
C(19)-H(19C)	0.9800
C(1)-O(2)-C(2)	115.12(9)
C(5)-O(3)-C(4)	107.48(8)
C(19)-O(5)-C(17)	116.49(9)
O(1)-C(1)-O(2)	118.96(11)
O(1)-C(1)-C(8)	127.83(11)
O(2)-C(1)-C(8)	113.19(10)
O(2)-C(2)-C(5)	112.80(9)
O(2)-C(2)-C(3)	113.09(10)
C(5)-C(2)-C(3)	99.60(9)
O(2)-C(2)-H(2)	110.3
C(5)-C(2)-H(2)	110.3
C(3)-C(2)-H(2)	110.3
C(2)-C(3)-C(18)	107.54(9)
C(2)-C(3)-C(4)	102.94(9)
C(18)-C(3)-C(4)	105.62(9)
C(2)-C(3)-H(3)	113.3
C(18)-C(3)-H(3)	113.3
C(4)-C(3)-H(3)	113.3
O(3)-C(4)-C(16)	107.95(9)
O(3)-C(4)-C(3)	105.45(9)

C(16)-C(4)-C(3)	106.03(9)
O(3)-C(4)-H(4)	112.3
C(16)-C(4)-H(4)	112.3
C(3)-C(4)-H(4)	112.3
O(3)-C(5)-C(2)	102.44(9)
O(3)-C(5)-C(6)	113.37(9)
C(2)-C(5)-C(6)	109.05(9)
O(3)-C(5)-H(5)	110.6
C(2)-C(5)-H(5)	110.6
C(6)-C(5)-H(5)	110.6
C(7)-C(6)-C(5)	110.11(10)
C(7)-C(6)-C(8)	113.43(10)
C(5)-C(6)-C(8)	109.08(9)
C(7)-C(6)-H(6)	108.0
C(5)-C(6)-H(6)	108.0
C(8)-C(6)-H(6)	108.0
C(6)-C(7)-H(7A)	109.5
C(6)-C(7)-H(7B)	109.5
H(7A)-C(7)-H(7B)	109.5
C(6)-C(7)-H(7C)	109.5
H(7A)-C(7)-H(7C)	109.5
H(7B)-C(7)-H(7C)	109.5
C(1)-C(8)-C(9)	111.80(9)
C(1)-C(8)-C(18)	103.06(9)
C(9)-C(8)-C(18)	110.55(9)
C(1)-C(8)-C(6)	106.39(9)
C(9)-C(8)-C(6)	114.11(9)
C(18)-C(8)-C(6)	110.30(9)
C(8)-C(9)-C(10)	114.07(9)
C(8)-C(9)-H(9A)	108.7
C(10)-C(9)-H(9A)	108.7
C(8)-C(9)-H(9B)	108.7
C(10)-C(9)-H(9B)	108.7
H(9A)-C(9)-H(9B)	107.6
C(11)-C(10)-C(9)	109.35(9)
C(11)-C(10)-H(10A)	109.8

C(9)-C(10)-H(10A)	109.8
C(11)-C(10)-H(10B)	109.8
C(9)-C(10)-H(10B)	109.8
H(10A)-C(10)-H(10B)	108.3
C(12)-C(11)-C(10)	127.66(11)
C(12)-C(11)-C(17)	119.07(10)
C(10)-C(11)-C(17)	113.21(10)
C(11)-C(12)-C(13)	120.94(11)
C(11)-C(12)-H(12)	119.5
C(13)-C(12)-H(12)	119.5
O(4)-C(13)-C(12)	121.19(11)
O(4)-C(13)-C(14)	121.03(11)
C(12)-C(13)-C(14)	117.56(10)
C(16)-C(14)-C(13)	117.79(10)
C(16)-C(14)-C(15)	125.13(11)
C(13)-C(14)-C(15)	116.92(10)
C(14)-C(15)-H(15A)	109.5
C(14)-C(15)-H(15B)	109.5
H(15A)-C(15)-H(15B)	109.5
C(14)-C(15)-H(15C)	109.5
H(15A)-C(15)-H(15C)	109.5
H(15B)-C(15)-H(15C)	109.5
C(14)-C(16)-C(17)	121.57(10)
C(14)-C(16)-C(4)	128.18(10)
C(17)-C(16)-C(4)	109.79(9)
O(5)-C(17)-C(16)	109.92(9)
O(5)-C(17)-C(11)	111.00(9)
C(16)-C(17)-C(11)	113.63(9)
O(5)-C(17)-C(18)	103.04(9)
C(16)-C(17)-C(18)	105.66(9)
C(11)-C(17)-C(18)	112.96(9)
C(17)-C(18)-C(3)	105.60(9)
C(17)-C(18)-C(8)	111.99(9)
C(3)-C(18)-C(8)	110.54(9)
C(17)-C(18)-H(18)	109.5
C(3)-C(18)-H(18)	109.5

C(8)-C(18)-H(18)	109.5
O(5)-C(19)-H(19A)	109.5
O(5)-C(19)-H(19B)	109.5
H(19A)-C(19)-H(19B)	109.5
O(5)-C(19)-H(19C)	109.5
H(19A)-C(19)-H(19C)	109.5
H(19B)-C(19)-H(19C)	109.5

Symmetry transformations used to generate equivalent atoms:

Supplementary Table 7: Anisotropic displacement parameters ($Å^2$x 10^3) for swiesler03_sarpong

The anisotropic displacement factor exponent takes the form: $-2\pi^2[h^2a^{*2}U^{11} + ... + 2 h k a^* b^* U^{12}]$

	U^{11}	U^{22}	U^{33}	U^{23}	U^{13}	U^{12}
O(1)	29(1)	24(1)	21(1)	-6(1)	2(1)	1(1)
O(2)	29(1)	17(1)	20(1)	-2(1)	1(1)	4(1)
O(3)	18(1)	22(1)	18(1)	-2(1)	-1(1)	2(1)
O(4)	33(1)	18(1)	27(1)	-5(1)	-5(1)	5(1)
O(5)	17(1)	23(1)	22(1)	-4(1)	6(1)	-5(1)
C(1)	20(1)	19(1)	20(1)	-1(1)	3(1)	-2(1)
C(2)	26(1)	18(1)	17(1)	-1(1)	-1(1)	3(1)
C(3)	23(1)	16(1)	18(1)	1(1)	1(1)	-1(1)
C(4)	20(1)	17(1)	16(1)	1(1)	2(1)	1(1)
C(5)	19(1)	21(1)	18(1)	-2(1)	1(1)	3(1)
C(6)	18(1)	17(1)	18(1)	0(1)	2(1)	-1(1)
C(7)	22(1)	27(1)	23(1)	0(1)	6(1)	-2(1)
C(8)	18(1)	16(1)	16(1)	-1(1)	2(1)	0(1)
C(9)	22(1)	22(1)	16(1)	0(1)	0(1)	2(1)
C(10)	20(1)	22(1)	17(1)	0(1)	-2(1)	1(1)
C(11)	13(1)	21(1)	18(1)	0(1)	1(1)	2(1)
C(12)	18(1)	19(1)	19(1)	2(1)	0(1)	3(1)
C(13)	17(1)	18(1)	21(1)	-2(1)	1(1)	0(1)
C(14)	16(1)	19(1)	17(1)	0(1)	1(1)	-1(1)
C(15)	24(1)	20(1)	20(1)	-1(1)	-4(1)	0(1)
C(16)	15(1)	19(1)	15(1)	1(1)	2(1)	-1(1)
C(17)	15(1)	17(1)	17(1)	-1(1)	2(1)	-2(1)
C(18)	18(1)	15(1)	17(1)	0(1)	2(1)	-2(1)
C(19)	20(1)	34(1)	33(1)	-10(1)	10(1)	-3(1)

Supplementary Table 8: Hydrogen coordinates (x 10^4) and isotropic displacement parameters ($Å^2$x 10^3) for swiesler03_sarpong

	x	y	z	U(eq)
H(2)	9881	2776	3309	24
H(3)	7199	3030	3552	22
H(4)	8160	4321	4623	21
H(5)	11290	4285	2749	23
H(6)	9160	5812	2268	21
H(7A)	10970	4542	1011	36
H(7B)	11553	5632	1534	36
H(7C)	10199	5700	752	36
H(9A)	6689	4657	375	24
H(9B)	7759	5699	593	24
H(10A)	5390	6434	830	23
H(10B)	4703	5309	1231	23
H(12)	5945	7802	2093	22
H(15A)	8729	6233	5207	32
H(15B)	7585	7247	5357	32
H(15C)	9091	7395	4738	32
H(18)	6002	3738	2233	20
H(19A)	3578	6282	3358	43
H(19B)	4413	5895	4314	43
H(19C)	2852	5308	3957	43

Supplementary Table 9: Torsion angles [°] for swiesler03_sarpong

C(2)-O(2)-C(1)-O(1)	-176.74(10)
C(2)-O(2)-C(1)-C(8)	1.96(14)
C(1)-O(2)-C(2)-C(5)	-57.08(13)
C(1)-O(2)-C(2)-C(3)	55.00(13)
O(2)-C(2)-C(3)-C(18)	-45.12(12)
C(5)-C(2)-C(3)-C(18)	74.84(10)
O(2)-C(2)-C(3)-C(4)	-156.37(9)
C(5)-C(2)-C(3)-C(4)	-36.41(10)
C(5)-O(3)-C(4)-C(16)	-97.38(10)
C(5)-O(3)-C(4)-C(3)	15.62(11)
C(2)-C(3)-C(4)-O(3)	14.37(11)
C(18)-C(3)-C(4)-O(3)	-98.30(10)
C(2)-C(3)-C(4)-C(16)	128.71(9)
C(18)-C(3)-C(4)-C(16)	16.04(11)
C(4)-O(3)-C(5)-C(2)	-39.54(11)
C(4)-O(3)-C(5)-C(6)	77.81(11)
O(2)-C(2)-C(5)-O(3)	167.02(9)
C(3)-C(2)-C(5)-O(3)	46.85(10)
O(2)-C(2)-C(5)-C(6)	46.63(12)
C(3)-C(2)-C(5)-C(6)	-73.54(10)
O(3)-C(5)-C(6)-C(7)	132.86(10)
C(2)-C(5)-C(6)-C(7)	-113.73(11)
O(3)-C(5)-C(6)-C(8)	-102.05(11)
C(2)-C(5)-C(6)-C(8)	11.36(12)
O(1)-C(1)-C(8)-C(9)	0.03(17)
O(2)-C(1)-C(8)-C(9)	-178.52(9)
O(1)-C(1)-C(8)-C(18)	118.78(13)
O(2)-C(1)-C(8)-C(18)	-59.78(12)
O(1)-C(1)-C(8)-C(6)	-125.15(13)
O(2)-C(1)-C(8)-C(6)	56.29(12)
C(7)-C(6)-C(8)-C(1)	61.89(12)
C(5)-C(6)-C(8)-C(1)	-61.24(11)
C(7)-C(6)-C(8)-C(9)	-61.87(13)
C(5)-C(6)-C(8)-C(9)	175.00(9)

C(7)-C(6)-C(8)-C(18)	172.99(10)
C(5)-C(6)-C(8)-C(18)	49.87(12)
C(1)-C(8)-C(9)-C(10)	138.14(10)
C(18)-C(8)-C(9)-C(10)	23.94(13)
C(6)-C(8)-C(9)-C(10)	-101.06(12)
C(8)-C(9)-C(10)-C(11)	34.23(14)
C(9)-C(10)-C(11)-C(12)	112.84(13)
C(9)-C(10)-C(11)-C(17)	-64.42(12)
C(10)-C(11)-C(12)-C(13)	-174.67(11)
C(17)-C(11)-C(12)-C(13)	2.44(16)
C(11)-C(12)-C(13)-O(4)	-165.77(12)
C(11)-C(12)-C(13)-C(14)	19.52(16)
O(4)-C(13)-C(14)-C(16)	170.36(11)
C(12)-C(13)-C(14)-C(16)	-14.92(15)
O(4)-C(13)-C(14)-C(15)	-14.04(17)
C(12)-C(13)-C(14)-C(15)	160.69(10)
C(13)-C(14)-C(16)-C(17)	-11.01(16)
C(15)-C(14)-C(16)-C(17)	173.78(10)
C(13)-C(14)-C(16)-C(4)	160.39(11)
C(15)-C(14)-C(16)-C(4)	-14.83(19)
O(3)-C(4)-C(16)-C(14)	-59.38(15)
C(3)-C(4)-C(16)-C(14)	-171.98(11)
O(3)-C(4)-C(16)-C(17)	112.83(10)
C(3)-C(4)-C(16)-C(17)	0.23(12)
C(19)-O(5)-C(17)-C(16)	64.34(13)
C(19)-O(5)-C(17)-C(11)	-62.22(13)
C(19)-O(5)-C(17)-C(18)	176.59(10)
C(14)-C(16)-C(17)-O(5)	-93.21(12)
C(4)-C(16)-C(17)-O(5)	93.97(10)
C(14)-C(16)-C(17)-C(11)	31.85(15)
C(4)-C(16)-C(17)-C(11)	-140.97(10)
C(14)-C(16)-C(17)-C(18)	156.25(10)
C(4)-C(16)-C(17)-C(18)	-16.57(12)
C(12)-C(11)-C(17)-O(5)	97.67(12)
C(10)-C(11)-C(17)-O(5)	-84.82(11)
C(12)-C(11)-C(17)-C(16)	-26.82(15)

C(10)-C(11)-C(17)-C(16)	150.70(10)
C(12)-C(11)-C(17)-C(18)	-147.17(11)
C(10)-C(11)-C(17)-C(18)	30.35(13)
O(5)-C(17)-C(18)-C(3)	-89.08(10)
C(16)-C(17)-C(18)-C(3)	26.27(11)
C(11)-C(17)-C(18)-C(3)	151.07(9)
O(5)-C(17)-C(18)-C(8)	150.54(9)
C(16)-C(17)-C(18)-C(8)	-94.11(11)
C(11)-C(17)-C(18)-C(8)	30.70(13)
C(2)-C(3)-C(18)-C(17)	-135.58(9)
C(4)-C(3)-C(18)-C(17)	-26.17(11)
C(2)-C(3)-C(18)-C(8)	-14.26(12)
C(4)-C(3)-C(18)-C(8)	95.15(10)
C(1)-C(8)-C(18)-C(17)	-177.83(9)
C(9)-C(8)-C(18)-C(17)	-58.22(12)
C(6)-C(8)-C(18)-C(17)	68.92(12)
C(1)-C(8)-C(18)-C(3)	64.71(11)
C(9)-C(8)-C(18)-C(3)	-175.67(9)
C(6)-C(8)-C(18)-C(3)	-48.54(12)

Symmetry transformations used to generate equivalent atoms:

8-2. Supplementary Figure 4: X-ray structure of *Iso*-harringtonolide (25) (CCDC 2293696)

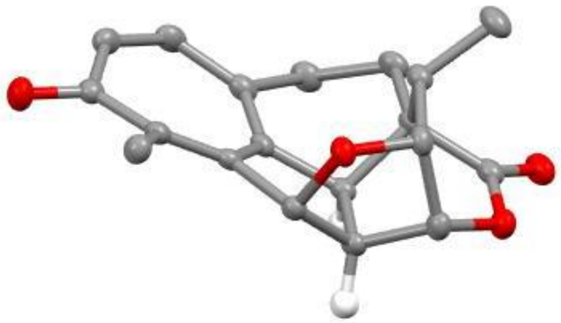

A colorless block 0.25 x 0.11 x 0.05 mm in size was mounted on a Cryoloop with Paratone oil. Data were collected in a nitrogen gas stream at 100(2) K using omega scans. Crystal-to-detector distance was 30.21 mm and exposure time was 0.50 seconds per frame at low angles and 0.75 seconds per frame at high angles, using a scan width of 0.5°. Data collection was 100% complete to 74.000° in θ. A total of 16587 reflections were collected covering the indices -9<=h<=10, -18<=k<=18, -27<=l<=25. 2911 reflections were found to be symmetry independent, with an R_{int} of 0.0354. Indexing and unit cell refinement indicated a primitive, orthorhombic lattice. The space group was found to be P b c a (No. 61). The data were integrated using the CrysAlis^{Pro} 1.172.42.90a software program and scaled using the SCALE3 ABSPACK scaling algorithm. Solution by intrinsic phasing (SHELXT-2015) produced a heavy-atom phasing model consistent with the proposed structure. All non-hydrogen atoms were refined anisotropically by full-matrix least-squares (SHELXL-2014). All hydrogen atoms were placed using a riding model. Their positions were constrained relative to their parent atom using the appropriate HFIX command in SHELXL-2014.

Supplementary Table 10: Crystal data and structure refinement for SWiesler02_Sarpong

Identification code	SWiesler02_Sarpong	
Empirical formula	C19 H18 O4	
Formula weight	310.33	
Temperature	100(2) K	
Wavelength	1.54184 Å	
Crystal system	Orthorhombic	
Space group	P b c a	
Unit cell dimensions	a = 8.6482(2) Å	α= 90°.
	b = 15.0331(2) Å	β= 90°.
	c = 21.8499(3) Å	γ = 90°.
Volume	2840.69(9) $Å^3$	
Z	8	
Density (calculated)	1.451 Mg/m^3	
Absorption coefficient	0.827 mm^{-1}	
F(000)	1312	
Crystal size	0.250 x 0.110 x 0.050 mm^3	
Theta range for data collection	4.046 to 74.496°.	
Index ranges	-9<=h<=10, -18<=k<=18, -27<=l<=25	
Reflections collected	16587	
Independent reflections	2911 [R(int) = 0.0354]	
Completeness to theta = 74.000°	100.0 %	
Absorption correction	Semi-empirical from equivalents	
Max. and min. transmission	1.00000 and 0.53445	
Refinement method	Full-matrix least-squares on F^2	
Data / restraints / parameters	2911 / 0 / 210	
Goodness-of-fit on F^2	1.056	
Final R indices [I>2sigma(I)]	R1 = 0.0361, wR2 = 0.0907	
R indices (all data)	R1 = 0.0403, wR2 = 0.0932	
Extinction coefficient	n/a	
Largest diff. peak and hole	0.231 and -0.196 e.$Å^{-3}$	

Supplementary Table 11: Atomic coordinates (x 10^4) and equivalent isotropic displacement parameters ($Å^2$x 10^3) for swiesler02_sarpong

U(eq) is defined as one third of the trace of the orthogonalized U^{ij} tensor.

	x	y	z	U(eq)
O(1)	277(1)	2951(1)	6232(1)	29(1)
O(2)	578(1)	4271(1)	6669(1)	25(1)
O(3)	9215(1)	5903(1)	5637(1)	30(1)
O(4)	4184(1)	5324(1)	7117(1)	21(1)
C(1)	1149(2)	3542(1)	6376(1)	23(1)
C(2)	2868(2)	3591(1)	6277(1)	20(1)
C(3)	3555(2)	2790(1)	5929(1)	24(1)
C(4)	4545(2)	3060(1)	5367(1)	25(1)
C(5)	5559(2)	3855(1)	5511(1)	21(1)
C(6)	7175(2)	3830(1)	5398(1)	24(1)
C(7)	8248(2)	4479(1)	5476(1)	24(1)
C(8)	8134(2)	5376(1)	5727(1)	21(1)
C(9)	6847(2)	5657(1)	6125(1)	19(1)
C(10)	7249(2)	6419(1)	6541(1)	24(1)
C(11)	5388(1)	5310(1)	6106(1)	18(1)
C(12)	4808(2)	4552(1)	5781(1)	18(1)
C(13)	3104(2)	4449(1)	5886(1)	19(1)
C(14)	2568(2)	5279(1)	6236(1)	20(1)
C(15)	4064(1)	5675(1)	6497(1)	19(1)
C(16)	1692(2)	4966(1)	6805(1)	22(1)
C(17)	3052(2)	4631(1)	7190(1)	22(1)
C(18)	3579(2)	3725(1)	6928(1)	21(1)
C(19)	3137(2)	2963(1)	7357(1)	29(1)

Supplementary Table 12: Bond lengths [Å] and angles [°] for swiesler02_sarpong

O(1)-C(1)	1.2077(16)
O(2)-C(1)	1.3622(16)
O(2)-C(16)	1.4522(15)
O(3)-C(8)	1.2413(16)
O(4)-C(17)	1.4379(15)
O(4)-C(15)	1.4585(14)
C(1)-C(2)	1.5044(19)
C(2)-C(3)	1.5428(17)
C(2)-C(13)	1.5605(16)
C(2)-C(18)	1.5634(17)
C(3)-C(4)	1.5505(18)
C(3)-H(3A)	0.9900
C(3)-H(3B)	0.9900
C(4)-C(5)	1.5154(18)
C(4)-H(4A)	0.9900
C(4)-H(4B)	0.9900
C(5)-C(12)	1.3662(17)
C(5)-C(6)	1.420(2)
C(6)-C(7)	1.3567(19)
C(6)-H(6)	0.9500
C(7)-C(8)	1.4590(18)
C(7)-H(7)	0.9500
C(8)-C(9)	1.4733(17)
C(9)-C(11)	1.3662(18)
C(9)-C(10)	1.5030(17)
C(10)-H(10A)	0.9800
C(10)-H(10B)	0.9800
C(10)-H(10C)	0.9800
C(11)-C(12)	1.4340(16)
C(11)-C(15)	1.5295(16)
C(12)-C(13)	1.4997(18)
C(13)-C(14)	1.5351(16)
C(13)-H(13)	1.0000
C(14)-C(16)	1.5289(17)

C(14)-C(15)	1.5336(17)
C(14)-H(14)	1.0000
C(15)-H(15)	1.0000
C(16)-C(17)	1.5308(18)
C(16)-H(16)	1.0000
C(17)-C(18)	1.5462(17)
C(17)-H(17)	1.0000
C(18)-C(19)	1.5285(17)
C(18)-H(18)	1.0000
C(19)-H(19A)	0.9800
C(19)-H(19B)	0.9800
C(19)-H(19C)	0.9800
C(1)-O(2)-C(16)	115.77(10)
C(17)-O(4)-C(15)	108.39(9)
O(1)-C(1)-O(2)	119.26(12)
O(1)-C(1)-C(2)	127.97(12)
O(2)-C(1)-C(2)	112.76(11)
C(1)-C(2)-C(3)	114.42(11)
C(1)-C(2)-C(13)	104.39(10)
C(3)-C(2)-C(13)	108.99(10)
C(1)-C(2)-C(18)	105.27(10)
C(3)-C(2)-C(18)	113.48(10)
C(13)-C(2)-C(18)	109.85(9)
C(2)-C(3)-C(4)	113.44(10)
C(2)-C(3)-H(3A)	108.9
C(4)-C(3)-H(3A)	108.9
C(2)-C(3)-H(3B)	108.9
C(4)-C(3)-H(3B)	108.9
H(3A)-C(3)-H(3B)	107.7
C(5)-C(4)-C(3)	111.24(10)
C(5)-C(4)-H(4A)	109.4
C(3)-C(4)-H(4A)	109.4
C(5)-C(4)-H(4B)	109.4
C(3)-C(4)-H(4B)	109.4
H(4A)-C(4)-H(4B)	108.0

C(12)-C(5)-C(6)	124.29(12)
C(12)-C(5)-C(4)	114.76(12)
C(6)-C(5)-C(4)	120.86(11)
C(7)-C(6)-C(5)	129.21(12)
C(7)-C(6)-H(6)	115.4
C(5)-C(6)-H(6)	115.4
C(6)-C(7)-C(8)	131.71(12)
C(6)-C(7)-H(7)	114.1
C(8)-C(7)-H(7)	114.1
O(3)-C(8)-C(7)	118.65(12)
O(3)-C(8)-C(9)	118.61(11)
C(7)-C(8)-C(9)	122.53(11)
C(11)-C(9)-C(8)	124.78(11)
C(11)-C(9)-C(10)	121.48(11)
C(8)-C(9)-C(10)	113.64(11)
C(9)-C(10)-H(10A)	109.5
C(9)-C(10)-H(10B)	109.5
H(10A)-C(10)-H(10B)	109.5
C(9)-C(10)-H(10C)	109.5
H(10A)-C(10)-H(10C)	109.5
H(10B)-C(10)-H(10C)	109.5
C(9)-C(11)-C(12)	129.88(11)
C(9)-C(11)-C(15)	122.55(11)
C(12)-C(11)-C(15)	107.44(10)
C(5)-C(12)-C(11)	131.07(12)
C(5)-C(12)-C(13)	117.02(11)
C(11)-C(12)-C(13)	110.47(10)
C(12)-C(13)-C(14)	106.80(10)
C(12)-C(13)-C(2)	107.31(10)
C(14)-C(13)-C(2)	111.08(10)
C(12)-C(13)-H(13)	110.5
C(14)-C(13)-H(13)	110.5
C(2)-C(13)-H(13)	110.5
C(16)-C(14)-C(15)	103.63(10)
C(16)-C(14)-C(13)	107.77(10)
C(15)-C(14)-C(13)	104.25(10)

C(16)-C(14)-H(14)	113.4
C(15)-C(14)-H(14)	113.4
C(13)-C(14)-H(14)	113.4
O(4)-C(15)-C(11)	109.64(9)
O(4)-C(15)-C(14)	105.34(9)
C(11)-C(15)-C(14)	106.57(10)
O(4)-C(15)-H(15)	111.7
C(11)-C(15)-H(15)	111.7
C(14)-C(15)-H(15)	111.7
O(2)-C(16)-C(14)	112.59(10)
O(2)-C(16)-C(17)	112.63(10)
C(14)-C(16)-C(17)	99.59(10)
O(2)-C(16)-H(16)	110.5
C(14)-C(16)-H(16)	110.5
C(17)-C(16)-H(16)	110.5
O(4)-C(17)-C(16)	102.96(10)
O(4)-C(17)-C(18)	113.38(10)
C(16)-C(17)-C(18)	108.26(10)
O(4)-C(17)-H(17)	110.7
C(16)-C(17)-H(17)	110.7
C(18)-C(17)-H(17)	110.7
C(19)-C(18)-C(17)	111.07(10)
C(19)-C(18)-C(2)	111.29(10)
C(17)-C(18)-C(2)	109.55(10)
C(19)-C(18)-H(18)	108.3
C(17)-C(18)-H(18)	108.3
C(2)-C(18)-H(18)	108.3
C(18)-C(19)-H(19A)	109.5
C(18)-C(19)-H(19B)	109.5
H(19A)-C(19)-H(19B)	109.5
C(18)-C(19)-H(19C)	109.5
H(19A)-C(19)-H(19C)	109.5
H(19B)-C(19)-H(19C)	109.5

Symmetry transformations used to generate equivalent atoms:

Supplementary Table 13: Anisotropic displacement parameters (Å^{2}x 10^3)for swiesler02_sarpong

The anisotropic displacement factor exponent takes the form: $-2\pi^2[h^2a^{*2}U^{11} + ... + 2 h k a^* b^* U^{12}]$

	U^{11}	U^{22}	U^{33}	U^{23}	U^{13}	U^{12}
O(1)	29(1)	22(1)	36(1)	4(1)	-5(1)	-8(1)
O(2)	21(1)	22(1)	32(1)	1(1)	2(1)	-4(1)
O(3)	25(1)	29(1)	35(1)	1(1)	8(1)	-3(1)
O(4)	24(1)	21(1)	19(1)	-1(1)	1(1)	-4(1)
C(1)	26(1)	19(1)	23(1)	5(1)	-4(1)	-2(1)
C(2)	24(1)	15(1)	20(1)	2(1)	-2(1)	-2(1)
C(3)	32(1)	14(1)	25(1)	0(1)	-2(1)	-1(1)
C(4)	35(1)	18(1)	24(1)	-4(1)	1(1)	0(1)
C(5)	30(1)	17(1)	17(1)	1(1)	1(1)	1(1)
C(6)	33(1)	18(1)	22(1)	-1(1)	5(1)	6(1)
C(7)	24(1)	25(1)	22(1)	2(1)	6(1)	6(1)
C(8)	21(1)	22(1)	20(1)	4(1)	0(1)	2(1)
C(9)	22(1)	16(1)	19(1)	2(1)	0(1)	2(1)
C(10)	22(1)	22(1)	27(1)	-3(1)	0(1)	-1(1)
C(11)	23(1)	14(1)	16(1)	2(1)	0(1)	3(1)
C(12)	23(1)	16(1)	16(1)	4(1)	0(1)	0(1)
C(13)	23(1)	15(1)	19(1)	2(1)	-2(1)	-1(1)
C(14)	20(1)	15(1)	24(1)	1(1)	0(1)	0(1)
C(15)	20(1)	16(1)	20(1)	1(1)	1(1)	1(1)
C(16)	21(1)	19(1)	27(1)	-1(1)	3(1)	-2(1)
C(17)	23(1)	22(1)	20(1)	1(1)	3(1)	-4(1)
C(18)	24(1)	19(1)	20(1)	3(1)	-2(1)	-1(1)
C(19)	38(1)	25(1)	24(1)	6(1)	-4(1)	-6(1)

Supplementary Table 14: Hydrogen coordinates (x 10^4) and isotropic displacement parameters ($Å^2$x 10^3) for swiesler02_sarpong

	x	y	z	U(eq)
H(3A)	2699	2402	5789	28
H(3B)	4204	2439	6213	28
H(4A)	5203	2552	5243	30
H(4B)	3855	3207	5020	30
H(6)	7560	3281	5245	29
H(7)	9258	4319	5345	28
H(10A)	7295	6971	6303	35
H(10B)	8256	6308	6732	35
H(10C)	6457	6474	6860	35
H(13)	2544	4398	5487	22
H(14)	1968	5706	5978	24
H(15)	4042	6340	6494	22
H(16)	1174	5480	7011	27
H(17)	2744	4571	7629	26
H(18)	4731	3735	6888	25
H(19A)	3637	3051	7755	43
H(19B)	3479	2397	7180	43
H(19C)	2012	2954	7411	43

Supplementary Table 15: Torsion angles [°] for swiesler02_sarpong

C(16)-O(2)-C(1)-O(1)	-177.61(11)
C(16)-O(2)-C(1)-C(2)	2.65(14)
O(1)-C(1)-C(2)-C(3)	2.04(18)
O(2)-C(1)-C(2)-C(3)	-178.26(10)
O(1)-C(1)-C(2)-C(13)	121.08(14)
O(2)-C(1)-C(2)-C(13)	-59.21(12)
O(1)-C(1)-C(2)-C(18)	-123.23(14)
O(2)-C(1)-C(2)-C(18)	56.47(12)
C(1)-C(2)-C(3)-C(4)	127.52(12)
C(13)-C(2)-C(3)-C(4)	11.10(15)
C(18)-C(2)-C(3)-C(4)	-111.66(12)
C(2)-C(3)-C(4)-C(5)	41.64(15)
C(3)-C(4)-C(5)-C(12)	-49.09(15)
C(3)-C(4)-C(5)-C(6)	127.70(12)
C(12)-C(5)-C(6)-C(7)	-6.0(2)
C(4)-C(5)-C(6)-C(7)	177.51(13)
C(5)-C(6)-C(7)-C(8)	3.8(2)
C(6)-C(7)-C(8)-O(3)	-165.68(14)
C(6)-C(7)-C(8)-C(9)	19.5(2)
O(3)-C(8)-C(9)-C(11)	156.62(12)
C(7)-C(8)-C(9)-C(11)	-28.60(19)
O(3)-C(8)-C(9)-C(10)	-19.76(16)
C(7)-C(8)-C(9)-C(10)	155.02(12)
C(8)-C(9)-C(11)-C(12)	9.2(2)
C(10)-C(9)-C(11)-C(12)	-174.69(11)
C(8)-C(9)-C(11)-C(15)	-175.29(11)
C(10)-C(9)-C(11)-C(15)	0.83(17)
C(6)-C(5)-C(12)-C(11)	-13.0(2)
C(4)-C(5)-C(12)-C(11)	163.68(12)
C(6)-C(5)-C(12)-C(13)	-177.79(11)
C(4)-C(5)-C(12)-C(13)	-1.13(16)
C(9)-C(11)-C(12)-C(5)	16.4(2)
C(15)-C(11)-C(12)-C(5)	-159.65(12)
C(9)-C(11)-C(12)-C(13)	-178.03(12)

C(15)-C(11)-C(12)-C(13)	5.93(13)
C(5)-C(12)-C(13)-C(14)	175.53(10)
C(11)-C(12)-C(13)-C(14)	7.70(13)
C(5)-C(12)-C(13)-C(2)	56.35(13)
C(11)-C(12)-C(13)-C(2)	-111.48(10)
C(1)-C(2)-C(13)-C(12)	178.70(10)
C(3)-C(2)-C(13)-C(12)	-58.63(13)
C(18)-C(2)-C(13)-C(12)	66.27(12)
C(1)-C(2)-C(13)-C(14)	62.30(12)
C(3)-C(2)-C(13)-C(14)	-175.03(10)
C(18)-C(2)-C(13)-C(14)	-50.13(14)
C(12)-C(13)-C(14)-C(16)	-127.51(10)
C(2)-C(13)-C(14)-C(16)	-10.80(14)
C(12)-C(13)-C(14)-C(15)	-17.83(12)
C(2)-C(13)-C(14)-C(15)	98.88(11)
C(17)-O(4)-C(15)-C(11)	-103.27(11)
C(17)-O(4)-C(15)-C(14)	11.05(12)
C(9)-C(11)-C(15)-O(4)	-80.15(13)
C(12)-C(11)-C(15)-O(4)	96.25(11)
C(9)-C(11)-C(15)-C(14)	166.33(11)
C(12)-C(11)-C(15)-C(14)	-17.27(12)
C(16)-C(14)-C(15)-O(4)	17.55(12)
C(13)-C(14)-C(15)-O(4)	-95.13(10)
C(16)-C(14)-C(15)-C(11)	133.99(10)
C(13)-C(14)-C(15)-C(11)	21.31(12)
C(1)-O(2)-C(16)-C(14)	54.62(14)
C(1)-O(2)-C(16)-C(17)	-57.03(14)
C(15)-C(14)-C(16)-O(2)	-156.73(10)
C(13)-C(14)-C(16)-O(2)	-46.63(13)
C(15)-C(14)-C(16)-C(17)	-37.20(11)
C(13)-C(14)-C(16)-C(17)	72.90(11)
C(15)-O(4)-C(17)-C(16)	-35.29(12)
C(15)-O(4)-C(17)-C(18)	81.44(12)
O(2)-C(16)-C(17)-O(4)	163.93(9)
C(14)-C(16)-C(17)-O(4)	44.42(11)
O(2)-C(16)-C(17)-C(18)	43.62(13)

C(14)-C(16)-C(17)-C(18)	-75.89(11)
O(4)-C(17)-C(18)-C(19)	138.09(11)
C(16)-C(17)-C(18)-C(19)	-108.33(12)
O(4)-C(17)-C(18)-C(2)	-98.56(12)
C(16)-C(17)-C(18)-C(2)	15.02(13)
C(1)-C(2)-C(18)-C(19)	58.63(13)
C(3)-C(2)-C(18)-C(19)	-67.23(14)
C(13)-C(2)-C(18)-C(19)	170.49(11)
C(1)-C(2)-C(18)-C(17)	-64.59(12)
C(3)-C(2)-C(18)-C(17)	169.55(10)
C(13)-C(2)-C(18)-C(17)	47.27(13)

Symmetry transformations used to generate equivalent atoms:

9. Computational Studies

9-1. Computational Methods

The range separated dispersion corrected wB97x-D density functional[10] was used in conjunction with the double-z def2-SVP basis set,[11] to optimize the geometry of all stationary points. Additional single points energy correction was carried out with the newer generation meta-augmented range separated density functional wB97m-V[12] that employs the Vydrov and van Voorhis VV10 dispersion correction,[13] together with the triple-z def2-TZVPP basis set. The VV10 dispersion corrected family of functionals developed by the Head-Gordon group have been demonstrated to be one of the most robust functionals for assessment of main group thermochemistry and for describing non-covalent interactions.[12–14,15] All calculations included the integral equation formalism variant of the polarizable continuum model (IEF-PCM), with the SMD solvation model to account for solvation effects (solvent = dichloromethane).[16] Conformational sampling was performed manually. *Gaussian16* version C.01 was employed for all density functional theory (DFT) geometry optimization calculations, using the default "ultrafine" pruned (99,590) grid for numerical integration of the exchange-correlation functional and its derivatives.[17] Single point corrections were carried our using *ORCA 5.0.4*.[18] Vibrational frequency calculations were used to verify that stationary points were either minima or first-order saddle points on the corresponding potential energy surface. Additional intrinsic reaction coordinate (IRC) calculations were performed to ensure that the transition state structures connected to their appropriate initial and final geometries.[19] The computed thermochemistry data were further corrected following Grimme's quasi-harmonic (QHA)[20] model for entropy with a frequency cut-off value of 100.0 cm^{-1} using the *GoodVibes* program at 213.15 K (-60 ℃).[21] Additionally, *GoodVibes* applied 1 M standard concentration corrections to all individual calculations to account for reactions in solution (i.e. change in standard concentration from 1 atm to 1 M).[22] XYZ coordinate files were also generated using *GoodVibes*.

Example Gaussian16 optimization input command line:

```
# opt freq=noraman wB97x-D/def2-SVP scrf=(smd,solvent=dichloromethane)
```

Note: For transition state optimization the keyword "opt" is replaced by "opt=(ts,calcfc,noeigen)"

Example ORCA 5.0.4 single point correction input command line:

```
! pal8 wb97m-v def2-tzvpp def2-tzvpp/C

% maxcore 4000

%CPCM SMD TRUE

        SMDSOLVENT "DICHLOROMETHANE"

END
```

9-2. Benchmarking

To support the accuracy of the density functional approximations (DFAs), we performed an additional benchmarking study using single point energy corrections on the initial addition of $TMSCHN_2$ to substrate **20-[B]** (**Supplementary Figure 5**) on the geometries obtained using the ωB97x-D/def2-SVP level of theory. In general, all functionals performed very similarly at predicting the regioselectivity of the transformation, with VV10 dispersion corrected functionals wB97x-V and wB97m-V providing the best results (**Supplementary Table 16**).

Supplementary Table 16: Benchmarking results; (a) calculations were performed using *ORCA 5.0.4*, unless otherwise stated; (b) calculations were performed using *Gaussian 16*.

**Functional**[(a)]	**Basis Set**	**$\Delta\Delta G_{(213K)}$**
**wB97x-D**[(b)]	def2-SVP	-0.2
**wB97x-D**[(b)]	def2-TZVPP	0.1
**wB97x-V**	def2-TZVPP	-0.4
**wB97m-V**	**def2-TZVPP**	**-0.5**
**M06-2X-D3**	def2-TZVPP	-1.1
**B2GP-PLYP-D4**	def2-TZVPP	-0.1
**DLPNO-CCSD(T)**	def2-TZVPP	-0.3
**Experimental Selectivity**		**-0.6**

Supplementary Figure 5: Transition states used for the selectivity benchmarking; NOTE: All possible conformers were considered.

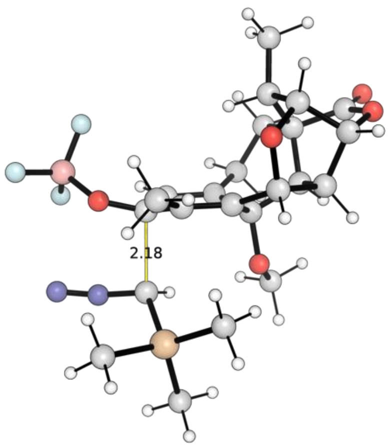

TS1a-[B]

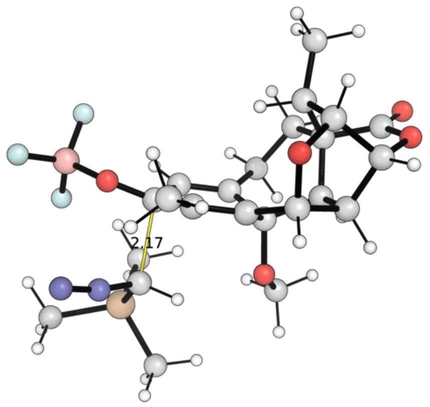

TS1b-[B]

9-3. Potential Energy Surface Using CH_2N_2 and BF_3•OEt_2

Supplementary Figure 6: Summary of the potential energy surface for the reaction of **20** with CH_2N_2

A ωB97X-D/def2-TZVPP(SMD=CH2Cl2)//ωB97X-D/def2-SVP(SMD=CH2Cl2) level of theory

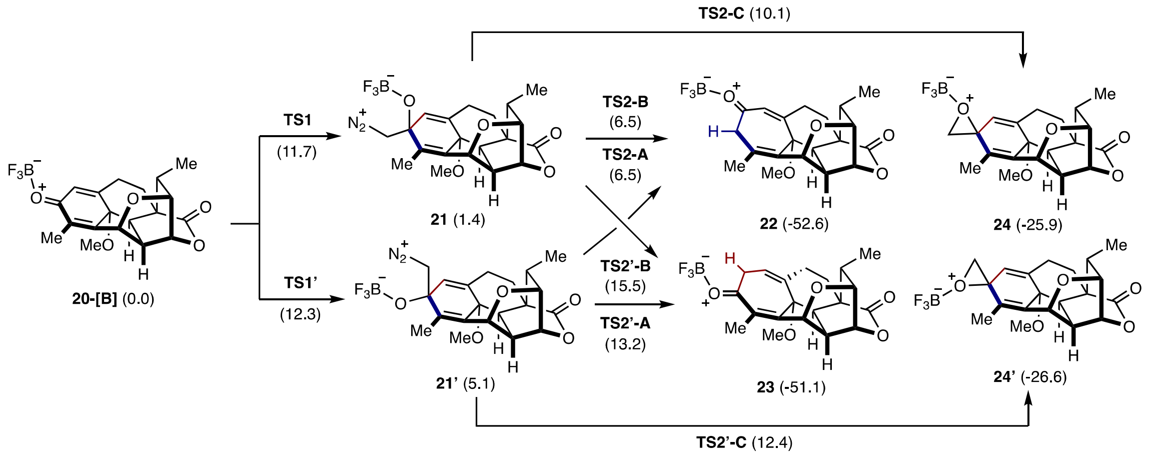

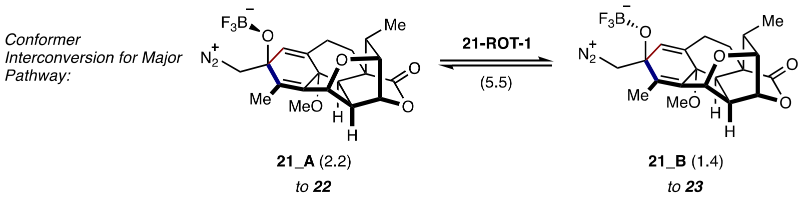

B ωB97M-V/def2-TZVPP(SMD=CH2Cl2)//ωB97X-D/def2-SVP(SMD=CH2Cl2) level of theory

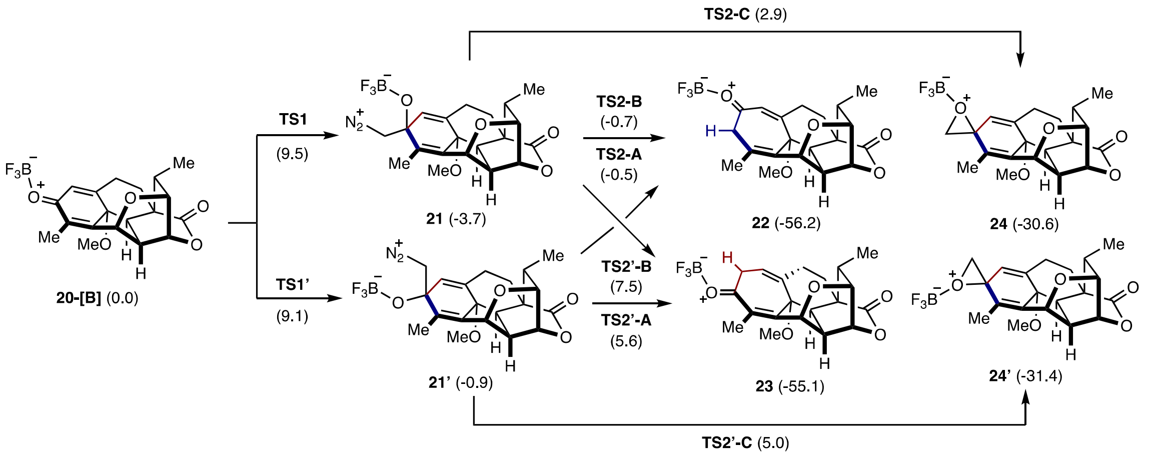

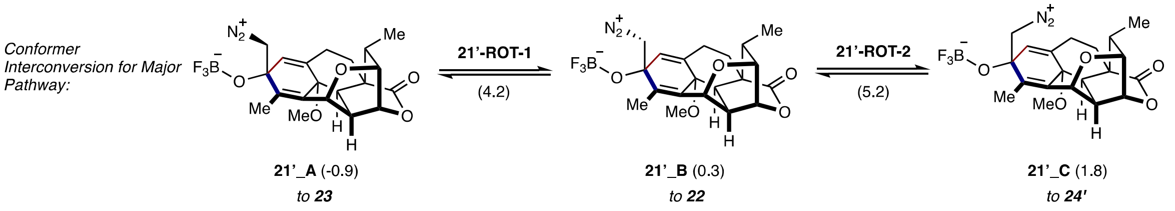

Supplementary Table 17: Thermochemistry for the reaction of **21'-A** with CH_2N_2 computed at the ωB97x-D/def2-TZVPP(SMD=CH_2Cl_2)//ωB97x-D/def2-SVP(SMD=CH_2Cl_2); ΔE = relative energy, ΔZPE = relative zero-point energy, ΔH = relative enthalpy, TΔS = temperature multiplied by relative entropy, $\Delta G_{(213K)}$ = relative Gibbs free energy computed at 213K

Entry	ΔE	ΔZPE	ΔH	TΔS	$\Delta G_{(213K)}$
**20-[B]+CH_2N_2**	0.0	0.0	0.0	0.0	0.0
**TS1**	2.1	-1.2	2.2	-8.4	11.8
**21**	-10	-12.5	3.9	-8.4	1.3
**TS2-A**	-3.1	-4	2.3	-7.9	6.4
**TS2-B**	-2.9	-3.8	2.1	-7.8	6.2
**TS2-C**	-0.1	-0.1	2.6	-8.3	10
**TS1'**	2.2	-0.7	2.2	-8.9	12.4
**21'**	-6.7	-9.2	3.9	-8.9	5.1
**TS2'-A**	3.3	3.2	2.1	-8.4	13
**TS2'-B**	6.3	6.7	1.6	-8.1	15.3
**TS2'-C**	2.3	2.7	2.4	-8.3	12.2
**22+N2**	-56.2	-55.2	1.7	-1.9	-52.7
**23+N2**	-55.2	-54.7	2.1	-2.1	-51.1
**24+N2**	-29.8	-29.2	2	-2.3	-25.9
**24'+N2**	-30.7	-30.2	2	-2.6	-26.4

Supplementary Table 18: Thermochemistry for intermediate **21'** conformer interconversion computed at the ωB97x-D/def2-TZVPP(SMD=CH_2Cl_2)//ωB97x-D/def2-SVP(SMD=CH_2Cl_2); ΔE = relative energy, ΔZPE = relative zero-point energy, ΔH = relative enthalpy, TΔS = temperature multiplied by relative entropy, $\Delta G_{(213K)}$ = relative Gibbs free energy computed at 213K

Entry	ΔE	ΔZPE	ΔH	TΔS	$\Delta G_{(213K)}$
**20-A+CH_2N_2**	0.0	0.0	0.0	0.0	0.0
**21-A**	-9.2	-12.2	3.9	-8.5	2.2
**21-ROT-1**	-5.6	-8	3.3	-9	5.6
**21-B**	-10.1	-12.6	3.9	-8.6	1.4

Supplementary Table 19: Thermochemistry for the reaction of **21'-A** with CH_2N_2 computed at the ωB97m-V/def2-TZVPP(SMD=CH_2Cl_2)//ωB97x-D/def2-SVP(SMD=CH_2Cl_2); ΔE = relative energy, ΔZPE = relative zero-point energy, ΔH = relative enthalpy, TΔS = temperature multiplied by relative entropy, $\Delta G_{(213K)}$ = relative Gibbs free energy computed at 213K

Entry	ΔE	ΔZPE	ΔH	TΔS	$\Delta G_{(213K)}$
**20-[B]+CH_2N_2**	0.0	0.0	0.0	0.0	0.0
**TS1**	-0.6	2.2	0.7	-8.8	9.5
**21**	-15.6	3.9	-12.7	-9.0	-3.7
**TS2-A**	-10.5	2.3	-9.0	-8.6	-0.5
**TS2-B**	-10.5	2.1	-9.1	-8.4	-0.7
**TS2-C**	-7.5	2.6	-5.7	-8.7	2.9
**TS1'**	-1.1	2.2	0.3	-8.8	9.1
**21'**	-12.9	3.9	-10.0	-9.1	-0.9
**TS2'-A**	-4.2	2.1	-3.0	-8.6	5.6
**TS2'-B**	-1.7	1.6	-0.7	-8.2	7.5
**TS2'-C**	-5.2	2.4	-3.6	-8.7	5.0
**22+N2**	-59.8	1.7	-58.1	-1.9	-56.2
**23+N2**	-59.2	2.1	-57.2	-2.1	-55.1
**24+N2**	-34.7	2.0	-33.1	-2.5	-30.6
**24'+N2**	-35.7	2.0	-34.0	-2.6	-31.4

Supplementary Table 20: Thermochemistry for intermediate **21'** conformer interconversion computed at the ωB97m-V/def2-TZVPP(SMD=CH_2Cl_2)//ωB97x-D/def2-SVP(SMD=CH_2Cl_2); ΔE = relative energy, ΔZPE = relative zero-point energy, ΔH = relative enthalpy, TΔS = temperature multiplied by relative entropy, $\Delta G_{(213K)}$ = relative Gibbs free energy computed at 213K

Entry	ΔE	ΔZPE	ΔH	TΔS	$\Delta G_{(213K)}$
**20'-A+CH_2N_2**	0.0	0.0	0.0	0.0	0.0
**21'-A**	-13.0	3.9	-10.1	-9.1	-0.9
**21'-ROT-1**	-7.8	3.7	-5.5	-9.7	-4.2
**21'-B**	-11.6	3.9	-8.6	-8.9	0.3
**21'-ROT-2**	-6.9	3.7	-4.6	-9.8	5.2
**21'-C**	-10.3	3.9	-7.4	-9.2	1.8

Supplementary Figure 7: 3D structure of key transition states

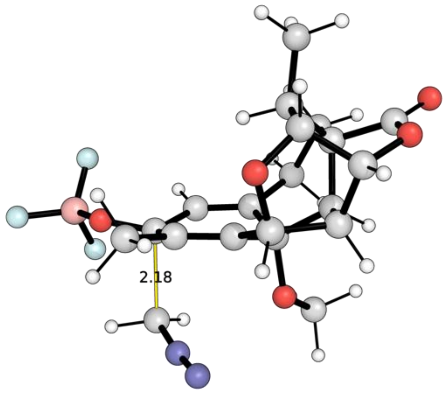

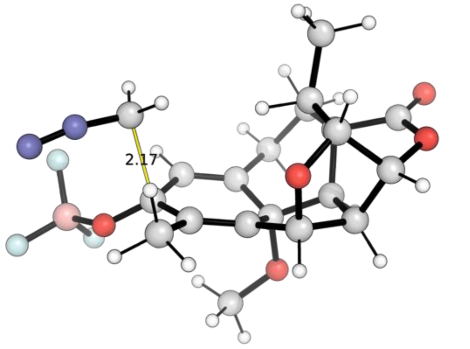

TS1_A_1

TS1’_1

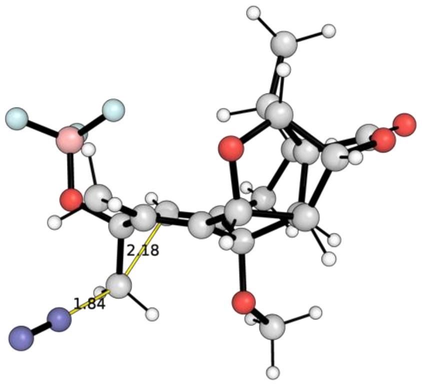

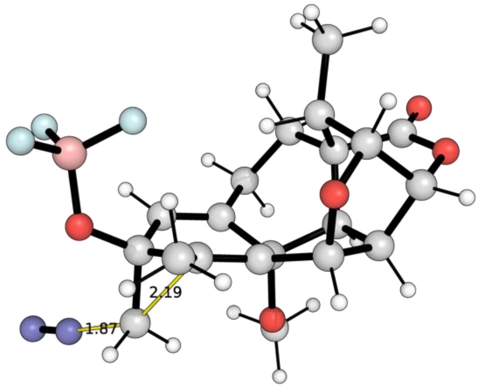

TS2_A_1

TS2_B_1

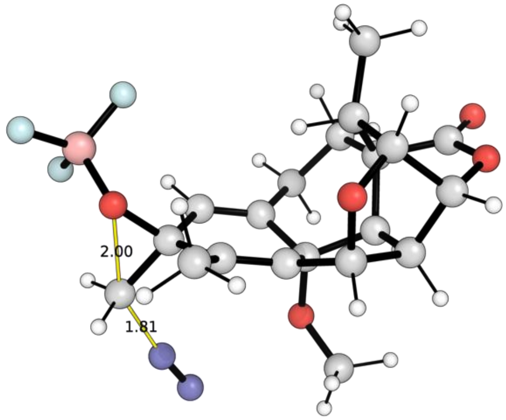

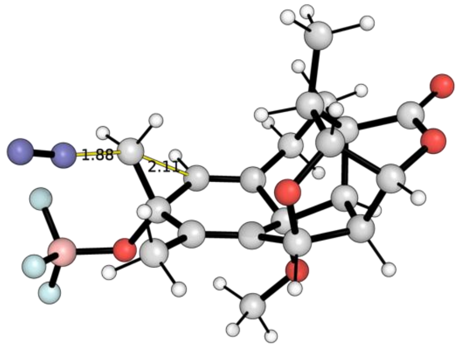

TS2_C_1

TS2’_A_1

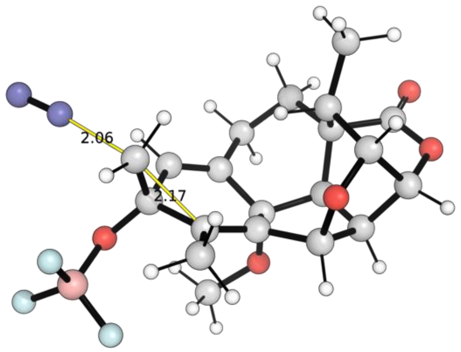

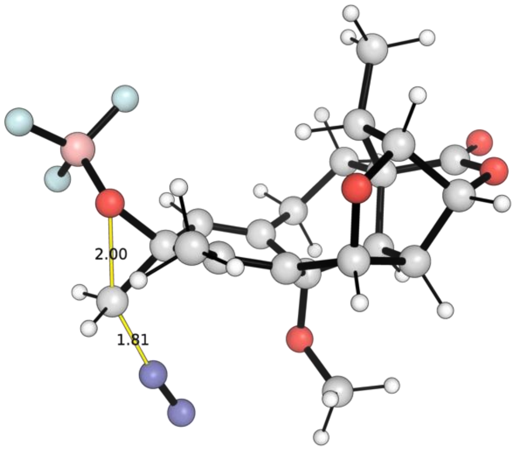

TS2’_B_1

TS2’_C_1

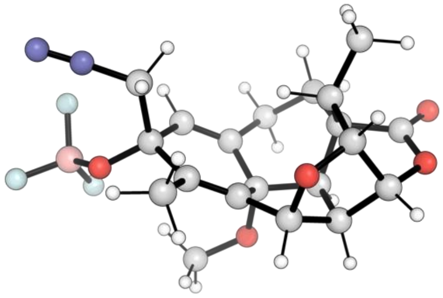

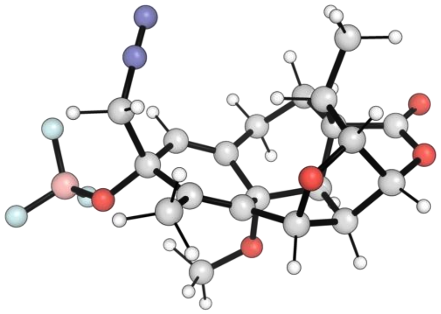

21’-ROT-1

21’-ROT-2

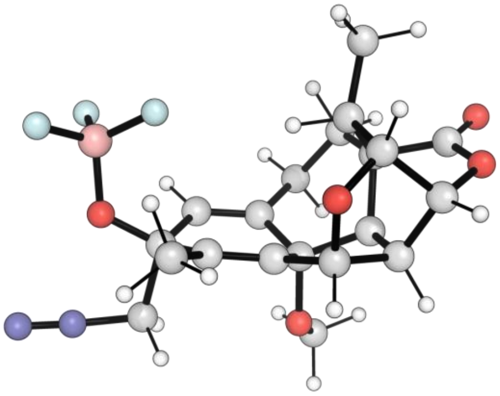

21-ROT-1

9-4. Potential Energy Surface Using $TMSCHN_2$ and $AlCl_3$

An expansive potential energy surface was computed using the best experimentally preforming catalyst $AlCl_3$ (**Supplementary Figure 8**). The rate determining and product determining step was found to be the initial addition of the diazo compound $TMSCHN_2$. Compared to the reaction with CH_2N_2, addition of the sterically more hindered reagent was found to occur preferentially on the *si*-face of the prochiral ketone via **TS1a-[Al]** and **TS1b-[Al]**, as such only these pathways were considered for further investigations. The pathway emerging from **TS1a-[Al]**, was the found to preferentially rearrange via **TS2a-[Al]**, leading to product **28b-[Al]**, while the intermediates arising from crossing **TS1b-[Al]**, preferentially rearrange via **TS2b-[Al]**', leading to the formation of the undesired product **28b-[Al]**. As **TS1b-[Al]** and **TS1b-[Al]** were found to be isoenergetic at the chosen level of theory, the predict product distribution upon rearomatization of 1:1, was found to closely match experimental ratio of 1:1.2 in favor of the undesired product **25** (emerging form **28b-[Al]**). Detailed thermochemical data can be found in **Supplementary Table 21**.

Supplementary Figure 8: Summary of the potential energy surface for the reaction of **20-[Al]** with $TMSCHN_2$

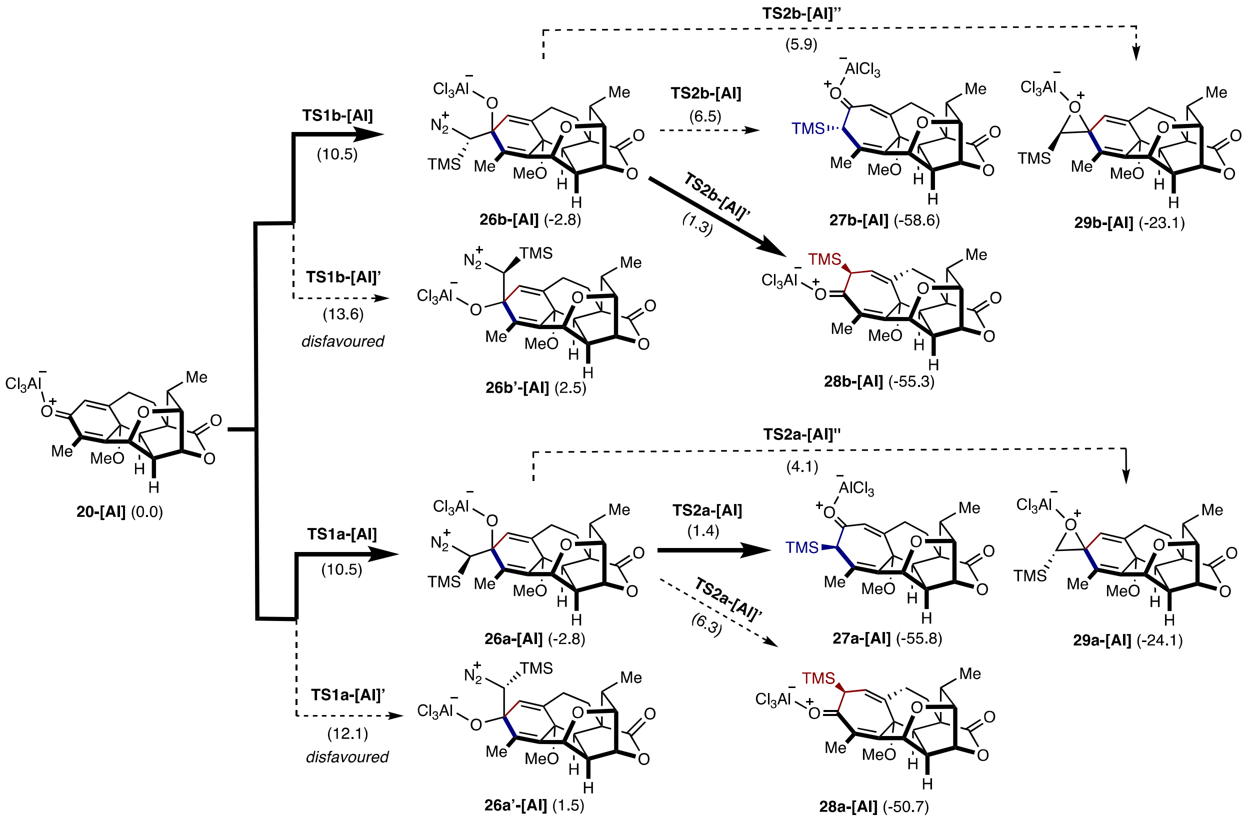

Supplementary Table 21: Thermochemistry for the reaction of **20-[Al]** with $TMSCHN_2$ computed at the ωB97m-V/def2-TZVPP(SMD=CH_2Cl_2)//ωB97x-D/def2-SVP(SMD=CH_2Cl_2); ΔE = relative energy, ΔZPE = relative zero-point energy, ΔH = relative enthalpy, TΔS = temperature multiplied by relative entropy, $\Delta G_{(213K)}$ = relative Gibbs free energy computed at 213K

Entry	ΔE	ΔZPE	ΔH	TΔS	$\Delta G_{(213K)}$
**20-[Al]+TMS**	0	0	0	0	0
**TS1a-[Al]**	-0.9	1.5	0	-10.5	10.5
**TS1a-[Al]'**	0.5	2.5	2	-11.6	13.6
**TS1b-[Al]**	-1.1	1.6	-0.1	-10.7	10.5
**TS1b-[Al]'**	-0.7	2.3	0.7	-11.4	12.1
**TS2a-[Al]**	-9.5	1.1	-8.8	-10.2	1.4
**TS2a-[Al]'**	-5.4	1.8	-4.2	-10.5	6.3
**TS2a-[Al]''**	-7.2	1.4	-6.3	-10.4	4.1
**TS2b-[Al]**	-4.9	1.4	-4	-10.5	6.5
**TS2b-[Al]'**	-10.5	1.6	-9.5	-10.7	1.3
**TS2b-[Al]''**	-5.7	1.5	-4.8	-10.6	5.9
**26a-[Al]**	-16	2.9	-13.8	-11	-2.8
**26a-[Al]'**	-12.2	3.2	-9.9	-11.4	1.5
**26b-[Al]**	-15.8	2.9	-13.7	-11	-2.8
**26b-[Al]'**	-10.9	3.1	-8.7	-11.2	2.5
**27a-[Al]+N2**	-61	1	-60	-4.2	-55.8
**27b-[Al]+N2**	-63.7	0.9	-62.8	-4.2	-58.6
**28a-[Al]+N2**	-56.5	1.5	-55.1	-4.4	-50.7
**28b-[Al]+N2**	-60.6	1.2	-59.4	-4.1	-55.3
**29a-[Al]+N2**	-28.3	0.3	-27.8	-3.8	-24.1
**29b-[Al]+N2**	-28	0.7	-27.3	-4.2	-23.1

Supplementary Figure 9: 3D structure of key transition states

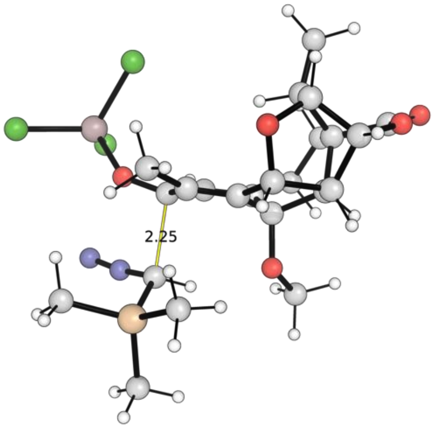

TS1a-[Al]_1

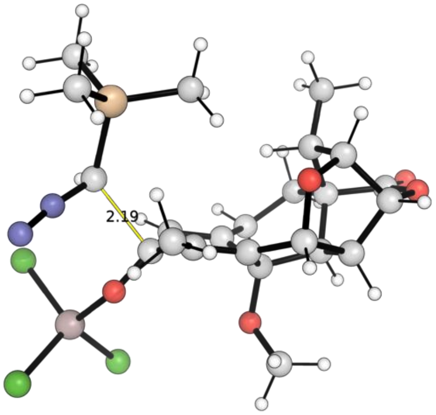

TS1a-[Al]'_1

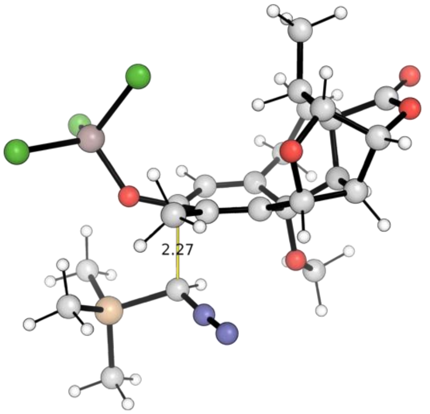

TS1b-[Al]_1

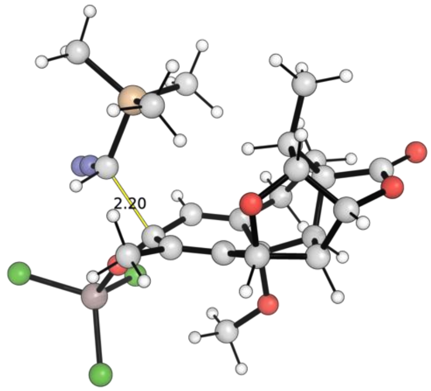

TS1b-[Al]'_1

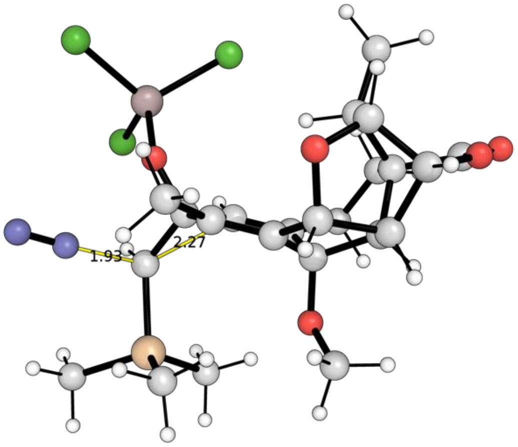

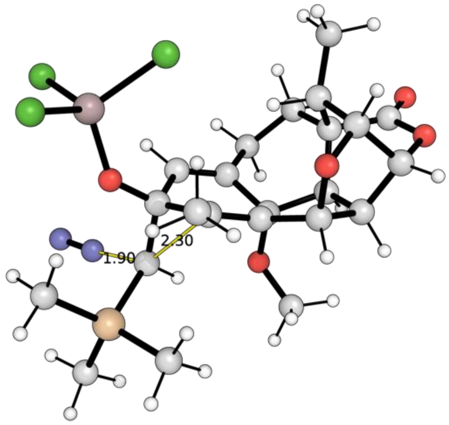

TS2a-[Al]_1 **TS2a-[Al]’_1**

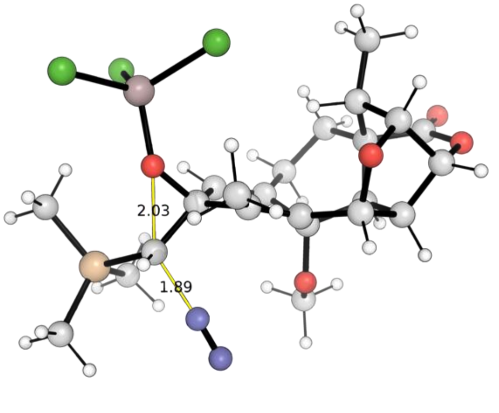

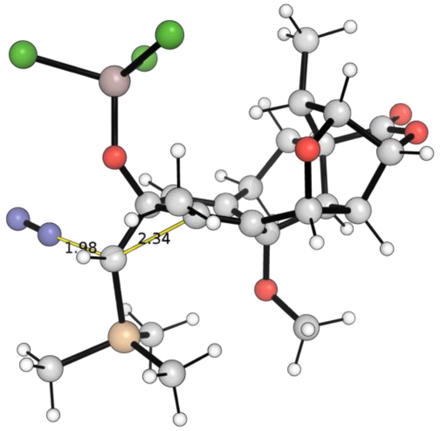

TS2a-[Al]’’_1 **TS2b-[Al]_1**

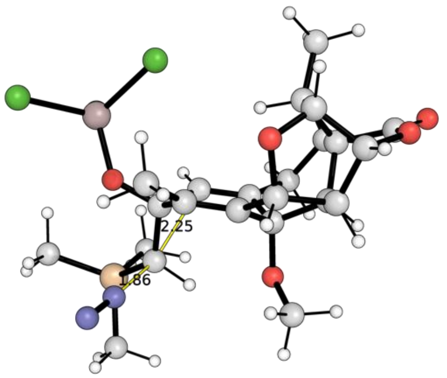

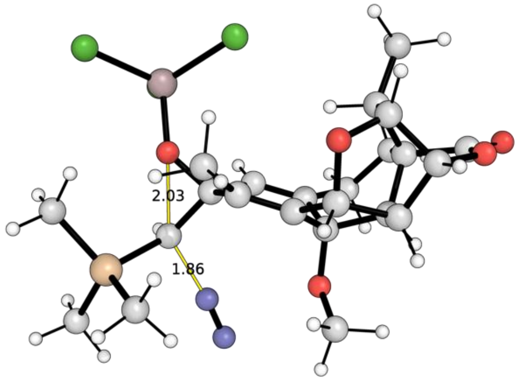

TS2b-[Al]’_1 **TS2b-[Al]’’_1**

9-5. Potential Energy Surface Using TMSCHN$_2$ and BF$_3$•OEt$_2$

A similar analysis was performed for the reaction with the less selective BF$_3$·Et$_2$O catalyst, focusing only on the key transition states (**Supplementary Figure 10**). An overall similar general outcome was observed, however the product determining transition state structure **TS1**, was found to be 0.5 kcal/mol lower in energy for **TS1b-[B]**, leading to the preferential formation of **28b-[B]** over vs **27a-[B]**, with a predicted ration of 3.3 : 1, which is in good agreement with the experimental overserved selectivity of 3.7 : 1 in favor of the undesired product **25**. Detailed thermochemical data can be found in **Supplementary Table 22**.

Supplementary Figure 10: Summary of the potential energy surface for the reaction of **20-[B]** with TMSCHN$_2$. Local minima that have not been specifically computed are shown in grey.

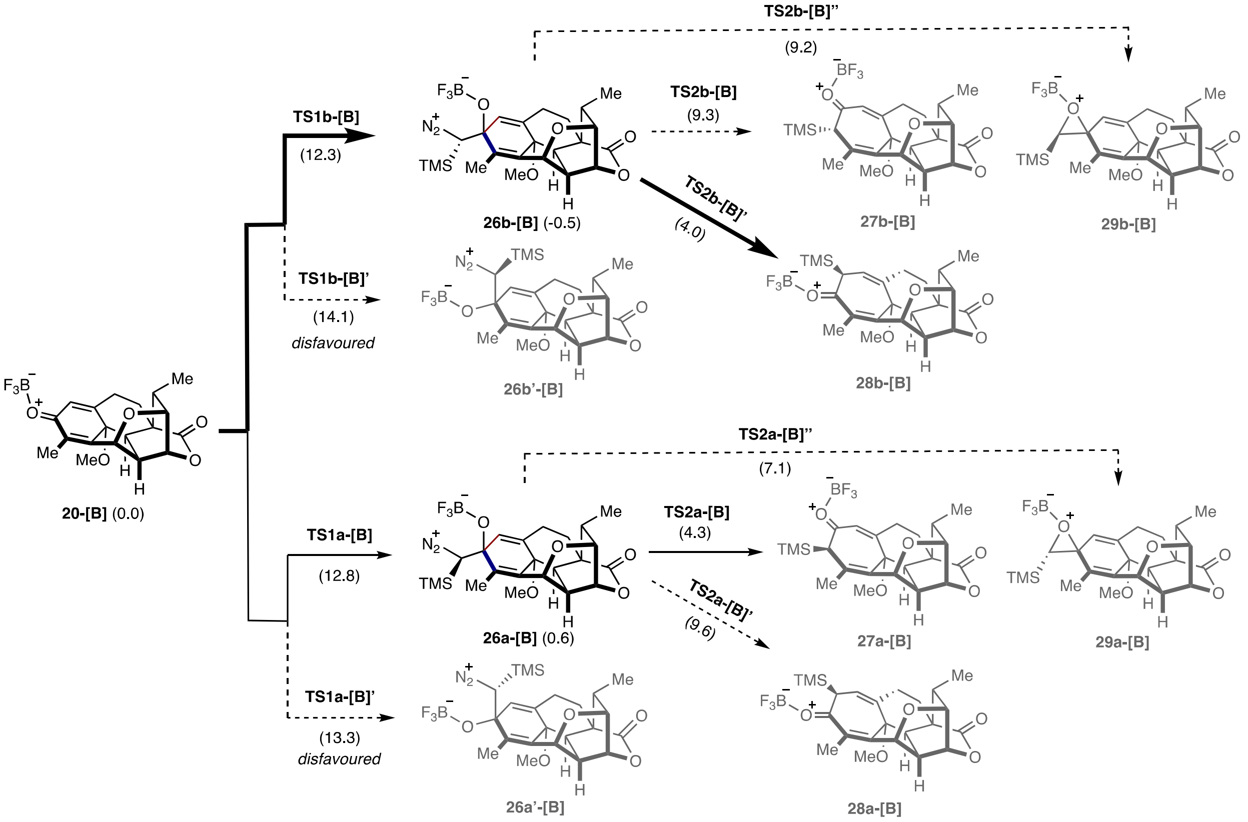

Supplementary Table 22: Thermochemistry for the reaction of **20-[B]** with $TMSCHN_2$ computed at the ωB97m-V/def2-TZVPP(SMD=CH_2Cl_2)//ωB97x-D/def2-SVP(SMD=CH_2Cl_2); ΔE = relative energy, ΔZPE = relative zero-point energy, ΔH = relative enthalpy, TΔS = temperature multiplied by relative entropy, $\Delta G_{(213K)}$ = relative Gibbs free energy computed at 213K

Entry	ΔE	ΔZPE	ΔH	TΔS	$\Delta G_{(213K)}$
**20-[B]+TMS**	0.0	0.0	0.0	0.0	0.0
**TS1a-[B]**	1.2	1.8	2.3	-10.5	12.8
**TS1a-[B]’**	1.0	2.3	2.4	-10.9	13.3
**26a-[B]**	-12.5	3.0	-10.2	-10.8	0.6
**TS2a-[B]**	-1.7	1.5	-0.8	-10.4	9.6
**TS2a-[B]'**	-6.7	1.3	-5.9	-10.2	4.3
**TS2a-[B]’’**	-3.8	1.3	-3.0	-10.2	7.1
**TS1b-[B]**	0.1	2.1	1.3	-11.0	12.3
**TS1b-[B]’**	1.4	2.4	2.8	-11.2	14.1
**26b-[B]**	-13.0	2.7	-11.0	-10.6	-0.5
**TS2b-[B]**	-2.0	1.5	-1.1	-10.4	9.3
**TS2b-[B]'**	-7.5	1.6	-6.5	-10.4	4.0
**TS2b-[B]’’**	-2.1	1.5	-1.1	-10.3	9.2

Supplementary Figure 11: 3D structure of key transition states

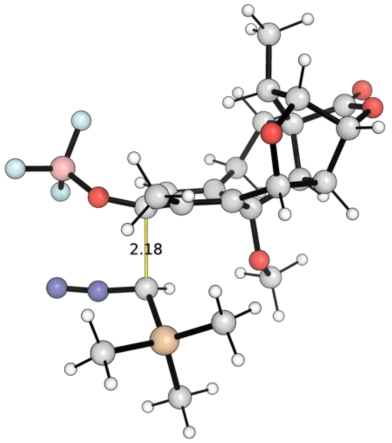

TS1a-[B]_1

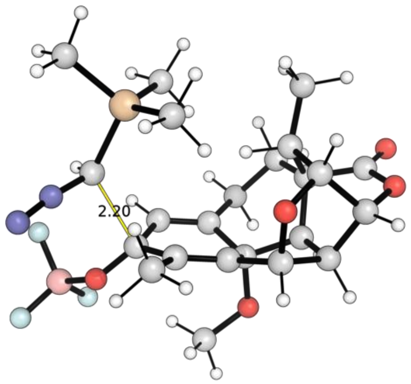

TS1a-[B]’_1

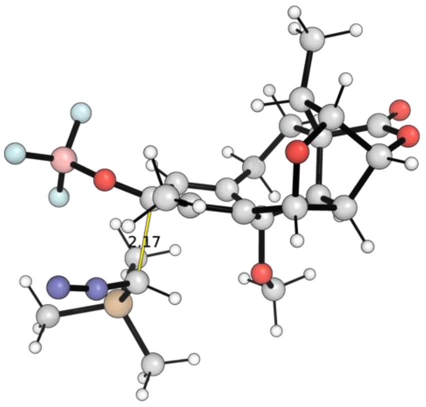

TS1b-[B]_1

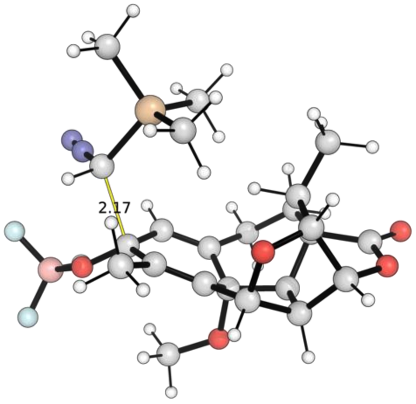

TS1b-[B]'_1

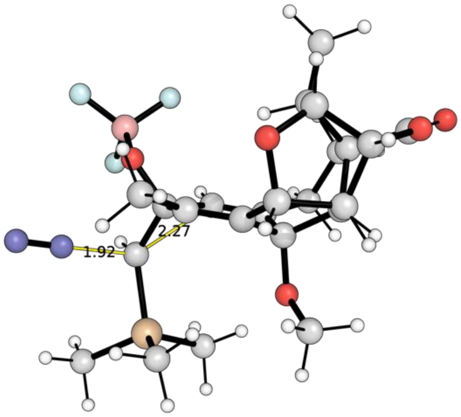

TS2a-[B]_1

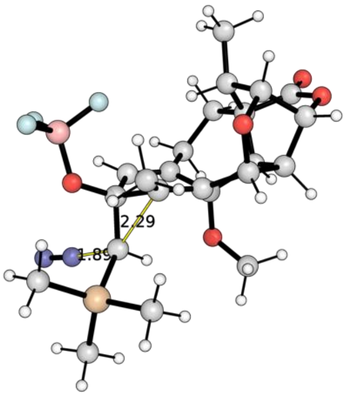

TS2a-[B]'_1

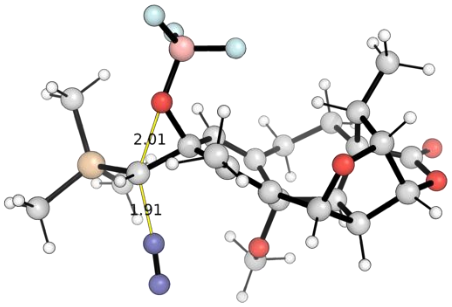

TS2a-[B]''_1

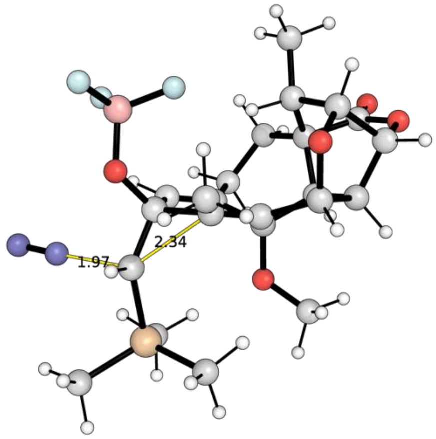

TS2b-[B]_1

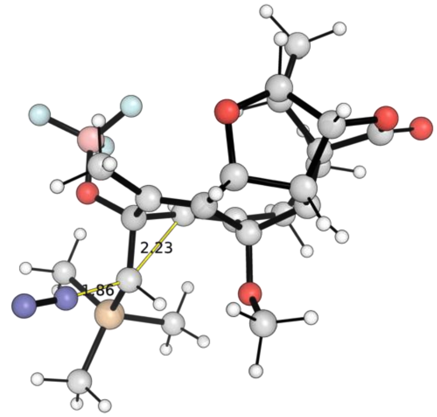

TS2b-[B]’_1

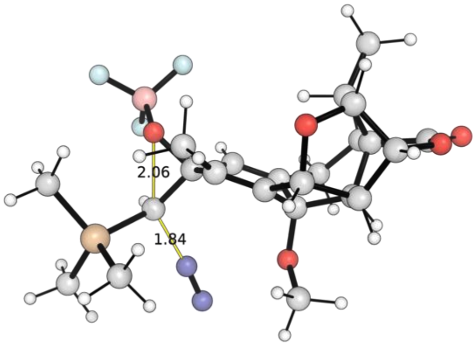

TS2-[B]’’_1

9-6. NCIPLOT of Key Transition States

Non-covalent interactions were visualized using *NCIPLOT*.[23] The wavefunction (.wfn) files were generated from the ORCA checkpoint files (.gbw) by first generating a molden files (.molden), which were then converted to the necessary .wfn files using the *Molden2AIM* program version 5.1.0.[24]

Supplementary Figure 12: NCI interactions the initial within competing **TS1** transition state structures for $AlCl_3$ and BF_3 Lewis acid catalyzed version

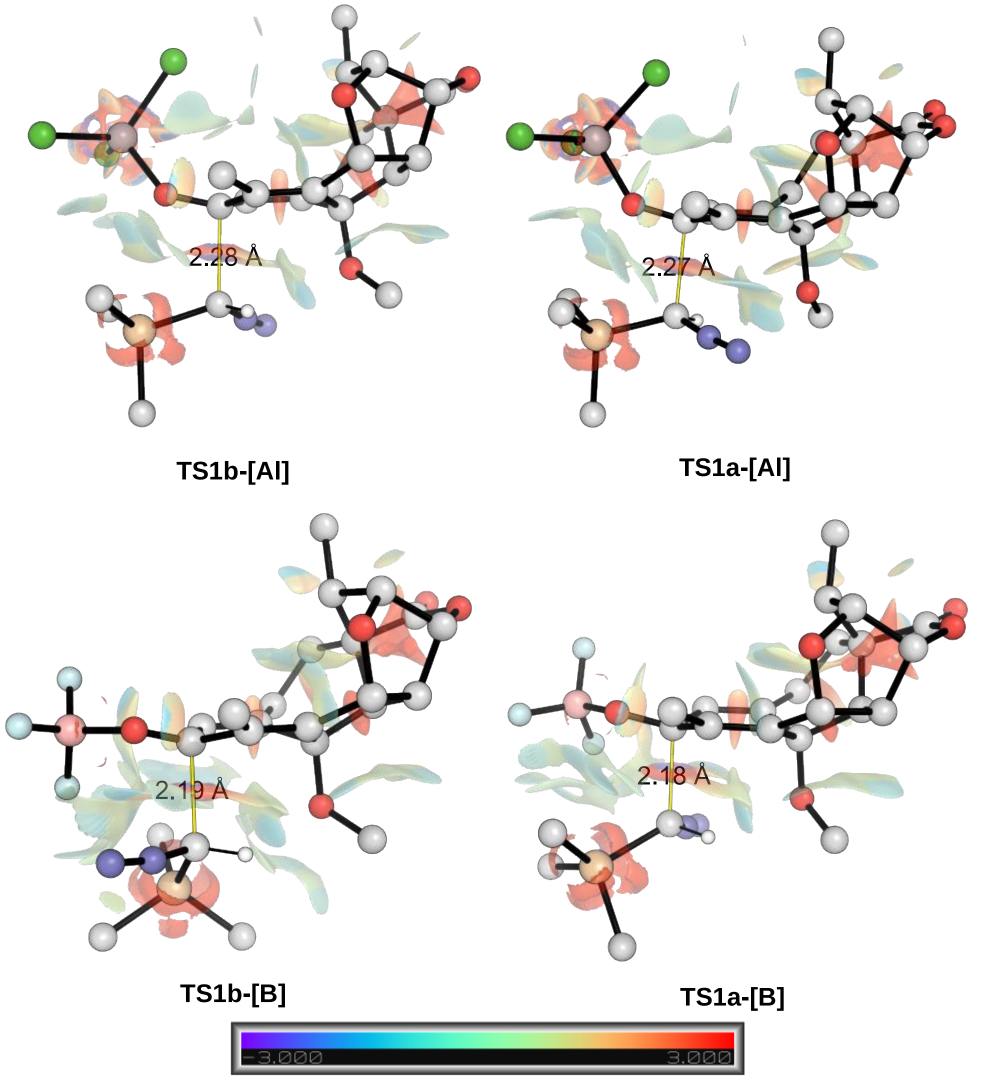

9-7. Lewis Acid Coordination Thermochemistry

Supplementary Figure 13: Diagram showing the thermochemistry of BF_3 coordination to various Lewis Basic coordination sites of compound **20** (Energies are shown in kcal/mol)

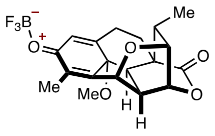

20'-A

0.0

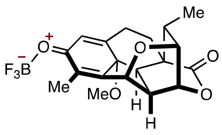

20'-B

5.2

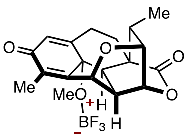

20'-C

9.7

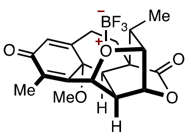

20'-D

3.9

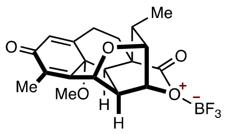

20'-E

13.2

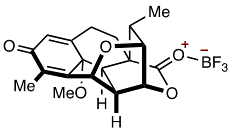

20'-F

3.1

9-8. Thermochemistry

Legend:

E_{spc} = energy obtained in the single-point energy correction

E = energy obtained in the geometry optimization

ZPE = zero point energy

H_{SPC} = enthalpy corrected with E_{SPC}

T·S = temperature multiplied by entropy with no corrections

T·qh-S = temperature multiplied by entropy with quasi-harmonic S correction

G(T) = Gibbs free energy corrected only with E_{SPC}

qh-G(T) = Gibbs free energy corrected with E_{SPC} and quasi-harmonic S correction

ν_{imag} = imaginary frequencies

Supplementary Table 23: Compiled thermochemical data for structures computed at the ωB97m-V/def2-TZVPP(SMD=CH_2Cl_2)//ωB97x-D/def2-SVP(SMD=CH_2Cl_2) level of theory

Name	E_{spc}	*E*	*ZPE*	H_{SPC}	*T·S*	*T·qh-S*	*G(T)*	*qh-G(T)*	ν_{imag}
20'-B_4	-1436.911283	-1435.311882	0.381112	-1436.517470	0.041964	0.040769	-1436.559434	-1436.558239	
20'-B_5	-1436.910295	-1435.310564	0.380820	-1436.516687	0.042386	0.041004	-1436.559073	-1436.557690	
20'-B_6	-1436.914240	-1435.315585	0.380644	-1436.520687	0.042527	0.041188	-1436.563214	-1436.561875	
20'-C_1	-1436.906763	-1435.309013	0.381089	-1436.501755	0.074295	0.071822	-1436.576050	-1436.573577	
20'-C_2	-1436.906874	-1435.308416	0.381254	-1436.501690	0.074995	0.071999	-1436.576685	-1436.573688	
20'-C_3	-1436.905562	-1435.306586	0.381635	-1436.500333	0.072945	0.071031	-1436.573278	-1436.571364	
20'-C_4	-1436.905866	-1435.308601	0.381034	-1436.500823	0.074511	0.072065	-1436.575334	-1436.572889	

20'-C_5	-1436.906019	-1435.307981	0.381447	-1436.500792	0.073722	0.071516	-1436.574514	-1436.572307	
20'-C_6	-1436.907619	-1435.309133	0.381742	-1436.502107	0.073893	0.071537	-1436.576000	-1436.573643	
20'-D_1	-1436.916298	-1435.318680	0.380575	-1436.511515	0.075718	0.072718	-1436.587234	-1436.584233	
20'-D_2	-1436.912689	-1435.314594	0.380940	-1436.507748	0.074740	0.072246	-1436.582488	-1436.579994	
20'-D_3	-1436.913041	-1435.315186	0.381347	-1436.507887	0.073919	0.071673	-1436.581806	-1436.579560	
20'-E_1	-1436.898931	-1435.300319	0.379110	-1436.494972	0.078216	0.074443	-1436.573188	-1436.569416	
20'-E_2	-1436.895766	-1435.296841	0.379615	-1436.491590	0.076701	0.073634	-1436.568292	-1436.565224	
20'-E_3	-1436.895806	-1435.296449	0.379842	-1436.491544	0.075999	0.073198	-1436.567544	-1436.564743	
20'-F_1	-1436.917256	-1435.315945	0.380582	-1436.512472	0.076194	0.072907	-1436.588666	-1436.585379	
20'-F_2	-1436.914254	-1435.312499	0.380399	-1436.509666	0.076210	0.072916	-1436.585876	-1436.582582	
20'-F_3	-1436.913917	-1435.312019	0.381106	-1436.508904	0.075042	0.072187	-1436.583945	-1436.581091	
20-Al_1	-2735.546489	-2733.859874	0.372692	-2735.159015	0.048086	0.045689	-2735.207101	-2735.204705	
20-Al_2	-2735.543334	-2733.857596	0.372454	-2735.156099	0.047960	0.045622	-2735.204059	-2735.201721	
20-Al_3	-2735.542876	-2733.855038	0.372594	-2735.155446	0.048454	0.045927	-2735.203900	-2735.201373	
20-Al_4	-2735.539560	-2733.853691	0.373156	-2735.151836	0.047457	0.045219	-2735.199294	-2735.197055	
20-Al_5	-2735.538989	-2733.852792	0.372575	-2735.151666	0.047906	0.045579	-2735.199571	-2735.197244	
20-Al_6	-2735.543321	-2733.855637	0.372788	-2735.155797	0.048538	0.045858	-2735.204335	-2735.201655	
20-B_1	-1436.921960	-1435.321616	0.380282	-1436.528616	0.043121	0.041622	-1436.571737	-1436.570238	
20-B_2	-1436.918754	-1435.317725	0.380824	-1436.525037	0.042723	0.041331	-1436.567759	-1436.566368	
20-B_3	-1436.918236	-1435.317169	0.380733	-1436.524572	0.042713	0.041367	-1436.567285	-1436.565939	
21'-ROT-1	-1585.680965	-1583.900499	0.418140	-1585.248912	0.043966	0.042962	-1585.292878	-1585.291875	-72.65
21'-ROT-2	-1585.679500	-1583.898920	0.418207	-1585.247438	0.044177	0.042870	-1585.291616	-1585.290309	-68.09

21'_A_1	-1585.681506	-1583.900637	0.419090	-1585.248291	0.044525	0.043365	-1585.292817	-1585.291656
21'_A_2	-1585.678309	-1583.895555	0.418933	-1585.245246	0.044828	0.043493	-1585.290074	-1585.288739
21'_A_3	-1585.689236	-1583.908367	0.418579	-1585.256246	0.045397	0.043961	-1585.301644	-1585.300208
21'_A_4	-1585.685661	-1583.902262	0.419356	-1585.252145	0.044992	0.043492	-1585.297138	-1585.295637
21'_B_1	-1585.686763	-1583.906410	0.418370	-1585.253924	0.045863	0.044136	-1585.299786	-1585.298060
21'_B_2	-1585.683679	-1583.900233	0.418613	-1585.250760	0.045137	0.043724	-1585.295896	-1585.294484
21'_B_3	-1585.686998	-1583.905287	0.418660	-1585.253880	0.046279	0.044398	-1585.300158	-1585.298278
21'_C_1	-1585.685302	-1583.906619	0.418698	-1585.252302	0.044897	0.043663	-1585.297199	-1585.295964
21'_C_2	-1585.681618	-1583.899810	0.418937	-1585.248531	0.044874	0.043455	-1585.293405	-1585.291986
21'_C_3	-1585.683345	-1583.901466	0.418331	-1585.250620	0.045498	0.043989	-1585.296118	-1585.294608
21'_C_4	-1585.684235	-1583.902703	0.418425	-1585.251479	0.045138	0.043768	-1585.296617	-1585.295248
21_A_1	-1585.687261	-1583.867875	0.419046	-1585.253977	0.044787	0.043611	-1585.298764	-1585.297588
21_A_2	-1585.687061	-1583.868154	0.419293	-1585.253552	0.044646	0.043553	-1585.298197	-1585.297105
21_A_3	-1585.691905	-1583.913188	0.418965	-1585.258594	0.045425	0.043945	-1585.304018	-1585.302539
21_A_4	-1585.692266	-1583.913506	0.418452	-1585.259513	0.045011	0.043761	-1585.304525	-1585.303275
21_A_5	-1585.691842	-1583.911873	0.418611	-1585.258741	0.045798	0.044157	-1585.304540	-1585.302898
21_A_6	-1585.691895	-1583.912210	0.418198	-1585.259215	0.045647	0.044104	-1585.304862	-1585.303319
21_B_1	-1585.691940	-1583.913185	0.418860	-1585.258856	0.044721	0.043580	-1585.303578	-1585.302437
21_B_2	-1585.684899	-1583.867467	0.419252	-1585.251479	0.044639	0.043443	-1585.296117	-1585.294922
21_B_3	-1585.692113	-1583.913405	0.418667	-1585.259158	0.044798	0.043681	-1585.303956	-1585.302839
21_B_4	-1585.689885	-1583.911711	0.418442	-1585.257052	0.045378	0.043964	-1585.302429	-1585.301016
21_B_5	-1585.693607	-1583.912947	0.418612	-1585.260509	0.045817	0.044144	-1585.306326	-1585.304653

21_B_6	-1585.693662	-1583.913678	0.418393	-1585.260803	0.045731	0.044103	-1585.306534	-1585.304905
22	-1476.216993	-1474.587150	0.409261	-1475.794183	0.044137	0.042553	-1475.838320	-1475.836736
23	-1476.216015	-1474.586366	0.409944	-1475.792683	0.043780	0.042266	-1475.836463	-1475.834949
24'_1	-1476.177127	-1474.546937	0.410068	-1475.754093	0.042331	0.041139	-1475.796425	-1475.795232
24'_2	-1476.175326	-1474.544223	0.409643	-1475.752629	0.042703	0.041318	-1475.795332	-1475.793947
24'_3	-1476.173376	-1474.541687	0.409603	-1475.750640	0.042776	0.041477	-1475.793416	-1475.792117
24'_4	-1476.177309	-1474.544796	0.409319	-1475.754773	0.043176	0.041735	-1475.797949	-1475.796508
24'_5	-1476.179062	-1474.547774	0.409835	-1475.756158	0.042598	0.041374	-1475.798756	-1475.797532
24'_6	-1476.175786	-1474.543552	0.409129	-1475.753292	0.043717	0.042088	-1475.797010	-1475.795381
24_1	-1476.177206	-1474.545957	0.409657	-1475.754419	0.042976	0.041624	-1475.797395	-1475.796044
24_2	-1476.176825	-1474.545316	0.409695	-1475.753948	0.043144	0.041765	-1475.797092	-1475.795714
26a-Al'_1	-3292.968990	-3290.931151	0.512921	-3292.436067	0.056931	0.054706	-3292.492998	-3292.490773
26a-Al'_10	-3292.964091	-3290.922967	0.512734	-3292.431139	0.057841	0.055231	-3292.488981	-3292.486370
26a-Al'_11	-3292.959800	-3290.918758	0.512772	-3292.426894	0.057425	0.055007	-3292.484319	-3292.481901
26a-Al'_12	-3292.964556	-3290.924192	0.513610	-3292.431061	0.056332	0.054391	-3292.487393	-3292.485452
26a-Al'_13	-3292.965706	-3290.925745	0.512801	-3292.432774	0.057426	0.055026	-3292.490201	-3292.487800
26a-Al'_14	-3292.966493	-3290.926057	0.512572	-3292.433665	0.057810	0.055301	-3292.491475	-3292.488966
26a-Al'_2	-3292.964525	-3290.923957	0.512851	-3292.431566	0.057628	0.055006	-3292.489194	-3292.486572
26a-Al'_3	-3292.962710	-3290.919997	0.513328	-3292.429271	0.057663	0.055089	-3292.486934	-3292.484360
26a-Al'_4	-3292.968042	-3290.929590	0.513616	-3292.434602	0.056311	0.054267	-3292.490913	-3292.488869
26a-Al'_5	-3292.958627	-3290.916142	0.513083	-3292.425486	0.057263	0.054833	-3292.482749	-3292.480319
26a-Al'_6	-3292.961460	-3290.919805	0.512665	-3292.428444	0.058386	0.055613	-3292.486831	-3292.484057

26a-Al'_7	-3292.965530	-3290.924307	0.512775	-3292.432652	0.057189	0.054894	-3292.489841	-3292.487546
26a-Al'_8	-3292.962364	-3290.921572	0.512497	-3292.429637	0.058239	0.055382	-3292.487876	-3292.485019
26a-Al'_9	-3292.966358	-3290.926714	0.512159	-3292.433770	0.058063	0.055659	-3292.491832	-3292.489428
26a-Al_1	-3292.974693	-3290.936701	0.512309	-3292.442039	0.058159	0.055527	-3292.500197	-3292.497565
26a-Al_10	-3292.967473	-3290.930557	0.512828	-3292.434492	0.057889	0.055249	-3292.492381	-3292.489741
26a-Al_2	-3292.970112	-3290.932540	0.512084	-3292.437545	0.058926	0.056017	-3292.496471	-3292.493562
26a-Al_3	-3292.967608	-3290.929734	0.513214	-3292.434287	0.057367	0.054934	-3292.491655	-3292.489222
26a-Al_4	-3292.967567	-3290.929630	0.513134	-3292.434249	0.057843	0.055178	-3292.492092	-3292.489427
26a-Al_5	-3292.969387	-3290.931604	0.511465	-3292.437289	0.059568	0.056421	-3292.496857	-3292.493710
26a-Al_6	-3292.973826	-3290.935276	0.512905	-3292.440643	0.058323	0.055470	-3292.498966	-3292.496113
26a-Al_7	-3292.970990	-3290.933746	0.512362	-3292.438309	0.058250	0.055583	-3292.496559	-3292.493892
26a-Al_8	-3292.968483	-3290.931290	0.512932	-3292.435510	0.056943	0.054787	-3292.492452	-3292.490297
26a-Al_9	-3292.972819	-3290.934993	0.512336	-3292.440220	0.058009	0.055369	-3292.498230	-3292.495589
26a-B_1	-1994.345046	-1992.392548	0.523127	-1993.804192	0.051356	0.049873	-1993.855548	-1993.854065
26a-B_10	-1994.337068	-1992.387046	0.520316	-1993.798540	0.052357	0.050833	-1993.850897	-1993.849373
26a-B_11	-1994.335369	-1992.385445	0.519904	-1993.796949	0.053533	0.051589	-1993.850482	-1993.848538
26a-B_12	-1994.335561	-1992.385390	0.519847	-1993.797202	0.053678	0.051597	-1993.850880	-1993.848799
26a-B_13	-1994.338562	-1992.386978	0.520832	-1993.799435	0.053278	0.051165	-1993.852713	-1993.850601
26a-B_14	-1994.335486	-1992.385747	0.520212	-1993.796883	0.053025	0.051278	-1993.849908	-1993.848161
26a-B_15	-1994.336952	-1992.386049	0.520255	-1993.798383	0.052736	0.051099	-1993.851119	-1993.849483
26a-B_16	-1994.337869	-1992.385951	0.520569	-1993.798961	0.053126	0.051166	-1993.852087	-1993.850127
26a-B_17	-1994.335763	-1992.385026	0.520836	-1993.796592	0.052966	0.051194	-1993.849558	-1993.847786

26a-B_18	-1994.335773	-1992.385261	0.520972	-1993.796722	0.052265	0.050552	-1993.848987	-1993.847273
26a-B_19	-1994.336252	-1992.385095	0.520533	-1993.797451	0.052575	0.050943	-1993.850026	-1993.848395
26a-B_2	-1994.342199	-1992.392354	0.519917	-1993.803663	0.053751	0.051801	-1993.857414	-1993.855465
26a-B_3	-1994.342200	-1992.392354	0.520190	-1993.803549	0.053172	0.051423	-1993.856721	-1993.854972
26a-B_4	-1994.345189	-1992.392870	0.520973	-1993.805925	0.052879	0.051036	-1993.858804	-1993.856961
26a-B_5	-1994.341029	-1992.391287	0.519999	-1993.802532	0.053511	0.051589	-1993.856043	-1993.854121
26a-B_6	-1994.344248	-1992.391541	0.520084	-1993.805532	0.053832	0.051790	-1993.859363	-1993.857322
26a-B_7	-1994.342409	-1992.392550	0.520225	-1993.803761	0.053321	0.051419	-1993.857082	-1993.855180
26a-B_8	-1994.344567	-1992.392755	0.519953	-1993.805939	0.053828	0.051925	-1993.859766	-1993.857864
26a-B_9	-1994.344116	-1992.392028	0.520210	-1993.805483	0.053189	0.051332	-1993.858672	-1993.856814
26b-Al'_1	-3292.966407	-3290.928760	0.512625	-3292.433593	0.057457	0.055096	-3292.491049	-3292.488689
26b-Al'_2	-3292.966414	-3290.928758	0.512563	-3292.433614	0.058046	0.055371	-3292.491660	-3292.488984
26b-Al'_3	-3292.961807	-3290.921270	0.512333	-3292.429199	0.057810	0.055309	-3292.487009	-3292.484508
26b-Al'_4	-3292.962544	-3290.921890	0.512377	-3292.429958	0.057538	0.055150	-3292.487496	-3292.485109
26b-Al'_5	-3292.962962	-3290.922142	0.512452	-3292.430258	0.057854	0.055282	-3292.488112	-3292.485540
26b-Al_1	-3292.974244	-3290.937737	0.512258	-3292.441682	0.058379	0.055529	-3292.500062	-3292.497211
26b-Al_10	-3292.960555	-3290.923340	0.512194	-3292.427968	0.059308	0.056053	-3292.487276	-3292.484021
26b-Al_11	-3292.969905	-3290.931363	0.513564	-3292.436449	0.056646	0.054483	-3292.493095	-3292.490932
26b-Al_12	-3292.968558	-3290.930103	0.512962	-3292.435550	0.057358	0.054846	-3292.492908	-3292.490396
26b-Al_13	-3292.966537	-3290.928384	0.512292	-3292.433890	0.058190	0.055610	-3292.492080	-3292.489500
26b-Al_14	-3292.969838	-3290.932680	0.513167	-3292.436628	0.056915	0.054732	-3292.493543	-3292.491360
26b-Al_15	-3292.965511	-3290.926368	0.512892	-3292.432401	0.057684	0.055287	-3292.490084	-3292.487687

26b-Al_16	-3292.968685	-3290.931833	0.513151	-3292.435306	0.057663	0.055244	-3292.492969	-3292.490550
26b-Al_17	-3292.969281	-3290.930866	0.513072	-3292.435979	0.057570	0.055247	-3292.493549	-3292.491226
26b-Al_2	-3292.965632	-3290.927319	0.513008	-3292.432427	0.058035	0.055348	-3292.490462	-3292.487775
26b-Al_3	-3292.954322	-3290.916015	0.513656	-3292.420912	0.056791	0.054326	-3292.477702	-3292.475237
26b-Al_4	-3292.967003	-3290.931148	0.511922	-3292.434663	0.058476	0.055756	-3292.493138	-3292.490419
26b-Al_5	-3292.968780	-3290.930768	0.512012	-3292.436361	0.058623	0.055807	-3292.494984	-3292.492168
26b-Al_6	-3292.968527	-3290.929762	0.512617	-3292.435617	0.058679	0.055604	-3292.494296	-3292.491221
26b-Al_7	-3292.969181	-3290.931326	0.512658	-3292.436262	0.058118	0.055439	-3292.494380	-3292.491701
26b-Al_8	-3292.960583	-3290.923338	0.512191	-3292.428039	0.058668	0.055746	-3292.486708	-3292.483785
26b-Al_9	-3292.968464	-3290.929771	0.512746	-3292.435477	0.058200	0.055377	-3292.493677	-3292.490853
26b-Al_18	-3292.962179	-3290.921716	0.512365	-3292.429511	0.058290	0.055534	-3292.487801	-3292.485045
26b-B_1	-1994.345375	-1992.394775	0.519522	-1993.807124	0.054458	0.052128	-1993.861582	-1993.859253
26b-B_10	-1994.328054	-1992.376879	0.521539	-1993.788528	0.052078	0.050371	-1993.840606	-1993.838899
26b-B_11	-1994.339815	-1992.387777	0.520707	-1993.800726	0.052930	0.051225	-1993.853656	-1993.851951
26b-B_12	-1994.328588	-1992.378323	0.521417	-1993.789384	0.051455	0.049902	-1993.840839	-1993.839286
26b-B_13	-1994.337091	-1992.385650	0.520911	-1993.797964	0.052515	0.050878	-1993.850479	-1993.848843
26b-B_14	-1994.338974	-1992.387794	0.521044	-1993.799764	0.052330	0.050742	-1993.852094	-1993.850506
26b-B_15	-1994.337192	-1992.385011	0.520713	-1993.798217	0.052867	0.051035	-1993.851084	-1993.849252
26b-B_16	-1994.338614	-1992.387446	0.520764	-1993.799421	0.053208	0.051356	-1993.852629	-1993.850777
26b-B_17	-1994.338549	-1992.386569	0.520174	-1993.799710	0.054684	0.052226	-1993.854394	-1993.851935
26b-B_18	-1994.335387	-1992.384753	0.520933	-1993.796187	0.053019	0.051122	-1993.849206	-1993.847310
26b-B_19	-1994.335247	-1992.382981	0.520272	-1993.796517	0.053927	0.051537	-1993.850443	-1993.848054

26b-B_2	-1994.344703	-1992.393598	0.520302	-1993.805950	0.053397	0.051382	-1993.859347	-1993.857332
26b-B_20	-1994.335334	-1992.383911	0.521265	-1993.795916	0.052439	0.050759	-1993.848355	-1993.846675
26b-B_21	-1994.334919	-1992.381647	0.520066	-1993.796538	0.052763	0.051026	-1993.849300	-1993.847564
26b-B_3	-1994.339100	-1992.388746	0.519945	-1993.800651	0.053458	0.051571	-1993.854109	-1993.852223
26b-B_4	-1994.337995	-1992.389660	0.520700	-1993.799020	0.052677	0.050992	-1993.851698	-1993.850012
26b-B_5	-1994.339052	-1992.388050	0.520378	-1993.800294	0.053108	0.051273	-1993.853402	-1993.851567
26b-B_6	-1994.338448	-1992.386892	0.521075	-1993.799178	0.052817	0.050874	-1993.851995	-1993.850052
26b-B_7	-1994.339902	-1992.388042	0.521383	-1993.800369	0.052394	0.050749	-1993.852762	-1993.851118
26b-B_8	-1994.333872	-1992.383796	0.520510	-1993.795008	0.053208	0.051211	-1993.848216	-1993.846218
26b-B_9	-1994.338510	-1992.386902	0.520813	-1993.799358	0.053293	0.051244	-1993.852650	-1993.850602
27a-Al_1	-3183.499813	-3181.610108	0.504682	-3182.976256	0.054923	0.052765	-3183.031179	-3183.029021
27a-Al_2	-3183.496261	-3181.605441	0.505056	-3182.972446	0.054959	0.052517	-3183.027405	-3183.024963
27a-Al_3	-3183.497467	-3181.607943	0.503968	-3182.974428	0.055966	0.053239	-3183.030393	-3183.027667
27a-Al_4	-3183.499724	-3181.608665	0.503664	-3182.977078	0.055468	0.053020	-3183.032546	-3183.030098
27a-Al_5	-3183.499458	-3181.608115	0.503326	-3182.976902	0.056588	0.053764	-3183.033491	-3183.030666
27a-Al_6	-3183.496557	-3181.605605	0.504270	-3182.973203	0.056003	0.053314	-3183.029206	-3183.026517
27a-Al_7	-3183.499275	-3181.608927	0.504161	-3182.976248	0.055391	0.052823	-3183.031639	-3183.029071
27a-Al_8	-3183.497152	-3181.605862	0.504426	-3182.973814	0.055386	0.052928	-3183.029200	-3183.026743
27b-Al_1	-3183.503814	-3181.614872	0.503301	-3182.981289	0.056136	0.053558	-3183.037425	-3183.034847
27b-Al_2	-3183.500851	-3181.612076	0.504597	-3182.977164	0.056033	0.053394	-3183.033196	-3183.030558
27b-Al_3	-3183.500997	-3181.611221	0.504112	-3182.977589	0.056967	0.053983	-3183.034556	-3183.031572
27b-Al_4	-3183.498649	-3181.610879	0.504449	-3182.975040	0.056029	0.053490	-3183.031070	-3183.028530

27b-Al_5	-3183.499360	-3181.609964	0.504195	-3182.976130	0.056083	0.053402	-3183.032212	-3183.029531	
27b-Al_6	-3183.500380	-3181.612889	0.503633	-3182.977464	0.056284	0.053765	-3183.033749	-3183.031229	
27b-Al_7	-3183.498764	-3181.611496	0.504221	-3182.975396	0.055791	0.053403	-3183.031187	-3183.028798	
28a-Al_1	-3183.492119	-3181.605992	0.504260	-3182.968747	0.055768	0.053359	-3183.024515	-3183.022107	
28a-Al_2	-3183.492467	-3181.606070	0.504241	-3182.969215	0.055201	0.053061	-3183.024415	-3183.022275	
28b-Al_1	-3183.499925	-3181.611440	0.504211	-3182.976602	0.055595	0.053233	-3183.032197	-3183.029835	
28b-Al_2	-3183.498453	-3181.608431	0.503546	-3182.975513	0.056763	0.054067	-3183.032277	-3183.029580	
28b-Al_3	-3183.497258	-3181.606568	0.502972	-3182.974835	0.057301	0.054225	-3183.032135	-3183.029059	
28b-Al_4	-3183.496321	-3181.606965	0.503926	-3182.973089	0.056907	0.053975	-3183.029996	-3183.027064	
28b-Al_5	-3183.494943	-3181.604614	0.503662	-3182.971915	0.057366	0.054156	-3183.029280	-3183.026070	
28b-Al_6	-3183.496434	-3181.606416	0.504204	-3182.973044	0.055994	0.053457	-3183.029038	-3183.026500	
28b-Al_7	-3183.497721	-3181.609672	0.504183	-3182.974384	0.057048	0.053815	-3183.031433	-3183.028200	
28b-Al_8	-3183.496178	-3181.607032	0.503394	-3182.973509	0.056569	0.053753	-3183.030078	-3183.027263	
28b-Al_9	-3183.495685	-3181.606226	0.503701	-3182.972687	0.056696	0.053807	-3183.029384	-3183.026494	
29a-Al_1	-3183.447389	-3181.557792	0.502387	-3182.925502	0.057641	0.054412	-3182.983143	-3182.979913	
29a-Al_2	-3183.447430	-3181.558374	0.502527	-3182.925556	0.056741	0.053909	-3182.982296	-3182.979465	
29b-Al_1	-3183.447203	-3181.556564	0.503074	-3182.924993	0.056094	0.053405	-3182.981088	-3182.978399	
29b-Al_2	-3183.445997	-3181.555698	0.503019	-3182.923718	0.056619	0.053735	-3182.980337	-3182.977453	
CH2N2	-148.746520	-148.571736	0.032038	-148.711527	0.016832	0.016833	-148.728358	-148.728360	
N2	-109.546736	-109.394137	0.005779	-109.538594	0.012817	0.012817	-109.551411	-109.551411	
TMSCHN2	-557.402630	-557.051931	0.135019	-557.260856	0.027765	0.027349	-557.288622	-557.288205	
TS1'_1	-1585.670202	-1583.894492	0.415859	-1585.239721	0.045781	0.044434	-1585.285502	-1585.284156	-319.66

TS1'_2	-1585.666131	-1583.888693	0.416331	-1585.235375	0.045391	0.044027	-1585.280766	-1585.279401	-322.53
TS1'_3	-1585.666065	-1583.888848	0.415906	-1585.235591	0.045828	0.044359	-1585.281419	-1585.279950	-324.27
TS1_A_1	-1585.668933	-1583.894956	0.415709	-1585.238618	0.045853	0.044428	-1585.284470	-1585.283046	-311.88
TS1_A_2	-1585.669004	-1583.894598	0.415761	-1585.238702	0.045543	0.044267	-1585.284245	-1585.282969	-289.35
TS1_A_3	-1585.661455	-1583.889297	0.416339	-1585.230795	0.045044	0.043734	-1585.275839	-1585.274529	-319.05
TS1_A_4	-1585.661024	-1583.888536	0.415905	-1585.230546	0.045810	0.044357	-1585.276356	-1585.274903	-304.44
TS1_B_1	-1585.669843	-1583.895652	0.415855	-1585.239374	0.045812	0.044408	-1585.285186	-1585.283782	-306.28
TS1_B_2	-1585.668633	-1583.894600	0.415982	-1585.238079	0.045743	0.044318	-1585.283822	-1585.282397	-315.69
TS1_B_3	-1585.660242	-1583.888194	0.415318	-1585.230254	0.046066	0.044552	-1585.276320	-1585.274805	-302.40
TS1_B_4	-1585.658368	-1583.886316	0.415788	-1585.228083	0.045458	0.044134	-1585.273541	-1585.272218	-313.91
TS1_B_5	-1585.659452	-1583.887223	0.415488	-1585.229322	0.045916	0.044467	-1585.275239	-1585.273789	-290.84
TS1_B_6	-1585.660342	-1583.888534	0.415484	-1585.230260	0.045797	0.044385	-1585.276057	-1585.274645	-289.28
TS1a-B'_1	-1994.318456	-1992.371519	0.518770	-1993.781284	0.053165	0.051366	-1993.834449	-1993.832650	-307.01
TS1a-B'_2	-1994.323100	-1992.374019	0.518948	-1993.785677	0.053342	0.051557	-1993.839018	-1993.837234	-266.84
TS1a-B'_3	-1994.314781	-1992.367267	0.519728	-1993.776921	0.052159	0.050700	-1993.829081	-1993.827621	-302.89
TS1a-B'_4	-1994.317101	-1992.369125	0.518690	-1993.780001	0.053122	0.051327	-1993.833124	-1993.831328	-288.27
TS1a-B'_5	-1994.308920	-1992.358750	0.518141	-1993.772120	0.053817	0.051913	-1993.825937	-1993.824032	-329.66
TS1a-B'_6	-1994.319759	-1992.368583	0.518901	-1993.782426	0.053152	0.051401	-1993.835578	-1993.833827	-272.20
TS1a-B'_7	-1994.319835	-1992.368526	0.518678	-1993.782583	0.054143	0.051873	-1993.836726	-1993.834456	-271.51
TS1a-B'_8	-1994.319607	-1992.367760	0.519629	-1993.781847	0.052313	0.050656	-1993.834160	-1993.832503	-304.74
TS1a-Al'_1	-3292.940122	-3290.903326	0.510020	-3292.409595	0.058615	0.055937	-3292.468210	-3292.465532	-296.77
TS1a-Al'_2	-3292.940980	-3290.904098	0.510504	-3292.410015	0.058890	0.056007	-3292.468906	-3292.466022	-296.36

TS1a-Al'_3	-3292.939832	-3290.903882	0.510311	-3292.409074	0.058729	0.055950	-3292.467803	-3292.465024	-305.34
TS1a-Al'_4	-3292.948156	-3290.915551	0.511636	-3292.416596	0.056655	0.054629	-3292.473251	-3292.471225	-291.29
TS1a-Al-6	-3292.947878	-3290.915781	0.510035	-3292.417156	0.060374	0.056879	-3292.477530	-3292.474035	-280.20
TS1a-Al-7	-3292.950183	-3290.917791	0.510160	-3292.419403	0.059637	0.056454	-3292.479040	-3292.475857	-252.48
TS1a-Al-8	-3292.948640	-3290.916394	0.511263	-3292.417353	0.057232	0.054811	-3292.474586	-3292.472165	-302.62
TS1a-Al-9	-3292.947726	-3290.915097	0.510616	-3292.416747	0.058385	0.055713	-3292.475131	-3292.472459	-296.32
TS1a-Al_1	-3292.950154	-3290.916555	0.510338	-3292.419250	0.059547	0.056312	-3292.478797	-3292.475562	-242.64
TS1a-Al_2	-3292.950156	-3290.918063	0.510082	-3292.419447	0.059524	0.056395	-3292.478971	-3292.475842	-235.26
TS1a-Al_3	-3292.950778	-3290.919298	0.510021	-3292.420144	0.059513	0.056379	-3292.479657	-3292.476523	-231.12
TS1a-Al_4	-3292.949696	-3290.917648	0.510406	-3292.418858	0.058739	0.055850	-3292.477596	-3292.474708	-295.29
TS1a-Al_5	-3292.947184	-3290.913042	0.510635	-3292.416162	0.058904	0.055892	-3292.475065	-3292.472054	-290.97
TS1a-B_1	-1994.321248	-1992.372486	0.518631	-1993.784159	0.053613	0.051558	-1993.837772	-1993.835718	-265.46
TS1a-B_2	-1994.321968	-1992.375158	0.518219	-1993.785010	0.054240	0.052134	-1993.839250	-1993.837145	-302.34
TS1a-B_3	-1994.322889	-1992.375681	0.517939	-1993.786096	0.054576	0.052402	-1993.840672	-1993.838499	-296.77
TS1a-B_4	-1994.322277	-1992.376885	0.519253	-1993.784785	0.052696	0.050974	-1993.837481	-1993.835759	-301.52
TS1a-B_5	-1994.319766	-1992.371641	0.518349	-1993.782907	0.053586	0.051620	-1993.836493	-1993.834527	-294.05
TS1a-B_6	-1994.322666	-1992.376392	0.518593	-1993.785491	0.053731	0.051781	-1993.839222	-1993.837272	-281.86
TS1a-B_7	-1994.322474	-1992.376114	0.518383	-1993.785381	0.054659	0.052277	-1993.840040	-1993.837657	-279.46
TS1a-B_8	-1994.321459	-1992.375453	0.518576	-1993.784403	0.053594	0.051576	-1993.837997	-1993.835979	-310.75
TS1a-B_9	-1994.319913	-1992.374356	0.518564	-1993.782908	0.053030	0.051369	-1993.835938	-1993.834277	-296.99
TS1b-B'_1	-1994.322413	-1992.376669	0.519202	-1993.784924	0.052555	0.051041	-1993.837480	-1993.835965	-290.75
TS1b-B'_2	-1994.315260	-1992.365178	0.518309	-1993.778161	0.054361	0.052231	-1993.832521	-1993.830391	-297.41

TS1b-B'_3	-1994.314480	-1992.364231	0.518445	-1993.777407	0.053743	0.051826	-1993.831151	-1993.829234	-300.89
TS1b-B'_4	-1994.314444	-1992.365247	0.517541	-1993.777944	0.054856	0.052637	-1993.832800	-1993.830581	-299.86
TS1b-Al'_1	-3292.948225	-3290.914735	0.510965	-3292.417109	0.057432	0.055086	-3292.474541	-3292.472195	-299.36
TS1b-Al'_2	-3292.950321	-3290.915455	0.511483	-3292.418811	0.057075	0.054844	-3292.475886	-3292.473655	-284.31
TS1b-Al'_3	-3292.942921	-3290.908135	0.510388	-3292.412179	0.058331	0.055650	-3292.470510	-3292.467829	-306.19
TS1b-Al'_4	-3292.945681	-3290.910722	0.511156	-3292.414507	0.057008	0.054789	-3292.471515	-3292.469296	-286.95
TS1b-Al'_5	-3292.938666	-3290.901970	0.509853	-3292.408119	0.060235	0.056730	-3292.468354	-3292.464849	-282.92
TS1b-Al'_6	-3292.944881	-3290.907474	0.511031	-3292.413603	0.057818	0.055370	-3292.471421	-3292.468972	-268.67
TS1b-Al'_7	-3292.943902	-3290.906434	0.511125	-3292.412723	0.057443	0.054993	-3292.470166	-3292.467716	-295.97
TS1b-Al'_8	-3292.945479	-3290.906893	0.511499	-3292.414024	0.056864	0.054631	-3292.470888	-3292.468655	-299.71
TS1b-Al-1	-3292.950900	-3290.919162	0.510137	-3292.420171	0.058875	0.056162	-3292.479046	-3292.476332	-232.54
TS1b-Al-2	-3292.951138	-3290.919779	0.510470	-3292.420272	0.058540	0.055782	-3292.478812	-3292.476054	-252.29
TS1b-Al-3	-3292.948343	-3290.915016	0.510178	-3292.417561	0.059585	0.056393	-3292.477146	-3292.473954	-276.80
TS1b-Al-4	-3292.948591	-3290.913796	0.510064	-3292.417930	0.059884	0.056496	-3292.477814	-3292.474426	-288.44
TS1b-Al-5	-3292.950237	-3290.916840	0.510335	-3292.419469	0.058754	0.055895	-3292.478224	-3292.475364	-292.82
TS1b-B_1	-1994.323291	-1992.375951	0.518106	-1993.786468	0.054304	0.052108	-1993.840772	-1993.838577	-295.52
TS1b-B_2	-1994.319032	-1992.373987	0.518353	-1993.781906	0.054767	0.052398	-1993.836673	-1993.834304	-297.09
TS1b-B_3	-1994.321451	-1992.374367	0.517987	-1993.784691	0.054421	0.052213	-1993.839112	-1993.836904	-282.43
TS1b-B_4	-1994.322001	-1992.374123	0.518652	-1993.784823	0.053766	0.051655	-1993.838589	-1993.836477	-293.76
TS1b-B_5	-1994.325271	-1992.377879	0.519019	-1993.787964	0.052805	0.051104	-1993.840769	-1993.839069	-287.12
TS1b-B_6	-1994.319649	-1992.374266	0.517778	-1993.783029	0.055229	0.052626	-1993.838259	-1993.835656	-278.83
TS1b-B_7	-1994.319706	-1992.373530	0.517335	-1993.783418	0.055066	0.052707	-1993.838484	-1993.836125	-278.09

TS2'-A_1	-1585.671843	-1583.885118	0.416141	-1585.241079	0.045922	0.044409	-1585.287001	-1585.285488	-453.55
TS2'-A_2	-1585.675247	-1583.888311	0.415592	-1585.244861	0.046556	0.044808	-1585.291417	-1585.289669	-431.46
TS2'-A_3	-1585.670495	-1583.880849	0.415536	-1585.240110	0.047145	0.045076	-1585.287255	-1585.285186	-433.33
TS2'-B_1	-1585.667789	-1583.880558	0.415079	-1585.237745	0.046882	0.045144	-1585.284626	-1585.282888	-343.85
TS2'-B_2	-1585.664296	-1583.873840	0.415070	-1585.234245	0.047147	0.045235	-1585.281392	-1585.279481	-350.60
TS2'-B_3	-1585.671179	-1583.882707	0.414796	-1585.241328	0.047090	0.045345	-1585.288418	-1585.286673	-379.42
TS2'-C_1	-1585.677204	-1583.891433	0.416871	-1585.245832	0.045177	0.044042	-1585.291008	-1585.289873	-506.73
TS2'-C_2	-1585.675608	-1583.887059	0.416641	-1585.244422	0.045511	0.044188	-1585.289934	-1585.288610	-513.17
TS2'-C_3	-1585.676755	-1583.888429	0.416029	-1585.245977	0.046725	0.044818	-1585.292702	-1585.290795	-513.59
TS2-A_1	-1585.685393	-1583.899753	0.416955	-1585.253908	0.045574	0.044140	-1585.299483	-1585.298049	-455.77
TS2-A_2	-1585.684463	-1583.898747	0.415698	-1585.253923	0.046517	0.044922	-1585.300440	-1585.298845	-467.87
TS2-A_4	-1585.685590	-1583.900325	0.415963	-1585.254797	0.046845	0.044913	-1585.301642	-1585.299710	-460.40
TS2-A_5	-1585.684646	-1583.899317	0.415905	-1585.254051	0.045983	0.044559	-1585.300033	-1585.298610	-467.82
TS2-B_1	-1585.685123	-1583.899207	0.415517	-1585.254620	0.047262	0.045233	-1585.301882	-1585.299852	-462.52
TS2-B_2	-1585.680915	-1583.896522	0.416110	-1585.250117	0.046043	0.044555	-1585.296160	-1585.294672	-445.90
TS2-B_3	-1585.683595	-1583.897974	0.415974	-1585.252865	0.046174	0.044721	-1585.299039	-1585.297585	-441.39
TS2-B_4	-1585.685264	-1583.899639	0.415741	-1585.254642	0.046728	0.044950	-1585.301370	-1585.299592	-461.95
TS2-B_5	-1585.681130	-1583.896812	0.416202	-1585.250246	0.046131	0.044574	-1585.296377	-1585.294820	-442.66
TS2-B_6	-1585.683518	-1583.898016	0.416023	-1585.252816	0.045945	0.044537	-1585.298761	-1585.297353	-433.03
TS2-C_1	-1585.680530	-1583.893992	0.416650	-1585.249234	0.046173	0.044554	-1585.295406	-1585.293788	-518.35
TS2-C_2	-1585.680388	-1583.893330	0.416395	-1585.249279	0.046289	0.044690	-1585.295568	-1585.293969	-513.53
TS2a-Al''_1	-3292.960515	-3290.915014	0.509822	-3292.429873	0.059877	0.056713	-3292.489750	-3292.486586	-443.87

TS2a-Al''_2	-3292.960323	-3290.914829	0.510084	-3292.429680	0.058594	0.055907	-3292.488275	-3292.485587	-440.62
TS2a-Al''_3	-3292.954542	-3290.908458	0.509764	-3292.424035	0.059311	0.056436	-3292.483346	-3292.480471	-421.38
TS2a-Al''_4	-3292.960574	-3290.914918	0.510136	-3292.429878	0.058797	0.055938	-3292.488675	-3292.485816	-460.06
TS2a-Al'_1	-3292.964399	-3290.918518	0.509700	-3292.433889	0.059959	0.056622	-3292.493848	-3292.490511	-371.46
TS2a-Al'_2	-3292.963968	-3290.917496	0.509370	-3292.433623	0.060368	0.057014	-3292.493991	-3292.490637	-383.56
TS2a-Al'_3	-3292.959428	-3290.913696	0.509768	-3292.428889	0.059947	0.056723	-3292.488836	-3292.485612	-367.22
TS2a-Al_1	-3292.957619	-3290.912363	0.510526	-3292.426434	0.059518	0.056358	-3292.485952	-3292.482792	-334.80
TS2a-B''_1	-1994.330656	-1992.370425	0.517192	-1993.794328	0.055295	0.052910	-1993.849623	-1993.847238	-415.76
TS2a-B''_2	-1994.330727	-1992.370595	0.517820	-1993.794039	0.054381	0.052246	-1993.848420	-1993.846285	-417.03
TS2a-B''_3	-1994.321586	-1992.362243	0.517558	-1993.785102	0.054217	0.052332	-1993.839319	-1993.837435	-453.52
TS2a-B'_1	-1994.335408	-1992.375430	0.517281	-1993.799054	0.055039	0.052700	-1993.854093	-1993.851754	-387.74
TS2a-B'_2	-1994.331963	-1992.373283	0.517410	-1993.795672	0.054274	0.052294	-1993.849946	-1993.847966	-352.69
TS2a-B'_3	-1994.334737	-1992.374368	0.517496	-1993.798232	0.055072	0.052586	-1993.853304	-1993.850818	-398.67
TS2a-B'_4	-1994.332469	-1992.373650	0.517675	-1993.796013	0.054089	0.052069	-1993.850102	-1993.848082	-337.79
TS2a-B_1	-1994.327347	-1992.368199	0.517679	-1993.790756	0.054552	0.052379	-1993.845307	-1993.843135	-335.31
TS2a-B_2	-1994.325647	-1992.367001	0.518257	-1993.788794	0.053310	0.051639	-1993.842104	-1993.840433	-344.64
TS2b-Al''_1	-3292.958221	-3290.912090	0.510043	-3292.427538	0.058988	0.056114	-3292.486526	-3292.483652	-456.66
TS2b-Al''_2	-3292.957578	-3290.910800	0.510431	-3292.426612	0.059117	0.056084	-3292.485729	-3292.482695	-456.45
TS2b-Al'_1	-3292.965671	-3290.921124	0.510317	-3292.434773	0.058947	0.055980	-3292.493720	-3292.490753	-415.81
TS2b-Al'_2	-3292.965840	-3290.920869	0.510350	-3292.434975	0.058802	0.055869	-3292.493777	-3292.490843	-411.45
TS2b-Al'_3	-3292.958674	-3290.913349	0.509534	-3292.428344	0.059158	0.056447	-3292.487502	-3292.484791	-429.63
TS2b-Al'_4	-3292.958263	-3290.913638	0.509802	-3292.427782	0.058789	0.056144	-3292.486572	-3292.483926	-417.22

TS2b-Al_1	-3292.957253	-3290.912316	0.510072	-3292.426498	0.058755	0.056162	-3292.485252	-3292.482660	-333.71
TS2b-Al_2	-3292.956114	-3290.911513	0.509958	-3292.425362	0.059038	0.056342	-3292.484400	-3292.481704	-334.78
TS2b-Al_3	-3292.954327	-3290.908630	0.509322	-3292.424131	0.059922	0.056797	-3292.484054	-3292.480928	-327.76
TS2b-Al_4	-3292.957151	-3290.912293	0.510006	-3292.426364	0.059217	0.056443	-3292.485581	-3292.482807	-335.24
TS2b-Al_5	-3292.955995	-3290.911437	0.509782	-3292.425346	0.059321	0.056519	-3292.484667	-3292.481865	-337.08
TS2b-Al_6	-3292.953145	-3290.907543	0.509503	-3292.422746	0.059726	0.056779	-3292.482472	-3292.479524	-331.62
TS2b-B''_1	-1994.328272	-1992.367994	0.517700	-1993.791612	0.054660	0.052514	-1993.846272	-1993.844126	-467.41
TS2b-B''_2	-1994.327474	-1992.366258	0.517795	-1993.790739	0.054873	0.052527	-1993.845611	-1993.843266	-465.49
TS2b-B''_3	-1994.327083	-1992.365805	0.517625	-1993.790371	0.055796	0.052981	-1993.846168	-1993.843352	-455.71
TS2b-B'_1	-1994.336679	-1992.378183	0.517911	-1993.799889	0.054616	0.052304	-1993.854505	-1993.852193	-433.28
TS2b-B'_2	-1994.336344	-1992.377606	0.517900	-1993.799559	0.055135	0.052481	-1993.854694	-1993.852040	-423.82
TS2b-B'_3	-1994.330551	-1992.371541	0.517818	-1993.793985	0.053725	0.051940	-1993.847710	-1993.845925	-433.83
TS2b-B'_4	-1994.329086	-1992.371687	0.517981	-1993.792308	0.054105	0.052091	-1993.846412	-1993.844399	-426.34
TS2b-B_1	-1994.328229	-1992.369044	0.517794	-1993.791595	0.054251	0.052213	-1993.845846	-1993.843808	-334.33
TS2b-B_2	-1994.327300	-1992.368631	0.517413	-1993.790777	0.055309	0.052894	-1993.846085	-1993.843670	-340.83
TS2b-B_3	-1994.323979	-1992.364386	0.517274	-1993.787755	0.054467	0.052490	-1993.842222	-1993.840245	-310.26
TS2b-B_4	-1994.327473	-1992.369134	0.517530	-1993.791093	0.054300	0.052189	-1993.845394	-1993.843282	-333.81
TS2b-B_5	-1994.323988	-1992.364197	0.518065	-1993.787210	0.053743	0.051926	-1993.840952	-1993.839136	-332.11
TS2b-B_6	-1994.326975	-1992.368863	0.517860	-1993.790215	0.054546	0.052322	-1993.844761	-1993.842537	-336.82
TS2b-B_7	-1994.322076	-1992.363603	0.517548	-1993.785677	0.054280	0.052273	-1993.839957	-1993.837950	-327.54
TS2b-B_8	-1994.321706	-1992.362757	0.517571	-1993.785234	0.054443	0.052366	-1993.839677	-1993.837600	-338.30

Supplementary Table 24: Compiled thermochemical data for structures computed at the ωB97x-D/def2-TZVPP(SMD=CH_2Cl_2)// ωB97x-D/def2-SVP(SMD=CH_2Cl_2) level of theory

Name	E_{spc}	*E*	*ZPE*	H_{SPC}	*T.S*	*T.qh-S*	*G(T)*	*qh-G(T)*	ν_{imag}
20'-A_1	-1436.920059	-1435.321616	0.380282	-1436.526732	0.043075	0.041578	-1436.569806	-1436.568310	
20'-A_2	-1436.916907	-1435.317725	0.380824	-1436.523206	0.042676	0.041288	-1436.565883	-1436.564494	
20'-A_3	-1436.916716	-1435.317169	0.380733	-1436.523068	0.042667	0.041323	-1436.565735	-1436.564391	
20'-B_4	-1436.909608	-1435.311882	0.381112	-1436.515811	0.041919	0.040725	-1436.557730	-1436.556537	
20'-B_5	-1436.908767	-1435.310564	0.380820	-1436.515175	0.042340	0.040960	-1436.557515	-1436.556135	
20'-B_6	-1436.912483	-1435.315585	0.380644	-1436.518947	0.042481	0.041145	-1436.561428	-1436.560092	
21'_A_1	-1585.667289	-1583.900637	0.419090	-1585.234093	0.044476	0.043318	-1585.278569	-1585.277411	
21'_A_2	-1585.663629	-1583.895555	0.418933	-1585.230584	0.044779	0.043446	-1585.275363	-1585.274030	
21'_A_3	-1585.675000	-1583.908367	0.418579	-1585.242029	0.045347	0.043914	-1585.287376	-1585.285943	
21'_A_4	-1585.671490	-1583.902262	0.419356	-1585.237993	0.044943	0.043444	-1585.282935	-1585.281437	
21'_B_1	-1585.672921	-1583.906410	0.418370	-1585.240100	0.045812	0.044088	-1585.285912	-1585.284189	
21'_B_2	-1585.669534	-1583.900233	0.418613	-1585.236634	0.045087	0.043677	-1585.281720	-1585.280311	
21'_B_3	-1585.672709	-1583.905287	0.418660	-1585.239609	0.046228	0.044350	-1585.285837	-1585.283959	
21'_C_1	-1585.672061	-1583.906619	0.418698	-1585.239079	0.044847	0.043615	-1585.283927	-1585.282695	
21'_C_2	-1585.668316	-1583.899810	0.418937	-1585.235248	0.044824	0.043408	-1585.280072	-1585.278656	
21'_C_3	-1585.670019	-1583.901466	0.418331	-1585.237313	0.045448	0.043941	-1585.282761	-1585.281254	
21'_C_4	-1585.671129	-1583.902703	0.418425	-1585.238392	0.045088	0.043721	-1585.283480	-1585.282113	
21-ROT_1_1	-1585.673211	-1583.906139	0.417721	-1585.241391	0.044706	0.043397	-1585.286097	-1585.284788	-53.62
21-ROT_1_2	-1585.673019	-1583.906514	0.417506	-1585.241383	0.044953	0.043515	-1585.286336	-1585.284898	-120.98
21-ROT_1_3	-1585.672057	-1583.904547	0.417467	-1585.240446	0.044831	0.043541	-1585.285277	-1585.283988	-101.27
21-ROT_1_4	-1585.671829	-1583.905121	0.417870	-1585.239934	0.044277	0.043242	-1585.284211	-1585.283176	-55.19
21_A_1	-1585.673606	-1583.867875	0.419046	-1585.240341	0.044738	0.043563	-1585.285078	-1585.283904	
21_A_2	-1585.673811	-1583.868154	0.419293	-1585.240320	0.044596	0.043506	-1585.284916	-1585.283826	
21_A_3	-1585.679100	-1583.913188	0.418965	-1585.245806	0.045375	0.043898	-1585.291181	-1585.289704	
21_A_4	-1585.678825	-1583.913506	0.418452	-1585.246091	0.044961	0.043714	-1585.291053	-1585.289805	
21_A_5	-1585.678590	-1583.911873	0.418611	-1585.245508	0.045748	0.044109	-1585.291256	-1585.289617	

21_A_6	-1585.678434	-1583.912210	0.418198	-1585.245773	0.045596	0.044056	-1585.291369	-1585.289829	
21_B_1	-1585.678495	-1583.913185	0.418860	-1585.245430	0.044672	0.043533	-1585.290101	-1585.288963	
21_B_2	-1585.671654	-1583.867467	0.419252	-1585.238252	0.044589	0.043396	-1585.282841	-1585.281648	
21_B_3	-1585.678858	-1583.913405	0.418667	-1585.245922	0.044748	0.043634	-1585.290670	-1585.289556	
21_B_4	-1585.676892	-1583.911711	0.418442	-1585.244077	0.045327	0.043917	-1585.289405	-1585.287994	
21_B_5	-1585.680167	-1583.912947	0.418612	-1585.247088	0.045767	0.044097	-1585.292855	-1585.291185	
21_B_6	-1585.680300	-1583.913678	0.418393	-1585.247460	0.045681	0.044055	-1585.293141	-1585.291515	
22	-1476.226424	-1474.587150	0.409261	-1475.803631	0.044089	0.042508	-1475.847720	-1475.846139	
23	-1476.224791	-1474.586366	0.409944	-1475.801476	0.043733	0.042221	-1475.845209	-1475.843697	
24'_1	-1476.184139	-1474.546937	0.410068	-1475.761123	0.042285	0.041094	-1475.803407	-1475.802217	
24'_2	-1476.181885	-1474.544223	0.409643	-1475.759206	0.042656	0.041273	-1475.801862	-1475.800479	
24'_3	-1476.180495	-1474.541687	0.409603	-1475.757776	0.042729	0.041433	-1475.800506	-1475.799209	
24'_4	-1476.183908	-1474.544796	0.409319	-1475.761389	0.043129	0.041690	-1475.804518	-1475.803079	
24'_5	-1476.186107	-1474.547774	0.409835	-1475.763220	0.042552	0.041330	-1475.805771	-1475.804550	
24'_6	-1476.182679	-1474.543552	0.409129	-1475.760203	0.043670	0.042044	-1475.803873	-1475.802246	
24_1	-1476.184373	-1474.545957	0.409657	-1475.761603	0.042929	0.041580	-1475.804532	-1475.803183	
24_2	-1476.184285	-1474.545316	0.409695	-1475.761426	0.043097	0.041721	-1475.804523	-1475.803146	
CH2N2	-148.744019	-148.571736	0.032038	-148.709029	0.016818	0.016819	-148.725847	-148.725848	
N2	-109.527191	-109.394137	0.005779	-109.519051	0.012807	0.012807	-109.531858	-109.531858	
TS1'_1	-1585.660579	-1583.894492	0.415859	-1585.230118	0.045730	0.044386	-1585.275848	-1585.274504	-319.66
TS1'_2	-1585.656426	-1583.888693	0.416331	-1585.225688	0.045341	0.043979	-1585.271029	-1585.269667	-322.53
TS1'_3	-1585.656011	-1583.888848	0.415906	-1585.225555	0.045778	0.044311	-1585.271333	-1585.269866	-324.27
TS1_A_1	-1585.660332	-1583.894956	0.415709	-1585.230035	0.045802	0.044380	-1585.275837	-1585.274415	-311.88
TS1_A_2	-1585.660327	-1583.894598	0.415761	-1585.230044	0.045493	0.044219	-1585.275537	-1585.274263	-289.35
TS1_A_3	-1585.653156	-1583.889297	0.416339	-1585.222514	0.044995	0.043687	-1585.267509	-1585.266201	-319.05
TS1_A_4	-1585.652864	-1583.888536	0.415905	-1585.222405	0.045759	0.044309	-1585.268164	-1585.266714	-304.44
TS1_B_1	-1585.661102	-1583.895652	0.415855	-1585.230652	0.045761	0.044360	-1585.276413	-1585.275012	-306.28
TS1_B_2	-1585.660353	-1583.894600	0.415982	-1585.229818	0.045692	0.044270	-1585.275510	-1585.274088	-315.69
TS1_B_3	-1585.652227	-1583.888194	0.415318	-1585.222258	0.046016	0.044504	-1585.268274	-1585.266762	-302.40

TS1_B_4	-1585.650797	-1583.886316	0.415788	-1585.220531	0.045408	0.044087	-1585.265939	-1585.264618	-313.91
TS1_B_5	-1585.652307	-1583.887223	0.415488	-1585.222196	0.045866	0.044419	-1585.268061	-1585.266615	-290.84
TS1_B_6	-1585.652900	-1583.888534	0.415484	-1585.222837	0.045746	0.044337	-1585.268583	-1585.267174	-289.28
TS2'-A_1	-1585.655342	-1583.885118	0.416141	-1585.224597	0.045871	0.044361	-1585.270468	-1585.268958	-453.55
TS2'-A_2	-1585.658739	-1583.888311	0.415592	-1585.228372	0.046505	0.044760	-1585.274877	-1585.273132	-431.46
TS2'-A_3	-1585.653729	-1583.880849	0.415536	-1585.223363	0.047093	0.045027	-1585.270457	-1585.268391	-433.33
TS2'-B_1	-1585.651335	-1583.880558	0.415079	-1585.221310	0.046830	0.045095	-1585.268140	-1585.266405	-343.85
TS2'-B_2	-1585.647405	-1583.873840	0.415070	-1585.217373	0.047095	0.045187	-1585.264469	-1585.262560	-350.60
TS2'-B_3	-1585.654000	-1583.882707	0.414796	-1585.224168	0.047039	0.045296	-1585.271207	-1585.269464	-379.42
TS2'-C_1	-1585.660684	-1583.891433	0.416871	-1585.229330	0.045126	0.043994	-1585.274457	-1585.273324	-506.73
TS2'-C_2	-1585.659133	-1583.887059	0.416641	-1585.227967	0.045461	0.044140	-1585.273428	-1585.272107	-513.17
TS2'-C_3	-1585.660271	-1583.888429	0.416029	-1585.229512	0.046674	0.044770	-1585.276186	-1585.274282	-513.59
TS2-A_1	-1585.669407	-1583.899753	0.416955	-1585.237941	0.045524	0.044093	-1585.283465	-1585.282034	-455.77
TS2-A_2	-1585.668581	-1583.898747	0.415698	-1585.238060	0.046466	0.044874	-1585.284526	-1585.282934	-467.87
TS2-A_4	-1585.669261	-1583.900325	0.415963	-1585.238487	0.046793	0.044865	-1585.285280	-1585.283352	-460.40
TS2-A_5	-1585.668468	-1583.899317	0.415905	-1585.237892	0.045932	0.044511	-1585.283823	-1585.282402	-467.82
TS2-B_1	-1585.668781	-1583.899207	0.415517	-1585.238297	0.047211	0.045184	-1585.285507	-1585.283481	-462.52
TS2-B_2	-1585.665341	-1583.896522	0.416110	-1585.234562	0.045992	0.044507	-1585.280554	-1585.279069	-445.90
TS2-B_3	-1585.667288	-1583.897974	0.415974	-1585.236576	0.046123	0.044672	-1585.282699	-1585.281248	-441.39
TS2-B_4	-1585.668812	-1583.899639	0.415741	-1585.238209	0.046676	0.044902	-1585.284886	-1585.283111	-461.95
TS2-B_5	-1585.665392	-1583.896812	0.416202	-1585.234528	0.046080	0.044526	-1585.280608	-1585.279053	-442.66
TS2-B_6	-1585.667079	-1583.898016	0.416023	-1585.236396	0.045894	0.044489	-1585.282290	-1585.280884	-433.03
TS2-C_1	-1585.664093	-1583.893992	0.416650	-1585.232815	0.046122	0.044506	-1585.278937	-1585.277321	-518.35
TS2-C_2	-1585.664206	-1583.893330	0.416395	-1585.233116	0.046238	0.044642	-1585.279354	-1585.277757	-513.53

NOTE: The square brackets from the nomenclature used up to now has been removed due to incompatibility with the *python* based programs.

9-9. XXY Coordinates

NOTE: The square brackets from the nomenclature used up to now has been removed due to incompatibility with the *python* based programs.

48

20'-B_4 Eopt -1435.311882

C	2.203991	0.510192	-0.534061
C	1.315095	1.599565	-0.917944
C	0.120090	1.729618	-0.318964
C	-0.212143	0.793494	0.822355
C	0.426049	-0.566527	0.645243
C	1.626237	-0.718645	0.057278
C	-0.937089	2.735264	-0.592362
C	-1.720634	0.502857	0.936181
C	-2.440896	0.623100	-0.440508
C	-2.234633	2.025447	-1.047955
C	-3.900470	0.355291	-0.110822
C	-2.930910	-1.649087	0.656820
C	-1.844076	-0.937984	1.469479
H	-1.123994	3.274597	0.350874
H	1.691244	2.312399	-1.654377
H	-3.095000	2.655658	-0.787773
H	-3.275070	-2.580395	1.119733
O	3.412786	0.649372	-0.840890
C	2.277190	-2.054509	-0.164347
H	2.992754	-2.279323	0.638418
H	1.512529	-2.839741	-0.191124
H	2.830253	-2.076478	-1.111294
C	-0.551110	-1.659621	1.024547
H	-0.147989	-2.361617	1.767991
H	-0.614185	3.470144	-1.340563
H	-2.225818	1.948656	-2.142688
O	-4.835901	1.084241	-0.299360
O	-4.088215	-0.849422	0.455351
C	-2.021968	-0.552957	-1.395186
H	-0.963563	-0.416429	-1.667241
C	-2.137950	-1.898620	-0.630674
H	-2.572098	-2.678455	-1.269160
H	-2.010146	-0.979075	2.551286
H	-2.172802	1.237648	1.617392
C	-2.845963	-0.600639	-2.680699
H	-3.906339	-0.821712	-2.477659
H	-2.799043	0.340942	-3.244262
H	-2.463866	-1.397997	-3.334997
O	-0.910720	-2.398212	-0.143600
O	0.307284	1.487249	1.944101
C	0.172027	0.876879	3.205650
H	0.732308	1.500501	3.914595
H	-0.877801	0.831641	3.542952
H	0.602551	-0.140353	3.225261
B	4.741517	-0.046431	-0.357780
F	5.004399	-1.081036	-1.224202
F	4.535807	-0.458213	0.941065
F	5.673083	0.957806	-0.444359

48

20'-B_5 Eopt -1435.310564

C	-2.216272	0.369661	0.543853
C	-1.354942	1.405399	1.110398
C	-0.145434	1.644290	0.577704
C	0.219635	0.910480	-0.692516
C	-0.405621	-0.460746	-0.766640
C	-1.609693	-0.733086	-0.233613

C	0.936660	2.500570	1.137272
C	1.725646	0.652563	-0.804725
C	2.398433	0.417535	0.581283
C	2.129109	1.598609	1.540457
C	3.868200	0.272169	0.223543
C	2.949592	-1.481774	-1.063260
C	1.854887	-0.604187	-1.680919
H	1.267253	3.216811	0.369824
H	-1.745882	1.952615	1.971094
H	3.033165	2.217855	1.612666
H	3.323856	-2.253507	-1.744419
O	-3.428514	0.434195	0.860053
C	-2.255384	-2.087595	-0.299814
H	-2.973289	-2.141920	-1.129531
H	-1.485964	-2.855391	-0.443028
H	-2.803576	-2.312353	0.623468
C	0.564492	-1.420504	-1.426751
H	0.146194	-1.895256	-2.324811
H	0.587453	3.075991	2.004466
H	1.933507	1.208218	2.547829
O	4.786462	0.940523	0.613306
O	4.086020	-0.737490	-0.638644
C	2.001270	-0.975474	1.189262
H	0.939636	-0.933511	1.478491
C	2.149886	-2.073315	0.104652
H	2.593200	-2.986125	0.522357
H	2.013809	-0.368764	-2.738290
H	2.213679	1.522116	-1.265364
C	2.825240	-1.333608	2.425501
H	3.885352	-1.499009	2.174249
H	2.780359	-0.554133	3.198353
H	2.443734	-2.265379	2.868269
O	0.934662	-2.446254	-0.508319

O	-0.278954	1.563044	-1.851698
C	0.015345	2.928591	-2.027782
H	-0.360072	3.198662	-3.023587
H	-0.494965	3.560966	-1.280529
H	1.098111	3.141985	-2.000064
B	-4.756606	-0.207286	0.300905
F	-4.967634	-1.381693	0.983347
F	-4.582979	-0.381790	-1.053884
F	-5.706745	0.740920	0.587096

48

20'-B_6 Eopt -1435.315585

C	2.136257	0.390428	-0.596763
C	1.272826	1.486653	-1.023251
C	0.102818	1.699929	-0.396799
C	-0.243051	0.843606	0.795308
C	0.368690	-0.530566	0.717234
C	1.544856	-0.768859	0.106868
C	-0.959078	2.676374	-0.753493
C	-1.746484	0.597807	0.923576
C	-2.466715	0.576706	-0.452269
C	-2.212870	1.893323	-1.220250
C	-3.928976	0.393033	-0.080169
C	-3.001519	-1.532107	0.920928
C	-1.884690	-0.760448	1.632244
H	-1.202402	3.264597	0.146074
H	1.645188	2.128875	-1.824273
H	-3.093695	2.541569	-1.122176
H	-3.368414	-2.391857	1.492080
O	3.339671	0.467664	-0.946939
C	2.165328	-2.132849	0.002832
H	2.898133	-2.293839	0.805183
H	1.384411	-2.899025	0.074610
H	2.690513	-2.259661	-0.951808

C	-0.613472	-1.554491	1.253703
H	-0.202517	-2.167941	2.067120
H	-0.630958	3.371907	-1.536446
H	-2.101555	1.674002	-2.290259
O	-4.848287	1.116830	-0.349783
O	-4.138675	-0.726784	0.634537
C	-2.094922	-0.716004	-1.262157
H	-1.037487	-0.641313	-1.560174
C	-2.232841	-1.954992	-0.337182
H	-2.694453	-2.795567	-0.870585
H	-2.026609	-0.660743	2.713256
H	-2.169164	1.416042	1.521331
C	-2.940520	-0.890243	-2.522351
H	-4.001059	-1.067280	-2.280771
H	-2.887306	-0.015042	-3.184633
H	-2.583560	-1.761049	-3.091728
O	-1.010167	-2.422677	0.191869
O	0.112627	1.492173	2.014255
C	1.459869	1.834818	2.239683
H	1.508929	2.207209	3.271533
H	2.141756	0.971436	2.155841
H	1.810168	2.632582	1.562936
B	4.658832	-0.195043	-0.397869
F	4.902187	-1.325290	-1.139671
F	4.449268	-0.452565	0.941963
F	5.607605	0.776740	-0.596227

48

20'-C_1
Eopt -1435.309013

C	1.785534	2.379040	1.100780
C	1.604721	1.152626	1.916713
C	1.034399	0.048286	1.415393
C	0.739159	-0.007850	-0.063130
C	0.481100	1.339830	-0.683991
C	0.986512	2.470193	-0.173102
C	0.478386	-1.096492	2.198827
C	-0.507481	-0.819270	-0.406961
C	-1.606875	-0.674273	0.691684
C	-1.063940	-1.061296	2.087395
C	-2.687633	-1.625221	0.199111
C	-2.539855	-0.169166	-1.652938
C	-1.013425	-0.263378	-1.746638
H	0.854120	-2.047932	1.802845
H	1.874766	1.231800	2.973097
H	-1.466319	-2.044485	2.366147
H	-3.028720	-0.063373	-2.627451
O	2.449271	3.318089	1.493066
C	0.756364	3.839260	-0.722711
H	0.097840	4.414487	-0.052242
H	1.710509	4.384087	-0.775225
H	0.294859	3.812381	-1.717241
C	-0.568128	1.215900	-1.771947
H	-0.214006	1.560494	-2.753883
H	0.777360	-1.029859	3.253021
H	-1.442724	-0.343238	2.827168
O	-3.147351	-2.572035	0.776355
O	-3.130083	-1.304361	-1.030235
C	-2.289779	0.739763	0.658925
H	-1.560642	1.479004	1.021450
C	-2.648160	1.106063	-0.805299
H	-3.633645	1.585825	-0.862111
H	-0.652289	-0.841831	-2.603832
H	-0.258478	-1.880351	-0.475394
C	-3.532171	0.811613	1.546076
H	-3.911389	1.843931	1.569619
H	-4.345086	0.172239	1.165572

H	-3.323604	0.509473	2.581708
O	-1.726845	1.972839	-1.429542
O	1.894034	-0.655866	-0.782351
C	2.964817	0.219165	-1.205947
H	2.595100	0.896645	-1.981338
H	3.369567	0.776471	-0.350875
H	3.740653	-0.423988	-1.626854
B	2.494439	-2.153563	-0.416516
F	3.322687	-1.941521	0.649688
F	1.435304	-2.969318	-0.129559
F	3.141616	-2.524944	-1.560506

48

20'-C_2
Eopt -1435.308416

C	2.077980	2.345204	0.701050
C	1.305839	1.838204	1.868083
C	0.558223	0.731081	1.787368
C	0.645431	-0.089076	0.516154
C	0.900639	0.750131	-0.711606
C	1.631503	1.869891	-0.652342
C	-0.415414	0.238956	2.812723
C	-0.636397	-0.868257	0.204686
C	-1.913087	-0.122993	0.722755
C	-1.843615	0.158199	2.235423
C	-3.038929	-1.084622	0.370765
C	-2.137805	-0.701381	-1.770447
C	-0.698437	-0.982823	-1.333543
H	-0.117288	-0.761910	3.154181
H	1.336164	2.450842	2.773001
H	-2.402169	-0.622544	2.768076
H	-2.344770	-1.024801	-2.796280
O	2.934753	3.196705	0.827920
C	1.951794	2.751357	-1.815428
H	1.384902	3.694204	-1.753625
H	3.019044	3.017395	-1.797179
H	1.716314	2.269001	-2.772208
C	0.072686	0.242600	-1.874719
H	0.674568	0.031745	-2.765930
H	-0.398686	0.893743	3.693984
H	-2.360031	1.103097	2.444495
O	-3.819684	-1.597390	1.125705
O	-3.106568	-1.338755	-0.946156
C	-2.219940	1.166605	-0.121280
H	-1.438414	1.910790	0.095066
C	-2.138576	0.826606	-1.630052
H	-2.946426	1.316209	-2.189153
H	-0.305570	-1.936516	-1.697812
H	-0.606709	-1.853313	0.691250
C	-3.580375	1.781845	0.206420
H	-3.704147	2.718550	-0.356777
H	-4.410108	1.115530	-0.080026
H	-3.693029	2.020535	1.272647
O	-0.923272	1.199597	-2.234225
O	1.805525	-1.029079	0.668505
C	1.958759	-1.678734	1.951397
H	2.147228	-0.919475	2.716988
H	1.070617	-2.279591	2.184789
H	2.835873	-2.324149	1.869556
B	2.400117	-1.982771	-0.529778
F	1.603763	-3.099515	-0.515295
F	2.322893	-1.274122	-1.690601
F	3.688272	-2.225347	-0.143502

48

20'-C_3
Eopt -1435.306586

C	1.971118	1.724992	1.253596

C	1.533749	0.479748	1.931538
C	0.847724	-0.469887	1.287442
C	0.570885	-0.302811	-0.187326
C	0.541809	1.134445	-0.639817
C	1.259834	2.084035	-0.024363
C	0.216967	-1.686736	1.876624
C	-0.805751	-0.865700	-0.589300
C	-1.829174	-0.775764	0.589268
C	-1.316189	-1.533360	1.831469
C	-3.088331	-1.413134	0.018370
C	-2.779349	0.335170	-1.523994
C	-1.306771	-0.003022	-1.768095
H	0.524968	-2.561890	1.284895
H	1.796086	0.382714	2.987722
H	-1.784394	-2.526058	1.862177
H	-3.282969	0.713899	-2.419962
O	2.758824	2.490095	1.771803
C	1.269636	3.529468	-0.401095
H	0.677318	4.117553	0.318173
H	2.298869	3.915151	-0.358551
H	0.858884	3.697656	-1.404357
C	-0.603829	1.364077	-1.607525
H	-0.294172	1.821500	-2.558098
H	0.557146	-1.849067	2.907457
H	-1.643783	-1.004938	2.736121
O	-3.675278	-2.370269	0.443769
O	-3.538060	-0.779949	-1.077886
C	-2.235962	0.716440	0.877969
H	-1.362673	1.233755	1.301233
C	-2.601618	1.418900	-0.456649
H	-3.481975	2.062752	-0.333610
H	-1.138205	-0.467694	-2.743896
H	-0.707143	-1.927322	-0.855977
C	-3.392411	0.831589	1.870207
H	-3.579321	1.891650	2.096612
H	-4.326838	0.413913	1.461609
H	-3.182308	0.321697	2.820248
O	-1.575839	2.219163	-1.002782
O	1.632318	-1.064969	-0.915870
C	1.515464	-1.080988	-2.354387
H	0.671012	-1.714607	-2.645364
H	1.404431	-0.057994	-2.735664
H	2.435547	-1.514846	-2.746418
B	3.162236	-1.315350	-0.305746
F	3.687795	-0.068467	-0.115822
F	2.992258	-2.045211	0.832573
F	3.800594	-2.025032	-1.283582

48

20'-C_4
Eopt -1435.308601

C	-1.949242	-2.458161	0.925935
C	-1.567675	-1.449434	1.946520
C	-0.964840	-0.299877	1.613116
C	-0.819319	0.055498	0.154205
C	-0.774964	-1.137008	-0.764511
C	-1.335622	-2.310867	-0.441569
C	-0.253948	0.634414	2.534132
C	0.477994	0.807971	-0.151586
C	1.641107	0.338106	0.777662
C	1.263462	0.497986	2.268837
C	2.782697	1.259649	0.370633
C	2.318998	0.237125	-1.702241
C	0.812898	0.502148	-1.619858
H	-0.578910	1.665066	2.337057
H	-1.733147	-1.731535	2.989649
H	1.782761	1.376044	2.675449

H	2.710273	0.286432	-2.724082
O	-2.631640	-3.423277	1.207665
C	-1.300709	-3.539063	-1.291005
H	-0.596781	-4.274797	-0.869995
H	-2.292522	-4.014343	-1.298551
H	-0.992635	-3.319853	-2.320566
C	0.194963	-0.884045	-1.902355
H	-0.263928	-0.976078	-2.896641
H	-0.472781	0.401117	3.584315
H	1.630547	-0.374515	2.826012
O	3.391983	2.018958	1.073115
O	3.088347	1.154431	-0.933973
C	2.148867	-1.099545	0.400169
H	1.371793	-1.823775	0.685150
C	2.342191	-1.189354	-1.137719
H	3.256076	-1.743872	-1.387174
H	0.456934	1.285068	-2.295416
H	0.359484	1.879483	0.027876
C	3.443996	-1.469371	1.122205
H	3.702989	-2.516595	0.907254
H	4.290164	-0.846979	0.788887
H	3.358084	-1.363715	2.212643
O	1.279035	-1.809669	-1.826818
O	-1.985267	0.931234	-0.213446
C	-3.233486	0.263766	-0.488675
H	-3.451574	-0.454738	0.309413
H	-4.009047	1.030339	-0.488800
H	-3.186079	-0.231492	-1.465246
B	-1.936636	2.576103	-0.396643
F	-3.242542	2.960827	-0.463146
F	-1.301707	3.059347	0.716954
F	-1.251572	2.794844	-1.559335

48

20'-C_5
Eopt -1435.307981

C	1.927546	2.510953	0.528181
C	1.248229	1.963309	1.730567
C	0.653874	0.764707	1.715793
C	0.722908	-0.082645	0.467397
C	0.936763	0.708134	-0.794085
C	1.569014	1.891641	-0.794604
C	-0.193749	0.168993	2.788041
C	-0.580059	-0.860288	0.229855
C	-1.816544	-0.090386	0.792484
C	-1.659601	0.176564	2.304796
C	-2.978172	-1.032237	0.505487
C	-2.167436	-0.705052	-1.677866
C	-0.719044	-1.018086	-1.298317
H	0.137684	-0.865571	2.972399
H	1.251697	2.594689	2.622712
H	-2.233626	-0.575301	2.862146
H	-2.425851	-1.040789	-2.687993
O	2.646379	3.488196	0.597326
C	1.796175	2.739649	-2.004087
H	1.107036	3.599278	-2.005210
H	2.817352	3.147852	-1.984598
H	1.648233	2.177700	-2.934427
C	0.065608	0.162025	-1.909883
H	0.621034	-0.093164	-2.821503
H	-0.099319	0.724896	3.729800
H	-2.098682	1.153188	2.547870
O	-3.736243	-1.513205	1.303174
O	-3.113856	-1.303687	-0.802009
C	-2.129525	1.192872	-0.059931
H	-1.325004	1.922943	0.111239
C	-2.119202	0.822285	-1.568028

H	-2.935587	1.327213	-2.100422
H	-0.381516	-1.994887	-1.651447
H	-0.536207	-1.826097	0.743037
C	-3.460859	1.840958	0.317555
H	-3.586061	2.781230	-0.239419
H	-4.316894	1.194224	0.065705
H	-3.522102	2.078517	1.388550
O	-0.923566	1.139112	-2.246460
O	1.844570	-1.074200	0.674031
C	3.087350	-0.514571	1.160587
H	2.945902	-0.150481	2.181957
H	3.817831	-1.323305	1.160183
H	3.429592	0.288311	0.493775
B	2.045738	-2.403838	-0.300844
F	3.199225	-2.991699	0.129160
F	0.956069	-3.198536	-0.087017
F	2.135580	-1.901314	-1.571283

48

20'-C_6
Eopt -1435.309133

C	2.051972	1.564947	1.507859
C	1.347330	0.553390	2.341119
C	0.543270	-0.368506	1.797807
C	0.516633	-0.492319	0.291270
C	0.774327	0.807337	-0.425324
C	1.527203	1.772582	0.114182
C	-0.470942	-1.200087	2.520736
C	-0.831230	-0.960768	-0.253392
C	-2.027988	-0.411614	0.588695
C	-1.888648	-0.769018	2.085476
C	-3.224498	-1.081127	-0.070038
C	-2.331229	0.087818	-1.917079
C	-0.904362	-0.421825	-1.692042
H	-0.330806	-2.266038	2.295542
H	1.441339	0.672169	3.423942
H	-2.600076	-1.568479	2.332422
H	-2.582399	0.221044	-2.974879
O	2.913060	2.283230	1.973082
C	1.852549	3.074163	-0.540097
H	1.389073	3.910830	0.006423
H	2.940387	3.237554	-0.512020
H	1.509133	3.107092	-1.581039
C	-0.061448	0.874835	-1.687863
H	0.548018	1.013797	-2.589291
H	-0.362986	-1.081020	3.606999
H	-2.176402	0.104334	2.685412
O	-4.030193	-1.807765	0.444488
O	-3.324031	-0.778630	-1.376300
C	-2.264534	1.123040	0.351324
H	-1.434019	1.671524	0.819950
C	-2.228963	1.424375	-1.168149
H	-3.011099	2.141898	-1.447723
H	-0.565367	-1.153705	-2.432717
H	-0.894341	-2.056219	-0.227034
C	-3.577923	1.612674	0.960952
H	-3.648487	2.705415	0.856833
H	-4.452651	1.177633	0.450921
H	-3.659181	1.374325	2.030421
O	-1.001316	1.945044	-1.620268
O	1.545923	-1.485071	-0.156868
C	1.590421	-2.739942	0.555292
H	0.580300	-3.154967	0.628781
H	2.208002	-3.417795	-0.035725
H	2.031705	-2.590569	1.549429
B	2.927703	-1.063557	-0.948063
F	3.531910	-2.256734	-1.230652

F	2.515687	-0.411305	-2.076482
F	3.647087	-0.296987	-0.073286

48

20'-D_1
Eopt -1435.318680

C	2.339520	-2.094440	-1.000254
C	2.939013	-0.748746	-1.170782
C	2.553243	0.285856	-0.411785
C	1.609443	0.044437	0.741528
C	0.688015	-1.128817	0.513863
C	1.035498	-2.180157	-0.242179
C	2.886535	1.727624	-0.603820
C	0.674999	1.229728	0.986890
C	0.320642	1.998758	-0.315691
C	1.603330	2.474484	-1.036180
C	-0.530369	3.154704	0.184668
C	-1.798047	1.334098	0.988636
C	-0.591368	0.632041	1.623113
H	3.255886	2.137050	0.350102
H	3.673340	-0.643366	-1.974134
H	1.732244	3.551038	-0.861489
H	-2.732318	1.182906	1.539021
O	2.811463	-3.076442	-1.539831
C	0.196384	-3.387122	-0.507285
H	-0.281329	-3.308894	-1.497613
H	0.833664	-4.282369	-0.529477
H	-0.592462	-3.516480	0.241407
C	-0.663369	-0.818346	1.109058
H	-1.045533	-1.550913	1.826690
H	3.673412	1.867475	-1.356894
H	1.479534	2.344321	-2.119542
O	-0.329601	4.328687	0.046501
O	-1.619333	2.734139	0.861912
C	-0.640948	1.154507	-1.226523
H	-0.082665	0.286376	-1.605294
C	-1.815997	0.637764	-0.373160
H	-2.772591	0.704616	-0.899644
H	-0.582032	0.680987	2.716530
H	1.178041	1.925765	1.670382
C	-1.179442	1.941034	-2.420935
H	-1.779139	1.277671	-3.060937
H	-1.828440	2.773157	-2.104279
H	-0.369918	2.357501	-3.035005
O	-1.651353	-0.750417	0.013345
O	2.327066	-0.083756	1.968841
C	3.197142	-1.176087	2.126815
H	2.677626	-2.146577	2.045478
H	4.032225	-1.160570	1.405358
H	3.613700	-1.092714	3.139706
B	-2.984589	-1.680221	-0.012055
F	-3.144585	-1.995171	-1.329838
F	-3.990857	-0.893118	0.487186
F	-2.688067	-2.744097	0.794641

48

20'-D_2
Eopt -1435.314594

C	1.549412	2.861523	0.882988
C	2.540887	1.784677	1.128395
C	2.497692	0.631051	0.449228
C	1.531303	0.514062	-0.709964
C	0.272737	1.325083	-0.509808
C	0.272895	2.475638	0.174125
C	3.234176	-0.629896	0.770030
C	1.030038	-0.923660	-0.900947
C	0.891293	-1.702774	0.442888
C	2.220218	-1.700285	1.231032

C	0.481042	-3.092469	-0.015831
C	-1.280486	-1.817187	-0.933515
C	-0.344848	-0.784640	-1.575082
H	3.762997	-0.990309	-0.125507
H	3.254421	1.960006	1.938350
H	2.683109	-2.693423	1.156460
H	-2.191463	-2.001143	-1.511884
O	1.696650	3.980126	1.335282
C	-0.901777	3.373606	0.386110
H	-1.308736	3.237579	1.401593
H	-0.583571	4.422866	0.307586
H	-1.706514	3.180606	-0.331442
C	-0.890878	0.582365	-1.115406
H	-1.453466	1.137498	-1.872452
H	3.985196	-0.465562	1.554419
H	2.006404	-1.532750	2.294715
O	1.048661	-4.130187	0.181238
O	-0.661415	-3.076847	-0.734440
C	-0.325890	-1.186032	1.291496
H	-0.095298	-0.169948	1.642260
C	-1.574190	-1.113826	0.392904
H	-2.476325	-1.466909	0.901501
H	-0.294509	-0.860197	-2.665807
H	1.732369	-1.483074	-1.533475
C	-0.620741	-2.060662	2.510101
H	-1.428100	-1.606135	3.102419
H	-0.950675	-3.070921	2.219679
H	0.255610	-2.164541	3.163387
O	-1.849687	0.238352	-0.049253
O	2.071774	1.020696	-1.924585
C	3.342380	0.573526	-2.325443
H	4.137768	0.910873	-1.637774
H	3.400831	-0.524944	-2.426213
H	3.531344	1.014986	-3.313206
B	-3.411100	0.680537	-0.117016
F	-3.743591	0.916563	1.184867
F	-4.072847	-0.388985	-0.664764
F	-3.428601	1.788539	-0.918466

48

20'-D_3
Eopt -1435.315186

C	-0.865750	3.105862	-0.954775
C	-2.052676	2.260559	-1.222117
C	-2.293528	1.156599	-0.504906
C	-1.423215	0.862225	0.698415
C	-0.005492	1.368315	0.527204
C	0.267161	2.472888	-0.181606
C	-3.359148	0.138263	-0.724801
C	-1.260335	-0.650798	0.952444
C	-1.369410	-1.481502	-0.362530
C	-2.717199	-1.223538	-1.067809
C	-1.242696	-2.922321	0.106547
C	0.794073	-2.054673	0.904866
C	0.139860	-0.837398	1.568935
H	-3.939250	0.050723	0.208321
H	-2.702939	2.578486	-2.041241
H	-3.413276	-2.030284	-0.803146
H	1.674079	-2.421394	1.443273
O	-0.749642	4.217441	-1.433138
C	1.621408	3.063281	-0.399902
H	2.004857	2.782414	-1.394525
H	1.550385	4.160171	-0.382943
H	2.345764	2.729365	0.350686
C	0.971034	0.365290	1.082629
H	1.689725	0.758928	1.808327
H	-4.051697	0.443558	-1.520196

H	-2.574536	-1.282185	-2.154359
O	-2.030947	-3.813344	-0.044053
O	-0.089101	-3.151635	0.766310
C	-0.115379	-1.238625	-1.280346
H	-0.136835	-0.194534	-1.624748
C	1.164681	-1.443524	-0.445870
H	1.935039	-1.993903	-0.994083
H	0.122756	-0.908733	2.661183
H	-2.054378	-0.990305	1.632175
C	-0.076762	-2.147575	-2.508095
H	0.796195	-1.892493	-3.126321
H	0.011850	-3.210086	-2.230172
H	-0.971846	-2.033344	-3.133216
O	1.765343	-0.190098	-0.033328
O	-2.108698	1.519502	1.753500
C	-1.551496	1.446145	3.040933
H	-1.596356	0.428483	3.467385
H	-0.504489	1.799212	3.066316
H	-2.149104	2.108408	3.681668
B	3.389357	-0.104907	-0.068850
F	3.681041	0.139674	-1.378630
F	3.830374	-1.327343	0.369781
F	3.707567	0.915477	0.784433

48

20'-E_1
Eopt -1435.300319

C	3.930717	-0.110794	-0.704576
C	3.335316	1.248882	-0.774591
C	2.202925	1.548071	-0.123534
C	1.617485	0.538936	0.833177
C	1.929579	-0.885572	0.452979
C	3.047643	-1.222440	-0.204762
C	1.359311	2.769459	-0.276326
C	0.089218	0.597357	0.893185
C	-0.549517	1.015943	-0.461479
C	0.030504	2.365382	-0.954570
C	-2.017716	1.125750	-0.132675
C	-1.569108	-1.192681	0.443790
C	-0.360670	-0.818219	1.293992
H	1.158385	3.192420	0.720935
H	3.842563	1.966049	-1.426056
H	-0.709985	3.161501	-0.800111
H	-2.141322	-2.039658	0.826913
O	5.043168	-0.340291	-1.141469
C	3.438215	-2.613528	-0.592339
H	3.330487	-2.756239	-1.679663
H	4.497462	-2.787801	-0.352295
H	2.824348	-3.368852	-0.085409
C	0.735076	-1.767260	0.749247
H	0.959616	-2.614319	1.412109
H	1.861523	3.541679	-0.874217
H	0.204438	2.302589	-2.037270
O	-2.754730	2.054744	-0.205480
O	-2.512206	-0.097680	0.324103
C	-0.460480	-0.135040	-1.528652
H	0.592058	-0.217723	-1.836967
C	-0.869034	-1.486907	-0.884051
H	-1.479950	-2.081791	-1.575084
H	-0.524879	-0.919518	2.371516
H	-0.191542	1.341879	1.649794
C	-1.320710	0.147202	-2.759674
H	-1.133134	-0.617340	-3.527493
H	-2.396120	0.113423	-2.521740
H	-1.103747	1.128412	-3.204024
O	0.208752	-2.293620	-0.473751
O	2.015532	0.826458	2.175204

C	3.372011	0.694531	2.516492
H	3.739031	-0.339663	2.399715
H	4.024034	1.368404	1.933912
H	3.450226	0.971638	3.576543
B	-4.291677	-0.372467	0.394845
F	-4.677780	0.454007	1.382090
F	-4.320281	-1.692432	0.688945
F	-4.667921	-0.049806	-0.858589

48

20'-E_2
Eopt -1435.296841

C	4.018732	0.039276	-0.648243
C	3.380418	1.379233	-0.623751
C	2.229790	1.588897	0.026684
C	1.668720	0.478803	0.887924
C	2.001938	-0.896799	0.353379
C	3.146694	-1.137327	-0.300048
C	1.392480	2.821695	0.036100
C	0.126363	0.497187	0.956518
C	-0.512670	1.101040	-0.332706
C	0.024024	2.524187	-0.612174
C	-1.988521	1.125482	-0.019323
C	-1.496281	-1.238656	0.198308
C	-0.314585	-0.970738	1.122497
H	1.249036	3.127097	1.085429
H	3.887509	2.169995	-1.182997
H	-0.707859	3.264393	-0.263001
H	-2.051003	-2.147616	0.438868
O	5.156569	-0.117303	-1.050105
C	3.570009	-2.464277	-0.845471
H	3.471326	-2.480412	-1.942951
H	4.631886	-2.640269	-0.618541
H	2.971528	-3.288018	-0.435729
C	0.808205	-1.816870	0.473563
H	1.022224	-2.753874	1.007260
H	1.884678	3.651308	-0.488436
H	0.113851	2.662579	-1.697478
O	-2.743987	2.040610	0.043540
O	-2.463866	-0.161944	0.226514
C	-0.373510	0.127147	-1.560833
H	0.686216	0.113790	-1.855023
C	-0.763513	-1.314966	-1.140606
H	-1.345934	-1.809616	-1.928322
H	-0.511344	-1.241058	2.165007
H	-0.185647	1.118868	1.807647
C	-1.215422	0.570928	-2.756831
H	-1.002002	-0.076565	-3.619641
H	-2.294355	0.490978	-2.547136
H	-1.005208	1.606612	-3.056556
O	0.322915	-2.154500	-0.830996
O	2.264215	0.729793	2.153391
C	1.958446	-0.145527	3.208543
H	0.911457	-0.054320	3.548406
H	2.160847	-1.201067	2.951750
H	2.610943	0.131909	4.047506
B	-4.241590	-0.477430	0.241552
F	-4.641244	0.164989	1.352503
F	-4.242654	-1.827873	0.310139
F	-4.621086	0.044764	-0.940836

48

20'-E_3
Eopt -1435.296449

C	-4.021280	-0.308164	0.529721
C	-3.443091	1.011327	0.898208
C	-2.279973	1.431412	0.383597
C	-1.657596	0.623510	-0.734130

C	-1.948402	-0.853455	-0.623398
C	-3.092957	-1.309736	-0.101409
C	-1.441394	2.572594	0.862565
C	-0.124080	0.707923	-0.737683
C	0.476594	0.824807	0.697687
C	-0.139967	2.012749	1.476761
C	1.947615	1.025313	0.434429
C	1.552732	-1.120215	-0.641348
C	0.346529	-0.592646	-1.410353
H	-1.191517	3.233017	0.018090
H	-3.978921	1.574598	1.667522
H	0.596694	2.823282	1.557043
H	2.148100	-1.854194	-1.187517
O	-5.158719	-0.611712	0.837269
C	-3.491484	-2.748596	-0.009304
H	-3.467979	-3.091412	1.037689
H	-4.525513	-2.876239	-0.362505
H	-2.828237	-3.396237	-0.596674
C	-0.743656	-1.644854	-1.082940
H	-0.963200	-2.328248	-1.914807
H	-1.973672	3.175830	1.610403
H	-0.359223	1.685003	2.501573
O	2.666809	1.924442	0.727714
O	2.470833	-0.058588	-0.274161
C	0.396438	-0.531252	1.490056
H	-0.658370	-0.698153	1.752762
C	0.843925	-1.705548	0.581940
H	1.459111	-2.423691	1.138927
H	0.521670	-0.472815	-2.484235
H	0.196500	1.593412	-1.303829
C	1.229191	-0.502441	2.771536
H	1.048690	-1.419545	3.350871
H	2.309018	-0.460059	2.555695
H	0.978788	0.353682	3.413076
O	-0.214229	-2.422644	-0.005670
O	-2.169216	0.979028	-2.013999
C	-2.196290	2.340064	-2.362504
H	-2.907001	2.913980	-1.741932
H	-1.203404	2.821136	-2.301829
H	-2.533416	2.389193	-3.406741
B	4.253469	-0.256437	-0.417144
F	4.605910	0.795475	-1.177047
F	4.315864	-1.464215	-1.023276
F	4.636404	-0.235851	0.875025

48

20'-F_1
Eopt -1435.315945

C	3.884622	-0.451764	0.922361
C	2.932549	-1.568108	1.158986
C	1.798979	-1.679230	0.453480
C	1.568764	-0.753408	-0.716168
C	2.242377	0.585164	-0.551806
C	3.373306	0.735664	0.151463
C	0.647497	-2.591642	0.716790
C	0.089416	-0.418652	-0.921928
C	-0.703208	-0.398796	0.419202
C	-0.567967	-1.747278	1.165258
C	-2.096460	-0.083923	-0.023526
C	-1.039949	1.784709	-0.925684
C	0.066603	0.966420	-1.590178
H	0.403844	-3.132954	-0.211429
H	3.185524	-2.255319	1.971104
H	-1.482757	-2.339372	1.032369
H	-1.362685	2.662544	-1.492458
O	4.990992	-0.443312	1.428750
C	4.112338	2.022781	0.337438

H	3.983321	2.396793	1.365892
H	5.190447	1.861588	0.190529
H	3.765995	2.799744	-0.355765
C	1.354927	1.678133	-1.109885
H	1.836971	2.294316	-1.881222
H	0.884463	-3.337043	1.487395
H	-0.476274	-1.549414	2.241210
O	-3.087352	-0.783251	0.240393
O	-2.234028	1.009335	-0.705303
C	-0.325483	0.864449	1.288331
H	0.701962	0.715334	1.649411
C	-0.332917	2.132914	0.391450
H	-0.789378	2.980565	0.917750
H	-0.016976	0.906490	-2.679821
H	-0.350580	-1.190899	-1.566286
C	-1.253874	1.046009	2.488494
H	-0.893869	1.879267	3.108961
H	-2.284522	1.289464	2.182272
H	-1.292803	0.150141	3.123243
O	0.935333	2.546755	-0.052774
O	1.940699	-1.383885	-1.942417
C	3.294737	-1.695564	-2.153368
H	3.939958	-0.800229	-2.151532
H	3.685004	-2.414005	-1.411852
H	3.351293	-2.160424	-3.146928
B	-4.600984	-0.404989	-0.102751
F	-4.690456	-0.331584	-1.469340
F	-4.840932	0.792229	0.527285
F	-5.314913	-1.445538	0.429552

48

20'-F_2
Eopt -1435.312499

C	3.956447	0.651612	-0.845440
C	2.958216	1.729147	-1.060799
C	1.805841	1.755044	-0.381086
C	1.602751	0.762171	0.742880
C	2.310606	-0.551811	0.492733
C	3.466983	-0.607824	-0.181689
C	0.652952	2.681615	-0.566645
C	0.121592	0.366525	0.929398
C	-0.676236	0.467319	-0.410958
C	-0.599269	1.890839	-1.003893
C	-2.060126	0.060437	-0.015991
C	-0.951260	-1.863761	0.667643
C	0.122065	-1.090179	1.432250
H	0.459367	3.175383	0.399521
H	3.206887	2.478639	-1.816788
H	-1.495268	2.457130	-0.718763
H	-1.243807	-2.810368	1.130482
O	5.084502	0.729199	-1.294444
C	4.247071	-1.853150	-0.459866
H	4.142076	-2.146804	-1.516837
H	5.317586	-1.670077	-0.285081
H	3.917427	-2.693948	0.163771
C	1.434451	-1.715303	0.898936
H	1.914292	-2.407409	1.605176
H	0.876835	3.465738	-1.301674
H	-0.611424	1.821738	-2.099091
O	-3.069327	0.760147	-0.196727
O	-2.169210	-1.110667	0.527199
C	-0.249214	-0.676090	-1.414720
H	0.775235	-0.453904	-1.746322
C	-0.222916	-2.038629	-0.670201
H	-0.648389	-2.832899	-1.296223
H	0.017321	-1.164339	2.519495
H	-0.347077	1.053018	1.648491

C	-1.163276	-0.756132	-2.636965
H	-0.773297	-1.508786	-3.337133
H	-2.187976	-1.061229	-2.368015
H	-1.225971	0.198533	-3.176051
O	1.052441	-2.464123	-0.259623
O	2.115838	1.451872	1.872477
C	2.093886	0.790074	3.111683
H	1.070521	0.658185	3.505546
H	2.592530	-0.195090	3.071180
H	2.648098	1.423939	3.817007
B	-4.572185	0.305567	0.101468
F	-4.650180	0.056639	1.448000
F	-4.788008	-0.807065	-0.674998
F	-5.313073	1.388411	-0.290858

48

20'-F_3
Eopt -1435.312019

C	-3.985273	0.379561	0.861365
C	-3.028445	1.447083	1.256886
C	-1.860471	1.607130	0.621284
C	-1.615770	0.803491	-0.637821
C	-2.284465	-0.549734	-0.610559
C	-3.444014	-0.749358	0.026653
C	-0.694786	2.429371	1.070293
C	-0.128573	0.485300	-0.850730
C	0.633689	0.274826	0.498339
C	0.478189	1.493591	1.437771
C	2.033483	0.026326	0.036152
C	0.993847	-1.708089	-1.120807
C	-0.106985	-0.807664	-1.682369
H	-0.380419	3.103933	0.259391
H	-3.299149	2.034828	2.138554
H	1.408307	2.076596	1.451099
H	1.326386	-2.499664	-1.797842
O	-5.121468	0.352313	1.295066
C	-4.209605	-2.034003	0.054516
H	-4.165194	-2.489676	1.056850
H	-5.271430	-1.843612	-0.161175
H	-3.819530	-2.760399	-0.669673
C	-1.402211	-1.568313	-1.298937
H	-1.891465	-2.064866	-2.147945
H	-0.954154	3.051882	1.937028
H	0.321440	1.130785	2.461985
O	3.017789	0.690882	0.397405
O	2.183319	-0.967932	-0.781786
C	0.253388	-1.096378	1.183316
H	-0.776308	-1.000519	1.555634
C	0.272613	-2.233078	0.128513
H	0.724471	-3.143710	0.541290
H	-0.019069	-0.615923	-2.756359
H	0.360127	1.316599	-1.376975
C	1.179621	-1.434143	2.351399
H	0.813979	-2.336721	2.861729
H	2.208105	-1.644473	2.014874
H	1.226966	-0.625719	3.093594
O	-0.991302	-2.580609	-0.377356
O	-2.152508	1.425797	-1.798545
C	-1.839162	2.774799	-2.037467
H	-2.272821	3.444290	-1.273930
H	-0.751938	2.961504	-2.098960
H	-2.281212	3.030113	-3.009963
B	4.535216	0.374478	0.012113
F	4.625930	0.476485	-1.352765
F	4.787306	-0.890365	0.485500
F	5.237848	1.346596	0.673397

48

20-Al_1 Eopt -2733.859874

C	-1.395716	0.413057	-0.072638
C	-0.933238	-0.937425	0.194322
C	0.287319	-1.130318	0.732279
C	1.065219	0.064057	1.221422
C	0.750110	1.329182	0.467549
C	-0.451049	1.543852	-0.097364
C	1.027105	-2.410951	0.860950
C	2.574747	-0.112982	1.070798
C	2.948505	-0.980648	-0.161383
C	2.255596	-2.360387	-0.084800
C	4.462487	-1.079000	-0.071518
C	4.203156	1.265471	-0.177476
C	3.145453	1.309072	0.930907
H	1.365092	-2.512035	1.904698
H	-1.573162	-1.769200	-0.109543
H	2.985153	-3.112370	0.243785
H	4.859479	2.142429	-0.186188
O	-2.588489	0.642601	-0.401224
C	-0.869906	2.786067	-0.820327
H	-1.013036	2.580854	-1.892585
H	-1.831197	3.150177	-0.428983
H	-0.119757	3.579030	-0.717521
C	2.011745	2.150767	0.299956
H	1.929625	3.172556	0.695586
H	0.396180	-3.275982	0.620235
H	1.925475	-2.657965	-1.088913
O	5.127408	-2.075042	0.011882
O	5.054597	0.129033	-0.089195
C	2.678837	-0.207229	-1.501408
H	1.589617	-0.110597	-1.630213
C	3.269682	1.224706	-1.394232
H	3.759012	1.519477	-2.331280
H	3.507933	1.714417	1.881126
H	2.946693	-0.616039	1.973153
C	3.245917	-0.930444	-2.721901
H	2.947032	-0.402565	-3.639423
H	4.347372	-0.959174	-2.702863
H	2.883451	-1.964910	-2.798783
O	2.321637	2.227005	-1.091074
O	0.860538	0.219105	2.624418
C	-0.385557	0.682054	3.091320
H	-0.570251	1.734534	2.818975
H	-1.233069	0.068065	2.740743
H	-0.343346	0.605612	4.185884
Al	-4.211938	-0.229897	-0.319057
Cl	-5.618203	1.236791	-0.962105
Cl	-4.400252	-0.776636	1.742056
Cl	-4.072787	-1.916195	-1.628619

48

20-Al_2 Eopt -2733.857596

C	1.324074	0.369806	-0.332626
C	0.531561	1.467755	-0.862535
C	-0.699167	1.709173	-0.372291
C	-1.164462	0.928233	0.829777
C	-0.538516	-0.437479	0.930338
C	0.693173	-0.702677	0.455664
C	-1.728796	2.633326	-0.911203
C	-2.670372	0.670934	0.817924
C	-3.231345	0.525392	-0.622124
C	-2.905394	1.783156	-1.459402
C	-4.724118	0.342611	-0.402523
C	-3.894745	-1.475780	0.852798
C	-2.874237	-0.626586	1.618919
H	-2.085708	3.273771	-0.088476
H	0.973908	2.052300	-1.671937

H	-3.800609	2.416176	-1.519249
H	-4.314978	-2.293175	1.448704
O	2.531489	0.300096	-0.669538
C	1.396518	-2.019997	0.579805
H	2.230387	-1.947191	1.294996
H	0.707826	-2.797208	0.930552
H	1.814457	-2.339283	-0.385747
C	-1.560996	-1.425778	1.454567
H	-1.237799	-1.960029	2.358173
H	-1.328241	3.282106	-1.700439
H	-2.661431	1.483997	-2.487172
O	-5.614826	1.022752	-0.833163
O	-5.000515	-0.719472	0.374736
C	-2.756781	-0.821567	-1.275817
H	-1.674294	-0.750804	-1.464476
C	-2.983414	-1.984438	-0.271134
H	-3.371869	-2.874755	-0.781487
H	-3.140063	-0.441158	2.664581
H	-3.161045	1.528405	1.296788
C	-3.455504	-1.114937	-2.602264
H	-4.534448	-1.292465	-2.465294
H	-3.337288	-0.296311	-3.325616
H	-3.028153	-2.022226	-3.054029
O	-1.824093	-2.384415	0.430044
O	-0.940134	1.654430	2.036675
C	0.366055	2.078161	2.350977
H	0.317014	2.476278	3.373037
H	1.099690	1.253575	2.342358
H	0.720291	2.876805	1.677796
Al	4.275770	-0.109413	-0.310070
Cl	4.396608	-0.161905	1.830774
Cl	4.701039	-1.973719	-1.262160
Cl	5.388869	1.512718	-1.144807

48

20-Al_3 Eopt -2733.855038

C	1.445690	0.414969	-0.198259
C	0.985762	-0.898153	0.226154
C	-0.250842	-1.314904	-0.105412
C	-1.039543	-0.488461	-1.095768
C	-0.725857	0.986007	-1.017293
C	0.491388	1.432539	-0.667873
C	-0.993209	-2.466692	0.473604
C	-2.550207	-0.569769	-0.856648
C	-2.902448	-0.701060	0.655592
C	-2.193862	-1.920601	1.287086
C	-4.415372	-0.841702	0.639052
C	-4.178059	1.245733	-0.439368
C	-3.119133	0.740900	-1.426493
H	-1.367530	-3.104330	-0.341981
H	1.632634	-1.475136	0.891243
H	-2.922427	-2.730425	1.425161
H	-4.843364	2.004354	-0.865711
O	2.645666	0.763326	-0.056400
C	0.926509	2.864814	-0.647725
H	1.151734	3.188758	0.380160
H	1.846493	2.993737	-1.236984
H	0.148090	3.521554	-1.054024
C	-1.983518	1.784350	-1.285451
H	-1.887924	2.467369	-2.140569
H	-0.349088	-3.081847	1.115060
H	-1.829934	-1.649326	2.286613
O	-5.070951	-1.752610	1.065980
O	-5.018783	0.208891	0.054246
C	-2.637658	0.640233	1.429651
H	-1.549580	0.799695	1.481328
C	-3.246811	1.824245	0.633617

H	-3.740818	2.538868	1.303963
H	-3.480467	0.633890	-2.454371
H	-2.961474	-1.449975	-1.369179
C	-3.187038	0.615327	2.855326
H	-4.287577	0.561507	2.868196
H	-2.801294	-0.232950	3.437345
H	-2.897655	1.537836	3.379818
O	-2.307072	2.551839	-0.128297
O	-0.709577	-0.810229	-2.439307
C	-0.746093	-2.163037	-2.831598
H	-0.561894	-2.173740	-3.913842
H	0.043508	-2.756256	-2.338540
H	-1.724557	-2.638751	-2.645560
Al	4.270482	-0.028213	0.320407
Cl	5.679030	1.554795	0.088699
Cl	4.458526	-1.587540	-1.129737
Cl	4.125645	-0.754681	2.328546

48

20-Al_4 Eopt -2733.853691

C	-1.381254	-0.504183	0.008231
C	-0.597150	-1.629506	-0.467015
C	0.686704	-1.766470	-0.088014
C	1.219932	-0.854013	0.993044
C	0.555396	0.504817	0.993716
C	-0.728054	0.665709	0.624500
C	1.686800	-2.742455	-0.584472
C	2.720974	-0.551883	0.836934
C	3.164097	-0.604476	-0.655434
C	2.849182	-1.983499	-1.271660
C	4.658345	-0.331611	-0.599113
C	3.842647	1.626022	0.427026
C	2.936550	0.866281	1.401337
H	2.070863	-3.292447	0.290491
H	-1.087333	-2.319173	-1.156813
H	3.752013	-2.606671	-1.231036
H	4.264792	2.541205	0.856102
O	-2.607806	-0.489677	-0.245988
C	-1.479059	1.960690	0.657410
H	-2.355264	1.875967	1.318749
H	-0.846549	2.776908	1.025004
H	-1.846649	2.231647	-0.343705
C	1.579088	1.590483	1.246767
H	1.322005	2.252556	2.085423
H	1.240647	-3.471063	-1.273004
H	2.603443	-1.859943	-2.334237
O	5.544123	-1.039278	-0.994230
O	4.943841	0.851139	-0.027222
C	2.565120	0.605911	-1.460440
H	1.475457	0.465718	-1.537908
C	2.815040	1.919574	-0.671420
H	3.113080	2.732810	-1.345256
H	3.308976	0.863112	2.430994
H	3.296065	-1.309562	1.387870
C	3.134814	0.718999	-2.873605
H	4.211207	0.954753	-2.861808
H	2.997524	-0.202819	-3.454886
H	2.626443	1.531424	-3.413266
O	1.701154	2.380803	0.064049
O	0.917639	-1.583883	2.170727
C	1.300980	-1.023997	3.405229
H	0.886496	-1.677860	4.183669
H	2.396560	-0.991130	3.531034
H	0.888620	-0.009606	3.550485
Al	-4.357259	0.010292	-0.304702
Cl	-4.901312	0.609115	1.677662
Cl	-4.454231	1.586893	-1.747059

Cl	-5.400137	-1.747232	-0.931991

48

20-Al_5 Eopt -2733.852792

C	1.381896	0.460849	-0.100514
C	0.607871	1.461809	-0.819363
C	-0.680080	1.682985	-0.496083
C	-1.214004	1.042454	0.764828
C	-0.558356	-0.279329	1.082636
C	0.719398	-0.537416	0.756144
C	-1.695005	2.419395	-1.297026
C	-2.707305	0.717860	0.672699
C	-3.134516	0.309904	-0.769168
C	-2.758679	1.406387	-1.791958
C	-4.635642	0.116086	-0.635713
C	-3.868474	-1.446599	0.961397
C	-2.927216	-0.450224	1.648500
H	-2.183076	3.176341	-0.664381
H	1.098814	1.946842	-1.665893
H	-3.663725	1.958369	-2.078426
H	-4.318112	-2.167418	1.652805
O	2.609169	0.383471	-0.337892
C	1.470041	-1.780534	1.120353
H	2.318923	-1.536211	1.777648
H	0.821644	-2.494099	1.641577
H	1.877284	-2.274181	0.225476
C	-1.581167	-1.213484	1.694708
H	-1.297379	-1.570514	2.694121
H	-1.238674	2.936606	-2.150835
H	-2.375202	0.933642	-2.705493
O	-5.504535	0.689812	-1.233647
O	-4.949003	-0.815208	0.283341
C	-2.586041	-1.111648	-1.153564
H	-1.494358	-1.038399	-1.275806
C	-2.863331	-2.103351	0.006587
H	-3.193822	-3.077214	-0.376070
H	-3.268727	-0.119284	2.634592
H	-3.296493	1.600315	0.957713
C	-3.182861	-1.639437	-2.457720
H	-4.262403	-1.838092	-2.360430
H	-3.043225	-0.942533	-3.295471
H	-2.697593	-2.588416	-2.729361
O	-1.750414	-2.343011	0.841331
O	-0.926636	1.819778	1.918782
C	-1.287491	3.181473	1.913754
H	-1.097612	3.554151	2.928865
H	-0.674766	3.767131	1.206555
H	-2.354767	3.341802	1.682920
Al	4.358039	-0.119883	-0.264295
Cl	5.406239	1.422573	-1.309958
Cl	4.465213	-2.006925	-1.265903
Cl	4.885364	-0.198718	1.808650

48

20-Al_6 Eopt -2733.855637

C	1.453443	-0.432292	-0.034434
C	0.971482	0.827426	0.499866
C	-0.273858	0.907921	1.005310
C	-1.070811	-0.365785	1.179050
C	-0.724117	-1.416693	0.147337
C	0.514202	-1.518473	-0.365362
C	-0.993717	2.123308	1.455360
C	-2.586578	-0.150447	1.016576
C	-2.897614	1.037557	0.058501
C	-2.248598	2.338873	0.573304
C	-4.415442	1.120548	0.057519
C	-4.124109	-1.093343	-0.691764
C	-3.148423	-1.461555	0.430486

H	-1.301716	1.951573	2.500012
H	1.626688	1.698572	0.434319
H	-2.992504	2.904426	1.149251
H	-4.765345	-1.927890	-0.995516
O	2.659239	-0.588178	-0.356420
C	0.968616	-2.552936	-1.347783
H	1.154593	-2.097793	-2.333111
H	1.914819	-3.004844	-1.015744
H	0.218816	-3.344190	-1.468215
C	-1.974986	-2.117064	-0.334940
H	-1.931086	-3.211544	-0.244849
H	-0.348525	3.010584	1.434869
H	-1.970024	2.970199	-0.279936
O	-5.093905	2.061412	0.367353
O	-4.993520	-0.024304	-0.345260
C	-2.513879	0.672384	-1.421588
H	-1.417424	0.590328	-1.484081
C	-3.107146	-0.718808	-1.775314
H	-3.526110	-0.721315	-2.789552
H	-3.585743	-2.115711	1.191993
H	-3.021533	0.083764	1.998680
C	-2.974005	1.720381	-2.433299
H	-4.072913	1.789316	-2.478990
H	-2.581694	2.721195	-2.207501
H	-2.620209	1.444502	-3.437573
O	-2.180571	-1.782541	-1.707782
O	-0.701978	-0.768652	2.487285
C	-1.276824	-1.948859	2.998111
H	-0.793768	-2.133892	3.966418
H	-2.362434	-1.851114	3.168903
H	-1.089395	-2.820929	2.346628
Al	4.275722	0.280841	-0.182255
Cl	4.587332	0.448639	1.924628
Cl	5.658461	-1.010772	-1.166083
Cl	4.042513	2.173843	-1.155588

48

20-B_1 Eopt -1435.321616

C	-2.202157	0.313866	-0.184325
C	-1.710004	-1.046712	-0.034374
C	-0.510359	-1.255081	0.541125
C	0.209024	-0.096092	1.180426
C	-0.103620	1.225151	0.529052
C	-1.280481	1.460960	-0.074815
C	0.261144	-2.523854	0.580691
C	1.728573	-0.222723	1.090466
C	2.186636	-0.955718	-0.199833
C	1.529563	-2.351844	-0.293995
C	3.696471	-1.027914	-0.043572
C	3.378191	1.308789	0.068271
C	2.264310	1.219350	1.117208
H	0.552634	-2.720006	1.624957
H	-2.308106	-1.861964	-0.441688
H	2.264378	-3.115718	-0.007048
H	4.009945	2.196700	0.179578
O	-3.387356	0.572270	-0.518033
C	-1.696777	2.754319	-0.703856
H	-1.867401	2.624093	-1.783540
H	-2.642748	3.106388	-0.266250
H	-0.931390	3.526788	-0.565245
C	1.139844	2.090706	0.509330
H	1.006678	3.065387	0.998693
H	-0.332825	-3.376043	0.226519
H	1.253777	-2.554785	-1.337393
O	4.384242	-2.011880	-0.028239
O	4.255016	0.188555	0.090804
C	1.962156	-0.059584	-1.470313

H	0.878406	0.026869	-1.642549
C	2.509576	1.367266	-1.194862
H	3.037944	1.761627	-2.072236
H	2.565670	1.539574	2.119903
H	2.071585	-0.802023	1.957964
C	2.605758	-0.645508	-2.725918
H	2.336400	-0.035703	-3.600855
H	3.705485	-0.651481	-2.655375
H	2.275435	-1.674781	-2.922643
O	1.521522	2.314683	-0.847267
O	-0.065650	-0.069903	2.579834
C	-1.358298	0.279092	3.018154
H	-1.604586	1.332338	2.803077
H	-2.145239	-0.366520	2.591693
H	-1.355369	0.136878	4.106969
B	-4.569891	-0.454476	-0.602460
F	-4.280812	-1.333098	-1.626482
F	-4.631126	-1.080219	0.625439
F	-5.670364	0.319780	-0.862532

48

20-B_2 Eopt -1435.317725

C	-2.281470	0.289492	-0.113715
C	-1.766698	-1.002155	0.307090
C	-0.537467	-1.082004	0.848903
C	0.203062	0.193585	1.179248
C	-0.147357	1.328086	0.242583
C	-1.367309	1.431647	-0.309647
C	0.217287	-2.310696	1.197808
C	1.730605	0.049186	1.053287
C	2.123750	-1.020646	-0.008856
C	1.501273	-2.389756	0.336086
C	3.642279	-1.049838	0.052490
C	3.294240	1.218501	-0.482287

C	2.260878	1.432992	0.628035
H	0.490026	-2.237403	2.263445
H	-2.379512	-1.887147	0.137967
H	2.246873	-2.997902	0.864759
H	3.914390	2.101687	-0.670481
O	-3.479490	0.463315	-0.455487
C	-1.823157	2.539400	-1.208069
H	-2.003125	2.165079	-2.227727
H	-2.772251	2.960156	-0.844605
H	-1.076486	3.340703	-1.260296
C	1.091271	2.119909	-0.115236
H	0.999999	3.196861	0.084842
H	-0.391586	-3.214108	1.066515
H	1.262985	-2.925197	-0.591896
O	4.344758	-1.991538	0.300376
O	4.190424	0.149859	-0.208919
C	1.791549	-0.518491	-1.461049
H	0.696759	-0.477279	-1.570199
C	2.336737	0.923943	-1.642326
H	2.794729	1.049750	-2.631618
H	2.641161	2.020519	1.470232
H	2.139834	-0.270855	2.022110
C	2.347761	-1.436809	-2.548385
H	3.449800	-1.444414	-2.551893
H	2.003398	-2.474034	-2.437806
H	2.018984	-1.081405	-3.536024
O	1.364596	1.939054	-1.505021
O	-0.223109	0.450838	2.506972
C	0.292714	1.590376	3.153331
H	-0.229954	1.663250	4.116158
H	1.374354	1.509540	3.355763
H	0.098703	2.517909	2.585703
F	-5.752694	0.111985	-0.774130

F -4.321170 -1.617557 -1.228740

F -4.715050 -0.996936 0.941737

B -4.643191 -0.585141 -0.370769

48

20-B_3 Eopt -1435.317169

C -2.264732 0.385434 0.042675

C -1.783075 -0.938968 -0.324403

C -0.560950 -1.336887 0.074923

C 0.187125 -0.479624 1.069325

C -0.125705 0.989769 0.930081

C -1.327110 1.420198 0.515093

C 0.206974 -2.502761 -0.441159

C 1.706229 -0.563720 0.891501

C 2.119473 -0.739635 -0.600575

C 1.437403 -1.978598 -1.223270

C 3.630574 -0.875758 -0.519309

C 3.347203 1.242495 0.485555

C 2.249501 0.765172 1.443570

H 0.550050 -3.117428 0.405134

H -2.396776 -1.541483 -0.994488

H 2.172029 -2.790167 -1.310176

H 3.993872 2.015072 0.915468

O -3.454223 0.747490 -0.146706

C -1.758779 2.851423 0.430716

H -1.993121 3.128237 -0.608593

H -2.671708 3.012553 1.023231

H -0.973633 3.522480 0.798509

C 1.118636 1.800530 1.223925

H 0.987175 2.509035 2.053163

H -0.410743 -3.137573 -1.089476

H 1.112119 -1.737146 -2.243633

O 4.304441 -1.796039 -0.894526

O 4.208483 0.193099 0.058239

C 1.885626 0.576849 -1.425663

H 0.800496 0.730806 -1.528121

C 2.459798 1.786181 -0.641687

H 2.979763 2.481555 -1.312706

H 2.568647 0.690864 2.488176

H 2.097992 -1.427489 1.445569

C 2.494458 0.509691 -2.825747

H 3.594733 0.459787 -2.791209

H 2.135661 -0.357785 -3.396605

H 2.224801 1.414370 -3.390314

O 1.489430 2.534150 0.059041

O -0.192617 -0.758327 2.409493

C -0.141511 -2.093097 2.855854

H -0.372704 -2.067359 3.928877

H -0.893444 -2.726013 2.353398

H 0.855414 -2.550064 2.731147

F -4.716181 -1.218103 0.251092

F -4.353586 -0.558669 -1.913606

F -5.738185 0.665534 -0.560309

B -4.643997 -0.157253 -0.624941

53

21-ROT_1_1
Eopt -1583.906139

C 2.229742 0.411780 0.013355

C 1.452284 0.499084 -1.289091

C 0.184065 0.905269 -1.330531

C -0.460931 1.369975 -0.047480

C 0.053862 0.623230 1.155726

C 1.309144 0.179911 1.221636

C -0.734026 0.843410 -2.513154

C -1.973921 1.110564 -0.026343

C -2.373670 -0.192624 -0.793520

C -1.856028 -0.178885 -2.248613

C	-3.890141	-0.177028	-0.709816
C	-3.346024	-0.180127	1.585888
C	-2.339945	0.968517	1.460511
H	-1.177784	1.836723	-2.685661
H	1.984456	0.157428	-2.179827
H	-2.697049	0.012815	-2.929005
H	-3.891441	-0.180377	2.536171
O	3.254314	-0.509792	-0.047621
C	1.903302	-0.538598	2.390072
H	2.056029	-1.598965	2.141858
H	2.890982	-0.124438	2.647918
H	1.252916	-0.480678	3.272207
C	-1.070551	0.399871	2.144322
H	-0.866468	0.842858	3.129907
H	-0.186297	0.567813	-3.425180
H	-1.470494	-1.176282	-2.498308
O	-4.676770	-0.161505	-1.618724
O	-4.333903	-0.171825	0.560248
C	-1.946577	-1.487500	-0.011486
H	-0.849584	-1.556977	-0.041736
C	-2.372802	-1.361822	1.473585
H	-2.787991	-2.306888	1.847556
H	-2.682128	1.910523	1.902587
H	-2.507182	1.946507	-0.500592
C	-2.529662	-2.760742	-0.623574
H	-3.627310	-2.796785	-0.527821
H	-2.280753	-2.865430	-1.688571
H	-2.124354	-3.640148	-0.101512
O	-1.330782	-0.988897	2.344113
O	-0.168277	2.743095	0.236855
C	-0.424676	3.689688	-0.767909
H	-0.214612	4.677531	-0.334739
H	0.226592	3.552488	-1.649963
H	-1.476659	3.683180	-1.104873
C	2.936436	1.824686	0.262833
H	2.775288	2.548847	-0.548739
H	2.686893	2.291123	1.226977
B	3.050365	-1.866414	-0.570234
F	3.996981	-2.692470	0.039675
F	3.239957	-1.891592	-1.965767
F	1.749877	-2.326408	-0.283636
N	5.420749	1.408818	0.322777
N	4.347514	1.626164	0.301884

53

21-ROT_1_2
Eopt -1583.906514

C	2.228616	0.390004	-0.170347
C	1.430224	0.071851	-1.422801
C	0.163602	0.453648	-1.564868
C	-0.464291	1.286744	-0.474675
C	0.067440	0.926892	0.892909
C	1.328531	0.524663	1.066926
C	-0.749200	0.168791	-2.712925
C	-1.986668	1.063734	-0.357712
C	-2.407551	-0.374394	-0.805259
C	-1.960204	-0.664291	-2.252102
C	-3.921217	-0.363267	-0.670965
C	-3.309292	0.146563	1.543101
C	-2.337214	1.255341	1.131896
H	-1.096734	1.137387	-3.110024
H	1.962825	-0.487844	-2.195142
H	-2.807251	-0.488036	-2.928736
H	-3.821606	0.351079	2.489865
O	3.267504	-0.492233	0.037767
C	1.932974	0.153121	2.382721
H	2.048216	-0.938156	2.450915

H	2.938913	0.588297	2.489666
H	1.308648	0.487112	3.221414
C	-1.049500	0.892977	1.911865
H	-0.864484	1.541833	2.781003
H	-0.220334	-0.347680	-3.525731
H	-1.708032	-1.728893	-2.342734
O	-4.733897	-0.564824	-1.533513
O	-4.328562	-0.087978	0.580165
C	-1.921346	-1.456436	0.228244
H	-0.823807	-1.508445	0.179968
C	-2.310929	-1.010948	1.662634
H	-2.691955	-1.858012	2.247989
H	-2.707674	2.261750	1.355527
H	-2.511256	1.782563	-1.004029
C	-2.483568	-2.847608	-0.060514
H	-3.578411	-2.882517	0.064241
H	-2.248064	-3.193284	-1.076242
H	-2.048760	-3.572947	0.643018
O	-1.259958	-0.432943	2.401165
O	-0.125001	2.626037	-0.846545
C	-0.558196	3.664174	-0.007281
H	-0.093871	4.589250	-0.376479
H	-1.653656	3.801137	-0.032734
H	-0.244653	3.516401	1.042534
C	2.912351	1.820390	-0.391618
H	2.733010	2.244343	-1.390086
H	2.659928	2.566662	0.375660
B	3.079245	-1.945760	-0.050955
F	4.017806	-2.538405	0.796760
F	3.292951	-2.390588	-1.369418
F	1.775757	-2.309092	0.343148
N	5.403102	1.479452	-0.228429
N	4.326339	1.665317	-0.304025

53

21-ROT_1_3
Eopt -1583.904547

C	2.077209	0.138957	0.288809
C	1.411230	-0.002530	-1.073736
C	0.175024	0.445712	-1.296597
C	-0.537498	1.171973	-0.182179
C	-0.180027	0.611410	1.169983
C	1.037808	0.129879	1.421940
C	-0.641710	0.239271	-2.536428
C	-2.063191	1.024812	-0.258239
C	-2.516426	-0.352394	-0.844300
C	-1.885708	-0.616942	-2.228331
C	-4.027568	-0.206028	-0.902591
C	-3.664683	0.124360	1.406218
C	-2.554529	1.157888	1.193113
H	-0.963885	1.220266	-2.920951
H	1.977915	-0.511538	-1.853415
H	-2.643998	-0.454911	-3.006567
H	-4.279533	0.320856	2.291554
O	3.036100	-0.805447	0.553702
C	1.485473	-0.415995	2.739832
H	1.717483	-1.487208	2.650359
H	2.411541	0.077288	3.076298
H	0.715214	-0.286541	3.510501
C	-1.397293	0.618569	2.070574
H	-1.238929	1.196381	2.992609
H	-0.045672	-0.238719	-3.326321
H	-1.595731	-1.674146	-2.290712
O	-4.736459	-0.277474	-1.870455
O	-4.565458	0.039105	0.306438
C	-2.275722	-1.532231	0.164998
H	-1.190681	-1.694401	0.243691

C	-2.794610	-1.129874	1.568379
H	-3.318344	-1.965188	2.051564
H	-2.845183	2.182958	1.447261
H	-2.484008	1.807948	-0.904245
C	-2.932121	-2.836631	-0.287338
H	-4.032066	-2.765395	-0.280155
H	-2.621293	-3.136173	-1.297606
H	-2.652249	-3.648866	0.399864
O	-1.786387	-0.700641	2.452394
O	-0.158283	2.551999	-0.108841
C	-0.245658	3.319707	-1.282412
H	0.010668	4.351681	-1.005507
H	0.464336	2.981434	-2.058482
H	-1.261497	3.326143	-1.715877
C	2.740890	1.614466	0.385981
H	2.456505	2.285213	-0.436012
H	2.540832	2.105891	1.349766
B	3.870645	-1.452532	-0.462594
F	3.187381	-2.487565	-1.110455
F	4.997465	-1.952210	0.189986
F	4.275477	-0.497633	-1.434286
N	5.243309	1.373216	0.345485
N	4.156834	1.515320	0.342543

53

21-ROT_1_4
Eopt -1583.905121

C	2.085112	0.235656	0.123248
C	1.395220	-0.587851	-0.956067
C	0.157821	-0.305488	-1.360394
C	-0.537415	0.898214	-0.773353
C	-0.159134	1.105489	0.674686
C	1.065539	0.810044	1.118533
C	-0.643691	-1.022364	-2.398686
C	-2.073084	0.755921	-0.748869
C	-2.531772	-0.735358	-0.653200
C	-1.959441	-1.571964	-1.813967
C	-4.048080	-0.645598	-0.707860
C	-3.626133	0.673326	1.195790
C	-2.555789	1.518863	0.501495
H	-0.872512	-0.292900	-3.194071
H	1.965902	-1.402827	-1.401130
H	-2.714241	-1.639176	-2.609006
H	-4.215338	1.237876	1.927084
O	3.060490	-0.431532	0.818816
C	1.521509	0.967463	2.533421
H	1.712969	-0.017153	2.984351
H	2.472260	1.521977	2.578570
H	0.773546	1.492140	3.141115
C	-1.373653	1.470687	1.499328
H	-1.243414	2.401426	2.071182
H	-0.063457	-1.833632	-2.859287
H	-1.789302	-2.598648	-1.464621
O	-4.782308	-1.149466	-1.514864
O	-4.558418	0.099080	0.288720
C	-2.220378	-1.340532	0.764112
H	-1.128550	-1.430015	0.863428
C	-2.713599	-0.362993	1.862620
H	-3.197595	-0.905805	2.685120
H	-2.886792	2.538419	0.275686
H	-2.490238	1.174001	-1.676571
C	-2.841431	-2.721651	0.970257
H	-3.942949	-2.679958	0.971250
H	-2.531756	-3.441064	0.199988
H	-2.527631	-3.125294	1.944326
O	-1.700195	0.435068	2.428494
O	-0.095613	1.973831	-1.607253

C	-0.560648	3.264564	-1.308187
H	-0.006868	3.961222	-1.952790
H	-1.636807	3.385727	-1.523350
H	-0.375227	3.546544	-0.255576
C	2.731610	1.542744	-0.591578
H	2.450693	1.647880	-1.648409
H	2.504365	2.475844	-0.054422
B	3.888498	-1.501802	0.256328
F	3.205679	-2.722347	0.222699
F	5.024431	-1.606918	1.058648
F	4.276928	-1.161307	-1.067361
N	5.234287	1.363780	-0.460586
N	4.147155	1.464322	-0.555080

53

21'-ROT-1 Eopt -1583.900499

C	-1.926361	0.490577	0.031239
C	-1.349120	-0.484558	-0.983977
C	-0.197378	-1.134172	-0.808579
C	0.505157	-1.087223	0.518910
C	0.198103	0.166403	1.285521
C	-0.945218	0.845718	1.158216
C	0.599174	-1.839020	-1.861979
C	2.026647	-1.100008	0.387814
C	2.524408	-0.382612	-0.896292
C	1.871600	-1.008073	-2.152674
C	4.028489	-0.588261	-0.847410
C	3.694990	0.528402	1.207536
C	2.548309	-0.386493	1.646552
H	0.879613	-2.838347	-1.493118
H	-1.885531	-0.554901	-1.933772
H	2.612998	-1.637676	-2.663819
H	4.320711	0.875745	2.037237
O	-3.154520	0.168923	0.565628
C	-1.298788	2.008080	2.044893
H	-0.871492	2.956091	1.678974
H	-2.390672	2.119949	2.117835
H	-0.906174	1.857133	3.059720
C	1.431227	0.607642	2.050156
H	1.281861	0.697478	3.135722
H	0.023757	-1.976373	-2.788121
H	1.604429	-0.206238	-2.855614
O	4.723882	-1.124891	-1.667316
O	4.577175	-0.088962	0.275502
C	2.337822	1.173564	-0.787208
H	1.259521	1.386664	-0.822486
C	2.867306	1.662832	0.589850
H	3.425782	2.602251	0.483408
H	2.813950	-1.084845	2.447077
H	2.359106	-2.145840	0.345967
C	3.023372	1.928812	-1.924709
H	4.120354	1.836504	-1.873142
H	2.703790	1.571311	-2.913813
H	2.782970	3.000782	-1.862223
O	1.868018	1.881126	1.560713
O	0.227895	-2.253775	1.294215
C	-1.048187	-2.371286	1.874296
H	-1.135033	-3.408953	2.226463
H	-1.175100	-1.698608	2.740938
H	-1.862449	-2.178327	1.160158
C	-2.057338	1.877781	-0.794361
H	-1.824689	2.767009	-0.191556
H	-1.463179	1.897645	-1.719068
B	-4.121791	-0.722705	-0.081557
F	-5.343106	-0.526500	0.562245
F	-3.743662	-2.069435	0.007008
F	-4.254214	-0.396266	-1.458743

N	-4.449931	2.267928	-1.456084
N	-3.398872	2.103053	-1.195383

53

21'-ROT-2 Eopt -1583.898920

C	-2.037006	0.110199	0.546987
C	-1.475654	0.597855	-0.782188
C	-0.340760	0.140928	-1.312245
C	0.355346	-1.035638	-0.690009
C	0.095880	-1.140616	0.785439
C	-1.025463	-0.703892	1.364459
C	0.419098	0.727698	-2.458997
C	1.875708	-0.963009	-0.815633
C	2.406862	0.497066	-0.799559
C	1.736590	1.331283	-1.917519
C	3.901093	0.323930	-1.010052
C	3.606664	-0.961587	0.949191
C	2.424029	-1.753681	0.384706
H	0.642615	-0.071279	-3.183575
H	-2.039500	1.381594	-1.295914
H	2.449318	1.457275	-2.744065
H	4.243024	-1.545515	1.623442
O	-3.215334	-0.590302	0.461813
C	-1.334003	-0.941682	2.816832
H	-0.919096	-0.153953	3.466685
H	-2.421124	-0.990895	2.979207
H	-0.898453	-1.890976	3.156906
C	1.347600	-1.625304	1.489957
H	1.204938	-2.538901	2.084777
H	-0.159815	1.500424	-2.983726
H	1.521403	2.339091	-1.535164
O	4.576101	0.780906	-1.892833
O	4.466288	-0.445537	-0.061937
C	2.282344	1.132239	0.631999
H	1.213283	1.287059	0.839939
C	2.825683	0.131592	1.689866
H	3.420995	0.652240	2.451643
H	2.661084	-2.792499	0.131887
H	2.165074	-1.420463	-1.771040
C	3.002805	2.474904	0.745214
H	4.094810	2.361175	0.649878
H	2.673158	3.191215	-0.020501
H	2.806732	2.924002	1.730458
O	1.832444	-0.602784	2.368816
O	0.013299	-2.242967	-1.371228
C	-1.305779	-2.719015	-1.256672
H	-1.380825	-3.580889	-1.934260
H	-1.541054	-3.057434	-0.232890
H	-2.059217	-1.971615	-1.551691
C	-2.350643	1.419066	1.463027
H	-3.432115	1.507553	1.632893
H	-1.811910	1.432392	2.420453
B	-4.286055	-0.161525	-0.436258
F	-5.469505	-0.752061	0.002397
F	-4.028700	-0.536442	-1.764983
F	-4.411644	1.256365	-0.394036
N	-1.682464	3.587707	0.383624
N	-1.983128	2.644967	0.857760

53

21'_A_1 Eopt -1583.900637

C	-1.910085	0.098877	0.430944
C	-1.010323	0.954352	1.324320
C	0.178153	1.423059	0.945492
C	0.654283	1.202183	-0.463952
C	0.066601	-0.042405	-1.072302
C	-1.123932	-0.545093	-0.727976
C	1.199414	2.091231	1.811025

C	2.168490	1.000323	-0.546326
C	2.759317	0.322615	0.721026
C	2.401582	1.134465	1.988259
C	4.252521	0.301626	0.441459
C	3.465175	-0.912972	-1.423432
C	2.397883	0.122531	-1.787549
H	1.529256	3.019837	1.318564
H	-1.397577	1.171933	2.325592
H	3.283383	1.708523	2.304314
H	3.904660	-1.413369	-2.293420
O	-2.974447	0.860264	-0.000862
C	-1.700723	-1.757625	-1.399823
H	-1.563157	-2.669510	-0.794605
H	-2.773441	-1.616638	-1.592835
H	-1.208459	-1.949865	-2.361537
C	1.112728	-0.725894	-1.934111
H	0.801178	-0.883150	-2.976624
H	0.789766	2.364053	2.793529
H	2.171223	0.440071	2.808229
O	5.129438	0.793727	1.099427
O	4.553379	-0.359297	-0.690916
C	2.351496	-1.192239	0.795755
H	1.271593	-1.239268	0.998489
C	2.597294	-1.860507	-0.585473
H	3.035711	-2.859949	-0.464289
H	2.631072	0.710268	-2.681693
H	2.637074	1.987648	-0.653893
C	3.090154	-1.950307	1.897454
H	4.170867	-2.018806	1.692416
H	2.965110	-1.480483	2.883203
H	2.705371	-2.978875	1.966828
O	1.441732	-2.005329	-1.379182
O	0.428260	2.352346	-1.277946
C	-0.899029	2.735594	-1.533548
H	-0.844690	3.627519	-2.173175
H	-1.469473	1.954165	-2.062888
H	-1.450738	2.990795	-0.613739
C	-2.306706	-1.028991	1.466439
H	-1.430562	-1.638618	1.736373
H	-2.769768	-0.589801	2.360601
B	-4.380559	0.515367	-0.042439
F	-5.088070	1.635081	-0.452269
F	-4.822916	0.098454	1.243951
F	-4.630145	-0.565953	-0.925454
N	-4.042096	-2.737662	0.784064
N	-3.287627	-1.988080	1.045429

53

21'_A_2 Eopt -1583.895555

C	-1.984438	0.029309	0.596243
C	-1.027801	0.838970	1.474952
C	0.129518	1.305400	1.010515
C	0.501760	1.009703	-0.423142
C	-0.034117	-0.326899	-0.869930
C	-1.191957	-0.822836	-0.424834
C	1.182717	2.020875	1.801569
C	2.022817	0.894483	-0.609960
C	2.764277	0.323555	0.639643
C	2.452324	1.150736	1.905899
C	4.224448	0.403298	0.228647
C	3.382305	-0.981077	-1.488118
C	2.205774	-0.053919	-1.805686
H	1.431380	2.968366	1.299274
H	-1.350937	1.065650	2.495946
H	3.315471	1.788795	2.140189
H	3.784163	-1.495162	-2.368261
O	-2.855723	0.919835	0.002789

C	-1.713127	-2.155474	-0.879614
H	-1.724418	-2.892441	-0.057974
H	-2.731411	-2.060362	-1.281206
H	-1.076436	-2.581423	-1.664205
C	0.983564	-1.003980	-1.767640
H	0.582939	-1.243219	-2.762965
H	0.819834	2.274550	2.807575
H	2.331596	0.467203	2.757359
O	5.109275	1.006944	0.773972
O	4.481501	-0.302601	-0.887418
C	2.495115	-1.212087	0.830422
H	1.446002	-1.334968	1.137513
C	2.673435	-1.942980	-0.525126
H	3.201410	-2.896761	-0.393494
H	2.303180	0.481036	-2.756276
H	2.440827	1.890960	-0.812619
C	3.394680	-1.840282	1.894656
H	4.453024	-1.838600	1.587037
H	3.326076	-1.323099	2.861877
H	3.103666	-2.889014	2.056321
O	1.464944	-2.221531	-1.191059
O	-0.024900	1.945364	-1.356308
C	0.063018	3.308005	-1.043738
H	-0.343005	3.857061	-1.904557
H	-0.537791	3.570793	-0.155003
H	1.103465	3.647706	-0.882916
C	-2.678775	-0.881151	1.677961
H	-1.954138	-1.558107	2.155530
H	-3.187568	-0.263981	2.432617
B	-4.221436	0.660327	-0.394816
F	-4.700225	1.770883	-1.073227
F	-5.022128	0.415111	0.759208
F	-4.325588	-0.494816	-1.208310
N	-4.520559	-2.435840	0.915279
N	-3.718395	-1.753190	1.214885

53

21'_A_3 Eopt -1583.908367

C	-1.950224	0.315804	-0.241495
C	-1.260566	-0.857376	-0.928782
C	-0.102318	-1.367727	-0.509482
C	0.508986	-0.882021	0.775588
C	0.139422	0.545271	1.078291
C	-1.004507	1.105246	0.674871
C	0.762275	-2.348683	-1.236720
C	2.037543	-0.888209	0.741705
C	2.614773	-0.616260	-0.675201
C	2.054576	-1.634578	-1.695960
C	4.113603	-0.753454	-0.469182
C	3.637842	0.966682	1.076950
C	2.472105	0.212857	1.722974
H	1.014306	-3.173971	-0.551707
H	-1.742715	-1.249404	-1.828706
H	2.828538	-2.382965	-1.914866
H	4.204127	1.584974	1.782428
O	-3.099915	0.019730	0.462071
C	-1.416231	2.494709	1.066374
H	-1.379555	3.200619	0.219422
H	-2.443091	2.488992	1.465319
H	-0.759208	2.903144	1.843926
C	1.324941	1.251503	1.710160
H	1.112066	1.689260	2.695843
H	0.246399	-2.783914	-2.103869
H	1.844459	-1.116745	-2.642416
O	4.864768	-1.511973	-1.020842
O	4.584367	0.103501	0.455309
C	2.411346	0.882961	-1.096488

H	1.336681	1.041928	-1.266798
C	2.843926	1.811016	0.071597
H	3.400776	2.679743	-0.303732
H	2.690242	-0.176382	2.723061
H	2.381417	-1.880047	1.064426
C	3.166976	1.242788	-2.374743
H	4.258711	1.202941	-2.229211
H	2.915645	0.572763	-3.208935
H	2.915844	2.268972	-2.682319
O	1.779434	2.304584	0.852818
O	0.195393	-1.752564	1.861941
C	-1.143475	-1.830726	2.285997
H	-1.172852	-2.600532	3.069711
H	-1.503311	-0.879614	2.714305
H	-1.831384	-2.121606	1.476720
C	-2.327225	1.250930	-1.481458
H	-1.524847	1.931666	-1.800125
H	-2.704285	0.661691	-2.328574
B	-4.132977	-0.876395	-0.053014
F	-5.250553	-0.731039	0.766409
F	-3.715252	-2.215077	-0.066863
F	-4.469541	-0.500588	-1.384634
N	-4.330483	2.622538	-0.814446
N	-3.440940	2.067487	-1.131754

53

21'_A_4 Eopt -1583.902262

C	2.024180	0.407262	0.263372
C	1.322214	-0.712773	1.023499
C	0.157873	-1.227633	0.630663
C	-0.419059	-0.780677	-0.686247
C	-0.092534	0.657999	-0.989347
C	1.057637	1.218655	-0.612668
C	-0.709059	-2.172766	1.404596
C	-1.951392	-0.842214	-0.709837
C	-2.594084	-0.539332	0.679434
C	-2.041915	-1.484607	1.769849
C	-4.076040	-0.742410	0.417130
C	-3.601040	0.949927	-1.159836
C	-2.378378	0.225459	-1.731153
H	-0.915548	-3.061877	0.789124
H	1.799867	-1.066038	1.941623
H	-2.797430	-2.249348	1.996545
H	-4.159593	1.522184	-1.908899
O	3.101896	0.033818	-0.513584
C	1.484929	2.589212	-1.048606
H	1.590476	3.294093	-0.207071
H	2.455423	2.531307	-1.568167
H	0.760793	3.033378	-1.742404
C	-1.271027	1.308070	-1.685971
H	-1.019875	1.722250	-2.672612
H	-0.204291	-2.520087	2.316949
H	-1.891062	-0.912564	2.695539
O	-4.819609	-1.515855	0.958744
O	-4.539618	0.067733	-0.552067
C	-2.468819	0.979660	1.061515
H	-1.409728	1.188617	1.272008
C	-2.888013	1.856470	-0.147731
H	-3.496003	2.711270	0.176864
H	-2.532778	-0.188052	-2.733380
H	-2.276496	-1.848216	-1.011214
C	-3.293086	1.346132	2.295399
H	-4.375043	1.255983	2.105908
H	-3.050955	0.717207	3.163378
H	-3.097433	2.391769	2.576568
O	-1.808263	2.370033	-0.892272
O	0.115210	-1.497685	-1.795386

C	0.207896	-2.893821	-1.692930
H	0.585831	-3.255865	-2.659065
H	0.918742	-3.204976	-0.908345
H	-0.768666	-3.378380	-1.506711
C	2.542591	1.344085	1.446225
H	1.803136	2.068955	1.817240
H	2.964299	0.753068	2.270770
B	4.073589	-0.961350	-0.066655
F	5.166182	-0.887944	-0.929058
F	3.540717	-2.257512	-0.072342
F	4.496998	-0.653747	1.258072
N	4.546235	2.603540	0.587755
N	3.659354	2.097941	0.985240

53

21'_B_1 Eopt -1583.906410

C	1.941116	0.530549	-0.228168
C	1.061369	1.533432	0.505385
C	-0.071829	1.199392	1.120973
C	-0.503204	-0.243532	1.157728
C	0.013645	-1.013389	-0.029929
C	1.147685	-0.717099	-0.674734
C	-1.078165	2.130442	1.720582
C	-2.025316	-0.395861	1.083504
C	-2.719031	0.734704	0.273124
C	-2.355325	2.122176	0.850455
C	-4.195900	0.408961	0.420004
C	-3.426161	-1.592336	-0.566529
C	-2.268616	-1.761492	0.420971
H	-1.316732	1.782370	2.738345
H	1.405671	2.574293	0.521447
H	-3.203765	2.497249	1.439159
H	-3.864698	-2.541459	-0.893935
O	3.060483	0.304170	0.549100
C	1.606088	-1.500875	-1.874498
H	1.259098	-1.031980	-2.810241
H	2.699786	-1.580069	-1.913404
H	1.197047	-2.519066	-1.855773
C	-1.046480	-1.993354	-0.498003
H	-0.701617	-3.036931	-0.524109
H	-0.688494	3.154605	1.804582
H	-2.212927	2.830823	0.022608
O	-5.058067	1.105752	0.884022
O	-4.499378	-0.817722	-0.040941
C	-2.436237	0.591777	-1.264740
H	-1.374362	0.822101	-1.434628
C	-2.669951	-0.881663	-1.696574
H	-3.186893	-0.929456	-2.664200
H	-2.416737	-2.563535	1.151739
H	-2.416298	-0.365515	2.109296
C	-3.285470	1.538345	-2.111580
H	-4.356074	1.281404	-2.061581
H	-3.178331	2.586991	-1.800547
H	-2.982376	1.470041	-3.167131
O	-1.494211	-1.648743	-1.814065
O	-0.169647	-0.866962	2.395062
C	1.190931	-1.021146	2.715042
H	1.222833	-1.569275	3.667009
H	1.741764	-1.595752	1.953668
H	1.703878	-0.053652	2.845222
C	2.403986	1.238635	-1.570876
H	2.945937	0.562438	-2.245157
H	1.602990	1.777000	-2.098477
B	4.106413	-0.669328	0.261782
F	5.134022	-0.447377	1.176071
F	4.604164	-0.472948	-1.060008
F	3.644208	-1.990100	0.368451

N 4.150133 2.919980 -0.886308
N 3.383358 2.212860 -1.221077
53
21'_B_2 Eopt -1583.900233
C 2.015520 -0.271610 0.545886
C 1.129689 -1.509987 0.572089
C -0.008890 -1.581458 -0.115056
C -0.410663 -0.395691 -0.960990
C 0.048403 0.904944 -0.353648
C 1.186308 1.014616 0.335473
C -1.016270 -2.690011 -0.064625
C -1.937042 -0.249616 -1.065632
C -2.692866 -0.729159 0.213366
C -2.329789 -2.187303 0.569237
C -4.152223 -0.565642 -0.175640
C -3.397497 1.654137 -0.445799
C -2.180575 1.253146 -1.284543
H -1.218165 -3.041041 -1.088525
H 1.471250 -2.365115 1.166620
H -3.156301 -2.846150 0.269799
H -3.833271 2.615517 -0.739542
O 3.014713 -0.491297 -0.382928
C 1.655486 2.316002 0.921538
H 1.442975 2.368956 2.002687
H 2.736186 2.452176 0.778952
H 1.144726 3.165651 0.451245
C -1.007108 1.969020 -0.571236
H -0.625995 2.843439 -1.117777
H -0.632661 -3.550418 0.501727
H -2.242016 -2.276458 1.660501
O -5.000600 -1.416097 -0.218185
O -4.455740 0.702549 -0.505417
C -2.492308 0.262275 1.414996
H -1.444999 0.186080 1.742855
C -2.725138 1.717609 0.932187
H -3.298922 2.291720 1.671820
H -2.262502 1.516397 -2.344396
H -2.306834 -0.848951 -1.909725
C -3.401704 -0.056373 2.601144
H -4.464481 0.099818 2.354389
H -3.287874 -1.091401 2.952352
H -3.161133 0.609210 3.443616
O -1.541670 2.433769 0.671556
O 0.165435 -0.406280 -2.262128
C 0.132602 -1.603950 -2.988593
H 0.570401 -1.388584 -3.973079
H 0.735333 -2.397106 -2.511271
H -0.893544 -1.984720 -3.148073
C 2.670307 -0.151045 1.982958
H 3.227876 0.783610 2.125905
H 1.972236 -0.336882 2.812511
B 4.036103 0.481326 -0.752241
F 4.969463 -0.186334 -1.543890
F 4.686840 0.967610 0.423032
F 3.509430 1.576810 -1.445649
N 4.450048 -1.931354 2.064243
N 3.675814 -1.156606 2.072233
53
21'_B_3 Eopt -1583.905287
C 1.952092 -0.060884 -0.379580
C 1.330129 0.811825 0.705623
C 0.160062 0.538954 1.284509
C -0.541765 -0.755349 0.985033
C -0.218725 -1.271989 -0.390401
C 0.934785 -1.024555 -1.016775
C -0.636273 1.446169 2.168800

C	-2.064898	-0.620146	0.999219
C	-2.549559	0.783705	0.541591
C	-1.907992	1.891250	1.410386
C	-4.057114	0.703560	0.711591
C	-3.712514	-1.090539	-0.784031
C	-2.581996	-1.708450	0.043418
H	-0.918124	0.896136	3.080887
H	1.866114	1.720319	0.992775
H	-2.653756	2.258825	2.128551
H	-4.334007	-1.831966	-1.298327
O	3.049843	-0.793792	0.011198
C	1.297057	-1.630595	-2.342209
H	1.132850	-0.932471	-3.179196
H	2.358528	-1.922543	-2.344864
H	0.696434	-2.525864	-2.547694
C	-1.452605	-1.914903	-0.994645
H	-1.310613	-2.962480	-1.296307
H	-0.057339	2.327359	2.478725
H	-1.645709	2.744494	0.769158
O	-4.757966	1.406064	1.389303
O	-4.601840	-0.294678	-0.007308
C	-2.339392	0.984296	-1.002073
H	-1.257792	1.055136	-1.188216
C	-2.865308	-0.265356	-1.761434
H	-3.408731	0.027967	-2.669401
H	-2.861652	-2.629363	0.565916
H	-2.413548	-0.782164	2.027936
C	-3.011137	2.251659	-1.528257
H	-4.109613	2.186895	-1.466887
H	-2.697549	3.149429	-0.977146
H	-2.752217	2.401105	-2.587201
O	-1.865455	-1.178854	-2.151563
O	-0.281227	-1.737741	1.987254

C	1.044902	-2.167120	2.166174
H	1.016792	-2.936690	2.950028
H	1.474134	-2.608490	1.251432
H	1.712164	-1.352438	2.494850
C	2.309217	0.913557	-1.583911
H	2.936079	0.426769	-2.343453
H	1.404124	1.351531	-2.032171
B	4.329275	-0.204173	0.366168
F	5.124748	-1.189498	0.930542
F	4.172785	0.882196	1.253753
F	4.953463	0.309842	-0.808109
N	3.643272	2.930566	-0.862438
N	3.076363	2.046288	-1.174011

53

21'_C_1 Eopt -1583.906619

C	2.048497	0.415319	0.648304
C	1.099422	0.037375	1.776515
C	0.003809	-0.699613	1.595058
C	-0.304533	-1.254481	0.228347
C	0.260607	-0.389738	-0.868884
C	1.360201	0.359819	-0.731578
C	-1.066493	-0.986127	2.600157
C	-1.809369	-1.303574	-0.053180
C	-2.607202	-0.183292	0.671409
C	-2.352957	-0.230789	2.195933
C	-4.049872	-0.491894	0.308160
C	-3.126952	-0.227978	-1.848432
C	-1.946179	-1.164058	-1.578003
H	-1.257002	-2.071184	2.609585
H	1.371799	0.394708	2.775729
H	-3.219744	-0.692827	2.687977
H	-3.488545	-0.266818	-2.881929
O	3.207798	-0.311655	0.789418

C	1.868579	1.234702	-1.846213
H	1.446867	2.252022	-1.781584
H	2.961647	1.317143	-1.832641
H	1.571136	0.834297	-2.823862
C	-0.721267	-0.332018	-2.024470
H	-0.291275	-0.652916	-2.984049
H	-0.763390	-0.694594	3.615424
H	-2.285491	0.795393	2.583486
O	-4.962115	-0.718467	1.056821
O	-4.254392	-0.497112	-1.021180
C	-2.329274	1.218898	0.023788
H	-1.288939	1.487719	0.252053
C	-2.448005	1.107865	-1.520695
H	-2.972600	1.977664	-1.937863
H	-2.017268	-2.132313	-2.084590
H	-2.186533	-2.271670	0.302204
C	-3.246208	2.317366	0.557565
H	-4.295120	2.151822	0.262675
H	-3.214799	2.393262	1.653498
H	-2.940621	3.291284	0.146506
O	-1.213786	1.002951	-2.191792
O	0.099238	-2.615892	0.111856
C	1.473970	-2.907422	0.175477
H	1.565237	-3.992069	0.024582
H	2.052585	-2.388698	-0.604945
H	1.913849	-2.647425	1.152837
C	2.498144	1.915617	0.938555
H	2.940083	1.991749	1.943457
H	3.197495	2.301739	0.183467
B	4.301639	-0.374075	-0.174910
F	5.332696	-1.092351	0.426498
F	4.763620	0.935128	-0.490866
F	3.912160	-1.001928	-1.369828

N	0.470855	3.407305	0.923151
N	1.382387	2.796978	0.944337

53

21'_C_2 Eopt -1583.899810

C	-2.125996	-0.325219	0.687086
C	-1.164956	0.151708	1.765750
C	-0.048616	0.823773	1.488708
C	0.242504	1.174862	0.048998
C	-0.292922	0.136280	-0.900944
C	-1.410788	-0.552923	-0.661858
C	1.035927	1.186615	2.456334
C	1.750810	1.215649	-0.241918
C	2.566970	0.193622	0.610670
C	2.317941	0.395660	2.121688
C	4.002480	0.485621	0.208196
C	3.083113	-0.061100	-1.895502
C	1.879762	0.872478	-1.735352
H	1.244104	2.265105	2.385842
H	-1.437470	-0.073736	2.802938
H	3.186725	0.901858	2.564165
H	3.441712	-0.138605	-2.927858
O	-3.210858	0.518016	0.666149
C	-1.943392	-1.572411	-1.631784
H	-1.614056	-2.593115	-1.371415
H	-3.039704	-1.567339	-1.661557
H	-1.575942	-1.374252	-2.646797
C	0.673910	-0.046508	-2.052038
H	0.214397	0.148441	-3.031535
H	0.730201	0.982801	3.492154
H	2.251047	-0.587819	2.606663
O	4.909541	0.824762	0.920029
O	4.205724	0.332517	-1.112351
C	2.320615	-1.284815	0.139885

H	1.287647	-1.554391	0.405871
C	2.438960	-1.364594	-1.405026
H	2.987187	-2.264740	-1.713059
H	1.918090	1.763576	-2.370740
H	2.143876	2.217415	-0.017603
C	3.272454	-2.282340	0.798701
H	4.315626	-2.120052	0.482369
H	3.243390	-2.230432	1.896032
H	2.998289	-3.307042	0.506088
O	1.202346	-1.375746	-2.076830
O	-0.372177	2.388623	-0.368205
C	-0.243525	3.512161	0.460668
H	-0.724200	4.348839	-0.064580
H	-0.755822	3.375818	1.429773
H	0.809941	3.789178	0.650965
C	-2.706707	-1.714989	1.204337
H	-3.189702	-1.582919	2.183079
H	-3.401691	-2.181722	0.492985
B	-4.257171	0.513543	-0.352427
F	-5.242017	1.402613	0.073940
F	-4.825270	-0.788861	-0.472271
F	-3.769547	0.898776	-1.607530
N	-0.773426	-3.291182	1.539538
N	-1.652884	-2.646514	1.416346

53

21'_C_3 Eopt -1583.901466

C	-2.166311	0.529580	0.175342
C	-1.550438	0.031102	-1.125423
C	-0.418207	-0.669112	-1.170598
C	0.192532	-1.153013	0.118413
C	-0.057246	-0.191711	1.256268
C	-1.166034	0.549512	1.338136
C	0.353366	-1.078870	-2.382205
C	1.728074	-1.274141	0.050627
C	2.355921	-0.257461	-0.952509
C	1.756561	-0.434914	-2.364210
C	3.840089	-0.580078	-0.911761
C	3.467088	-0.072323	1.359154
C	2.243580	-0.984814	1.473840
H	0.449029	-2.176688	-2.361686
H	-2.072226	0.302769	-2.047508
H	2.444984	-1.042903	-2.966543
H	4.062436	-0.028568	2.277869
O	-3.328149	-0.079349	0.583210
C	-1.482341	1.425107	2.516171
H	-1.276370	2.491273	2.323913
H	-2.548001	1.329781	2.776886
H	-0.889085	1.140426	3.394397
C	1.179192	-0.067096	2.122907
H	0.997902	-0.282743	3.186041
H	-0.174893	-0.813181	-3.308156
H	1.700021	0.544726	-2.857496
O	4.542319	-0.912371	-1.828468
O	4.359957	-0.461444	0.322789
C	2.253824	1.210781	-0.401160
H	1.190967	1.487226	-0.393152
C	2.748176	1.249520	1.070418
H	3.370371	2.135395	1.253774
H	2.433997	-1.896208	2.050414
H	1.993942	-2.286201	-0.287311
C	3.014580	2.227795	-1.249287
H	4.101503	2.046612	-1.227658
H	2.694150	2.222314	-2.300190
H	2.844031	3.240973	-0.855323
O	1.715824	1.257543	2.030126
O	-0.433245	-2.413282	0.322093

C	-0.118444	-3.126820	1.487286
H	-0.795197	-3.991634	1.521359
H	0.919160	-3.506964	1.488895
H	-0.281297	-2.531658	2.404316
C	-2.613700	2.035828	-0.128865
H	-3.317768	2.035829	-0.975007
H	-3.055299	2.542192	0.740425
B	-4.346337	-0.559410	-0.354248
F	-5.515124	-0.760145	0.379430
F	-3.955789	-1.749161	-0.976547
F	-4.588444	0.415275	-1.364718
N	-0.598240	3.362543	-0.833890
N	-1.507715	2.828679	-0.529506

53

21'_C_4 Eopt -1583.902703

C	2.152510	0.647724	-0.029695
C	1.556322	-0.245247	1.051413
C	0.440884	-0.951478	0.867685
C	-0.171101	-0.984831	-0.508920
C	0.028953	0.310764	-1.249931
C	1.123713	1.057107	-1.094029
C	-0.345776	-1.673477	1.918280
C	-1.692425	-1.176945	-0.473510
C	-2.361887	-0.507925	0.767024
C	-1.730637	-1.012123	2.084293
C	-3.820310	-0.904963	0.618827
C	-3.489771	0.239965	-1.420353
C	-2.206970	-0.521649	-1.766419
H	-0.477981	-2.723338	1.614434
H	2.063708	-0.251526	2.020799
H	-2.418985	-1.721684	2.563415
H	-4.094023	0.499852	-2.296601
O	3.287533	0.185216	-0.652636
C	1.421404	2.268241	-1.929485
H	1.244639	3.213864	-1.389711
H	2.475940	2.255559	-2.246819
H	0.794682	2.294195	-2.829987
C	-1.197570	0.609718	-2.086901
H	-0.976520	0.714514	-3.158584
H	0.185111	-1.678741	2.880531
H	-1.625609	-0.165687	2.776883
O	-4.495218	-1.526663	1.394710
O	-4.349612	-0.482119	-0.543854
C	-2.370542	1.058342	0.651353
H	-1.333020	1.407276	0.754179
C	-2.861742	1.474569	-0.759976
H	-3.543158	2.333722	-0.703173
H	-2.321058	-1.239038	-2.585878
H	-1.929498	-2.249800	-0.436465
C	-3.222510	1.725514	1.730218
H	-4.292672	1.493846	1.604768
H	-2.930741	1.421062	2.745001
H	-3.115560	2.818736	1.666254
O	-1.827632	1.818140	-1.652069
O	0.424062	-1.960414	-1.358477
C	0.626164	-3.248486	-0.840020
H	1.040326	-3.855251	-1.656947
H	1.351493	-3.251834	-0.008779
H	-0.311148	-3.727033	-0.500769
C	2.646933	1.961396	0.731279
H	3.373142	1.681181	1.509065
H	3.072696	2.714299	0.053796
B	4.296962	-0.614074	0.043850
F	5.440265	-0.617380	-0.753269
F	3.858620	-1.928501	0.260748
F	4.602366	-0.034158	1.307382

N	0.675600	3.012923	1.885926
N	1.568008	2.597928	1.400274

53

21_A_1 Eopt -1583.867875

C	2.052603	-0.204061	0.054666
C	1.236360	-1.163230	-0.807694
C	0.038072	-0.824960	-1.281308
C	-0.511956	0.551246	-0.977552
C	-0.053839	1.054173	0.373356
C	1.136043	0.712267	0.881425
C	-0.875749	-1.667646	-2.112916
C	-2.052937	0.559425	-0.880789
C	-2.634478	-0.816192	-0.423009
C	-2.196582	-1.949060	-1.371776
C	-4.143536	-0.601853	-0.463987
C	-3.524232	1.075345	1.062899
C	-2.416327	1.634464	0.163441
H	-1.081088	-1.112006	-3.043417
H	1.726212	-2.112911	-1.028971
H	-3.003551	-2.134801	-2.093470
H	-4.023322	1.850069	1.656596
O	2.940336	-0.817278	0.867037
C	1.596579	1.047646	2.265262
H	1.571726	0.137138	2.881698
H	2.645538	1.376752	2.276651
H	0.959289	1.805635	2.736530
C	-1.198269	1.711723	1.115934
H	-0.963620	2.735039	1.448271
H	-0.391258	-2.609888	-2.402347
H	-2.086083	-2.876304	-0.794831
O	-4.942459	-1.208505	-1.115168
O	-4.541649	0.402405	0.347053
C	-2.305467	-1.099375	1.086928
H	-1.220418	-1.258502	1.178389
C	-2.666858	0.148779	1.934602
H	-3.153172	-0.140270	2.875494
H	-2.676154	2.598857	-0.288383
H	-2.475836	0.781497	-1.872187
C	-3.029264	-2.331630	1.630542
H	-4.122347	-2.194320	1.631428
H	-2.808087	-3.238707	1.053086
H	-2.715667	-2.517619	2.668015
O	-1.569850	0.964287	2.270336
O	-0.035149	1.368631	-2.060831
C	-0.416026	2.717653	-2.072648
H	0.136156	3.200851	-2.889909
H	-1.493891	2.849212	-2.270404
H	-0.165706	3.231211	-1.125307
C	2.784915	0.684117	-1.070694
H	3.492132	0.046054	-1.622423
H	2.032341	1.181122	-1.706556
B	4.250633	-1.304289	0.361789
F	4.072752	-1.884378	-0.915095
F	4.785716	-2.166615	1.267993
F	5.068815	-0.149839	0.174282
N	4.084404	2.519231	0.054903
N	3.557590	1.712412	-0.473334

53

21_A_2 Eopt -1583.868154

C	-2.053091	0.028921	0.194409
C	-1.269286	1.331550	0.346871
C	-0.062644	1.488871	-0.199747
C	0.501580	0.366001	-1.042167
C	0.071507	-0.990533	-0.537798
C	-1.104574	-1.174969	0.068092
C	0.874528	2.638267	0.028452

C	2.035230	0.308957	-0.984682
C	2.618957	0.757148	0.394041
C	2.127025	2.165584	0.791147
C	4.122641	0.713732	0.149276
C	3.553268	-1.529143	-0.304443
C	2.398964	-1.161791	-1.244923
H	1.177879	3.063991	-0.941627
H	-1.763963	2.095788	0.948634
H	2.947260	2.882794	0.649518
H	4.076182	-2.446097	-0.599715
O	-2.946918	-0.180672	1.185442
C	-1.544545	-2.453021	0.710838
H	-1.582661	-2.308640	1.800616
H	-2.568419	-2.730139	0.421095
H	-0.856900	-3.277902	0.489981
C	1.209378	-1.980533	-0.684561
H	0.935678	-2.842773	-1.310460
H	0.377328	3.444087	0.585864
H	1.891591	2.170250	1.863665
O	4.898384	1.620379	0.234233
O	4.543194	-0.519136	-0.209607
C	2.366942	-0.310121	1.516910
H	1.290304	-0.321683	1.744152
C	2.747043	-1.716906	0.989661
H	3.277808	-2.297463	1.755627
H	2.602101	-1.362898	-2.302865
H	2.466381	0.972378	-1.748090
C	3.137749	-0.001051	2.801254
H	4.226426	-0.070410	2.648031
H	2.920886	1.002180	3.191399
H	2.864391	-0.725879	3.581786
O	1.649623	-2.489352	0.569724
O	0.050845	0.426566	-2.409287

C	0.219454	1.637802	-3.100964
H	-0.105193	1.461451	-4.134969
H	-0.392597	2.450254	-2.669806
H	1.272466	1.968285	-3.127359
C	-2.775925	0.221509	-1.230146
H	-3.486216	1.059482	-1.168541
H	-2.014986	0.308749	-2.024211
B	-4.258283	0.520439	1.198917
F	-4.078869	1.855746	0.771409
F	-4.801244	0.428529	2.443188
F	-5.070777	-0.115458	0.212746
N	-4.070356	-1.863177	-1.787545
N	-3.545724	-0.925573	-1.559106

53

21_A_3 Eopt -1583.913188

C	-2.057179	-0.040757	-0.108006
C	-1.266964	-1.345672	-0.169610
C	-0.048893	-1.453521	0.363434
C	0.527735	-0.259688	1.092514
C	0.082499	1.042413	0.471334
C	-1.113281	1.174811	-0.108296
C	0.879960	-2.623172	0.235261
C	2.060976	-0.206721	1.008611
C	2.628297	-0.792133	-0.326001
C	2.134805	-2.234098	-0.570091
C	4.130686	-0.713837	-0.109996
C	3.556404	1.562626	0.124834
C	2.421287	1.284449	1.114650
H	1.182277	-2.952457	1.241876
H	-1.759713	-2.168490	-0.690403
H	2.948384	-2.936086	-0.341803
H	4.082478	2.503934	0.318671
O	-2.950898	0.082173	-1.138995

C	-1.569990	2.405134	-0.829858
H	-1.552303	2.224234	-1.915671
H	-2.608328	2.670680	-0.583994
H	-0.919322	3.261956	-0.613778
C	1.220748	2.041455	0.496018
H	0.958701	2.963788	1.034444
H	0.376490	-3.473515	-0.245217
H	1.907767	-2.356822	-1.637366
O	4.921668	-1.618517	-0.116804
O	4.551297	0.543950	0.116722
C	2.358407	0.156232	-1.549125
H	1.279169	0.136074	-1.760748
C	2.735947	1.610633	-1.171591
H	3.255022	2.112976	-1.998401
H	2.640704	1.592492	2.142664
H	2.499461	-0.789995	1.830142
C	3.111121	-0.275771	-2.807327
H	4.201942	-0.173284	-2.686951
H	2.900115	-1.316773	-3.087838
H	2.814127	0.362312	-3.652871
O	1.635927	2.414754	-0.817481
O	0.095780	-0.191255	2.459361
C	0.260573	-1.335781	3.262625
H	-0.039388	-1.052081	4.280655
H	-0.378729	-2.172867	2.930748
H	1.307781	-1.682001	3.299427
C	-2.774755	-0.111304	1.302593
H	-3.454666	-0.971169	1.382779
H	-2.027609	-0.092413	2.115108
B	-4.226196	-0.619596	-1.194412
F	-4.726681	-0.507864	-2.484861
F	-5.131687	-0.021666	-0.273321
F	-4.096582	-1.975528	-0.828182

N	-4.199360	1.936325	1.690682
N	-3.599114	1.034016	1.528404

53

21_A_4 Eopt -1583.913506

C	2.057929	-0.166520	-0.005906
C	1.239777	-1.098975	-0.894356
C	0.026211	-0.762167	-1.329681
C	-0.530813	0.595807	-0.963182
C	-0.060717	1.037795	0.405409
C	1.142453	0.696023	0.878679
C	-0.889214	-1.580664	-2.182242
C	-2.070623	0.595430	-0.852604
C	-2.645820	-0.801604	-0.451722
C	-2.207171	-1.892710	-1.447735
C	-4.150381	-0.584422	-0.471364
C	-3.522724	1.029461	1.122718
C	-2.423537	1.624507	0.239672
H	-1.104008	-0.991906	-3.089859
H	1.728542	-2.035474	-1.169348
H	-3.008844	-2.046088	-2.182565
H	-4.023869	1.775745	1.749355
O	2.942926	-0.842210	0.792646
C	1.621464	1.003574	2.263652
H	1.597362	0.090277	2.877295
H	2.666255	1.347391	2.269446
H	0.991535	1.760327	2.747990
C	-1.199199	1.660648	1.184878
H	-0.962871	2.667693	1.559554
H	-0.404216	-2.511688	-2.505786
H	-2.095108	-2.844151	-0.911677
O	-4.963868	-1.164190	-1.139221
O	-4.545190	0.380456	0.377679
C	-2.315063	-1.151092	1.044984

H	-1.231212	-1.321308	1.123308
C	-2.666530	0.060690	1.946996
H	-3.149955	-0.266683	2.876797
H	-2.680822	2.607554	-0.169400
H	-2.501350	0.862293	-1.828822
C	-3.040359	-2.402814	1.537961
H	-4.132791	-2.258626	1.567165
H	-2.833314	-3.283031	0.914345
H	-2.713830	-2.638960	2.561593
O	-1.561045	0.855537	2.308679
O	-0.064083	1.455000	-2.010467
C	-0.429480	2.812197	-1.953464
H	0.134616	3.326937	-2.743327
H	-1.504511	2.965891	-2.148637
H	-0.171981	3.275634	-0.983888
C	2.783528	0.764850	-1.060135
H	3.487224	0.201468	-1.689863
H	2.038646	1.309636	-1.665848
B	4.233011	-1.330555	0.330224
F	4.722047	-2.231130	1.266429
F	5.129363	-0.233122	0.200060
F	4.134565	-1.940471	-0.940283
N	4.147402	2.543538	0.099576
N	3.573650	1.768681	-0.421670

53

21_A_5 Eopt -1583.911873

C	2.252355	0.359535	-0.256046
C	1.387835	0.236818	-1.502607
C	0.126813	0.667347	-1.527033
C	-0.435606	1.328620	-0.288524
C	0.154367	0.758032	0.976754
C	1.405548	0.297238	1.029286
C	-0.861853	0.450236	-2.632022
C	-1.945239	1.080744	-0.136309
C	-2.393576	-0.313236	-0.687879
C	-1.972573	-0.506796	-2.160555
C	-3.902050	-0.272478	-0.510665
C	-3.212063	0.036150	1.723236
C	-2.217916	1.148675	1.375900
H	-1.307772	1.415376	-2.918737
H	1.862852	-0.242784	-2.361724
H	-2.854286	-0.394223	-2.806142
H	-3.694348	0.171966	2.697638
O	3.320675	-0.507340	-0.232200
C	2.008622	-0.352684	2.236806
H	1.932020	-1.445664	2.135174
H	3.078275	-0.127142	2.343994
H	1.485026	-0.058772	3.155905
C	-0.908297	0.671446	2.051004
H	-0.644862	1.242713	2.953178
H	-0.370694	0.039889	-3.525243
H	-1.613295	-1.535519	-2.295523
O	-4.744836	-0.375671	-1.361407
O	-4.263741	-0.089279	0.772020
C	-1.919095	-1.492479	0.236561
H	-0.826491	-1.578241	0.144275
C	-2.247105	-1.157566	1.713548
H	-2.636026	-2.037642	2.242257
H	-2.532911	2.145532	1.703153
H	-2.509976	1.846154	-0.686965
C	-2.546209	-2.831281	-0.151791
H	-3.634518	-2.840149	0.023682
H	-2.372703	-3.086712	-1.206142
H	-2.108047	-3.634277	0.459265
O	-1.150545	-0.676012	2.453374
O	-0.137692	2.730802	-0.235177

C	-0.458595	3.515843	-1.357256
H	-0.238712	4.558052	-1.087793
H	0.148230	3.248907	-2.240794
H	-1.526356	3.452598	-1.630182
C	2.883544	1.786595	-0.380087
H	3.424122	1.915530	-1.329779
H	2.155101	2.594457	-0.203849
B	3.145665	-1.933616	-0.523915
F	4.089171	-2.640939	0.224861
F	3.353427	-2.182534	-1.894817
F	1.843007	-2.359324	-0.184182
N	4.682425	2.000222	1.387618
N	3.903611	1.933871	0.620148

53

21_A_6 Eopt -1583.912210

C	-2.253088	0.248621	0.402605
C	-1.367496	-0.228565	1.544949
C	-0.111164	0.192739	1.674008
C	0.437783	1.181847	0.670744
C	-0.169218	0.985456	-0.700329
C	-1.424954	0.555072	-0.859737
C	0.870136	-0.209132	2.726986
C	1.954475	1.003327	0.440028
C	2.424853	-0.468697	0.680142
C	2.070424	-0.944595	2.102652
C	3.928084	-0.403863	0.463327
C	3.177891	0.358585	-1.631728
C	2.214980	1.385603	-1.030803
H	1.217765	0.711328	3.225156
H	-1.836595	-0.907605	2.260192
H	2.951226	-0.825793	2.747835
H	3.629113	0.690569	-2.573402
O	-3.332669	-0.562159	0.142188
C	-2.039229	0.243272	-2.189979
H	-1.960730	-0.837362	-2.380063
H	-3.110352	0.486959	-2.221090
H	-1.525039	0.771926	-3.003326
C	0.889883	1.094484	-1.775552
H	0.645741	1.840669	-2.546400
H	0.396624	-0.837595	3.493495
H	1.848663	-2.019360	2.076547
O	4.792925	-0.687530	1.248339
O	4.256300	0.030610	-0.765585
C	1.896641	-1.426466	-0.450293
H	0.804952	-1.509103	-0.345805
C	2.192480	-0.797697	-1.837395
H	2.550883	-1.556259	-2.545470
H	2.556414	2.419903	-1.147341
H	2.506056	1.647303	1.140588
C	2.497521	-2.829067	-0.370401
H	3.583726	-2.822132	-0.558826
H	2.326822	-3.304515	0.604978
H	2.034930	-3.470818	-1.134785
O	1.089762	-0.156681	-2.435322
O	0.116985	2.459089	1.235943
C	0.524616	3.604268	0.529632
H	0.081802	4.469289	1.042419
H	1.620645	3.732215	0.532455
H	0.167740	3.601767	-0.516310
C	-2.861432	1.576871	0.963048
H	-3.448892	1.400049	1.876584
H	-2.103902	2.364755	1.106523
B	-3.189512	-2.016842	0.037427
F	-4.181040	-2.476765	-0.832294
F	-3.355361	-2.621673	1.298992
F	-1.913737	-2.362581	-0.458729

N	-4.533815	2.448949	-0.723771
N	-3.815375	2.092219	0.022523

53

21_B_1 Eopt -1583.913185

C	-2.052036	-0.175632	-0.229087
C	-1.374083	0.299556	1.050411
C	-0.170555	0.871290	1.038018
C	0.522408	1.087442	-0.287527
C	0.182851	-0.003916	-1.278274
C	-1.014608	-0.598474	-1.286944
C	0.616135	1.350239	2.215640
C	2.060084	1.028427	-0.171034
C	2.538700	0.116080	1.004567
C	1.943186	0.578882	2.348724
C	4.051683	0.260704	0.971484
C	3.670188	-0.569404	-1.197696
C	2.569946	0.448893	-1.505742
H	0.826969	2.421065	2.056132
H	-1.940368	0.150805	1.972016
H	2.681135	1.205142	2.867791
H	4.273714	-0.830796	-2.074049
O	-2.903545	-1.226470	-0.010023
C	-1.411744	-1.705461	-2.210225
H	-1.506214	-2.649492	-1.653660
H	-2.397699	-1.507569	-2.658352
H	-0.675415	-1.844434	-3.011911
C	1.417725	-0.434535	-2.040746
H	1.291213	-0.378638	-3.132024
H	0.033793	1.267307	3.143489
H	1.778335	-0.297578	2.988943
O	4.766979	0.663021	1.849311
O	4.582182	-0.120109	-0.203670
C	2.277784	-1.403280	0.695250
H	1.190716	-1.570644	0.705389
C	2.790320	-1.734964	-0.730598
H	3.304288	-2.704889	-0.749033
H	2.883298	1.223405	-2.214014
H	2.447474	2.040497	0.017062
C	2.923840	-2.337636	1.717638
H	4.024207	-2.279775	1.687874
H	2.602941	-2.123293	2.746127
H	2.644496	-3.378009	1.494443
O	1.783672	-1.777117	-1.715415
O	0.057547	2.373926	-0.714279
C	0.557997	2.882274	-1.926910
H	-0.007732	3.798598	-2.144631
H	1.626294	3.150266	-1.860605
H	0.415588	2.179765	-2.767985
C	-2.762702	1.064545	-0.911061
H	-2.009657	1.829316	-1.165036
H	-3.388207	0.780913	-1.768323
B	-4.227216	-1.112701	0.574542
F	-4.724523	-2.390569	0.781778
F	-5.080281	-0.395058	-0.310002
F	-4.201395	-0.389788	1.791089
N	-4.249880	2.284151	0.726421
N	-3.639363	1.745004	-0.006547

53

21_B_2 Eopt -1583.867467

C	2.051478	-0.059105	-0.225495
C	1.213115	0.859131	-1.105954
C	0.023897	1.319891	-0.721412
C	-0.514823	0.901612	0.629185
C	-0.055574	-0.488743	1.009490
C	1.134917	-0.969553	0.628028
C	-0.893210	2.217873	-1.488796

C	-2.055825	0.807871	0.638530
C	-2.650416	0.468085	-0.765672
C	-2.217975	1.504962	-1.820211
C	-4.157302	0.499835	-0.534089
C	-3.531615	-1.165173	1.003526
C	-2.413490	-0.321772	1.625186
H	-1.092950	3.100240	-0.857706
H	1.666628	1.120302	-2.066414
H	-3.023755	2.241688	-1.940799
H	-4.027900	-1.818705	1.730245
O	2.928319	-0.760142	-0.982654
C	1.611318	-2.364347	0.862343
H	1.727038	-2.869269	-0.108030
H	2.612105	-2.361442	1.315152
H	0.907575	-2.937285	1.476983
C	-1.198631	-1.281824	1.609001
H	-0.956047	-1.701464	2.597382
H	-0.415474	2.581897	-2.408826
H	-2.114873	1.005565	-2.792238
O	-4.956203	1.205510	-1.077127
O	-4.551106	-0.389390	0.401761
C	-2.334109	-1.014202	-1.177834
H	-1.251454	-1.100256	-1.354872
C	-2.688010	-1.960936	-0.000815
H	-3.182851	-2.871776	-0.362693
H	-2.661135	0.048464	2.626786
H	-2.474999	1.778042	0.945642
C	-3.074163	-1.448147	-2.443540
H	-4.165694	-1.455775	-2.294717
H	-2.859923	-0.797507	-3.301611
H	-2.768356	-2.467717	-2.719548
O	-1.585301	-2.367600	0.770694
O	-0.030408	1.913944	1.529875

C	-0.410558	1.815751	2.877434
H	0.149246	2.584878	3.426205
H	-1.486689	2.010707	3.025275
H	-0.164826	0.827681	3.308294
C	2.769916	0.866702	0.862407
H	2.006318	1.347249	1.495132
H	3.543085	0.294530	1.399496
B	4.357483	-0.937907	-0.642198
F	4.972418	0.346044	-0.712310
F	4.912999	-1.825772	-1.509610
F	4.485657	-1.355495	0.712374
N	3.828577	2.851433	-0.271559
N	3.430252	1.962915	0.235777

53

21_B_3 Eopt -1583.913405

C	-2.041604	-0.312175	-0.170704
C	-1.389121	0.597493	0.864191
C	-0.187781	1.142626	0.665573
C	0.509033	0.888361	-0.651454
C	0.199221	-0.485203	-1.194704
C	-0.983553	-1.071105	-0.995638
C	0.619274	1.912845	1.666881
C	2.038700	0.901916	-0.520432
C	2.542560	0.370881	0.861339
C	1.902193	1.142549	2.034979
C	4.044774	0.580186	0.768679
C	3.706928	-0.843846	-1.082440
C	2.548499	-0.014730	-1.645189
H	0.892883	2.888179	1.234385
H	-1.950240	0.740424	1.789772
H	2.640712	1.840148	2.452979
H	4.326934	-1.308716	-1.856937
O	-2.891059	-1.220325	0.403456

C	-1.365963	-2.425508	-1.499542
H	-1.502824	-3.119707	-0.657312
H	-2.328284	-2.389553	-2.033877
H	-0.600115	-2.835060	-2.170170
C	1.431700	-1.066864	-1.855889
H	1.262578	-1.322811	-2.911699
H	0.032003	2.114212	2.573610
H	1.656472	0.432185	2.835416
O	4.739341	1.247100	1.487761
O	4.591734	-0.082962	-0.266311
C	2.372893	-1.185306	0.995441
H	1.298006	-1.403474	1.076924
C	2.896737	-1.877499	-0.287861
H	3.465109	-2.784875	-0.045077
H	2.797485	0.537115	-2.558021
H	2.414726	1.928675	-0.629969
C	3.079821	-1.749542	2.227719
H	4.175127	-1.653027	2.150892
H	2.762936	-1.256662	3.157190
H	2.851859	-2.821282	2.326137
O	1.884868	-2.247369	-1.194434
O	0.084630	1.793578	-1.682120
C	0.105744	3.171854	-1.399209
H	-0.172108	3.689262	-2.327646
H	-0.621117	3.447162	-0.614236
H	1.105228	3.528848	-1.095779
C	-2.747009	0.575711	-1.278496
H	-1.989490	1.178738	-1.809119
H	-3.366778	-0.018414	-1.964021
B	-4.219325	-0.904986	0.898188
F	-4.716402	-2.019930	1.555902
F	-5.066361	-0.562995	-0.192402
F	-4.202459	0.213005	1.765733

N	-4.249747	2.313925	-0.233064
N	-3.630520	1.544189	-0.707474

53

21_B_4 Eopt -1583.911711

C	-2.049050	-0.087497	0.128942
C	-1.252684	0.838937	1.039070
C	-0.041157	1.289140	0.709758
C	0.529516	0.885858	-0.631967
C	0.092972	-0.502230	-1.033467
C	-1.107015	-0.986796	-0.703664
C	0.886155	2.084936	1.577362
C	2.062776	0.808395	-0.617713
C	2.644882	0.361790	0.763104
C	2.149902	1.268325	1.910144
C	4.144296	0.463581	0.538443
C	3.573440	-1.083899	-1.149358
C	2.424424	-0.229793	-1.693140
H	1.177004	3.002496	1.042059
H	-1.733182	1.085387	1.990510
H	2.958990	1.950493	2.204728
H	4.100377	-1.650567	-1.925024
O	-2.910182	-0.832883	0.893721
C	-1.599701	-2.356512	-1.041055
H	-1.720811	-2.947024	-0.120166
H	-2.592194	-2.312191	-1.510891
H	-0.901288	-2.883814	-1.702642
C	1.233720	-1.219327	-1.726038
H	0.967204	-1.552212	-2.739402
H	0.386809	2.395718	2.505615
H	1.932624	0.645324	2.787853
O	4.932660	1.141469	1.141149
O	4.565132	-0.317750	-0.472454
C	2.395044	-1.163152	1.046001

H	1.319205	-1.302987	1.227529
C	2.770380	-1.992221	-0.207721
H	3.301817	-2.912027	0.069794
H	2.630846	0.227404	-2.666906
H	2.489291	1.795328	-0.845590
C	3.166332	-1.668492	2.265263
H	4.255384	-1.650967	2.096106
H	2.956895	-1.080572	3.169482
H	2.884628	-2.711087	2.474668
O	1.667195	-2.367495	-0.997306
O	0.079847	1.736460	-1.697453
C	0.204383	3.128138	-1.530403
H	-0.107245	3.588868	-2.477714
H	-0.447103	3.510942	-0.724535
H	1.242575	3.440791	-1.323838
C	-2.769988	0.790035	-0.970037
H	-2.016672	1.254892	-1.629979
H	-3.530343	0.227860	-1.532210
B	-4.330527	-1.012883	0.657019
F	-4.999877	0.235702	0.776808
F	-4.817335	-1.908552	1.598319
F	-4.595332	-1.490385	-0.651658
N	-3.917149	2.779113	0.079521
N	-3.456887	1.900568	-0.386402

53

21_B_5 Eopt -1583.912947

C	2.247570	0.266733	0.323496
C	1.572426	0.601298	-0.999439
C	0.322523	1.061385	-1.053529
C	-0.406850	1.316695	0.245183
C	-0.000322	0.347430	1.327410
C	1.230171	-0.163377	1.393981
C	-0.506929	1.240022	-2.288998
C	-1.922841	1.105901	0.113466
C	-2.293300	-0.025439	-0.901702
C	-1.669071	0.228710	-2.290830
C	-3.811548	0.029159	-0.921803
C	-3.440157	-0.402969	1.367070
C	-2.402355	0.713404	1.520867
H	-0.912435	2.263810	-2.313660
H	2.151965	0.387511	-1.900936
H	-2.452380	0.569631	-2.981588
H	-4.053991	-0.553401	2.262105
O	3.302115	-0.606286	0.180529
C	1.703143	-1.135398	2.425437
H	1.835921	-2.127601	1.969752
H	2.681482	-0.837815	2.833476
H	0.984813	-1.228688	3.249752
C	-1.201238	-0.012612	2.176300
H	-1.059943	0.240210	3.237097
H	0.101452	1.108512	-3.194652
H	-1.289894	-0.721552	-2.689431
O	-4.527175	0.234682	-1.865416
O	-4.348531	-0.177136	0.294275
C	-1.957239	-1.454623	-0.339756
H	-0.862933	-1.559150	-0.308856
C	-2.488147	-1.579744	1.110903
H	-2.951001	-2.560785	1.280051
H	-2.755186	1.572847	2.101176
H	-2.402429	2.030485	-0.237209
C	-2.528066	-2.575644	-1.207975
H	-3.630281	-2.587020	-1.190322
H	-2.208319	-2.499753	-2.256322
H	-2.183326	-3.548318	-0.826859
O	-1.504659	-1.404477	2.103052
O	-0.114118	2.613071	0.785651

C	-0.285657	3.728541	-0.053407
H	-0.104534	4.619514	0.563446
H	0.432680	3.734166	-0.892767
H	-1.307896	3.800320	-0.464180
C	2.879846	1.560954	0.936996
H	2.174693	2.405839	0.986249
H	3.358382	1.365718	1.908851
B	3.176033	-1.831213	-0.613584
F	4.034613	-2.785402	-0.062984
F	3.545728	-1.588832	-1.952696
F	1.848198	-2.307607	-0.593393
N	4.758548	2.188284	-0.631618
N	3.956214	1.959825	0.078423

53

21_B_6 Eopt -1583.913678

C	2.251321	0.382812	0.176058
C	1.548342	0.308570	-1.171354
C	0.297983	0.735482	-1.332610
C	-0.423998	1.342251	-0.152541
C	0.011346	0.721083	1.155761
C	1.257387	0.276255	1.344624
C	-0.520853	0.682079	-2.581507
C	-1.946621	1.090033	-0.200290
C	-2.305428	-0.233404	-0.952337
C	-1.747728	-0.228861	-2.389438
C	-3.825288	-0.254071	-0.935907
C	-3.390819	-0.189190	1.375134
C	-2.412226	0.982934	1.265627
H	-0.853740	1.709235	-2.806027
H	2.127704	-0.113339	-1.995969
H	-2.545506	0.077437	-3.079349
H	-3.976147	-0.177215	2.301257
O	3.315481	-0.483830	0.282745
C	1.753373	-0.380838	2.592212
H	1.824615	-1.467180	2.435581
H	2.763236	-0.027847	2.850013
H	1.080591	-0.197078	3.439886
C	-1.180408	0.480023	2.056575
H	-1.073307	0.945725	3.047550
H	0.077458	0.337300	-3.435951
H	-1.471380	-1.253129	-2.671217
O	-4.566279	-0.283064	-1.881925
O	-4.330595	-0.233424	0.309386
C	-1.877749	-1.496302	-0.117191
H	-0.779028	-1.533196	-0.088191
C	-2.382271	-1.343325	1.341946
H	-2.789166	-2.290482	1.719453
H	-2.817908	1.923901	1.652604
H	-2.435525	1.919430	-0.732131
C	-2.388530	-2.806230	-0.715419
H	-3.488979	-2.867791	-0.691438
H	-2.064648	-2.948204	-1.755362
H	-1.998500	-3.652941	-0.131408
O	-1.400129	-0.917015	2.257420
O	-0.094735	2.734319	-0.230713
C	-0.635473	3.587472	0.747138
H	-0.171174	4.572796	0.602734
H	-1.727426	3.708258	0.642402
H	-0.410313	3.246486	1.773887
C	2.876425	1.809671	0.337113
H	2.158529	2.615527	0.115184
H	3.375835	1.944599	1.308467
B	3.199518	-1.888424	-0.118052
F	4.094470	-2.626703	0.659891
F	3.530735	-2.041706	-1.479705
F	1.884592	-2.360080	0.080577

N 4.709670 1.919974 -1.399291
N 3.928381 1.921695 -0.631488
51
22 Eopt -1474.587150
C -2.093431 2.037024 -0.435723
C -1.950117 -0.540313 -0.737346
C -0.725559 -0.839318 -0.262791
C 0.040406 0.047300 0.712236
C 0.263957 1.498802 0.317709
C -0.644350 2.344460 -0.184866
C -0.137393 -2.197391 -0.488282
C 1.473566 -0.493680 0.926824
C 2.161296 -1.071396 -0.356737
C 1.290288 -2.140554 -1.035021
C 3.462777 -1.632268 0.195195
C 3.682102 0.631728 0.806141
C 2.287718 0.700370 1.436042
H -0.134415 -2.708009 0.490228
H -2.513421 -1.305517 -1.276340
H 1.759727 -3.126669 -0.921206
H 4.421892 1.254790 1.320493
C -0.319213 3.783027 -0.484657
H -0.509332 3.995427 -1.549396
H -0.974006 4.454147 0.094821
H 0.725357 4.039390 -0.277635
C 1.654828 1.929555 0.756462
H 1.600160 2.803284 1.420779
H -0.779278 -2.789576 -1.153559
H 1.235895 -1.937370 -2.112975
O 3.844674 -2.770654 0.154150
O 4.216772 -0.688090 0.784262
C 2.609086 0.042860 -1.362538
H 1.701666 0.483905 -1.803263
C 3.341940 1.165069 -0.593985
H 4.217476 1.526616 -1.149186
H 2.295436 0.770451 2.528880
H 1.443429 -1.307112 1.664649
C 3.487085 -0.504307 -2.487848
H 4.459395 -0.860009 -2.109980
H 3.011094 -1.336057 -3.025663
H 3.691682 0.292114 -3.218646
O 2.522719 2.274637 -0.321860
O -0.663611 0.131254 1.948681
C -1.088053 -1.060116 2.569167
H -1.510888 -0.770707 3.540404
H -1.875123 -1.571868 1.989367
H -0.259372 -1.766157 2.752143
C -2.677625 0.667908 -0.425572
H -2.709282 2.659793 0.233818
H -2.360752 2.393174 -1.451553
O -3.923397 0.641282 -0.263514
B -4.854850 -0.624618 -0.243102
F -4.350200 -1.481203 0.712887
F -6.085972 -0.123623 0.089138
F -4.825627 -1.171945 -1.509902
51
23 Eopt -1474.586366
C -2.482470 -0.216359 -0.442204
C -1.556469 1.848658 0.513019
C -0.236558 1.707596 0.395261
C 0.374120 0.974894 -0.796252
C -0.124693 -0.435345 -1.126695
C -1.351998 -0.982910 -0.981891
C 0.754531 2.320105 1.345053
C 1.886609 0.760603 -0.582253
C 2.283629 0.318305 0.864452

C	1.709119	1.274622	1.926626
C	3.803117	0.337368	0.800608
C	3.327823	-1.228363	-0.897460
C	2.290900	-0.327701	-1.578169
H	1.331759	3.085435	0.798788
H	-1.988326	2.393592	1.356346
H	2.533017	1.774142	2.454385
H	3.897726	-1.844981	-1.600994
O	-3.422630	-0.890172	0.033797
C	-1.646684	-2.430235	-1.279991
H	-1.681480	-3.012358	-0.346230
H	-2.626673	-2.534552	-1.766214
H	-0.883288	-2.880087	-1.923166
C	1.049634	-1.234312	-1.685248
H	0.827914	-1.560400	-2.712039
H	0.227931	2.840459	2.157066
H	1.158227	0.689888	2.676789
O	4.557798	0.990257	1.468883
O	4.284956	-0.495057	-0.140938
C	1.923276	-1.177476	1.159072
H	0.827014	-1.255634	1.223255
C	2.383453	-2.062800	-0.020885
H	2.823244	-3.005838	0.327995
H	2.614455	0.076390	-2.542598
H	2.407505	1.706683	-0.781048
C	2.521112	-1.670277	2.476247
H	3.621666	-1.709785	2.435004
H	2.238284	-1.032807	3.325495
H	2.163831	-2.688703	2.688827
O	1.338262	-2.389560	-0.908885
O	0.156728	1.663673	-2.026730
C	0.469899	3.035411	-2.079821
H	0.259390	3.361791	-3.106956
H	-0.152273	3.629217	-1.388569
H	1.533590	3.237869	-1.866211
F	-5.463302	0.301813	-0.235964
F	-4.319730	0.487773	1.742981
F	-5.348758	-1.484034	1.193204
B	-4.734816	-0.351309	0.732698
C	-2.520358	1.273060	-0.500028
H	-2.203432	1.564663	-1.513021
H	-3.538768	1.629385	-0.328040

51

24'_1 Eopt -1474.546937

C	1.936456	-0.145596	1.108199
C	1.111341	-1.369378	1.288848
C	0.073702	-1.621750	0.486306
C	-0.188789	-0.717789	-0.693376
C	0.291277	0.694384	-0.455106
C	1.324388	0.993686	0.343367
C	-0.964804	-2.680210	0.661718
C	-1.684274	-0.570133	-0.988270
C	-2.574235	-0.679740	0.281840
C	-2.310357	-2.010574	1.022399
C	-3.985397	-0.590137	-0.274899
C	-3.102258	1.455589	-1.052295
C	-1.841816	0.813717	-1.638911
H	-1.063270	-3.231820	-0.286900
H	1.366231	-2.024574	2.125993
H	-3.132919	-2.707311	0.811110
H	-3.463603	2.314487	-1.628562
O	3.380341	-0.511214	0.801867
C	1.764408	2.395236	0.645333
H	1.490121	2.670175	1.676522
H	2.853059	2.504086	0.546453
H	1.290479	3.115457	-0.031901

C	-0.700134	1.675336	-1.051862
H	-0.246589	2.366714	-1.776094
H	-0.683581	-3.402598	1.440043
H	-2.323434	-1.829140	2.105844
O	-4.869105	-1.396733	-0.165728
O	-4.196159	0.549504	-0.957712
C	-2.429303	0.591058	1.192727
H	-1.421527	0.578994	1.633605
C	-2.542994	1.867175	0.316117
H	-3.142522	2.637260	0.818998
H	-1.829579	0.768833	-2.733015
H	-1.971239	-1.377907	-1.674727
C	-3.455512	0.626310	2.324336
H	-4.481744	0.748181	1.941528
H	-3.432515	-0.285249	2.937772
H	-3.250001	1.479941	2.987292
O	-1.306183	2.455456	-0.018043
O	0.341880	-1.279088	-1.890439
C	1.739703	-1.381234	-2.006346
H	1.937541	-1.846004	-2.981665
H	2.231750	-0.396422	-1.982652
H	2.181292	-2.022298	-1.224361
C	3.035108	0.088265	2.057438
H	3.163850	-0.592095	2.903469
H	3.425230	1.098219	2.198929
B	4.494494	0.039525	-0.230933
F	4.862194	-1.081127	-0.919866
F	5.472013	0.540242	0.587558
F	3.915264	0.994971	-1.025244

51

24'_2 Eopt -1474.544223

C	2.026744	0.316334	-1.208698
C	1.135011	1.500737	-1.330706
C	0.095044	1.649634	-0.508158
C	-0.083757	0.651473	0.613686
C	0.390221	-0.733002	0.221952
C	1.431448	-0.920652	-0.596996
C	-0.935560	2.727429	-0.523794
C	-1.564448	0.450662	1.001543
C	-2.535842	0.716754	-0.190934
C	-2.343416	2.138804	-0.754841
C	-3.914624	0.527695	0.420118
C	-2.979052	-1.589472	0.852013
C	-1.696005	-1.015324	1.458003
H	-0.899840	3.222181	0.460873
H	1.366274	2.230429	-2.110686
H	-3.092771	2.802923	-0.303883
H	-3.297861	-2.525966	1.322985
O	3.410643	0.722403	-0.736604
C	1.924679	-2.265923	-1.035525
H	1.782342	-2.398255	-2.120039
H	2.998622	-2.377040	-0.826231
H	1.389063	-3.073329	-0.522516
C	-0.583126	-1.784501	0.709184
H	-0.104686	-2.569364	1.312649
H	-0.710567	3.489659	-1.281956
H	-2.549680	2.130070	-1.833056
O	-4.806326	1.331270	0.474083
O	-4.084558	-0.699653	0.941867
C	-2.424216	-0.413526	-1.277797
H	-1.438448	-0.328444	-1.759185
C	-2.488333	-1.800571	-0.584901
H	-3.109416	-2.499436	-1.160322
H	-1.639417	-1.129028	2.545911
H	-1.819475	1.157595	1.804228
C	-3.500656	-0.312080	-2.358019

H	-4.510302	-0.473655	-1.946636
H	-3.496838	0.661536	-2.866955
H	-3.331418	-1.085151	-3.122248
O	-1.233845	-2.414951	-0.397493
O	0.680549	1.215123	1.667900
C	0.770224	0.488449	2.865528
H	1.521201	0.993400	3.487608
H	-0.184072	0.474463	3.422164
H	1.105679	-0.550312	2.701797
C	3.209043	0.242320	-2.076560
H	3.365131	1.017381	-2.831541
H	3.676924	-0.722742	-2.279545
B	4.364249	0.025885	0.364598
F	5.306552	0.985979	0.606456
F	4.886184	-1.099370	-0.230702
F	3.565747	-0.248977	1.437022

51

24'_3 Eopt -1474.541687

C	-2.036140	0.022668	1.186270
C	-1.178691	1.185039	1.543863
C	-0.123634	1.507823	0.791396
C	0.094970	0.747687	-0.497462
C	-0.365666	-0.687215	-0.403677
C	-1.412119	-1.053380	0.342205
C	0.952162	2.490256	1.130433
C	1.581961	0.644353	-0.865424
C	2.518438	0.559055	0.380377
C	2.290431	1.749355	1.338257
C	3.904673	0.559584	-0.241335
C	2.985695	-1.355519	-1.271678
C	1.700653	-0.640890	-1.700846
H	1.058908	3.208533	0.303073
H	-1.415886	1.720342	2.467074
H	3.119474	2.461897	1.230718
H	3.320781	-2.116919	-1.984508
O	-3.436340	0.460770	0.806715
C	-1.914624	-2.461057	0.453187
H	-1.784474	-2.841759	1.478974
H	-2.986216	-2.520747	0.212774
H	-1.373321	-3.130334	-0.225889
C	0.584218	-1.577452	-1.177703
H	0.079462	-2.149483	-1.968838
H	0.697669	3.064826	2.031720
H	2.323162	1.386158	2.374332
O	4.791974	1.348510	-0.056420
O	4.084248	-0.467285	-1.091744
C	2.411437	-0.833802	1.098402
H	1.423270	-0.893394	1.578387
C	2.488817	-1.968291	0.044671
H	3.110979	-2.798980	0.402645
H	1.632847	-0.451660	-2.777312
H	1.881544	1.531697	-1.440258
C	3.486268	-1.027763	2.167477
H	4.494376	-1.087430	1.726123
H	3.492112	-0.216721	2.908778
H	3.308802	-1.970513	2.706024
O	1.237389	-2.507580	-0.312541
O	-0.650744	1.285093	-1.582433
C	-0.588936	2.668441	-1.811890
H	-1.165986	2.859945	-2.726732
H	-1.045509	3.247782	-0.989771
H	0.441285	3.034260	-1.975234
C	-3.204935	-0.254517	2.031386
H	-3.378892	0.357830	2.920121
H	-3.636686	-1.256505	2.051818
B	-4.401027	-0.040322	-0.398663

F	-5.282898	0.995650	-0.528329
F	-4.991755	-1.197916	0.050889
F	-3.598085	-0.224349	-1.483313

51

24'_4 Eopt -1474.544796

C	2.060361	0.798921	0.767448
C	1.543261	-0.517197	1.229909
C	0.512634	-1.094028	0.605415
C	-0.019725	-0.452546	-0.655900
C	0.107366	1.051287	-0.635360
C	1.120625	1.668121	-0.020931
C	-0.270180	-2.274116	1.087455
C	-1.522032	-0.702381	-0.846831
C	-2.308201	-0.774568	0.499177
C	-1.708553	-1.840520	1.442791
C	-3.718503	-1.112796	0.046818
C	-3.380130	0.912240	-1.118968
C	-2.021899	0.486042	-1.684651
H	-0.304088	-3.033814	0.291401
H	1.986896	-0.952515	2.127237
H	-2.362167	-2.723365	1.449661
H	-3.956882	1.547344	-1.800350
O	3.455471	0.800822	0.178497
C	1.332031	3.151065	-0.020325
H	1.241088	3.563703	0.997044
H	2.338550	3.403162	-0.391020
H	0.597522	3.658332	-0.657238
C	-1.097069	1.671700	-1.313064
H	-0.826654	2.306920	-2.168256
H	0.207729	-2.738501	1.960966
H	-1.703282	-1.447847	2.468493
O	-4.377360	-2.070540	0.350749
O	-4.220447	-0.190941	-0.794950
C	-2.442077	0.637072	1.175476
H	-1.446080	0.937074	1.533982
C	-2.892717	1.678069	0.118487
H	-3.644441	2.362567	0.532892
H	-2.033091	0.270337	-2.758188
H	-1.672569	-1.659089	-1.366129
C	-3.406377	0.631665	2.361227
H	-4.444317	0.442831	2.042362
H	-3.142800	-0.125837	3.112379
H	-3.390283	1.613214	2.857821
O	-1.845007	2.468675	-0.393967
O	0.700528	-0.838756	-1.819138
C	0.919733	-2.208884	-2.034231
H	1.451727	-2.294483	-2.991236
H	1.552049	-2.657419	-1.249770
H	-0.020590	-2.784929	-2.112195
C	3.210509	1.384717	1.470103
H	3.643450	0.821049	2.300519
H	3.373125	2.463794	1.485879
B	4.401943	-0.483563	-0.041731
F	5.607504	0.074156	-0.370174
F	3.813934	-1.182613	-1.055076
F	4.436958	-1.166208	1.150651

51

24'_5 Eopt -1474.547774

C	-1.966516	0.494990	-0.809290
C	-1.436881	-0.882781	-0.981607
C	-0.385116	-1.306443	-0.274107
C	0.161056	-0.446903	0.836462
C	-0.060251	1.024950	0.593316
C	-1.091727	1.499982	-0.115352
C	0.420134	-2.540519	-0.516631
C	1.675905	-0.586928	0.995833

C	2.404365	-0.873665	-0.347177
C	1.822199	-2.134951	-1.026323
C	3.852971	-1.050748	0.076080
C	3.449755	1.132613	0.878389
C	2.150689	0.748102	1.592799
H	0.511925	-3.089742	0.434241
H	-1.882413	-1.498057	-1.765039
H	2.518039	-2.972769	-0.883090
H	4.019885	1.910791	1.397715
O	-3.420057	0.637877	-0.386943
C	-1.353317	2.958530	-0.340142
H	-1.163463	3.238177	-1.388509
H	-2.401911	3.207190	-0.112790
H	-0.712207	3.580814	0.295502
C	1.142095	1.806688	1.087282
H	0.892932	2.575669	1.831937
H	-0.064774	-3.208419	-1.241468
H	1.756250	-1.963549	-2.109581
O	4.554411	-2.011413	-0.092489
O	4.338316	0.033277	0.708220
C	2.418203	0.394945	-1.272866
H	1.390260	0.572060	-1.621809
C	2.853191	1.633354	-0.443580
H	3.538276	2.270337	-1.018325
H	2.232613	0.716485	2.684277
H	1.868273	-1.428181	1.675000
C	3.323342	0.223908	-2.491958
H	4.385436	0.155663	-2.205575
H	3.073903	-0.674330	-3.073834
H	3.219730	1.093014	-3.158503
O	1.788136	2.451340	-0.013213
O	-0.355329	-0.840975	2.106052
C	-1.746808	-0.778615	2.294369
H	-1.933292	-1.054153	3.341123
H	-2.149927	0.234675	2.128373
H	-2.287183	-1.486807	1.645945
C	-3.023377	0.941748	-1.731027
H	-3.380918	0.225265	-2.476335
H	-3.146475	1.995042	-1.989865
B	-4.565164	-0.440522	-0.032284
F	-5.651783	0.041552	-0.713209
F	-4.682409	-0.364942	1.326295
F	-4.150520	-1.666272	-0.482841

51

24'_6 Eopt -1474.543552

C	-2.074639	0.564745	-0.937699
C	-1.502295	-0.782663	-1.199373
C	-0.464806	-1.220746	-0.482782
C	0.015289	-0.386348	0.681993
C	-0.151118	1.097121	0.425560
C	-1.164798	1.578425	-0.302407
C	0.297796	-2.488827	-0.667444
C	1.523281	-0.555811	0.959274
C	2.329698	-0.916123	-0.327287
C	1.779169	-2.197238	-0.986581
C	3.750096	-1.097671	0.181334
C	3.353237	1.125082	0.852569
C	2.012823	0.795713	1.514630
H	0.227379	-3.049065	0.279629
H	-1.939777	-1.378646	-2.001565
H	2.393091	-3.051204	-0.670541
H	3.906772	1.917750	1.367914
O	-3.460682	0.611236	-0.326926
C	-1.390698	3.031656	-0.585111
H	-1.302840	3.241483	-1.662980
H	-2.401478	3.337889	-0.270708

H	-0.664629	3.661020	-0.056927
C	1.060057	1.859155	0.919730
H	0.807975	2.668442	1.620107
H	-0.146379	-3.115575	-1.452416
H	1.895429	-2.120530	-2.075682
O	4.436203	-2.080599	0.100034
O	4.228377	0.006836	0.781151
C	2.413154	0.309131	-1.308696
H	1.406243	0.489762	-1.713868
C	2.835531	1.575830	-0.518225
H	3.562769	2.170458	-1.086398
H	2.051287	0.816839	2.608990
H	1.660615	-1.373645	1.681244
C	3.371442	0.071706	-2.475197
H	4.415197	-0.022481	-2.133751
H	3.121872	-0.832050	-3.047631
H	3.329986	0.925178	-3.168190
O	1.769341	2.434042	-0.180398
O	-0.785860	-0.850868	1.756653
C	-0.666950	-0.208887	2.998032
H	-1.459876	-0.616137	3.639701
H	0.304503	-0.403733	3.486881
H	-0.816468	0.883442	2.922874
C	-3.259344	0.986682	-1.698200
H	-3.677353	0.278869	-2.419173
H	-3.467811	2.040908	-1.889012
B	-4.409538	-0.605982	0.127931
F	-5.659421	-0.044677	0.082966
F	-3.985154	-0.923027	1.383068
F	-4.250915	-1.615609	-0.789032

51

24_1 Eopt -1474.545957

C	2.237293	0.761273	-0.228415
C	1.638555	-0.113289	-1.274112
C	0.343994	-0.005118	-1.572817
C	-0.477800	1.077852	-0.914974
C	0.009253	1.385573	0.483199
C	1.299566	1.286930	0.820953
C	-0.433306	-0.846006	-2.530784
C	-1.951129	0.661683	-0.717592
C	-2.116766	-0.883422	-0.562945
C	-1.531465	-1.628079	-1.780616
C	-3.620352	-1.072390	-0.447067
C	-3.242843	0.355888	1.386330
C	-2.428903	1.362350	0.569208
H	-0.896414	-0.171797	-3.269692
H	2.295797	-0.814080	-1.790973
H	-2.347072	-1.869199	-2.475469
H	-3.842385	0.824197	2.174829
O	3.484414	0.246181	0.383528
C	1.840399	1.524718	2.197482
H	2.088517	0.567763	2.682220
H	2.764064	2.122786	2.169928
H	1.108635	2.046521	2.826171
C	-1.158710	1.563057	1.429505
H	-1.139020	2.519174	1.972712
H	0.218916	-1.539077	-3.079173
H	-1.113552	-2.587546	-1.448963
O	-4.333007	-1.724464	-1.161860
O	-4.146531	-0.403831	0.594220
C	-1.545970	-1.381842	0.815242
H	-0.451684	-1.271261	0.791266
C	-2.087659	-0.476983	1.953935
H	-2.369481	-1.074230	2.830890
H	-2.966064	2.295911	0.370307
H	-2.532042	0.972798	-1.598079

C	-1.878644	-2.845621	1.101921
H	-2.962115	-3.001691	1.231319
H	-1.533849	-3.518891	0.305246
H	-1.388263	-3.159652	2.035286
O	-1.184388	0.510682	2.396594
O	-0.337357	2.182263	-1.801161
C	-0.983692	3.379592	-1.458215
H	-0.655835	4.137072	-2.183461
H	-2.083944	3.303921	-1.520259
H	-0.708668	3.732284	-0.447173
C	3.547986	1.392733	-0.479704
H	3.829725	2.307758	0.048378
H	4.036218	1.203894	-1.439492
B	4.186345	-1.197538	0.153472
F	4.523906	-1.261932	-1.174982
F	3.219642	-2.086605	0.525786
F	5.266775	-1.153575	0.983978
51			
24_2			Eopt -1474.545316
C	2.236284	0.615667	0.472936
C	1.652195	0.844493	-0.878240
C	0.360878	1.156642	-1.004567
C	-0.461722	1.372945	0.244449
C	0.002569	0.508378	1.388971
C	1.285094	0.172010	1.548866
C	-0.413735	1.219182	-2.284508
C	-1.931760	0.976139	0.046055
C	-2.114551	-0.215587	-0.946422
C	-1.455301	0.080805	-2.311780
C	-3.625532	-0.345302	-1.037003
C	-3.317875	-0.664451	1.281523
C	-2.433922	0.576043	1.443600
H	-0.930399	2.188559	-2.355817

H	2.308545	0.730952	-1.742601
H	-2.237475	0.320112	-3.045065
H	-3.953029	-0.864458	2.151546
O	3.478048	-0.191285	0.486357
C	1.812153	-0.683243	2.659944
H	2.124601	-1.666367	2.275377
H	2.693032	-0.226690	3.137809
H	1.047577	-0.844846	3.429425
C	-1.191016	0.028425	2.187823
H	-1.144393	0.322416	3.246044
H	0.251047	1.137564	-3.155517
H	-0.955703	-0.828572	-2.671623
O	-4.313637	-0.252066	-2.017736
O	-4.191823	-0.583132	0.160325
C	-1.639117	-1.576476	-0.321519
H	-0.542595	-1.547187	-0.238541
C	-2.217134	-1.720102	1.109803
H	-2.562529	-2.745535	1.295521
H	-2.919657	1.404429	1.970196
H	-2.494459	1.828766	-0.359215
C	-2.030506	-2.782513	-1.174960
H	-3.123267	-2.922816	-1.208186
H	-1.670759	-2.698679	-2.209733
H	-1.595579	-3.697188	-0.745435
O	-1.308168	-1.393564	2.135676
O	-0.362631	2.704450	0.743781
C	-0.582885	3.763332	-0.150852
H	-0.551810	4.688522	0.440991
H	0.200188	3.823898	-0.927827
H	-1.568484	3.710258	-0.647716
C	3.547991	1.207717	0.804412
H	3.822275	1.395047	1.845731
H	4.046384	1.814783	0.043970

B	4.196825	-0.949740	-0.754450
F	4.543234	0.022397	-1.659203
F	3.237337	-1.810075	-1.203812
F	5.271140	-1.551668	-0.169818

65

26a-Al'_1 Eopt -3290.931151

C	1.195873	0.242619	0.320718
C	0.307730	0.253177	1.555918
C	-0.787743	-0.495546	1.681533
C	-1.140590	-1.476701	0.597470
C	-0.632163	-1.036494	-0.751240
C	0.480846	-0.313042	-0.922259
C	-1.804913	-0.437536	2.777753
C	-2.650232	-1.652580	0.416340
C	-3.457179	-0.375405	0.770005
C	-3.156955	0.068124	2.219448
C	-4.903543	-0.796212	0.577935
C	-4.054347	-1.244360	-1.577922
C	-2.844187	-2.028488	-1.063326
H	-1.932170	-1.453800	3.184594
H	0.603784	0.928511	2.366762
H	-3.970190	-0.280861	2.870204
H	-4.447222	-1.620542	-2.529050
O	2.375721	-0.396687	0.608529
C	1.054495	-0.013742	-2.278857
H	0.717945	-0.749550	-3.020195
H	0.762029	0.979890	-2.647807
H	2.154142	-0.045840	-2.266779
C	-1.655928	-1.382763	-1.814424
H	-1.261531	-1.994353	-2.638031
H	-1.475436	0.208087	3.603973
H	-3.174027	1.165040	2.272933
O	-5.789391	-0.768133	1.389471
O	-5.148731	-1.240321	-0.667940
C	-3.220631	0.739620	-0.306321
H	-2.175666	1.062042	-0.213396
C	-3.396097	0.132079	-1.726363
H	-3.954543	0.814944	-2.380078
H	-2.913487	-3.110413	-1.217870
H	-2.978652	-2.458588	1.085790
C	-4.122175	1.957710	-0.125820
H	-3.843397	2.737624	-0.850177
H	-5.182061	1.711042	-0.300836
H	-4.041365	2.393212	0.879820
O	-2.185013	-0.175564	-2.382387
O	-0.702393	-2.796922	0.920271
C	0.676994	-3.046988	1.013598
H	1.200342	-2.881684	0.057794
H	1.167440	-2.432159	1.786506
H	0.778943	-4.106107	1.288496
C	1.557409	1.771985	-0.012971
H	2.161464	1.808982	-0.938320
N	3.171669	2.459494	1.768600
N	2.478227	2.163683	0.967228
Si	0.211528	3.233260	-0.189106
C	-0.700220	2.967373	-1.790080
H	-1.143924	1.967840	-1.904108
H	-1.512618	3.712670	-1.830439
H	-0.037334	3.156881	-2.648945
C	-0.882781	3.280135	1.318615
H	-0.294193	3.392361	2.242848
H	-1.537620	4.163773	1.232059
H	-1.517625	2.390474	1.416826
C	1.272280	4.769640	-0.277344
H	0.621258	5.637363	-0.475859
H	1.807837	4.971573	0.664399

H	2.008688	4.711166	-1.094794
Al	3.872643	-0.984769	-0.063992
Cl	3.592723	-2.547349	-1.529307
Cl	5.055593	-1.700851	1.586473
Cl	4.842094	0.725844	-1.011621

65

26a-Al'_10 Eopt -3290.922967

C	1.260143	0.473463	0.288493
C	0.337389	0.709159	1.479380
C	-0.763528	-0.025713	1.634755
C	-1.060098	-1.078334	0.591485
C	-0.634092	-0.634069	-0.786372
C	0.457375	0.108781	-0.978255
C	-1.810309	0.120535	2.696612
C	-2.562267	-1.363752	0.452921
C	-3.458172	-0.110059	0.701839
C	-3.162821	0.528583	2.076656
C	-4.864691	-0.671231	0.596869
C	-4.029217	-1.234730	-1.539889
C	-2.738334	-1.852162	-0.994884
H	-1.924809	-0.843720	3.216844
H	0.620017	1.460983	2.224056
H	-3.974698	0.274937	2.772130
H	-4.408497	-1.734629	-2.438056
O	2.168237	-0.506711	0.618043
C	0.969177	0.489198	-2.335748
H	2.018960	0.181590	-2.469363
H	0.380115	0.012277	-3.128451
H	0.923066	1.577170	-2.508423
C	-1.630069	-1.136996	-1.810040
H	-1.161481	-1.776926	-2.571180
H	-1.507917	0.859622	3.452154
H	-3.175379	1.621970	1.972301
O	-5.728526	-0.652548	1.432668
O	-5.097939	-1.245633	-0.597285
C	-3.355964	0.916840	-0.480028
H	-2.345197	1.341735	-0.459297
C	-3.509521	0.178407	-1.833092
H	-4.146826	0.744357	-2.525329
H	-2.693779	-2.943030	-1.079094
H	-2.857510	-2.131797	1.182320
C	-4.364303	2.059099	-0.373924
H	-5.400728	1.703072	-0.493058
H	-4.301109	2.590275	0.585980
H	-4.175942	2.793210	-1.171863
O	-2.286072	-0.062538	-2.488347
O	-0.362781	-2.302199	0.801946
C	-0.289440	-2.806024	2.106631
H	-1.284075	-3.022265	2.540157
H	0.271319	-3.749064	2.048798
H	0.255490	-2.126971	2.785885
C	2.091609	1.800640	0.000095
H	2.681257	1.665666	-0.923287
N	3.840225	1.872637	1.784926
N	3.085151	1.847383	0.985785
Si	1.210714	3.587169	-0.109639
C	2.153079	4.460217	-1.459561
H	3.227734	4.517644	-1.222913
H	2.038038	3.946514	-2.427091
H	1.776100	5.489505	-1.575290
C	-0.590288	3.340624	-0.508139
H	-1.036696	4.339194	-0.652595
H	-0.758636	2.760125	-1.426223
H	-1.113118	2.854301	0.327569
C	1.432960	4.404247	1.554415
H	0.941386	5.391024	1.525534

H	0.964780	3.824943	2.365669
H	2.491985	4.574413	1.805907
Al	3.241730	-1.646898	-0.151880
Cl	2.422608	-2.798148	-1.782592
Cl	3.954139	-2.957533	1.406369
Cl	4.875662	-0.417863	-0.931829

65

26a-Al'_11 Eopt -3290.918758

C	1.067335	0.607891	-0.236427
C	0.250948	1.225323	0.897753
C	-0.875555	0.662753	1.333642
C	-1.337487	-0.612451	0.673256
C	-0.987255	-0.630156	-0.792080
C	0.119453	-0.055670	-1.268126
C	-1.816929	1.223000	2.355917
C	-2.865711	-0.760591	0.696165
C	-3.622431	0.601773	0.597088
C	-3.165226	1.583252	1.698714
C	-5.075609	0.184092	0.741180
C	-4.467503	-1.066869	-1.167468
C	-3.197499	-1.632540	-0.524824
H	-1.984406	0.470216	3.141895
H	0.610784	2.137421	1.376349
H	-3.943919	1.645308	2.471294
H	-4.959096	-1.765254	-1.853722
O	1.999189	-0.278627	0.234678
C	0.488469	-0.131413	-2.721272
H	-0.320361	-0.590176	-3.301934
H	0.667862	0.860480	-3.166966
H	1.387475	-0.750774	-2.868622
C	-2.097147	-1.312768	-1.567046
H	-1.743786	-2.195267	-2.118789
H	-1.390509	2.110729	2.844426
H	-3.075788	2.588632	1.265009
O	-5.861772	0.534247	1.580201
O	-5.454186	-0.685725	-0.212928
C	-3.538038	1.222691	-0.843695
H	-2.503392	1.558488	-1.006059
C	-3.849347	0.131667	-1.900347
H	-4.484884	0.528567	-2.702913
H	-3.257839	-2.697728	-0.278125
H	-3.177133	-1.238701	1.635686
C	-4.476133	2.416314	-1.024251
H	-4.306629	2.877168	-2.008885
H	-5.534740	2.112263	-0.984063
H	-4.321560	3.192818	-0.262132
O	-2.708172	-0.425877	-2.507176
O	-0.725404	-1.779319	1.211304
C	-0.719876	-1.929304	2.606089
H	-0.265358	-2.905809	2.816643
H	-0.111398	-1.155068	3.105478
H	-1.737007	-1.918701	3.039896
C	1.904671	1.794893	-0.879280
H	1.296212	2.491923	-1.480862
N	3.622312	0.780903	-2.387703
N	2.826995	1.230697	-1.772931
Si	3.042430	2.900342	0.359474
C	2.016541	4.406619	0.765332
H	1.119718	4.190995	1.364016
H	2.646929	5.099678	1.347313
H	1.705279	4.929631	-0.152913
C	3.567615	1.872861	1.817528
H	2.724702	1.487184	2.406939
H	4.171313	1.017190	1.479635
H	4.193603	2.503377	2.471688
C	4.523534	3.397057	-0.667537

H	5.107477	4.128505	-0.084112
H	5.194415	2.550679	-0.885430
H	4.240763	3.883339	-1.615114
Al	2.717243	-1.855507	0.208894
Cl	2.754927	-2.740906	2.178489
Cl	1.863590	-3.210124	-1.245755
Cl	4.787941	-1.450125	-0.380377

65

26a-Al'_12 Eopt -3290.924192

C	1.245504	0.242222	0.341679
C	0.339155	0.239900	1.563491
C	-0.777391	-0.481078	1.645175
C	-1.116164	-1.403840	0.504497
C	-0.629132	-0.870592	-0.817749
C	0.505634	-0.176085	-0.943142
C	-1.812794	-0.405313	2.725915
C	-2.629302	-1.588250	0.321181
C	-3.443327	-0.314594	0.703012
C	-3.150486	0.117154	2.156243
C	-4.886484	-0.743407	0.503475
C	-4.038281	-1.126564	-1.664118
C	-2.814603	-1.906264	-1.172863
H	-1.969314	-1.410236	3.147657
H	0.627610	0.883001	2.401900
H	-3.974226	-0.221523	2.799027
H	-4.428302	-1.485353	-2.623052
O	2.366607	-0.505759	0.601365
C	1.107927	0.121272	-2.288310
H	1.501205	1.140391	-2.394280
H	1.942008	-0.570865	-2.486727
H	0.368196	-0.018852	-3.085940
C	-1.637341	-1.208012	-1.897786
H	-1.217570	-1.796684	-2.724673
H	-1.478721	0.241716	3.549425
H	-3.152398	1.213673	2.212074
O	-5.769831	-0.747263	1.318548
O	-5.130318	-1.159659	-0.751923
C	-3.217438	0.830052	-0.347143
H	-2.172791	1.155430	-0.254587
C	-3.401609	0.263100	-1.781052
H	-3.975815	0.955603	-2.410355
H	-2.861659	-2.981917	-1.372551
H	-2.983297	-2.410718	0.958678
C	-4.119883	2.041941	-0.128973
H	-4.023356	2.462582	0.881535
H	-3.853105	2.834336	-0.844445
H	-5.182185	1.796692	-0.291219
O	-2.191668	-0.008242	-2.452439
O	-0.482716	-2.677223	0.604975
C	-0.539269	-3.347947	1.834951
H	-0.053672	-4.322044	1.685152
H	0.008904	-2.809915	2.628750
H	-1.573680	-3.532103	2.179696
C	1.714313	1.758985	0.121183
H	2.386365	1.803442	-0.755853
N	3.250737	2.269951	2.025282
N	2.587468	2.053261	1.174100
Si	0.427007	3.265489	-0.088714
C	-0.754196	3.292217	1.352720
H	-1.397395	2.403003	1.385777
H	-0.223128	3.375001	2.314096
H	-1.398362	4.181806	1.249003
C	1.529499	4.773063	-0.079396
H	2.310510	4.712642	-0.854194
H	0.917599	5.665983	-0.289675
H	2.015156	4.931114	0.897212

C	-0.416458	3.045260	-1.733932
H	-1.170073	3.846796	-1.820161
H	0.293709	3.168924	-2.565956
H	-0.929151	2.079357	-1.852253
Al	3.825690	-1.112512	-0.136440
Cl	3.442944	-2.750535	-1.487609
Cl	5.128988	-1.734648	1.462865
Cl	4.744751	0.547412	-1.217760

65

26a-Al'_13 Eopt -3290.925745

C	1.276078	0.564139	-0.376674
C	0.719896	0.183188	0.984259
C	-0.292582	-0.667045	1.148116
C	-0.829395	-1.379639	-0.064025
C	-0.727681	-0.537942	-1.309849
C	0.292484	0.295890	-1.525800
C	-1.021431	-0.953209	2.425246
C	-2.317892	-1.732666	0.056669
C	-3.114505	-0.693475	0.900501
C	-2.510219	-0.550126	2.313448
C	-4.523978	-1.257579	0.929346
C	-4.207037	-1.060562	-1.399123
C	-2.842651	-1.756314	-1.391001
H	-0.957459	-2.031679	2.638178
H	1.169212	0.665845	1.857937
H	-3.101181	-1.158853	3.011006
H	-4.791248	-1.256935	-2.305029
O	2.503893	0.017955	-0.648311
C	0.585684	0.921349	-2.857671
H	0.375213	2.000839	-2.892129
H	1.652306	0.780372	-3.095285
H	-0.003115	0.452256	-3.656157
C	-1.942357	-0.773033	-2.180359
H	-1.702658	-1.110437	-3.198271
H	-0.553039	-0.431349	3.271671
H	-2.624407	0.488910	2.648855
O	-5.177256	-1.547712	1.895616
O	-5.035285	-1.430301	-0.302562
C	-3.242263	0.666439	0.127968
H	-2.234260	1.096218	0.060479
C	-3.730288	0.394513	-1.321855
H	-4.495714	1.121274	-1.624015
H	-2.853533	-2.764136	-1.819298
H	-2.429227	-2.710506	0.546058
C	-4.150175	1.679894	0.819803
H	-3.829621	1.899276	1.847699
H	-4.139073	2.627418	0.260369
H	-5.196072	1.334359	0.859506
O	-2.707206	0.434938	-2.291586
O	-0.081505	-2.546801	-0.398696
C	0.209150	-3.463518	0.623658
H	0.731372	-4.305650	0.149662
H	0.878658	-3.033853	1.388541
H	-0.698955	-3.857063	1.117182
C	1.503735	2.152266	-0.348613
H	1.868099	2.475212	-1.339594
N	3.476347	2.482878	1.152395
N	2.618340	2.359272	0.474276
Si	0.087223	3.458299	0.156214
C	-1.153933	3.468127	-1.232474
H	-0.717655	3.909542	-2.142278
H	-1.560481	2.477805	-1.485602
H	-1.992161	4.114858	-0.922063
C	-0.634038	3.020604	1.818178
H	-1.336964	3.821852	2.102912
H	-1.181334	2.069020	1.810211

H	0.143538	2.968419	2.596642
C	1.016041	5.077362	0.244551
H	0.297867	5.892327	0.434455
H	1.754873	5.094419	1.062029
H	1.534578	5.302232	-0.701042
Al	3.711101	-1.101796	-0.067438
Cl	5.581317	-0.006520	-0.218904
Cl	3.786849	-2.872077	-1.296524
Cl	3.435787	-1.647078	2.014187

65

26a-Al'_14 Eopt -3290.926057

C	1.256072	0.379427	0.342529
C	0.338241	0.463851	1.550151
C	-0.751280	-0.285849	1.692153
C	-1.040900	-1.336965	0.654173
C	-0.552587	-0.925077	-0.714689
C	0.555803	-0.199209	-0.897380
C	-1.756653	-0.237333	2.796171
C	-2.548904	-1.600768	0.465907
C	-3.409499	-0.343731	0.801020
C	-3.151852	0.131146	2.245805
C	-4.841114	-0.809225	0.600071
C	-3.966472	-1.236295	-1.544386
C	-2.736552	-1.979319	-1.016825
H	-1.795478	-1.238927	3.254959
H	0.626414	1.163832	2.341050
H	-3.925158	-0.294685	2.899170
H	-4.336436	-1.632828	-2.496419
O	2.406679	-0.281750	0.692929
C	1.135755	0.064764	-2.258185
H	2.234076	-0.002507	-2.244228
H	0.772435	-0.668516	-2.989069
H	0.879611	1.063195	-2.640707
C	-1.567105	-1.303024	-1.771948
H	-1.154040	-1.911874	-2.589041
H	-1.458526	0.470472	3.582196
H	-3.279196	1.220799	2.294954
O	-5.731175	-0.806492	1.407606
O	-5.067392	-1.262909	-0.645241
C	-3.192899	0.773247	-0.281330
H	-2.155890	1.120752	-0.185236
C	-3.350372	0.157412	-1.699014
H	-3.928451	0.820642	-2.355754
H	-2.780951	-3.061744	-1.177354
H	-2.859299	-2.411040	1.141199
C	-4.119253	1.975031	-0.114687
H	-3.865752	2.742099	-0.861784
H	-5.176344	1.703224	-0.268482
H	-4.032704	2.439091	0.877356
O	-2.130714	-0.118232	-2.352521
O	-0.357429	-2.484770	1.143813
C	-0.365590	-3.641682	0.350260
H	0.338221	-4.348105	0.811225
H	-1.359528	-4.123776	0.312802
H	-0.020863	-3.448567	-0.680786
C	1.673104	1.884798	-0.031781
H	2.301578	1.879370	-0.940879
N	3.245846	2.572157	1.785698
N	2.574195	2.277527	0.965482
Si	0.354501	3.365620	-0.269858
C	1.448283	4.870755	-0.440809
H	2.165384	4.759001	-1.269851
H	0.817401	5.748330	-0.659520
H	2.010710	5.092139	0.480768
C	-0.598665	3.037594	-1.834878
H	-1.063647	2.041769	-1.881556

H	-1.400117	3.794073	-1.888202
H	0.038471	3.170062	-2.723084
C	-0.738543	3.504493	1.234793
H	-0.155269	3.612165	2.162769
H	-1.348316	4.416734	1.117982
H	-1.420202	2.650173	1.347208
Al	3.762532	-1.138715	0.008232
Cl	4.854694	0.280408	-1.239037
Cl	5.006289	-1.762880	1.652740
Cl	3.165004	-2.838677	-1.183960

65

26a-Al'_2 Eopt -3290.923957

C	1.232006	0.416119	0.329386
C	0.284344	0.672390	1.497534
C	-0.830980	-0.040847	1.648926
C	-1.123335	-1.125211	0.637964
C	-0.653153	-0.723364	-0.744873
C	0.456049	-0.001516	-0.933990
C	-1.830971	0.038945	2.756838
C	-2.632063	-1.409158	0.464732
C	-3.520226	-0.172764	0.799632
C	-3.256482	0.322405	2.234652
C	-4.941338	-0.675779	0.619850
C	-4.081090	-1.095487	-1.532751
C	-2.827310	-1.802096	-1.013184
H	-1.818542	-0.940786	3.263290
H	0.581110	1.405706	2.255525
H	-3.988162	-0.145765	2.906804
H	-4.451925	-1.509646	-2.476990
O	2.167248	-0.525421	0.700557
C	0.980900	0.362751	-2.291389
H	0.423485	-0.148654	-3.085296
H	0.910163	1.444986	-2.489901
H	2.041026	0.080534	-2.397193
C	-1.684678	-1.100617	-1.785314
H	-1.272443	-1.704548	-2.606682
H	-1.540834	0.790826	3.503992
H	-3.448530	1.402278	2.283773
O	-5.821835	-0.689587	1.438044
O	-5.171314	-1.142142	-0.620313
C	-3.345659	0.938731	-0.292549
H	-2.315921	1.305405	-0.216338
C	-3.501788	0.312484	-1.702863
H	-4.103202	0.956286	-2.358150
H	-2.844091	-2.886436	-1.166247
H	-2.918431	-2.221368	1.148458
C	-4.292218	2.124371	-0.124017
H	-4.066328	2.885947	-0.885774
H	-5.346897	1.831029	-0.254305
H	-4.196071	2.604808	0.859348
O	-2.278557	0.069866	-2.360994
O	-0.437708	-2.257746	1.154082
C	-0.435876	-3.428052	0.381597
H	-1.427083	-3.916823	0.349075
H	-0.084788	-3.249452	-0.649311
H	0.269452	-4.122306	0.859086
C	2.034867	1.757365	0.011896
H	2.676254	1.587342	-0.870823
N	3.686780	1.976953	1.875142
N	2.975382	1.882472	1.041673
Si	1.145434	3.525728	-0.241912
C	2.063247	4.275003	-1.680772
H	3.142099	4.351938	-1.470773
H	1.930083	3.681090	-2.598811
H	1.682714	5.290938	-1.876436
C	-0.663301	3.261217	-0.584825

H	-1.189405	2.954882	0.330369
H	-1.083581	4.228519	-0.908688
H	-0.860092	2.524527	-1.376779
C	1.404313	4.480994	1.341251
H	2.466904	4.694044	1.539849
H	0.887377	5.450850	1.251943
H	0.978535	3.958758	2.213147
Al	3.397126	-1.537227	-0.011285
Cl	4.886751	-0.177702	-0.859668
Cl	4.265204	-2.689003	1.587474
Cl	2.698577	-2.835893	-1.591206

65

26a-Al'_3 Eopt -3290.919997

C	1.144920	0.327716	-0.358474
C	0.509982	0.398522	1.013233
C	-0.607823	-0.234550	1.362694
C	-1.189130	-1.273268	0.446667
C	-0.852567	-1.016667	-0.998582
C	0.277849	-0.416457	-1.387603
C	-1.418885	-0.022745	2.598201
C	-2.727660	-1.314096	0.489165
C	-3.327059	0.081774	0.840433
C	-2.781190	0.587328	2.195284
C	-4.825245	-0.164318	0.880154
C	-4.355316	-0.845465	-1.326800
C	-3.186058	-1.744870	-0.917385
H	-1.574175	-0.997066	3.087916
H	1.013076	1.035226	1.741604
H	-3.523490	0.375378	2.976645
H	-4.920231	-1.231364	-2.182644
O	2.456434	-0.062764	-0.343556
C	0.685589	-0.334513	-2.830553
H	0.507859	0.657994	-3.276457
H	1.756112	-0.572563	-2.930332
H	0.129310	-1.058353	-3.438954
C	-2.052398	-1.310303	-1.877359
H	-1.859951	-2.025090	-2.690310
H	-0.908067	0.632429	3.317427
H	-2.676699	1.680252	2.156122
O	-5.573399	0.020939	1.802144
O	-5.299904	-0.645378	-0.282595
C	-3.122191	1.091236	-0.348418
H	-2.046183	1.306352	-0.424392
C	-3.565569	0.419648	-1.678904
H	-4.128202	1.125501	-2.304104
H	-3.416715	-2.813714	-0.980335
H	-3.049732	-2.024504	1.263893
C	-3.867923	2.409242	-0.145108
H	-4.960676	2.266823	-0.164453
H	-3.611486	2.895073	0.806569
H	-3.616248	3.109405	-0.955870
O	-2.512149	-0.086189	-2.467441
O	-0.613759	-2.482620	0.927937
C	-0.879887	-3.666811	0.225873
H	-0.631806	-3.586825	-0.847370
H	-0.239004	-4.444636	0.662133
H	-1.931090	-3.993068	0.327302
C	1.172023	1.862193	-0.867886
H	0.246655	2.144686	-1.399732
N	3.089522	2.002574	-2.458368
N	2.200078	1.949882	-1.811011
Si	1.523841	3.352641	0.415719
C	1.863327	4.814145	-0.699545
H	1.963963	5.715206	-0.071692
H	2.798955	4.711667	-1.271769
H	1.035849	4.991813	-1.405209

C	-0.065202	3.625143	1.356517
H	-0.931491	3.603803	0.675892
H	-0.229430	2.894669	2.159899
H	-0.025593	4.629369	1.810728
C	3.034896	2.897456	1.405904
H	2.882856	2.041109	2.077690
H	3.873788	2.656120	0.733369
H	3.336117	3.763481	2.018508
Al	3.398631	-1.398666	0.282265
Cl	5.434626	-0.912031	-0.272382
Cl	2.803480	-3.253635	-0.659635
Cl	3.252945	-1.533430	2.435964

65

26a-Al'_4 Eopt -3290.929590

C	1.213243	0.334530	-0.351168
C	0.645628	0.008967	1.022664
C	-0.404661	-0.790643	1.216873
C	-0.998945	-1.530776	0.052277
C	-0.813251	-0.788508	-1.244957
C	0.236133	0.004260	-1.493157
C	-1.154790	-1.005849	2.494196
C	-2.506168	-1.751323	0.177178
C	-3.222485	-0.619426	0.957839
C	-2.597982	-0.458753	2.362863
C	-4.668763	-1.078770	1.008572
C	-4.330872	-1.036876	-1.328532
C	-3.024618	-1.833551	-1.268981
H	-1.190064	-2.088751	2.696209
H	1.117816	0.495775	1.882476
H	-3.242777	-0.965426	3.093791
H	-4.927350	-1.239520	-2.224974
O	2.441363	-0.218468	-0.602821
C	0.556114	0.550267	-2.853769
H	0.372477	1.631573	-2.944460
H	1.621572	0.374408	-3.071667
H	-0.037232	0.055034	-3.632682
C	-2.045119	-0.979425	-2.109974
H	-1.838613	-1.390651	-3.107960
H	-0.650256	-0.529322	3.346422
H	-2.600259	0.603760	2.641366
O	-5.345611	-1.266262	1.983590
O	-5.186737	-1.279500	-0.217071
C	-3.255380	0.699195	0.110816
H	-2.222308	1.060136	0.034192
C	-3.742106	0.378210	-1.329998
H	-4.443214	1.143597	-1.687623
H	-3.112491	-2.864699	-1.626870
H	-2.666893	-2.692445	0.719405
C	-4.109110	1.798635	0.736204
H	-4.014048	2.724186	0.148740
H	-5.177382	1.527493	0.750609
H	-3.807415	2.026470	1.768013
O	-2.708372	0.280543	-2.286501
O	-0.497063	-2.864904	-0.051046
C	0.882074	-3.051507	-0.231223
H	1.034693	-4.129737	-0.377452
H	1.268979	-2.521707	-1.116565
H	1.462154	-2.735941	0.652911
C	1.434292	1.928289	-0.399369
H	1.852672	2.187205	-1.387661
N	3.293528	2.406248	1.203300
N	2.494995	2.202687	0.473138
Si	0.030965	3.293156	-0.020693
C	-1.173395	3.260345	-1.438921
H	-1.577019	2.266026	-1.677740
H	-2.015759	3.922191	-1.175222

H	-0.703057	3.673077	-2.345384
C	-0.691949	2.968743	1.666007
H	0.092095	2.995232	2.439822
H	-1.409997	3.774255	1.894455
H	-1.214910	2.007211	1.738349
C	0.992223	4.896499	-0.009939
H	1.554503	5.044322	-0.945810
H	0.279519	5.732764	0.085705
H	1.692675	4.967754	0.837664
Al	3.890437	-0.954394	0.027675
Cl	5.487872	0.484028	-0.285435
Cl	4.325882	-2.764730	-1.058995
Cl	3.717053	-1.345201	2.150582

65

26a-Al'_5 Eopt -3290.916142

C	-1.152933	0.201675	0.579293
C	-0.596845	0.067956	-0.828879
C	0.534954	-0.581533	-1.112250
C	1.223609	-1.353634	-0.016827
C	1.025021	-0.720708	1.332683
C	-0.086936	-0.059931	1.659914
C	1.284066	-0.546416	-2.409969
C	2.744909	-1.420944	-0.192634
C	3.334083	-0.139813	-0.852166
C	2.654146	0.144327	-2.210658
C	4.809316	-0.471832	-0.989895
C	4.546791	-0.708218	1.345771
C	3.302643	-1.593589	1.230129
H	1.447935	-1.574755	-2.766868
H	-1.158002	0.560707	-1.626340
H	3.332564	-0.163452	-3.018019
H	5.186248	-0.960552	2.198935
O	-2.255737	-0.578013	0.828256
C	-0.364329	0.412833	3.059090
H	-0.041799	1.454033	3.224410
H	-1.440480	0.346963	3.282406
H	0.168530	-0.203264	3.795657
C	2.280182	-0.896435	2.163016
H	2.108040	-1.428014	3.109834
H	0.711403	-0.020225	-3.186902
H	2.518403	1.228223	-2.321978
O	5.465012	-0.502610	-1.996778
O	5.382060	-0.760428	0.193012
C	3.296242	1.079668	0.135770
H	2.244304	1.354796	0.284952
C	3.854110	0.649037	1.518017
H	4.509464	1.423167	1.938755
H	3.475273	-2.643202	1.490438
H	3.010016	-2.270444	-0.836586
C	4.045984	2.299904	-0.394055
H	3.677644	2.624157	-1.377406
H	3.922361	3.144061	0.300947
H	5.127497	2.107947	-0.486018
O	2.859203	0.374422	2.477552
O	0.699371	-2.669103	0.143855
C	0.637498	-3.494114	-0.989704
H	0.265721	-4.469949	-0.648991
H	-0.064534	-3.109376	-1.747893
H	1.625501	-3.650898	-1.460877
C	-1.590094	1.767985	0.760355
H	-1.115194	2.194670	1.659279
N	-4.031350	1.826460	1.250187
N	-2.945466	1.810762	1.067908
Si	-1.246806	3.248182	-0.537699
C	-2.247714	3.045005	-2.094380
H	-3.306387	2.840645	-1.872941

H	-2.198437	3.999085	-2.646277
H	-1.874792	2.253056	-2.759173
C	-1.842214	4.736378	0.423293
H	-1.306812	4.850191	1.379128
H	-1.661792	5.645640	-0.173756
H	-2.923713	4.690623	0.630778
C	0.594282	3.281695	-0.789393
H	0.948263	2.404581	-1.347249
H	0.846302	4.184908	-1.369838
H	1.127517	3.339036	0.171654
Al	-3.514201	-1.384142	-0.090679
Cl	-4.245337	0.034595	-1.572569
Cl	-5.077764	-1.792734	1.339845
Cl	-2.918272	-3.195010	-1.088829

65

26a-Al'_6 Eopt -3290.919805

C	-1.060085	0.348967	0.528048
C	-0.468380	0.314699	-0.868359
C	0.662476	-0.318025	-1.178455
C	1.326360	-1.167695	-0.131597
C	1.097945	-0.643216	1.259022
C	-0.030325	-0.024245	1.614001
C	1.422974	-0.204169	-2.463278
C	2.849629	-1.213622	-0.289783
C	3.432662	0.121406	-0.847868
C	2.769909	0.502357	-2.192010
C	4.913321	-0.185426	-0.985526
C	4.618645	-0.584012	1.323599
C	3.390576	-1.477706	1.125550
H	1.612962	-1.207865	-2.872739
H	-1.014643	0.835242	-1.654688
H	3.468982	0.283664	-3.010793
H	5.248252	-0.886398	2.167764
O	-2.213698	-0.376543	0.664180
C	-0.348251	0.311095	3.043178
H	0.363997	-0.164407	3.728892
H	-0.316495	1.392723	3.253897
H	-1.352230	-0.059697	3.304307
C	2.345183	-0.864679	2.091193
H	2.172801	-1.466773	2.994324
H	0.852291	0.353239	-3.219387
H	2.597572	1.587287	-2.210510
O	5.583111	-0.143537	-1.982531
O	5.472247	-0.545820	0.184117
C	3.367742	1.271666	0.220975
H	2.310784	1.542038	0.360782
C	3.902425	0.748220	1.581257
H	4.538844	1.498544	2.068880
H	3.576994	-2.540256	1.313972
H	3.127877	-2.014970	-0.988976
C	4.137546	2.519860	-0.209341
H	3.808427	2.899580	-1.186775
H	3.987245	3.322910	0.527763
H	5.221577	2.330583	-0.269885
O	2.898036	0.390398	2.501699
O	0.797513	-2.490366	-0.081038
C	0.633806	-3.189667	-1.286739
H	0.276404	-4.193549	-1.020149
H	-0.122635	-2.720911	-1.939846
H	1.578801	-3.299368	-1.850540
C	-1.476489	1.886087	0.784268
H	-0.661707	2.475898	1.237931
N	-3.395186	1.779893	2.375820
N	-2.506831	1.857037	1.729713
Si	-2.176520	3.052480	-0.682816
C	-3.535572	2.125118	-1.551329

H	-3.181694	1.241903	-2.102747
H	-4.300865	1.789023	-0.833340
H	-4.025654	2.802792	-2.270185
C	-2.836086	4.537594	0.240900
H	-3.181903	5.280737	-0.496944
H	-3.695444	4.299433	0.887229
H	-2.055566	5.016454	0.853753
C	-0.704100	3.547341	-1.718421
H	0.171105	3.766907	-1.086078
H	-0.416188	2.791094	-2.461663
H	-0.964148	4.472520	-2.259306
Al	-3.224744	-1.574138	-0.099388
Cl	-2.961525	-1.670926	-2.252276
Cl	-5.247916	-0.919416	0.340302
Cl	-2.889842	-3.527455	0.754367

65

26a-Al'_7 Eopt -3290.924307

C	-1.244529	0.271263	-0.307674
C	-0.344414	0.277281	-1.531486
C	0.762291	-0.457231	-1.628682
C	1.095192	-1.399270	-0.500662
C	0.614815	-0.882440	0.830320
C	-0.516413	-0.186118	0.967040
C	1.789144	-0.387183	-2.718015
C	2.606865	-1.602470	-0.322671
C	3.434715	-0.338071	-0.705114
C	3.144250	0.099120	-2.156745
C	4.873723	-0.781307	-0.508457
C	4.025289	-1.158175	1.660181
C	2.794822	-1.927519	1.170714
H	1.920543	-1.389255	-3.154651
H	-0.639874	0.932547	-2.357193
H	3.954526	-0.262419	-2.804140
H	4.413778	-1.520602	2.618388
O	-2.377966	-0.448234	-0.585579
C	-1.109414	0.143123	2.308151
H	-0.764242	-0.564337	3.073083
H	-0.840284	1.152221	2.652567
H	-2.208234	0.088220	2.288319
C	1.625340	-1.223529	1.902544
H	1.205823	-1.805868	2.734786
H	1.459689	0.276929	-3.529662
H	3.176449	1.195148	-2.213846
O	5.755402	-0.793568	-1.325273
O	5.115850	-1.199911	0.746445
C	3.220974	0.807473	0.345673
H	2.179416	1.141674	0.251709
C	3.399779	0.236634	1.778960
H	3.980881	0.923212	2.408514
H	2.835142	-3.004279	1.366166
H	2.948155	-2.427604	-0.963690
C	4.135153	2.010970	0.130085
H	4.036761	2.440471	-0.876537
H	3.882234	2.800853	0.853226
H	5.195771	1.753038	0.283476
O	2.187837	-0.023817	2.450670
O	0.448105	-2.664581	-0.617010
C	0.495594	-3.321273	-1.854870
H	1.527573	-3.508864	-2.205082
H	0.003488	-4.293522	-1.714555
H	-0.051511	-2.770307	-2.640514
C	-1.682280	1.794706	-0.044238
H	-2.272079	1.851073	0.889090
N	-3.353106	2.311751	-1.829341
N	-2.633188	2.094396	-1.026123
Si	-0.381153	3.305170	0.050558

C	0.553256	3.125231	1.651387
H	-0.101727	3.317843	2.515382
H	1.034208	2.145320	1.786723
H	1.341016	3.897691	1.655977
C	0.726908	3.319839	-1.448500
H	0.151855	3.362369	-2.386803
H	1.344830	4.232424	-1.395097
H	1.398630	2.451942	-1.490224
C	-1.484086	4.813178	0.076929
H	-0.858796	5.707706	0.234590
H	-2.027734	4.956874	-0.870915
H	-2.216667	4.768912	0.898705
Al	-3.817072	-1.126965	0.126032
Cl	-5.015061	-1.900404	-1.489315
Cl	-3.386876	-2.667294	1.569502
Cl	-4.873542	0.527770	1.085165

65

26a-Al'_8 Eopt -3290.921572

C	1.292092	0.587398	-0.352655
C	0.655219	0.379046	1.015679
C	-0.384153	-0.438043	1.181161
C	-0.906638	-1.177683	-0.026269
C	-0.791233	-0.334269	-1.276993
C	0.244095	0.487270	-1.478306
C	-1.067882	-0.802376	2.458104
C	-2.405784	-1.532951	0.072093
C	-3.194261	-0.554271	0.992077
C	-2.586784	-0.524456	2.407586
C	-4.611564	-1.097723	0.989052
C	-4.306679	-0.732854	-1.318614
C	-2.959206	-1.456062	-1.365540
H	-0.897576	-1.882327	2.603897
H	1.127722	0.852438	1.882976
H	-3.111288	-1.264127	3.027301
H	-4.896172	-0.856153	-2.234022
O	2.333016	-0.277651	-0.598493
C	0.457703	1.246475	-2.752949
H	-0.254200	0.936739	-3.527883
H	0.339901	2.335256	-2.625304
H	1.475132	1.065690	-3.137266
C	-2.048028	-0.452194	-2.111248
H	-1.857215	-0.725462	-3.159347
H	-0.611110	-0.288725	3.315431
H	-2.790298	0.452209	2.866073
O	-5.262122	-1.449970	1.936342
O	-5.137621	-1.162347	-0.247178
C	-3.301258	0.856481	0.317933
H	-2.286247	1.265026	0.269715
C	-3.795743	0.700422	-1.143948
H	-4.543552	1.464811	-1.392988
H	-3.008602	-2.434410	-1.855327
H	-2.504192	-2.541568	0.498757
C	-4.181252	1.842883	1.080591
H	-3.848835	1.989917	2.117423
H	-4.150367	2.823801	0.582373
H	-5.234852	1.519687	1.106171
O	-2.770238	0.786788	-2.109014
O	-0.097024	-2.346477	-0.085674
C	-0.329608	-3.247434	-1.135507
H	0.465353	-4.001698	-1.083736
H	-1.303218	-3.761581	-1.044093
H	-0.271766	-2.761875	-2.126758
C	1.946553	2.040917	-0.401337
H	2.384359	2.184858	-1.403563
N	3.940288	1.912417	1.107278
N	3.080786	1.971294	0.422967

Si	0.965149	3.718299	0.054924
C	1.513670	4.918621	-1.262069
H	1.055511	5.904813	-1.081004
H	2.607866	5.047627	-1.255004
H	1.210046	4.584507	-2.266712
C	-0.863446	3.386428	0.036210
H	-1.379983	4.360500	0.070248
H	-1.210789	2.847917	-0.857139
H	-1.152934	2.821919	0.933955
C	1.539170	4.225276	1.757411
H	0.971333	5.119035	2.064863
H	1.350453	3.439724	2.506770
H	2.607700	4.491444	1.784286
Al	3.261270	-1.609706	0.044724
Cl	2.799902	-2.193538	2.074272
Cl	3.222376	-3.295080	-1.310451
Cl	5.309706	-0.842802	0.065309

65

26a-Al'_9 Eopt -3290.926714

C	-1.000182	0.052515	0.510666
C	-0.441545	0.064387	-0.896043
C	0.694543	-0.532601	-1.254640
C	1.397115	-1.437376	-0.286554
C	1.118285	-1.083880	1.146548
C	-0.023844	-0.519349	1.551638
C	1.441488	-0.349899	-2.536137
C	2.917777	-1.387023	-0.422489
C	3.418311	0.009040	-0.887200
C	2.757547	0.404074	-2.229308
C	4.921436	-0.172005	-1.009681
C	4.619005	-0.753954	1.257360
C	3.469782	-1.725527	0.973372
H	1.667646	-1.339743	-2.963524
H	-1.016445	0.619945	-1.638386
H	3.475946	0.233310	-3.042600
H	5.255132	-1.062436	2.094454
O	-2.238577	-0.510284	0.618209
C	-0.373058	-0.337210	3.001243
H	-1.423880	-0.618198	3.174065
H	0.250882	-0.974188	3.640702
H	-0.232479	0.698536	3.352315
C	2.374738	-1.280581	1.972216
H	2.257138	-1.952902	2.833752
H	0.852869	0.205766	-3.279531
H	2.545578	1.482040	-2.222428
O	5.603582	-0.001096	-1.984083
O	5.486681	-0.571272	0.143810
C	3.239986	1.073673	0.254285
H	2.161721	1.245608	0.387449
C	3.793749	0.496123	1.588061
H	4.360424	1.259268	2.137916
H	3.742292	-2.781429	1.074346
H	3.223701	-2.128221	-1.172681
C	3.906436	2.409216	-0.070407
H	3.569896	2.820844	-1.032387
H	3.667986	3.146612	0.710858
H	5.004136	2.318721	-0.110261
O	2.812555	-0.007236	2.466158
O	1.069293	-2.807576	-0.533964
C	-0.284123	-3.181737	-0.464958
H	-0.878906	-2.758129	-1.292319
H	-0.310324	-4.277259	-0.543887
H	-0.756134	-2.892741	0.489205
C	-1.144906	1.623574	0.906596
H	-0.258029	1.982440	1.456884
N	-3.129193	1.736105	2.410263

N	-2.210322	1.711298	1.803099
Si	-1.444538	3.065228	-0.441017
C	0.168432	3.258569	-1.358768
H	0.323600	2.489420	-2.127097
H	0.162645	4.242894	-1.856264
H	1.022930	3.243151	-0.663608
C	-2.943282	2.637595	-1.457187
H	-3.201179	3.505187	-2.087350
H	-2.805388	1.768294	-2.115880
H	-3.806469	2.430551	-0.804179
C	-1.768481	4.563463	0.628959
H	-2.706630	4.482782	1.201034
H	-0.942195	4.752789	1.332763
H	-1.860070	5.447804	-0.023491
Al	-3.610307	-1.153933	-0.244641
Cl	-5.288358	0.110942	0.308586
Cl	-3.955213	-3.176169	0.426710
Cl	-3.358534	-1.137210	-2.396196

65

26a-Al_1 Eopt -3290.936701

C	-1.284087	0.227656	0.274579
C	-0.426246	-0.281119	1.429626
C	0.814303	0.159456	1.628011
C	1.376358	1.195720	0.679247
C	0.829399	1.018636	-0.720360
C	-0.406260	0.558136	-0.941249
C	1.756909	-0.247047	2.713631
C	2.906154	1.061867	0.504238
C	3.408433	-0.401490	0.733147
C	3.016756	-0.915193	2.131799
C	4.916596	-0.293512	0.575135
C	4.226823	0.494440	-1.530380
C	3.213299	1.482267	-0.947074
H	2.042853	0.667707	3.259496
H	-0.902167	-0.993494	2.111279
H	3.862709	-0.763283	2.815471
H	4.703993	0.857926	-2.447285
O	-2.314290	-0.616077	-0.027956
C	-0.931736	0.192846	-2.294536
H	-0.368659	0.693020	-3.093158
H	-0.824785	-0.893171	-2.444301
H	-1.998764	0.426140	-2.403336
C	1.927518	1.170585	-1.749476
H	1.691981	1.925859	-2.514303
H	1.268248	-0.917584	3.433617
H	2.851051	-1.999071	2.082706
O	5.757760	-0.572780	1.386989
O	5.279979	0.176971	-0.630179
C	2.946571	-1.346932	-0.435183
H	1.853155	-1.452459	-0.377673
C	3.282847	-0.683843	-1.796559
H	3.689854	-1.418288	-2.503731
H	3.528792	2.528113	-1.028641
H	3.410823	1.705840	1.239456
C	3.563537	-2.742320	-0.360084
H	4.657764	-2.717389	-0.492234
H	3.348633	-3.246178	0.592012
H	3.148447	-3.369166	-1.163197
O	2.186449	-0.061242	-2.424987
O	1.001078	2.444882	1.269839
C	1.391993	3.617864	0.599490
H	0.878239	4.454836	1.092500
H	2.479021	3.796307	0.667988
H	1.099740	3.609235	-0.465654
C	-1.951300	1.592167	0.774331
H	-1.173501	2.255792	1.198004

N	-3.467073	0.844937	2.613510
N	-2.773704	1.219089	1.843887
Si	-3.084275	2.680650	-0.437092
C	-3.799524	3.979731	0.702946
H	-4.441571	3.548445	1.487853
H	-3.006801	4.572693	1.186870
H	-4.423837	4.672216	0.114067
C	-1.934078	3.477998	-1.667638
H	-2.510991	4.237063	-2.222794
H	-1.112932	3.996290	-1.148167
H	-1.507283	2.779029	-2.398569
C	-4.415109	1.578490	-1.130614
H	-4.012498	0.705981	-1.662708
H	-5.059175	1.203286	-0.318752
H	-5.050155	2.156958	-1.821811
Al	-2.484286	-2.356910	-0.121872
Cl	-0.594455	-3.252613	-0.698659
Cl	-3.089802	-3.091566	1.824741
Cl	-4.011268	-2.756558	-1.590845

65

26a-Al_10 Eopt -3290.930557

C	-1.047975	0.086675	-0.078334
C	-0.292103	0.517850	1.164323
C	0.969896	0.937847	1.149126
C	1.660645	1.117255	-0.176502
C	1.162922	0.128658	-1.207775
C	-0.091976	-0.335971	-1.206115
C	1.827595	1.266284	2.327349
C	3.177056	0.849177	-0.093224
C	3.538709	-0.176061	1.028279
C	3.029240	0.302891	2.403307
C	5.056574	-0.241777	0.975579
C	4.540497	-0.897396	-1.225276
C	3.588057	0.276768	-1.464213
H	2.187954	2.301091	2.203181
H	-0.845702	0.404968	2.100061
H	3.856311	0.787536	2.939366
H	5.092600	-1.194875	-2.123739
O	-1.888042	-0.925789	0.270599
C	-0.632885	-1.304502	-2.209198
H	-1.622901	-0.979888	-2.570582
H	0.034364	-1.407569	-3.074073
H	-0.772957	-2.298159	-1.759754
C	2.319659	-0.411592	-2.023942
H	2.190046	-0.280623	-3.108334
H	1.252819	1.229979	3.263030
H	2.741998	-0.570639	3.003203
O	5.830157	0.008764	1.860454
O	5.516528	-0.628966	-0.226812
C	3.064663	-1.626762	0.649198
H	1.965260	-1.642022	0.670412
C	3.512988	-1.952832	-0.800632
H	3.887927	-2.982064	-0.874103
H	3.999134	1.036626	-2.137545
H	3.701609	1.787238	0.139255
C	3.583472	-2.691759	1.614372
H	4.680603	-2.787877	1.568346
H	3.307044	-2.483721	2.657089
H	3.157076	-3.670310	1.348277
O	2.503797	-1.806795	-1.773309
O	1.361466	2.470540	-0.536659
C	1.993170	3.004802	-1.673292
H	3.080409	3.130660	-1.532942
H	1.821844	2.395442	-2.580008
H	1.559925	4.000758	-1.840025
C	-1.992370	1.284223	-0.616203

H	-2.852708	0.813637	-1.132913
N	-0.935836	2.688731	-2.377608
N	-1.392120	2.048388	-1.605194
Si	-2.796747	2.556645	0.684836
C	-3.746789	1.518126	1.897250
H	-4.540442	0.948294	1.391611
H	-3.112319	0.815457	2.454596
H	-4.226460	2.195421	2.623992
C	-1.423491	3.585264	1.423670
H	-0.588562	3.721775	0.717657
H	-1.819960	4.579356	1.688406
H	-1.016815	3.123801	2.335269
C	-3.926129	3.591641	-0.384012
H	-4.474292	4.303103	0.255811
H	-3.371201	4.182048	-1.131345
H	-4.668579	2.968635	-0.907342
Al	-3.333406	-1.874459	0.141931
Cl	-4.172097	-2.171699	2.107685
Cl	-4.758513	-0.785770	-1.112038
Cl	-2.930530	-3.802992	-0.744862

65

26a-Al_2 Eopt -3290.932540

C	1.062288	-0.138204	0.102262
C	0.277367	0.475351	1.258932
C	-0.973275	0.098218	1.530056
C	-1.605348	-0.973390	0.664274
C	-1.118976	-0.889631	-0.763429
C	0.113888	-0.471856	-1.062129
C	-1.885282	0.703197	2.554139
C	-3.128252	-0.800544	0.545300
C	-3.591090	0.692701	0.602884
C	-3.078402	1.400938	1.874404
C	-5.105925	0.571522	0.570322
C	-4.558394	-0.500273	-1.458316
C	-3.499875	-1.404191	-0.819391
H	-2.259272	-0.093257	3.216383
H	0.785895	1.252597	1.836715
H	-3.904871	1.496092	2.591782
H	-5.100229	-0.978752	-2.281571
O	2.102527	0.629344	-0.300974
C	0.605029	-0.188528	-2.446011
H	-0.076245	-0.591039	-3.205933
H	0.675018	0.901051	-2.591486
H	1.615477	-0.581681	-2.618789
C	-2.258507	-1.181452	-1.717029
H	-2.037736	-2.030058	-2.380783
H	-1.344000	1.421102	3.185991
H	-2.772863	2.422712	1.613635
O	-5.891080	0.958670	1.393626
O	-5.546679	-0.058961	-0.533278
C	-3.232027	1.474674	-0.711380
H	-2.139590	1.600032	-0.743339
C	-3.643338	0.632279	-1.944535
H	-4.100948	1.260646	-2.719847
H	-3.796160	-2.456308	-0.747901
H	-3.626979	-1.325925	1.371477
C	-3.884102	2.855907	-0.772316
H	-4.981877	2.787770	-0.844531
H	-3.642339	3.474755	0.102705
H	-3.529848	3.392179	-1.665156
O	-2.575901	-0.062613	-2.544011
O	-1.284100	-2.300201	1.107534
C	-1.506066	-2.617321	2.461001
H	-2.551401	-2.447404	2.771075
H	-1.287616	-3.687986	2.573615
H	-0.839794	-2.050930	3.135831

C	1.644032	-1.544387	0.613852
H	0.810098	-2.173367	0.982257
N	3.105791	-0.986419	2.560344
N	2.435098	-1.264380	1.731246
Si	2.722199	-2.702983	-0.582903
C	1.501608	-3.510708	-1.738813
H	0.665301	-3.953120	-1.174976
H	1.092809	-2.829155	-2.496512
H	2.022108	-4.327813	-2.266443
C	4.066528	-1.686216	-1.372099
H	4.727546	-2.350905	-1.952946
H	3.680661	-0.905155	-2.041291
H	4.675980	-1.192945	-0.598258
C	3.439762	-3.975286	0.586247
H	4.162288	-3.539688	1.295293
H	2.653055	-4.493148	1.158382
H	3.979598	-4.735510	-0.002622
Al	3.184170	1.967889	-0.091446
Cl	2.222444	3.603117	0.952323
Cl	4.892579	1.290152	1.072191
Cl	3.818312	2.593983	-2.055513

65

26a-Al_3 Eopt -3290.929734

C	-1.367744	0.167813	-0.164536
C	-0.692268	0.096480	1.193119
C	0.540673	0.537331	1.443866
C	1.239273	1.398984	0.426552
C	0.775448	1.128090	-0.979094
C	-0.445489	0.679980	-1.279121
C	1.411462	0.081327	2.576258
C	2.747909	1.127540	0.381212
C	3.093057	-0.370444	0.650413
C	2.495639	-0.851318	1.993534
C	4.611648	-0.366147	0.646777
C	4.186613	0.469419	-1.519903
C	3.187007	1.525769	-1.035497
H	1.888232	0.941754	3.069958
H	-1.226747	-0.507479	1.933961
H	3.305679	-0.976857	2.725090
H	4.780756	0.792980	-2.381771
O	-1.768437	-1.099565	-0.539781
C	-0.922469	0.486487	-2.689019
H	-1.708783	1.220106	-2.937096
H	-0.109160	0.620214	-3.411328
H	-1.344038	-0.519392	-2.823517
C	1.948008	1.281828	-1.932597
H	1.796284	2.058925	-2.694779
H	0.829742	-0.459284	3.335951
H	2.038972	-1.839010	1.845647
O	5.351012	-0.695748	1.535179
O	5.119450	0.082814	-0.515989
C	2.696220	-1.287438	-0.562222
H	1.598769	-1.341732	-0.600797
C	3.195897	-0.644682	-1.883124
H	3.617974	-1.403424	-2.554960
H	3.563134	2.553136	-1.088727
H	3.265621	1.719948	1.147336
C	3.243453	-2.706654	-0.420396
H	4.343145	-2.730426	-0.495163
H	2.958439	-3.167983	0.534856
H	2.843626	-3.340763	-1.225453
O	2.208786	0.051324	-2.609745
O	0.983257	2.792661	0.619978
C	1.328213	3.382109	1.847651
H	2.411028	3.334703	2.057937
H	1.042018	4.440666	1.776600

H	0.786419	2.936826	2.699754
C	-2.704007	1.013494	0.001313
H	-3.230084	0.615576	0.890662
N	-4.241028	0.357153	-1.864780
N	-3.566651	0.640656	-1.043378
Si	-2.762568	2.986009	0.218049
C	-2.253716	3.237312	1.989918
H	-2.150768	4.315101	2.197047
H	-3.010217	2.826007	2.676641
H	-1.291085	2.752489	2.198433
C	-1.673761	3.812109	-1.042195
H	-1.880244	3.453679	-2.062919
H	-1.877526	4.896091	-1.018705
H	-0.613812	3.646092	-0.803441
C	-4.566641	3.400651	-0.049515
H	-4.882356	3.252314	-1.094989
H	-5.227125	2.809235	0.604828
H	-4.725282	4.465052	0.191383
Al	-2.088142	-2.650681	0.202144
Cl	-3.017683	-3.849482	-1.329208
Cl	-3.465870	-2.339356	1.858365
Cl	-0.276221	-3.596257	0.912410

65

26a-Al_4 Eopt -3290.929630

C	-1.312316	-0.183672	0.172035
C	-0.644989	-0.081219	-1.187565
C	0.599079	-0.484424	-1.446156
C	1.323688	-1.337806	-0.440275
C	0.858000	-1.095267	0.969862
C	-0.373015	-0.681755	1.278227
C	1.454056	0.009042	-2.574820
C	2.824361	-1.026828	-0.396742
C	3.128510	0.483314	-0.647542
C	2.512095	0.965135	-1.982044
C	4.646781	0.518738	-0.650202
C	4.253512	-0.357101	1.507056
C	3.279189	-1.432155	1.012830
H	1.954080	-0.831270	-3.080017
H	-1.197542	0.514051	-1.921846
H	3.315387	1.125826	-2.714172
H	4.860060	-0.676442	2.361807
O	-1.738298	1.071421	0.560529
C	-0.852920	-0.516131	2.690480
H	-0.036961	-0.642072	3.411236
H	-1.295609	0.478981	2.837014
H	-1.624080	-1.269039	2.928574
C	2.037671	-1.231751	1.917523
H	1.908264	-2.022498	2.669742
H	0.855675	0.542349	-3.326663
H	2.026739	1.936819	-1.819735
O	5.373250	0.879957	-1.536928
O	5.171145	0.067102	0.504119
C	2.712389	1.372499	0.579319
H	1.614212	1.391043	0.625842
C	3.236248	0.726133	1.889263
H	3.641968	1.486769	2.568992
H	3.681459	-2.450232	1.050963
H	3.354300	-1.595263	-1.172408
C	3.215706	2.809583	0.454807
H	2.808069	3.417937	1.275716
H	4.314984	2.865204	0.516221
H	2.906165	3.278760	-0.489177
O	2.270211	-0.004266	2.610447
O	1.105320	-2.735666	-0.648818
C	1.459218	-3.298344	-1.886466
H	1.210731	-4.367014	-1.826118

H	0.894259	-2.861567	-2.727927
H	2.537734	-3.210240	-2.105793
C	-2.624842	-1.067948	0.004819
H	-3.161030	-0.689749	-0.887868
N	-4.182170	-0.443221	1.863798
N	-3.500573	-0.715527	1.044392
Si	-2.624307	-3.041145	-0.208766
C	-4.414582	-3.506495	0.069210
H	-4.544594	-4.576213	-0.165076
H	-4.730667	-3.361133	1.115014
H	-5.094301	-2.937657	-0.585545
C	-2.120895	-3.277039	-1.984526
H	-2.897257	-2.893248	-2.664993
H	-1.177551	-2.759424	-2.202362
H	-1.982070	-4.350799	-2.191512
C	-1.505835	-3.841490	1.042346
H	-1.681705	-4.930287	1.014086
H	-0.451923	-3.647319	0.798537
H	-1.715985	-3.494425	2.066192
Al	-2.284527	2.552050	-0.194953
Cl	-3.711334	1.999764	-1.747921
Cl	-3.268857	3.679546	1.354575
Cl	-0.647699	3.674823	-1.049146

65

26a-Al_5 Eopt -3290.931604

C	1.089685	-0.154941	0.061634
C	0.254560	0.501777	1.156298
C	-1.023141	0.181757	1.380408
C	-1.614433	-0.948683	0.539298
C	-1.080495	-0.840853	-0.878220
C	0.177919	-0.472161	-1.133629
C	-1.879817	0.917568	2.384545
C	-3.149121	-0.904848	0.375186
C	-3.742441	0.498464	0.661896
C	-3.393050	0.883135	2.097293
C	-5.240811	0.322653	0.497766
C	-4.508895	-0.351391	-1.627019
C	-3.454444	-1.317446	-1.078501
H	-1.703695	0.496916	3.387976
H	0.747227	1.300222	1.718642
H	-3.879317	0.140799	2.747643
H	-4.961737	-0.694593	-2.563922
O	2.155726	0.597107	-0.308322
C	0.714562	-0.172839	-2.497200
H	0.770650	0.918111	-2.637192
H	1.737206	-0.549553	-2.630846
H	0.072790	-0.585415	-3.286236
C	-2.199289	-0.946530	-1.885409
H	-1.975262	-1.643942	-2.705229
H	-1.522596	1.956777	2.427113
H	-3.847487	1.848397	2.358594
O	-6.089074	0.529004	1.324027
O	-5.582419	-0.118266	-0.724462
C	-3.312719	1.512552	-0.447821
H	-2.221653	1.644883	-0.394469
C	-3.616126	0.880197	-1.834974
H	-4.051311	1.621410	-2.518572
H	-3.729062	-2.374382	-1.169373
H	-3.610287	-1.603283	1.086418
C	-3.965809	2.882084	-0.288953
H	-3.603722	3.563169	-1.073500
H	-5.063414	2.830171	-0.376599
H	-3.724510	3.336568	0.682527
O	-2.484192	0.331794	-2.471484
O	-1.225553	-2.234149	1.045387
C	-1.440338	-2.454811	2.419722

H	-2.477413	-2.232040	2.724596
H	-1.248496	-3.519898	2.607520
H	-0.755062	-1.856089	3.046230
C	1.639368	-1.551073	0.613290
H	0.785354	-2.170584	0.951680
N	3.027206	-0.989122	2.610905
N	2.387003	-1.263863	1.756864
Si	2.756410	-2.722828	-0.531587
C	4.146074	-1.722262	-1.261011
H	3.799161	-0.956368	-1.967967
H	4.708683	-1.212167	-0.462827
H	4.840290	-2.397800	-1.788116
C	3.408075	-3.999458	0.670867
H	3.966937	-4.766579	0.109361
H	4.102219	-3.566721	1.409301
H	2.592472	-4.507959	1.209803
C	1.579553	-3.513470	-1.742926
H	1.217446	-2.824121	-2.517416
H	2.111477	-4.337770	-2.247369
H	0.710628	-3.942883	-1.219918
Al	3.218616	1.946569	-0.074548
Cl	2.193360	3.608599	0.861990
Cl	3.968907	2.518958	-2.014104
Cl	4.858450	1.310640	1.205801

65

26a-Al_6 Eopt -3290.935276

C	1.292384	-0.243608	0.244107
C	0.446225	0.193864	1.436884
C	-0.797696	-0.249930	1.616174
C	-1.360965	-1.230583	0.609808
C	-0.822385	-0.981637	-0.777431
C	0.406054	-0.501713	-0.984157
C	-1.767352	0.213614	2.659834
C	-2.882169	-1.076363	0.445761
C	-3.372682	0.397862	0.636292
C	-2.926895	0.978548	1.995621
C	-4.882263	0.264735	0.523981
C	-4.232491	-0.587384	-1.576509
C	-3.185550	-1.538983	-0.988989
H	-2.165672	-0.658963	3.201022
H	0.916473	0.895237	2.133946
H	-3.788168	1.007387	2.676850
H	-4.731193	-0.985031	-2.467324
O	2.319789	0.620478	-0.008769
C	0.929448	-0.083129	-2.323140
H	0.360301	-0.545697	-3.139856
H	0.826114	1.008903	-2.425731
H	1.994927	-0.313896	-2.448800
C	-1.911880	-1.205160	-1.804568
H	-1.641427	-1.980726	-2.536071
H	-1.271731	0.857050	3.400083
H	-2.606135	2.018398	1.849831
O	-5.708296	0.558015	1.346411
O	-5.266026	-0.256623	-0.655101
C	-2.963338	1.311756	-0.574307
H	-1.871719	1.443789	-0.545688
C	-3.317432	0.600541	-1.904249
H	-3.754703	1.302603	-2.626143
H	-3.465326	-2.596795	-1.040701
H	-3.404041	-1.691524	1.191908
C	-3.613825	2.693388	-0.521395
H	-4.707803	2.638578	-0.645784
H	-3.407370	3.219349	0.420733
H	-3.219599	3.315688	-1.338490
O	-2.219963	-0.018191	-2.532882
O	-1.010881	-2.587105	0.915342

C	-1.288631	-3.066585	2.208449
H	-1.025046	-4.133150	2.212469
H	-0.687146	-2.555170	2.981089
H	-2.355982	-2.978582	2.476014
C	1.962510	-1.640375	0.639045
H	1.193014	-2.340503	1.014295
N	3.502552	-1.024717	2.505529
N	2.798135	-1.345311	1.721911
Si	3.067303	-2.666849	-0.651376
C	4.399353	-1.538641	-1.299876
H	3.998365	-0.629202	-1.767472
H	5.062890	-1.224112	-0.478070
H	5.013986	-2.078135	-2.039359
C	3.790391	-4.030576	0.405764
H	4.402659	-4.690756	-0.231087
H	4.446855	-3.646276	1.203380
H	3.002287	-4.647632	0.866437
C	1.883751	-3.381416	-1.901198
H	1.514187	-2.648243	-2.629843
H	2.416032	-4.171652	-2.457217
H	1.020895	-3.845602	-1.398524
Al	2.464504	2.367050	-0.017108
Cl	0.559803	3.263763	-0.543184
Cl	3.059718	3.017812	1.962604
Cl	3.981898	2.864729	-1.465166

65

26a-Al_7 Eopt -3290.933746

C	1.076182	0.153511	-0.152329
C	0.272726	-0.497615	-1.274574
C	-0.975124	-0.117867	-1.546619
C	-1.603218	0.976135	-0.708090
C	-1.098733	0.931857	0.718731
C	0.144805	0.534777	1.009376
C	-1.866597	-0.644240	-2.624868
C	-3.133249	0.808109	-0.571903
C	-3.592407	-0.680677	-0.701437
C	-3.136651	-1.291102	-2.040234
C	-5.107665	-0.598095	-0.604916
C	-4.511781	0.368755	1.455123
C	-3.501819	1.333993	0.829750
H	-2.149071	0.212193	-3.259970
H	0.785137	-1.263960	-1.863329
H	-3.957253	-1.208349	-2.765362
H	-5.029662	0.789966	2.323919
O	2.121499	-0.598295	0.269688
C	0.649200	0.282547	2.394772
H	0.710401	-0.802420	2.572724
H	1.665573	0.671831	2.540820
H	-0.015818	0.715723	3.152309
C	-2.238031	1.122288	1.696134
H	-2.053012	1.937867	2.411146
H	-1.333987	-1.361187	-3.264533
H	-2.956088	-2.364961	-1.902222
O	-5.912305	-0.961065	-1.420364
O	-5.524334	-0.045100	0.547210
C	-3.158724	-1.523574	0.552776
H	-2.062427	-1.613485	0.539700
C	-3.550693	-0.760569	1.844941
H	-3.963864	-1.444993	2.597429
H	-3.845269	2.374102	0.816865
H	-3.629140	1.376704	-1.372145
C	-3.760376	-2.928132	0.565763
H	-4.858186	-2.903382	0.662048
H	-3.513527	-3.500470	-0.338644
H	-3.368225	-3.488432	1.427445
O	-2.486959	-0.064817	2.451772

O	-1.251523	2.185817	-1.389289
C	-1.708430	3.395980	-0.835880
H	-1.199902	4.206704	-1.375988
H	-2.796372	3.529131	-0.965276
H	-1.463393	3.484884	0.237349
C	1.671005	1.518686	-0.751557
H	0.850832	2.113545	-1.199610
N	3.181677	0.791904	-2.604493
N	2.489318	1.142347	-1.822222
Si	2.750806	2.733392	0.382632
C	3.463226	3.951860	-0.844725
H	4.168999	3.481882	-1.548500
H	2.672252	4.454229	-1.424614
H	4.019914	4.728965	-0.294847
C	1.544877	3.593662	1.514966
H	2.088313	4.395323	2.043231
H	0.736377	4.064382	0.934029
H	1.099368	2.934953	2.272143
C	4.098481	1.748118	1.206873
H	4.681064	1.190703	0.456081
H	4.784851	2.438973	1.724250
H	3.716477	1.023185	1.938457
Al	3.132099	-2.002416	0.160962
Cl	4.875141	-1.473442	-1.028205
Cl	3.731943	-2.534729	2.163682
Cl	2.090389	-3.647842	-0.782644

65

26a-Al_8 Eopt -3290.931290

C	-1.031901	0.147212	-0.081719
C	-0.280375	0.698663	1.117495
C	0.989538	1.103065	1.078074
C	1.684659	1.172656	-0.256426
C	1.173575	0.131177	-1.219349
C	-0.077934	-0.334205	-1.187809
C	1.874971	1.366043	2.259078
C	3.186728	0.879206	-0.150361
C	3.526074	-0.161676	0.962968
C	2.966739	0.278096	2.334797
C	5.044252	-0.201193	0.928849
C	4.559998	-0.835032	-1.293314
C	3.587129	0.324911	-1.527071
H	2.349931	2.353862	2.154677
H	-0.825006	0.628633	2.063195
H	3.794602	0.632128	2.964411
H	5.130303	-1.113376	-2.186472
O	-1.835581	-0.858352	0.366187
C	-0.611765	-1.348078	-2.151341
H	-0.634889	-2.348055	-1.694590
H	-1.645952	-1.112804	-2.449899
H	0.004216	-1.404545	-3.058014
C	2.319355	-0.379543	-2.070892
H	2.156748	-0.226748	-3.147604
H	1.296734	1.378066	3.193656
H	2.539073	-0.597398	2.841781
O	5.806443	0.052296	1.822782
O	5.519869	-0.561188	-0.277287
C	3.087030	-1.615874	0.561026
H	1.988254	-1.655431	0.578163
C	3.547996	-1.915725	-0.888779
H	3.941458	-2.937049	-0.974906
H	3.970662	1.100494	-2.198672
H	3.732064	1.802546	0.089498
C	3.627850	-2.676177	1.519714
H	4.725570	-2.757936	1.462556
H	3.360013	-2.470966	2.565442
H	3.211659	-3.660070	1.257099

O	2.537258	-1.774470	-1.859630
O	1.471666	2.421630	-0.922905
C	1.735764	3.605024	-0.211838
H	1.556964	4.437202	-0.906951
H	1.067846	3.727663	0.658074
H	2.781798	3.671419	0.135719
C	-2.024233	1.249444	-0.719541
H	-2.770297	0.701504	-1.327183
N	-0.832418	2.769421	-2.293227
N	-1.374080	2.079646	-1.625738
Si	-3.072037	2.447136	0.472633
C	-4.305662	3.221258	-0.695336
H	-4.958142	2.457351	-1.146731
H	-4.945344	3.928464	-0.141903
H	-3.811959	3.783086	-1.504831
C	-3.882888	1.399692	1.772098
H	-4.614116	0.709145	1.327962
H	-3.166307	0.815605	2.365223
H	-4.426393	2.074360	2.455202
C	-1.894127	3.722745	1.158747
H	-2.484937	4.509772	1.656956
H	-1.205108	3.294571	1.901038
H	-1.301337	4.209078	0.367303
Al	-3.177885	-1.950924	0.219859
Cl	-2.581419	-3.902337	-0.493709
Cl	-4.097711	-2.207149	2.155507
Cl	-4.607075	-1.067065	-1.180037

65

26a-Al_9 Eopt -3290.934993

C	1.323047	-0.392043	-0.471880
C	0.759810	-0.501745	0.929242
C	-0.460793	-0.956172	1.205347
C	-1.307669	-1.538858	0.104969
C	-0.985152	-0.929891	-1.241784
C	0.222772	-0.438010	-1.546034
C	-1.160053	-0.887191	2.524134
C	-2.806778	-1.235125	0.304754
C	-3.043786	0.111990	1.058869
C	-2.321994	0.121193	2.424172
C	-4.555053	0.169940	1.207327
C	-4.375389	0.045930	-1.137969
C	-3.418569	-1.149038	-1.106140
H	-1.548805	-1.889035	2.768320
H	1.395611	-0.091602	1.717850
H	-3.053846	-0.076389	3.219077
H	-5.059073	0.029289	-1.994064
O	2.082659	0.739631	-0.606607
C	0.548350	0.213266	-2.855902
H	1.554645	-0.068762	-3.203235
H	-0.178564	-0.058635	-3.631953
H	0.536435	1.307743	-2.746596
C	-2.261439	-0.698875	-2.031714
H	-2.263741	-1.194504	-3.013823
H	-0.473820	-0.590849	3.329393
H	-1.919303	1.125976	2.609794
O	-5.189550	0.236119	2.226024
O	-5.191619	0.135220	0.023275
C	-2.684538	1.345198	0.152250
H	-1.595153	1.363256	0.006746
C	-3.341509	1.172209	-1.242528
H	-3.760625	2.121494	-1.600509
H	-3.888012	-2.085423	-1.425371
H	-3.262222	-2.035279	0.906100
C	-3.103913	2.675979	0.773741
H	-2.674771	2.821932	1.774569
H	-2.751080	3.503678	0.141752

H	-4.199634	2.764446	0.857721
O	-2.476694	0.695428	-2.248964
O	-1.030730	-2.940933	0.161666
C	-1.765285	-3.792460	-0.681814
H	-1.341581	-4.799536	-0.565785
H	-2.832365	-3.844649	-0.406735
H	-1.685650	-3.505004	-1.746493
C	2.396495	-1.544756	-0.747505
H	2.908077	-1.302027	-1.695248
N	1.280283	-3.749067	-1.075111
N	1.783460	-2.774658	-0.973546
Si	3.839181	-1.856722	0.594581
C	4.601166	-0.210867	0.992363
H	3.928015	0.426598	1.583076
H	5.511473	-0.394245	1.588265
H	4.889964	0.335510	0.082645
C	3.078003	-2.721415	2.063795
H	2.435775	-3.566604	1.766946
H	3.891032	-3.128473	2.688254
H	2.483029	-2.038119	2.686780
C	5.008315	-2.989521	-0.322143
H	5.858738	-3.244236	0.331790
H	4.523998	-3.934448	-0.617978
H	5.411663	-2.505969	-1.226031
Al	2.080261	2.412915	-0.100784
Cl	0.319721	3.445153	-0.831565
Cl	2.131047	2.609613	2.062745
Cl	3.860579	3.280765	-0.962655

65

26a-B_1 Eopt -1992.392548

C	1.559803	0.557085	0.211473
C	0.618789	0.916225	1.357749
C	-0.479938	0.205876	1.601707
C	-0.805870	-0.967298	0.703563
C	-0.330320	-0.731386	-0.712265
C	0.767582	-0.014665	-0.975324
C	-1.479516	0.435432	2.689097
C	-2.329123	-1.183656	0.557083
C	-3.140469	0.141777	0.734472
C	-2.857840	0.791671	2.102583
C	-4.589410	-0.302302	0.612955
C	-3.766820	-1.015560	-1.470709
C	-2.552287	-1.726738	-0.868616
H	-1.555806	-0.497601	3.272385
H	0.919925	1.757290	1.987181
H	-3.646903	0.494592	2.806403
H	-4.161923	-1.517232	-2.361082
O	2.410816	1.577437	-0.141045
C	1.193008	0.384517	-2.353320
H	0.739627	-0.259905	-3.117531
H	0.868048	1.418410	-2.541664
H	2.284294	0.371005	-2.469933
C	-1.376883	-1.174055	-1.710838
H	-0.987368	-1.893130	-2.447286
H	-1.143663	1.223739	3.376654
H	-2.927915	1.882506	2.003057
O	-5.463298	-0.174201	1.428257
O	-4.853530	-0.898104	-0.562274
C	-2.914941	1.112630	-0.482631
H	-1.872419	1.461870	-0.452797
C	-3.111381	0.328845	-1.807393
H	-3.680146	0.921902	-2.535489
H	-2.629496	-2.819110	-0.899855
H	-2.672870	-1.887497	1.329304
C	-3.833741	2.332931	-0.450774
H	-4.894710	2.052432	-0.555867

H	-3.728046	2.914492	0.475072
H	-3.585286	3.001232	-1.288504
O	-1.913273	-0.065151	-2.434278
O	-0.149260	-2.081175	1.323754
C	-0.319426	-3.344963	0.731800
H	0.386204	-4.027966	1.225041
H	-1.338508	-3.742123	0.879584
H	-0.097407	-3.336435	-0.350104
C	2.502764	-0.599351	0.761451
H	1.884739	-1.392009	1.226642
B	1.946584	2.955782	-0.293661
F	2.046401	3.647546	0.931483
F	0.600938	2.992051	-0.719626
F	2.764879	3.575253	-1.242271
N	3.835850	0.495602	2.572149
N	3.232927	-0.019483	1.808705
Si	3.823549	-1.504951	-0.405340
C	4.813143	-2.543221	0.795638
H	5.399133	-1.930156	1.499209
H	4.168162	-3.223378	1.374940
H	5.528552	-3.162475	0.229076
C	2.833040	-2.602185	-1.539588
H	3.529221	-3.304902	-2.027321
H	2.106753	-3.195162	-0.962369
H	2.297714	-2.055566	-2.326945
C	4.888287	-0.212115	-1.217215
H	4.304906	0.508548	-1.806057
H	5.445544	0.354798	-0.454178
H	5.623297	-0.701672	-1.877449

65

26a-B_10 Eopt -1992.387046

C	1.448025	0.704341	-0.444291
C	0.873025	0.516570	0.947934
C	-0.214666	-0.213968	1.184674
C	-0.871000	-0.943106	0.041252
C	-0.718374	-0.189140	-1.260417
C	0.357997	0.560485	-1.519975
C	-0.905669	-0.399386	2.497480
C	-2.396449	-1.079452	0.223888
C	-3.005882	0.085489	1.068847
C	-2.331789	0.182601	2.452322
C	-4.476002	-0.287578	1.173967
C	-4.248385	-0.166762	-1.166073
C	-3.000830	-1.052827	-1.193965
H	-0.954681	-1.482302	2.701075
H	1.384765	1.055479	1.745019
H	-2.962161	-0.328663	3.192308
H	-4.892231	-0.300227	-2.042526
O	2.015035	1.941813	-0.606872
C	0.558905	1.335188	-2.785134
H	1.566405	1.160751	-3.194927
H	-0.184073	1.064461	-3.545772
H	0.484044	2.414456	-2.589676
C	-2.008830	-0.231050	-2.052364
H	-1.881022	-0.624073	-3.071778
H	-0.335170	0.060733	3.315956
H	-2.289800	1.236296	2.757724
O	-5.111600	-0.483792	2.174835
O	-5.068949	-0.396653	-0.028017
C	-2.995300	1.440247	0.270917
H	-1.949507	1.763632	0.164409
C	-3.564238	1.205287	-1.153076
H	-4.227452	2.027160	-1.452709
H	-3.189245	-2.054780	-1.594585
H	-2.618271	-2.017971	0.752352
C	-3.778081	2.548570	0.974246

H	-4.855200	2.319997	1.025907
H	-3.422923	2.729755	1.997830
H	-3.669068	3.489612	0.415019
O	-2.585993	1.071654	-2.158461
O	-0.204855	-2.208512	0.005830
C	-0.679265	-3.185462	-0.885080
H	-1.674253	-3.569071	-0.601378
H	-0.726580	-2.822821	-1.928573
H	0.029675	-4.024384	-0.843119
C	2.619337	-0.405619	-0.719613
H	3.411580	0.124345	-1.274360
B	3.150094	2.509769	0.103565
F	3.433251	3.738173	-0.487836
F	4.288316	1.661064	-0.015081
F	2.894951	2.694666	1.477641
N	1.886101	-2.195494	-2.284289
N	2.200685	-1.390922	-1.598569
Si	3.610830	-1.361738	0.743928
C	3.595471	-0.428178	2.352958
H	3.727817	0.653022	2.220421
H	2.675340	-0.615827	2.924510
H	4.446046	-0.811556	2.942630
C	2.800888	-3.034775	0.931118
H	2.928212	-3.674496	0.043022
H	3.277514	-3.554165	1.779365
H	1.725498	-2.932765	1.142365
C	5.325711	-1.508116	0.020536
H	5.963265	-2.105802	0.692710
H	5.312971	-2.007423	-0.961804
H	5.783565	-0.514332	-0.096182

65

26a-B_11 Eopt -1992.385445

C	-1.447022	0.672836	-0.117245
C	-0.766873	-0.101936	-1.232359
C	0.347572	-0.808574	-1.062872
C	0.938736	-0.914279	0.318522
C	0.674819	0.334748	1.132552
C	-0.425415	1.078977	0.964396
C	1.129288	-1.527136	-2.115039
C	2.477359	-1.029336	0.290555
C	3.109393	-0.333962	-0.957985
C	2.531698	-0.907150	-2.267789
C	4.593808	-0.628925	-0.813838
C	4.203045	0.510911	1.207858
C	2.982311	-0.337662	1.571758
H	1.222703	-2.580912	-1.803605
H	-1.234994	0.001543	-2.212350
H	3.226814	-1.659725	-2.663778
H	4.789187	0.820374	2.080356
O	-2.017241	1.789316	-0.686259
C	-0.700439	2.348232	1.706560
H	-1.724711	2.355049	2.104883
H	0.011780	2.501624	2.526427
H	-0.631147	3.206440	1.021541
C	1.910657	0.723419	1.919455
H	1.724777	0.822289	2.999056
H	0.602710	-1.517363	-3.079362
H	2.482324	-0.107112	-3.018080
O	5.302108	-1.212112	-1.590086
O	5.105943	-0.157319	0.336111
C	3.002564	1.231247	-0.856497
H	1.941908	1.505834	-0.952714
C	3.482214	1.690760	0.545713
H	4.100051	2.595390	0.473606
H	3.172515	-1.042071	2.388719
H	2.762095	-2.090692	0.252162

C	3.793263	1.952387	-1.947195
H	3.496795	1.639256	-2.957614
H	3.620390	3.036292	-1.873103
H	4.878033	1.784834	-1.845951
O	2.443406	1.966434	1.456371
O	0.310689	-2.073209	0.873700
C	0.763978	-2.538715	2.119471
H	1.788571	-2.945169	2.070836
H	0.731229	-1.758436	2.902343
H	0.092380	-3.356064	2.417394
C	-2.617318	-0.222160	0.545389
H	-3.402670	0.473404	0.895299
B	-3.338848	2.356093	-0.497843
F	-3.357041	3.605595	-1.103664
F	-3.679291	2.480244	0.878476
F	-4.333862	1.523827	-1.075309
N	-1.946761	-1.324973	2.674392
N	-2.214958	-0.824143	1.730307
Si	-3.570979	-1.640163	-0.498404
C	-3.587482	-1.255812	-2.320214
H	-3.686384	-0.177851	-2.504627
H	-2.697326	-1.644389	-2.834716
H	-4.473536	-1.757164	-2.745673
C	-2.679251	-3.238537	-0.122192
H	-2.829711	-3.570556	0.917810
H	-3.077157	-4.028384	-0.780836
H	-1.596995	-3.146016	-0.302689
C	-5.279902	-1.593321	0.249162
H	-5.252321	-1.746220	1.340120
H	-5.763274	-0.625898	0.042746
H	-5.903566	-2.390643	-0.187444

65

26a-B_12 Eopt -1992.385390

C	1.433713	0.845800	-0.245215
C	0.824721	0.457271	1.087973
C	-0.258586	-0.309001	1.181632
C	-0.862877	-0.862235	-0.082417
C	-0.708446	0.102329	-1.237093
C	0.361244	0.896877	-1.345014
C	-0.962123	-0.731041	2.430451
C	-2.386637	-1.076302	0.032421
C	-3.053422	-0.095467	1.050750
C	-2.418357	-0.227661	2.448826
C	-4.513790	-0.518508	1.049220
C	-4.238499	0.013845	-1.226103
C	-2.963428	-0.819301	-1.374637
H	-0.953608	-1.834185	2.455639
H	1.330719	0.853788	1.968377
H	-3.035242	-0.904230	3.055404
H	-4.858064	0.018532	-2.129767
O	2.020912	2.084954	-0.174762
C	0.578202	1.883862	-2.448780
H	1.607250	1.818668	-2.836105
H	-0.126322	1.725928	-3.275435
H	0.452602	2.909988	-2.073494
C	-1.981632	0.167174	-2.052382
H	-1.821542	-0.036079	-3.121620
H	-0.426844	-0.382567	3.324423
H	-2.448541	0.748237	2.950425
O	-5.165330	-0.899750	1.984299
O	-5.076776	-0.433311	-0.168615
C	-3.065408	1.377944	0.501613
H	-2.027002	1.739724	0.477482
C	-3.598742	1.380435	-0.955429
H	-4.281948	2.223367	-1.123151
H	-3.112311	-1.741010	-1.947428

H	-2.588000	-2.098835	0.383662
C	-3.891298	2.329764	1.366259
H	-3.555163	2.348061	2.411899
H	-3.803751	3.353724	0.973704
H	-4.961543	2.065981	1.359510
O	-2.596371	1.452653	-1.942978
O	-0.144141	-2.081972	-0.310430
C	-0.582053	-2.910254	-1.358175
H	-1.561672	-3.372528	-1.149033
H	-0.645983	-2.376562	-2.324488
H	0.155904	-3.718480	-1.458972
C	2.584788	-0.239707	-0.672451
H	3.424522	0.365100	-1.067146
B	3.326117	2.355275	0.397168
F	3.384388	3.691797	0.768845
F	4.341716	2.085737	-0.564291
F	3.583586	1.522166	1.513459
N	1.892979	-1.594740	-2.639761
N	2.189850	-0.984328	-1.770619
Si	3.498963	-1.526699	0.582677
C	3.170430	-3.230717	-0.119898
H	3.664761	-3.964509	0.538524
H	2.092926	-3.458507	-0.120056
H	3.574145	-3.380895	-1.133958
C	5.295796	-1.058434	0.408075
H	5.459901	-0.022813	0.738129
H	5.914171	-1.727739	1.028572
H	5.636284	-1.151737	-0.635598
C	2.921415	-1.471407	2.350018
H	3.044382	-0.476786	2.796590
H	1.880875	-1.804347	2.465262
H	3.569630	-2.177907	2.897897

65

26a-B_13 Eopt -1992.386978

C	-1.504636	0.636007	0.100127
C	-0.800939	0.580431	-1.241114
C	0.295621	-0.134627	-1.477879
C	0.783766	-1.133389	-0.466629
C	0.370432	-0.777504	0.938219
C	-0.740358	-0.086736	1.220258
C	1.205967	0.005482	-2.655194
C	2.321409	-1.203229	-0.411094
C	2.979260	0.164619	-0.774387
C	2.528661	0.639127	-2.174476
C	4.468269	-0.131310	-0.719246
C	3.852757	-0.706173	1.480784
C	2.679787	-1.585009	1.036943
H	1.397050	-0.991896	-3.082850
H	-1.183977	1.271596	-1.994078
H	3.326869	0.426251	-2.898538
H	4.354427	-1.076783	2.381647
O	-1.620818	1.946575	0.544352
C	-1.199063	0.174295	2.625483
H	-0.441207	-0.123413	3.359444
H	-1.431025	1.236725	2.776225
H	-2.110472	-0.410108	2.842174
C	1.505455	-1.070532	1.905349
H	1.234890	-1.751188	2.725307
H	0.757576	0.626902	-3.442397
H	2.399228	1.729573	-2.159323
O	5.273006	-0.012128	-1.603983
O	4.861277	-0.579000	0.486582
C	2.739172	1.226445	0.362006
H	1.670500	1.485448	0.365706
C	3.086220	0.595606	1.738637
H	3.633561	1.308386	2.369218

H	2.870606	-2.656448	1.160053
H	2.678948	-1.948798	-1.135860
C	3.544271	2.507580	0.149885
H	4.629215	2.328981	0.229520
H	3.347942	2.965895	-0.829117
H	3.274396	3.245532	0.919749
O	1.976870	0.148676	2.485946
O	0.211246	-2.368115	-0.898615
C	0.540266	-3.543037	-0.205611
H	-0.126282	-4.330314	-0.585252
H	1.582174	-3.861867	-0.384059
H	0.378202	-3.455456	0.883796
C	-2.986876	0.092865	-0.131093
H	-3.377737	0.626578	-1.017375
B	-1.907225	3.100398	-0.305354
F	-2.395148	4.108746	0.527434
F	-2.897709	2.773416	-1.274085
F	-0.759132	3.548931	-0.974399
N	-4.405572	1.014598	1.712505
N	-3.787434	0.611598	0.896527
Si	-3.458816	-1.807280	-0.419199
C	-5.330271	-1.789736	-0.426217
H	-5.695752	-2.803199	-0.662226
H	-5.754391	-1.509222	0.551842
H	-5.730151	-1.104510	-1.190807
C	-2.773958	-2.240492	-2.091373
H	-3.165490	-3.229404	-2.383782
H	-3.091031	-1.511063	-2.853177
H	-1.676379	-2.289731	-2.063364
C	-2.816007	-2.822189	1.005003
H	-3.332622	-2.559611	1.942013
H	-3.013008	-3.888560	0.804489
H	-1.735432	-2.692612	1.148265

65

26a-B_14 Eopt -1992.385747

C	1.446982	0.494252	0.104651
C	0.778397	-0.382994	1.151433
C	-0.372189	-1.029677	0.966273
C	-0.998894	-1.006687	-0.401606
C	-0.689870	0.270155	-1.141838
C	0.434306	0.970576	-0.957186
C	-1.213258	-1.666964	2.031695
C	-2.530960	-1.048395	-0.343450
C	-3.116230	-0.310052	0.901490
C	-2.514013	-0.857211	2.215227
C	-4.605666	-0.580412	0.775250
C	-4.204843	0.518646	-1.275932
C	-2.999801	-0.357053	-1.633108
H	-1.463362	-2.698904	1.740361
H	1.262574	-0.358690	2.130638
H	-3.264208	-1.478001	2.724143
H	-4.795873	0.824749	-2.146254
O	2.036194	1.514304	0.812288
C	0.751102	2.219298	-1.719656
H	0.716449	3.095464	-1.058454
H	1.770740	2.182696	-2.130882
H	0.043797	2.377086	-2.542620
C	-1.895839	0.676099	-1.969108
H	-1.675806	0.749866	-3.043772
H	-0.671011	-1.719485	2.986263
H	-2.296833	-0.014414	2.885233
O	-5.321138	-1.139430	1.562723
O	-5.112469	-0.120531	-0.383359
C	-2.988648	1.250661	0.773871
H	-1.925166	1.512786	0.870636
C	-3.459672	1.696940	-0.634282

H	-4.059625	2.614648	-0.577474
H	-3.188655	-1.065610	-2.446659
H	-2.873768	-2.091213	-0.293470
C	-3.774202	1.991787	1.855370
H	-4.861439	1.852287	1.739498
H	-3.499868	1.667318	2.868759
H	-3.572779	3.071072	1.786010
O	-2.413958	1.937744	-1.546251
O	-0.513608	-2.052239	-1.250654
C	-0.537134	-3.368192	-0.758446
H	-0.178359	-4.015926	-1.570468
H	0.127358	-3.500958	0.112627
H	-1.552318	-3.702402	-0.480753
C	2.639572	-0.296780	-0.635571
H	3.279453	0.478787	-1.096456
B	3.004039	2.560085	0.497192
F	3.734593	2.284464	-0.695429
F	3.918076	2.636146	1.559080
F	2.373948	3.803435	0.361128
N	1.829534	-1.710464	-2.515155
N	2.195103	-1.063525	-1.700971
Si	3.873940	-1.437608	0.421973
C	5.348042	-1.601356	-0.712301
H	5.813185	-0.620162	-0.898311
H	6.105999	-2.253707	-0.248263
H	5.073523	-2.046797	-1.682530
C	4.287181	-0.552583	2.001040
H	4.587320	0.489107	1.822250
H	3.447547	-0.550953	2.710363
H	5.129581	-1.087422	2.471674
C	3.025370	-3.080286	0.683884
H	3.733099	-3.768586	1.175802
H	2.140761	-2.984796	1.331042
H	2.721749	-3.547635	-0.266838

65

26a-B_15 Eopt -1992.386049

C	-1.468836	0.642810	0.018568
C	-0.759798	0.460754	-1.312136
C	0.365678	-0.231986	-1.469855
C	0.887356	-1.096533	-0.355401
C	0.473132	-0.584104	1.001169
C	-0.650561	0.112419	1.210723
C	1.271263	-0.192560	-2.659354
C	2.427298	-1.118260	-0.303484
C	3.053480	0.212608	-0.824178
C	2.585345	0.514425	-2.265297
C	4.548950	-0.046521	-0.748907
C	3.959338	-0.379421	1.507428
C	2.801308	-1.324791	1.175784
H	1.482114	-1.225605	-2.980529
H	-1.169404	1.064421	-2.123774
H	3.382718	0.230771	-2.965659
H	4.473635	-0.635792	2.440305
O	-1.605248	2.004181	0.252276
C	-1.073136	0.559487	2.583676
H	-1.734672	-0.192366	3.048672
H	-0.203309	0.685098	3.240006
H	-1.604916	1.518684	2.543476
C	1.620522	-0.737378	1.987217
H	1.364996	-1.324517	2.881117
H	0.803654	0.329477	-3.505482
H	2.441222	1.597418	-2.377067
O	5.344917	-0.013333	-1.648791
O	4.958711	-0.347250	0.496336
C	2.799974	1.391266	0.186431
H	1.725571	1.624569	0.174137

C	3.169325	0.928320	1.622000
H	3.705380	1.718494	2.163701
H	3.014605	-2.370739	1.419877
H	2.800603	-1.932017	-0.941873
C	3.575316	2.656922	-0.175996
H	4.664230	2.511599	-0.084778
H	3.364973	2.997130	-1.199286
H	3.294071	3.471576	0.507684
O	2.072838	0.547647	2.423346
O	0.351176	-2.389959	-0.634385
C	0.746951	-3.471926	0.166943
H	0.110241	-4.322334	-0.114394
H	1.798100	-3.763078	-0.003922
H	0.603346	-3.278154	1.245265
C	-2.908537	-0.016546	-0.109809
H	-3.418515	0.490158	-0.950146
B	-2.558340	2.898361	-0.396203
F	-2.024393	4.182091	-0.396419
F	-3.780719	2.888821	0.319066
F	-2.854252	2.495086	-1.723643
N	-4.290624	0.537206	1.901734
N	-3.685343	0.333232	1.005766
Si	-3.245063	-1.952732	-0.411568
C	-5.113043	-2.029562	-0.498803
H	-5.502114	-1.405425	-1.319239
H	-5.415397	-3.070985	-0.700393
H	-5.606765	-1.723013	0.437096
C	-2.501631	-2.400655	-2.053672
H	-2.791426	-1.676188	-2.830815
H	-1.407257	-2.459925	-1.992845
H	-2.898289	-3.387554	-2.346865
C	-2.610501	-2.858278	1.087838
H	-3.207870	-2.600507	1.977456
H	-2.697029	-3.946238	0.933327
H	-1.558181	-2.618782	1.288832

65

26a-B_16 Eopt -1992.385951

C	-1.502066	0.632233	0.056255
C	-0.806236	0.497696	-1.286428
C	0.309562	-0.205055	-1.483141
C	0.804975	-1.133615	-0.408746
C	0.389520	-0.697337	0.969569
C	-0.715665	0.009171	1.216487
C	1.271988	-0.000879	-2.615430
C	2.336133	-1.174990	-0.342132
C	2.984998	0.204530	-0.675161
C	2.513575	0.725640	-2.053689
C	4.470281	-0.109531	-0.636796
C	3.855680	-0.730736	1.556984
C	2.665046	-1.581614	1.101509
H	1.572269	-0.966355	-3.050147
H	-1.183513	1.163814	-2.064933
H	3.342225	0.649516	-2.771198
H	4.360709	-1.125253	2.445681
O	-1.636738	1.973244	0.389933
C	-1.170094	0.356015	2.604500
H	-0.411243	0.096731	3.351691
H	-1.388545	1.429286	2.688745
H	-2.089163	-0.199477	2.862289
C	1.491581	-1.041514	1.957163
H	1.172224	-1.730268	2.751795
H	0.824331	0.601186	-3.418397
H	2.264032	1.791942	-1.965467
O	5.273398	0.019812	-1.521581
O	4.859931	-0.595273	0.556397
C	2.770974	1.244141	0.483743

H	1.706656	1.519415	0.498197
C	3.112686	0.580613	1.844813
H	3.674774	1.269503	2.488890
H	2.819688	-2.660305	1.212354
H	2.729758	-1.899723	-1.067083
C	3.597879	2.513831	0.286101
H	3.339452	3.249848	1.061783
H	4.679344	2.315925	0.366398
H	3.412057	2.982392	-0.690402
O	1.994114	0.141514	2.581405
O	0.273591	-2.455710	-0.535343
C	0.507030	-3.169775	-1.722033
H	0.012867	-4.143881	-1.602143
H	0.076444	-2.673236	-2.608546
H	1.579550	-3.352606	-1.909560
C	-2.973378	0.027633	-0.085983
H	-3.395275	0.431167	-1.024698
B	-2.040605	3.039420	-0.525847
F	-2.574122	4.067326	0.253723
F	-3.037227	2.573034	-1.427875
F	-0.955577	3.520894	-1.272286
N	-4.384397	1.154083	1.645917
N	-3.771389	0.656337	0.880030
Si	-3.398944	-1.907630	-0.137485
C	-5.246810	-1.953177	0.155219
H	-5.606516	-2.983482	-0.004589
H	-5.516623	-1.669964	1.185751
H	-5.792278	-1.298183	-0.543149
C	-2.969599	-2.385515	-1.884687
H	-3.667260	-1.918637	-2.597685
H	-1.948396	-2.073712	-2.140701
H	-3.037459	-3.479305	-2.001805
C	-2.486387	-2.847315	1.182837
H	-2.899669	-3.869601	1.221461
H	-1.415214	-2.907876	0.945707
H	-2.618541	-2.394763	2.177889

65

26a-B_17 Eopt -1992.385026

C	-1.472498	0.651180	-0.027535
C	-0.774359	0.344962	-1.345850
C	0.362865	-0.344540	-1.450370
C	0.887552	-1.099490	-0.259067
C	0.481352	-0.463584	1.042882
C	-0.632439	0.256322	1.199084
C	1.312253	-0.284967	-2.611421
C	2.419450	-1.110908	-0.206537
C	3.053634	0.207197	-0.748368
C	2.560874	0.515672	-2.181696
C	4.541023	-0.095036	-0.685322
C	3.959489	-0.384507	1.585202
C	2.768255	-1.297994	1.276828
H	1.613059	-1.299898	-2.914158
H	-1.179166	0.895607	-2.197013
H	3.378944	0.331046	-2.891630
H	4.478205	-0.640529	2.515673
O	-1.624967	2.028267	0.054235
C	-1.035619	0.832640	2.529209
H	-1.690916	0.136581	3.080467
H	-0.152545	1.017636	3.152956
H	-1.564400	1.786332	2.402291
C	1.600931	-0.641629	2.056058
H	1.294831	-1.203382	2.949780
H	0.847362	0.196139	-3.483359
H	2.316160	1.584349	-2.251573
O	5.330419	-0.099794	-1.591594
O	4.949440	-0.396424	0.561337

C	2.851366	1.406314	0.245926
H	1.785984	1.677744	0.235682
C	3.213285	0.952451	1.684557
H	3.778330	1.730825	2.213797
H	2.930087	-2.347885	1.544158
H	2.814782	-1.929668	-0.821833
C	3.670421	2.634734	-0.147995
H	4.753495	2.453375	-0.052138
H	3.472320	2.953066	-1.181014
H	3.418445	3.477018	0.513262
O	2.104482	0.623124	2.490156
O	0.375248	-2.432373	-0.178437
C	0.672292	-3.346574	-1.202546
H	1.746005	-3.597189	-1.255974
H	0.120940	-4.267413	-0.965735
H	0.344170	-2.997289	-2.196026
C	-2.905193	-0.036742	-0.037778
H	-3.449789	0.362745	-0.913675
B	-2.606078	2.829149	-0.669754
F	-3.813070	2.884234	0.069278
F	-2.922735	2.273063	-1.935904
F	-2.093495	4.111693	-0.828291
N	-4.248482	0.733389	1.929009
N	-3.656650	0.431516	1.052032
Si	-3.234063	-1.996868	-0.114868
C	-2.696452	-2.525011	-1.814273
H	-2.830800	-3.615163	-1.910433
H	-3.321444	-2.035998	-2.578157
H	-1.646672	-2.283095	-2.014774
C	-2.407637	-2.813011	1.337387
H	-2.705265	-2.332895	2.283735
H	-2.731195	-3.866502	1.383682
H	-1.314330	-2.783272	1.234821
C	-5.097524	-2.105996	0.030803
H	-5.471729	-1.817813	1.026154
H	-5.604376	-1.487522	-0.727354
H	-5.400489	-3.153072	-0.138384

65

26a-B_18 Eopt -1992.385261

C	-1.390859	0.832210	0.167301
C	-0.815891	0.239623	-1.106211
C	0.279835	-0.519401	-1.118982
C	0.910816	-0.887260	0.198850
C	0.759474	0.211771	1.220345
C	-0.307006	1.013135	1.241023
C	1.034549	-0.979948	-2.329157
C	2.428524	-1.090572	0.092374
C	3.093608	-0.165069	-0.976979
C	2.435306	-0.336288	-2.363569
C	4.543917	-0.617392	-0.954450
C	4.277472	0.038113	1.296841
C	2.981000	-0.755004	1.487848
H	1.139213	-2.076187	-2.294178
H	-1.322712	0.536545	-2.025341
H	3.098637	-0.932565	-3.005001
H	4.901282	0.077231	2.196707
O	-1.931088	2.069180	-0.078241
C	-0.530029	2.104093	2.240989
H	-1.551763	2.057531	2.650828
H	0.188908	2.045988	3.068150
H	-0.432292	3.088601	1.760780
C	2.007485	0.297544	2.073862
H	1.800784	0.158534	3.144857
H	0.488411	-0.735946	-3.251165
H	2.346172	0.649913	-2.838241
O	5.190126	-1.060152	-1.865931

O	5.103792	-0.484452	0.261824
C	3.143458	1.333690	-0.506957
H	2.114828	1.722813	-0.509006
C	3.671355	1.404588	0.948943
H	4.373974	2.238815	1.074994
H	3.089849	-1.641135	2.122495
H	2.651163	-2.127950	-0.193161
C	3.997711	2.209598	-1.423115
H	5.062892	1.929246	-1.380828
H	3.677151	2.157077	-2.472732
H	3.922694	3.260218	-1.105567
O	2.663674	1.555636	1.921029
O	0.285024	-2.027619	0.800947
C	0.185214	-3.203493	0.037320
H	1.160603	-3.546841	-0.349257
H	-0.204541	-3.983668	0.705616
H	-0.509187	-3.090972	-0.813402
C	-2.566562	-0.131021	0.785801
H	-3.311478	0.562888	1.218963
B	-3.229407	2.334578	-0.665150
F	-3.228193	3.627432	-1.175785
F	-4.241352	2.218192	0.325374
F	-3.532101	1.405932	-1.690844
N	-1.708865	-1.430330	2.728475
N	-2.096613	-0.839889	1.882765
Si	-3.699265	-1.418828	-0.276353
C	-5.422113	-0.714107	-0.200113
H	-5.743491	-0.545561	0.839751
H	-5.485873	0.235883	-0.746255
H	-6.118639	-1.435993	-0.658157
C	-3.121622	-1.709696	-2.021505
H	-2.111576	-2.138104	-2.078166
H	-3.826301	-2.439510	-2.457257
H	-3.162833	-0.791959	-2.621537
C	-3.631745	-3.024979	0.688508
H	-4.035283	-2.928754	1.709496
H	-4.266361	-3.753787	0.156739
H	-2.619937	-3.455686	0.746365

65

26a-B_19 Eopt -1992.385095

C	-1.392292	0.691601	0.374853
C	-0.852005	0.288915	-0.985406
C	0.246741	-0.450777	-1.146653
C	0.931486	-1.015991	0.070663
C	0.772185	-0.122078	1.274234
C	-0.298326	0.654057	1.454706
C	0.990246	-0.652279	-2.433755
C	2.452267	-1.122096	-0.111253
C	3.043039	0.020431	-0.998293
C	2.341568	0.088187	-2.373196
C	4.508707	-0.366291	-1.101279
C	4.293573	-0.165519	1.240815
C	3.034388	-1.035502	1.308424
H	1.170356	-1.726738	-2.594539
H	-1.357685	0.744114	-1.837480
H	3.016199	-0.313342	-3.141838
H	4.945476	-0.272179	2.114924
O	-1.883731	1.970931	0.361442
C	-0.501728	1.543311	2.642347
H	-1.508314	1.405072	3.069306
H	0.242790	1.348098	3.424196
H	-0.430425	2.599600	2.346018
C	2.039102	-0.156527	2.106329
H	1.866985	-0.507958	3.133797
H	0.405014	-0.287762	-3.289703
H	2.163503	1.141032	-2.630476

O	5.139405	-0.600281	-2.097197
O	5.103541	-0.444844	0.103362
C	3.054655	1.402228	-0.250684
H	2.013810	1.743259	-0.153672
C	3.625862	1.215115	1.178321
H	4.299920	2.039230	1.446127
H	3.196497	-2.020580	1.759107
H	2.708411	-2.072450	-0.599386
C	3.848254	2.468623	-1.005015
H	4.923768	2.229938	-1.040859
H	3.497591	2.597962	-2.038248
H	3.744081	3.437906	-0.495198
O	2.645820	1.132677	2.186656
O	0.382593	-2.273821	0.479178
C	0.346674	-3.316099	-0.462357
H	-0.289908	-3.075419	-1.331830
H	1.349352	-3.595674	-0.830360
H	-0.080099	-4.189190	0.050196
C	-2.608525	-0.296562	0.852370
H	-3.248887	0.313933	1.511175
B	-2.995601	2.512029	-0.399446
F	-3.178247	3.828422	0.016182
F	-4.178480	1.767932	-0.135740
F	-2.769875	2.486700	-1.792109
N	-1.714894	-2.127027	2.288219
N	-2.121748	-1.301543	1.681893
Si	-3.884456	-1.214320	-0.405663
C	-5.565003	-0.933193	0.352667
H	-5.853272	0.125618	0.303911
H	-6.313985	-1.528130	-0.195903
H	-5.584622	-1.259441	1.405083
C	-3.731033	-0.630943	-2.165418
H	-4.618366	-1.015407	-2.697613
H	-3.718692	0.462014	-2.256853
H	-2.838577	-1.049003	-2.653805
C	-3.459006	-3.037248	-0.318305
H	-3.658386	-3.487450	0.667341
H	-4.098636	-3.560693	-1.048863
H	-2.413602	-3.239009	-0.596031

65

26a-B_2 Eopt -1992.392354

C	1.399398	0.519035	-0.292440
C	0.538144	1.394664	0.615961
C	-0.589974	0.936403	1.161355
C	-1.015023	-0.483975	0.856048
C	-0.612372	-0.893624	-0.539344
C	0.504251	-0.434826	-1.111386
C	-1.568370	1.727029	1.977077
C	-2.541091	-0.653315	0.872827
C	-3.312911	0.624686	0.408539
C	-2.911885	1.864306	1.235751
C	-4.765932	0.228317	0.611030
C	-4.081743	-1.451353	-0.898274
C	-2.827399	-1.812033	-0.097021
H	-1.733169	1.211155	2.936237
H	0.895839	2.412689	0.783960
H	-3.708576	2.087812	1.958319
H	-4.541110	-2.310564	-1.399355
O	2.182288	1.235194	-1.153370
C	0.896121	-0.716916	-2.527165
H	0.231189	-1.460778	-2.982990
H	0.839087	0.209346	-3.118540
H	1.932979	-1.070772	-2.608236
C	-1.700430	-1.747823	-1.157501
H	-1.330598	-2.736703	-1.464857
H	-1.167840	2.723229	2.211057

H	-2.845836	2.732518	0.566828
O	-5.583331	0.766131	1.308734
O	-5.103545	-0.862973	-0.099686
C	-3.185815	0.862541	-1.139230
H	-2.149287	1.162898	-1.351854
C	-3.453854	-0.464328	-1.892323
H	-4.067601	-0.295270	-2.786837
H	-2.885580	-2.780933	0.410465
H	-2.875826	-0.883441	1.894533
C	-4.125348	1.956631	-1.645900
H	-5.183469	1.662252	-1.553031
H	-3.992598	2.907199	-1.111255
H	-3.930675	2.145990	-2.711974
O	-2.286559	-1.134119	-2.305052
O	-0.403761	-1.453092	1.722073
C	-0.436503	-1.212446	3.108184
H	-1.463009	-1.074450	3.489387
H	-0.008203	-2.100001	3.593592
H	0.168949	-0.332107	3.389652
C	2.260874	-0.456351	0.658549
H	1.540627	-1.162946	1.120325
B	3.189173	2.216968	-0.783714
F	3.905535	2.534485	-1.933952
F	4.073446	1.674716	0.194390
F	2.623903	3.386451	-0.238520
N	3.115683	0.755215	2.669420
N	2.743906	0.234682	1.773471
Si	3.695145	-1.605894	-0.082824
C	2.777733	-3.176336	-0.517682
H	2.268747	-3.594908	0.365197
H	2.027841	-3.028190	-1.308829
H	3.501830	-3.925714	-0.878674
C	4.593846	-0.853317	-1.527455
H	5.203052	-1.642771	-1.999068
H	3.916903	-0.432351	-2.281906
H	5.262136	-0.049472	-1.189991
C	4.856057	-1.889102	1.355362
H	5.385840	-0.966076	1.641951
H	4.339730	-2.291892	2.241715
H	5.620284	-2.624540	1.053428

65

26a-B_3 Eopt -1992.392354

C	1.399357	0.518714	-0.292400
C	0.538140	1.394150	0.616210
C	-0.590001	0.935838	1.161506
C	-1.015137	-0.484443	0.855905
C	-0.612493	-0.893799	-0.539575
C	0.504177	-0.434970	-1.111503
C	-1.568261	1.726357	1.977485
C	-2.541222	-0.653702	0.872660
C	-3.312964	0.624487	0.408777
C	-2.911940	1.863778	1.236471
C	-4.766033	0.228168	0.611058
C	-4.081907	-1.451075	-0.898726
C	-2.827619	-1.812112	-0.097540
H	-1.732870	1.210346	2.936605
H	0.895858	2.412122	0.784452
H	-3.708513	2.086811	1.959313
H	-4.541315	-2.310089	-1.400108
O	2.182253	1.235030	-1.153205
C	0.896029	-0.716809	-2.527335
H	0.231265	-1.460796	-2.983200
H	0.838730	0.209495	-3.118619
H	1.932973	-1.070384	-2.608468
C	-1.700630	-1.747708	-1.157983
H	-1.330913	-2.736535	-1.465646

H	-1.167660	2.722512	2.211538
H	-2.846202	2.732323	0.567954
O	-5.583433	0.765851	1.308863
O	-5.103698	-0.862875	-0.100001
C	-3.185733	0.862828	-1.138903
H	-2.149149	1.163102	-1.351369
C	-3.453900	-0.463782	-1.892433
H	-4.067614	-0.294366	-2.786902
H	-2.885908	-2.781155	0.409660
H	-2.875945	-0.884119	1.894304
C	-4.125060	1.957211	-1.645323
H	-3.930322	2.146793	-2.711345
H	-5.183239	1.663008	-1.552550
H	-3.992142	2.907628	-1.110450
O	-2.286673	-1.133568	-2.305353
O	-0.403911	-1.453780	1.721707
C	-0.436670	-1.213457	3.107874
H	-1.463188	-1.075650	3.489115
H	-0.008283	-2.101085	3.593075
H	0.168701	-0.333133	3.389552
C	2.260936	-0.456842	0.658353
H	1.540842	-1.163810	1.119792
B	3.188621	2.217241	-0.783309
F	3.905221	2.535040	-1.933317
F	4.072785	1.675355	0.195141
F	2.622781	3.386553	-0.238330
N	3.115096	0.754390	2.669718
N	2.743593	0.233957	1.773598
Si	3.695885	-1.605459	-0.083182
C	4.594622	-0.851695	-1.527113
H	5.257836	-0.043642	-1.189688
H	5.209029	-1.639197	-1.995241
H	3.917595	-0.435956	-2.284394
C	4.856675	-1.888441	1.355161
H	5.621129	-2.623680	1.053324
H	5.386179	-0.965283	1.641844
H	4.340342	-2.291382	2.241443
C	2.779452	-3.176232	-0.518844
H	2.029436	-3.028097	-1.309880
H	3.503990	-3.924971	-0.880275
H	2.270755	-3.595593	0.363830

65

26a-B_4 Eopt -1992.392870

C	1.556435	0.553227	0.214952
C	0.615372	0.917469	1.360038
C	-0.489308	0.215927	1.602782
C	-0.813729	-0.963982	0.713378
C	-0.332424	-0.739095	-0.703269
C	0.763889	-0.021051	-0.969542
C	-1.492045	0.453956	2.685432
C	-2.336032	-1.183075	0.561761
C	-3.149454	0.141930	0.729070
C	-2.871531	0.799675	2.094374
C	-4.597391	-0.304384	0.605164
C	-3.766725	-1.028790	-1.471640
C	-2.553632	-1.735323	-0.861214
H	-1.566260	-0.473807	3.277379
H	0.918466	1.760153	1.986331
H	-3.659785	0.501253	2.798551
H	-4.158297	-1.535724	-2.360614
O	2.406997	1.573686	-0.138292
C	1.188817	0.375967	-2.348340
H	0.740374	-0.273118	-3.111499
H	0.858078	1.407468	-2.539771
H	2.280437	0.369103	-2.462971
C	-1.376098	-1.186954	-1.702534

H	-0.984069	-1.909705	-2.434009
H	-1.159807	1.249667	3.366182
H	-2.948194	1.889583	1.989808
O	-5.474291	-0.172910	1.416716
O	-4.856728	-0.907166	-0.567642
C	-2.920401	1.105977	-0.492696
H	-1.878171	1.456059	-0.461092
C	-3.111299	0.314339	-1.813586
H	-3.677977	0.902665	-2.547163
H	-2.629416	-2.827957	-0.886193
H	-2.680049	-1.883778	1.336753
C	-3.840385	2.325566	-0.471312
H	-4.900641	2.043381	-0.579161
H	-3.739083	2.912369	0.451695
H	-3.589275	2.989474	-1.311742
O	-1.910494	-0.081976	-2.433687
O	-0.165091	-2.070022	1.353583
C	-0.294769	-3.333214	0.749814
H	-1.313525	-3.744635	0.855431
H	-0.028649	-3.314893	-0.322005
H	0.400028	-4.009218	1.267460
C	2.500313	-0.605853	0.758893
H	1.882108	-1.403831	1.214589
B	1.942724	2.951268	-0.296239
F	0.597505	2.986468	-0.723741
F	2.762014	3.567283	-1.246500
F	2.041595	3.647957	0.926272
N	3.817297	0.482108	2.586004
N	3.221813	-0.031646	1.815833
Si	3.840632	-1.490505	-0.408217
C	4.830863	-2.528644	0.792785
H	5.559235	-3.133348	0.226995
H	5.402387	-1.917622	1.509836
H	4.187473	-3.222718	1.357175
C	2.888562	-2.592667	-1.570467
H	3.617844	-3.229613	-2.099101
H	2.210053	-3.255111	-1.010906
H	2.309246	-2.046890	-2.326470
C	4.897746	-0.176328	-1.196010
H	5.434446	0.394852	-0.421535
H	5.650252	-0.651426	-1.847025
H	4.312738	0.538572	-1.790245

65

26a-B_5 Eopt -1992.391287

C	1.440922	0.478680	-0.322701
C	0.519751	1.386537	0.488035
C	-0.653235	0.976324	0.979938
C	-1.033077	-0.485489	0.760994
C	-0.577881	-0.911387	-0.622466
C	0.576756	-0.492809	-1.149418
C	-1.606460	1.914396	1.684407
C	-2.548574	-0.776067	0.777811
C	-3.405280	0.489672	0.520833
C	-3.091891	1.510060	1.612322
C	-4.840537	0.008582	0.623621
C	-4.058387	-1.383889	-1.096307
C	-2.811317	-1.822940	-0.322902
H	-1.316480	1.993317	2.744879
H	0.859833	2.417736	0.603920
H	-3.401227	1.054752	2.564846
H	-4.463551	-2.169984	-1.743344
O	2.255406	1.170010	-1.177098
C	1.009280	-0.785973	-2.551400
H	0.916286	0.122578	-3.165302
H	2.064200	-1.087378	-2.603121
H	0.395295	-1.574518	-3.004592

C	-1.685308	-1.631931	-1.352612
H	-1.354714	-2.566732	-1.827106
H	-1.462527	2.917662	1.258134
H	-3.719594	2.403800	1.494863
O	-5.685077	0.404355	1.381862
O	-5.125698	-0.968566	-0.253523
C	-3.232156	0.980431	-0.953553
H	-2.191881	1.311428	-1.090304
C	-3.442691	-0.231896	-1.903069
H	-4.041833	0.054189	-2.777886
H	-2.867949	-2.844473	0.069571
H	-2.832425	-1.164594	1.765256
C	-4.153044	2.144798	-1.305379
H	-3.964821	2.472607	-2.338637
H	-5.217320	1.866240	-1.235742
H	-3.985214	3.008793	-0.646683
O	-2.243316	-0.799287	-2.379677
O	-0.376679	-1.353672	1.697712
C	-0.470258	-0.996194	3.056733
H	-1.515200	-0.853241	3.382940
H	-0.039990	-1.824039	3.636440
H	0.094842	-0.074136	3.282872
C	2.268914	-0.448320	0.693522
H	1.530528	-1.131494	1.163031
B	3.239643	2.175314	-0.811125
F	2.647286	3.355524	-0.319193
F	3.982781	2.464211	-1.952493
F	4.104004	1.677684	0.206987
N	3.062827	0.857664	2.669290
N	2.719562	0.292999	1.788684
Si	3.714420	-1.632512	0.038804
C	4.697873	-0.917359	-1.369294
H	4.065569	-0.500742	-2.164073
H	5.359578	-0.116613	-1.012094
H	5.319143	-1.722809	-1.796052
C	4.793966	-1.915422	1.539414
H	5.552012	-2.677873	1.294501
H	5.333357	-1.000188	1.832638
H	4.220999	-2.282584	2.406282
C	2.792752	-3.192483	-0.423050
H	2.100994	-3.045964	-1.265547
H	3.523551	-3.963309	-0.719730
H	2.217251	-3.581888	0.431798

65

26a-B_6 Eopt -1992.391541

C	1.563724	0.514980	0.245950
C	0.639825	0.753916	1.437572
C	-0.474692	0.043823	1.610170
C	-0.809964	-1.026697	0.593587
C	-0.340178	-0.649507	-0.790115
C	0.752280	0.091633	-0.989354
C	-1.520558	0.272667	2.659098
C	-2.327810	-1.212591	0.428442
C	-3.134109	0.111995	0.639619
C	-2.831539	0.753669	2.010059
C	-4.576945	-0.349338	0.518761
C	-3.753828	-1.003369	-1.591322
C	-2.522699	-1.709022	-1.014417
H	-1.705649	-0.668557	3.200142
H	0.939060	1.545786	2.129112
H	-3.672042	0.561766	2.690932
H	-4.151531	-1.487429	-2.490209
O	2.422156	1.562095	0.011677
C	1.174548	0.624495	-2.322431
H	0.719668	0.059169	-3.146016
H	0.845815	1.671140	-2.406235

H	2.265133	0.629089	-2.443821
C	-1.354945	-1.090890	-1.822009
H	-0.923638	-1.776185	-2.566434
H	-1.178490	1.009560	3.399117
H	-2.770735	1.842860	1.886660
O	-5.448695	-0.256139	1.341010
O	-4.835601	-0.923869	-0.669516
C	-2.939931	1.114718	-0.555061
H	-1.906854	1.490520	-0.517250
C	-3.123937	0.362589	-1.897547
H	-3.705655	0.961656	-2.610363
H	-2.562666	-2.801618	-1.082402
H	-2.699938	-1.940568	1.163082
C	-3.894348	2.306457	-0.484506
H	-4.944590	1.998834	-0.617252
H	-3.820310	2.849608	0.467536
H	-3.655928	3.017190	-1.289606
O	-1.917334	0.012092	-2.531493
O	-0.171178	-2.278850	0.885319
C	-0.328983	-2.817258	2.175219
H	0.150355	-3.805827	2.165647
H	0.160948	-2.198547	2.948300
H	-1.388297	-2.954507	2.452897
C	2.496214	-0.718515	0.616115
H	1.874515	-1.577257	0.934445
B	1.967808	2.952241	0.006473
F	0.615447	3.043748	-0.389829
F	2.775337	3.659749	-0.888174
F	2.094402	3.515111	1.293971
N	3.825113	0.072014	2.580550
N	3.222350	-0.317691	1.745686
Si	3.820062	-1.449239	-0.672537
C	4.881086	-0.052333	-1.294958
H	4.297289	0.736620	-1.788023
H	5.425445	0.411417	-0.456652
H	5.626398	-0.444652	-2.006466
C	4.816537	-2.643508	0.367528
H	5.541473	-3.158391	-0.285032
H	5.393115	-2.143814	1.162663
H	4.177771	-3.414287	0.827700
C	2.831232	-2.386198	-1.945344
H	2.309908	-1.738655	-2.662302
H	3.528511	-3.026214	-2.511799
H	2.091445	-3.041234	-1.458909

65

26a-B_7 Eopt -1992.392550

C	-1.401521	0.554538	0.206240
C	-0.518943	1.336396	-0.765489
C	0.603702	0.817351	-1.262389
C	1.022706	-0.572192	-0.834315
C	0.597793	-0.856259	0.589165
C	-0.526548	-0.344852	1.103135
C	1.540411	1.460133	-2.234795
C	2.556641	-0.748067	-0.816531
C	3.317895	0.594431	-0.569471
C	2.942841	1.650931	-1.626060
C	4.781879	0.194369	-0.660842
C	4.082101	-1.204051	1.097811
C	2.863442	-1.727029	0.334065
H	1.607676	0.793786	-3.111198
H	-0.881833	2.323618	-1.059005
H	3.695288	1.635438	-2.425910
H	4.534855	-1.955577	1.754180
O	-2.171319	1.347250	1.010707
C	-0.917818	-0.480793	2.540981
H	-0.833874	0.494371	3.043275

H	-1.963170	-0.798500	2.654591
H	-0.273166	-1.197170	3.064557
C	1.711260	-1.544951	1.349665
H	1.389369	-2.485320	1.821167
H	1.145082	2.422110	-2.588627
H	2.998407	2.648599	-1.171607
O	5.608667	0.615820	-1.424340
O	5.119625	-0.742482	0.242333
C	3.132628	1.094248	0.909380
H	2.083044	1.398282	1.035727
C	3.402162	-0.079122	1.886887
H	3.982666	0.257129	2.755927
H	2.977231	-2.761628	-0.007117
H	2.883637	-1.137906	-1.791593
C	4.030081	2.282456	1.253819
H	5.097754	2.008999	1.232281
H	3.885934	3.131738	0.572257
H	3.802393	2.633313	2.271324
O	2.239750	-0.704677	2.378256
O	0.394316	-1.443843	-1.782978
C	0.609163	-2.827036	-1.636771
H	-0.067056	-3.330398	-2.341542
H	1.642913	-3.118589	-1.889660
H	0.376492	-3.181487	-0.616705
C	-2.282323	-0.455929	-0.685892
H	-1.569760	-1.169431	-1.146174
B	-3.171247	2.311836	0.583135
F	-2.604107	3.408280	-0.094735
F	-3.837657	2.752254	1.723104
F	-4.104637	1.699593	-0.304092
N	-3.168830	0.683024	-2.727508
N	-2.784692	0.197258	-1.817858
Si	-3.704633	-1.562088	0.133854

C	-4.871432	-1.950796	-1.275056
H	-5.414701	-1.055421	-1.618583
H	-4.354051	-2.403652	-2.136282
H	-5.624562	-2.674407	-0.921085
C	-2.772878	-3.095588	0.661618
H	-3.486298	-3.817536	1.092951
H	-2.289243	-3.576945	-0.203376
H	-2.004199	-2.891285	1.421514
C	-4.597717	-0.716500	1.530589
H	-5.294449	0.037462	1.139370
H	-5.175271	-1.477308	2.082081
H	-3.919217	-0.212034	2.230415

65

26a-B_8 Eopt -1992.392755

C	-1.575085	0.479984	0.437800
C	-1.054406	0.083550	-0.930551
C	0.034513	-0.661888	-1.119400
C	0.730422	-1.263967	0.073766
C	0.567451	-0.421184	1.313806
C	-0.487776	0.371521	1.519225
C	0.768623	-0.820087	-2.417409
C	2.251122	-1.342849	-0.124170
C	2.815767	-0.156880	-0.969091
C	2.099698	-0.045169	-2.334224
C	4.286391	-0.511111	-1.099746
C	4.087919	-0.412090	1.250567
C	2.843769	-1.305098	1.293167
H	0.973257	-1.884133	-2.611364
H	-1.566099	0.550277	-1.775018
H	2.777703	-0.389809	-3.127236
H	4.749466	-0.543522	2.114114
O	-2.148838	1.735779	0.426916
C	-0.654970	1.247561	2.721684

H	-0.446170	2.293861	2.454540
H	-1.687933	1.213770	3.101291
H	0.027789	0.958387	3.530624
C	1.841352	-0.476294	2.135708
H	1.683458	-0.874802	3.148364
H	0.171619	-0.441104	-3.258739
H	1.885189	1.012654	-2.536870
O	4.913965	-0.690790	-2.109301
O	4.892593	-0.630392	0.095983
C	2.801957	1.191148	-0.162340
H	1.756025	1.504859	-0.041282
C	3.395870	0.957554	1.249888
H	4.058512	1.782426	1.542753
H	3.025731	-2.305304	1.700876
H	2.512425	-2.272389	-0.649049
C	3.555201	2.311291	-0.878475
H	4.637291	2.108527	-0.938766
H	3.185187	2.475778	-1.899930
H	3.425730	3.253553	-0.325626
O	2.431739	0.815901	2.267867
O	0.218684	-2.555402	0.425476
C	0.068173	-3.507577	-0.597009
H	1.014087	-3.725511	-1.123275
H	-0.273307	-4.435770	-0.117939
H	-0.685888	-3.202020	-1.344304
C	-2.832446	-0.402424	0.862080
H	-3.298725	0.090380	1.733349
B	-1.543303	2.877479	-0.251578
F	-2.039939	4.034014	0.354729
F	-1.886756	2.892992	-1.624667
F	-0.135812	2.847338	-0.148792
N	-2.147244	-2.676068	1.626639
N	-2.458524	-1.663418	1.325879

Si	-4.283048	-0.672903	-0.474534
C	-4.689800	0.996722	-1.177213
H	-5.070743	1.666680	-0.391539
H	-3.817819	1.480908	-1.637618
H	-5.478803	0.872539	-1.938054
C	-3.675174	-1.916264	-1.728204
H	-2.891611	-1.505943	-2.381066
H	-3.291593	-2.831528	-1.248399
H	-4.526701	-2.212564	-2.363828
C	-5.667053	-1.391397	0.554523
H	-5.997959	-0.684605	1.331981
H	-6.532868	-1.612833	-0.091198
H	-5.369393	-2.333287	1.043688

65

26a-B_9 Eopt -1992.392028

C	1.587421	0.359974	-0.476748
C	1.047116	0.160372	0.924964
C	-0.045824	-0.548045	1.200227
C	-0.757186	-1.295368	0.102345
C	-0.565030	-0.634855	-1.245053
C	0.512768	0.100785	-1.545244
C	-0.736271	-0.636809	2.523138
C	-2.288537	-1.301596	0.302795
C	-2.792481	-0.039319	1.070743
C	-2.093820	0.090137	2.441322
C	-4.286275	-0.277285	1.212314
C	-4.080103	-0.345267	-1.131910
C	-2.906436	-1.327855	-1.108460
H	-0.893313	-1.700707	2.764456
H	1.575591	0.703262	1.711031
H	-2.765444	-0.291711	3.222197
H	-4.744812	-0.486968	-1.991458
O	2.164203	1.602749	-0.646677

C	0.687105	0.829285	-2.842397
H	1.737597	0.814707	-3.169178
H	0.061437	0.399805	-3.635637
H	0.404195	1.884475	-2.715618
C	-1.863434	-0.650150	-2.030386
H	-1.773405	-1.125320	-3.018604
H	-0.124848	-0.201815	3.325694
H	-1.930211	1.153179	2.661727
O	-4.925390	-0.339317	2.228650
O	-4.901004	-0.428897	0.026016
C	-2.673099	1.247412	0.175714
H	-1.607036	1.469645	0.033604
C	-3.288063	0.963536	-1.220293
H	-3.887445	1.815307	-1.567361
H	-3.180211	-2.336909	-1.434837
H	-2.572669	-2.183698	0.894900
C	-3.330067	2.475471	0.803262
H	-2.937428	2.693344	1.805954
H	-3.131331	3.356631	0.175393
H	-4.424138	2.364433	0.883896
O	-2.347143	0.678961	-2.231478
O	-0.207416	-2.615285	0.164658
C	-0.760708	-3.597830	-0.674131
H	-0.149475	-4.502852	-0.552839
H	-1.797555	-3.855604	-0.399052
H	-0.737332	-3.305870	-1.740288
C	2.851308	-0.573084	-0.735224
H	3.322216	-0.248035	-1.679746
B	1.576581	2.837964	-0.139454
F	0.172341	2.836348	-0.283980
F	2.127554	3.890620	-0.874436
F	1.880773	3.022421	1.230235
N	2.204281	-2.959558	-1.050711
N	2.489477	-1.900373	-0.955568
Si	4.296782	-0.594097	0.640372
C	4.743652	1.174855	0.981211
H	3.886097	1.748277	1.358658
H	5.550120	1.191431	1.733765
H	5.110949	1.670953	0.070195
C	3.661966	-1.528542	2.128172
H	3.105899	-2.438664	1.849849
H	4.526037	-1.841499	2.738046
H	3.008939	-0.907758	2.758103
C	5.660645	-1.544556	-0.213010
H	6.533527	-1.612199	0.457302
H	5.355176	-2.574102	-0.461627
H	5.984796	-1.042459	-1.138333

65

26b-Al'_1 Eopt -3290.928760

C	1.002645	0.210342	0.094653
C	0.177686	0.729206	1.263164
C	-0.996896	0.211051	1.624313
C	-1.502336	-1.033227	0.946256
C	-0.999630	-1.152642	-0.468071
C	0.159846	-0.632934	-0.885046
C	-1.971688	0.779850	2.606199
C	-3.026497	-1.058690	0.820323
C	-3.646113	0.357613	0.664631
C	-3.221553	1.270713	1.839191
C	-5.140185	0.085600	0.639936
C	-4.448437	-1.231627	-1.194478
C	-3.319791	-1.918019	-0.420444
H	-2.256731	-0.009257	3.320091
H	0.585641	1.580772	1.814581
H	-4.064601	1.369924	2.536623
H	-4.927277	-1.878992	-1.937472

O	2.128288	-0.427780	0.525889
C	0.642392	-0.791342	-2.300978
H	0.342671	0.060074	-2.934453
H	1.737271	-0.874327	-2.358405
H	0.221277	-1.694225	-2.760916
C	-2.091413	-1.738032	-1.344791
H	-1.792754	-2.655406	-1.871951
H	-1.536885	1.608155	3.183029
H	-3.014303	2.279205	1.454251
O	-5.978080	0.522903	1.381956
O	-5.492185	-0.750892	-0.353070
C	-3.332667	0.957341	-0.750992
H	-2.254905	1.163890	-0.787961
C	-3.635337	-0.104360	-1.843629
H	-4.135362	0.353080	-2.707419
H	-3.520406	-2.964893	-0.169542
H	-3.435089	-1.510415	1.733974
C	-4.090151	2.252689	-1.032968
H	-3.755115	2.680587	-1.989753
H	-5.175815	2.080547	-1.112722
H	-3.927737	3.009939	-0.253160
O	-2.498056	-0.780405	-2.328941
O	-1.213756	-2.193627	1.724020
C	0.129492	-2.580669	1.872542
H	0.595953	-2.857561	0.912891
H	0.746717	-1.797174	2.343328
H	0.124468	-3.463699	2.526529
C	1.533306	1.508109	-0.701850
H	2.165733	1.182446	-1.546011
N	-0.427411	2.709056	-1.660641
N	0.459886	2.176201	-1.279680
Si	2.647105	2.874940	0.252304
C	1.484441	4.181810	0.913905
H	0.920912	4.689353	0.114448
H	0.769866	3.798802	1.657781
H	2.098631	4.951245	1.411705
C	3.630891	2.046314	1.592612
H	4.400080	1.382159	1.173879
H	4.141276	2.841868	2.161932
H	3.007915	1.464887	2.285468
C	3.709274	3.566608	-1.114449
H	4.344419	4.375056	-0.716366
H	4.367172	2.789546	-1.533371
H	3.098755	3.988792	-1.928931
Al	3.468650	-1.410755	-0.001893
Cl	2.839150	-3.301495	-0.840084
Cl	4.537879	-0.290711	-1.543113
Cl	4.734477	-1.731695	1.708698

65

26b-Al'_2 Eopt -3290.928758

C	1.002567	0.210713	0.094649
C	0.177481	0.727897	1.263886
C	-0.996934	0.208907	1.624399
C	-1.502201	-1.034478	0.944484
C	-0.999546	-1.151649	-0.470060
C	0.159983	-0.631434	-0.886151
C	-1.971498	0.775618	2.607670
C	-3.026383	-1.060154	0.818660
C	-3.646344	0.356296	0.665800
C	-3.222171	1.267042	1.842298
C	-5.140390	0.084133	0.640444
C	-4.448346	-1.229427	-1.196435
C	-3.319655	-1.917198	-0.423710
H	-2.255671	-0.014829	3.320438
H	0.585466	1.578432	1.816903
H	-4.065049	1.363685	2.540293

H	-4.927034	-1.875380	-1.940754
O	2.128093	-0.427731	0.525517
C	0.642903	-0.787926	-2.302149
H	0.343382	0.064277	-2.934644
H	1.737846	-0.870730	-2.359161
H	0.222090	-1.690244	-2.763464
C	-2.091347	-1.735422	-1.347768
H	-1.792721	-2.651729	-1.876807
H	-1.536789	1.603192	3.185619
H	-3.016261	2.276676	1.459655
O	-5.978417	0.520082	1.383117
O	-5.492235	-0.750486	-0.354179
C	-3.332836	0.958872	-0.748607
H	-2.255096	1.165691	-0.785035
C	-3.635370	-0.100788	-1.843313
H	-4.135462	0.358282	-2.706200
H	-3.520182	-2.964557	-0.174759
H	-3.434718	-1.513754	1.731491
C	-4.090394	2.254696	-1.028157
H	-3.755521	2.684273	-1.984249
H	-5.176081	2.082736	-1.108005
H	-3.927818	3.010589	-0.247066
O	-2.498060	-0.775797	-2.329981
O	-1.213197	-2.196021	1.720429
C	0.130118	-2.583249	1.867989
H	0.596066	-2.859316	0.907837
H	0.747612	-1.800231	2.339196
H	0.125338	-3.466876	2.521173
C	1.533165	1.509122	-0.700705
H	2.165408	1.184481	-1.545360
N	-0.427086	2.711831	-1.658315
N	0.459868	2.178046	-1.277996
Si	2.647650	2.875210	0.253438

C	1.485865	4.178542	0.923359
H	2.100834	4.945948	1.423340
H	0.921155	4.689464	0.126867
H	0.772445	3.792588	1.666742
C	3.639040	2.043111	1.585803
H	4.155030	2.836864	2.152561
H	3.020029	1.461554	2.282067
H	4.403867	1.378546	1.159712
C	3.702717	3.573160	-1.115687
H	4.337964	4.381617	-0.717775
H	4.360356	2.798918	-1.540156
H	3.087706	3.996815	-1.926033
Al	3.468045	-1.411645	-0.002229
Cl	2.838739	-3.299017	-0.848098
Cl	4.728512	-1.739816	1.710977
Cl	4.542317	-0.286834	-1.536340

65

26b-Al'_3 Eopt -3290.921270

C	1.267420	0.356807	-0.246019
C	0.533556	-0.019563	1.037310
C	-0.611611	-0.705523	1.029318
C	-1.129758	-1.162823	-0.324720
C	-0.881023	-0.076683	-1.352780
C	0.256703	0.621674	-1.376778
C	-1.384716	-1.031648	2.282700
C	-2.648294	-1.429489	-0.384477
C	-3.428568	-0.698327	0.735100
C	-2.909434	-1.189321	2.086330
C	-4.877761	-1.095019	0.532438
C	-4.412824	-0.142334	-1.563576
C	-3.120513	-0.939022	-1.768185
H	-0.985061	-1.966659	2.708660
H	0.984316	0.283703	1.987502

H	-3.181711	-2.252503	2.156351
H	-4.953610	0.051938	-2.496707
O	2.187317	-0.574007	-0.669491
C	0.599946	1.583355	-2.474791
H	0.756742	2.613166	-2.114802
H	1.517397	1.261985	-2.994465
H	-0.197204	1.635302	-3.226168
C	-2.113811	0.145667	-2.195042
H	-1.918785	0.165983	-3.276116
H	-1.172119	-0.249658	3.026349
H	-3.448242	-0.692903	2.904884
O	-5.600547	-1.636423	1.326022
O	-5.337604	-0.777132	-0.689938
C	-3.374982	0.843291	0.495059
H	-2.326164	1.153810	0.575585
C	-3.808551	1.136244	-0.969005
H	-4.485305	2.000066	-1.015477
H	-3.209364	-1.751644	-2.497669
H	-2.829210	-2.506175	-0.262269
C	-4.182998	1.649168	1.505490
H	-5.258818	1.413198	1.459646
H	-3.841248	1.469765	2.535088
H	-4.071051	2.724857	1.300489
O	-2.729312	1.396756	-1.840767
O	-0.436703	-2.306644	-0.810255
C	-0.270554	-3.368335	0.091429
H	0.413751	-3.106830	0.916581
H	-1.228770	-3.719519	0.519719
H	0.177270	-4.198268	-0.472464
C	2.082073	1.668622	0.158101
H	2.719918	1.384645	1.016470
N	3.791388	2.128650	-1.607591
N	3.036691	1.927801	-0.833291
Si	1.204087	3.367102	0.738835
C	1.755870	3.545189	2.511008
H	1.322578	4.461791	2.944050
H	1.418991	2.692484	3.121652
H	2.852281	3.618242	2.588166
C	1.904801	4.719016	-0.342814
H	1.654998	4.593905	-1.408431
H	1.475504	5.680324	-0.015044
H	2.999734	4.799126	-0.246638
C	-0.645643	3.209416	0.616239
H	-1.011052	2.851289	-0.356852
H	-1.018097	2.538460	1.404223
H	-1.075842	4.208998	0.798434
Al	3.417837	-1.598741	0.032733
Cl	5.187583	-0.336947	0.248365
Cl	3.796438	-3.220763	-1.329821
Cl	2.931938	-2.351255	2.006024

65

26b-Al'_4 Eopt -3290.921890

C	1.050482	0.267189	0.040807
C	0.215526	0.816827	1.188351
C	-0.961198	0.299988	1.542176
C	-1.449354	-0.936374	0.829970
C	-0.989211	-0.980899	-0.603125
C	0.180196	-0.473635	-0.998401
C	-1.934620	0.882324	2.521113
C	-2.980308	-0.995737	0.742993
C	-3.639160	0.413151	0.618393
C	-3.209210	1.338310	1.779045
C	-5.123917	0.094076	0.636423
C	-4.449205	-1.165858	-1.243558
C	-3.276586	-1.829092	-0.515171
H	-2.202369	0.119570	3.268313

H	0.612820	1.678837	1.730927
H	-4.038015	1.423891	2.495075
H	-4.929936	-1.816716	-1.982208
O	2.086677	-0.489776	0.505036
C	0.671409	-0.593854	-2.414546
H	0.470746	0.318542	-3.000783
H	1.754613	-0.780674	-2.459111
H	0.173659	-1.421953	-2.934527
C	-2.079999	-1.579600	-1.466820
H	-1.753750	-2.476032	-2.013141
H	-1.494846	1.730231	3.065089
H	-3.033647	2.348375	1.384124
O	-5.949593	0.484986	1.417351
O	-5.481585	-0.732866	-0.362514
C	-3.391632	1.047893	-0.796751
H	-2.324570	1.304068	-0.866966
C	-3.693182	-0.002085	-1.898405
H	-4.234300	0.452509	-2.738619
H	-3.432258	-2.889647	-0.291195
H	-3.384098	-1.469317	1.648901
C	-4.214648	2.314453	-1.028246
H	-3.931801	2.771715	-1.988236
H	-5.294041	2.096714	-1.074833
H	-4.058139	3.066294	-0.242182
O	-2.548778	-0.629005	-2.427725
O	-0.947645	-2.142731	1.395557
C	-1.018523	-2.301369	2.786943
H	-0.383750	-1.573153	3.322505
H	-2.050516	-2.228117	3.177233
H	-0.643044	-3.309811	3.008433
C	1.734245	1.531923	-0.684486
H	2.371766	1.172951	-1.511335
N	-0.079295	2.923638	-1.674170
N	0.748962	2.310282	-1.281477
Si	2.928519	2.756384	0.360129
C	1.865021	4.152086	1.008555
H	2.525243	4.856178	1.542557
H	1.378106	4.719274	0.198776
H	1.092020	3.822064	1.718954
C	3.762163	1.798994	1.715566
H	4.476490	1.068081	1.311114
H	4.324930	2.523243	2.329041
H	3.051192	1.268707	2.363564
C	4.115047	3.379473	-0.935740
H	4.804739	4.112584	-0.486020
H	4.714916	2.553664	-1.348464
H	3.586346	3.878825	-1.763814
Al	3.341365	-1.578230	-0.029768
Cl	2.570620	-3.387600	-0.913406
Cl	4.571276	-2.031326	1.678492
Cl	4.525998	-0.511938	-1.529830

65

26b-Al'_5 Eopt -3290.922142

C	1.237595	0.377713	-0.201534
C	0.559123	-0.001813	1.113295
C	-0.542495	-0.753311	1.127535
C	-1.091437	-1.233570	-0.194296
C	-0.891683	-0.205697	-1.280171
C	0.197328	0.565270	-1.325293
C	-1.353243	-1.144058	2.325061
C	-2.607495	-1.473407	-0.158669
C	-3.367023	-0.512120	0.805588
C	-2.786746	-0.577293	2.235070
C	-4.800453	-1.002890	0.722118
C	-4.403661	-0.484033	-1.548974
C	-3.076622	-1.245857	-1.605892

H	-1.402243	-2.243492	2.370665
H	1.023163	0.326743	2.048338
H	-3.454956	-1.179917	2.865389
H	-4.970216	-0.520700	-2.486042
O	2.167999	-0.544121	-0.628414
C	0.492201	1.484999	-2.472674
H	0.669015	2.526248	-2.156965
H	1.384039	1.142310	-3.023079
H	-0.343963	1.514368	-3.181930
C	-2.094410	-0.206067	-2.202389
H	-1.831163	-0.404397	-3.250940
H	-0.874340	-0.806021	3.254810
H	-2.786093	0.433075	2.665788
O	-5.495884	-1.400396	1.618308
O	-5.281940	-0.962270	-0.534148
C	-3.418286	0.947199	0.234930
H	-2.396200	1.340843	0.259689
C	-3.857529	0.917574	-1.249926
H	-4.571657	1.722217	-1.469423
H	-3.119071	-2.174365	-2.184959
H	-2.807810	-2.501047	0.176171
C	-4.318898	1.880517	1.040727
H	-4.056283	1.903641	2.107411
H	-4.227021	2.906768	0.653898
H	-5.380303	1.593273	0.962047
O	-2.789378	1.043935	-2.160485
O	-0.439117	-2.399601	-0.687933
C	-0.229385	-3.463037	0.202726
H	-1.170764	-3.862201	0.625134
H	0.252475	-4.264569	-0.374125
H	0.446553	-3.185137	1.029303
C	2.038490	1.712746	0.136863
H	2.706631	1.464681	0.982985

N	3.693662	2.134809	-1.690505
N	2.961715	1.949581	-0.891012
Si	1.164998	3.421043	0.687678
C	1.706303	3.623204	2.460018
H	1.358793	2.782651	3.081431
H	2.802688	3.689042	2.543329
H	1.277247	4.549473	2.876219
C	1.868581	4.746001	-0.423722
H	2.963305	4.829721	-0.328706
H	1.619215	4.586120	-1.485205
H	1.437024	5.716067	-0.126529
C	-0.682443	3.262257	0.553784
H	-1.033178	2.994788	-0.452784
H	-1.053478	2.519655	1.274626
H	-1.122055	4.238142	0.821256
Al	3.435683	-1.544435	0.040872
Cl	5.178496	-0.241618	0.234926
Cl	3.826872	-3.139184	-1.351238
Cl	3.014565	-2.338728	2.011036

65

26b-Al_1 Eopt -3290.937737

C	1.294747	-0.273494	-0.391497
C	0.640845	-0.338273	0.977528
C	-0.586373	-0.824283	1.149658
C	-1.329741	-1.354308	-0.054073
C	-0.981811	-0.577800	-1.305473
C	0.235368	-0.055937	-1.492390
C	-1.338626	-0.937670	2.434550
C	-2.859388	-1.193077	0.078413
C	-3.255287	0.016638	0.986058
C	-2.641010	-0.115476	2.392855
C	-4.773597	-0.046331	1.026841
C	-4.434420	0.165990	-1.290822

C	-3.392194	-0.953632	-1.348715
H	-1.580208	-2.004287	2.578976
H	1.246781	0.014562	1.816995
H	-3.383568	-0.569450	3.062618
H	-5.056309	0.222581	-2.191109
O	2.306992	0.644911	-0.459752
C	0.599345	0.815756	-2.656338
H	1.648561	0.695664	-2.958643
H	-0.049050	0.621288	-3.521068
H	0.471679	1.872933	-2.375047
C	-2.223549	-0.298069	-2.123542
H	-2.137394	-0.643611	-3.164746
H	-0.718191	-0.626563	3.286062
H	-2.439138	0.886903	2.791780
O	-5.473777	-0.162439	1.996838
O	-5.329622	0.040447	-0.193658
C	-2.926631	1.383352	0.281767
H	-1.833070	1.479228	0.212974
C	-3.486866	1.364515	-1.164751
H	-3.958856	2.322951	-1.417482
H	-3.763448	-1.868603	-1.823058
H	-3.281327	-2.101261	0.533266
C	-3.463778	2.594900	1.041314
H	-4.565533	2.601758	1.080638
H	-3.087814	2.644560	2.072192
H	-3.146134	3.516737	0.531965
O	-2.523760	1.098130	-2.157777
O	-0.929303	-2.728367	-0.128675
C	-1.485486	-3.513541	-1.154457
H	-1.341303	-3.061460	-2.152476
H	-0.963283	-4.479991	-1.133704
H	-2.561395	-3.704058	-1.000216
C	1.948614	-1.708385	-0.623168

H	1.157359	-2.465001	-0.777187
N	3.309813	-1.559801	-2.709519
N	2.677510	-1.650762	-1.811809
Si	3.152203	-2.476333	0.763304
C	3.923402	-3.943380	-0.103769
H	3.159322	-4.639940	-0.485106
H	4.579975	-3.648660	-0.938147
H	4.545606	-4.495317	0.620414
C	4.414991	-1.191335	1.217408
H	5.131610	-1.625879	1.934378
H	4.980701	-0.864755	0.330520
H	3.957796	-0.303506	1.677136
C	2.038753	-3.038255	2.149739
H	1.765980	-2.217063	2.827109
H	1.113847	-3.488623	1.757111
H	2.573970	-3.803741	2.736308
Al	2.468885	2.282632	0.152495
Cl	3.903502	3.270167	-1.117093
Cl	3.165504	2.272561	2.209574
Cl	0.547885	3.292102	0.110432

65

26b-Al_10 Eopt -3290.923340

C	-1.162983	-0.067405	-0.013768
C	-0.332494	-0.678055	1.106693
C	0.940676	-0.343860	1.342787
C	1.532918	0.775224	0.485715
C	1.017499	0.635942	-0.930863
C	-0.242873	0.287225	-1.189925
C	1.792463	-1.079594	2.351156
C	3.068619	0.742612	0.336863
C	3.664058	-0.656867	0.637773
C	3.306428	-1.029823	2.075016
C	5.163624	-0.475274	0.487381

C	4.450964	0.185742	-1.647333
C	3.384266	1.148363	-1.115875
H	1.604374	-0.669611	3.356998
H	-0.828861	-1.470899	1.674431
H	3.777650	-0.273294	2.720217
H	4.911969	0.526234	-2.581219
O	-2.124478	-0.926453	-0.455216
C	-0.778383	0.061560	-2.568892
H	-0.117229	0.486611	-3.334934
H	-0.877927	-1.018081	-2.757702
H	-1.783707	0.485691	-2.692492
C	2.138863	0.761698	-1.930843
H	1.911398	1.456700	-2.751758
H	1.444581	-2.122310	2.383092
H	3.770681	-1.986138	2.351621
O	6.004227	-0.676025	1.322809
O	5.515997	-0.036090	-0.732132
C	3.249594	-1.678429	-0.470488
H	2.158260	-1.812713	-0.428906
C	3.568161	-1.052385	-1.858527
H	4.016958	-1.795761	-2.530882
H	3.652721	2.206594	-1.211679
H	3.533110	1.437953	1.046201
C	3.904414	-3.045131	-0.295658
H	5.002927	-2.990445	-0.369224
H	3.651987	-3.494404	0.675480
H	3.554588	-3.731963	-1.080759
O	2.443657	-0.513141	-2.515245
O	1.081598	2.060238	0.940202
C	1.563433	2.509847	2.183661
H	2.627285	2.796978	2.148787
H	0.989296	3.410089	2.444458
H	1.432477	1.760644	2.984913
C	-1.961595	1.238716	0.531665
H	-2.988632	0.880356	0.753368
N	-1.275850	1.960705	2.814131
N	-1.537067	1.641227	1.792235
Si	-2.337194	2.852840	-0.567175
C	-0.847402	3.451622	-1.509923
H	-1.051109	4.485723	-1.836278
H	0.043048	3.453959	-0.863344
H	-0.634966	2.847924	-2.403066
C	-3.784269	2.328450	-1.620397
H	-4.632612	2.019645	-0.990099
H	-4.107883	3.185760	-2.233945
H	-3.547481	1.498005	-2.300826
C	-2.860418	4.124020	0.700981
H	-3.661096	3.752458	1.360815
H	-2.017178	4.465040	1.323611
H	-3.251976	5.007445	0.169659
Al	-3.507568	-1.876739	0.005309
Cl	-2.988449	-3.391116	1.459762
Cl	-4.988286	-0.535165	0.895529
Cl	-4.293531	-2.761206	-1.790951

65

26b-Al_11 Eopt -3290.931363

C	-1.448390	-0.199984	0.301525
C	-0.837561	-0.125272	-1.085307
C	0.383561	-0.565139	-1.384204
C	1.144559	-1.399231	-0.388091
C	0.773736	-1.066157	1.035776
C	-0.432156	-0.601954	1.383599
C	1.167398	-0.260919	-2.620450
C	2.663132	-1.153747	-0.452645
C	3.015811	0.301194	-0.892168
C	2.356638	0.649194	-2.245824

C	4.532558	0.277014	-0.966295
C	4.217146	-0.316781	1.295239
C	3.195380	-1.408731	0.969365
H	1.536138	-1.206467	-3.050221
H	-1.417399	0.441474	-1.822628
H	3.116556	0.608333	-3.038156
H	4.852028	-0.560318	2.154406
O	-1.972199	1.029938	0.641793
C	-0.804168	-0.253072	2.793284
H	-0.083718	-0.662400	3.512189
H	-0.845757	0.837857	2.923434
H	-1.802055	-0.641803	3.052860
C	2.012471	-1.084935	1.914671
H	1.931986	-1.771717	2.770369
H	0.546854	0.231879	-3.381540
H	1.991394	1.684178	-2.207844
O	5.225893	0.501447	-1.921847
O	5.098765	-0.044237	0.211828
C	2.696247	1.344732	0.238936
H	1.603721	1.433161	0.324722
C	3.246803	0.831870	1.592959
H	3.698161	1.650028	2.169141
H	3.590498	-2.421361	1.101423
H	3.108504	-1.837085	-1.190094
C	3.270781	2.726842	-0.068589
H	4.373064	2.725592	-0.042549
H	2.955577	3.100919	-1.052042
H	2.923546	3.448058	0.685454
O	2.287854	0.219568	2.426649
O	0.809476	-2.741667	-0.739221
C	1.477621	-3.798781	-0.101809
H	2.532997	-3.881508	-0.414269
H	1.439450	-3.725766	0.999692
H	0.961631	-4.722020	-0.400709
C	-2.690935	-1.189040	0.361347
H	-3.111977	-1.123189	1.381425
N	-4.443690	-0.160645	-1.104301
N	-3.692910	-0.600611	-0.430771
Si	-2.689022	-3.130801	-0.045729
C	-1.702554	-3.893756	1.335932
H	-1.595479	-4.976277	1.157346
H	-0.701525	-3.454257	1.422873
H	-2.229018	-3.759102	2.294227
C	-4.498559	-3.586498	0.112277
H	-4.910213	-3.310227	1.096192
H	-5.123241	-3.127092	-0.671125
H	-4.597102	-4.679919	0.005617
C	-2.101206	-3.403961	-1.788085
H	-1.031213	-3.176909	-1.885874
H	-2.269820	-4.459530	-2.060196
H	-2.674245	-2.781927	-2.495099
Al	-2.053454	2.687213	0.095137
Cl	-2.879127	3.803007	1.746371
Cl	-3.384568	2.786045	-1.623639
Cl	-0.142194	3.510119	-0.486988

65

26b-Al_12 Eopt -3290.930103

C	1.325857	-0.319985	0.331764
C	0.428868	-0.099076	1.541715
C	-0.825244	-0.548287	1.629829
C	-1.376876	-1.427023	0.530420
C	-0.736229	-1.126810	-0.802384
C	0.499559	-0.629907	-0.926857
C	-1.842309	-0.104596	2.639924
C	-2.872038	-1.171074	0.287244
C	-3.299437	0.309421	0.531229

C	-2.885449	0.791920	1.940082
C	-4.804506	0.249287	0.338531
C	-4.079548	-0.527583	-1.772161
C	-3.111277	-1.555898	-1.179779
H	-2.344667	-0.979025	3.081001
H	0.859806	0.539024	2.319913
H	-3.780478	0.876004	2.571928
H	-4.549855	-0.856305	-2.705554
O	2.148269	0.754100	0.126224
C	1.053133	-0.151845	-2.237373
H	0.769314	0.902128	-2.383896
H	2.148260	-0.188786	-2.270070
H	0.646039	-0.723020	-3.082578
C	-1.776247	-1.251177	-1.902393
H	-1.503217	-2.000212	-2.660076
H	-1.369114	0.452072	3.460825
H	-2.458599	1.800728	1.857726
O	-5.661270	0.533874	1.131975
O	-5.144746	-0.192711	-0.886874
C	-2.795814	1.262634	-0.608078
H	-1.705392	1.359746	-0.512738
C	-3.093544	0.628178	-1.988144
H	-3.451672	1.381276	-2.702211
H	-3.437162	-2.595244	-1.294902
H	-3.469121	-1.791729	0.968563
C	-3.409400	2.658627	-0.517880
H	-4.495107	2.643676	-0.709054
H	-3.245737	3.123769	0.464131
H	-2.950919	3.313002	-1.273665
O	-1.985466	-0.012183	-2.578172
O	-1.164890	-2.823485	0.762414
C	-1.597983	-3.366657	1.985520
H	-1.027628	-2.970643	2.845081

H	-2.672876	-3.201736	2.172862
H	-1.429847	-4.450850	1.927697
C	2.382929	-1.469362	0.683088
H	2.892548	-1.147794	1.608914
N	1.296831	-3.654243	1.199881
N	1.772163	-2.677371	1.019327
Si	3.881004	-1.964129	-0.541037
C	3.183078	-2.943099	-1.969194
H	2.527053	-3.756343	-1.618684
H	2.624324	-2.334445	-2.692293
H	4.025748	-3.412593	-2.504248
C	4.805959	-0.416407	-0.982746
H	5.192992	0.071139	-0.075015
H	5.666681	-0.697563	-1.612916
H	4.192719	0.317148	-1.521188
C	4.904975	-3.087904	0.549295
H	5.793726	-3.421135	-0.012068
H	5.256599	-2.566560	1.453747
H	4.354662	-3.991989	0.857265
Al	2.105254	2.495433	0.260589
Cl	0.252937	3.284215	-0.539101
Cl	2.308297	3.079371	2.339238
Cl	3.776983	3.247456	-0.880169

65

26b-Al_13 Eopt -3290.928384

C	-1.144203	0.222476	0.191785
C	-0.337793	0.470669	1.451626
C	0.937445	0.851813	1.460349
C	1.605434	1.281370	0.180979
C	0.984616	0.635964	-1.032564
C	-0.295256	0.250264	-1.086742
C	1.868961	0.817650	2.629380
C	3.085550	0.851639	0.124060

C	3.348342	-0.458886	0.929330
C	2.913080	-0.296174	2.402636
C	4.848195	-0.664094	0.795669
C	4.228857	-0.667791	-1.476498
C	3.407438	0.619420	-1.364427
H	2.372584	1.794326	2.711400
H	-0.835654	0.166143	2.378264
H	3.802370	-0.099686	3.016879
H	4.731624	-0.776884	-2.443894
O	-1.691525	-1.029527	0.328563
C	-0.897611	-0.393164	-2.300741
H	-0.807132	-1.488250	-2.245360
H	-1.968851	-0.161850	-2.402768
H	-0.390018	-0.062472	-3.216491
C	2.060377	0.235462	-2.024588
H	1.920649	0.662941	-3.028515
H	1.330853	0.641136	3.570899
H	2.490178	-1.244857	2.759534
O	5.659579	-0.731498	1.679729
O	5.243175	-0.769466	-0.485126
C	2.709119	-1.703584	0.209833
H	1.615362	-1.611847	0.275542
C	3.097514	-1.685313	-1.293947
H	3.354268	-2.693307	-1.644849
H	3.889020	1.482635	-1.835896
H	3.710587	1.641923	0.564465
C	3.121995	-3.030314	0.845329
H	4.199303	-3.227606	0.720255
H	2.895045	-3.068260	1.919666
H	2.577933	-3.855648	0.362592
O	2.100576	-1.187431	-2.157664
O	1.462991	2.700639	0.172074
C	2.086784	3.434604	-0.849092
H	1.837535	3.058961	-1.857504
H	1.714960	4.465699	-0.768382
H	3.185336	3.457437	-0.742590
C	-2.342038	1.258128	0.049048
H	-2.955631	0.953719	-0.820097
N	-3.830472	0.848757	2.018354
N	-3.199299	1.019827	1.132754
Si	-2.158614	3.225662	-0.146369
C	-3.940439	3.795141	-0.160214
H	-4.529574	3.290250	-0.942382
H	-4.437151	3.636765	0.810992
H	-3.967786	4.878179	-0.366816
C	-1.255694	3.915173	1.324456
H	-1.722028	3.582143	2.265883
H	-0.198710	3.615879	1.313807
H	-1.315497	5.016150	1.293884
C	-1.365537	3.454892	-1.813697
H	-0.393488	2.951001	-1.880365
H	-2.020818	3.061757	-2.607112
H	-1.215047	4.531241	-1.999060
Al	-2.922020	-2.206265	-0.015853
Cl	-2.099481	-3.881273	-1.090341
Cl	-3.750204	-2.834049	1.878945
Cl	-4.503501	-1.236990	-1.166069

65

26b-Al_14 Eopt -3290.932680

C	1.569785	0.026712	-0.167661
C	0.951452	-0.167757	1.200366
C	-0.173709	-0.843097	1.420283
C	-0.783494	-1.673092	0.323444
C	-0.438353	-1.152692	-1.050110
C	0.679545	-0.466587	-1.316877
C	-1.008277	-0.793988	2.659175

C	-2.324464	-1.642775	0.362189
C	-2.867969	-0.293299	0.928978
C	-2.302554	-0.016627	2.340015
C	-4.373529	-0.495854	0.942406
C	-3.933817	-0.826986	-1.347842
C	-2.793540	-1.817703	-1.094021
H	-1.249610	-1.822002	2.974107
H	1.407115	0.420461	2.002785
H	-3.072008	-0.252903	3.087517
H	-4.512687	-1.056346	-2.249432
O	1.831439	1.369864	-0.349614
C	1.008017	0.061221	-2.682062
H	0.733535	1.123160	-2.757058
H	2.084925	-0.012727	-2.900257
H	0.466501	-0.488706	-3.462536
C	-1.642760	-1.260983	-1.968925
H	-1.453173	-1.850741	-2.878123
H	-0.475413	-0.306671	3.487285
H	-2.084332	1.055195	2.433900
O	-5.112276	-0.442592	1.889072
O	-4.868502	-0.768153	-0.277944
C	-2.631525	0.884154	-0.086949
H	-1.551329	1.078651	-0.139568
C	-3.103402	0.451865	-1.501625
H	-3.640191	1.268055	-2.002106
H	-3.058792	-2.852363	-1.335098
H	-2.688629	-2.449498	1.014666
C	-3.330082	2.175720	0.332053
H	-4.427682	2.081022	0.289831
H	-3.053228	2.488399	1.347642
H	-3.037514	2.986241	-0.350999
O	-2.075301	0.037761	-2.373053
O	-0.267618	-2.983583	0.554871

C	-0.739451	-4.042262	-0.236898
H	-1.786567	-4.307005	-0.008428
H	-0.654804	-3.835227	-1.318869
H	-0.110198	-4.913303	-0.006286
C	2.994974	-0.663426	-0.265952
H	3.415586	-0.439167	-1.262956
N	4.427306	0.632475	1.326164
N	3.821631	0.071761	0.598890
Si	3.363442	-2.592918	0.010635
C	2.575474	-3.418728	-1.459380
H	2.713393	-4.509809	-1.381910
H	1.500028	-3.208383	-1.515619
H	3.050254	-3.081519	-2.394296
C	5.230783	-2.669373	-0.081468
H	5.550577	-3.723413	-0.026198
H	5.613907	-2.253017	-1.026826
H	5.712568	-2.138530	0.756035
C	2.753962	-3.122528	1.684653
H	1.655644	-3.139716	1.712458
H	3.132263	-4.137652	1.892574
H	3.130524	-2.456064	2.477327
Al	1.177173	2.966904	-0.070828
Cl	2.905008	4.269126	-0.038352
Cl	-0.158592	3.554784	-1.673072
Cl	0.111879	3.142981	1.810690

65

26b-Al_15 Eopt -3290.926368

C	-1.174389	0.200147	-0.151005
C	-0.516504	0.210147	1.218484
C	0.749546	0.569938	1.437109
C	1.536258	1.233145	0.335299
C	1.058928	0.832705	-1.035251
C	-0.192145	0.453123	-1.301006

C	1.570381	0.174539	2.629235
C	3.012112	0.810119	0.347882
C	3.212168	-0.668331	0.804377
C	2.568450	-0.917573	2.188066
C	4.724700	-0.807124	0.825550
C	4.392438	-0.209972	-1.433747
C	3.495259	0.986680	-1.099446
H	2.122447	1.042362	3.020783
H	-1.088663	-0.303910	1.998564
H	3.362040	-1.017712	2.941381
H	5.018919	-0.052147	-2.318642
O	-1.700600	-1.048617	-0.346949
C	-0.662996	0.053086	-2.667386
H	-0.737212	-1.041747	-2.741356
H	-1.664366	0.457634	-2.884754
H	0.022397	0.406454	-3.447905
C	2.243362	0.749619	-1.979891
H	2.167151	1.435645	-2.835756
H	0.937700	-0.209749	3.441552
H	2.030939	-1.875418	2.164550
O	5.423765	-1.092972	1.760527
O	5.279076	-0.555334	-0.374285
C	2.736721	-1.687862	-0.292567
H	1.639710	-1.639198	-0.347886
C	3.302561	-1.263139	-1.674013
H	3.654357	-2.135179	-2.240658
H	3.967317	1.959922	-1.271901
H	3.581416	1.440190	1.043907
C	3.143120	-3.124907	0.031047
H	2.698262	-3.810892	-0.705000
H	4.236260	-3.260960	-0.009900
H	2.801543	-3.440171	1.026852
O	2.387422	-0.574645	-2.494919

O	1.425500	2.658034	0.353278
C	1.841102	3.356568	1.498958
H	2.911111	3.210476	1.727114
H	1.681591	4.423230	1.289284
H	1.253432	3.090475	2.394428
C	-2.362117	1.255738	-0.267681
H	-2.940107	1.009889	-1.177669
N	-3.916154	0.832256	1.644695
N	-3.260934	0.996715	0.774474
Si	-2.134384	3.236990	-0.300102
C	-3.865912	3.814238	-0.705183
H	-4.185644	3.470190	-1.701695
H	-4.603911	3.468205	0.036870
H	-3.891478	4.916778	-0.705405
C	-1.652559	3.737356	1.427960
H	-2.495354	3.599858	2.124692
H	-0.798936	3.150335	1.791000
H	-1.373175	4.803396	1.446334
C	-0.934695	3.669801	-1.650530
H	-1.033964	4.750855	-1.848284
H	0.096831	3.456927	-1.340534
H	-1.159501	3.134411	-2.585847
Al	-2.884081	-2.268241	-0.001240
Cl	-3.089303	-2.513177	2.144013
Cl	-4.781188	-1.551895	-0.785560
Cl	-2.274984	-4.095730	-0.952542

65

26b-Al_16 Eopt -3290.931833

C	-1.578457	0.061291	0.163596
C	-0.966184	-0.198429	-1.200407
C	0.161906	-0.882274	-1.398074
C	0.757553	-1.672136	-0.262630
C	0.417804	-1.092422	1.084843

C	-0.685716	-0.380895	1.329384
C	1.043358	-0.774708	-2.607025
C	2.291064	-1.661851	-0.293877
C	2.869735	-0.312266	-0.820190
C	2.297466	0.036208	-2.213793
C	4.364707	-0.578664	-0.846686
C	3.913369	-0.964304	1.437886
C	2.726077	-1.893354	1.160508
H	1.342676	-1.773942	-2.958754
H	-1.405206	0.387637	-2.013984
H	3.080777	-0.105632	-2.971074
H	4.486835	-1.242303	2.329155
O	-1.836432	1.413909	0.268320
C	-1.010058	0.190062	2.678609
H	-0.468765	-0.337685	3.474223
H	-0.727368	1.251866	2.720309
H	-2.085691	0.132426	2.906890
C	1.592276	-1.289227	2.027174
H	1.341644	-1.892265	2.911794
H	0.525813	-0.271967	-3.436033
H	2.022101	1.098786	-2.229629
O	5.104206	-0.528832	-1.793014
O	4.846731	-0.916006	0.363540
C	2.691149	0.843579	0.228001
H	1.621323	1.085731	0.293095
C	3.145104	0.350458	1.627522
H	3.721856	1.124467	2.150237
H	2.923437	-2.948144	1.380464
H	2.663377	-2.451757	-0.959447
C	3.443525	2.113302	-0.163985
H	4.536118	1.968244	-0.139844
H	3.169324	2.464370	-1.168295
H	3.196253	2.917907	0.543415
O	2.093515	-0.034221	2.483583
O	0.265033	-3.013206	-0.207626
C	0.485306	-3.868870	-1.299801
H	0.050131	-3.483973	-2.237736
H	1.555243	-4.080093	-1.470328
H	-0.013396	-4.817463	-1.056308
C	-3.002388	-0.617113	0.330460
H	-3.402761	-0.316894	1.315121
N	-4.468519	0.557987	-1.323757
N	-3.846620	0.053993	-0.569111
Si	-3.378882	-2.561401	0.198583
C	-2.402501	-3.375395	1.553701
H	-2.554706	-2.866532	2.518564
H	-2.761259	-4.413282	1.659580
H	-1.331637	-3.393131	1.309582
C	-5.217004	-2.643981	0.535679
H	-5.467245	-2.237252	1.528553
H	-5.809835	-2.106385	-0.222161
H	-5.539624	-3.698456	0.515715
C	-2.997688	-3.090933	-1.544318
H	-1.962431	-2.853666	-1.819149
H	-3.146781	-4.178349	-1.646680
H	-3.672077	-2.590550	-2.258268
Al	-1.136521	2.986810	-0.038160
Cl	-2.818704	4.346665	-0.042015
Cl	0.258017	3.554828	1.520558
Cl	-0.111659	3.111090	-1.946832

65

26b-Al_17 Eopt -3290.930866

C	-1.446572	-0.174795	0.314404
C	-0.843599	-0.146295	-1.081189
C	0.378027	-0.594852	-1.377465
C	1.130096	-1.412169	-0.359535

C	0.760973	-1.048135	1.054145
C	-0.430644	-0.555526	1.400150
C	1.189664	-0.201840	-2.576420
C	2.643008	-1.171157	-0.419863
C	3.011010	0.298302	-0.790924
C	2.328628	0.731767	-2.109462
C	4.524773	0.237063	-0.890920
C	4.222823	-0.464991	1.345649
C	3.159229	-1.507658	0.986383
H	1.618401	-1.093331	-3.059475
H	-1.409051	0.437145	-1.816847
H	3.086308	0.809568	-2.901421
H	4.864965	-0.764818	2.181228
O	-1.956221	1.071368	0.611005
C	-0.790390	-0.176894	2.806082
H	-0.082143	-0.597681	3.530284
H	-0.792571	0.917205	2.919158
H	-1.800091	-0.523948	3.077617
C	1.987458	-1.169404	1.940971
H	1.858378	-1.896007	2.756096
H	0.572975	0.316869	-3.323825
H	1.901025	1.733976	-1.972699
O	5.211997	0.490472	-1.843715
O	5.095935	-0.166197	0.260065
C	2.740100	1.295358	0.391924
H	1.651858	1.408859	0.499117
C	3.295895	0.702751	1.710676
H	3.784579	1.477059	2.316352
H	3.502710	-2.544115	1.073887
H	3.097823	-1.816443	-1.183180
C	3.345466	2.674237	0.133213
H	3.028112	3.373001	0.921113
H	4.447539	2.644667	0.141879

H	3.025495	3.094683	-0.829935
O	2.326169	0.086715	2.527454
O	0.844275	-2.809116	-0.462566
C	1.152721	-3.481623	-1.656710
H	0.611668	-3.072815	-2.527034
H	2.232873	-3.479998	-1.882817
H	0.837186	-4.524759	-1.516402
C	-2.694781	-1.150076	0.434972
H	-3.120681	-1.006041	1.444368
N	-4.424837	-0.215543	-1.116601
N	-3.686421	-0.614668	-0.404812
Si	-2.698565	-3.115776	0.169913
C	-4.466524	-3.575682	0.577553
H	-5.197059	-3.095708	-0.093890
H	-4.586914	-4.666270	0.464838
H	-4.724773	-3.317183	1.616909
C	-2.333303	-3.429169	-1.628815
H	-1.427321	-2.901179	-1.952437
H	-2.191140	-4.507542	-1.806571
H	-3.174849	-3.094458	-2.256972
C	-1.522241	-3.832465	1.417751
H	-1.688732	-4.922305	1.458103
H	-0.479417	-3.641961	1.131927
H	-1.711469	-3.426093	2.423893
Al	-2.025187	2.710802	0.015085
Cl	-0.095923	3.502892	-0.558200
Cl	-2.864784	3.884961	1.617460
Cl	-3.337981	2.753624	-1.719942

65

26b-Al_2 Eopt -3290.927319

C	1.148418	0.224666	-0.201398
C	0.337764	0.532328	-1.449729
C	-0.943601	0.902460	-1.442529

C	-1.597732	1.279857	-0.138850
C	-0.969139	0.589589	1.041631
C	0.302597	0.186143	1.076196
C	-1.894637	0.774872	-2.595886
C	-3.067684	0.840285	-0.082835
C	-3.324181	-0.498574	-0.842103
C	-2.845767	-0.407620	-2.310046
C	-4.829498	-0.666630	-0.723893
C	-4.238284	-0.592228	1.560004
C	-3.381711	0.669786	1.411326
H	-2.481144	1.698751	-2.715183
H	0.824862	0.232262	-2.383693
H	-3.721237	-0.343044	-2.970735
H	-4.758692	-0.655950	2.522070
O	1.704051	-1.011934	-0.418190
C	0.907317	-0.497333	2.267023
H	1.975951	-0.262278	2.383761
H	0.393574	-0.209723	3.193682
H	0.827775	-1.590581	2.168239
C	-2.038087	0.266406	2.068120
H	-1.861265	0.739523	3.044881
H	-1.359194	0.598625	-3.539357
H	-2.319270	-1.335357	-2.572494
O	-5.631928	-0.751769	-1.614631
O	-5.241618	-0.707401	0.556095
C	-2.728196	-1.730557	-0.069120
H	-1.631847	-1.670641	-0.126682
C	-3.131435	-1.646629	1.427413
H	-3.418795	-2.633203	1.813728
H	-3.827573	1.566610	1.854870
H	-3.709058	1.601248	-0.547175
C	-3.171507	-3.065797	-0.665352
H	-2.649790	-3.889526	-0.155900
H	-4.253850	-3.232282	-0.539209
H	-2.941533	-3.140514	-1.737385
O	-2.126455	-1.143441	2.276960
O	-1.479260	2.673271	0.155243
C	-2.018811	3.607012	-0.744644
H	-3.113359	3.514170	-0.854122
H	-1.802639	4.600969	-0.329092
H	-1.560392	3.546637	-1.746585
C	2.343542	1.250297	0.017555
H	2.974623	0.858657	0.838024
N	3.777074	1.056565	-2.023394
N	3.174509	1.130792	-1.104785
Si	2.160297	3.187319	0.413569
C	3.934084	3.688801	0.728652
H	4.588772	3.486130	-0.134505
H	3.976799	4.772712	0.927538
H	4.348092	3.169403	1.607722
C	1.487661	3.996500	-1.122250
H	2.226350	3.963192	-1.939391
H	0.569099	3.500830	-1.462073
H	1.256843	5.055411	-0.921256
C	1.128488	3.340464	1.951063
H	1.241401	4.369291	2.333169
H	0.068203	3.155493	1.732625
H	1.471307	2.649772	2.737368
Al	2.920101	-2.211132	-0.098122
Cl	4.467450	-1.328542	1.163624
Cl	3.809368	-2.735441	-1.996606
Cl	2.049272	-3.938384	0.850283

65

26b-Al_3 Eopt -3290.916015

C	1.058190	0.017972	0.040270
C	0.312115	0.254545	1.345023

C	-0.924356	-0.179049	1.590292
C	-1.593510	-1.117073	0.623611
C	-1.076406	-0.936445	-0.782986
C	0.142630	-0.479114	-1.075916
C	-1.830655	0.313462	2.676213
C	-3.098295	-0.839562	0.502981
C	-3.461543	0.662880	0.688893
C	-2.929241	1.193308	2.040317
C	-4.979294	0.656867	0.619953
C	-4.464011	-0.279498	-1.483731
C	-3.487967	-1.309812	-0.907427
H	-2.291294	-0.546949	3.188120
H	0.811660	0.949512	2.027999
H	-3.768673	1.297042	2.741437
H	-5.021084	-0.643572	-2.354123
O	1.581401	1.219301	-0.357523
C	0.639439	-0.251617	-2.472488
H	1.718018	-0.454419	-2.557471
H	0.104649	-0.876084	-3.200156
H	0.499222	0.801338	-2.758125
C	-2.215603	-1.114955	-1.768498
H	-2.042984	-1.923509	-2.493965
H	-1.279356	0.892909	3.429716
H	-2.514886	2.200120	1.893354
O	-5.754215	1.032141	1.458481
O	-5.438230	0.154292	-0.540382
C	-3.012857	1.523657	-0.546712
H	-1.914288	1.570332	-0.545787
C	-3.456932	0.816702	-1.856100
H	-3.849266	1.542498	-2.580467
H	-3.863882	-2.338494	-0.919721
H	-3.615919	-1.413223	1.283307
C	-3.560949	2.948918	-0.498068

H	-4.658045	2.969533	-0.603087
H	-3.302559	3.463150	0.438136
H	-3.140347	3.537350	-1.327001
O	-2.439109	0.089439	-2.504888
O	-1.508620	-2.500705	1.003041
C	-0.558769	-2.899126	1.950748
H	0.473871	-2.656169	1.654496
H	-0.736530	-2.458750	2.947030
H	-0.637495	-3.992293	2.034545
C	2.287123	-1.034821	0.218456
H	3.164556	-0.577522	-0.280503
N	2.892228	-1.156336	2.631242
N	2.653689	-1.084928	1.556969
Si	2.429203	-2.910480	-0.534484
C	3.747875	-2.714360	-1.838820
H	3.443248	-2.017003	-2.634316
H	4.695433	-2.355105	-1.407576
H	3.936705	-3.695285	-2.305752
C	3.049221	-3.961109	0.882001
H	4.007976	-3.599547	1.286796
H	2.326238	-4.051091	1.707867
H	3.224537	-4.976348	0.488313
C	0.851758	-3.582456	-1.257716
H	0.606915	-3.096826	-2.212957
H	1.049092	-4.649016	-1.464823
H	-0.020129	-3.511225	-0.594173
Al	2.974316	2.235474	-0.100967
Cl	2.534884	4.218854	-0.786128
Cl	3.492240	2.191556	2.007606
Cl	4.616445	1.347909	-1.222351

65

26b-Al_4 Eopt -3290.931148

C	-0.961914	-0.121512	-0.066471

C	-0.134237	-0.532221	-1.279165
C	1.108858	-0.996595	-1.160816
C	1.705618	-1.121086	0.223363
C	1.197091	-0.023450	1.136779
C	-0.035436	0.483324	1.013305
C	2.016468	-1.440475	-2.262677
C	3.238655	-0.935148	0.227298
C	3.743165	-0.035939	-0.947795
C	3.308725	-0.603252	-2.312073
C	5.254883	-0.052162	-0.784241
C	4.625590	0.831242	1.303240
C	3.587442	-0.261438	1.569262
H	2.270458	-2.495522	-2.064273
H	-0.626107	-0.422974	-2.246793
H	4.126037	-1.214533	-2.717471
H	5.128532	1.178657	2.212493
O	-1.975014	0.695597	-0.435088
C	-0.536014	1.659531	1.791167
H	-0.586870	2.545768	1.139044
H	-1.553074	1.504205	2.181126
H	0.127083	1.898890	2.631394
C	2.329029	0.545195	1.966330
H	2.118429	0.526733	3.045940
H	1.503359	-1.408860	-3.233589
H	3.168283	0.226031	-3.017351
O	6.071511	-0.428702	-1.581398
O	5.652060	0.418999	0.410255
C	3.334507	1.467034	-0.734588
H	2.241636	1.539939	-0.836239
C	3.699499	1.899046	0.709488
H	4.131917	2.908053	0.722332
H	3.899555	-0.978170	2.336543
H	3.718319	-1.919107	0.119527

C	3.980308	2.412175	-1.746898
H	5.076352	2.446255	-1.635044
H	3.755785	2.135337	-2.785818
H	3.603053	3.433462	-1.589225
O	2.613064	1.898508	1.605782
O	1.323807	-2.434257	0.653351
C	1.711234	-2.837881	1.944913
H	2.799848	-2.998164	2.023205
H	1.398326	-2.115145	2.719652
H	1.211432	-3.796261	2.141703
C	-1.547304	-1.500047	0.510393
H	-0.694679	-2.195033	0.656771
N	-2.492941	-1.128201	2.790786
N	-2.053004	-1.301360	1.794732
Si	-2.932010	-2.499418	-0.525375
C	-2.737782	-2.142184	-2.339470
H	-3.530331	-2.704451	-2.862645
H	-2.888685	-1.072971	-2.549065
H	-1.767818	-2.469192	-2.740637
C	-2.525116	-4.268504	-0.082672
H	-1.511185	-4.538208	-0.419506
H	-2.584054	-4.435170	1.005443
H	-3.239602	-4.954512	-0.566470
C	-4.627385	-2.013994	0.080267
H	-4.743809	-2.043005	1.174509
H	-4.922218	-1.018969	-0.283435
H	-5.340771	-2.743135	-0.340820
Al	-3.335657	1.753163	-0.294526
Cl	-4.332700	1.399984	1.606562
Cl	-4.704278	1.294550	-1.909919
Cl	-2.725060	3.818208	-0.456213

65

26b-Al_5 Eopt -3290.930768

C	-1.119602	-0.224298	-0.101100
C	-0.316798	0.263038	-1.296335
C	0.927571	-0.128138	-1.563507
C	1.589137	-1.180061	-0.707412
C	1.034678	-1.191595	0.697579
C	-0.213830	-0.806449	0.991765
C	1.828256	0.430292	-2.617881
C	3.096647	-0.902657	-0.518188
C	3.437475	0.618983	-0.601521
C	2.966973	1.221290	-1.942898
C	4.950181	0.652581	-0.459820
C	4.372189	-0.413189	1.558278
C	3.461183	-1.438830	0.878779
H	2.245011	-0.406624	-3.201086
H	-0.822081	0.986386	-1.943221
H	3.825268	1.289180	-2.624933
H	4.897434	-0.813681	2.432454
O	-1.868285	0.775177	0.455173
C	-0.691787	-0.657699	2.406588
H	-0.505869	0.369978	2.752019
H	-1.770308	-0.827807	2.502666
H	-0.162810	-1.341426	3.083697
C	2.157164	-1.354483	1.706440
H	2.011389	-2.204258	2.389759
H	1.279985	1.080180	-3.313518
H	2.619210	2.248959	-1.773165
O	5.748712	1.097371	-1.240042
O	5.374163	0.102150	0.691286
C	2.899570	1.390044	0.659217
H	1.800874	1.398848	0.608931
C	3.311842	0.623879	1.944520
H	3.644428	1.318540	2.726974
H	3.888692	-2.446761	0.849876

H	3.655368	-1.413260	-1.315848
C	3.388998	2.835879	0.723196
H	4.481348	2.894653	0.859308
H	3.129184	3.405245	-0.179652
H	2.922736	3.345624	1.579337
O	2.294859	-0.175600	2.503790
O	1.365408	-2.399732	-1.414737
C	1.855423	-3.587568	-0.846407
H	1.470171	-4.416086	-1.457067
H	2.957688	-3.641039	-0.862178
H	1.505486	-3.731454	0.192211
C	-2.211808	-1.259043	-0.668755
H	-2.820715	-0.679772	-1.390217
N	-1.121950	-3.078078	-1.981484
N	-1.619658	-2.261409	-1.434692
Si	-3.558923	-2.154826	0.482780
C	-4.453569	-0.834672	1.437624
H	-3.802338	-0.248798	2.098912
H	-4.947974	-0.139548	0.741674
H	-5.236112	-1.317256	2.046935
C	-4.689593	-2.952527	-0.774555
H	-5.513394	-3.462608	-0.248056
H	-5.135422	-2.200885	-1.445469
H	-4.173669	-3.708581	-1.388460
C	-2.679844	-3.464759	1.482392
H	-2.078901	-4.120405	0.830470
H	-2.022955	-3.056431	2.262572
H	-3.436523	-4.097906	1.975188
Al	-2.663496	2.247555	-0.051668
Cl	-3.985831	1.721650	-1.706637
Cl	-3.797093	2.983976	1.622629
Cl	-1.216205	3.719535	-0.695749

65

26b-Al_6 Eopt -3290.929762

C	-1.133230	-0.283952	-0.077947	H	3.837013	-2.555719	0.851698
C	-0.338812	0.124598	-1.308402	H	3.652603	-1.428029	-1.261211
C	0.911741	-0.263974	-1.558238	C	3.371941	2.739310	0.898890
C	1.574242	-1.269856	-0.648754	H	4.465564	2.788138	1.028178
C	1.012578	-1.230556	0.749281	H	3.109653	3.330414	0.010545
C	-0.231705	-0.832895	1.033753	H	2.915138	3.231851	1.770094
C	1.829527	0.371072	-2.560158	O	2.296610	-0.322693	2.602227
C	3.067668	-0.959573	-0.458129	O	1.402672	-2.620596	-1.087204
C	3.381361	0.570001	-0.493104	C	1.755802	-2.940689	-2.410115
C	2.862130	1.223597	-1.794330	H	2.805499	-2.694909	-2.646334
C	4.896173	0.601155	-0.383650	H	1.631900	-4.027201	-2.516102
C	4.358283	-0.538997	1.611023	H	1.103947	-2.442679	-3.150261
C	3.429941	-1.540962	0.916942	C	-2.251366	-1.329368	-0.570113
H	2.348594	-0.399254	-3.149842	H	-2.787248	-0.833543	-1.400849
H	-0.839427	0.835900	-1.972769	N	-1.156412	-3.365249	-1.502152
H	3.713641	1.451397	-2.450047	N	-1.659400	-2.454488	-1.139179
H	4.904373	-0.965571	2.459672	Si	-3.718246	-2.006037	0.594541
O	-1.847617	0.767028	0.424994	C	-4.499552	-0.563068	1.464240
C	-0.719946	-0.661632	2.442850	H	-3.807888	-0.014117	2.115649
H	-0.518036	0.365639	2.781320	H	-4.914056	0.144906	0.730631
H	-1.802462	-0.809673	2.532474	H	-5.335117	-0.946342	2.074020
H	-0.210057	-1.347999	3.131888	C	-4.887483	-2.749954	-0.660441
C	2.129101	-1.458170	1.752337	H	-5.752639	-3.191180	-0.138232
H	1.959428	-2.333585	2.395752	H	-5.268635	-1.983139	-1.353635
H	1.272871	1.007102	-3.262477	H	-4.414344	-3.551220	-1.251133
H	2.385721	2.181454	-1.546270	C	-3.012118	-3.342732	1.690570
O	5.679988	1.070406	-1.164568	H	-2.567093	-4.154824	1.092700
O	5.341674	0.006963	0.738297	H	-2.254603	-2.978546	2.398159
C	2.873820	1.297971	0.803213	H	-3.836024	-3.783637	2.276524
H	1.774452	1.312698	0.773350	Al	-2.504682	2.280507	-0.156481
C	3.308550	0.489187	2.053378	Cl	-3.749919	1.807154	-1.882365
H	3.659502	1.155433	2.852395	Cl	-3.683685	3.133474	1.428572
				Cl	-0.920735	3.641748	-0.725292

65

26b-Al_7 Eopt -3290.931326

C	1.330998	-0.260285	0.342954
C	0.427107	0.044798	1.527917
C	-0.815337	-0.424414	1.643186
C	-1.363269	-1.366814	0.596105
C	-0.734351	-1.120800	-0.755870
C	0.506533	-0.637140	-0.899212
C	-1.809700	-0.072910	2.703096
C	-2.874035	-1.155789	0.355797
C	-3.333423	0.307744	0.645489
C	-2.971459	0.730769	2.084751
C	-4.835212	0.255424	0.421125
C	-4.076445	-0.438603	-1.698430
C	-3.123317	-1.490918	-1.126644
H	-2.193286	-1.010531	3.137678
H	0.863743	0.697787	2.290201
H	-3.862518	0.637805	2.720327
H	-4.525963	-0.735404	-2.652584
O	2.172493	0.779490	0.056452
C	1.047875	-0.200406	-2.229014
H	2.137484	-0.300862	-2.294140
H	0.586596	-0.758140	-3.054714
H	0.816981	0.865594	-2.380057
C	-1.788044	-1.187140	-1.846557
H	-1.541591	-1.908027	-2.640433
H	-1.344611	0.502531	3.515162
H	-2.695887	1.793695	2.086550
O	-5.703968	0.512380	1.210937
O	-5.157224	-0.134675	-0.825133
C	-2.800124	1.299894	-0.449571
H	-1.709967	1.384501	-0.335085
C	-3.082420	0.716818	-1.857809
H	-3.426633	1.499120	-2.546780
H	-3.471947	-2.517820	-1.279578
H	-3.435665	-1.818816	1.029846
C	-3.401517	2.698587	-0.325979
H	-2.934492	3.366090	-1.064770
H	-4.486867	2.698779	-0.519853
H	-3.235112	3.139407	0.666482
O	-1.975735	0.087590	-2.463452
O	-1.101694	-2.670030	1.118227
C	-1.487666	-3.777563	0.344440
H	-1.091562	-3.728073	-0.686209
H	-1.068569	-4.669414	0.831343
H	-2.583136	-3.901750	0.301732
C	2.377714	-1.385732	0.801758
H	2.975478	-0.916140	1.603299
N	1.288774	-3.360682	1.871343
N	1.771616	-2.476228	1.427718
Si	3.728589	-2.134457	-0.448706
C	4.721293	-0.702157	-1.095159
H	4.111788	0.059070	-1.598611
H	5.249836	-0.208014	-0.264882
H	5.477212	-1.083904	-1.801504
C	4.760180	-3.228537	0.664279
H	5.581118	-3.667464	0.072980
H	5.212383	-2.656820	1.490229
H	4.182126	-4.064296	1.091078
C	2.831542	-3.180573	-1.708577
H	2.236882	-2.597930	-2.424728
H	3.578564	-3.756757	-2.279873
H	2.166227	-3.907119	-1.212985
Al	2.190867	2.523616	0.134867
Cl	2.541961	3.161237	2.177206
Cl	3.816177	3.166473	-1.132564

Cl	0.313311	3.340119	-0.572268

65

26b-Al_8 Eopt -3290.923338

C	-1.165592	-0.067322	-0.016185
C	-0.336294	-0.681050	1.103663
C	0.937501	-0.349754	1.340401
C	1.530494	0.771303	0.486435
C	1.016127	0.634825	-0.930910
C	-0.244420	0.287786	-1.191339
C	1.788772	-1.091151	2.345314
C	3.066286	0.740212	0.338517
C	3.662606	-0.659108	0.638123
C	3.303135	-1.033844	2.074378
C	5.162046	-0.475986	0.489390
C	4.450604	0.187013	-1.645227
C	3.382796	1.148191	-1.113388
H	1.596002	-0.691262	3.354312
H	-0.833631	-1.474901	1.669123
H	3.768684	-0.274904	2.720950
H	4.912163	0.529028	-2.578282
O	-2.128106	-0.924519	-0.459065
C	-0.780161	0.066427	-2.570824
H	-0.116176	0.488527	-3.336022
H	-0.886113	-1.012381	-2.760768
H	-1.782882	0.496819	-2.694464
C	2.138312	0.761902	-1.929786
H	1.911304	1.457658	-2.750192
H	1.444637	-2.135411	2.366525
H	3.771042	-1.987951	2.352483
O	6.002052	-0.676890	1.325374
O	5.515066	-0.035193	-0.729389
C	3.249998	-1.679641	-0.471690
H	2.158766	-1.815164	-0.431170
C	3.568806	-1.051472	-1.858671
H	4.018497	-1.793585	-2.531826
H	3.650638	2.206731	-1.207494
H	3.529706	1.435106	1.049005
C	3.906238	-3.045812	-0.298018
H	5.004693	-2.989852	-0.371458
H	3.654265	-3.496169	0.672744
H	3.557236	-3.732342	-1.083751
O	2.444141	-0.512254	-2.515174
O	1.078306	2.054987	0.943949
C	1.556322	2.499383	2.190751
H	2.621195	2.783200	2.161351
H	0.983758	3.400530	2.451867
H	1.419657	1.748029	2.989052
C	-1.962177	1.240201	0.529685
H	-2.991932	0.885365	0.744093
N	-1.283519	1.949498	2.818239
N	-1.542303	1.635416	1.794045
Si	-2.324694	2.861483	-0.563876
C	-3.773081	2.349295	-1.621437
H	-3.543230	1.512110	-2.295996
H	-4.628127	2.054374	-0.993546
H	-4.083462	3.206935	-2.241292
C	-2.843347	4.131529	0.707292
H	-1.999666	4.467289	1.332113
H	-3.230285	5.017978	0.177596
H	-3.646723	3.761920	1.364954
C	-0.830418	3.455120	-1.502702
H	-0.620993	2.853704	-2.398114
H	-1.028007	4.491667	-1.825047
H	0.059295	3.449568	-0.855157
Al	-3.510222	-1.876034	0.002366
Cl	-4.294402	-2.762571	-1.793818

Cl	-2.990735	-3.389675	1.457624
Cl	-4.991918	-0.535205	0.891355

65

26b-Al_9 Eopt -3290.929771

C	-1.128975	-0.289254	-0.083231
C	-0.334736	0.113401	-1.315471
C	0.917316	-0.273107	-1.561267
C	1.580731	-1.273352	-0.646108
C	1.017094	-1.228991	0.750957
C	-0.228037	-0.831714	1.031871
C	1.835504	0.360083	-2.564072
C	3.073258	-0.959772	-0.454758
C	3.384379	0.570078	-0.495489
C	2.865540	1.217467	-1.799978
C	4.898977	0.604208	-0.384163
C	4.360314	-0.528850	1.614433
C	3.434535	-1.535092	0.923177
H	2.356950	-0.411179	-3.150400
H	-0.835362	0.821569	-1.983056
H	3.717501	1.444276	-2.455467
H	4.906017	-0.951082	2.465505
O	-1.843161	0.764087	0.413597
C	-0.719847	-0.654963	2.438832
H	-1.804406	-0.790419	2.522492
H	-0.220444	-1.346747	3.130270
H	-0.507933	0.370021	2.778094
C	2.132370	-1.450972	1.756497
H	1.963014	-2.324024	2.403221
H	1.278677	0.992655	-3.269379
H	2.387109	2.175450	-1.556282
O	5.683068	1.071728	-1.165834
O	5.343948	0.015188	0.740741
C	2.874130	1.302536	0.797269
H	1.774775	1.315796	0.765838
C	3.308314	0.499414	2.051286
H	3.657128	1.169370	2.848134
H	3.843273	-2.549461	0.862578
H	3.659792	-1.430624	-1.255328
C	3.370514	2.744804	0.887845
H	4.463883	2.795304	1.018631
H	3.108986	3.331987	-0.003326
H	2.911914	3.240355	1.756395
O	2.296879	-0.311841	2.602018
O	1.413463	-2.626650	-1.078297
C	1.763318	-2.950663	-2.401042
H	1.645026	-4.038299	-2.501856
H	1.105772	-2.459471	-3.140721
H	2.810736	-2.700012	-2.642284
C	-2.244044	-1.340543	-0.569318
H	-2.771039	-0.858756	-1.413736
N	-1.139860	-3.390825	-1.458194
N	-1.646373	-2.474662	-1.114229
Si	-3.727064	-1.996730	0.587707
C	-3.045322	-3.313440	1.722343
H	-2.331936	-2.930111	2.464880
H	-3.888492	-3.767686	2.269511
H	-2.555742	-4.120264	1.152829
C	-4.520405	-0.537077	1.417464
H	-4.914214	0.160023	0.662260
H	-5.371489	-0.906593	2.014109
H	-3.840308	0.017876	2.076136
C	-4.876169	-2.763323	-0.672466
H	-5.749741	-3.194812	-0.156200
H	-5.246044	-2.009265	-1.385460
H	-4.394148	-3.575275	-1.240950
Al	-2.508321	2.276528	-0.159675

Cl	-3.615235	3.157042	1.462470
Cl	-3.819804	1.796717	-1.833209
Cl	-0.937589	3.620466	-0.802618

65

26b-Al'_6 Eopt -3290.921716

C	1.254016	0.392318	-0.187849
C	0.554757	0.110674	1.140523
C	-0.545379	-0.638699	1.188886
C	-1.065637	-1.217201	-0.105172
C	-0.854103	-0.254611	-1.254226
C	0.230636	0.522179	-1.329499
C	-1.304958	-1.070705	2.399859
C	-2.587555	-1.477615	-0.092874
C	-3.345356	-0.534411	0.890089
C	-2.796012	-0.675770	2.322398
C	-4.794338	-0.975962	0.790472
C	-4.379047	-0.422537	-1.460668
C	-3.077901	-1.225686	-1.532689
H	-1.219015	-2.169421	2.446745
H	1.028916	0.468864	2.059477
H	-3.397754	-1.424848	2.854382
H	-4.939609	-0.420735	-2.402124
O	2.167363	-0.571324	-0.549298
C	0.528024	1.395759	-2.510902
H	-0.305479	1.403005	-3.223544
H	0.715443	2.446289	-2.238086
H	1.418835	1.024302	-3.044524
C	-2.078071	-0.215119	-2.143635
H	-1.855797	-0.405187	-3.203628
H	-0.849383	-0.675328	3.318286
H	-2.946117	0.269992	2.859407
O	-5.502754	-1.366719	1.679424
O	-5.275844	-0.892924	-0.462527
C	-3.334128	0.938566	0.349698
H	-2.293680	1.283376	0.370080
C	-3.787057	0.952107	-1.134270
H	-4.476993	1.783461	-1.330264
H	-3.167177	-2.147325	-2.118050
H	-2.765222	-2.513996	0.228626
C	-4.177003	1.904947	1.177502
H	-5.248713	1.648712	1.144732
H	-3.870192	1.934259	2.232013
H	-4.069773	2.923691	0.774972
O	-2.726432	1.060272	-2.057453
O	-0.334480	-2.429118	-0.246041
C	-0.414077	-3.110088	-1.467845
H	-0.158311	-2.463253	-2.326122
H	0.323743	-3.922584	-1.426795
H	-1.409610	-3.558269	-1.642280
C	2.078304	1.726794	0.079305
H	2.725741	1.511095	0.949686
N	3.774299	1.990201	-1.739656
N	3.024115	1.877213	-0.943670
Si	1.224363	3.475928	0.520214
C	1.781305	3.789835	2.271489
H	1.361080	4.743930	2.629819
H	1.434801	2.993484	2.949268
H	2.878768	3.854130	2.341361
C	1.927680	4.727287	-0.674339
H	3.025723	4.792941	-0.606290
H	1.652184	4.521751	-1.720800
H	1.521799	5.719251	-0.415079
C	-0.626732	3.320204	0.412300
H	-0.999172	2.665658	1.213586
H	-1.055888	4.323820	0.572741
H	-0.990600	2.944489	-0.554565

Al	3.335124	-1.626757	0.210242
Cl	2.831957	-2.343220	2.184423
Cl	3.680985	-3.273084	-1.140612
Cl	5.139872	-0.402385	0.390893

65

26b-B_1 Eopt -1992.394775

C	-1.577882	0.485168	0.269503
C	-0.930539	0.096614	-1.048872
C	0.168555	-0.652432	-1.091265
C	0.778306	-1.127657	0.206627
C	0.579683	-0.115568	1.313780
C	-0.509308	0.658776	1.367867
C	0.892516	-1.115338	-2.313343
C	2.311144	-1.289580	0.112274
C	2.948645	-0.340317	-0.953610
C	2.334666	-0.574337	-2.347062
C	4.425005	-0.702448	-0.921271
C	4.111274	-0.063019	1.319844
C	2.868385	-0.936445	1.506162
H	0.912829	-2.217965	-2.285240
H	-1.432029	0.444221	-1.953815
H	2.975992	-1.270188	-2.904381
H	4.722928	0.014925	2.225610
O	-2.425796	1.565740	0.191405
C	-0.708824	1.757812	2.364904
H	-1.751729	1.825118	2.704368
H	-0.053625	1.635380	3.237040
H	-0.472624	2.721612	1.889784
C	1.845752	0.045886	2.125287
H	1.688786	-0.105778	3.203723
H	0.354905	-0.823732	-3.225941
H	2.345596	0.369383	-2.907280
O	5.098613	-1.104927	-1.831811
O	4.975049	-0.532418	0.293148
C	2.892034	1.159551	-0.483432
H	1.838975	1.477044	-0.478135
C	3.420395	1.260756	0.972132
H	4.069915	2.137387	1.094987
H	3.047655	-1.820009	2.128541
H	2.548182	-2.323055	-0.180124
C	3.676313	2.101696	-1.395582
H	4.756500	1.881476	-1.383427
H	3.334401	2.058920	-2.438552
H	3.547867	3.138214	-1.050255
O	2.412900	1.346448	1.952461
O	0.114300	-2.371847	0.468203
C	0.495814	-3.084649	1.618770
H	0.432011	-2.468751	2.534020
H	-0.204035	-3.925775	1.718527
H	1.515684	-3.498449	1.537439
C	-2.496967	-0.747216	0.657960
H	-1.874707	-1.614892	0.945931
B	-2.090002	2.752604	-0.590663
F	-2.455192	2.578949	-1.947959
F	-0.704684	3.017201	-0.540243
F	-2.805947	3.826827	-0.058903
N	-3.859767	-0.077865	2.640629
N	-3.230819	-0.393849	1.793266
Si	-3.785693	-1.429231	-0.698868
C	-4.955038	-2.495995	0.298446
H	-4.418352	-3.285479	0.849093
H	-5.560747	-1.915870	1.013110
H	-5.655972	-2.991845	-0.393633
C	-4.643657	0.022835	-1.479032
H	-5.315077	-0.337998	-2.276116
H	-5.254004	0.560672	-0.736683

H	-3.929917	0.740124	-1.907872
C	-2.771718	-2.474456	-1.865755
H	-2.283928	-1.871223	-2.644183
H	-1.994715	-3.037086	-1.325208
H	-3.442421	-3.196752	-2.360430

65

26b-B_10 Eopt -1992.376879

C	1.375510	0.732938	-0.086280
C	0.604248	0.955382	1.207496
C	-0.489659	0.275073	1.551682
C	-0.928974	-0.905147	0.730051
C	-0.489438	-0.778926	-0.707971
C	0.579764	-0.084152	-1.101061
C	-1.477444	0.674355	2.605465
C	-2.457641	-1.006608	0.640508
C	-3.168220	0.378297	0.660093
C	-2.758779	1.187532	1.912669
C	-4.640248	0.002722	0.659191
C	-3.943075	-1.042578	-1.338417
C	-2.739485	-1.729570	-0.686160
H	-1.717127	-0.198078	3.234815
H	0.930037	1.825499	1.783536
H	-3.591176	1.186207	2.629739
H	-4.407677	-1.635711	-2.133859
O	1.593141	1.968473	-0.667728
C	0.993109	0.076245	-2.533534
H	2.087627	0.136126	-2.631549
H	0.624531	-0.749508	-3.156660
H	0.595303	1.016902	-2.941619
C	-1.562561	-1.346186	-1.617194
H	-1.207455	-2.175328	-2.246831
H	-1.071582	1.456320	3.262235
H	-2.591484	2.234589	1.624938
O	-5.471365	0.285806	1.479964
O	-4.980247	-0.737775	-0.411486
C	-2.959211	1.157712	-0.687884
H	-1.905301	1.466578	-0.740456
C	-3.235769	0.205794	-1.882777
H	-3.801115	0.719437	-2.671470
H	-2.855342	-2.811421	-0.560050
H	-2.814418	-1.582965	1.504642
C	-3.837588	2.404170	-0.785058
H	-4.907845	2.147737	-0.846971
H	-3.702143	3.077088	0.073224
H	-3.582292	2.969200	-1.693844
O	-2.080648	-0.333965	-2.482969
O	-0.511890	-2.175892	1.257999
C	0.493929	-2.225933	2.229826
H	1.450221	-1.803818	1.882980
H	0.214650	-1.709447	3.164133
H	0.658608	-3.288904	2.456568
C	2.799018	0.002318	0.160302
H	3.543553	0.597045	-0.403943
B	2.687264	2.858581	-0.328769
F	2.347010	4.151304	-0.699302
F	3.866456	2.458060	-1.022550
F	2.985835	2.800966	1.055869
N	3.451560	0.267250	2.549709
N	3.183241	0.177888	1.484760
Si	3.344017	-1.857894	-0.402818
C	4.527088	-1.523900	-1.806621
H	4.030482	-1.029695	-2.655626
H	5.372139	-0.895563	-1.483983
H	4.934023	-2.483079	-2.167389
C	4.258384	-2.562605	1.068975
H	5.120829	-1.942866	1.362612

H 3.616494 -2.718713 1.950359
H 4.652866 -3.548881 0.772309
C 1.952439 -2.968679 -0.946226
H 1.570891 -2.678268 -1.935514
H 2.390703 -3.977971 -1.038930
H 1.107810 -3.016541 -0.246221

65

26b-B_11 Eopt -1992.387777

C -1.568203 0.683466 0.352996
C -0.980512 0.601270 -1.040671
C 0.039059 -0.186811 -1.371320
C 0.565624 -1.206862 -0.399685
C 0.302482 -0.820016 1.033490
C -0.729414 -0.054581 1.407258
C 0.847405 -0.099765 -2.625801
C 2.099242 -1.346372 -0.480371
C 2.784270 -0.011717 -0.910405
C 2.231538 0.480952 -2.266838
C 4.256654 -0.375792 -0.987771
C 3.817447 -0.915249 1.262518
C 2.568381 -1.737733 0.933497
H 0.958876 -1.107792 -3.056527
H -1.366306 1.327200 -1.760558
H 2.953838 0.236929 -3.057848
H 4.380385 -1.307149 2.117028
O -1.699770 1.996513 0.787982
C -0.989418 0.324070 2.835319
H -0.480495 -0.358235 3.528138
H -0.640706 1.347076 3.034363
H -2.066416 0.301395 3.067067
C 1.502953 -1.160561 1.898669
H 1.267712 -1.823604 2.744294
H 0.360294 0.534445 -3.379165
H 2.145577 1.575215 -2.242225
O 4.983334 -0.294651 -1.941960
O 4.736402 -0.839572 0.180057
C 2.694193 1.061280 0.235952
H 1.644069 1.367750 0.334816
C 3.140666 0.422727 1.577937
H 3.776816 1.111434 2.149186
H 2.717450 -2.816560 1.049457
H 2.358129 -2.109469 -1.228511
C 3.518884 2.312368 -0.059746
H 4.600615 2.099764 -0.080329
H 3.245393 2.772718 -1.018878
H 3.343305 3.062441 0.725509
O 2.084456 0.032570 2.428143
O -0.100238 -2.414751 -0.767894
C 0.264048 -3.606822 -0.123009
H 1.269376 -3.956189 -0.416305
H 0.225768 -3.524951 0.978275
H -0.464327 -4.368949 -0.433622
C -3.058718 0.134320 0.371901
H -3.454630 0.283182 1.392551
B -1.206627 3.200172 0.126604
F -1.415455 4.256728 1.016000
F -1.933436 3.446488 -1.066011
F 0.161901 3.114339 -0.192539
N -4.316983 1.768767 -1.048037
N -3.794780 1.056175 -0.393006
Si -3.632317 -1.692638 -0.133681
C -2.933129 -2.780361 1.205366
H -3.175021 -3.833064 0.984416
H -1.842890 -2.683192 1.280284
H -3.378225 -2.527361 2.180632
C -5.497443 -1.589323 -0.009253

H	-5.825249	-1.249453	0.986228
H	-5.934149	-0.920231	-0.768654
H	-5.922863	-2.593035	-0.176474
C	-3.102732	-2.069320	-1.875560
H	-2.015238	-2.216046	-1.929948
H	-3.605324	-2.993968	-2.205977
H	-3.398080	-1.262713	-2.565919

65

26b-B_12 Eopt -1992.378323

C	-1.489247	0.712085	-0.606724
C	-0.468192	0.472247	-1.713719
C	0.692106	-0.164795	-1.531758
C	0.926109	-0.783294	-0.151898
C	0.386961	0.162032	0.904013
C	-0.737895	0.860852	0.727355
C	1.756062	-0.228700	-2.605112
C	2.407974	-0.983188	0.237034
C	3.367992	-0.118287	-0.618966
C	3.175431	-0.505105	-2.082985
C	4.755806	-0.488989	-0.132487
C	3.796811	0.258829	1.874957
C	2.540822	-0.604928	1.724441
H	1.492172	-1.004834	-3.341945
H	-0.731976	0.927826	-2.672553
H	3.400415	-1.579901	-2.158858
H	4.108890	0.388409	2.917298
O	-2.150177	1.880897	-0.916431
C	-1.176227	1.956254	1.649707
H	-0.763232	1.826792	2.658658
H	-0.810973	2.918806	1.258045
H	-2.266653	2.029172	1.706181
C	1.403070	0.390401	1.996253
H	0.976081	0.313615	3.006570
H	1.733421	0.725146	-3.152464
H	3.914423	0.000931	-2.718986
O	5.655424	-0.943822	-0.787326
O	4.924200	-0.261422	1.181015
C	3.192751	1.396564	-0.277941
H	2.181092	1.707318	-0.579091
C	3.261277	1.562336	1.265767
H	3.853858	2.445368	1.540239
H	2.519636	-1.477742	2.386900
H	2.699377	-2.028256	0.076713
C	4.200289	2.287970	-0.997195
H	5.238024	2.059948	-0.703821
H	4.127429	2.183337	-2.089307
H	4.011708	3.343480	-0.750474
O	1.999776	1.691692	1.880620
O	0.192819	-2.007361	-0.032477
C	0.577503	-3.066691	-0.873712
H	1.569589	-3.475515	-0.619342
H	-0.158635	-3.870612	-0.734165
H	0.588298	-2.778361	-1.940197
C	-2.563752	-0.504502	-0.568016
H	-3.483985	-0.109460	-1.038248
B	-3.560511	2.166954	-0.751989
F	-3.743515	3.540910	-0.801878
F	-4.062975	1.649937	0.474494
F	-4.316600	1.542003	-1.783772
N	-2.013433	-2.324819	-2.178583
N	-2.220009	-1.509370	-1.466627
Si	-3.298145	-1.436316	1.053061
C	-5.104946	-1.575469	0.601528
H	-5.555205	-0.577646	0.487762
H	-5.243257	-2.129115	-0.341529
H	-5.651094	-2.115304	1.392403

C	-2.503910	-3.123743	1.160252
H	-1.424829	-3.040338	1.354729
H	-2.973307	-3.660239	2.002046
H	-2.659470	-3.737655	0.258880
C	-3.046515	-0.511099	2.644650
H	-3.513539	0.481472	2.627778
H	-3.549805	-1.114068	3.420466
H	-1.987959	-0.419811	2.924397

65

26b-B_13 Eopt -1992.385650

C	-1.465358	0.629983	0.196176
C	-0.806851	0.633898	-1.171625
C	0.299039	-0.046464	-1.468208
C	0.856725	-1.054521	-0.499180
C	0.491435	-0.733527	0.927605
C	-0.617520	-0.068819	1.269472
C	1.162617	0.149771	-2.673591
C	2.398509	-1.077169	-0.501063
C	3.006741	0.313339	-0.862122
C	2.493145	0.798086	-2.235982
C	4.504263	0.055892	-0.868960
C	3.988590	-0.573974	1.341117
C	2.822140	-1.474453	0.925726
H	1.356584	-0.831842	-3.135291
H	-1.212652	1.365567	-1.874742
H	3.265260	0.604775	-2.993083
H	4.533238	-0.947561	2.215399
O	-1.563076	1.936719	0.648177
C	-0.972136	0.244192	2.693553
H	-0.522791	-0.482996	3.383073
H	-0.614405	1.246550	2.967965
H	-2.061274	0.239801	2.851755
C	1.668599	-1.004226	1.846187
H	1.442464	-1.703315	2.665045
H	0.669070	0.779642	-3.426462
H	2.352852	1.886858	-2.203895
O	5.270463	0.213325	-1.781477
O	4.955001	-0.402739	0.312047
C	2.785053	1.348550	0.301755
H	1.710849	1.577555	0.354189
C	3.200218	0.702832	1.652150
H	3.753364	1.418130	2.274765
H	3.044088	-2.542611	1.022076
H	2.750091	-1.798553	-1.252849
C	3.549559	2.653361	0.083786
H	4.640649	2.500060	0.119848
H	3.306800	3.124584	-0.878584
H	3.291830	3.370211	0.877409
O	2.130379	0.216161	2.430744
O	0.311555	-2.298927	-0.934534
C	0.722409	-3.478831	-0.295516
H	0.637109	-3.422214	0.804089
H	0.053248	-4.277301	-0.646753
H	1.757943	-3.760917	-0.554243
C	-2.905015	-0.048365	0.157896
H	-3.453318	0.275942	1.059012
B	-2.508175	2.957528	0.230203
F	-2.119511	4.158516	0.811036
F	-3.819350	2.616526	0.661731
F	-2.558944	3.091406	-1.177810
N	-4.218410	0.838174	-1.777813
N	-3.653405	0.496345	-0.897292
Si	-3.252878	-2.008966	0.033092
C	-5.112523	-2.085205	0.231988
H	-5.440026	-1.640565	1.185286
H	-5.652009	-1.586877	-0.589718

H	-5.425421	-3.142908	0.231281
C	-2.745053	-2.588526	-1.659034
H	-3.283791	-2.036378	-2.446168
H	-1.662124	-2.472808	-1.806400
H	-3.006119	-3.654844	-1.765360
C	-2.422944	-2.824733	1.484331
H	-1.329119	-2.769957	1.439336
H	-2.761706	-2.375921	2.431085
H	-2.721016	-3.886742	1.490333

65

26b-B_14 Eopt -1992.387794

C	-1.603649	0.844371	0.084961
C	-1.016721	0.505468	-1.269495
C	-0.025135	-0.362196	-1.454207
C	0.420879	-1.249900	-0.325499
C	0.168663	-0.632010	1.026824
C	-0.811980	0.248496	1.258967
C	0.822832	-0.494356	-2.678079
C	1.939503	-1.505901	-0.347108
C	2.722908	-0.288173	-0.929523
C	2.224067	0.062981	-2.350117
C	4.167036	-0.755848	-0.924638
C	3.657063	-0.969977	1.365603
C	2.353533	-1.735568	1.118063
H	0.892552	-1.556905	-2.961702
H	-1.359834	1.137748	-2.092959
H	2.952387	-0.302727	-3.086896
H	4.178970	-1.286823	2.275479
O	-1.701823	2.228118	0.208915
C	-1.104214	0.800977	2.624662
H	-0.168573	1.056865	3.137795
H	-1.716837	1.709246	2.567292
H	-1.637672	0.053292	3.235395
C	1.317547	-0.954322	1.969121
H	1.004215	-1.483870	2.881079
H	0.394648	0.051860	-3.529952
H	2.185294	1.155081	-2.454942
O	4.914795	-0.841425	-1.862297
O	4.593228	-1.101643	0.303158
C	2.687613	0.926252	0.068356
H	1.659235	1.303663	0.116767
C	3.077109	0.442390	1.490791
H	3.755454	1.154551	1.978143
H	2.418767	-2.796449	1.381703
H	2.156780	-2.377813	-0.980798
C	3.585966	2.082342	-0.364748
H	4.654638	1.811726	-0.336895
H	3.350908	2.434061	-1.378622
H	3.437994	2.931649	0.318337
O	1.991952	0.237841	2.369416
O	-0.325162	-2.453342	-0.519863
C	-0.047097	-3.562214	0.293507
H	0.929132	-4.022762	0.062121
H	-0.077389	-3.320383	1.371123
H	-0.829563	-4.306867	0.090088
C	-3.111087	0.390835	0.212791
H	-3.475371	0.709423	1.206975
B	-0.583253	3.169236	0.107331
F	-1.138179	4.443780	0.276638
F	0.057843	3.103810	-1.145767
F	0.383534	2.940756	1.104422
N	-4.367633	1.820946	-1.418487
N	-3.830406	1.218687	-0.671496
Si	-3.789450	-1.460593	-0.008267
C	-3.153578	-2.371873	1.484563
H	-3.432053	-3.435737	1.406817

H	-2.061913	-2.304684	1.571518
H	-3.609431	-1.969167	2.402744
C	-5.644469	-1.228924	0.090604
H	-6.126895	-2.220730	0.084301
H	-5.947527	-0.716973	1.017943
H	-6.046483	-0.666520	-0.767971
C	-3.295932	-2.121014	-1.674103
H	-2.217500	-2.327331	-1.709389
H	-3.846674	-3.058439	-1.860447
H	-3.560287	-1.412825	-2.476110

65

26b-B_15 Eopt -1992.385011

C	-1.470061	0.659356	0.168268
C	-0.827259	0.565511	-1.207208
C	0.287750	-0.121008	-1.465551
C	0.847723	-1.056372	-0.425808
C	0.495090	-0.633028	0.974214
C	-0.599811	0.063830	1.281774
C	1.195706	0.110358	-2.637998
C	2.382698	-1.074081	-0.428408
C	3.000935	0.313903	-0.782418
C	2.464564	0.833800	-2.136574
C	4.490498	0.017284	-0.813098
C	3.983701	-0.621898	1.402166
C	2.783555	-1.483958	0.996597
H	1.477373	-0.847738	-3.100994
H	-1.229795	1.268430	-1.941075
H	3.257635	0.752050	-2.892687
H	4.532503	-1.015059	2.265128
O	-1.591174	1.999402	0.498518
C	-0.955411	0.464663	2.682625
H	-0.705457	1.520955	2.856074
H	-2.035849	0.358482	2.869921
H	-0.420129	-0.144426	3.422240
C	1.645004	-0.960822	1.907622
H	1.368176	-1.655349	2.713881
H	0.704677	0.719363	-3.409943
H	2.228058	1.902325	-2.040538
O	5.250497	0.161914	-1.732929
O	4.940143	-0.469669	0.358009
C	2.829606	1.345889	0.390217
H	1.764215	1.609023	0.456379
C	3.239930	0.679449	1.730744
H	3.823345	1.371362	2.352270
H	2.954990	-2.561393	1.094325
H	2.753204	-1.789506	-1.174247
C	3.633568	2.625484	0.163524
H	3.403609	3.353543	0.955536
H	4.719455	2.438220	0.193624
H	3.399810	3.098939	-0.800255
O	2.159976	0.225699	2.513665
O	0.336437	-2.386922	-0.541231
C	0.531765	-3.081447	-1.746287
H	1.598295	-3.217714	-1.996261
H	0.087201	-4.076791	-1.610143
H	0.031653	-2.594355	-2.601266
C	-2.887334	-0.058428	0.236004
H	-3.418983	0.335448	1.119015
B	-2.573055	2.952065	0.010327
F	-2.673084	2.938124	-1.402115
F	-2.192922	4.214563	0.448548
F	-3.860568	2.631544	0.519580
N	-4.289203	0.602746	-1.727320
N	-3.684451	0.361436	-0.839855
Si	-3.163898	-2.038279	0.296369
C	-4.968244	-2.149384	0.779743

H	-5.140447	-1.738049	1.787210
H	-5.632917	-1.626849	0.072846
H	-5.272300	-3.209515	0.793377
C	-2.900454	-2.656080	-1.443530
H	-3.754843	-2.399806	-2.090260
H	-1.987492	-2.234250	-1.884712
H	-2.803887	-3.753821	-1.439219
C	-2.086799	-2.810580	1.598612
H	-2.491984	-3.819090	1.790757
H	-1.050742	-2.902419	1.248505
H	-2.112137	-2.251820	2.546178

65

26b-B_16 Eopt -1992.387446

C	-1.602986	0.856660	0.017806
C	-1.028177	0.418346	-1.317254
C	-0.019933	-0.444169	-1.449878
C	0.423702	-1.249289	-0.260021
C	0.170265	-0.546454	1.046545
C	-0.803114	0.349888	1.225409
C	0.902337	-0.530688	-2.630149
C	1.934225	-1.500312	-0.256187
C	2.739490	-0.285198	-0.810482
C	2.243694	0.116939	-2.220207
C	4.165788	-0.802980	-0.802781
C	3.623459	-1.035170	1.485226
C	2.294035	-1.749464	1.214562
H	1.068655	-1.578809	-2.923183
H	-1.354495	1.020478	-2.170064
H	3.016193	-0.130981	-2.961359
H	4.129528	-1.380751	2.393502
O	-1.699366	2.246489	0.018412
C	-1.122829	0.962343	2.558394
H	-0.205179	1.119185	3.139129
H	-1.620712	1.932832	2.440537
H	-1.784205	0.296755	3.139048
C	1.267174	-0.921092	2.031801
H	0.887853	-1.440706	2.923319
H	0.490794	-0.001760	-3.501288
H	2.104746	1.205493	-2.247904
O	4.919242	-0.905283	-1.733994
O	4.563864	-1.184274	0.425629
C	2.747772	0.915936	0.201531
H	1.737209	1.341295	0.237658
C	3.095927	0.398735	1.621287
H	3.793174	1.076717	2.130450
H	2.297338	-2.813354	1.475113
H	2.179733	-2.365781	-0.885577
C	3.706971	2.028109	-0.217114
H	4.761074	1.708563	-0.166840
H	3.507897	2.381211	-1.238585
H	3.586268	2.888398	0.457689
O	1.984527	0.229500	2.473477
O	-0.284360	-2.487611	-0.142069
C	-0.209216	-3.411628	-1.196911
H	-0.596639	-3.004615	-2.146762
H	0.815451	-3.785029	-1.369076
H	-0.838159	-4.265817	-0.909459
C	-3.106260	0.423901	0.225773
H	-3.448867	0.874326	1.175576
B	-0.579129	3.179703	-0.121951
F	0.152254	2.970118	-1.308862
F	0.312956	3.086722	0.963632
F	-1.146181	4.459565	-0.150425
N	-4.394881	1.624966	-1.557484
N	-3.842195	1.128441	-0.746527
Si	-3.787667	-1.439697	0.255681

C	-2.941873	-2.305330	1.665056
H	-3.018638	-1.723719	2.596848
H	-3.452335	-3.269832	1.827228
H	-1.886253	-2.498633	1.431195
C	-5.609929	-1.198539	0.605352
H	-5.774300	-0.704775	1.576418
H	-6.118940	-0.610878	-0.175965
H	-6.101058	-2.185257	0.644656
C	-3.522219	-2.157333	-1.441314
H	-2.471250	-2.081083	-1.747813
H	-3.811346	-3.221130	-1.447654
H	-4.143355	-1.636426	-2.187928

65

26b-B_17 Eopt -1992.386569

C	1.563236	0.719604	-0.312077
C	0.994874	0.538746	1.083306
C	-0.030675	-0.262462	1.374336
C	-0.552429	-1.223010	0.339451
C	-0.294569	-0.751782	-1.065940
C	0.714938	0.054134	-1.402200
C	-0.902961	-0.131847	2.588289
C	-2.078935	-1.365262	0.405150
C	-2.786187	-0.033003	0.804903
C	-2.230042	0.517762	2.139379
C	-4.244187	-0.445880	0.895440
C	-3.787099	-1.018049	-1.349219
C	-2.506315	-1.787669	-1.007996
H	-1.103651	-1.116859	3.036181
H	1.363131	1.245670	1.831111
H	-2.987679	0.394267	2.925610
H	-4.340958	-1.443996	-2.193219
O	1.689630	2.064002	-0.639499
C	0.956762	0.522355	-2.806607
H	0.460931	-0.129837	-3.536724
H	0.575427	1.544961	-2.940025
H	2.031072	0.549781	-3.047920
C	-1.452302	-1.163683	-1.957439
H	-1.152590	-1.825905	-2.782392
H	-0.429032	0.494097	3.357300
H	-2.050263	1.595270	2.030375
O	-4.972512	-0.374157	1.849147
O	-4.705553	-0.953374	-0.262799
C	-2.741993	1.019991	-0.360157
H	-1.704516	1.365778	-0.462238
C	-3.161991	0.341256	-1.689569
H	-3.824210	0.992880	-2.274601
H	-2.596261	-2.874017	-1.116143
H	-2.359907	-2.116027	1.155599
C	-3.619266	2.238282	-0.078117
H	-3.468660	2.992391	-0.864778
H	-4.691329	1.981056	-0.066133
H	-3.374167	2.711424	0.882756
O	-2.086680	-0.019096	-2.527357
O	0.074706	-2.506200	0.410005
C	-0.035976	-3.255941	1.592482
H	0.388775	-2.736641	2.468357
H	-1.077245	-3.539360	1.824795
H	0.539008	-4.178442	1.430779
C	3.051899	0.177974	-0.424889
H	3.412145	0.426844	-1.439333
B	1.147717	3.209207	0.083933
F	-0.220967	3.056048	0.383687
F	1.324489	4.322239	-0.741211
F	1.856797	3.411769	1.294333
N	4.344662	1.680497	1.104988
N	3.810364	1.027175	0.399617

Si	3.646434	-1.684768	-0.113424
C	5.479469	-1.590910	-0.474189
H	6.005181	-0.901784	0.206623
H	5.927166	-2.589665	-0.339291
H	5.674563	-1.273936	-1.511167
C	3.342623	-2.086745	1.678582
H	2.290204	-1.937134	1.950272
H	3.609398	-3.137550	1.877993
H	3.964542	-1.452419	2.330798
C	2.759339	-2.738625	-1.360906
H	3.234314	-3.734314	-1.369904
H	1.700211	-2.853596	-1.092839
H	2.841669	-2.317249	-2.375147

65

26b-B_18 Eopt -1992.384753

C	1.444503	0.651304	0.150793
C	0.707424	0.357002	1.445086
C	-0.410952	-0.360177	1.529051
C	-0.914072	-1.115742	0.327817
C	-0.477055	-0.480697	-0.968005
C	0.648081	0.232105	-1.096035
C	-1.335250	-0.435878	2.702293
C	-2.454218	-1.124852	0.247951
C	-3.086311	0.150763	0.886129
C	-2.644759	0.305213	2.358440
C	-4.581122	-0.091225	0.758655
C	-3.951548	-0.203446	-1.507979
C	-2.803511	-1.182850	-1.250940
H	-1.548448	-1.495660	2.916020
H	1.081037	0.911041	2.312744
H	-3.452605	-0.051288	3.011910
H	-4.449533	-0.364090	-2.470644
O	1.559844	2.033928	0.095063
C	1.062390	0.865752	-2.390561
H	0.746763	1.918657	-2.418770
H	2.153909	0.862229	-2.512415
H	0.611288	0.349995	-3.248274
C	-1.607199	-0.523327	-1.979990
H	-1.338700	-1.018525	-2.924984
H	-0.882961	0.000674	3.603438
H	-2.508446	1.371580	2.583423
O	-5.393141	-0.140569	1.643459
O	-4.969337	-0.265761	-0.516673
C	-2.811765	1.422956	0.002258
H	-1.737517	1.651065	0.056492
C	-3.154930	1.105474	-1.478974
H	-3.679279	1.947634	-1.949205
H	-3.016027	-2.199037	-1.599550
H	-2.840484	-1.995732	0.797019
C	-3.592115	2.648718	0.474424
H	-4.679456	2.515927	0.351008
H	-3.399284	2.885964	1.529714
H	-3.298621	3.525994	-0.120902
O	-2.044187	0.802192	-2.292121
O	-0.388828	-2.431375	0.495070
C	-0.752780	-3.423644	-0.428392
H	-0.615988	-3.101953	-1.475927
H	-0.091101	-4.282731	-0.247038
H	-1.796107	-3.760533	-0.297127
C	2.871406	-0.041431	0.138894
H	3.500228	0.483522	-0.603966
B	2.737144	2.853537	-0.125512
F	2.314690	4.166064	-0.299102
F	3.478740	2.436727	-1.265969
F	3.615126	2.768869	0.983377
N	3.981872	0.290451	2.354080

N	3.507910	0.200967	1.364443
Si	3.224402	-1.971907	-0.227700
C	5.091563	-1.998145	-0.343036
H	5.456314	-1.351697	-1.157108
H	5.581128	-1.687340	0.594059
H	5.421460	-3.028376	-0.558004
C	2.632692	-2.963586	1.229714
H	3.149948	-2.661338	2.154824
H	1.547193	-2.854341	1.363998
H	2.863227	-4.028071	1.055693
C	2.477026	-2.382236	-1.880454
H	1.380935	-2.373380	-1.871974
H	2.830850	-1.682431	-2.653404
H	2.817666	-3.393249	-2.161337

65

26b-B_19 Eopt -1992.382981

C	1.616107	0.740091	0.469858
C	0.662660	0.623817	1.652452
C	-0.385917	-0.201231	1.651260
C	-0.608829	-1.096009	0.451947
C	-0.121115	-0.458229	-0.819485
C	0.900068	0.398028	-0.847007
C	-1.492294	-0.223348	2.662694
C	-2.102883	-1.337262	0.183263
C	-2.994095	-0.106681	0.554949
C	-2.778239	0.331201	2.020397
C	-4.398278	-0.627885	0.299548
C	-3.456232	-0.899565	-1.845921
C	-2.204552	-1.613498	-1.325305
H	-1.667940	-1.256911	3.000200
H	0.867060	1.297101	2.488790
H	-3.649023	0.031425	2.619478
H	-3.789220	-1.261655	-2.824948
O	2.231636	1.971587	0.410372
C	1.308971	1.134677	-2.088115
H	0.574775	1.927706	-2.289551
H	2.282107	1.622190	-1.969080
H	1.326339	0.474467	-2.967947
C	-1.046389	-0.806910	-1.964241
H	-0.529098	-1.340677	-2.775700
H	-1.232654	0.372478	3.548856
H	-2.729078	1.427506	2.060296
O	-5.303171	-0.718117	1.085672
O	-4.574578	-1.025638	-0.973413
C	-2.819707	1.076721	-0.463476
H	-1.816477	1.503105	-0.320746
C	-2.904262	0.531202	-1.911368
H	-3.495027	1.198568	-2.552495
H	-2.169647	-2.682569	-1.562172
H	-2.467834	-2.185433	0.777235
C	-3.849382	2.186356	-0.253498
H	-4.873095	1.847158	-0.482348
H	-3.843943	2.572194	0.775205
H	-3.624004	3.029422	-0.923306
O	-1.654672	0.350979	-2.534496
O	0.115331	-2.330919	0.554313
C	-0.191732	-3.203155	1.613818
H	-0.003277	-2.753285	2.604937
H	-1.235850	-3.558044	1.588596
H	0.463252	-4.078315	1.501833
C	2.878266	-0.232919	0.697064
H	3.631539	0.394488	1.207508
B	1.477426	3.222509	0.514139
F	0.160290	3.072170	0.028345
F	2.153472	4.186438	-0.240554
F	1.410263	3.646037	1.857250

N	2.481302	-2.044395	2.366988
N	2.623639	-1.234100	1.635191
Si	3.888825	-1.115187	-0.781153
C	2.732942	-2.183737	-1.778935
H	1.986510	-2.664030	-1.126399
H	2.197272	-1.615786	-2.552237
H	3.322791	-2.971022	-2.277214
C	4.761450	0.246668	-1.704135
H	5.187738	0.990954	-1.013973
H	5.593086	-0.206271	-2.269340
H	4.111029	0.765453	-2.420294
C	5.124574	-2.171041	0.145252
H	5.829809	-2.597809	-0.587482
H	5.712702	-1.584267	0.869030
H	4.655532	-3.017292	0.672385

65

26b-B_2 Eopt -1992.393598

C	-1.551267	0.494577	0.219763
C	-0.929246	-0.093003	-1.037412
C	0.171045	-0.844101	-0.994203
C	0.795956	-1.139492	0.350329
C	0.604426	-0.001426	1.320473
C	-0.473150	0.784611	1.284444
C	0.949826	-1.337160	-2.175777
C	2.323470	-1.289352	0.256741
C	2.959733	-0.384246	-0.849672
C	2.321234	-0.638483	-2.231977
C	4.426396	-0.779433	-0.803412
C	4.125400	-0.038967	1.414725
C	2.858619	-0.871253	1.636559
H	1.097663	-2.424898	-2.086118
H	-1.419530	0.162285	-1.978999
H	3.012962	-1.232384	-2.844898
H	4.742373	0.063284	2.314368
O	-2.355778	1.586951	-0.008730
C	-0.658070	1.984716	2.163443
H	-0.320879	2.881351	1.621970
H	-1.710937	2.159344	2.421498
H	-0.068092	1.903332	3.086015
C	1.844406	0.170128	2.170976
H	1.636732	0.070573	3.246502
H	0.401896	-1.158780	-3.111603
H	2.192155	0.322848	-2.746410
O	5.092326	-1.235825	-1.694020
O	4.976361	-0.574383	0.406804
C	2.946289	1.134123	-0.444832
H	1.902949	1.481013	-0.463815
C	3.470247	1.287669	1.005692
H	4.141914	2.151685	1.094536
H	2.997679	-1.723192	2.311064
H	2.587542	-2.327976	0.015072
C	3.765272	2.004254	-1.397649
H	3.647207	3.063010	-1.123316
H	4.840960	1.768445	-1.346424
H	3.446089	1.897063	-2.443426
O	2.457434	1.443140	1.970709
O	0.201662	-2.278199	0.990190
C	0.190166	-3.497918	0.289851
H	-0.282010	-4.237239	0.951625
H	-0.394846	-3.441677	-0.643353
H	1.205286	-3.858368	0.047705
C	-2.513883	-0.633495	0.781091
H	-1.920084	-1.493021	1.146850
B	-1.957502	2.658841	-0.917003
F	-0.562276	2.867768	-0.875529
F	-2.630853	3.817923	-0.526068

F	-2.314473	2.343768	-2.250943
N	-3.730107	0.303481	2.756172
N	-3.165987	-0.122378	1.911756
Si	-3.938220	-1.334012	-0.429244
C	-3.130784	-2.646628	-1.476810
H	-2.670220	-3.426885	-0.851714
H	-3.919598	-3.124299	-2.082538
H	-2.373295	-2.242710	-2.162667
C	-5.181064	-2.107056	0.735882
H	-5.949130	-2.624496	0.137103
H	-4.715455	-2.856177	1.396773
H	-5.703700	-1.363079	1.358647
C	-4.667995	0.078778	-1.389959
H	-5.165687	0.792985	-0.715979
H	-3.909720	0.632903	-1.959734
H	-5.424760	-0.320353	-2.086185

65

26b-B_20 Eopt -1992.383911

C	-1.457116	0.654304	-0.202407
C	-0.733014	0.224981	-1.469726
C	0.394204	-0.488262	-1.491256
C	0.900144	-1.111417	-0.215860
C	0.476090	-0.340349	1.004734
C	-0.637330	0.392371	1.068990
C	1.359859	-0.556961	-2.637743
C	2.433280	-1.107204	-0.139538
C	3.069294	0.147335	-0.814513
C	2.600984	0.289782	-2.281412
C	4.557462	-0.132369	-0.694804
C	3.940923	-0.172204	1.583708
C	2.761380	-1.124292	1.360801
H	1.665587	-1.598304	-2.820677
H	-1.102201	0.712175	-2.378752
H	3.431021	0.025754	-2.951270
H	4.445928	-0.319190	2.544853
O	-1.603681	2.029376	-0.316620
C	-1.051930	1.143298	2.298292
H	-2.134711	1.066236	2.472230
H	-0.527457	0.773242	3.188169
H	-0.827288	2.214052	2.187667
C	1.579007	-0.394378	2.044922
H	1.262235	-0.855696	2.991540
H	0.908025	-0.177528	-3.565010
H	2.358384	1.343138	-2.477601
O	5.361360	-0.229100	-1.583090
O	4.947913	-0.289070	0.583174
C	2.843345	1.448820	0.036183
H	1.776902	1.710074	-0.021427
C	3.183575	1.161273	1.522756
H	3.735784	1.998401	1.969574
H	2.925710	-2.136420	1.746044
H	2.843156	-1.986813	-0.652954
C	3.662905	2.631325	-0.478782
H	3.394207	3.540379	0.079479
H	4.744975	2.470177	-0.343797
H	3.482834	2.830840	-1.544401
O	2.063980	0.916058	2.341736
O	0.394546	-2.432085	-0.004103
C	0.666509	-3.422083	-0.962403
H	1.746493	-3.608033	-1.094336
H	0.207861	-4.349610	-0.593410
H	0.228688	-3.189826	-1.948689
C	-2.861253	-0.065470	-0.053596
H	-3.478746	0.533380	0.640784
B	-2.795776	2.843794	-0.169210
F	-3.700545	2.597913	-1.231899

F	-2.402988	4.176982	-0.179150
F	-3.494466	2.561996	1.036552
N	-4.057815	-0.038996	-2.248399
N	-3.548736	0.005918	-1.273286
Si	-3.130063	-1.955539	0.542750
C	-4.959384	-1.952276	0.932534
H	-5.569843	-1.630973	0.073006
H	-5.277442	-2.973705	1.200329
H	-5.187850	-1.293202	1.785267
C	-2.770223	-3.028019	-0.941023
H	-3.605745	-3.005732	-1.658925
H	-1.854931	-2.707453	-1.456701
H	-2.634412	-4.073813	-0.621500
C	-2.128162	-2.313101	2.065565
H	-2.537356	-3.238197	2.507209
H	-1.071905	-2.473048	1.814765
H	-2.216519	-1.514235	2.817075

65

26b-B_21 Eopt -1992.381647

C	1.646051	0.766012	-0.327728
C	0.751020	1.414848	0.723998
C	-0.420203	0.899038	1.106796
C	-0.755730	-0.483213	0.558519
C	-0.355784	-0.534748	-0.904146
C	0.776162	0.019564	-1.340435
C	-1.399800	1.658502	1.974446
C	-2.249481	-0.865153	0.557732
C	-3.173999	0.374770	0.653416
C	-2.836092	1.114852	1.945252
C	-4.577394	-0.197089	0.695215
C	-3.825883	-1.084688	-1.344719
C	-2.519470	-1.625294	-0.754863
H	-1.043117	1.668626	3.017072
H	1.073460	2.395855	1.077808
H	-2.984850	0.399910	2.769226
H	-4.229893	-1.712650	-2.146652
O	2.405085	1.691867	-0.996713
C	1.226557	0.024856	-2.766054
H	0.612365	-0.638826	-3.388040
H	1.165577	1.043717	-3.176379
H	2.280747	-0.279761	-2.853138
C	-1.458869	-1.122019	-1.747180
H	-1.104151	-1.891999	-2.447411
H	-1.388762	2.707918	1.644075
H	-3.549037	1.931161	2.124789
O	-5.397002	-0.044375	1.561014
O	-4.863879	-0.948373	-0.381771
C	-3.102159	1.217738	-0.660879
H	-2.086659	1.629991	-0.757961
C	-3.307942	0.262193	-1.869624
H	-3.965805	0.717026	-2.622312
H	-2.508299	-2.713427	-0.624457
H	-2.475351	-1.496302	1.426104
C	-4.090442	2.379840	-0.673035
H	-5.136087	2.033980	-0.626796
H	-3.922508	3.063214	0.171729
H	-3.975547	2.961346	-1.599933
O	-2.110863	-0.102506	-2.519021
O	0.023797	-1.478741	1.240529
C	-0.209736	-1.624117	2.620367
H	-1.214303	-2.021058	2.842663
H	0.525862	-2.348669	2.996757
H	-0.085322	-0.674144	3.170868
C	2.693020	-0.306117	0.330648
H	3.690280	0.119484	0.133309
B	3.200950	2.716256	-0.322276

F	4.275391	3.037627	-1.151677
F	3.691258	2.224319	0.918192
F	2.441445	3.870025	-0.063623
N	2.552486	-0.193530	2.812994
N	2.619701	-0.245983	1.714495
Si	2.973650	-2.211918	-0.213777
C	1.565496	-3.106769	-1.039376
H	1.904988	-4.149405	-1.170560
H	0.664297	-3.103365	-0.412442
H	1.325682	-2.704225	-2.032983
C	4.439926	-2.060397	-1.362735
H	5.315631	-1.636166	-0.846585
H	4.716480	-3.058846	-1.739568
H	4.213307	-1.424023	-2.232755
C	3.445772	-3.068593	1.380982
H	4.290744	-2.579473	1.891922
H	2.597658	-3.132734	2.082008
H	3.755378	-4.099814	1.143561

65

26b-B_3 Eopt -1992.388746

C	-1.407014	0.538399	-0.322857
C	-0.653158	-0.447262	-1.210583
C	0.462307	-1.056954	-0.813663
C	1.000486	-0.755256	0.565942
C	0.710687	0.677288	0.961484
C	-0.395771	1.315326	0.557747
C	1.273051	-2.048312	-1.584379
C	2.538188	-0.868575	0.637294
C	3.231633	-0.590157	-0.735610
C	2.706736	-1.539664	-1.829249
C	4.705121	-0.823560	-0.442270
C	4.242508	0.884021	1.109084
C	2.998488	0.185349	1.663319
H	1.309766	-2.973470	-0.984607
H	-1.100579	-0.630159	-2.189053
H	3.390598	-2.394787	-1.913795
H	4.794195	1.449025	1.868670
O	-2.152811	1.359454	-1.127772
C	-0.652462	2.764988	0.820422
H	-0.722007	3.307637	-0.134117
H	-1.611995	2.922793	1.329788
H	0.152918	3.212024	1.414577
C	1.925314	1.298414	1.621457
H	1.703660	1.728731	2.609149
H	0.788783	-2.299729	-2.537964
H	2.739013	-1.024249	-2.797852
O	5.437663	-1.618487	-0.967444
O	5.174376	-0.017633	0.525619
C	3.138802	0.929512	-1.127179
H	2.086360	1.160287	-1.348600
C	3.563079	1.799807	0.084427
H	4.192572	2.640616	-0.235135
H	3.146745	-0.232577	2.664967
H	2.807930	-1.890702	0.941146
C	3.982741	1.283683	-2.351016
H	3.820548	2.338815	-2.616863
H	5.060322	1.156482	-2.156833
H	3.725147	0.678898	-3.231061
O	2.487573	2.337929	0.816621
O	0.352815	-1.712749	1.413168
C	0.654208	-1.681222	2.787223
H	1.687035	-2.007328	2.996885
H	0.497979	-0.679569	3.226640
H	-0.030558	-2.386334	3.278097
C	-2.272058	-0.413432	0.626414
H	-1.549055	-0.970528	1.255202

B	-3.475906	1.925830	-0.932663
F	-4.420358	0.899942	-0.636624
F	-3.834702	2.565593	-2.113582
F	-3.536057	2.834485	0.144354
N	-3.645762	0.881873	2.260474
N	-3.024813	0.321715	1.544056
Si	-3.399761	-1.859772	-0.162216
C	-3.609322	-1.683957	-2.008656
H	-4.578644	-2.129472	-2.287784
H	-3.615131	-0.634358	-2.327695
H	-2.818925	-2.225905	-2.549065
C	-2.422699	-3.400164	0.253961
H	-1.406101	-3.356520	-0.165266
H	-2.340031	-3.550902	1.342011
H	-2.933690	-4.279518	-0.172829
C	-5.011215	-1.843622	0.783194
H	-5.610983	-2.712180	0.464032
H	-4.854682	-1.936498	1.870490
H	-5.595755	-0.934184	0.581596

65

26b-B_4 Eopt -1992.389660

C	-1.384718	0.434386	-0.376273
C	-0.598139	-0.453934	-1.337323
C	0.533207	-1.060900	-0.982112
C	1.034999	-0.875811	0.431620
C	0.704692	0.509384	0.950179
C	-0.403377	1.160940	0.574540
C	1.387526	-1.953990	-1.823245
C	2.571559	-0.964933	0.536858
C	3.294304	-0.548029	-0.784873
C	2.816901	-1.399543	-1.976503
C	4.764300	-0.780280	-0.473623
C	4.225298	0.766915	1.216042
C	2.981826	-0.002193	1.668460
H	1.426880	-2.932860	-1.316056
H	-1.027666	-0.557218	-2.335267
H	3.519324	-2.231367	-2.120890
H	4.745257	1.269578	2.039062
O	-2.148140	1.274848	-1.134952
C	-0.711749	2.573308	0.958950
H	-0.767492	3.201172	0.057911
H	-1.693016	2.653515	1.447460
H	0.056387	2.986341	1.622851
C	1.887585	1.090192	1.698246
H	1.629213	1.423260	2.714249
H	0.934556	-2.119465	-2.810379
H	2.865849	-0.795023	-2.891344
O	5.527407	-1.507467	-1.050684
O	5.190320	-0.060582	0.578863
C	3.181021	0.999325	-1.037290
H	2.130313	1.230023	-1.267298
C	3.554389	1.761124	0.260770
H	4.174473	2.639338	0.038080
H	3.111937	-0.508531	2.631163
H	2.853482	-2.005649	0.754306
C	4.050697	1.479567	-2.198693
H	5.124871	1.353343	-1.985954
H	3.829218	0.953410	-3.137223
H	3.874735	2.551608	-2.371789
O	2.448013	2.209219	1.007860
O	0.390998	-1.926603	1.166042
C	0.608647	-1.984695	2.555297
H	1.640260	-2.286672	2.803165
H	0.386996	-1.023655	3.053119
H	-0.074526	-2.747925	2.952716
C	-2.226676	-0.614854	0.504264

H	-1.499474	-1.400359	0.799518
B	-3.335858	2.038400	-0.795056
F	-3.712658	1.821689	0.556813
F	-4.399269	1.625495	-1.620773
F	-3.111387	3.403361	-0.998513
N	-2.903854	0.266056	2.739893
N	-2.604042	-0.098097	1.744508
Si	-3.763889	-1.619250	-0.270019
C	-3.623700	-1.590175	-2.124641
H	-4.532262	-2.061915	-2.535785
H	-3.575741	-0.554947	-2.490155
H	-2.753568	-2.155868	-2.488359
C	-3.499758	-3.327746	0.443585
H	-2.533974	-3.748757	0.120253
H	-3.517569	-3.310391	1.545684
H	-4.298375	-4.008533	0.105747
C	-5.358139	-0.867735	0.334625
H	-5.402043	-0.772892	1.431315
H	-5.517554	0.120610	-0.117034
H	-6.184607	-1.530548	0.027727

65

26b-B_5 Eopt -1992.388050

C	-1.438002	0.508440	-0.080903
C	-0.547295	0.897134	-1.251378
C	0.592727	0.270706	-1.541541
C	1.026855	-0.930756	-0.735807
C	0.492764	-0.874845	0.675255
C	-0.648803	-0.251697	0.994095
C	1.583949	0.667076	-2.589203
C	2.560439	-0.990730	-0.560151
C	3.223466	0.421327	-0.602068
C	2.885583	1.152039	-1.918503
C	4.708452	0.121260	-0.481341
C	3.927774	-0.858384	1.511851
C	2.811481	-1.639547	0.814024
H	1.793498	-0.214845	-3.216173
H	-0.891374	1.740434	-1.853388
H	3.725735	1.033899	-2.616219
H	4.360055	-1.391577	2.365842
O	-2.022130	1.587309	0.539165
C	-1.076051	-0.049750	2.418592
H	-0.727021	0.931952	2.771287
H	-2.166195	-0.050027	2.532818
H	-0.649883	-0.817093	3.078042
C	1.562875	-1.303127	1.662548
H	1.244360	-2.123341	2.322932
H	1.183235	1.451776	-3.245352
H	2.796056	2.228132	-1.719424
O	5.578275	0.406240	-1.260203
O	5.011773	-0.543756	0.647240
C	2.875753	1.249020	0.688790
H	1.804247	1.494776	0.656046
C	3.120370	0.370151	1.944339
H	3.601118	0.950357	2.742867
H	3.012257	-2.714248	0.748518
H	2.988882	-1.582943	-1.381645
C	3.669474	2.550542	0.790503
H	4.749005	2.364147	0.913603
H	3.536012	3.192381	-0.091062
H	3.332426	3.121352	1.668449
O	1.957413	-0.207139	2.492163
O	0.548351	-2.051008	-1.480376
C	0.762158	-3.333446	-0.948872
H	0.205125	-4.038408	-1.581947
H	1.825253	-3.628707	-0.970544
H	0.385685	-3.427238	0.086213

C	-2.680109	-0.330757	-0.680487
H	-3.221150	0.396268	-1.316060
B	-2.598098	2.754943	-0.120376
F	-1.605274	3.637768	-0.572856
F	-3.412152	3.394963	0.813043
F	-3.386594	2.368201	-1.243777
N	-1.965193	-2.160397	-2.210610
N	-2.284271	-1.332730	-1.556794
Si	-4.090729	-1.112669	0.468901
C	-4.801121	0.272085	1.485872
H	-4.053185	0.790890	2.098831
H	-5.266888	1.017978	0.823917
H	-5.584246	-0.138512	2.144823
C	-5.340586	-1.736469	-0.775390
H	-6.208390	-2.153107	-0.237358
H	-5.706255	-0.921538	-1.420273
H	-4.937121	-2.536795	-1.417024
C	-3.346921	-2.554129	1.395717
H	-2.838094	-3.241340	0.699157
H	-2.631254	-2.257920	2.174501
H	-4.159608	-3.120589	1.880466

65

26b-B_6 Eopt -1992.386892

C	-1.408077	0.397697	-0.131013
C	-0.543234	0.659817	-1.355761
C	0.627307	0.060722	-1.582769
C	1.102643	-1.032843	-0.657725
C	0.548394	-0.873664	0.734786
C	-0.606092	-0.254463	1.002509
C	1.652906	0.517615	-2.578809
C	2.625633	-0.990913	-0.458589
C	3.213287	0.453594	-0.506159
C	2.823442	1.174431	-1.816836
C	4.707894	0.206663	-0.389864
C	3.962358	-0.795447	1.613430
C	2.868447	-1.616026	0.923223
H	2.027414	-0.336735	-3.162653
H	-0.895446	1.455830	-2.014704
H	3.703355	1.231603	-2.472368
H	4.418426	-1.306576	2.468473
O	-1.957586	1.554795	0.367870
C	-1.069003	0.014631	2.403963
H	-0.754170	1.022899	2.711581
H	-2.161204	-0.008784	2.493562
H	-0.641381	-0.704574	3.114556
C	1.600583	-1.287200	1.748671
H	1.271763	-2.109778	2.400112
H	1.223720	1.238552	-3.288516
H	2.535748	2.208428	-1.582942
O	5.567573	0.516499	-1.170469
O	5.032523	-0.447977	0.740380
C	2.849139	1.276456	0.781304
H	1.772522	1.497987	0.748084
C	3.118065	0.412507	2.040809
H	3.581559	1.009541	2.837187
H	3.081959	-2.688942	0.869252
H	3.119611	-1.565015	-1.253995
C	3.615077	2.596070	0.867550
H	4.696186	2.433728	1.008870
H	3.482089	3.215703	-0.030187
H	3.255345	3.178226	1.728926
O	1.969389	-0.191966	2.588001
O	0.711465	-2.343724	-1.077967
C	1.007652	-2.735573	-2.395292
H	2.083786	-2.667771	-2.631827
H	0.708694	-3.788834	-2.486948

H	0.446037	-2.147933	-3.143685
C	-2.653770	-0.515508	-0.590986
H	-3.107977	0.000092	-1.455078
B	-2.606192	2.604764	-0.414105
F	-1.672196	3.406210	-1.092771
F	-3.337449	3.393062	0.472712
F	-3.486451	2.040967	-1.382246
N	-1.885922	-2.744544	-1.389110
N	-2.231582	-1.745266	-1.077472
Si	-4.220793	-0.899375	0.579360
C	-4.756164	0.637725	1.472267
H	-3.991555	1.046309	2.144697
H	-5.030036	1.423569	0.754903
H	-5.650250	0.379182	2.064741
C	-5.504213	-1.427623	-0.673030
H	-6.427664	-1.728176	-0.150776
H	-5.755660	-0.598495	-1.353409
H	-5.170323	-2.286461	-1.277886
C	-3.766855	-2.334674	1.688385
H	-3.554925	-3.246673	1.106837
H	-2.905826	-2.130515	2.340867
H	-4.631759	-2.557404	2.335742

65

26b-B_7 Eopt -1992.388042

C	-1.556750	0.450120	-0.445642
C	-0.603291	0.417580	-1.629377
C	0.503571	-0.323190	-1.662597
C	0.836636	-1.243592	-0.512361
C	0.251131	-0.745725	0.788716
C	-0.856687	0.005256	0.849462
C	1.560130	-0.296181	-2.720686
C	2.357194	-1.307995	-0.243666
C	3.092812	0.004520	-0.657578
C	2.847380	0.329566	-2.146333
C	4.554741	-0.294938	-0.373227
C	3.643335	-0.626075	1.771676
C	2.516490	-1.533930	1.271576
H	1.757583	-1.332078	-3.041143
H	-0.874206	1.073334	-2.460538
H	3.713727	-0.005942	-2.732500
H	4.012294	-0.903057	2.765610
O	-2.160200	1.679756	-0.288156
C	-1.316597	0.645720	2.126051
H	-2.397075	0.827262	2.139710
H	-1.043687	0.041116	3.001463
H	-0.827979	1.625391	2.228435
C	1.253502	-0.913776	1.916452
H	0.860987	-1.494688	2.764505
H	1.230720	0.267293	-3.604417
H	2.788985	1.418770	-2.271533
O	5.471883	-0.271467	-1.150045
O	4.776684	-0.617207	0.913163
C	2.726352	1.182402	0.316730
H	1.670153	1.441356	0.161368
C	2.883551	0.704248	1.784319
H	3.362189	1.475948	2.401297
H	2.661321	-2.587808	1.532334
H	2.787483	-2.131341	-0.832283
C	3.561069	2.438862	0.076674
H	3.198327	3.248415	0.727159
H	4.627041	2.278450	0.308549
H	3.490513	2.795872	-0.959934
O	1.674928	0.354902	2.420435
O	0.331554	-2.518283	-0.916318
C	0.483944	-3.597303	-0.029641
H	0.074726	-3.378129	0.973543

H	-0.079020	-4.440795	-0.453458
H	1.535578	-3.914477	0.076053
C	-2.829042	-0.434086	-0.823261
H	-3.258214	0.024957	-1.731795
B	-1.446134	2.952324	-0.356266
F	-2.176744	3.872540	0.403538
F	-1.359355	3.409137	-1.686230
F	-0.137386	2.839476	0.163017
N	-2.196207	-2.756474	-1.487458
N	-2.475464	-1.724073	-1.227015
Si	-4.382954	-0.636954	0.404931
C	-4.966616	1.069203	0.857243
H	-4.178765	1.692589	1.298721
H	-5.332877	1.585520	-0.043865
H	-5.806213	0.981542	1.566874
C	-5.633445	-1.518473	-0.671136
H	-6.560805	-1.671874	-0.094289
H	-5.888851	-0.925049	-1.563376
H	-5.283664	-2.510684	-0.999237
C	-3.862206	-1.755294	1.806582
H	-3.144328	-1.295041	2.498425
H	-4.759873	-2.031751	2.384666
H	-3.422990	-2.689040	1.417857

65

26b-B_8 Eopt -1992.383796

C	1.494721	0.798417	-0.060742
C	0.618518	1.276739	1.093724
C	-0.505108	0.647878	1.445507
C	-0.877379	-0.624223	0.718752
C	-0.442632	-0.584590	-0.721833
C	0.648045	0.073776	-1.114663
C	-1.533917	1.138427	2.419614
C	-2.397434	-0.826449	0.633268
C	-3.189474	0.517970	0.522948
C	-2.840191	1.481966	1.677819
C	-4.634878	0.049769	0.562343
C	-3.868702	-1.119719	-1.338852
C	-2.623129	-1.666040	-0.634114
H	-1.735386	0.351954	3.164031
H	0.950543	2.191598	1.587204
H	-3.673897	1.505048	2.392936
H	-4.291858	-1.811578	-2.075424
O	2.128912	1.848352	-0.678478
C	1.057754	0.223175	-2.546632
H	2.148269	0.227538	-2.663446
H	0.623015	-0.565196	-3.175517
H	0.706345	1.193614	-2.930483
C	-1.472039	-1.271337	-1.592438
H	-1.049209	-2.121550	-2.148194
H	-1.172930	2.020546	2.966661
H	-2.744220	2.499164	1.274971
O	-5.482557	0.348157	1.360262
O	-4.924912	-0.801823	-0.438052
C	-3.035004	1.186224	-0.890506
H	-2.003792	1.558325	-0.977961
C	-3.246771	0.120631	-1.995502
H	-3.844540	0.522396	-2.824104
H	-2.663519	-2.741039	-0.426944
H	-2.766494	-1.338723	1.531875
C	-3.996793	2.356994	-1.092940
H	-5.046816	2.022887	-1.122190
H	-3.907222	3.115942	-0.303531
H	-3.783025	2.849788	-2.053005
O	-2.052910	-0.382423	-2.545654
O	-0.222189	-1.772119	1.277912
C	-0.512876	-2.129777	2.606704

H 0.079133 -3.028818 2.827214
H -0.233977 -1.344203 3.330870
H -1.576444 -2.378665 2.759603
C 2.643525 -0.221969 0.482825
H 3.583236 0.360942 0.552364
B 3.231865 2.619381 -0.128355
F 3.306912 3.821785 -0.820068
F 4.457622 1.900161 -0.303065
F 3.090646 2.848658 1.253904
N 2.277696 -0.937724 2.836197
N 2.418162 -0.612132 1.792422
Si 3.299451 -1.822049 -0.517299
C 4.331487 -1.115363 -1.907718
H 5.278775 -1.674898 -1.970935
H 3.823382 -1.200516 -2.879268
H 4.573406 -0.056801 -1.728856
C 4.380571 -2.666475 0.756250
H 4.926464 -3.485091 0.258085
H 5.129585 -1.980531 1.184085
H 3.801075 -3.116102 1.578599
C 1.912753 -2.946004 -1.049558
H 1.422326 -2.593662 -1.967983
H 2.338917 -3.943904 -1.248888
H 1.152550 -3.034402 -0.258539

65

26b-B_9 Eopt -1992.386902

C 1.412720 0.411726 0.132276
C 0.545368 0.670434 1.356100
C -0.621443 0.063259 1.582062
C -1.091272 -1.031235 0.654221
C -0.538544 -0.864392 -0.738009
C 0.611343 -0.236136 -1.004109
C -1.649370 0.512102 2.579426
C -2.614752 -0.995174 0.454599
C -3.209616 0.446295 0.506898
C -2.823529 1.164977 1.819792
C -4.703030 0.191971 0.390234
C -3.952990 -0.799503 -1.616492
C -2.854772 -1.616827 -0.929227
H -2.019392 -0.345819 3.160841
H 0.893080 1.467616 2.016121
H -3.703646 1.215133 2.475622
H -4.406585 -1.310117 -2.473162
O 1.965431 1.568395 -0.363888
C 1.064195 0.052436 -2.405308
H 2.156100 0.062604 -2.498344
H 0.654097 -0.674217 -3.118830
H 0.718698 1.052635 -2.706365
C -1.588775 -1.278644 -1.753523
H -1.255570 -2.097086 -2.407988
H -1.223791 1.233124 3.291212
H -2.541621 2.201310 1.589254
O -5.563958 0.494517 1.172329
O -5.024720 -0.460398 -0.742110
C -2.850187 1.275331 -0.777797
H -1.774702 1.502215 -0.744080
C -3.115012 0.414178 -2.040013
H -3.581674 1.011445 -2.834374
H -3.062834 -2.690999 -0.878885
H -3.106900 -1.573810 1.247753
C -3.623044 2.591199 -0.859416
H -4.703290 2.423588 -1.001175
H -3.493262 3.208448 0.040430
H -3.266526 3.178207 -1.718830
O -1.963418 -0.182601 -2.589216
O -0.693457 -2.341073 1.071590

C	-1.001240	-2.743019	2.383413
H	-0.697300	-3.795148	2.471507
H	-0.450818	-2.157217	3.141558
H	-2.080009	-2.682988	2.609351
C	2.659241	-0.500514	0.591655
H	3.134145	0.039712	1.429820
B	2.610209	2.620684	0.417716
F	3.348672	3.402995	-0.468277
F	3.483477	2.061363	1.394949
F	1.672169	3.425740	1.086366
N	1.903161	-2.698230	1.481016
N	2.241801	-1.711014	1.126396
Si	4.196467	-0.929640	-0.598610
C	3.683267	-2.324737	-1.731726
H	2.894103	-2.043844	-2.442947
H	4.565811	-2.636210	-2.315679
H	3.341415	-3.206093	-1.165045
C	4.771043	0.615206	-1.453485
H	5.068291	1.372651	-0.714658
H	5.655166	0.353294	-2.059016
H	4.012207	1.062428	-2.107808
C	5.473387	-1.530322	0.628308
H	6.392305	-1.814879	0.089247
H	5.736643	-0.738030	1.347064
H	5.133717	-2.414964	1.191228

63

27a-Al_1 Eopt -3181.610108

C	-1.260932	0.740725	0.468580
C	-0.512168	-0.813360	-1.435902
C	0.746074	-1.172796	-1.115090
C	1.305413	-1.200446	0.295231
C	1.066378	-0.001970	1.194794
C	-0.018721	0.787151	1.309339
C	1.672161	-1.746200	-2.143909
C	2.837405	-1.354681	0.272256
C	3.555139	-0.562119	-0.857873
C	2.961788	-0.917148	-2.233976
C	5.005892	-0.990973	-0.708761
C	4.613906	-0.075018	1.427361
C	3.320202	-0.865334	1.640621
H	1.911039	-2.780707	-1.848107
H	-0.889913	-1.037224	-2.438644
H	3.705669	-1.469533	-2.823663
H	5.185856	0.080935	2.348643
C	-0.051025	1.868200	2.364537
H	-1.082526	2.117286	2.649130
H	0.484828	1.563927	3.272711
H	0.435448	2.787528	2.003630
C	2.305315	0.215317	2.051630
H	2.073972	0.200388	3.126215
H	1.183928	-1.793037	-3.126250
H	2.739413	0.005833	-2.787656
O	5.698736	-1.527905	-1.530465
O	5.502159	-0.701141	0.506611
C	3.564455	0.976043	-0.574368
H	2.530097	1.331505	-0.664506
C	4.009405	1.228354	0.886541
H	4.689539	2.087861	0.953713
H	3.406325	-1.675957	2.371533
H	3.056203	-2.420400	0.126018
C	4.435979	1.753681	-1.557571
H	4.322569	2.833950	-1.381742
H	5.504607	1.511461	-1.437804
H	4.161995	1.555574	-2.603511
O	2.937444	1.467386	1.768694
O	0.882967	-2.413378	0.926420

C	-0.461497	-2.558008	1.301110
H	-0.518160	-3.457303	1.929523
H	-0.829126	-1.704362	1.895038
H	-1.127050	-2.714374	0.434439
C	-1.518874	-0.276658	-0.539117
H	-2.149795	0.855541	1.108601
O	-2.710763	-0.620427	-0.798013
C	-2.496485	2.162690	-2.093647
H	-2.131261	1.390027	-2.787024
H	-3.519923	1.906372	-1.781802
H	-2.546670	3.112875	-2.651645
C	0.363621	2.831555	-1.195461
H	1.052799	2.971535	-0.349470
H	0.759597	2.041438	-1.851787
H	0.341860	3.769062	-1.775779
C	-2.082486	3.769954	0.468801
H	-1.375162	4.125271	1.230792
H	-2.345270	4.627075	-0.174108
H	-3.003757	3.430522	0.966105
Si	-1.369409	2.435427	-0.626934
Al	-4.351087	-0.761857	-0.014254
Cl	-4.043531	-2.108785	1.624289
Cl	-4.980008	1.175641	0.670327
Cl	-5.620065	-1.530089	-1.547966

63

27a-Al_2 Eopt -3181.605441

C	1.101244	1.511532	-0.853296
C	0.969858	-0.611631	0.595569
C	-0.278082	-1.080734	0.421532
C	-1.113036	-0.782041	-0.811370
C	-1.258495	0.665797	-1.257192
C	-0.308378	1.614478	-1.349147
C	-0.838235	-2.143232	1.310233
C	-2.579499	-1.253968	-0.608698
C	-3.114014	-1.063565	0.851833
C	-2.206187	-1.759686	1.878395
C	-4.503759	-1.680073	0.799710
C	-4.726215	-0.005999	-0.835062
C	-3.440636	-0.410116	-1.559966
H	-0.933878	-3.048873	0.687298
H	1.583386	-1.037081	1.396125
H	-2.706279	-2.660134	2.258562
H	-5.506386	0.355686	-1.513900
C	-0.639053	2.948606	-1.976629
H	-1.311816	2.831686	-2.836689
H	-1.152067	3.609654	-1.261346
H	0.266370	3.461605	-2.327265
C	-2.685535	0.922601	-1.702127
H	-2.731188	1.330341	-2.722290
H	-0.138155	-2.382883	2.121243
H	-2.050218	-1.098070	2.740801
O	-4.918150	-2.601522	1.449892
O	-5.303290	-1.072871	-0.092859
C	-3.359773	0.446606	1.194244
H	-2.379448	0.942116	1.239898
C	-4.160703	1.111592	0.050107
H	-4.928407	1.791134	0.443065
H	-3.624980	-0.903253	-2.519926
H	-2.634703	-2.326935	-0.842745
C	-4.070136	0.644051	2.532392
H	-5.100068	0.252007	2.510088
H	-3.543509	0.158006	3.365269
H	-4.133521	1.718323	2.761475
O	-3.362127	1.847969	-0.845436
O	-0.422640	-1.557467	-1.789808
C	-0.960352	-1.646125	-3.084878

H	-0.202990	-2.147586	-3.702490
H	-1.883676	-2.250066	-3.118507
H	-1.161410	-0.654276	-3.527133
C	1.672755	0.295547	-0.291654
H	1.808173	1.916345	-1.595165
O	2.917346	0.129587	-0.456076
C	1.553327	4.516900	-0.032426
H	1.909785	5.170852	0.781429
H	2.314940	4.531755	-0.827954
H	0.622522	4.948309	-0.425366
C	2.882155	2.341535	1.619369
H	3.769878	2.354525	0.967171
H	3.036111	3.096281	2.408731
H	2.827344	1.357109	2.107575
C	-0.195741	2.657416	1.741048
H	-0.300629	1.643117	2.156537
H	-0.118581	3.361402	2.586171
H	-1.108253	2.902077	1.176175
Si	1.333152	2.800460	0.677600
Al	4.236961	-1.047359	0.011469
Cl	6.025962	-0.105467	-0.679518
Cl	3.754082	-2.867550	-1.001717
Cl	4.221654	-1.275010	2.149472

63

27a-Al_3 Eopt -3181.607943

C	0.998268	1.418462	-0.871131
C	0.935201	-0.847607	0.361724
C	-0.330261	-1.280116	0.189458
C	-1.245463	-0.893912	-0.954128
C	-1.340728	0.557736	-1.370440
C	-0.384879	1.501790	-1.425771
C	-0.909984	-2.320096	1.096892
C	-2.700508	-1.289678	-0.649044
C	-3.124632	-1.046336	0.828072
C	-2.161670	-1.763529	1.793597
C	-4.534852	-1.612579	0.878314
C	-4.795314	0.018963	-0.800942
C	-3.566943	-0.445231	-1.589354
H	-1.175781	-3.201631	0.491748
H	1.548152	-1.341972	1.122055
H	-2.690588	-2.584304	2.296219
H	-5.605828	0.390780	-1.437355
C	-0.680292	2.812111	-2.124882
H	-0.805434	2.630589	-3.204749
H	-1.606364	3.270971	-1.756559
H	0.136551	3.537121	-2.011145
C	-2.768253	0.852658	-1.802929
H	-2.829289	1.240354	-2.829655
H	-0.174018	-2.644934	1.843806
H	-1.837165	-1.066148	2.578605
O	-4.944266	-2.494037	1.584439
O	-5.360036	-1.009284	0.005649
C	-3.294354	0.479460	1.139591
H	-2.294499	0.938980	1.113721
C	-4.141476	1.138547	0.021026
H	-4.862480	1.853458	0.438718
H	-3.805287	-0.965270	-2.522921
H	-2.808737	-2.361302	-0.860235
C	-3.913683	0.732904	2.512410
H	-4.958692	0.385878	2.559023
H	-3.357732	0.233505	3.318466
H	-3.916799	1.812071	2.726761
O	-3.379328	1.824172	-0.944349
O	-0.914363	-1.724752	-2.072721
C	0.285841	-1.495386	-2.763679
H	0.256344	-2.132472	-3.658059

H	0.390428	-0.446713	-3.091275
H	1.176296	-1.782457	-2.176505
C	1.620349	0.211344	-0.348902
H	1.713905	1.903950	-1.551820
O	2.890640	0.202536	-0.362422
C	2.692741	3.650894	0.390772
H	2.902634	4.323747	1.238420
H	3.567806	2.996730	0.250136
H	2.571133	4.266665	-0.514337
C	1.374917	1.641929	2.308884
H	0.485015	1.038242	2.544502
H	2.255758	0.982112	2.293508
H	1.517398	2.361109	3.133359
C	-0.392373	3.667206	0.897266
H	-0.400096	4.108229	1.908087
H	-0.435957	4.490322	0.170150
H	-1.302427	3.056643	0.786579
Si	1.162933	2.639818	0.739611
Al	4.295175	-0.870119	0.085498
Cl	4.168297	-1.284057	2.189293
Cl	4.066552	-2.634308	-1.108228
Cl	6.009641	0.302691	-0.413469

63

27a-Al_4 Eopt -3181.608665

C	1.415599	0.821149	-0.443581
C	0.489167	-0.215215	1.683996
C	-0.806293	-0.436999	1.405932
C	-1.164461	-0.833773	-0.027168
C	-0.973560	0.283087	-1.050762
C	0.172820	0.952198	-1.272291
C	-1.825101	-0.374534	2.515747
C	-2.640946	-1.275220	-0.217829
C	-3.627132	-0.676764	0.824063
C	-3.149357	-1.059310	2.215553
C	-4.965039	-1.303671	0.485719
C	-4.477098	-0.225755	-1.538986
C	-3.068122	-0.816778	-1.623280
H	-1.375043	-0.781646	3.432653
H	0.832992	-0.139058	2.720168
H	-3.036113	-2.153576	2.257660
H	-4.948216	-0.099889	-2.520102
C	0.299235	1.915702	-2.426990
H	-0.067240	2.917916	-2.153452
H	1.345353	2.016802	-2.747437
H	-0.286325	1.589171	-3.295705
C	-2.198895	0.410990	-1.925791
H	-1.934544	0.488617	-2.989264
H	-2.004326	0.694139	2.726904
H	-3.892360	-0.792069	2.978959
O	-5.645187	-1.985756	1.203798
O	-5.369306	-1.017314	-0.763428
C	-3.821191	0.860111	0.612562
H	-2.865317	1.368489	0.807486
C	-4.134215	1.118143	-0.880133
H	-4.919915	1.876185	-0.999502
H	-2.969078	-1.605574	-2.377373
H	-2.686341	-2.368342	-0.109050
C	-4.874629	1.457440	1.540287
H	-4.956789	2.540512	1.365103
H	-5.871317	1.018624	1.370974
H	-4.616452	1.310004	2.598786
O	-3.005327	1.551804	-1.604689
O	-0.253401	-1.903072	-0.287052
C	-0.287620	-2.509660	-1.554746
H	-1.217234	-3.081918	-1.719257
H	-0.164594	-1.780434	-2.373929

H	0.557622	-3.208734	-1.587832
C	1.536580	-0.105842	0.678349
H	2.292168	0.743876	-1.105094
O	2.649703	-0.642363	0.948175
C	2.584285	3.711585	-0.732213
H	2.928576	4.603892	-0.182720
H	3.472656	3.216898	-1.154785
H	1.940581	4.050366	-1.555428
C	2.805212	2.353550	1.981094
H	3.019420	3.352040	2.397965
H	2.334460	1.756565	2.776085
H	3.766255	1.890659	1.712203
C	0.024977	3.224120	0.987259
H	0.142810	4.213573	1.459979
H	-0.658735	3.335255	0.132337
H	-0.445354	2.554166	1.723780
Si	1.710997	2.590912	0.482326
Al	4.166524	-1.093462	0.029131
Cl	3.479678	-1.987928	-1.794553
Cl	5.200946	-2.438229	1.322019
Cl	5.276229	0.705100	-0.357852

63

27a-Al_5 Eopt -3181.608115

C	1.383353	0.977885	-0.420412
C	0.475843	-0.317011	1.567127
C	-0.817224	-0.530482	1.276739
C	-1.181926	-0.731834	-0.198599
C	-1.026252	0.532292	-1.038943
C	0.107432	1.246331	-1.167151
C	-1.827747	-0.653953	2.389814
C	-2.644927	-1.177413	-0.440264
C	-3.642048	-0.769699	0.679417
C	-3.134169	-1.330656	1.998647
C	-4.955319	-1.394800	0.253173
C	-4.514731	-0.021221	-1.599385
C	-3.083277	-0.537125	-1.766656
H	-1.360088	-1.176286	3.237230
H	0.837821	-0.361693	2.598914
H	-2.984229	-2.415291	1.880544
H	-4.997304	0.225894	-2.551433
C	0.196144	2.394918	-2.140843
H	-0.488120	2.272945	-2.988760
H	-0.068195	3.347469	-1.654390
H	1.215017	2.495743	-2.540929
C	-2.263384	0.753097	-1.880500
H	-2.007034	0.997259	-2.920019
H	-2.036530	0.367038	2.753370
H	-3.876581	-1.203490	2.798038
O	-5.605793	-2.199543	0.863876
O	-5.371877	-0.951120	-0.945258
C	-3.902317	0.770811	0.691469
H	-2.970843	1.287739	0.964772
C	-4.222900	1.226543	-0.751195
H	-5.037159	1.963535	-0.766018
H	-2.948049	-1.205025	-2.624650
H	-2.676582	-2.273098	-0.499403
C	-4.982581	1.182728	1.687165
H	-5.105618	2.275988	1.674882
H	-5.961213	0.737818	1.444573
H	-4.723693	0.888339	2.714467
O	-3.109414	1.802525	-1.394657
O	-0.262582	-1.648214	-0.790863
C	-0.162341	-2.943425	-0.250026
H	0.665927	-3.434485	-0.776722
H	0.070199	-2.933868	0.829353
H	-1.078404	-3.538787	-0.406398

C	1.505046	-0.096983	0.557278
H	2.228455	0.949943	-1.126649
O	2.596271	-0.711196	0.722402
C	0.226695	3.198543	1.446689
H	-0.526104	3.428336	0.677188
H	-0.203940	2.461341	2.142035
H	0.423420	4.122659	2.015544
C	2.659976	3.843791	-0.392545
H	1.968303	4.337869	-1.088166
H	3.100809	4.620408	0.255033
H	3.478416	3.386107	-0.969447
C	3.037963	2.085688	2.053997
H	3.297360	2.995569	2.620967
H	2.632938	1.354750	2.769701
H	3.970239	1.685134	1.627533
Si	1.832062	2.582408	0.711416
Al	4.134461	-1.138583	-0.161454
Cl	5.362524	-2.061633	1.321209
Cl	3.523722	-2.458528	-1.731762
Cl	5.019827	0.661351	-0.938040

63

27a-Al_6 Eopt -3181.605605

C	-1.363959	0.752057	0.444285
C	-0.531573	-0.508868	-1.631296
C	0.720706	-0.891073	-1.328889
C	1.190899	-1.093486	0.102329
C	0.971933	0.027648	1.107941
C	-0.135830	0.764766	1.302195
C	1.652055	-1.416990	-2.373544
C	2.724420	-1.333785	0.148225
C	3.534583	-0.498113	-0.902010
C	3.019566	-0.733031	-2.330914
C	4.961289	-0.990592	-0.712025
C	4.508778	-0.171096	1.444846
C	3.183379	-0.927511	1.556260
H	1.769576	-2.495128	-2.169638
H	-0.893142	-0.612251	-2.658930
H	3.746016	-1.338595	-2.888612
H	5.038368	-0.088966	2.400277
C	-0.213089	1.732265	2.458868
H	0.246358	2.699422	2.201710
H	-1.255158	1.919087	2.753173
H	0.321032	1.353162	3.339581
C	2.193083	0.164589	1.996581
H	1.933445	0.096011	3.062938
H	1.207962	-1.324804	-3.373453
H	2.942187	0.228648	-2.855606
O	5.667255	-1.516269	-1.529881
O	5.417480	-0.777038	0.533538
C	3.587135	1.024470	-0.531965
H	2.574510	1.434551	-0.654983
C	3.972732	1.181278	0.957108
H	4.677642	2.010521	1.102120
H	3.226465	-1.773033	2.250502
H	2.914172	-2.397004	-0.057250
C	4.545663	1.816452	-1.420344
H	4.471977	2.887558	-1.179886
H	5.593938	1.515925	-1.260461
H	4.322385	1.700712	-2.489994
O	2.871610	1.408914	1.804019
O	0.447325	-2.258052	0.459598
C	0.635557	-2.820769	1.733341
H	-0.147148	-3.581546	1.855292
H	1.617359	-3.314492	1.836526
H	0.517372	-2.079550	2.542856
C	-1.561415	-0.169909	-0.665164

H	-2.262229	0.773357	1.080161
O	-2.725515	-0.582874	-0.945358
C	-2.272104	3.736305	0.697272
H	-2.521390	4.654705	0.139491
H	-3.209405	3.328427	1.106144
H	-1.612564	4.015671	1.530330
C	-2.561318	2.372066	-2.015636
H	-2.675269	3.378016	-2.453390
H	-2.126441	1.721347	-2.789365
H	-3.566432	2.002244	-1.763927
C	0.260636	3.010482	-0.968184
H	0.686617	2.289883	-1.683578
H	0.242534	3.998599	-1.457550
H	0.926341	3.074088	-0.094382
Si	-1.483174	2.534251	-0.495974
Al	-4.287397	-0.886963	-0.040506
Cl	-3.686952	-1.954500	1.717462
Cl	-5.486964	-2.022204	-1.390099
Cl	-5.165524	1.012826	0.443762

63

27a-Al_7 Eopt -3181.608927

C	1.160997	1.591852	-0.842287
C	0.877013	-0.401377	0.714672
C	-0.428615	-0.713137	0.627824
C	-1.134599	-0.509231	-0.717366
C	-1.272629	0.957008	-1.114464
C	-0.264281	1.841413	-1.233890
C	-1.114945	-1.330580	1.820186
C	-2.584319	-1.057914	-0.767839
C	-3.290583	-1.152615	0.611326
C	-2.438100	-2.020714	1.523455
C	-4.632919	-1.786466	0.305030
C	-4.749611	0.131184	-1.042587
C	-3.380480	-0.113447	-1.681915
H	-0.416129	-2.025075	2.309251
H	1.460251	-0.723691	1.583427
H	-2.264120	-2.985519	1.021972
H	-5.468822	0.590910	-1.729418
C	-0.512750	3.215027	-1.809716
H	-1.291500	3.200706	-2.582334
H	-0.848192	3.918918	-1.031560
H	0.399336	3.625278	-2.264304
C	-2.691075	1.247625	-1.549267
H	-2.721175	1.829008	-2.480129
H	-1.277999	-0.518296	2.549798
H	-2.961008	-2.247107	2.462391
O	-5.063267	-2.817904	0.746134
O	-5.362208	-1.058692	-0.557848
C	-3.619486	0.262427	1.184823
H	-2.675946	0.787922	1.393841
C	-4.321825	1.088452	0.080783
H	-5.152909	1.678420	0.490249
H	-3.429751	-0.467696	-2.717583
H	-2.564243	-2.076687	-1.175480
C	-4.429271	0.205240	2.476290
H	-3.902250	-0.353375	3.263189
H	-4.607519	1.223657	2.852580
H	-5.413016	-0.268519	2.326508
O	-3.443848	1.977405	-0.571041
O	-0.339254	-1.078404	-1.754535
C	-0.060217	-2.456811	-1.689164
H	0.651072	-2.670727	-2.497007
H	0.407139	-2.749079	-0.733179
H	-0.958915	-3.077499	-1.846627
C	1.628951	0.324180	-0.295438
H	1.847412	1.912730	-1.642365

O	2.826906	-0.002132	-0.538360
C	0.174352	2.889552	1.820331
H	-0.752918	3.195548	1.311965
H	0.008957	1.904298	2.283344
H	0.372877	3.612357	2.629217
C	2.004579	4.530517	-0.093497
H	1.109085	5.065714	-0.437253
H	2.485565	5.144800	0.686412
H	2.711973	4.447673	-0.933813
C	3.185033	2.243101	1.503872
H	3.474021	2.991813	2.260396
H	3.055930	1.282131	2.023363
H	4.023108	2.146245	0.795668
Si	1.635522	2.855662	0.654590
Al	4.098090	-1.224705	-0.065911
Cl	4.036685	-1.517881	2.063767
Cl	3.617593	-3.014754	-1.136592
Cl	5.930971	-0.321888	-0.695511

63

27a-Al_8 Eopt -3181.605862

C	-1.322165	0.835813	0.419946
C	-0.546349	-0.694389	-1.484918
C	0.716598	-1.023020	-1.165621
C	1.215841	-1.056945	0.270404
C	1.022151	0.177454	1.132446
C	-0.072290	0.948801	1.241716
C	1.660118	-1.584926	-2.184304
C	2.741803	-1.292283	0.306158
C	3.540047	-0.523901	-0.799516
C	2.987134	-0.820826	-2.204084
C	4.956736	-1.037657	-0.599298
C	4.543087	-0.094427	1.519650
C	3.196915	-0.805564	1.685060
H	1.847711	-2.639490	-1.920615
H	-0.921680	-0.896128	-2.493103
H	3.727480	-1.393647	-2.778391
H	5.088482	0.028290	2.461709
C	-0.121525	2.049368	2.274384
H	0.339010	2.973929	1.892703
H	-1.156704	2.279525	2.561767
H	0.427150	1.775512	3.184981
C	2.231279	0.342166	2.036172
H	1.940210	0.344796	3.096009
H	1.201877	-1.579316	-3.182292
H	2.830150	0.124353	-2.741560
O	5.642884	-1.621914	-1.394142
O	5.425916	-0.774857	0.632602
C	3.632671	1.013596	-0.516298
H	2.627312	1.438813	-0.648828
C	4.040602	1.243720	0.957065
H	4.770672	2.058864	1.048649
H	3.203764	-1.604583	2.433967
H	2.947596	-2.362061	0.163866
C	4.594883	1.726994	-1.464877
H	5.638666	1.413645	-1.300306
H	4.353170	1.547344	-2.521867
H	4.548443	2.812624	-1.292298
O	2.952886	1.551259	1.794237
O	0.527157	-2.071857	0.999869
C	0.461826	-3.372044	0.468069
H	-0.067875	-3.983721	1.210517
H	-0.109128	-3.411588	-0.476426
H	1.458378	-3.817665	0.301682
C	-1.555636	-0.222785	-0.551218
H	-2.205079	0.944872	1.068727
O	-2.728136	-0.649498	-0.762160

C	-2.200696	3.843110	0.323992
H	-1.500946	4.225879	1.079466
H	-2.470669	4.680083	-0.341954
H	-3.120151	3.506203	0.826979
C	-2.629425	2.136733	-2.161816
H	-2.739416	3.070160	-2.738832
H	-2.257339	1.363334	-2.850363
H	-3.630231	1.844899	-1.808743
C	0.251873	2.875735	-1.327983
H	0.928966	3.102466	-0.490479
H	0.676032	2.040615	-1.906612
H	0.217176	3.759367	-1.986728
Si	-1.474712	2.483402	-0.731844
Al	-4.323450	-0.846417	0.107060
Cl	-3.856871	-2.111453	1.768403
Cl	-5.016426	1.089979	0.732514
Cl	-5.619688	-1.735290	-1.336894

63

27b-Al_1 Eopt -3181.614872

C	-1.185613	1.495024	-0.905822
C	-0.738633	-1.024187	-0.532438
C	0.537771	-1.054884	-0.078981
C	1.210506	0.197915	0.487707
C	1.294053	1.308298	-0.559224
C	0.236721	1.861760	-1.177475
C	1.227532	-2.398930	-0.080881
C	2.684878	-0.004589	0.934838
C	3.398381	-1.235994	0.315285
C	2.567630	-2.466060	0.637368
C	4.756548	-1.257274	0.987173
C	4.807817	0.863564	-0.019686
C	3.446737	1.272861	0.547855
H	0.529984	-3.138076	0.341825
H	-1.200032	-1.976713	-0.809928
H	2.418678	-2.510064	1.727230
H	5.510153	1.700661	-0.098748
C	0.375975	2.974745	-2.180350
H	1.401809	3.346099	-2.283651
H	0.049749	2.613041	-3.169084
H	-0.279873	3.822210	-1.923575
C	2.711397	1.820799	-0.677915
H	2.732426	2.918025	-0.728071
H	1.352414	-2.691411	-1.137456
H	3.091798	-3.389715	0.358091
O	5.218545	-2.139678	1.658905
O	5.460043	-0.134652	0.758985
C	3.693718	-1.035351	-1.204792
H	2.740518	-0.988002	-1.751406
C	4.350233	0.351245	-1.394922
H	5.159955	0.311862	-2.136003
H	3.505819	1.995736	1.369401
H	2.700746	-0.152292	2.022138
C	4.527732	-2.165189	-1.801219
H	5.525262	-2.233254	-1.337728
H	4.034636	-3.141025	-1.684969
H	4.676008	-1.992629	-2.877622
O	3.426927	1.328567	-1.816684
O	0.439002	0.707160	1.568330
C	0.147195	-0.173628	2.629168
H	-0.327110	0.427465	3.415914
H	-0.551331	-0.973054	2.327190
H	1.052475	-0.639156	3.054293
C	-1.605174	0.111474	-0.698349
H	-1.834010	1.854374	-1.722024
O	-2.859757	-0.092175	-0.756445
C	-0.570279	3.703777	1.259576

H	-1.008232	4.381501	2.011812
H	0.186821	3.073552	1.746641
H	-0.078649	4.325042	0.494690
C	-2.826180	1.635221	1.831211
H	-2.308334	0.705644	2.100889
H	-2.926463	2.243169	2.745922
H	-3.841654	1.377947	1.492852
C	-3.192537	3.736365	-0.341506
H	-3.983543	3.119681	-0.798599
H	-3.672135	4.419088	0.379784
H	-2.729985	4.349526	-1.131606
Si	-1.937945	2.655124	0.536476
Al	-4.054963	-1.376233	-0.261517
Cl	-3.373019	-2.202801	1.599449
Cl	-4.061338	-2.833234	-1.831985
Cl	-5.900859	-0.310757	-0.077677

63

27b-Al_2 Eopt -3181.612076

C	-1.142180	1.353462	-0.904807
C	-0.752709	-0.883655	0.349492
C	0.487114	-0.727289	0.864265
C	1.378133	0.502133	0.773655
C	1.362987	1.292922	-0.525168
C	0.284756	1.677516	-1.227851
C	1.081234	-1.819830	1.694380
C	2.873051	0.088465	0.940957
C	3.216949	-1.329984	0.379773
C	2.328415	-2.405741	1.022996
C	4.684030	-1.511777	0.743308
C	4.818504	0.422954	-0.579814
C	3.700557	1.136895	0.182788
H	1.351925	-1.373862	2.665392
H	-1.257688	-1.827994	0.567124
H	2.909408	-2.967002	1.766363
H	5.600583	1.105023	-0.931208
C	0.413220	2.479708	-2.496069
H	-0.346179	3.275657	-2.536029
H	1.401950	2.933789	-2.627065
H	0.229575	1.822106	-3.361668
C	2.779086	1.636293	-0.942122
H	2.897591	2.711282	-1.141223
H	0.346057	-2.611059	1.887663
H	2.006253	-3.127396	0.260806
O	5.161652	-2.372770	1.431669
O	5.472407	-0.572019	0.197033
C	3.185448	-1.359274	-1.187339
H	2.140419	-1.231864	-1.509463
C	3.989694	-0.154584	-1.734816
H	4.594307	-0.444034	-2.604467
H	4.064046	1.941252	0.831199
H	3.110138	0.076805	2.014026
C	3.719489	-2.669226	-1.764627
H	3.594646	-2.670127	-2.857601
H	4.794194	-2.798375	-1.557134
H	3.192107	-3.548551	-1.369705
O	3.188267	0.935628	-2.120426
O	0.919843	1.275843	1.878459
C	1.651927	2.423243	2.233485
H	1.034448	2.981079	2.950630
H	2.608098	2.172570	2.723908
H	1.856670	3.076687	1.368219
C	-1.551774	0.046221	-0.408460
H	-1.764319	1.535572	-1.794610
O	-2.762000	-0.265043	-0.639801
C	-2.244473	1.946367	2.007579
H	-2.697786	2.703915	2.668413

H	-2.915050	1.072314	1.992454
H	-1.278163	1.636446	2.430699
C	-3.698063	2.988992	-0.488724
H	-4.286834	3.679386	0.137953
H	-3.589493	3.442853	-1.486936
H	-4.271740	2.055049	-0.595354
C	-0.991193	4.225392	0.308287
H	-1.468209	4.965216	0.972892
H	0.026277	4.042637	0.680838
H	-0.919993	4.673452	-0.694783
Si	-2.032143	2.674379	0.301830
Al	-4.042467	-1.488575	-0.207615
Cl	-3.457251	-3.316117	-1.157065
Cl	-4.063274	-1.648899	1.930669
Cl	-5.846989	-0.656219	-0.997158

63

27b-Al_3 Eopt -3181.611221

C	-1.124113	1.374350	-0.896614
C	-0.782948	-0.939582	0.221908
C	0.471637	-0.845664	0.718071
C	1.343135	0.403194	0.755995
C	1.367060	1.273085	-0.487824
C	0.307349	1.692185	-1.194683
C	1.094671	-2.048427	1.359386
C	2.830797	0.010677	0.951554
C	3.263737	-1.303675	0.220848
C	2.356886	-2.483280	0.607176
C	4.698596	-1.490136	0.690814
C	4.860957	0.619463	-0.339799
C	3.643114	1.185578	0.396610
H	1.360144	-1.785166	2.395486
H	-1.288262	-1.902695	0.332813
H	2.918084	-3.194710	1.227265
H	5.642593	1.365248	-0.520676
C	0.458420	2.600002	-2.386264
H	-0.250276	3.441537	-2.333342
H	1.471086	3.001914	-2.502091
H	0.214532	2.039507	-3.303752
C	2.781731	1.760206	-0.742005
H	2.816957	2.857850	-0.790379
H	0.378551	-2.878568	1.408233
H	2.047673	-3.023775	-0.297546
O	5.154290	-2.423241	1.295082
O	5.485109	-0.452533	0.358927
C	3.375901	-1.119547	-1.330536
H	2.359140	-0.980675	-1.728749
C	4.171226	0.170848	-1.637056
H	4.867439	0.022440	-2.473015
H	3.891361	1.926779	1.163789
H	3.027116	-0.143172	2.020925
C	4.013952	-2.325583	-2.018575
H	3.494289	-3.265156	-1.784769
H	3.982475	-2.189579	-3.109746
H	5.071776	-2.445257	-1.733464
O	3.351351	1.267503	-1.955096
O	0.910734	1.275344	1.798533
C	0.802481	0.781588	3.113360
H	1.752090	0.367998	3.493394
H	0.530233	1.639118	3.742981
H	0.014953	0.014251	3.212518
C	-1.573456	0.064491	-0.447705
H	-1.747176	1.610069	-1.772741
O	-2.797137	-0.194272	-0.672779
C	-3.413893	3.369747	-0.544661
H	-3.128218	3.867126	-1.485322
H	-4.115560	2.554353	-0.782614

H	-3.947181	4.103874	0.081824
C	-0.649415	4.026831	0.709432
H	-0.333396	4.552452	-0.204542
H	-1.086939	4.772790	1.394135
H	0.234035	3.583823	1.192538
C	-2.473965	1.867716	1.946377
H	-2.921478	2.629438	2.606669
H	-3.236533	1.095340	1.766696
H	-1.634785	1.405715	2.482317
Si	-1.923209	2.705384	0.370387
Al	-4.095197	-1.419771	-0.302563
Cl	-4.088222	-1.706179	1.822419
Cl	-3.549355	-3.199409	-1.362171
Cl	-5.891844	-0.512531	-1.021609

63

27b-Al_4 Eopt -3181.610879

C	1.328702	0.802985	0.530828
C	0.422311	-0.728664	-1.356265
C	-0.862459	-0.330363	-1.479153
C	-1.570620	0.766886	-0.699882
C	-1.195293	0.960808	0.761138
C	0.042409	1.015791	1.278789
C	-1.734893	-0.958914	-2.518647
C	-3.103943	0.484584	-0.659645
C	-3.479422	-1.032421	-0.652021
C	-2.873633	-1.752642	-1.866883
C	-5.001004	-1.023550	-0.697584
C	-4.635956	0.184749	1.282139
C	-3.633125	1.136432	0.626511
H	-2.148914	-0.142379	-3.132293
H	0.779779	-1.511639	-2.029988
H	-3.658383	-1.939514	-2.611578
H	-5.245072	0.669068	2.053239
C	0.283654	1.264769	2.744063
H	1.074431	2.018956	2.881762
H	-0.610427	1.592379	3.286089
H	0.646742	0.337807	3.217437
C	-2.446082	1.115081	1.603149
H	-2.405551	2.009259	2.241411
H	-1.150606	-1.608769	-3.182125
H	-2.484341	-2.733145	-1.562196
O	-5.705906	-1.510248	-1.539953
O	-5.546214	-0.387369	0.351650
C	-3.123481	-1.713280	0.714364
H	-2.026850	-1.752419	0.802672
C	-3.650265	-0.831732	1.873578
H	-4.084207	-1.446937	2.672995
H	-4.036376	2.141128	0.460444
H	-3.559794	0.922513	-1.558382
C	-3.673062	-3.134737	0.826211
H	-3.318786	-3.596414	1.759711
H	-4.774845	-3.145676	0.852532
H	-3.349890	-3.776550	-0.005158
O	-2.667226	-0.011506	2.457564
O	-1.268139	1.917958	-1.484638
C	-1.949292	3.115280	-1.200767
H	-1.436433	3.909059	-1.760763
H	-3.001854	3.085407	-1.530592
H	-1.920815	3.372451	-0.128148
C	1.450738	-0.259149	-0.459965
H	2.132976	0.629838	1.261919
O	2.582506	-0.809459	-0.622596
C	1.189367	3.912525	0.493092
H	1.538720	4.839910	0.009017
H	0.096896	3.867685	0.394556
H	1.440216	3.980281	1.563242

C	1.845471	2.401728	-2.162542
H	2.430083	1.574613	-2.597620
H	0.790759	2.282582	-2.448060
H	2.228087	3.341366	-2.595064
C	3.874703	2.510926	0.116218
H	4.053070	2.418888	1.198540
H	4.448534	1.726655	-0.400748
H	4.280748	3.482476	-0.213923
Si	2.053794	2.463092	-0.307650
Al	4.133844	-1.416448	0.093344
Cl	3.935658	-3.547223	0.068574
Cl	5.674307	-0.705470	-1.210057
Cl	4.307087	-0.668245	2.101372

63

27b-Al_5 Eopt -3181.609964

C	-1.201227	1.396780	-0.904351
C	-0.725723	-1.049082	-0.203914
C	0.557786	-1.029377	0.231377
C	1.285133	0.265259	0.592757
C	1.301750	1.270761	-0.562443
C	0.219456	1.769468	-1.188321
C	1.206978	-2.385310	0.398732
C	2.792281	0.066887	0.941461
C	3.422401	-1.247726	0.411344
C	2.614813	-2.404614	0.970612
C	4.842909	-1.235452	0.943242
C	4.838602	0.746858	-0.306013
C	3.555734	1.262772	0.348136
H	0.535997	-3.002381	1.015695
H	-1.185934	-2.033373	-0.334782
H	2.588876	-2.309282	2.066701
H	5.544359	1.547788	-0.552729
C	0.340124	2.773161	-2.305967
H	0.242189	2.249883	-3.271594
H	-0.471240	3.515065	-2.264147
H	1.297171	3.307842	-2.313852
C	2.717296	1.713879	-0.851598
H	2.776539	2.797167	-1.025533
H	1.199732	-2.860909	-0.596336
H	3.090104	-3.370492	0.753680
O	5.349862	-2.050537	1.665528
O	5.547759	-0.176329	0.511543
C	3.560247	-1.232100	-1.143728
H	2.555274	-1.215052	-1.591490
C	4.226364	0.101751	-1.558667
H	4.951906	-0.050770	-2.368947
H	3.726949	2.063030	1.077037
H	2.883601	0.028113	2.035116
C	4.302452	-2.449696	-1.685795
H	4.340616	-2.407089	-2.784460
H	5.341140	-2.497376	-1.320745
H	3.802999	-3.388247	-1.405503
O	3.292746	1.064420	-1.993189
O	0.554813	0.758549	1.710521
C	1.071851	1.890658	2.368130
H	1.368972	2.684928	1.663669
H	0.270250	2.279123	3.011169
H	1.935887	1.644727	3.008509
C	-1.597284	0.034742	-0.556019
H	-1.819379	1.642937	-1.784423
O	-2.845027	-0.204028	-0.646927
C	-3.762541	3.046792	-0.478635
H	-4.347707	3.724947	0.165033
H	-3.602708	3.550017	-1.445833
H	-4.363632	2.140271	-0.650123
C	-1.104076	4.162911	0.554890

H	-1.549648	4.785299	1.348991
H	-0.062199	3.954291	0.833244
H	-1.100082	4.759760	-0.370246
C	-2.482956	1.746807	1.978867
H	-3.394787	1.134836	1.907256
H	-1.652909	1.092223	2.279193
H	-2.646694	2.496659	2.770399
Si	-2.144192	2.619263	0.358276
Al	-4.051226	-1.480917	-0.162971
Cl	-3.679674	-1.936972	1.896443
Cl	-3.703168	-3.163988	-1.446155
Cl	-5.950479	-0.552868	-0.491265

63

27b-Al_6 Eopt -3181.612889

C	-1.335824	-0.761106	0.530180
C	-0.419962	1.346870	-0.666810
C	0.907486	1.094704	-0.790021
C	1.480583	-0.316927	-0.643388
C	1.171038	-0.925852	0.724320
C	-0.060338	-1.123125	1.223458
C	1.781548	2.270112	-1.163423
C	3.030046	-0.402667	-0.749341
C	3.778329	0.931880	-0.497403
C	3.237230	1.957887	-1.478264
C	5.235485	0.601286	-0.749319
C	4.744347	-0.913281	0.975215
C	3.492971	-1.449661	0.276400
H	1.308796	2.789557	-2.011171
H	-0.774784	2.350019	-0.920306
H	3.333827	1.549594	-2.495863
H	5.276319	-1.678957	1.550815
C	-0.300753	-1.781103	2.555632
H	-1.006853	-2.621270	2.453910
H	0.612441	-2.149754	3.035359
H	-0.772632	-1.056189	3.238537
C	2.438994	-1.416686	1.383953
H	2.291983	-2.396629	1.857690
H	1.726268	2.983577	-0.323762
H	3.827168	2.884049	-1.457771
O	5.959245	1.099470	-1.568801
O	5.694344	-0.359239	0.070832
C	3.701391	1.359890	1.001759
H	2.655493	1.589820	1.253107
C	4.081744	0.141540	1.875768
H	4.706977	0.441586	2.727442
H	3.621026	-2.445386	-0.162957
H	3.293280	-0.716779	-1.767100
C	4.547074	2.590830	1.312316
H	4.246586	3.455017	0.702455
H	4.426945	2.870604	2.369520
H	5.620302	2.410512	1.138591
O	2.952489	-0.529687	2.385756
O	0.879564	-1.164801	-1.614526
C	0.981881	-0.783043	-2.965897
H	0.552277	-1.602177	-3.557474
H	0.419747	0.141867	-3.184051
H	2.026966	-0.642184	-3.289722
C	-1.478759	0.495859	-0.197198
H	-2.159225	-0.755298	1.263319
O	-2.653913	0.945685	-0.389029
C	-0.716591	-3.658317	-0.571856
H	0.223286	-3.329719	-1.034752
H	-0.507761	-4.009307	0.451096
H	-1.119435	-4.512055	-1.142740
C	-2.438951	-1.689475	-2.288203
H	-3.305764	-1.012606	-2.246895

H	-1.613085	-1.168431	-2.790218
H	-2.723087	-2.557251	-2.906658
C	-3.538353	-2.892934	0.293510
H	-3.905498	-3.803527	-0.209475
H	-3.343842	-3.145267	1.348203
H	-4.347168	-2.147112	0.260186
Si	-1.990840	-2.292742	-0.576795
Al	-4.325900	1.207003	0.242208
Cl	-4.393095	0.371674	2.223218
Cl	-5.665352	0.221479	-1.108169
Cl	-4.592119	3.330351	0.258771

63

27b-Al_7 Eopt -3181.611496

C	-1.289307	-0.690464	0.584799
C	-0.433625	1.084306	-1.110702
C	0.859812	0.735974	-1.288167
C	1.528726	-0.524621	-0.761507
C	1.227530	-0.930158	0.671609
C	0.018670	-1.002559	1.248029
C	1.746828	1.599961	-2.133177
C	3.069267	-0.362704	-0.777401
C	3.581941	1.073800	-0.425069
C	2.930753	2.138403	-1.322782
C	5.081255	0.950826	-0.650661
C	4.764542	-0.693429	1.004340
C	3.598934	-1.354920	0.263061
H	2.125673	0.990423	-2.969253
H	-0.799667	1.980869	-1.618107
H	3.679854	2.552180	-2.010783
H	5.387611	-1.405944	1.555738
C	-0.162127	-1.481114	2.664000
H	-0.926651	-2.273006	2.715442
H	0.761791	-1.854172	3.119492
H	-0.531249	-0.648773	3.285350
C	2.497837	-1.429827	1.335867
H	2.359891	-2.445478	1.732733
H	1.177945	2.431228	-2.568782
H	2.570751	2.971148	-0.703834
O	5.757306	1.572403	-1.425344
O	5.642041	0.017403	0.136759
C	3.435117	1.411426	1.096803
H	2.361779	1.516881	1.317245
C	3.964983	0.227772	1.938211
H	4.541493	0.579091	2.804120
H	3.843290	-2.330694	-0.170432
H	3.446327	-0.593852	-1.782386
C	4.146594	2.708909	1.478541
H	3.934076	2.952947	2.529938
H	5.240455	2.618901	1.377536
H	3.821512	3.561704	0.866362
O	2.945626	-0.612992	2.417976
O	1.130385	-1.646678	-1.547505
C	1.277622	-1.606588	-2.947026
H	2.322940	-1.446009	-3.261794
H	0.957463	-2.589562	-3.317823
H	0.641805	-0.835749	-3.416342
C	-1.454314	0.442998	-0.316411
H	-2.070680	-0.576053	1.352189
O	-2.611276	0.947209	-0.448716
C	-3.606150	-2.737529	0.542846
H	-4.385486	-1.983242	0.358240
H	-3.980770	-3.701469	0.158311
H	-3.469134	-2.838834	1.631020
C	-0.788675	-3.733911	-0.085691
H	-0.633419	-3.961025	0.980555
H	-1.208324	-4.635688	-0.563090

H	0.180290	-3.503848	-0.548897
C	-2.318091	-1.943290	-2.124952
H	-3.099722	-1.174765	-2.230351
H	-1.412137	-1.600481	-2.644256
H	-2.678321	-2.854705	-2.630429
Si	-1.998126	-2.332150	-0.324784
Al	-4.270121	1.279587	0.194821
Cl	-5.609524	0.212357	-1.091820
Cl	-4.340699	0.598094	2.231070
Cl	-4.500097	3.401026	0.054507

63

28a-Al_1 Eopt -3181.605992

C	-1.388493	-0.219060	-0.048960
C	-0.042603	1.330332	1.516260
C	1.210434	1.317189	1.056052
C	1.571561	0.891618	-0.352990
C	0.963854	-0.412799	-0.868061
C	-0.300121	-0.890808	-0.769131
C	2.354186	1.914795	1.826400
C	3.103870	0.649926	-0.476323
C	3.771661	0.051692	0.810758
C	3.490865	0.918694	2.049295
C	5.250938	0.021140	0.451570
C	4.382510	-1.350384	-1.246629
C	3.301686	-0.337733	-1.634747
H	2.727356	2.779430	1.250586
H	-0.208761	1.767733	2.505941
H	4.407150	1.451600	2.336817
H	4.768260	-1.913973	-2.103220
O	-2.534519	-0.722003	-0.241572
C	-0.717562	-2.179635	-1.434971
H	-1.310699	-2.795843	-0.745654
H	-1.362288	-1.969095	-2.303023
H	0.140502	-2.773088	-1.761816
C	2.021764	-1.186429	-1.647306
H	1.664878	-1.417138	-2.661679
H	2.001692	2.306434	2.790553
H	3.224529	0.269699	2.894505
O	6.150999	0.583210	1.014043
O	5.505885	-0.743557	-0.624769
C	3.386521	-1.453025	1.030885
H	2.320399	-1.493300	1.303931
C	3.560714	-2.227481	-0.294655
H	3.993544	-3.222018	-0.126944
H	3.501636	0.153878	-2.591884
H	3.583841	1.619246	-0.672940
C	4.199251	-2.112902	2.144524
H	5.265899	-2.192294	1.878944
H	4.129064	-1.567250	3.095347
H	3.828176	-3.133826	2.317968
O	2.354041	-2.411582	-1.002625
O	1.137626	1.986901	-1.153507
C	1.434144	2.012228	-2.528435
H	0.895206	2.873916	-2.945307
H	2.509856	2.158173	-2.723259
H	1.090768	1.104853	-3.056826
C	-1.300648	0.937919	0.828372
H	-2.109860	0.817773	1.565780
Si	-2.063677	2.504459	-0.174950
C	-0.984452	3.993184	0.142386
H	0.037493	3.829323	-0.225523
H	-1.420550	4.859361	-0.383581
H	-0.944903	4.236759	1.215814
C	-2.215022	2.061216	-1.986185
H	-2.546401	2.949472	-2.549294
H	-1.262918	1.719831	-2.415905

H	-2.970948	1.273794	-2.132496
C	-3.764324	2.802703	0.547512
H	-4.473103	1.995993	0.308980
H	-3.728977	2.927088	1.641313
H	-4.168481	3.733819	0.114650
Al	-4.187809	-1.206848	0.301475
Cl	-5.529820	-0.308722	-1.103957
Cl	-4.200321	-3.345988	0.211240
Cl	-4.440803	-0.490101	2.309308

63

28a-Al_2 Eopt -3181.606070

C	-1.432501	-0.294052	-0.019071
C	-0.043803	1.258265	1.475873
C	1.224669	1.125735	1.068668
C	1.523162	0.663765	-0.358408
C	0.936699	-0.719540	-0.653472
C	-0.346320	-1.137781	-0.525308
C	2.331681	1.462187	2.058493
C	3.037881	0.506793	-0.682930
C	3.951365	0.311157	0.555535
C	3.739748	1.494799	1.484164
C	5.357316	0.269096	-0.010518
C	4.388088	-1.536312	-1.149370
C	3.183579	-0.710844	-1.610389
H	2.101399	2.430072	2.528470
H	-0.203987	1.682359	2.472959
H	3.904527	2.417903	0.906713
H	4.696515	-2.288882	-1.883376
O	-2.601766	-0.716392	-0.272268
C	-0.770295	-2.524077	-0.947375
H	-1.207606	-2.509644	-1.958777
H	0.072930	-3.222981	-0.936605
H	-1.542891	-2.918082	-0.274821
C	2.000904	-1.616682	-1.262214
H	1.607580	-2.164582	-2.129796
H	2.298613	0.721176	2.873733
H	4.478952	1.499987	2.296944
O	6.263572	1.013805	0.247365
O	5.534094	-0.739369	-0.883033
C	3.727606	-1.085692	1.218171
H	2.707114	-1.123768	1.627276
C	3.780436	-2.164703	0.112871
H	4.308916	-3.064875	0.452934
H	3.220679	-0.442531	-2.671864
H	3.371265	1.433577	-1.170261
C	4.706754	-1.367091	2.353885
H	5.750763	-1.396266	2.002057
H	4.637605	-0.607192	3.145320
H	4.483910	-2.344021	2.808022
O	2.494322	-2.566210	-0.317442
O	0.927557	1.642141	-1.202014
C	1.077754	1.501687	-2.593876
H	0.497247	2.311768	-3.054638
H	2.125430	1.613189	-2.919620
H	0.689261	0.536828	-2.966443
C	-1.306594	0.924886	0.769782
H	-2.129616	0.868222	1.502179
Si	-2.020636	2.491492	-0.253698
C	-3.637044	2.948356	0.577794
H	-4.015541	3.880563	0.124633
H	-4.413214	2.179802	0.446401
H	-3.504641	3.128907	1.656673
C	-0.843932	3.930272	-0.075671
H	-1.261953	4.792906	-0.621824
H	-0.740320	4.222340	0.981589
H	0.149927	3.696078	-0.478294

C	-2.363578	1.979942	-2.021000
H	-1.533373	1.428305	-2.481316
H	-3.264182	1.348734	-2.065925
H	-2.556150	2.880350	-2.627847
Al	-4.287316	-1.082081	0.271596
Cl	-4.458031	-0.346228	2.282814
Cl	-5.599348	-0.098343	-1.104220
Cl	-4.471376	-3.214069	0.189751

63

28b-Al_1 Eopt -3181.611440

C	-1.429553	-0.100240	0.244220
C	-0.044289	1.050827	-1.611105
C	1.066699	0.305325	-1.667809
C	1.359997	-0.926449	-0.835923
C	0.859745	-0.947870	0.602326
C	-0.350775	-0.617863	1.095942
C	2.184040	0.643741	-2.617983
C	2.886674	-1.135820	-0.698897
C	3.721924	0.176616	-0.624778
C	3.434237	1.076255	-1.842000
C	5.159849	-0.319945	-0.596607
C	4.311946	-1.397237	1.315464
C	3.091879	-1.958254	0.579734
H	2.410414	-0.243092	-3.231720
H	-0.107104	1.886121	-2.316216
H	4.307751	1.077592	-2.508120
H	4.691869	-2.065778	2.095856
O	-2.590487	-0.498126	0.546967
C	-0.738397	-0.882573	2.530949
H	-1.167443	-1.891382	2.639925
H	0.132945	-0.797817	3.190363
H	-1.498636	-0.167584	2.873245
C	1.925467	-1.562869	1.495845

H	1.525459	-2.399217	2.086083
H	1.878713	1.442262	-3.308237
H	3.291835	2.112642	-1.504804
O	6.037326	-0.063340	-1.375772
O	5.406356	-1.128800	0.449132
C	3.524698	0.902593	0.748530
H	2.491697	1.278167	0.782800
C	3.678609	-0.124789	1.895225
H	4.238517	0.298010	2.739362
H	3.142003	-3.034958	0.387456
H	3.213367	-1.690032	-1.588135
C	4.476193	2.081288	0.941482
H	4.412809	2.806446	0.118064
H	4.228497	2.611422	1.873221
H	5.524899	1.751020	1.019437
O	2.446527	-0.591561	2.402461
O	0.938320	-2.117947	-1.501490
C	-0.433489	-2.312305	-1.729549
H	-0.523233	-3.197468	-2.373652
H	-0.990148	-2.518511	-0.798915
H	-0.900902	-1.461160	-2.254627
C	-1.255576	0.931861	-0.756502
H	-2.169058	1.013392	-1.367652
Si	-1.385888	2.633894	0.288841
C	-2.830186	2.520504	1.466455
H	-2.708677	1.737354	2.229973
H	-2.920755	3.485710	1.992540
H	-3.775168	2.346517	0.929123
C	0.231508	2.881905	1.189456
H	0.461359	2.046419	1.868733
H	1.066738	3.002077	0.482862
H	0.169347	3.801248	1.795382
C	-1.685868	3.950299	-1.001976

H	-2.590276	3.731412	-1.591140
H	-1.840583	4.916521	-0.493307
H	-0.835531	4.066489	-1.690776
Al	-4.291561	-0.746599	-0.049834
Cl	-4.854103	0.857552	-1.367821
Cl	-4.188147	-2.617378	-1.087713
Cl	-5.472120	-0.788665	1.727389

63

28b-Al_2 Eopt -3181.608431

C	-1.443910	0.066173	0.381006
C	-0.063827	1.292752	-1.411169
C	1.121316	0.674681	-1.307633
C	1.256556	-0.574680	-0.432958
C	0.866372	-0.328394	1.023440
C	-0.391794	-0.058426	1.409011
C	2.282002	1.200354	-2.140198
C	2.692037	-1.159473	-0.333839
C	3.818222	-0.168382	-0.730691
C	3.529629	0.325510	-2.138719
C	5.089622	-0.985936	-0.638933
C	4.297849	-1.175372	1.565563
C	2.900755	-1.616135	1.122552
H	1.936599	1.347571	-3.174910
H	-0.124438	2.126546	-2.118509
H	3.391216	-0.551638	-2.790300
H	4.621016	-1.647939	2.499779
O	-2.520098	-0.545073	0.627119
C	-0.867714	-0.029124	2.837026
H	-1.377883	-0.970179	3.094865
H	-0.034491	0.125573	3.532342
H	-1.590603	0.784918	2.992341
C	1.982934	-0.723038	1.961132
H	1.594909	-1.221504	2.859678
H	2.547347	2.203970	-1.769838
H	4.382585	0.884305	-2.547800
O	5.868672	-1.219785	-1.522849
O	5.303055	-1.465373	0.600457
C	3.986153	0.970599	0.324157
H	3.081431	1.595760	0.319052
C	4.036182	0.330920	1.727560
H	4.767939	0.833695	2.374004
H	2.704080	-2.684502	1.265774
H	2.764598	-2.012187	-1.022153
C	5.188219	1.867086	0.043427
H	5.122056	2.334594	-0.949523
H	5.237275	2.673709	0.790044
H	6.139318	1.312736	0.093517
O	2.783380	0.383366	2.379047
O	0.339554	-1.580981	-0.861380
C	0.354060	-1.941834	-2.220264
H	0.013451	-1.120413	-2.873497
H	1.352739	-2.269374	-2.559817
H	-0.342178	-2.784034	-2.326069
C	-1.352322	0.992769	-0.729755
H	-2.156106	0.795353	-1.458312
Si	-1.975973	2.728297	0.039100
C	-3.528639	2.447628	1.038971
H	-3.845166	3.415735	1.462220
H	-4.349625	2.080432	0.403808
H	-3.395189	1.747098	1.876735
C	-0.584294	3.422070	1.076657
H	-0.879057	4.411616	1.464113
H	-0.347036	2.780748	1.939553
H	0.334232	3.552106	0.483046
C	-2.348337	3.798983	-1.446698
H	-2.784113	4.749166	-1.095024

H	-1.449725	4.041296	-2.034045	H	-4.753679	1.518317	1.708931
H	-3.084139	3.316975	-2.109561	O	-5.899825	-0.978349	1.357914
Al	-4.086205	-1.172356	-0.059387	O	-4.940328	-1.917998	-0.398191
Cl	-3.634619	-3.171588	-0.673320	C	-3.847324	0.595535	-0.890262
Cl	-4.704586	0.041816	-1.726219	H	-2.996714	1.285026	-0.994186
Cl	-5.459012	-1.055950	1.572839	C	-3.607535	-0.516262	-1.933814
63				H	-4.243376	-0.382385	-2.819096
28b-Al_3			Eopt -3181.606568	H	-2.188956	-2.955467	-0.168203
C	1.541417	0.441212	0.152657	H	-2.710379	-1.506909	1.655994
C	-0.210567	2.046037	1.142418	C	-5.130374	1.378880	-1.151141
C	-1.315299	1.285370	1.123322	H	-6.023493	0.734108	-1.119455
C	-1.211825	-0.206199	0.787810	H	-5.270039	2.185199	-0.416796
C	-0.589270	-0.453429	-0.582037	H	-5.091891	1.839340	-2.149616
C	0.688516	-0.170492	-0.882917	O	-2.265316	-0.551961	-2.374111
C	-2.638510	1.926747	1.517330	O	-0.323353	-0.858285	1.692842
C	-2.566352	-0.963642	0.712886	C	-0.573625	-0.709824	3.068038
C	-3.804958	-0.046176	0.532858	H	-1.583026	-1.055197	3.353691
C	-3.807497	0.961182	1.671119	H	0.163545	-1.334830	3.589316
C	-4.986707	-0.992354	0.577477	H	-0.451135	0.334007	3.404440
C	-3.797787	-1.877813	-1.245702	C	1.197441	1.680112	0.824819
C	-2.478177	-1.943545	-0.472924	H	1.858377	1.812316	1.699138
H	-2.495187	2.483385	2.456142	Si	1.920474	3.098113	-0.375427
H	-0.334639	3.081746	1.475288	C	1.736810	4.689574	0.589996
H	-3.734112	0.408087	2.620769	H	2.274515	5.488301	0.052176
H	-3.916099	-2.697297	-1.963361	H	0.686882	5.003970	0.689905
O	2.692917	-0.031022	0.352458	H	2.177172	4.607128	1.596342
C	1.342806	-0.573392	-2.180914	C	3.722798	2.726014	-0.688914
H	2.268330	-0.010105	-2.360161	H	3.881870	1.790577	-1.245604
H	1.599557	-1.644328	-2.181167	H	4.159720	3.547740	-1.280679
H	0.661723	-0.385114	-3.021144	H	4.283785	2.657575	0.256935
C	-1.484059	-1.307390	-1.448372	C	0.901337	3.122153	-1.942205
H	-0.898544	-2.042082	-2.018323	H	1.126308	2.267479	-2.597937
H	-2.896411	2.683814	0.759188	H	-0.177744	3.114070	-1.719763

H	1.122420	4.043076	-2.507120
Al	3.868686	-1.404298	0.156842
Cl	2.738875	-3.215900	-0.021188
Cl	5.037279	-1.341501	1.947530
Cl	5.030985	-0.962722	-1.589581

63

28b-Al_4 Eopt -3181.606965

C	-1.458494	-0.065350	0.218884
C	-0.063220	1.162924	-1.574136
C	1.063884	0.444337	-1.603073
C	1.305821	-0.810789	-0.787131
C	0.840804	-0.797257	0.667086
C	-0.385132	-0.493923	1.128459
C	2.152614	0.719857	-2.600726
C	2.833253	-1.109528	-0.672561
C	3.736741	0.171142	-0.675080
C	3.489970	1.029033	-1.926453
C	5.152931	-0.385121	-0.636826
C	4.294265	-1.307330	1.344347
C	3.042820	-1.857220	0.655549
H	2.254070	-0.180375	-3.230257
H	-0.135873	1.993128	-2.283745
H	4.312102	0.876428	-2.638515
H	4.653369	-1.949882	2.155905
O	-2.598767	-0.542952	0.473484
C	-0.795156	-0.696992	2.565710
H	-1.236825	-1.696294	2.707081
H	0.066138	-0.593423	3.235812
H	-1.552999	0.038437	2.868751
C	1.911617	-1.369901	1.573779
H	1.504338	-2.161482	2.219220
H	1.866367	1.544010	-3.268552
H	3.508392	2.091980	-1.650574
O	6.025265	-0.215193	-1.445008
O	5.386366	-1.136084	0.453260
C	3.590997	0.982356	0.659723
H	2.575781	1.407445	0.688012
C	3.721851	0.018691	1.861694
H	4.312719	0.465675	2.671574
H	3.057760	-2.945634	0.535442
H	3.126807	-1.720706	-1.537867
C	4.600367	2.123144	0.779772
H	4.393117	2.704958	1.690207
H	5.633432	1.747250	0.858628
H	4.558337	2.814399	-0.072935
O	2.481001	-0.366382	2.411617
O	0.607828	-1.808287	-1.522577
C	0.649888	-3.136273	-1.063927
H	0.334323	-3.229787	-0.009081
H	-0.061655	-3.703204	-1.679280
H	1.648080	-3.592733	-1.181092
C	-1.301269	0.980826	-0.768766
H	-2.190870	1.015482	-1.418375
Si	-1.542694	2.668174	0.275254
C	-3.053925	2.506740	1.360450
H	-3.187468	3.452797	1.911449
H	-3.960112	2.345467	0.756154
H	-2.980765	1.698574	2.103345
C	0.018933	2.957420	1.260265
H	-0.077967	3.900386	1.823882
H	0.215579	2.151852	1.984166
H	0.892742	3.050397	0.596682
C	-1.809876	3.985439	-1.022403
H	-2.656504	3.730600	-1.679330
H	-2.049745	4.936027	-0.517263
H	-0.917688	4.152120	-1.644580

Al	-4.294838	-0.837338	-0.122653
Cl	-4.856869	0.688613	-1.530725
Cl	-4.178514	-2.766825	-1.038735
Cl	-5.489677	-0.779148	1.646638

63

28b-Al_5 Eopt -3181.604614

C	1.523810	0.371746	0.265305
C	-0.239160	1.753199	1.559057
C	-1.285054	0.919236	1.600521
C	-1.278488	-0.504597	1.072088
C	-0.589638	-0.760198	-0.260308
C	0.648537	-0.391157	-0.635314
C	-2.580006	1.325543	2.253513
C	-2.731532	-0.992540	0.821720
C	-3.709860	0.115513	0.304767
C	-3.730811	1.333376	1.245283
C	-5.049253	-0.604193	0.255835
C	-3.797497	-1.953182	-1.209108
C	-2.628921	-2.105358	-0.230280
H	-2.810440	0.618149	3.066239
H	-0.369484	2.729804	2.036636
H	-4.692947	1.369716	1.774058
H	-3.966625	-2.845392	-1.821909
O	2.748593	0.076460	0.304173
C	1.255774	-0.850908	-1.937921
H	1.694996	-1.856315	-1.836776
H	0.486114	-0.890860	-2.718109
H	2.049532	-0.170589	-2.274491
C	-1.411958	-1.728476	-1.091018
H	-0.802297	-2.592726	-1.390475
H	-2.480764	2.317515	2.715800
H	-3.662363	2.253747	0.649098
O	-6.055107	-0.318153	0.847272
O	-5.031592	-1.671402	-0.561768
C	-3.415708	0.510428	-1.182534
H	-2.446054	1.031858	-1.206987
C	-3.270003	-0.770634	-2.034467
H	-3.760541	-0.663544	-3.010536
H	-2.546195	-3.103051	0.213347
H	-3.145449	-1.369576	1.766622
C	-4.480217	1.433314	-1.773521
H	-4.631810	2.339503	-1.170892
H	-4.176827	1.750546	-2.782332
H	-5.453457	0.924823	-1.868272
O	-1.927335	-1.126874	-2.274894
O	-0.591117	-1.397787	1.944589
C	-0.924264	-1.393297	3.310295
H	-1.987667	-1.629500	3.493340
H	-0.315313	-2.176683	3.781109
H	-0.684875	-0.430432	3.794061
C	1.114899	1.567462	0.975473
H	1.901068	1.839356	1.699227
Si	1.353042	3.010689	-0.385112
C	0.009137	2.828915	-1.670826
H	0.097178	1.895377	-2.247038
H	-0.988443	2.860085	-1.205141
H	0.073445	3.669605	-2.381799
C	1.174215	4.604764	0.574392
H	1.426878	5.447930	-0.090072
H	0.146988	4.760702	0.937241
H	1.860985	4.637383	1.434992
C	3.071200	2.850324	-1.096790
H	3.224938	1.914711	-1.654961
H	3.250826	3.688411	-1.790695
H	3.834135	2.901436	-0.303446
Al	4.166122	-1.024476	0.037147

Cl	3.423853	-3.027836	0.194359
Cl	5.523075	-0.522752	1.613533
Cl	4.969164	-0.561593	-1.894430
63			
28b-Al_6		Eopt	-3181.606416
C	-1.477418	-0.013644	0.342576
C	-0.137519	0.959980	-1.644730
C	0.992990	0.245904	-1.604398
C	1.255549	-0.892083	-0.635536
C	0.826013	-0.709553	0.813377
C	-0.386804	-0.345015	1.267854
C	2.101816	0.478370	-2.596259
C	2.779132	-1.169158	-0.527636
C	3.678393	0.109641	-0.616438
C	3.391822	0.913007	-1.897676
C	5.089548	-0.458071	-0.588958
C	4.271370	-1.276489	1.459231
C	2.991260	-1.841287	0.835815
H	2.284173	-0.454593	-3.153676
H	-0.220213	1.711391	-2.436920
H	4.241829	0.816513	-2.586719
H	4.649054	-1.880308	2.291710
O	-2.618329	-0.438015	0.684367
C	-0.761975	-0.443278	2.725813
H	-1.580973	0.243359	2.976571
H	-1.104564	-1.461766	2.969804
H	0.098844	-0.203080	3.361546
C	1.889737	-1.265964	1.740937
H	1.458405	-2.007405	2.427930
H	1.802666	1.234676	-3.335179
H	3.307442	1.979481	-1.647653
O	5.943229	-0.337037	-1.425575
O	5.339717	-1.163582	0.528122
C	3.576581	0.986990	0.678134
H	2.563714	1.417771	0.712234
C	3.734415	0.085205	1.923421
H	4.356621	0.563178	2.691072
H	2.969127	-2.934569	0.778092
H	3.076908	-1.830240	-1.352921
C	4.593201	2.127149	0.704579
H	5.626658	1.750365	0.773222
H	4.527786	2.768464	-0.185152
H	4.416097	2.760948	1.586247
O	2.504084	-0.248123	2.526375
O	0.551779	-2.077440	-1.000892
C	0.635224	-2.529283	-2.329434
H	0.043456	-3.453089	-2.379611
H	0.205351	-1.805495	-3.043110
H	1.669638	-2.764303	-2.637077
C	-1.345031	0.891617	-0.780849
H	-2.261134	0.873153	-1.392605
Si	-1.505097	2.699877	0.066054
C	-2.902027	2.677750	1.304261
H	-3.047868	3.704089	1.680771
H	-3.846061	2.353680	0.840114
H	-2.695402	2.032743	2.171356
C	0.132488	3.101893	0.872291
H	0.065053	4.098094	1.340792
H	0.395972	2.378510	1.659557
H	0.948809	3.132122	0.134445
C	-1.895035	3.846538	-1.355997
H	-1.074568	3.905750	-2.087043
H	-2.812473	3.534223	-1.879080
H	-2.062900	4.860878	-0.956984
Al	-4.253318	-0.873511	0.000277
Cl	-5.477153	-1.182172	1.721198

Cl	-4.984248	0.728874	-1.236004
Cl	-3.868198	-2.636989	-1.149403

63

28b-Al_7 Eopt -3181.609672

C	1.485671	0.359717	0.268188
C	-0.294942	1.662583	1.620852
C	-1.307910	0.790975	1.709721
C	-1.331987	-0.617847	1.151222
C	-0.609605	-0.869566	-0.165336
C	0.617014	-0.479382	-0.568170
C	-2.589767	1.156529	2.408505
C	-2.787631	-1.058573	0.862087
C	-3.732053	0.084063	0.384582
C	-3.739685	1.244680	1.397963
C	-5.088195	-0.597008	0.273856
C	-3.837568	-1.922100	-1.213849
C	-2.699401	-2.147808	-0.214778
H	-2.812723	0.389707	3.167816
H	-0.437781	2.627475	2.118550
H	-4.703433	1.261276	1.924840
H	-4.015988	-2.782545	-1.868152
O	2.727857	0.141677	0.255651
C	1.210134	-0.952983	-1.873916
H	1.642905	-1.960491	-1.769484
H	0.434905	-0.990986	-2.648389
H	2.006773	-0.282300	-2.221414
C	-1.449173	-1.795217	-1.032202
H	-0.873070	-2.672046	-1.359830
H	-2.484577	2.113132	2.938546
H	-3.655365	2.199752	0.860713
O	-6.101912	-0.304428	0.848111
O	-5.078480	-1.631390	-0.584996
C	-3.387875	0.530558	-1.076165
H	-2.401729	1.017032	-1.052046
C	-3.255191	-0.722752	-1.974204
H	-3.714768	-0.562036	-2.958013
H	-2.667328	-3.158180	0.205687
H	-3.192096	-1.456737	1.801402
C	-4.401497	1.513442	-1.656919
H	-5.389552	1.045642	-1.797535
H	-4.536625	2.398365	-1.019399
H	-4.059383	1.861494	-2.643029
O	-1.917079	-1.114211	-2.196129
O	-0.902883	-1.573022	2.121212
C	0.420692	-1.514430	2.585819
H	1.152787	-1.824946	1.819517
H	0.690912	-0.514900	2.968154
H	0.491590	-2.226908	3.418871
C	1.038479	1.544201	0.974044
H	1.838191	1.864024	1.662639
Si	1.162263	2.991154	-0.398196
C	2.847585	2.883459	-1.193599
H	2.990405	1.965129	-1.782722
H	2.974408	3.741669	-1.874496
H	3.648183	2.932058	-0.438274
C	-0.233332	2.766403	-1.619071
H	-0.216127	1.779427	-2.106309
H	-1.209392	2.899540	-1.127298
H	-0.149965	3.532514	-2.407960
C	0.977758	4.578875	0.570338
H	-0.029273	4.689020	1.000742
H	1.717963	4.645165	1.383381
H	1.146871	5.430700	-0.109428
Al	4.186812	-0.900980	-0.007818
Cl	4.909799	-0.491341	-1.980945
Cl	3.541188	-2.930885	0.255164

Cl	5.559831	-0.277798	1.509586

63

28b-Al_8 Eopt -3181.607032

C	-1.515181	0.461246	-0.218290
C	0.238665	2.039550	-1.240421
C	1.347046	1.284867	-1.212673
C	1.253940	-0.204536	-0.875160
C	0.620459	-0.445533	0.497513
C	-0.653124	-0.149799	0.809287
C	2.668004	1.941731	-1.588960
C	2.619817	-0.944098	-0.774530
C	3.841439	-0.007216	-0.574967
C	3.853148	0.994448	-1.717348
C	5.038687	-0.935618	-0.596512
C	3.837363	-1.831361	1.210587
C	2.533055	-1.919478	0.414817
H	2.531474	2.491890	-2.532126
H	0.363869	3.072378	-1.581237
H	3.805972	0.436805	-2.665750
H	3.954471	-2.647159	1.932641
O	-2.671814	-0.010806	-0.396857
C	-1.293300	-0.541085	2.118248
H	-0.603392	-0.340924	2.948491
H	-2.218751	0.021051	2.301837
H	-1.546532	-1.612693	2.133385
C	1.511734	-1.295940	1.372688
H	0.924989	-2.040469	1.929398
H	2.901647	2.706143	-0.830309
H	4.791820	1.564999	-1.737521
O	5.963440	-0.909415	-1.362565
O	4.993572	-1.857350	0.382778
C	3.848465	0.640082	0.846733
H	2.984005	1.314481	0.934038
C	3.613755	-0.472053	1.891472
H	4.234287	-0.323681	2.785184
H	2.268540	-2.939145	0.112191
H	2.785836	-1.475972	-1.721435
C	5.112332	1.448077	1.125678
H	5.248664	2.254587	0.390998
H	5.048731	1.910689	2.121855
H	6.017684	0.820024	1.110685
O	2.266507	-0.533571	2.314179
O	0.430377	-0.739039	-1.904994
C	0.144894	-2.116765	-1.867233
H	-0.606157	-2.303677	-2.646455
H	1.032143	-2.735471	-2.088396
H	-0.280944	-2.433544	-0.899928
C	-1.168459	1.684420	-0.912224
H	-1.837267	1.813115	-1.780550
Si	-1.866401	3.127715	0.276783
C	-0.843468	3.150382	1.840991
H	0.234854	3.121638	1.616587
H	-1.047575	4.081045	2.396280
H	-1.082105	2.306859	2.506315
C	-1.659914	4.706108	-0.705130
H	-2.195653	5.514467	-0.179834
H	-0.606836	5.011053	-0.799916
H	-2.092987	4.616782	-1.714040
C	-3.673557	2.789543	0.596490
H	-4.235803	2.709294	-0.347693
H	-3.846890	1.869962	1.174560
H	-4.097338	3.631032	1.169819
Al	-3.913679	-1.309668	-0.133487
Cl	-5.029718	-0.748993	1.608972
Cl	-5.091996	-1.270129	-1.916891
Cl	-2.897978	-3.183130	0.126427

63

28b-Al_9 Eopt -3181.606226

C	1.541848	0.465184	0.290461
C	-0.263166	1.740476	1.622923
C	-1.268661	0.859140	1.649836
C	-1.216153	-0.533428	1.049240
C	-0.543108	-0.670010	-0.314060
C	0.684214	-0.251042	-0.667326
C	-2.517519	1.110279	2.446283
C	-2.657770	-1.076022	0.790566
C	-3.713699	0.035346	0.464232
C	-3.775458	1.093816	1.576754
C	-5.021388	-0.733896	0.339375
C	-3.760445	-1.790862	-1.335120
C	-2.565667	-2.034352	-0.409941
H	-2.582377	0.314196	3.207288
H	-0.415748	2.677600	2.167735
H	-4.659963	0.912084	2.201795
H	-3.904589	-2.593036	-2.067300
O	2.765822	0.170554	0.351422
C	1.280755	-0.556435	-2.020342
H	0.505819	-0.491761	-2.794128
H	2.080756	0.149600	-2.279495
H	1.706631	-1.571935	-2.045956
C	-1.383747	-1.519373	-1.245053
H	-0.783848	-2.323716	-1.695249
H	-2.444920	2.062915	2.988780
H	-3.908100	2.087723	1.128757
O	-6.018991	-0.586441	0.992132
O	-4.987613	-1.661460	-0.632642
C	-3.479385	0.655656	-0.957695
H	-2.537821	1.225980	-0.928669
C	-3.297520	-0.482194	-1.988276

H	-3.813076	-0.256214	-2.930652
H	-2.454668	-3.082529	-0.112864
H	-2.995694	-1.591533	1.700814
C	-4.604188	1.593320	-1.394246
H	-5.554637	1.053133	-1.534580
H	-4.781438	2.403325	-0.673742
H	-4.345420	2.056401	-2.358069
O	-1.947595	-0.745862	-2.301875
O	-0.516970	-1.284435	2.032869
C	-0.336180	-2.662784	1.818147
H	0.136422	-2.879144	0.844010
H	0.341484	-3.017817	2.606105
H	-1.280965	-3.228702	1.895527
C	1.098097	1.628328	1.030678
H	1.866606	1.885395	1.778698
Si	1.315166	3.133857	-0.256729
C	3.023200	3.012516	-1.002180
H	3.796534	3.036635	-0.217661
H	3.176198	2.100595	-1.598739
H	3.188752	3.878628	-1.664393
C	-0.053605	3.018883	-1.523954
H	-0.001964	2.099665	-2.126499
H	-1.040696	3.060256	-1.036936
H	0.016950	3.877058	-2.213084
C	1.153547	4.676876	0.785950
H	0.139659	4.798606	1.196621
H	1.873504	4.677445	1.619566
H	1.367717	5.555554	0.154747
Al	4.098216	-1.029612	0.042469
Cl	3.181329	-2.970274	0.025769
Cl	4.995808	-0.488423	-1.823362
Cl	5.429835	-0.775470	1.695059

63

29a-Al_1 Eopt -3181.557792

C	-1.080277	0.418532	-0.544214
C	-0.538769	0.651473	0.823475
C	0.718868	1.067151	0.981355
C	1.532561	1.374597	-0.256031
C	1.167226	0.476525	-1.413061
C	-0.075467	0.021582	-1.590035
C	1.464508	1.169401	2.275070
C	3.031179	1.109832	-0.041118
C	3.310395	-0.074316	0.940504
C	2.611187	0.138638	2.301005
C	4.825633	-0.067260	1.051973
C	4.580207	-0.382549	-1.273770
C	3.586033	0.771626	-1.435199
H	1.881382	2.183507	2.375191
H	-1.192536	0.432440	1.670748
H	3.358450	0.442713	3.046467
H	5.242764	-0.511149	-2.136817
O	-2.280851	-0.410510	-0.556134
C	-0.505682	-0.906044	-2.684218
H	-0.623820	-1.926204	-2.286165
H	-1.473732	-0.611252	-3.117387
H	0.238515	-0.941997	-3.489379
C	2.408861	0.116833	-2.199939
H	2.345190	0.418591	-3.255234
H	0.795208	1.006248	3.131127
H	2.199064	-0.819620	2.644122
O	5.488415	0.071555	2.044791
O	5.427926	-0.234116	-0.139375
C	2.971615	-1.465983	0.293583
H	1.878844	-1.536549	0.193395
C	3.581358	-1.539015	-1.129434
H	4.024463	-2.525283	-1.319742
H	3.998148	1.646888	-1.948643
H	3.509423	2.004899	0.380855
C	3.457846	-2.643035	1.138713
H	4.558288	-2.688424	1.182697
H	3.081199	-2.603932	2.170122
H	3.107643	-3.586218	0.693703
O	2.660386	-1.287497	-2.165070
O	1.312113	2.696224	-0.742820
C	1.428032	3.759814	0.166147
H	2.414204	3.797049	0.663208
H	1.305641	4.685340	-0.413148
H	0.642813	3.733018	0.942916
C	-2.392439	0.985939	-0.959769
H	-2.524754	1.029096	-2.050486
Si	-3.530221	2.233885	-0.050727
C	-5.262884	1.549995	-0.233487
H	-5.483879	1.274213	-1.276937
H	-5.402245	0.656638	0.396095
H	-6.004798	2.300746	0.085015
C	-3.096397	2.503288	1.747063
H	-3.189556	1.579933	2.338198
H	-2.080952	2.908251	1.874118
H	-3.806216	3.238518	2.163123
C	-3.261378	3.801195	-1.041237
H	-3.566382	3.675390	-2.092412
H	-3.848197	4.631190	-0.614967
H	-2.198702	4.094704	-1.025381
Al	-3.000710	-1.895537	0.290168
Cl	-3.709584	-1.277447	2.209881
Cl	-4.550801	-2.513454	-1.030023
Cl	-1.291989	-3.170022	0.407094

63

29a-Al_2 Eopt -3181.558374

C	-1.078718	0.440104	-0.471899
C	-0.531016	0.499208	0.911532
C	0.724440	0.900488	1.106356
C	1.529515	1.355823	-0.089231
C	1.165077	0.580948	-1.338973
C	-0.080148	0.145009	-1.555322
C	1.452802	0.996914	2.405376
C	3.042089	1.093677	0.084514
C	3.333420	-0.121065	1.023865
C	2.691664	0.079466	2.410834
C	4.850753	-0.158911	1.102773
C	4.554813	-0.392574	-1.220032
C	3.593612	0.794315	-1.323897
H	1.769685	2.046298	2.524885
H	-1.190538	0.219328	1.735453
H	3.446478	0.489124	3.095200
H	5.193711	-0.508921	-2.102456
O	-2.306670	-0.338000	-0.585561
C	-0.512779	-0.692486	-2.718413
H	-1.476872	-0.355275	-3.128939
H	0.233278	-0.673144	-3.522360
H	-0.642012	-1.738710	-2.399366
C	2.404381	0.207144	-2.120783
H	2.369545	0.536927	-3.169591
H	0.794776	0.755788	3.251247
H	2.404549	-0.898283	2.818972
O	5.533316	-0.072062	2.088003
O	5.428570	-0.306931	-0.101949
C	2.935629	-1.478815	0.337171
H	1.839778	-1.508026	0.248857
C	3.524621	-1.521509	-1.097677
H	3.935174	-2.513920	-1.325355
H	4.038522	1.673929	-1.801556

H	3.511750	1.981565	0.532455
C	3.388641	-2.704611	1.128943
H	3.006054	-2.705821	2.158587
H	3.017369	-3.616728	0.638568
H	4.487388	-2.779795	1.175891
O	2.602062	-1.208111	-2.115690
O	1.217852	2.741538	-0.190148
C	1.802958	3.463545	-1.241877
H	1.349269	4.464131	-1.232703
H	2.894068	3.583243	-1.119840
H	1.603587	3.005570	-2.228000
C	-2.366854	1.101145	-0.815818
H	-2.492334	1.278008	-1.893806
Si	-3.443870	2.294766	0.229789
C	-5.210622	1.734710	-0.030041
H	-5.399891	0.778991	0.484458
H	-5.912195	2.479465	0.380686
H	-5.441588	1.602489	-1.099164
C	-3.002728	2.348858	2.044473
H	-1.967853	2.687441	2.205662
H	-3.675089	3.071404	2.537840
H	-3.141832	1.374523	2.536050
C	-3.074102	3.942186	-0.582995
H	-3.613977	4.754862	-0.070148
H	-1.995604	4.164312	-0.526151
H	-3.372988	3.952376	-1.643319
Al	-3.088995	-1.867969	0.114518
Cl	-1.420938	-3.200233	0.146704
Cl	-3.823815	-1.403643	2.067226
Cl	-4.627413	-2.317134	-1.285599

63

29b-Al_1 Eopt -3181.556564

C	-1.139526	0.339405	0.331459

C	-0.372775	-0.353964	1.405445
C	0.895991	-0.012088	1.628075
C	1.508435	1.097278	0.800532
C	0.942302	1.125574	-0.602128
C	-0.327817	0.790773	-0.849850
C	1.819402	-0.580848	2.654006
C	3.026641	0.900798	0.588888
C	3.449570	-0.602114	0.644891
C	3.037963	-1.246313	1.983240
C	4.959712	-0.557552	0.478517
C	4.295731	0.501244	-1.515254
C	3.346123	1.470985	-0.807534
H	2.157508	0.253571	3.290338
H	-0.903815	-1.075884	2.031678
H	3.894702	-1.215965	2.669657
H	4.784517	0.939263	-2.392550
O	-2.386696	-0.308850	-0.073798
C	-0.916405	0.652885	-2.220280
H	-0.956357	-0.412682	-2.497855
H	-1.944911	1.033792	-2.275387
H	-0.308236	1.175278	-2.968894
C	2.038844	1.331270	-1.622496
H	1.845104	2.178606	-2.296783
H	1.301929	-1.299502	3.303863
H	2.813764	-2.308039	1.817468
O	5.791341	-0.971465	1.240784
O	5.336915	0.025322	-0.672672
C	2.931956	-1.385265	-0.616441
H	1.835797	-1.448456	-0.552587
C	3.283332	-0.584839	-1.898082
H	3.638527	-1.252014	-2.694345
H	3.721470	2.499430	-0.770543
H	3.570002	1.429136	1.385792

C	3.491437	-2.804055	-0.709988
H	4.582832	-2.805568	-0.864626
H	3.276561	-3.400489	0.187069
H	3.037179	-3.322870	-1.567178
O	2.213577	0.163942	-2.428380
O	1.203095	2.274541	1.539135
C	1.641127	3.497982	1.008871
H	1.196220	4.291217	1.625379
H	2.739371	3.610307	1.051046
H	1.310115	3.646599	-0.035240
C	-2.454633	0.928695	0.703630
H	-2.735209	0.697174	1.740775
Si	-3.326515	2.493103	-0.003890
C	-4.591713	1.942735	-1.266853
H	-4.137178	1.449177	-2.139092
H	-5.311233	1.240017	-0.818022
H	-5.155409	2.818074	-1.630047
C	-4.152728	3.251250	1.496015
H	-4.680085	4.178527	1.218180
H	-4.890062	2.561296	1.937425
H	-3.412350	3.504664	2.272412
C	-2.049137	3.678799	-0.690330
H	-1.246300	3.843764	0.046135
H	-1.592745	3.330817	-1.628615
H	-2.528216	4.651971	-0.889676
Al	-3.057275	-2.049654	-0.021244
Cl	-4.785946	-1.981284	-1.261180
Cl	-3.492162	-2.431790	2.035131
Cl	-1.418160	-3.164155	-0.809933

63

29b-Al_2 Eopt -3181.555698

C	-1.139837	0.344716	0.280047
C	-0.383328	-0.288979	1.397738

C	0.885881	0.063723	1.609146
C	1.496802	1.124739	0.716922
C	0.943603	1.070386	-0.684296
C	-0.321889	0.721182	-0.923485
C	1.827571	-0.548255	2.598879
C	3.008427	0.915333	0.528223
C	3.429744	-0.590081	0.560709
C	2.962437	-1.283315	1.858591
C	4.943320	-0.518259	0.447712
C	4.324475	0.578101	-1.547036
C	3.331690	1.514491	-0.851529
H	2.257671	0.245588	3.228816
H	-0.909467	-1.003400	2.036904
H	3.821204	-1.402614	2.533031
H	4.837515	1.041410	-2.396986
O	-2.394679	-0.306260	-0.085821
C	-0.911581	0.536840	-2.287716
H	-1.939658	0.916202	-2.357526
H	-0.301322	1.030719	-3.053944
H	-0.953833	-0.537943	-2.527578
C	2.040876	1.340480	-1.689165
H	1.815467	2.199269	-2.337775
H	1.302710	-1.245679	3.266302
H	2.612254	-2.295374	1.616160
O	5.758212	-0.934203	1.226839
O	5.345101	0.101265	-0.676709
C	2.974649	-1.355053	-0.734299
H	1.878808	-1.447224	-0.706451
C	3.347727	-0.521641	-1.986495
H	3.742620	-1.162605	-2.785585
H	3.667506	2.555237	-0.791047
H	3.555821	1.425891	1.332951
C	3.575998	-2.756843	-0.833099
H	4.669369	-2.724935	-0.968534
H	3.364491	-3.368462	0.054757
H	3.152745	-3.279449	-1.703748
O	2.275364	0.211651	-2.530526
O	1.215468	2.448887	1.166155
C	1.470652	2.759378	2.511635
H	1.267389	3.832289	2.631734
H	0.810496	2.203242	3.200943
H	2.520761	2.576668	2.802343
C	-2.445383	0.979357	0.610266
H	-2.733743	0.816773	1.658528
Si	-3.270051	2.524703	-0.190448
C	-4.566886	1.938969	-1.404640
H	-5.103702	2.807056	-1.821940
H	-4.137134	1.373033	-2.245021
H	-5.305677	1.292982	-0.904511
C	-4.053744	3.397115	1.270249
H	-4.559439	4.319929	0.941918
H	-4.802906	2.756972	1.763735
H	-3.294931	3.678043	2.018999
C	-1.952690	3.616812	-0.954105
H	-1.614061	3.257605	-1.937114
H	-2.354113	4.634992	-1.088338
H	-1.073877	3.680054	-0.292027
Al	-3.089102	-2.032111	0.060248
Cl	-1.448762	-3.204590	-0.636031
Cl	-3.549664	-2.282416	2.131166
Cl	-4.800441	-2.013615	-1.204015

5

CH2N2 Eopt -148.571736

C	0.002757	-1.135102	-0.000000
H	0.969059	-1.638754	-0.000000
H	-0.961345	-1.642952	0.000000

N -0.003465 1.289070 0.000000

N 0.000000 0.152689 0.000000

2

N2 Eopt -109.394137

N -0.000000 -0.000000 0.548288

N 0.000000 0.000000 -0.548288

17

TMSCHN2 Eopt -557.051931

C -0.904863 0.997929 0.000514

H -0.957975 2.092322 0.001061

N -3.050031 -0.136741 -0.000099

N -2.058637 0.416784 0.000203

Si 0.676662 -0.016869 -0.000027

C 1.679419 0.400309 1.532274

H 1.124331 0.153066 2.451603

H 2.627469 -0.163261 1.542453

H 1.926734 1.474316 1.562856

C 1.679370 0.401732 -1.531958

H 2.627376 -0.161903 -1.542655

H 1.124291 0.155424 -2.451543

H 1.926773 1.475748 -1.561487

C 0.163101 -1.822145 -0.000797

H -0.434106 -2.072329 -0.893047

H 1.054203 -2.471371 -0.001197

H -0.433854 -2.073098 0.891407

53

TS1'_1 Eopt -1583.894492

C -1.935335 0.098704 0.141418

C -1.347154 -0.885910 -0.804310

C -0.127511 -1.390281 -0.586569

C 0.573277 -1.109965 0.714472

C 0.176989 0.207965 1.320483

C -1.032240 0.753908 1.139349

C 0.702901 -2.182524 -1.541440

C 2.091930 -1.026938 0.565277

C 2.521373 -0.470811 -0.821044

C 1.921787 -1.326677 -1.961130

C 4.038401 -0.538834 -0.772764

C 3.626017 0.839185 1.098923

C 2.567653 -0.094486 1.693706

H 1.046153 -3.099157 -1.036103

H -1.923250 -1.134292 -1.696418

H 2.704642 -1.982928 -2.364987

H 4.223873 1.358964 1.855740

O -3.184931 0.072119 0.460470

C -1.509748 1.997843 1.826325

H -1.380877 2.889208 1.191618

H -2.576527 1.912501 2.078554

H -0.946386 2.176133 2.751199

C 1.378721 0.855192 1.975789

H 1.235503 1.094539 3.038980

H 0.130846 -2.483908 -2.429309

H 1.612104 -0.666866 -2.783145

O 4.769737 -1.131848 -1.518759

O 4.550951 0.167360 0.251260

C 2.196021 1.061313 -0.937784

H 1.101681 1.170772 -0.974397

C 2.697415 1.793718 0.337452

H 3.170742 2.750512 0.080907

H 2.902096 -0.642553 2.580787

H 2.503066 -2.039529 0.672332

C 2.792502 1.699777 -2.191004

H 3.893976 1.713704 -2.156202

H 2.492782 1.176849 -3.109920

H 2.454867 2.743619 -2.275211

O 1.690995 2.069625 1.286661

O	0.370180	-2.183093	1.636140
C	-0.928573	-2.405194	2.127133
H	-0.873037	-3.310564	2.746649
H	-1.289542	-1.574005	2.757384
H	-1.665262	-2.580997	1.324980
C	-1.843493	1.697665	-1.325278
H	-0.963927	2.307781	-1.098953
H	-1.888484	1.210241	-2.302894
B	-4.253232	-0.679368	-0.319221
F	-5.452826	-0.336585	0.267360
F	-4.001713	-2.040133	-0.224465
F	-4.192061	-0.251242	-1.647740
N	-3.983691	2.643250	-0.646390
N	-2.990673	2.308646	-1.029624

53

TS1'_2 Eopt -1583.888693

C	-1.990956	-0.099474	-0.189938
C	-1.402585	0.902323	0.740069
C	-0.165770	1.371664	0.544470
C	0.545423	1.047441	-0.745307
C	0.147550	-0.290634	-1.309663
C	-1.077103	-0.802043	-1.145976
C	0.665524	2.119049	1.538925
C	2.065470	0.949978	-0.570048
C	2.469750	0.386209	0.826403
C	1.852721	1.226581	1.967844
C	3.986841	0.455263	0.795028
C	3.593614	-0.930999	-1.076009
C	2.539703	0.000078	-1.683693
H	1.050363	3.044383	1.084479
H	-1.983813	1.167766	1.624026
H	2.635163	1.854873	2.414766
H	4.199859	-1.451649	-1.825447
O	-3.232814	-0.055624	-0.541308
C	-1.568931	-2.050366	-1.814794
H	-1.523002	-2.923339	-1.143202
H	-2.613904	-1.926417	-2.134266
H	-0.961588	-2.289261	-2.697065
C	1.346178	-0.948764	-1.957478
H	1.196704	-1.185644	-3.020216
H	0.075323	2.405151	2.420221
H	1.503164	0.551110	2.760383
O	4.710132	1.055224	1.543584
O	4.509874	-0.255365	-0.220841
C	2.146371	-1.146459	0.944785
H	1.051929	-1.258715	0.974989
C	2.659432	-1.885018	-0.320186
H	3.131613	-2.840168	-0.055540
H	2.878109	0.533818	-2.577949
H	2.516675	1.947070	-0.667998
C	2.737357	-1.777238	2.205156
H	3.838936	-1.792390	2.174118
H	2.435398	-1.249566	3.120506
H	2.398890	-2.820433	2.293538
O	1.659864	-2.164559	-1.273975
O	0.222859	1.958242	-1.791996
C	0.311675	3.334036	-1.522840
H	0.112231	3.852683	-2.470420
H	-0.441099	3.662183	-0.784452
H	1.313409	3.639851	-1.170326
C	-1.958326	-1.637872	1.308816
H	-1.077757	-2.261048	1.126377
H	-2.028983	-1.133063	2.276271
B	-4.303513	0.726285	0.202911
F	-5.497320	0.390790	-0.400755
F	-4.030643	2.080039	0.094980

F	-4.277346	0.318401	1.540677
N	-4.087346	-2.590076	0.601239
N	-3.101970	-2.253863	1.000398

53

TS1'_3 Eopt -1583.888848

C	-1.996698	0.005322	0.181001
C	-1.386027	-0.835782	-0.881851
C	-0.145054	-1.312935	-0.749741
C	0.566131	-1.175760	0.572061
C	0.139080	0.056717	1.331668
C	-1.100501	0.556550	1.248008
C	0.673044	-2.004363	-1.787879
C	2.092059	-1.022510	0.419904
C	2.474434	-0.344051	-0.930642
C	1.914608	-1.146923	-2.124560
C	3.993609	-0.327486	-0.912482
C	3.546121	0.890950	1.053529
C	2.556640	-0.142605	1.598231
H	0.993532	-2.974888	-1.376291
H	-1.971118	-1.003292	-1.786785
H	2.707973	-1.797009	-2.517118
H	4.127372	1.388017	1.837987
O	-3.243389	-0.107227	0.503993
C	-1.607444	1.690631	2.088465
H	-1.499391	2.662351	1.580191
H	-2.671638	1.545992	2.324477
H	-1.049690	1.757575	3.031563
C	1.330914	0.718459	1.986671
H	1.218184	0.877454	3.068825
H	0.091162	-2.205171	-2.697392
H	1.653580	-0.454443	-2.935813
O	4.739686	-0.826998	-1.710633
O	4.489781	0.336798	0.146315
C	2.057454	1.171679	-0.930280
H	0.958264	1.216538	-0.937811
C	2.548234	1.841347	0.382936
H	2.961232	2.838879	0.184025
H	2.954385	-0.727020	2.434657
H	2.557898	-2.018064	0.439333
C	2.581516	1.937612	-2.143936
H	3.681763	2.001166	-2.143423
H	2.271729	1.482464	-3.094606
H	2.195122	2.967698	-2.127584
O	1.554588	1.992799	1.372974
O	0.208043	-2.369996	1.256141
C	0.712757	-2.561431	2.552299
H	0.225216	-3.461487	2.950938
H	1.803882	-2.731986	2.563842
H	0.475940	-1.718097	3.226168
C	-1.963813	1.755673	-1.044274
H	-1.098583	2.357237	-0.747826
H	-2.002644	1.401164	-2.078048
B	-4.304462	-0.745799	-0.376244
F	-5.509296	-0.486870	0.244776
F	-4.054957	-2.103983	-0.475599
F	-4.240928	-0.134535	-1.633886
N	-4.122056	2.570863	-0.256274
N	-3.124697	2.306687	-0.678126

53

TS1_A_1 Eopt -1583.894956

C	-2.096814	-0.126662	-0.273360
C	-1.505664	-0.797965	0.911837
C	-0.284288	-0.465610	1.347876
C	0.387550	0.746708	0.753178
C	0.023861	0.960118	-0.696975
C	-1.168740	0.596848	-1.188213

C	0.556794	-1.240901	2.310245
C	1.915782	0.634704	0.747188
C	2.411758	-0.829440	0.535193
C	1.807952	-1.786426	1.587054
C	3.919899	-0.692095	0.657371
C	3.510723	0.781881	-1.141461
C	2.380337	1.522908	-0.419463
H	0.871979	-0.581595	3.133909
H	-2.049824	-1.647721	1.325079
H	2.576440	-2.033618	2.331851
H	4.106337	1.427477	-1.796217
O	-3.119456	-0.658469	-0.856705
C	-1.625754	0.828151	-2.593376
H	-1.786601	-0.127721	-3.114288
H	-2.590141	1.360016	-2.600045
H	-0.892695	1.415327	-3.159628
C	1.224658	1.491798	-1.450748
H	1.031311	2.460791	-1.931782
H	-0.010761	-2.070145	2.753814
H	1.533031	-2.729153	1.095714
O	4.645334	-1.237158	1.444582
O	4.430280	0.162051	-0.248479
C	2.179695	-1.317611	-0.940124
H	1.097228	-1.445739	-1.090230
C	2.666966	-0.228526	-1.930259
H	3.199989	-0.676034	-2.779100
H	2.643618	2.536626	-0.099634
H	2.317429	0.982986	1.708532
C	2.871272	-2.648682	-1.232249
H	3.968892	-2.554322	-1.200280
H	2.580410	-3.436421	-0.523745
H	2.601146	-2.992841	-2.241560
O	1.635283	0.572311	-2.460889
O	-0.024847	1.954426	1.394565
C	0.044324	2.025187	2.797809
H	-0.243653	3.049211	3.071634
H	-0.654063	1.323347	3.286476
H	1.062186	1.841317	3.184121
C	-3.055188	1.574294	0.693618
H	-4.063004	1.455242	0.289033
H	-2.909199	1.412101	1.764851
B	-4.170963	-1.516403	-0.186726
F	-5.241731	-1.545857	-1.059919
F	-4.522647	-0.920939	1.025640
F	-3.653566	-2.789125	0.026986
N	-1.861752	3.513143	-0.182976
N	-2.466425	2.686689	0.262308

53

TS1_A_2 Eopt -1583.894598

C	1.964021	-0.046884	0.165032
C	1.330018	-0.811919	-0.927544
C	0.108492	-0.485739	-1.367233
C	-0.541664	0.784065	-0.873313
C	-0.122646	1.134408	0.536071
C	1.094034	0.828324	1.008619
C	-0.732716	-1.249910	-2.330358
C	-2.077942	0.679492	-0.800939
C	-2.554090	-0.783804	-0.545245
C	-2.024586	-1.735945	-1.637627
C	-4.069987	-0.677036	-0.565994
C	-3.575445	0.825331	1.179089
C	-2.516611	1.580523	0.370504
H	-0.990169	-0.568728	-3.158418
H	1.861097	-1.683157	-1.310078
H	-2.808046	-1.881200	-2.393194
H	-4.136246	1.470677	1.864312

O	2.994672	-0.512462	0.777977
C	1.592445	1.164534	2.379384
H	1.678211	0.257458	2.996842
H	2.597644	1.609091	2.325026
H	0.921180	1.867728	2.887137
C	-1.309075	1.626716	1.336675
H	-1.148515	2.610181	1.801795
H	-0.185267	-2.100298	-2.757937
H	-1.833409	-2.722196	-1.194975
O	-4.833030	-1.250235	-1.295583
O	-4.539919	0.174535	0.362343
C	-2.207433	-1.244447	0.917552
H	-1.114436	-1.344135	0.995648
C	-2.654611	-0.148838	1.922141
H	-3.116777	-0.596891	2.811290
H	-2.845507	2.573256	0.045058
H	-2.505390	1.008253	-1.759224
C	-2.842243	-2.585635	1.281990
H	-3.942202	-2.522637	1.312384
H	-2.569979	-3.383728	0.577961
H	-2.503593	-2.895833	2.281573
O	-1.616272	0.692499	2.373590
O	-0.107429	1.753723	-1.823490
C	-0.544531	3.080007	-1.657691
H	-0.013078	3.682319	-2.407018
H	-1.627722	3.193394	-1.836655
H	-0.302607	3.482087	-0.657186
C	2.889255	1.542667	-1.122620
H	3.036367	1.032664	-2.077028
H	2.210624	2.394837	-1.065633
B	3.976323	-1.541995	0.226386
F	5.035301	-1.550568	1.108687
F	4.366377	-1.119561	-1.042726

F	3.346187	-2.776046	0.168707
N	4.891917	1.667605	0.252041
N	4.009955	1.692745	-0.437079

53

TS1_A_3 Eopt -1583.889297

C	-1.957545	-0.365805	0.653521
C	-1.046427	-0.258632	1.816769
C	0.144851	0.339761	1.716715
C	0.502729	1.025580	0.418541
C	-0.134761	0.347656	-0.775444
C	-1.328663	-0.267274	-0.703840
C	1.207788	0.430729	2.756656
C	2.015827	0.979345	0.126194
C	2.706521	-0.267155	0.761720
C	2.480413	-0.306660	2.286750
C	4.173949	-0.082967	0.408492
C	3.231853	-0.109113	-1.748935
C	2.155912	0.925481	-1.407048
H	1.433305	1.499717	2.906513
H	-1.404108	-0.710796	2.744387
H	3.356519	0.126498	2.787317
H	3.589951	-0.033760	-2.781543
O	-2.982861	-1.114075	0.864649
C	-1.953248	-0.983874	-1.862298
H	-2.847942	-0.451777	-2.212814
H	-1.243305	-1.077202	-2.691092
H	-2.276157	-1.992074	-1.569295
C	0.857457	0.259270	-1.918961
H	0.475980	0.691483	-2.855021
H	0.865021	0.027105	3.718596
H	2.421432	-1.352305	2.615539
O	5.102078	0.000971	1.166426
O	4.380960	-0.012050	-0.917907

C	2.281208	-1.587783	0.023404
H	1.218535	-1.774404	0.240112
C	2.417988	-1.385408	-1.508936
H	2.847279	-2.274997	-1.987592
H	2.340949	1.908403	-1.853189
H	2.489971	1.873693	0.555188
C	3.086479	-2.806525	0.471557
H	4.150968	-2.716043	0.200466
H	3.027785	-2.973337	1.555769
H	2.697430	-3.708398	-0.023721
O	1.203744	-1.103923	-2.168780
O	0.025717	2.357721	0.601517
C	0.275471	3.293277	-0.420909
H	-0.291212	4.198676	-0.164258
H	1.342353	3.565447	-0.489902
H	-0.066289	2.939762	-1.410417
C	-2.881909	1.648681	0.890406
H	-3.870855	1.369050	1.258229
H	-2.180077	2.176495	1.538978
B	-4.342807	-1.217896	0.197612
F	-4.414560	-2.422385	-0.478782
F	-4.522816	-0.132252	-0.660552
F	-5.264767	-1.166246	1.231884
N	-2.793491	2.460953	-1.404784
N	-2.891746	2.148409	-0.334736

53

TS1_A_4 Eopt -1583.888536

C	-1.992197	-0.028576	0.689700
C	-1.104022	0.601081	1.699473
C	0.107692	1.065471	1.374543
C	0.489589	1.080838	-0.086079
C	-0.134657	-0.058235	-0.855239
C	-1.334438	-0.573177	-0.539820
C	1.194210	1.480466	2.313172
C	1.997192	0.903460	-0.299035
C	2.659247	-0.016379	0.774011
C	2.376584	0.492436	2.205079
C	4.131558	0.048859	0.403575
C	3.231136	-0.795617	-1.609518
C	2.135283	0.272346	-1.693629
H	1.539888	2.491564	2.046782
H	-1.477656	0.589760	2.726090
H	3.282988	0.967739	2.603861
H	3.614699	-1.104985	-2.587815
O	-3.033620	-0.581274	1.205765
C	-1.965772	-1.697391	-1.303134
H	-2.800111	-1.331873	-1.917909
H	-1.226966	-2.181686	-1.951379
H	-2.377935	-2.453351	-0.622136
C	0.845686	-0.559185	-1.898825
H	0.439302	-0.505231	-2.918305
H	0.831564	1.519552	3.349256
H	2.161324	-0.367087	2.853744
O	5.044299	0.427627	1.086622
O	4.361310	-0.374461	-0.852808
C	2.270629	-1.525135	0.573422
H	1.208817	-1.642509	0.837506
C	2.427980	-1.906747	-0.920287
H	2.875428	-2.902985	-1.029835
H	2.296223	1.016988	-2.480173
H	2.494933	1.881404	-0.244021
C	3.096596	-2.465301	1.450924
H	4.157476	-2.474928	1.152468
H	3.049081	-2.196165	2.515112
H	2.718739	-3.493472	1.350242
O	1.218369	-1.912150	-1.643901

O 0.025163 2.252128 -0.759100
C 0.319204 3.506446 -0.193344
H -0.048082 4.261177 -0.901662
H -0.191491 3.657800 0.774282
H 1.401801 3.667982 -0.051004
C -2.899213 1.845275 -0.107815
H -3.943081 1.670903 0.157989
H -2.354958 2.655407 0.382522
B -4.368160 -1.044385 0.651010
F -4.377447 -2.428639 0.607229
F -4.566874 -0.487236 -0.611633
F -5.318672 -0.567367 1.540386
N -2.386268 1.662494 -2.482257
N -2.682610 1.812438 -1.414914
53
TS1_B_1 Eopt -1583.895652
C 2.038757 -0.317009 0.389284
C 1.507580 -0.163722 -0.976770
C 0.320194 0.422186 -1.183988
C -0.400928 1.050899 -0.014248
C -0.120657 0.334503 1.286482
C 1.060455 -0.253252 1.522006
C -0.414986 0.537250 -2.473556
C -1.933666 0.997615 -0.158334
C -2.402510 -0.231600 -0.997674
C -1.754246 -0.227062 -2.397291
C -3.911678 -0.064266 -1.065912
C -3.611996 -0.156331 1.268346
C -2.495909 0.891944 1.273193
H -0.609967 1.609007 -2.644053
H 2.088299 -0.579566 -1.799972
H -2.460830 0.208792 -3.115912
H -4.246794 -0.114602 2.160235
O 3.060101 -1.076203 0.600629
C 1.437217 -0.959120 2.785325
H 1.546186 -2.040083 2.609186
H 2.410524 -0.596946 3.150592
H 0.684292 -0.808429 3.568507
C -1.384541 0.212300 2.108879
H -1.284643 0.615704 3.127040
H 0.188590 0.171869 -3.314797
H -1.583799 -1.262818 -2.718563
O -4.591305 0.041354 -2.050781
O -4.482986 -0.037901 0.150951
C -2.185224 -1.573751 -0.207862
H -1.102199 -1.751101 -0.125869
C -2.747559 -1.421721 1.230770
H -3.284398 -2.327523 1.540945
H -2.810343 1.859160 1.679368
H -2.277157 1.906491 -0.673076
C -2.821841 -2.778337 -0.899832
H -3.921007 -2.701232 -0.925424
H -2.468657 -2.904380 -1.932353
H -2.570188 -3.696626 -0.348970
O -1.772878 -1.158732 2.215083
O 0.100025 2.386275 -0.013915
C -0.357899 3.262004 0.987315
H 0.237144 4.180967 0.896995
H -1.420632 3.529450 0.857987
H -0.212321 2.852410 2.002989
C 2.964450 1.656671 0.759567
H 2.358336 2.153483 1.520731
H 3.972236 1.325679 1.017731
B 4.167786 -1.340139 -0.398870
F 5.223046 -1.843003 0.337085
F 4.505098 -0.128702 -1.006804

F	3.723086	-2.254852	-1.345065
N	2.755943	2.663588	-1.448903
N	2.908856	2.273046	-0.413359

53

TS1_B_2 Eopt -1583.894600

C	-2.009275	-0.501969	-0.236596
C	-1.516253	0.295990	0.903755
C	-0.330837	0.920820	0.839485
C	0.406686	0.944982	-0.479118
C	0.154409	-0.290030	-1.307829
C	-1.008328	-0.949906	-1.256577
C	0.427167	1.497349	1.990286
C	1.928878	0.967625	-0.299812
C	2.400511	0.161679	0.951481
C	1.697930	0.654285	2.236517
C	3.898889	0.414798	0.962215
C	3.657348	-0.577611	-1.166782
C	2.495397	0.332108	-1.579801
H	0.717814	2.532791	1.754548
H	-2.095223	0.266827	1.827205
H	2.408234	1.240337	2.835101
H	4.314518	-0.850287	-1.999778
O	-3.006188	-1.302183	-0.070885
C	-1.362226	-2.143122	-2.085502
H	-1.511633	-3.028946	-1.449915
H	-2.309012	-1.971990	-2.621197
H	-0.576218	-2.367969	-2.816365
C	1.414614	-0.669681	-2.056145
H	1.276525	-0.689286	-3.146357
H	-0.187891	1.524729	2.899720
H	1.414327	-0.214421	2.845506
O	4.551600	0.921674	1.833991
O	4.494039	0.005059	-0.172854
C	2.267882	-1.389157	0.738820
H	1.197312	-1.642439	0.733762
C	2.849686	-1.774298	-0.645669
H	3.432613	-2.702518	-0.585722
H	2.758958	1.075585	-2.339508
H	2.278725	2.000277	-0.169624
C	2.951817	-2.191010	1.845803
H	4.046251	-2.062573	1.826559
H	2.597923	-1.908706	2.846952
H	2.745018	-3.262745	1.709195
O	1.877750	-1.956663	-1.650095
O	-0.036636	2.018924	-1.313430
C	0.085166	3.338677	-0.836540
H	-0.278960	3.992711	-1.640295
H	-0.526929	3.522661	0.063374
H	1.128963	3.619093	-0.615117
C	-2.943093	1.020448	-1.537770
H	-2.217296	1.199284	-2.333840
H	-3.888256	0.521064	-1.761161
B	-4.142599	-1.092426	0.910178
F	-5.153462	-1.937436	0.497276
F	-4.535854	0.245178	0.838460
F	-3.706153	-1.406915	2.191160
N	-3.103585	2.883330	0.024465
N	-3.081596	2.073077	-0.746054

53

TS1_B_3 Eopt -1583.888194

C	2.029354	-0.031776	-0.448123
C	1.194182	0.746717	-1.382914
C	-0.015625	1.202188	-1.034290
C	-0.481012	0.998879	0.389159
C	0.094035	-0.255732	1.002246
C	1.315143	-0.717060	0.686971

C	-1.008298	1.899704	-1.898281
C	-2.007694	0.818419	0.493194
C	-2.618842	0.191261	-0.798331
C	-2.287938	1.046547	-2.038807
C	-4.110734	0.158477	-0.508551
C	-3.313703	-1.142197	1.286134
C	-2.246913	-0.121031	1.690519
H	-1.253805	2.856881	-1.409634
H	1.617572	0.913095	-2.375847
H	-3.139855	1.704994	-2.254350
H	-3.742900	-1.678977	2.139269
O	3.061919	-0.556365	-1.010892
C	1.901243	-1.959397	1.281320
H	2.812044	-1.723901	1.848467
H	1.182926	-2.451713	1.946043
H	2.190837	-2.670490	0.495779
C	-0.966551	-0.980735	1.806029
H	-0.663658	-1.185352	2.842984
H	-0.590141	2.130025	-2.887103
H	-2.175961	0.389536	-2.910963
O	-4.988494	0.686803	-1.135869
O	-4.407031	-0.558342	0.589501
C	-2.207369	-1.319145	-0.943551
H	-1.128347	-1.361511	-1.155400
C	-2.449193	-2.049650	0.404076
H	-2.884697	-3.043828	0.240209
H	-2.488764	0.418220	2.612558
H	-2.472825	1.803941	0.640110
C	-2.950541	-2.032822	-2.071996
H	-4.031799	-2.102033	-1.869913
H	-2.820031	-1.535282	-3.042637
H	-2.569520	-3.059827	-2.172940
O	-1.290156	-2.227502	1.186927
O	-0.032503	2.177321	1.053377
C	-0.271698	2.290524	2.435140
H	0.261938	3.189447	2.772750
H	-1.342348	2.422018	2.667076
H	0.114425	1.424480	3.001741
C	2.974376	1.601325	0.727082
H	2.629779	1.461231	1.754858
H	4.024336	1.402563	0.502957
B	4.364515	-1.173976	-0.543477
F	4.275650	-2.551148	-0.663584
F	4.623192	-0.790131	0.773115
F	5.334951	-0.662510	-1.390784
N	2.081392	3.599168	-0.347252
N	2.541591	2.737713	0.195271

53

TS1_B_4 Eopt -1583.886316

C	-2.032022	-0.011216	0.382826
C	-1.231630	0.840467	1.288537
C	-0.005367	1.268616	0.959042
C	0.491896	0.991610	-0.439582
C	-0.066333	-0.288411	-1.008398
C	-1.285059	-0.750724	-0.695016
C	1.006763	1.869926	1.878485
C	2.010402	0.794168	-0.489309
C	2.571948	0.100897	0.791796
C	2.168465	0.869406	2.070615
C	4.071015	0.114367	0.541999
C	3.343264	-1.105835	-1.344485
C	2.260343	-0.089004	-1.721000
H	1.396156	2.800832	1.438445
H	-1.671289	1.026524	2.271104
H	3.044111	1.404376	2.462269
H	3.808458	-1.588535	-2.210789

O	-3.067614	-0.506978	0.964631
C	-1.876565	-1.988440	-1.291702
H	-2.781034	-1.745239	-1.866899
H	-1.156111	-2.486580	-1.949962
H	-2.179107	-2.696356	-0.508585
C	0.988911	-0.961210	-1.865006
H	0.665725	-1.104531	-2.905623
H	0.564061	2.122307	2.851431
H	1.868549	0.147813	2.842190
O	4.922944	0.625345	1.217404
O	4.405832	-0.532540	-0.589134
C	2.198656	-1.423745	0.849093
H	1.117971	-1.503313	1.039181
C	2.482732	-2.078566	-0.527019
H	2.937567	-3.070305	-0.406905
H	2.488129	0.493802	-2.619896
H	2.515694	1.764851	-0.575873
C	2.943367	-2.167504	1.957358
H	4.026711	-2.215698	1.760532
H	2.800740	-1.702168	2.942603
H	2.575377	-3.202174	2.021165
O	1.339679	-2.238857	-1.335636
O	0.061649	1.995137	-1.364324
C	0.504822	3.319517	-1.182757
H	0.053921	3.909591	-1.992148
H	0.178893	3.748488	-0.219794
H	1.600590	3.417783	-1.258930
C	-2.942769	1.463920	-0.986605
H	-2.412021	1.330853	-1.931689
H	-3.988279	1.157914	-0.910950
B	-4.336475	-1.235816	0.569551
F	-4.147254	-2.598487	0.733057
F	-4.667997	-0.919961	-0.749338
F	-5.308133	-0.763228	1.436069
N	-2.392549	3.554553	0.138684
N	-2.690157	2.639395	-0.432037

53

TS1_B_5 Eopt -1583.887223

C	-1.922075	0.018135	0.338371
C	-1.157308	0.969272	1.173760
C	0.066731	1.389615	0.829285
C	0.587856	1.030789	-0.540839
C	0.039289	-0.282288	-1.041662
C	-1.174438	-0.749359	-0.706973
C	1.055422	2.071520	1.717595
C	2.108363	0.838803	-0.554622
C	2.654422	0.244951	0.780063
C	2.233860	1.108412	1.991034
C	4.156773	0.239265	0.552765
C	3.456016	-1.115344	-1.250383
C	2.381683	-0.127602	-1.717603
H	1.432684	2.979199	1.222406
H	-1.609880	1.214153	2.137381
H	3.099467	1.688872	2.337634
H	3.933761	-1.660705	-2.071532
O	-2.965156	-0.426013	0.932089
C	-1.733562	-2.034759	-1.231193
H	-2.700331	-1.864455	-1.723991
H	-1.045447	-2.497081	-1.947525
H	-1.912324	-2.746139	-0.413490
C	1.113920	-1.008576	-1.827836
H	0.814917	-1.228789	-2.862235
H	0.594720	2.378326	2.666206
H	1.946232	0.447635	2.819613
O	4.999164	0.798305	1.201475
O	4.507700	-0.489949	-0.521978

C	2.276156	-1.271109	0.942376
H	1.192753	-1.335037	1.122820
C	2.579486	-2.024343	-0.378741
H	3.029482	-3.005560	-0.179578
H	2.627573	0.392200	-2.649538
H	2.602920	1.807558	-0.707645
C	3.002829	-1.934457	2.111830
H	4.088575	-1.998899	1.934112
H	2.848058	-1.399544	3.059109
H	2.631016	-2.961181	2.244718
O	1.449734	-2.242075	-1.193300
O	0.200333	1.980431	-1.536753
C	0.473803	3.342523	-1.301201
H	0.201391	3.877773	-2.220633
H	-0.126928	3.749351	-0.468854
H	1.541232	3.534140	-1.097412
C	-2.853796	1.446460	-1.184276
H	-1.988859	2.055995	-1.455029
H	-3.309186	0.752532	-1.892794
B	-4.222824	-1.221702	0.574291
F	-3.974441	-2.567881	0.762114
F	-4.574508	-0.932378	-0.740804
F	-5.185339	-0.762892	1.452487
N	-4.397201	2.350389	0.461652
N	-3.713641	1.995252	-0.354813

53

TS1_B_6 Eopt -1583.888534

C	-1.916540	0.017435	0.386229
C	-1.126854	0.872927	1.295916
C	0.088592	1.323718	0.964672
C	0.593041	1.086589	-0.437839
C	0.030407	-0.180998	-1.040373
C	-1.188646	-0.659492	-0.734739
C	1.059687	2.042328	1.835337
C	2.120646	0.897217	-0.494663
C	2.689042	0.293162	0.826190
C	2.322460	1.174913	2.038543
C	4.188721	0.250321	0.581943
C	3.440771	-1.081771	-1.211449
C	2.391108	-0.064394	-1.667159
H	1.332543	2.981689	1.326842
H	-1.575353	1.064581	2.273103
H	3.174175	1.826181	2.275658
H	3.893511	-1.636103	-2.040787
O	-2.959696	-0.472219	0.945388
C	-1.751114	-1.909605	-1.335131
H	-2.731144	-1.711186	-1.789201
H	-1.080952	-2.312504	-2.102492
H	-1.903400	-2.679864	-0.566931
C	1.110863	-0.920001	-1.805640
H	0.836066	-1.141645	-2.846922
H	0.616496	2.304448	2.805074
H	2.167035	0.534625	2.916606
O	5.049054	0.788171	1.224915
O	4.514766	-0.488259	-0.493110
C	2.268269	-1.213014	0.987389
H	1.183730	-1.248408	1.169767
C	2.545887	-1.969061	-0.339248
H	2.970941	-2.962165	-0.144426
H	2.663607	0.456723	-2.590874
H	2.594494	1.877835	-0.645243
C	2.977438	-1.906224	2.149854
H	4.063052	-1.986113	1.977255
H	2.825231	-1.385873	3.105330
H	2.587513	-2.928506	2.263658
O	1.409741	-2.156249	-1.153749

O	0.178591	2.259829	-1.136752
C	0.478671	2.347100	-2.510497
H	-0.030181	3.244855	-2.886377
H	1.559857	2.463758	-2.695642
H	0.105937	1.475061	-3.076423
C	-2.825648	1.635581	-0.948150
H	-1.961495	2.297569	-1.051084
H	-3.228391	1.073403	-1.792402
B	-4.232819	-1.198241	0.502565
F	-4.015863	-2.562121	0.547467
F	-4.567894	-0.761740	-0.776353
F	-5.192852	-0.817377	1.419590
N	-4.464133	2.250529	0.739124
N	-3.740173	2.032814	-0.089740

65

TS1a-Al'_1 Eopt -3290.903326

C	1.221354	-0.056389	-0.263585
C	0.611674	-0.391891	1.059023
C	-0.577674	-1.002187	1.087753
C	-1.198725	-1.389283	-0.233902
C	-0.924032	-0.357063	-1.304316
C	0.265438	0.253404	-1.383392
C	-1.332355	-1.493880	2.277385
C	-2.729949	-1.530033	-0.193923
C	-3.373556	-0.642835	0.911042
C	-2.792917	-0.985028	2.296332
C	-4.857467	-0.947086	0.816934
C	-4.452779	-0.185840	-1.377677
C	-3.235032	-1.089407	-1.584185
H	-1.332798	-2.594559	2.205244
H	1.197364	-0.225817	1.966435
H	-3.430920	-1.746577	2.764133
H	-5.036601	-0.030111	-2.291670
O	2.338737	-0.601867	-0.609555
C	0.705951	1.120189	-2.519525
H	0.033783	1.018070	-3.380946
H	0.723741	2.183307	-2.239645
H	1.720812	0.839109	-2.840458
C	-2.165238	-0.114263	-2.132177
H	-1.995111	-0.200907	-3.215037
H	-0.819267	-1.232317	3.212511
H	-2.855673	-0.099017	2.941110
O	-5.573677	-1.372637	1.682560
O	-5.360474	-0.683888	-0.402896
C	-3.246225	0.871739	0.532087
H	-2.179091	1.122661	0.565071
C	-3.725634	1.085522	-0.927910
H	-4.336645	1.993285	-1.014937
H	-3.426516	-1.934745	-2.253785
H	-2.985276	-2.576737	0.025189
C	-3.991693	1.808114	1.479296
H	-5.080681	1.640420	1.448141
H	-3.663867	1.701493	2.522357
H	-3.811212	2.851750	1.181098
O	-2.679233	1.195781	-1.870275
O	-0.557268	-2.624465	-0.529772
C	-0.869153	-3.254576	-1.744482
H	-0.714912	-2.590845	-2.614694
H	-0.182342	-4.106449	-1.839500
H	-1.902969	-3.642849	-1.770627
C	1.962689	1.922238	0.304729
H	2.291245	1.596289	1.302588
N	3.894235	1.826288	-1.186101
N	3.042326	1.931222	-0.472090
Si	1.024772	3.612473	0.359502
C	1.499851	4.370917	2.003509

H	1.006011	5.348687	2.130206
H	1.190678	3.725790	2.841906
H	2.588291	4.527436	2.073255
C	1.655148	4.663011	-1.057475
H	2.745285	4.813510	-0.993823
H	1.423512	4.242968	-2.048414
H	1.180444	5.656694	-1.000828
C	-0.813638	3.327738	0.285499
H	-1.144338	2.803971	1.195169
H	-1.326199	4.304004	0.260140
H	-1.130918	2.749357	-0.594967
Al	3.642822	-1.530568	0.236967
Cl	2.869585	-3.379820	0.979772
Cl	4.315210	-0.257196	1.848826
Cl	5.158373	-1.793558	-1.251433

65

TS1a-Al'_2 Eopt -3290.904098

C	1.210785	-0.043063	-0.276649
C	0.616289	-0.457367	1.032868
C	-0.578429	-1.060880	1.041651
C	-1.204976	-1.383569	-0.294075
C	-0.937999	-0.309537	-1.319501
C	0.244391	0.314637	-1.372576
C	-1.383434	-1.488426	2.227992
C	-2.728553	-1.520843	-0.245321
C	-3.390705	-0.598305	0.820108
C	-2.787017	-0.838342	2.221070
C	-4.860803	-0.963101	0.730761
C	-4.463935	-0.277224	-1.497277
C	-3.207546	-1.137612	-1.657626
H	-1.497997	-2.583131	2.186262
H	1.193991	-0.302079	1.947135
H	-3.473894	-1.468414	2.802239
H	-5.049388	-0.182336	-2.418447
O	2.331733	-0.552787	-0.664248
C	0.687097	1.212981	-2.482817
H	0.756954	2.260860	-2.155992
H	1.680874	0.907561	-2.845355
H	-0.013736	1.178276	-3.326315
C	-2.157338	-0.134916	-2.199075
H	-1.936712	-0.256302	-3.268935
H	-0.863592	-1.253826	3.166925
H	-2.725924	0.120494	2.752116
O	-5.569773	-1.380882	1.606191
O	-5.358819	-0.772293	-0.505589
C	-3.327484	0.902549	0.380147
H	-2.273512	1.199889	0.414576
C	-3.794521	1.041107	-1.090142
H	-4.440371	1.918833	-1.221868
H	-3.341720	-2.008924	-2.307291
H	-2.998227	-2.555686	0.005809
C	-4.125306	1.839028	1.284364
H	-3.825534	1.765588	2.338883
H	-3.966264	2.880338	0.966248
H	-5.207789	1.639102	1.226889
O	-2.734350	1.159655	-2.014226
O	-0.623666	-2.545800	-0.882874
C	-0.584808	-3.724931	-0.119060
H	-1.586791	-4.057446	0.207453
H	-0.162612	-4.503253	-0.768898
H	0.068263	-3.626906	0.763898
C	1.937289	1.913237	0.386858
H	2.278711	1.546721	1.366185
N	3.848604	1.913445	-1.133538
N	3.007578	1.970202	-0.401525
Si	0.990007	3.595199	0.512703

C	1.411103	4.257387	2.211556
H	1.076488	3.564156	3.000403
H	2.496400	4.409829	2.324867
H	0.911309	5.225931	2.378776
C	1.655330	4.721005	-0.828160
H	2.738457	4.890870	-0.714007
H	1.472264	4.337750	-1.844241
H	1.156274	5.701154	-0.750039
C	-0.844209	3.314782	0.363894
H	-1.130561	2.800124	-0.565353
H	-1.199672	2.727208	1.223809
H	-1.359651	4.289342	0.391513
Al	3.686559	-1.473066	0.101117
Cl	3.003517	-3.417930	0.680963
Cl	4.300356	-0.312759	1.817588
Cl	5.212186	-1.543952	-1.395736

65

TS1a-Al'_3 Eopt -3290.903882

C	-1.239374	-0.034478	0.323577
C	-0.587193	-0.352629	-0.983913
C	0.651977	-0.862892	-1.007021
C	1.219922	-1.267696	0.347595
C	0.912983	-0.192365	1.371891
C	-0.304901	0.356755	1.437550
C	1.455799	-1.053480	-2.270823
C	2.742529	-1.471432	0.422903
C	3.487294	-0.759787	-0.730221
C	2.955757	-1.320467	-2.048582
C	4.948384	-1.094418	-0.518224
C	4.449587	-0.067388	1.540056
C	3.196763	-0.913463	1.787119
H	1.025758	-1.880852	-2.858024
H	-1.136454	-0.146691	-1.905654
H	3.144669	-2.404510	-2.033704
H	4.986880	0.189719	2.459811
O	-2.347385	-0.591447	0.671918
C	-0.785265	1.238392	2.545850
H	-0.862477	2.289419	2.230462
H	-1.779266	0.912898	2.888685
H	-0.102001	1.203160	3.403746
C	2.142120	0.134350	2.182823
H	1.954623	0.201627	3.263195
H	1.320054	-0.149796	-2.884578
H	3.530456	-0.927003	-2.898078
O	5.687838	-1.646564	-1.287764
O	5.396655	-0.699922	0.687389
C	3.376327	0.784755	-0.557524
H	2.318494	1.055601	-0.660405
C	3.782805	1.154994	0.894988
H	4.411000	2.055420	0.912987
H	3.329229	-1.696353	2.541928
H	2.969537	-2.542358	0.345908
C	4.166158	1.569721	-1.598087
H	5.249969	1.383955	-1.521391
H	3.852059	1.315489	-2.620781
H	4.003875	2.648891	-1.456221
O	2.681837	1.393431	1.749943
O	0.549167	-2.426985	0.835525
C	0.555124	-3.549543	-0.010144
H	0.016880	-3.358976	-0.953675
H	1.576274	-3.899715	-0.246864
H	0.032582	-4.353070	0.525530
C	-2.028436	1.898827	-0.327193
H	-2.431884	1.488785	-1.265006
N	-3.868412	1.964123	1.277156
N	-3.053248	1.991568	0.514400

Si	-1.032685	3.529902	-0.600674
C	-1.601685	4.158985	-2.269877
H	-1.076881	5.094150	-2.526495
H	-1.388734	3.424173	-3.063352
H	-2.684328	4.363862	-2.273563
C	-1.490354	4.738958	0.753706
H	-1.158553	4.418456	1.753368
H	-1.005035	5.706108	0.540806
H	-2.577761	4.915680	0.790043
C	0.788638	3.144448	-0.633641
H	1.143850	2.690502	0.303554
H	1.016413	2.464588	-1.469269
H	1.352930	4.077463	-0.800050
Al	-3.682622	-1.532351	-0.098380
Cl	-4.627441	-0.199043	-1.510383
Cl	-4.986172	-2.037316	1.520779
Cl	-2.901886	-3.257612	-1.101477

65

TS1a-Al'_4 Eopt -3290.915551

C	-1.241210	-0.163706	0.297178
C	-0.673738	-0.493282	-1.039054
C	0.494294	-1.141923	-1.132489
C	1.145440	-1.651389	0.125609
C	0.866402	-0.769481	1.313231
C	-0.289504	-0.104613	1.456809
C	1.283299	-1.435108	-2.366202
C	2.666909	-1.736893	0.036776
C	3.279963	-0.651338	-0.884059
C	2.664228	-0.734817	-2.298723
C	4.764754	-0.969653	-0.869983
C	4.387386	-0.650550	1.439852
C	3.166746	-1.574437	1.483102
H	1.429005	-2.526147	-2.422921
H	-1.243129	-0.196818	-1.923524
H	3.364177	-1.266424	-2.957415
H	4.985950	-0.673455	2.357088
O	-2.461763	-0.463644	0.580364
C	-0.709057	0.608622	2.704449
H	-0.664446	1.701061	2.586359
H	-1.743627	0.338087	2.964317
H	-0.059971	0.343604	3.548502
C	2.095794	-0.715512	2.198556
H	1.906870	-1.010735	3.239993
H	0.747505	-1.125618	-3.273798
H	2.566140	0.278169	-2.710602
O	5.470644	-1.218056	-1.809594
O	5.279893	-0.953308	0.373201
C	3.173160	0.765213	-0.220870
H	2.109822	1.038509	-0.200975
C	3.662441	0.687097	1.250993
H	4.276799	1.559249	1.509106
H	3.348923	-2.534732	1.976622
H	2.926343	-2.721009	-0.375241
C	3.941871	1.841688	-0.982453
H	3.658495	1.887708	-2.042702
H	3.740261	2.827853	-0.538386
H	5.030192	1.674846	-0.931793
O	2.624829	0.613389	2.206240
O	0.764041	-3.002401	0.398942
C	-0.579765	-3.294372	0.688348
H	-0.622880	-4.375866	0.876351
H	-0.942987	-2.774451	1.591653
H	-1.255035	-3.065282	-0.151952
C	-1.547589	1.969079	-0.174675
H	-2.064893	1.739565	-1.119185
N	-3.266096	2.368748	1.513901

N	-2.488350	2.246839	0.721036
Si	-0.169733	3.284916	-0.423815
C	0.937668	3.385922	1.076976
H	1.775362	4.071064	0.863173
H	0.380530	3.813617	1.926950
H	1.353581	2.418584	1.394306
C	0.667369	2.755524	-2.008811
H	1.050833	1.727237	-1.960190
H	-0.050966	2.810382	-2.842987
H	1.506173	3.430297	-2.242817
C	-1.042237	4.926057	-0.667648
H	-0.304518	5.724903	-0.852316
H	-1.729237	4.892303	-1.528331
H	-1.623601	5.208276	0.225524
Al	-3.994406	-0.955140	-0.236706
Cl	-4.683740	0.780418	-1.316451
Cl	-5.304934	-1.495131	1.364959
Cl	-3.621674	-2.580374	-1.588124

65

TS1a-Al-6 Eopt -3290.915781

C	0.892602	0.183612	-0.135575
C	0.226918	0.625790	1.108743
C	-0.988235	0.169973	1.443924
C	-1.603096	-0.940313	0.621481
C	-1.168947	-0.899134	-0.823112
C	0.032721	-0.438083	-1.194315
C	-1.887511	0.746575	2.488583
C	-3.131649	-0.841864	0.560651
C	-3.647514	0.630706	0.578584
C	-3.110044	1.399829	1.806102
C	-5.156370	0.452175	0.606935
C	-4.637897	-0.684801	-1.396912
C	-3.526003	-1.524327	-0.759488
H	-2.230713	-0.055081	3.160051
H	0.716167	1.417860	1.680432
H	-3.916543	1.511981	2.543250
H	-5.190495	-1.215866	-2.179652
O	1.900557	0.852481	-0.561484
C	0.514778	-0.351203	-2.608225
H	0.655722	0.700384	-2.901372
H	1.489966	-0.844622	-2.726752
H	-0.198264	-0.812666	-3.301396
C	-2.326234	-1.304234	-1.712879
H	-2.095117	-2.173563	-2.344541
H	-1.359486	1.488760	3.102135
H	-2.822909	2.413520	1.496539
O	-5.926236	0.842034	1.442526
O	-5.608242	-0.240381	-0.454525
C	-3.364976	1.369262	-0.779173
H	-2.280957	1.542133	-0.855259
C	-3.780952	0.456535	-1.961173
H	-4.283913	1.033207	-2.748111
H	-3.783221	-2.580571	-0.627823
H	-3.569174	-1.353335	1.429185
C	-4.079107	2.717523	-0.872372
H	-5.173883	2.597747	-0.914241
H	-3.844869	3.375731	-0.024354
H	-3.770855	3.237094	-1.791639
O	-2.708405	-0.228136	-2.568259
O	-1.212435	-2.240781	1.072595
C	-1.322131	-2.533566	2.447363
H	-1.063549	-3.594631	2.561149
H	-0.622536	-1.934494	3.056421
H	-2.346489	-2.386044	2.829963
C	1.789764	-1.716245	0.619144
H	0.826267	-2.248377	0.661666

N	2.419598	-1.019032	2.871561
N	2.141742	-1.382361	1.849139
Si	3.116153	-2.539744	-0.496601
C	4.257711	-3.490293	0.646557
H	5.031958	-4.015788	0.063253
H	4.773105	-2.807911	1.342985
H	3.710026	-4.239969	1.239925
C	2.106509	-3.684091	-1.582487
H	1.340291	-3.137015	-2.153933
H	2.759859	-4.202229	-2.303434
H	1.596152	-4.449026	-0.975281
C	4.122426	-1.324633	-1.498330
H	3.514510	-0.628852	-2.092391
H	4.783598	-0.732003	-0.848463
H	4.760947	-1.899051	-2.190992
Al	3.155443	2.047234	-0.083933
Cl	2.164781	3.737262	0.802840
Cl	4.468181	1.087073	1.327011
Cl	4.142311	2.572600	-1.909831

65

TS1a-Al-7 Eopt -3290.917791

C	-1.073774	-0.049897	0.135763
C	-0.350616	-0.454076	1.356014
C	0.881139	0.004866	1.609630
C	1.465132	1.076528	0.716816
C	0.944722	0.993544	-0.700737
C	-0.282952	0.533926	-0.985251
C	1.800357	-0.433676	2.694819
C	2.994972	0.945694	0.566964
C	3.473329	-0.532396	0.718659
C	3.042789	-1.114974	2.080738
C	4.985815	-0.437473	0.595889
C	4.347001	0.490810	-1.470265
C	3.336616	1.457864	-0.845945
H	2.107571	0.464780	3.255096
H	-0.844651	-1.179719	2.006676
H	3.882885	-1.036378	2.783330
H	4.846998	0.901990	-2.354030
O	-2.133811	-0.702145	-0.179294
C	-0.816230	0.329956	-2.369114
H	-0.821134	-0.744562	-2.610856
H	-1.852932	0.682860	-2.455885
H	-0.201330	0.845537	-3.116536
C	2.063940	1.221979	-1.693228
H	1.850330	2.027900	-2.410602
H	1.300545	-1.112300	3.398317
H	2.832208	-2.186114	1.966255
O	5.806235	-0.780035	1.403708
O	5.376819	0.102984	-0.571034
C	3.023420	-1.398808	-0.514599
H	1.928124	-1.499865	-0.483242
C	3.390824	-0.653223	-1.825958
H	3.797441	-1.347255	-2.572735
H	3.670994	2.500747	-0.851789
H	3.484325	1.538450	1.353013
C	3.632050	-2.800078	-0.513581
H	4.726793	-2.772176	-0.638258
H	3.410725	-3.352701	0.409553
H	3.219420	-3.380308	-1.352074
O	2.314827	0.026859	-2.433688
O	1.083788	2.283603	1.373012
C	1.496469	3.498403	0.792072
H	0.964189	4.299511	1.322611
H	2.580104	3.668570	0.911359
H	1.238942	3.559040	-0.279396
C	-1.991886	1.844810	0.963963

H	-1.091880	2.202920	1.482968
N	-3.514912	0.475895	2.277461
N	-2.804658	1.165635	1.748703
Si	-2.909304	2.964906	-0.291186
C	-3.747301	4.321221	0.696349
H	-4.315162	4.988015	0.025939
H	-4.454008	3.901375	1.430650
H	-3.010490	4.934187	1.240192
C	-1.646459	3.720104	-1.444087
H	-1.101173	2.982471	-2.048458
H	-2.167887	4.403647	-2.135062
H	-0.916326	4.317301	-0.875417
C	-4.194120	1.892573	-1.128574
H	-3.763847	0.991058	-1.587993
H	-4.951279	1.558744	-0.399780
H	-4.717760	2.466186	-1.910861
Al	-2.765012	-2.382220	-0.158117
Cl	-3.029273	-3.055791	1.865391
Cl	-4.617014	-2.299496	-1.228983
Cl	-1.233338	-3.518282	-1.160740

65

TS1a-Al-8 Eopt -3290.916394

C	-0.985921	-0.409182	-0.366624
C	-0.458086	-0.202489	1.001470
C	0.740264	0.350542	1.203160
C	1.482379	0.934620	0.026762
C	1.192488	0.192722	-1.260553
C	0.010754	-0.402795	-1.481521
C	1.469750	0.472610	2.496389
C	3.011804	0.831773	0.187722
C	3.434288	-0.397662	1.052371
C	2.774155	-0.350092	2.446146
C	4.947460	-0.271594	1.132720
C	4.668246	-0.397620	-1.201817
C	3.584377	0.683342	-1.235713
H	1.709394	1.538180	2.650011
H	-1.071385	-0.583330	1.820407
H	3.492967	0.059825	3.167979
H	5.312210	-0.389697	-2.088073
O	-1.943697	-1.270951	-0.505455
C	-0.361153	-1.147723	-2.724132
H	0.378149	-0.998272	-3.520512
H	-0.443047	-2.226187	-2.519765
H	-1.348235	-0.819508	-3.086560
C	2.460452	0.021783	-2.069994
H	2.378073	0.411945	-3.094757
H	0.846412	0.150221	3.341102
H	2.553548	-1.374298	2.774367
O	5.620261	-0.165188	2.122242
O	5.531691	-0.285112	-0.078152
C	3.186653	-1.746493	0.283886
H	2.099928	-1.894491	0.195516
C	3.766533	-1.636148	-1.151414
H	4.279073	-2.562539	-1.441264
H	3.932075	1.632858	-1.655865
H	3.386250	1.732284	0.694757
C	3.783848	-2.955120	1.003319
H	4.884698	-2.909898	1.033209
H	3.422442	-3.048644	2.036496
H	3.507798	-3.876829	0.470142
O	2.809818	-1.360888	-2.150275
O	1.018637	2.286073	-0.024488
C	1.671552	3.179152	-0.895033
H	1.093205	4.113097	-0.879761
H	2.698994	3.409248	-0.566976
H	1.703986	2.811247	-1.935196

C	-2.037253	1.395635	-0.754984
H	-2.923129	0.876745	-1.153717
N	-0.775860	2.359640	-2.612077
N	-1.401695	1.950915	-1.782081
Si	-2.508616	2.691608	0.624859
C	-4.318791	2.361353	0.952454
H	-4.466874	1.356622	1.378968
H	-4.699749	3.096425	1.680962
H	-4.924471	2.441159	0.036424
C	-1.521020	2.585100	2.203823
H	-0.447097	2.712842	2.008693
H	-1.859960	3.414233	2.848978
H	-1.683225	1.645422	2.750327
C	-2.234232	4.369775	-0.164428
H	-1.167459	4.540753	-0.383832
H	-2.805400	4.489840	-1.099271
H	-2.559739	5.159657	0.532511
Al	-3.493018	-1.883927	0.112836
Cl	-4.995182	-0.866951	-1.057794
Cl	-3.716586	-1.411027	2.206228
Cl	-3.488916	-3.997001	-0.234675

65

TS1a-Al-9 Eopt -3290.915097

C	-0.928360	-0.369857	-0.328957
C	-0.416840	-0.044316	1.021974
C	0.787617	0.512607	1.194552
C	1.533769	1.012587	-0.016859
C	1.233761	0.207684	-1.259374
C	0.060292	-0.411205	-1.445141
C	1.555444	0.584111	2.474602
C	3.055305	0.893795	0.142844
C	3.478190	-0.344863	0.991997
C	2.792419	-0.336608	2.377058
C	4.987268	-0.190175	1.081938
C	4.716925	-0.286286	-1.261370
C	3.606357	0.769158	-1.286899
H	1.884608	1.620205	2.648735
H	-1.017303	-0.404169	1.859941
H	3.525634	-0.041172	3.139686
H	5.367256	-0.255747	-2.142414
O	-1.864570	-1.257250	-0.399610
C	-0.315699	-1.201903	-2.657926
H	0.435175	-1.102796	-3.451006
H	-0.422442	-2.268456	-2.407727
H	-1.291209	-0.869027	-3.047072
C	2.485102	0.081259	-2.103149
H	2.360569	0.484760	-3.117736
H	0.933446	0.287221	3.330028
H	2.475464	-1.358441	2.624863
O	5.656492	-0.085157	2.074104
O	5.572428	-0.168583	-0.129508
C	3.267496	-1.689380	0.207093
H	2.185269	-1.864725	0.115980
C	3.844670	-1.548268	-1.225770
H	4.379741	-2.457990	-1.527454
H	3.915147	1.732699	-1.705916
H	3.449361	1.783920	0.650632
C	3.895135	-2.888006	0.917772
H	4.994623	-2.816962	0.945345
H	3.538755	-2.995411	1.951585
H	3.639733	-3.813155	0.380227
O	2.877511	-1.285928	-2.217289
O	1.160445	2.348987	-0.357310
C	1.302163	3.354497	0.616738
H	1.112758	4.309727	0.108569
H	0.576359	3.245062	1.440311

H	2.318240	3.398916	1.044790
C	-2.049250	1.362227	-0.929027
H	-2.802704	0.746093	-1.442407
N	-0.596259	2.432513	-2.575230
N	-1.315070	1.973370	-1.854383
Si	-2.831455	2.584904	0.364747
C	-4.676592	2.346601	0.179886
H	-4.979018	1.330215	0.476162
H	-5.209116	3.062132	0.828328
H	-5.006577	2.513062	-0.857863
C	-2.331539	2.257177	2.133312
H	-1.253105	2.365513	2.313223
H	-2.857992	2.999794	2.757709
H	-2.649986	1.257617	2.464249
C	-2.311122	4.303226	-0.170545
H	-1.217967	4.433090	-0.156269
H	-2.673163	4.539172	-1.184436
H	-2.743081	5.045729	0.520523
Al	-3.377756	-1.965970	0.191841
Cl	-4.901683	-1.207037	-1.131557
Cl	-3.751508	-1.351715	2.224499
Cl	-3.152190	-4.090662	0.033806

65

TS1a-Al_1 Eopt -3290.916555

C	-1.146772	-0.062024	0.110073
C	-0.448134	-0.431566	1.360588
C	0.798658	-0.006364	1.603214
C	1.407089	1.018443	0.672134
C	0.902378	0.890806	-0.742511
C	-0.331186	0.455253	-1.024161
C	1.729933	-0.536657	2.643587
C	2.926993	0.852227	0.541646
C	3.378158	-0.639653	0.638841
C	2.881000	-1.294598	1.946953
C	4.892860	-0.538690	0.572504
C	4.316650	0.482579	-1.475207
C	3.279739	1.418264	-0.843861
H	2.144559	0.298840	3.227846
H	-0.960239	-1.139697	2.017450
H	3.723549	-1.392983	2.644574
H	4.847875	0.930498	-2.322081
O	-2.202792	-0.724804	-0.203024
C	-0.878980	0.261890	-2.403131
H	-0.237446	0.731583	-3.158406
H	-0.945861	-0.813636	-2.631122
H	-1.895045	0.671827	-2.491836
C	2.021409	1.186794	-1.716113
H	1.786050	2.022387	-2.391070
H	1.209356	-1.205179	3.342450
H	2.532298	-2.312480	1.728021
O	5.689417	-0.913867	1.389841
O	5.317068	0.057655	-0.556162
C	2.980163	-1.454202	-0.644965
H	1.886134	-1.570116	-0.651783
C	3.379652	-0.652120	-1.910762
H	3.814168	-1.309502	-2.674921
H	3.590603	2.467604	-0.805174
H	3.433012	1.401964	1.346718
C	3.609611	-2.846482	-0.675768
H	4.706304	-2.798831	-0.774576
H	3.377151	-3.430362	0.225493
H	3.225051	-3.406059	-1.541242
O	2.313066	0.042610	-2.516728
O	1.071655	2.354055	1.050335
C	1.307471	2.754630	2.378683
H	1.059513	3.823405	2.429265

H	0.666435	2.214262	3.097663
H	2.361819	2.632863	2.682247
C	-2.052455	1.856046	0.846862
H	-1.335601	2.072508	1.646381
N	-4.006480	0.580353	1.529436
N	-3.125347	1.236340	1.303937
Si	-2.444447	3.278270	-0.387237
C	-3.916859	2.703109	-1.391283
H	-4.827022	2.668560	-0.770228
H	-4.108136	3.401417	-2.222475
H	-3.764290	1.699480	-1.818263
C	-2.894178	4.754413	0.678395
H	-3.174073	5.613317	0.045613
H	-3.745818	4.528232	1.340094
H	-2.042163	5.061811	1.306589
C	-0.930731	3.682934	-1.405014
H	-0.779806	2.987604	-2.242370
H	-1.054732	4.695796	-1.823387
H	-0.030496	3.672562	-0.771432
Al	-2.847652	-2.396120	-0.083995
Cl	-1.258323	-3.610218	-0.884179
Cl	-3.239835	-2.883093	1.973131
Cl	-4.624239	-2.408814	-1.276524

65

TS1a-Al_2 Eopt -3290.918063

C	-1.060202	0.156427	-0.102931
C	-0.431232	0.284747	1.215546
C	0.804450	0.794004	1.348565
C	1.470591	1.419026	0.142483
C	1.010996	0.823607	-1.163199
C	-0.214106	0.309717	-1.323842
C	1.694916	0.625778	2.534586
C	2.985677	1.183316	0.140001
C	3.373415	-0.207101	0.734612
C	2.798851	-0.388663	2.158516
C	4.892105	-0.166077	0.712082
C	4.431086	0.150098	-1.581719
C	3.403808	1.261366	-1.336333
H	2.155867	1.589449	2.799144
H	-0.964222	-0.161706	2.058879
H	3.615256	-0.321892	2.890244
H	5.010790	0.288392	-2.500896
O	-2.061050	-0.653118	-0.203877
C	-0.738061	-0.277285	-2.596220
H	-1.763926	0.067389	-2.790348
H	-0.104605	-0.009400	-3.450556
H	-0.771803	-1.375883	-2.528086
C	2.168220	0.782586	-2.139429
H	1.979847	1.360693	-3.055187
H	1.137682	0.265731	3.409694
H	2.371281	-1.396384	2.247398
O	5.643218	-0.274664	1.643338
O	5.379429	0.024796	-0.527229
C	2.996977	-1.384360	-0.234738
H	1.900931	-1.473252	-0.256188
C	3.472421	-1.044944	-1.671167
H	3.911891	-1.923549	-2.160700
H	3.747980	2.259816	-1.626726
H	3.490471	1.945317	0.747571
C	3.574934	-2.724287	0.217816
H	4.675780	-2.736041	0.162721
H	3.285574	-2.977000	1.247220
H	3.203330	-3.526189	-0.437005
O	2.454688	-0.561585	-2.519035
O	1.141687	2.802823	0.009533
C	1.585422	3.707912	0.993781

H	2.684936	3.781556	1.040584
H	1.192081	4.692012	0.704770
H	1.201744	3.466958	1.999133
C	-2.099245	2.161455	-0.320753
H	-1.566517	2.544917	-1.199893
N	-1.386038	3.213029	1.756563
N	-1.710859	2.794840	0.767748
Si	-3.998695	1.885998	-0.429293
C	-4.363648	0.815938	-1.913779
H	-3.941008	1.254412	-2.832112
H	-3.978131	-0.205834	-1.793973
H	-5.456932	0.757144	-2.046960
C	-4.536301	1.151259	1.202689
H	-4.062504	0.177766	1.395174
H	-4.290484	1.825453	2.039911
H	-5.628979	1.004608	1.205948
C	-4.757670	3.587181	-0.656207
H	-4.408611	4.060312	-1.588311
H	-5.856832	3.510504	-0.706757
H	-4.504997	4.255064	0.183522
Al	-2.370238	-2.345474	0.300790
Cl	-3.845858	-3.113573	-1.048104
Cl	-3.048351	-2.367685	2.342452
Cl	-0.460907	-3.330770	0.110795
65			
TS1a-Al_3		Eopt	-3290.919298
C	-1.055773	0.142888	-0.089800
C	-0.405655	0.158766	1.223131
C	0.824833	0.669223	1.378809
C	1.480310	1.378043	0.214114
C	1.017405	0.854260	-1.123931
C	-0.218904	0.368364	-1.306228
C	1.683636	0.591266	2.590915
C	3.009894	1.186900	0.192197
C	3.434653	-0.169361	0.836161
C	2.929718	-0.271265	2.290576
C	4.952714	-0.147739	0.762897
C	4.428055	0.074238	-1.521287
C	3.425768	1.209599	-1.292358
H	1.994269	1.617948	2.844594
H	-0.944064	-0.313594	2.048270
H	3.741531	0.018997	2.970422
H	4.977267	0.168443	-2.464469
O	-2.079232	-0.628909	-0.241151
C	-0.741925	-0.168710	-2.601071
H	-1.777494	0.159605	-2.769170
H	-0.122903	0.156142	-3.446461
H	-0.746886	-1.269749	-2.584811
C	2.181979	0.736495	-2.082382
H	2.026563	1.270019	-3.031375
H	1.134179	0.185501	3.450353
H	2.692078	-1.318246	2.518693
O	5.728916	-0.226929	1.676385
O	5.407348	-0.021055	-0.495670
C	3.005184	-1.378859	-0.073048
H	1.906902	-1.444262	-0.065579
C	3.449452	-1.105206	-1.535421
H	3.862300	-2.011545	-1.996701
H	3.796065	2.188145	-1.616463
H	3.480808	1.997195	0.767096
C	3.562253	-2.716920	0.409391
H	3.274722	-2.940794	1.445738
H	3.170010	-3.526982	-0.222919
H	4.662226	-2.751284	0.347035
O	2.422912	-0.639042	-2.383285
O	1.101290	2.736056	0.413170

C	1.544476	3.690701	-0.519483
H	1.049547	4.635849	-0.257997
H	2.635091	3.850894	-0.470683
H	1.265559	3.429332	-1.555959
C	-2.051635	2.191850	-0.102853
H	-1.519543	2.653971	-0.944456
N	-1.289132	3.032186	2.052940
N	-1.646935	2.713548	1.039866
Si	-3.952949	1.960566	-0.234489
C	-4.333479	1.038437	-1.812118
H	-3.871284	1.534058	-2.681130
H	-3.995300	-0.006088	-1.773680
H	-5.424890	1.040696	-1.970664
C	-4.508392	1.094065	1.325649
H	-4.271995	1.700229	2.216035
H	-5.600747	0.946593	1.306047
H	-4.034505	0.109173	1.446239
C	-4.687116	3.686695	-0.308579
H	-5.787413	3.631004	-0.362758
H	-4.423414	4.274332	0.585947
H	-4.332952	4.235242	-1.196442
Al	-2.438173	-2.341105	0.152668
Cl	-4.012565	-2.939384	-1.170331
Cl	-0.586495	-3.384533	-0.212700
Cl	-3.015076	-2.493606	2.219883

65

TS1a-Al_4 Eopt -3290.917648

C	-0.965245	-0.276582	-0.399990
C	-0.460867	-0.075549	0.976805
C	0.741285	0.461797	1.205540
C	1.521853	1.043423	0.052967
C	1.217872	0.355007	-1.260655
C	0.026707	-0.210099	-1.513054
C	1.464826	0.520127	2.507525
C	3.042932	0.867876	0.229831
C	3.400429	-0.406185	1.056081
C	2.719613	-0.376826	2.441150
C	4.915967	-0.345589	1.160590
C	4.665301	-0.379411	-1.181077
C	3.628336	0.747352	-1.190431
H	1.763274	1.565991	2.687143
H	-1.073876	-0.488580	1.779712
H	3.448200	-0.040785	3.190914
H	5.322088	-0.367964	-2.057810
O	-1.901350	-1.151827	-0.564176
C	-0.346750	-0.894207	-2.789993
H	-1.312359	-0.512701	-3.158652
H	0.411999	-0.741764	-3.566927
H	-0.475713	-1.975229	-2.629841
C	2.489488	0.165313	-2.062324
H	2.437937	0.594724	-3.073182
H	0.820715	0.210208	3.341251
H	2.431075	-1.398334	2.722200
O	5.578742	-0.301134	2.161468
O	5.515922	-0.342116	-0.042787
C	3.109555	-1.716393	0.237762
H	2.019124	-1.818786	0.132952
C	3.711645	-1.579302	-1.186199
H	4.187846	-2.515880	-1.503909
H	4.022520	1.695268	-1.571123
H	3.446027	1.734073	0.773107
C	3.650759	-2.970544	0.922658
H	4.752257	-2.971200	0.962403
H	3.276720	-3.080802	1.949711
H	3.342250	-3.863347	0.358994
O	2.781239	-1.227578	-2.186474

O	1.126080	2.412043	0.037675
C	1.817915	3.300905	-0.805917
H	2.854718	3.475556	-0.471889
H	1.832608	2.963633	-1.856550
H	1.279860	4.257585	-0.760096
C	-2.106700	1.505882	-0.783128
H	-2.908219	0.949386	-1.290686
N	-0.743573	2.690873	-2.424596
N	-1.420281	2.183591	-1.692849
Si	-2.755492	2.606371	0.674350
C	-3.008740	1.601350	2.227613
H	-3.514177	0.642285	2.040614
H	-2.066939	1.407908	2.760705
H	-3.658283	2.190114	2.897645
C	-1.524096	3.986382	0.937789
H	-1.426814	4.622079	0.042520
H	-1.868886	4.627915	1.765639
H	-0.530914	3.585086	1.188456
C	-4.396491	3.264983	0.055586
H	-4.839589	3.945420	0.801797
H	-4.276541	3.824578	-0.886189
H	-5.107593	2.442043	-0.119007
Al	-3.345293	-1.988273	0.047475
Cl	-3.195733	-2.329695	2.167106
Cl	-5.008739	-0.689097	-0.410380
Cl	-3.447016	-3.826780	-1.048861

65

TS1a-Al_5 Eopt -3290.913042

C	1.132798	-0.071993	0.105666
C	0.534399	-0.203354	-1.238224
C	-0.673628	0.297633	-1.513367
C	-1.345405	1.231190	-0.539187
C	-0.872820	1.046059	0.882525
C	0.348048	0.583343	1.191133
C	-1.539826	-0.069712	-2.669038
C	-2.866347	0.993703	-0.467358
C	-3.231444	-0.496216	-0.750094
C	-2.696905	-0.941609	-2.130640
C	-4.749881	-0.507452	-0.691456
C	-4.260282	0.299342	1.467198
C	-3.293713	1.375396	0.962577
H	-1.937115	0.850403	-3.126019
H	1.054934	-0.848511	-1.949994
H	-3.523860	-0.939802	-2.853257
H	-4.824800	0.607849	2.353970
O	1.859228	-1.052134	0.528939
C	0.858521	0.413469	2.588264
H	0.793105	-0.637989	2.905204
H	1.918390	0.703227	2.649982
H	0.287794	1.026467	3.296854
C	-2.039287	1.160980	1.844756
H	-1.902651	1.925795	2.623087
H	-0.982273	-0.618105	-3.439854
H	-2.338072	-1.977122	-2.061547
O	-5.513325	-0.834464	-1.559452
O	-5.222760	-0.085176	0.494403
C	-2.776020	-1.422265	0.437240
H	-1.676794	-1.455700	0.443491
C	-3.242778	-0.801962	1.782697
H	-3.629850	-1.575944	2.457788
H	-3.700603	2.389814	1.029454
H	-3.367471	1.610170	-1.227108
C	-3.295136	-2.852573	0.302866
H	-4.392458	-2.900643	0.395344
H	-3.016471	-3.308709	-0.656903
H	-2.868446	-3.477223	1.101416

O	-2.244793	-0.097419	2.490162
O	-1.021698	2.523371	-1.039305
C	-1.600020	3.645420	-0.419179
H	-1.091685	4.527996	-0.831289
H	-2.677836	3.736817	-0.637389
H	-1.455604	3.647888	0.675063
C	2.736400	1.372801	-0.414108
H	2.826872	1.059580	-1.464325
N	4.389298	0.194450	0.934970
N	3.693008	0.778985	0.283243
Si	2.603743	3.282707	-0.193005
C	4.366015	3.907539	-0.034095
H	4.370407	5.005970	0.065281
H	4.865712	3.491445	0.856378
H	4.967518	3.645259	-0.919388
C	1.815543	3.883424	-1.773738
H	1.643180	4.971229	-1.727026
H	2.476886	3.682229	-2.631986
H	0.851955	3.379627	-1.940909
C	1.657097	3.727414	1.355818
H	2.173479	3.352683	2.254048
H	1.601687	4.825975	1.438061
H	0.633858	3.330365	1.353134
Al	2.452412	-2.641780	-0.077431
Cl	3.714131	-2.201559	-1.766161
Cl	3.529894	-3.509585	1.552199
Cl	0.718629	-3.789840	-0.624268

65

TS1a-B'_1 Eopt -1992.371519

C	-1.776279	-0.550465	0.348724
C	-1.221632	-0.619038	-1.033432
C	0.022388	-1.069644	-1.232403
C	0.781822	-1.646048	-0.069760
C	0.438316	-0.959949	1.227584
C	-0.782611	-0.466635	1.477763
C	0.796940	-1.073562	-2.510540
C	2.297093	-1.511181	-0.200999
C	2.728922	-0.238678	-0.976202
C	2.072274	-0.206356	-2.374047
C	4.241292	-0.359796	-1.039087
C	3.918801	-0.402153	1.300359
C	2.826838	-1.474534	1.243191
H	1.084749	-2.115252	-2.727791
H	-1.845271	-0.302934	-1.871309
H	2.809335	-0.535550	-3.118909
H	4.551219	-0.468577	2.192442
O	-2.880534	-1.123167	0.682472
C	-1.250588	0.017378	2.814916
H	-1.470299	1.094184	2.816451
H	-2.177874	-0.507365	3.091052
H	-0.498047	-0.169895	3.590872
C	1.682841	-0.863210	2.088902
H	1.569382	-1.317756	3.082862
H	0.188165	-0.718154	-3.353213
H	1.821383	0.831689	-2.629680
O	4.935299	-0.387728	-2.019200
O	4.799580	-0.441275	0.182848
C	2.471388	1.051363	-0.123133
H	1.384191	1.179110	-0.043672
C	3.024711	0.843243	1.312725
H	3.534439	1.745757	1.674082
H	3.147170	-2.459008	1.599734
H	2.668684	-2.387304	-0.748769
C	3.066234	2.310641	-0.748340
H	2.757026	3.194857	-0.171262
H	4.168172	2.284763	-0.743838

H	2.738715	2.457461	-1.786654
O	2.043668	0.508621	2.271660
O	0.589825	-3.059387	0.033498
C	-0.701644	-3.560125	0.273671
H	-0.603100	-4.653514	0.311522
H	-1.117418	-3.218199	1.236840
H	-1.411570	-3.304029	-0.529863
C	-2.441080	1.520111	0.297517
H	-3.029896	1.460312	1.223001
B	-4.015671	-1.694360	-0.144580
F	-3.577125	-2.033009	-1.418107
F	-5.004465	-0.717961	-0.200215
F	-4.444160	-2.811135	0.548918
N	-3.858707	1.424577	-1.693174
N	-3.267769	1.486079	-0.746747
Si	-1.238628	3.024639	0.116762
C	-0.072183	3.081454	1.571138
H	0.488181	2.153205	1.751369
H	0.653749	3.893800	1.396764
H	-0.626394	3.334322	2.489484
C	-0.422463	2.838674	-1.556530
H	-1.179821	2.878799	-2.357117
H	0.281520	3.669064	-1.727503
H	0.124725	1.891369	-1.657193
C	-2.324418	4.552846	0.115930
H	-2.875499	4.652665	1.064924
H	-1.706567	5.457322	-0.013191
H	-3.057303	4.527548	-0.707043

65

TS1a-B'_2 Eopt -1992.374019

C	-1.518975	-0.841449	0.293391
C	-1.000715	-0.423950	-1.039374
C	0.278245	-0.643814	-1.367883
C	1.139064	-1.475689	-0.455630
C	0.751076	-1.328674	0.989695
C	-0.512172	-1.113589	1.376969
C	1.005503	-0.149649	-2.576794
C	2.621676	-1.109890	-0.507651
C	2.861142	0.388132	-0.828787
C	2.186526	0.765764	-2.165428
C	4.375207	0.498925	-0.890433
C	4.110170	-0.323318	1.305892
C	3.182839	-1.465322	0.881796
H	1.396882	-1.027066	-3.117027
H	-1.692946	0.069851	-1.721801
H	2.944908	0.745743	-2.959553
H	4.763145	-0.580823	2.147154
O	-2.615493	-1.517409	0.415537
C	-0.975276	-1.160920	2.803419
H	-1.962556	-1.641312	2.866849
H	-0.274119	-1.735205	3.422575
H	-1.056293	-0.158308	3.252213
C	1.990582	-1.330869	1.857246
H	1.977471	-2.084145	2.657747
H	0.333975	0.385112	-3.262660
H	1.831683	1.803118	-2.108712
O	5.045858	0.870966	-1.815128
O	4.965224	0.118632	0.257464
C	2.449695	1.297907	0.382414
H	1.353405	1.267085	0.472336
C	3.046629	0.712969	1.691656
H	3.431568	1.513435	2.336939
H	3.651161	-2.454898	0.906911
H	3.090516	-1.706872	-1.301253
C	2.887646	2.750357	0.202244
H	2.529807	3.180603	-0.743417

H	2.490052	3.367025	1.022277
H	3.985110	2.849305	0.223290
O	2.140066	-0.042738	2.464284
O	1.123131	-2.850014	-0.853002
C	-0.077447	-3.574103	-0.748448
H	-0.899352	-3.128826	-1.332012
H	0.132066	-4.573430	-1.153957
H	-0.411600	-3.688771	0.297384
C	-2.064556	1.141548	0.943450
H	-1.278687	1.336280	1.687210
B	-3.687987	-1.671291	-0.642732
F	-4.174504	-0.403854	-0.953930
F	-4.663474	-2.462425	-0.068624
F	-3.139666	-2.276810	-1.768180
N	-4.127395	0.326567	1.954730
N	-3.185385	0.781898	1.565417
Si	-2.274805	2.692408	-0.202310
C	-0.561993	3.107519	-0.810216
H	-0.158275	2.317452	-1.457930
H	-0.600156	4.041488	-1.394801
H	0.129484	3.265954	0.030669
C	-3.483983	2.425068	-1.595937
H	-3.601203	3.387762	-2.122200
H	-3.145322	1.677491	-2.326778
H	-4.472813	2.118058	-1.225511
C	-2.913448	4.029855	0.946420
H	-3.901942	3.767628	1.358206
H	-2.223945	4.201013	1.788673
H	-3.021451	4.980187	0.397458

65

TS1a-B'_3 Eopt -1992.367267

C	1.777498	-0.658236	0.310034
C	0.973146	-0.385352	1.534505
C	-0.311609	-0.740717	1.601193
C	-0.872243	-1.525051	0.442212
C	-0.268031	-1.093303	-0.870984
C	1.019503	-0.737408	-0.994552
C	-1.283859	-0.434803	2.695828
C	-2.383622	-1.341450	0.265151
C	-2.876863	0.071773	0.696992
C	-2.488688	0.374030	2.159705
C	-4.380613	-0.004654	0.497871
C	-3.647727	-0.496868	-1.690642
C	-2.640060	-1.557730	-1.236553
H	-1.650292	-1.385247	3.114429
H	1.483215	0.081335	2.381564
H	-3.359006	0.184289	2.802003
H	-4.111670	-0.721774	-2.657313
O	2.863004	-1.311540	0.543615
C	1.647681	-0.489250	-2.336537
H	2.280794	0.404996	-2.358145
H	2.275862	-1.342595	-2.624935
H	0.869192	-0.358956	-3.097625
C	-1.333877	-1.129790	-1.948773
H	-1.064076	-1.769722	-2.799667
H	-0.796552	0.112294	3.514665
H	-2.266771	1.444487	2.258275
O	-5.238826	0.166075	1.321233
O	-4.715648	-0.307067	-0.769406
C	-2.396797	1.169047	-0.315906
H	-1.305754	1.248848	-0.220928
C	-2.703698	0.708855	-1.765245
H	-3.096171	1.536758	-2.369648
H	-2.937543	-2.585308	-1.470220
H	-2.919588	-2.078591	0.878911
C	-3.007062	2.543425	-0.053473

H	-2.841711	2.888471	0.976229
H	-2.554850	3.283932	-0.729915
H	-4.093443	2.548947	-0.239534
O	-1.586373	0.185525	-2.449368
O	-0.546765	-2.910545	0.516923
C	-0.817561	-3.588647	1.716454
H	-0.592321	-4.648366	1.535381
H	-0.178483	-3.236942	2.545725
H	-1.875805	-3.511936	2.025495
C	2.622170	1.354311	0.081300
H	3.239128	1.112777	-0.794376
B	3.917270	-1.846016	-0.404836
F	4.896251	-2.379067	0.410451
F	4.419239	-0.777841	-1.151568
F	3.355682	-2.813739	-1.223391
N	3.977826	1.147273	2.099308
N	3.400598	1.296671	1.152944
Si	1.522199	2.923686	-0.059801
C	0.399563	3.008010	1.434408
H	-0.296474	2.160471	1.485670
H	0.990046	3.021996	2.365329
H	-0.188595	3.939963	1.401048
C	2.688667	4.390887	-0.053032
H	3.399925	4.339418	-0.893050
H	2.120912	5.331845	-0.145784
H	3.267216	4.438858	0.884133
C	0.629537	2.809486	-1.695811
H	-0.050524	3.672196	-1.794386
H	1.352409	2.864768	-2.525776
H	0.038419	1.890516	-1.820205

65

TS1a-B'_4 Eopt -1992.369125

C	1.771785	-0.591336	0.407801
C	0.962842	-0.153762	1.576794
C	-0.314145	-0.521818	1.689709
C	-0.866471	-1.467696	0.651531
C	-0.259902	-1.210963	-0.709523
C	1.022932	-0.856629	-0.873836
C	-1.272850	-0.183257	2.781223
C	-2.385607	-1.320644	0.436682
C	-2.888581	0.116591	0.771471
C	-2.544087	0.497106	2.224262
C	-4.388570	0.047137	0.543217
C	-3.619850	-0.581343	-1.592033
C	-2.641162	-1.627859	-1.053039
H	-1.552144	-1.131581	3.269534
H	1.475655	0.405371	2.363447
H	-3.398561	0.241322	2.864647
H	-4.063456	-0.859526	-2.554412
O	2.856739	-1.202589	0.734633
C	1.634442	-0.668390	-2.233664
H	2.725098	-0.754620	-2.213901
H	1.246721	-1.422355	-2.932282
H	1.372215	0.319861	-2.638861
C	-1.322120	-1.280505	-1.783477
H	-1.067825	-1.969611	-2.601765
H	-0.801149	0.449666	3.545102
H	-2.429722	1.586602	2.293397
O	-5.259510	0.277558	1.338177
O	-4.704332	-0.321151	-0.710821
C	-2.364343	1.142350	-0.294029
H	-1.273267	1.201461	-0.181154
C	-2.653568	0.600180	-1.719796
H	-3.020656	1.397155	-2.379322
H	-2.967566	-2.659194	-1.224603
H	-2.910038	-2.023352	1.100088

C	-2.942608	2.544920	-0.126849
H	-2.485088	3.222540	-0.862831
H	-4.031395	2.560950	-0.297692
H	-2.751334	2.962971	0.870645
O	-1.535558	0.015931	-2.352802
O	-0.514129	-2.747489	1.161654
C	-0.822214	-3.869492	0.375396
H	-0.368131	-4.737569	0.872094
H	-1.909186	-4.049524	0.298412
H	-0.396907	-3.796807	-0.641756
C	2.583591	1.382133	-0.139506
H	3.072190	1.096508	-1.081043
B	3.859118	-1.929680	-0.139121
F	4.524356	-0.973146	-0.912444
F	3.198676	-2.852079	-0.935733
F	4.726816	-2.545864	0.739008
N	4.207167	1.292276	1.679224
N	3.505845	1.391709	0.814481
Si	1.495607	2.966702	-0.248436
C	2.690566	4.408263	-0.347433
H	3.358042	4.316216	-1.219282
H	2.136861	5.357356	-0.441910
H	3.316120	4.474476	0.558037
C	0.518320	2.822301	-1.833186
H	-0.083858	1.904209	-1.898508
H	-0.158559	3.688155	-1.922219
H	1.199474	2.846875	-2.699370
C	0.444806	3.146623	1.289577
H	1.074539	3.159084	2.194300
H	-0.087519	4.111517	1.244980
H	-0.299269	2.346768	1.402024

65

TS1a-B'_5 Eopt -1992.358750

C	1.624928	-0.729865	-0.288253
C	1.009688	-0.751964	1.074601
C	-0.289725	-1.043317	1.191804
C	-1.037260	-1.435142	-0.060111
C	-0.576212	-0.621852	-1.250105
C	0.712896	-0.300944	-1.411126
C	-1.086932	-1.177208	2.447529
C	-2.557670	-1.202010	0.012518
C	-2.933223	-0.058872	1.001149
C	-2.396890	-0.356324	2.413370
C	-4.449824	-0.013627	0.962182
C	-3.968331	0.358602	-1.314403
C	-3.001323	-0.823300	-1.416804
H	-1.335860	-2.248094	2.537951
H	1.634447	-0.615082	1.957745
H	-3.172242	-0.895544	2.973653
H	-4.535703	0.534694	-2.235081
O	2.511422	-1.590466	-0.656531
C	1.286683	0.328240	-2.641061
H	0.615401	0.199515	-3.500100
H	1.448053	1.408596	-2.513624
H	2.255909	-0.131454	-2.885888
C	-1.756843	-0.197230	-2.092104
H	-1.649865	-0.454083	-3.156022
H	-0.487264	-0.912725	3.328969
H	-2.243281	0.590865	2.946147
O	-5.209645	-0.148960	1.883057
O	-4.927404	0.210006	-0.275467
C	-2.471941	1.325589	0.431166
H	-1.375636	1.317195	0.417246
C	-2.950260	1.469568	-1.037674
H	-3.339082	2.477777	-1.230576
H	-3.408691	-1.673586	-1.974284

H	-3.044094	-2.122807	0.365315
C	-2.933405	2.520041	1.261871
H	-2.591627	2.465477	2.304499
H	-2.527053	3.446537	0.829113
H	-4.031613	2.614751	1.270192
O	-1.947245	1.218284	-1.999344
O	-0.712031	-2.813548	-0.207314
C	-1.209304	-3.489636	-1.332284
H	-0.753369	-4.488946	-1.325911
H	-2.306469	-3.614767	-1.304077
H	-0.929956	-2.992814	-2.279053
C	2.894574	1.008567	-0.103997
H	3.505332	0.718675	-0.969465
B	3.529291	-2.374943	0.143461
F	3.133736	-2.501593	1.467218
F	4.724876	-1.661802	0.055654
F	3.628785	-3.605290	-0.476688
N	4.028696	0.454100	1.992078
N	3.567273	0.706513	1.007366
Si	2.352243	2.873537	-0.105165
C	3.152832	3.633318	-1.615919
H	2.922986	4.711194	-1.650682
H	4.248196	3.520876	-1.576787
H	2.795335	3.184197	-2.555300
C	0.496638	3.041508	-0.111673
H	0.239506	4.109213	-0.215737
H	0.000324	2.489165	-0.922925
H	0.093603	2.696143	0.852464
C	3.039177	3.630009	1.465499
H	2.765788	4.697641	1.500224
H	2.622075	3.153815	2.368229
H	4.138220	3.565210	1.514741

65

TS1a-B'_6 Eopt -1992.368583

C	-1.554325	-0.810512	0.435571
C	-1.043400	-0.508218	-0.932306
C	0.241427	-0.715830	-1.237146
C	1.098501	-1.449252	-0.236426
C	0.724172	-1.118579	1.184693
C	-0.544440	-0.904341	1.546643
C	0.949328	-0.290739	-2.485612
C	2.589183	-1.108823	-0.341099
C	2.837160	0.355653	-0.807302
C	2.156403	0.624311	-2.165270
C	4.351204	0.448373	-0.892672
C	4.101633	-0.152606	1.374477
C	3.151329	-1.316666	1.078399
H	1.313625	-1.189892	-3.006613
H	-1.747466	-0.096225	-1.654452
H	2.905521	0.514783	-2.960710
H	4.760665	-0.335432	2.230443
O	-2.609030	-1.540556	0.614280
C	-1.009594	-0.802360	2.969565
H	-0.324770	-1.334959	3.642662
H	-1.060542	0.241007	3.319347
H	-2.009764	-1.246712	3.077929
C	1.967096	-1.052962	2.041142
H	1.937211	-1.727568	2.908509
H	0.260726	0.219958	-3.173235
H	1.829682	1.671507	-2.200607
O	5.015162	0.725127	-1.855251
O	4.950337	0.171658	0.279206
C	2.441297	1.382089	0.313865
H	1.347093	1.354790	0.426333
C	3.059682	0.934736	1.666037
H	3.467490	1.793281	2.215586

H	3.601498	-2.305805	1.213394
H	3.070781	-1.775064	-1.070875
C	2.861653	2.813359	-0.014837
H	2.459642	3.159448	-0.976832
H	2.493835	3.497763	0.764282
H	3.958308	2.918068	-0.049617
O	2.156513	0.279190	2.526711
O	0.912499	-2.862411	-0.300181
C	0.960944	-3.478610	-1.561119
H	0.899608	-4.561113	-1.384188
H	0.107759	-3.184852	-2.197266
H	1.901934	-3.274185	-2.103859
C	-2.221270	1.174728	0.865039
H	-1.513832	1.479420	1.649319
B	-3.604360	-1.889116	-0.473953
F	-2.958350	-2.626941	-1.457011
F	-4.107913	-0.698099	-1.000057
F	-4.595339	-2.623830	0.146266
N	-4.322884	0.365830	1.799503
N	-3.375328	0.827659	1.433735
Si	-2.384245	2.610081	-0.428509
C	-3.467936	2.164292	-1.880031
H	-3.022743	1.405948	-2.539672
H	-4.452740	1.799651	-1.552853
H	-3.624816	3.079696	-2.475376
C	-3.164318	4.016612	0.534987
H	-3.249324	4.911022	-0.104529
H	-4.178318	3.754124	0.879027
H	-2.560747	4.286158	1.416555
C	-0.636253	3.035378	-0.921761
H	-0.027914	3.290197	-0.040442
H	-0.152578	2.207376	-1.457266
H	-0.647396	3.913199	-1.588693

65

TS1a-B'_7 Eopt -1992.368526

C	-1.553010	-0.806153	0.435754
C	-1.040110	-0.514362	-0.933974
C	0.244417	-0.727638	-1.236166
C	1.101235	-1.452396	-0.228879
C	0.724932	-1.112217	1.189324
C	-0.543942	-0.895252	1.548358
C	0.954465	-0.312066	-2.486603
C	2.591460	-1.109396	-0.334276
C	2.836299	0.352798	-0.809558
C	2.154363	0.612894	-2.168794
C	4.350051	0.448787	-0.894363
C	4.099873	-0.138459	1.376521
C	3.152575	-1.306686	1.087103
H	1.326235	-1.214237	-2.996913
H	-1.741255	-0.105614	-1.661381
H	2.904360	0.507730	-2.964013
H	4.758658	-0.314244	2.234153
O	-2.607776	-1.535787	0.617875
C	-1.010400	-0.785506	2.970368
H	-1.062696	0.259630	3.314658
H	-2.010183	-1.230375	3.080296
H	-0.325742	-1.313828	3.646966
C	1.966694	-1.039577	2.046907
H	1.937144	-1.708967	2.918301
H	0.266337	0.188082	-3.182475
H	1.818252	1.657009	-2.205348
O	5.014233	0.721630	-1.857892
O	4.948715	0.180780	0.279837
C	2.437266	1.385342	0.304746
H	1.342854	1.355758	0.414990
C	3.055012	0.948209	1.660548

H	3.460218	1.811273	2.204959
H	3.605030	-2.293805	1.229024
H	3.075364	-1.779180	-1.059267
C	2.855601	2.815309	-0.032430
H	2.462479	3.151584	-1.001586
H	2.477895	3.505221	0.737093
H	3.952185	2.923404	-0.057456
O	2.152915	0.295863	2.524662
O	0.918270	-2.866331	-0.282184
C	0.970726	-3.492447	-1.537962
H	0.909381	-4.573542	-1.352584
H	0.119305	-3.204199	-2.178957
H	1.913318	-3.291900	-2.079395
C	-2.215054	1.178792	0.858124
H	-1.506464	1.484389	1.640834
B	-3.596404	-1.900521	-0.470862
F	-4.100893	-0.718336	-1.014763
F	-4.588416	-2.630612	0.153392
F	-2.942678	-2.649214	-1.440770
N	-4.319293	0.373175	1.789545
N	-3.370071	0.834326	1.427238
Si	-2.391258	2.613038	-0.435470
C	-3.592768	2.184664	-1.795775
H	-4.573794	1.898918	-1.386837
H	-3.735357	3.082560	-2.420492
H	-3.244625	1.369151	-2.444495
C	-3.049306	4.057034	0.563049
H	-2.375764	4.311228	1.397236
H	-3.144819	4.948254	-0.079481
H	-4.045843	3.836541	0.979640
C	-0.667515	2.977235	-1.048283
H	-0.255849	2.147575	-1.638245
H	-0.692818	3.873452	-1.689948
H	0.011242	3.186448	-0.207435

65

TS1a-B'_8 Eopt -1992.367760

C	-1.513808	-0.966382	0.361836
C	-1.005072	-0.705370	-1.012759
C	0.283382	-0.872844	-1.325625
C	1.191589	-1.558631	-0.340650
C	0.778844	-1.335590	1.088099
C	-0.498540	-1.183026	1.452288
C	0.981302	-0.337577	-2.537294
C	2.632546	-1.045475	-0.409679
C	2.708574	0.469965	-0.767566
C	1.962175	0.769923	-2.088725
C	4.204309	0.717164	-0.858463
C	4.042723	-0.059772	1.367868
C	3.215720	-1.293083	0.992040
H	1.542334	-1.141247	-3.037058
H	-1.715616	-0.302947	-1.729984
H	2.697457	0.949206	-2.884990
H	4.729513	-0.231752	2.203983
O	-2.643841	-1.589690	0.544812
C	-0.957032	-1.170651	2.881653
H	-1.921482	-1.690227	2.976969
H	-0.228444	-1.678373	3.526800
H	-1.078549	-0.149791	3.278653
C	2.009724	-1.223174	1.961397
H	2.045760	-1.961509	2.774771
H	0.267550	0.066987	-3.268054
H	1.393741	1.703743	-1.972356
O	4.827574	1.116200	-1.804902
O	4.839925	0.424825	0.291471
C	2.234869	1.375103	0.425694
H	1.145306	1.254435	0.532246

C	2.896215	0.889383	1.742426
H	3.216056	1.739958	2.358539
H	3.766850	-2.236896	1.059076
H	3.186913	-1.594738	-1.183014
C	2.544616	2.853623	0.193162
H	2.143993	3.221518	-0.761787
H	2.104971	3.459875	0.999030
H	3.629427	3.047026	0.196860
O	2.068191	0.080978	2.547222
O	1.167593	-2.978787	-0.470686
C	1.334631	-3.531177	-1.750812
H	1.371317	-4.621343	-1.619780
H	0.489522	-3.293095	-2.420719
H	2.274945	-3.214866	-2.238218
C	-1.915571	1.007503	0.821294
H	-1.040614	1.258900	1.437634
B	-3.749856	-1.635934	-0.476583
F	-4.841181	-2.191263	0.163175
F	-3.351110	-2.399418	-1.564670
F	-4.026552	-0.317972	-0.882913
N	-3.877926	0.428205	2.153376
N	-2.965293	0.780908	1.620189
Si	-2.318839	2.481516	-0.407115
C	-1.188914	3.843951	0.204072
H	-0.130230	3.571598	0.071059
H	-1.369775	4.770074	-0.366178
H	-1.357775	4.062712	1.270663
C	-1.977523	2.155419	-2.212487
H	-0.930669	1.881431	-2.407592
H	-2.642129	1.390668	-2.639773
H	-2.176873	3.104287	-2.740037
C	-4.118159	2.917317	-0.144928
H	-4.381433	3.762609	-0.801918
H	-4.775404	2.072161	-0.400622
H	-4.326659	3.224348	0.892667

65

TS1a-B_1 Eopt -1992.372486

C	1.397316	0.887375	-0.123893
C	0.642810	1.250839	1.102913
C	-0.448281	0.566713	1.467313
C	-0.793610	-0.702159	0.727541
C	-0.377125	-0.655658	-0.719406
C	0.699384	0.025446	-1.127020
C	-1.457700	0.989413	2.485709
C	-2.304580	-0.959398	0.676244
C	-3.136200	0.359768	0.604826
C	-2.793715	1.304993	1.778206
C	-4.568521	-0.144471	0.652975
C	-3.796990	-1.263645	-1.276670
C	-2.529675	-1.792339	-0.596676
H	-1.612404	0.175338	3.210682
H	0.963500	2.142646	1.640543
H	-3.609166	1.276470	2.513550
H	-4.212341	-1.953018	-2.019893
O	2.217965	1.748724	-0.634133
C	1.178609	0.102680	-2.541585
H	0.641424	-0.604339	-3.185396
H	1.031336	1.116942	-2.942952
H	2.257019	-0.108146	-2.601518
C	-1.403635	-1.368039	-1.572083
H	-0.975128	-2.202174	-2.146198
H	-1.113998	1.869569	3.045833
H	-2.743564	2.336003	1.403698
O	-5.413005	0.119536	1.465822
O	-4.848773	-0.986611	-0.358167
C	-3.009963	1.054529	-0.799090

H	-1.987857	1.451031	-0.891684
C	-3.211870	0.001025	-1.919373
H	-3.828438	0.403342	-2.733570
H	-2.545627	-2.869536	-0.399445
H	-2.623181	-1.499578	1.578720
C	-3.996451	2.208925	-0.972828
H	-5.040060	1.854810	-0.993228
H	-3.910455	2.957765	-0.173446
H	-3.804270	2.719560	-1.928015
O	-2.014998	-0.468763	-2.496204
O	-0.107483	-1.836581	1.263393
C	-0.178389	-2.071610	2.648165
H	0.334518	-3.025742	2.829659
H	0.331947	-1.284650	3.231314
H	-1.215849	-2.167003	3.013367
C	2.730370	-0.613515	0.727918
H	2.225842	-0.748665	1.691711
B	2.447063	3.186260	-0.193536
F	1.247897	3.868792	-0.359185
F	3.432896	3.672374	-1.029254
F	2.850425	3.225477	1.140298
N	4.564883	0.987445	0.776913
N	3.764624	0.209990	0.852411
Si	3.239631	-2.252742	-0.171881
C	4.632795	-1.795261	-1.338055
H	5.527360	-1.446028	-0.797338
H	4.925677	-2.681572	-1.924709
H	4.333436	-1.007971	-2.048359
C	3.849647	-3.363308	1.209824
H	4.223677	-4.316502	0.800492
H	4.669490	-2.890113	1.773888
H	3.035880	-3.595389	1.916005
C	1.828695	-3.082877	-1.067858
H	1.550990	-2.566128	-1.996947
H	2.161316	-4.100884	-1.333466
H	0.944089	-3.157765	-0.419571

65

TS1a-B_2 Eopt -1992.375158

C	-1.215665	0.677564	0.275531
C	-0.519699	1.004862	-0.992843
C	0.588865	0.349524	-1.363516
C	1.046086	-0.836314	-0.544502
C	0.693109	-0.690548	0.914600
C	-0.393413	-0.023464	1.321306
C	1.526594	0.757113	-2.454158
C	2.573572	-0.988714	-0.544167
C	3.321671	0.380250	-0.615672
C	2.868087	1.208415	-1.838014
C	4.779673	-0.040689	-0.697945
C	4.169327	-1.045628	1.348654
C	2.907857	-1.700914	0.776602
H	1.703590	-0.099869	-3.122389
H	-0.891453	1.853834	-1.566982
H	3.650280	1.172512	-2.608280
H	4.660750	-1.647479	2.120880
O	-2.074855	1.519311	0.753517
C	-0.762710	0.196178	2.753844
H	-1.829825	0.007232	2.922097
H	-0.174015	-0.445209	3.420831
H	-0.578472	1.244823	3.035338
C	1.802741	-1.265307	1.770197
H	1.456719	-2.073224	2.430551
H	1.101133	1.566711	-3.062313
H	2.767852	2.260654	-1.540754
O	5.567614	0.209062	-1.569711
O	5.158316	-0.782118	0.358904

C	3.220385	1.177349	0.734274
H	2.181948	1.522077	0.849560
C	3.537006	0.230564	1.920041
H	4.163566	0.731303	2.669560
H	2.981775	-2.787721	0.663328
H	2.891168	-1.577488	-1.415571
C	4.143080	2.395319	0.766491
H	5.205781	2.103135	0.762261
H	3.976429	3.070614	-0.084035
H	3.964917	2.970135	1.687329
O	2.396859	-0.258177	2.588161
O	0.410010	-2.051472	-0.948410
C	0.516025	-2.451529	-2.294600
H	1.561530	-2.603136	-2.612955
H	-0.007919	-3.413427	-2.375084
H	0.038217	-1.736948	-2.986673
C	-2.437324	-1.020930	-0.312519
H	-1.718224	-1.811424	-0.057989
B	-2.931097	2.449054	-0.066591
F	-4.031856	2.746666	0.719977
F	-3.317676	1.790562	-1.237718
F	-2.201131	3.591688	-0.370440
N	-2.457966	-0.698224	-2.733255
N	-2.462642	-0.904686	-1.634541
Si	-4.203961	-1.226600	0.452783
C	-4.328475	-0.434419	2.137089
H	-3.709530	-0.952976	2.884669
H	-4.055779	0.629233	2.107338
H	-5.378642	-0.511926	2.466169
C	-5.452892	-0.486241	-0.725082
H	-5.374273	0.609573	-0.751041
H	-5.343184	-0.867941	-1.753055
H	-6.465032	-0.752826	-0.377945
C	-4.430742	-3.083697	0.587537
H	-3.665610	-3.531713	1.242378
H	-5.420046	-3.316692	1.015698
H	-4.364974	-3.569883	-0.399298

65

TS1a-B_3 Eopt -1992.375681

C	1.223886	0.652807	-0.342602
C	0.513090	1.126454	0.866447
C	-0.592666	0.510419	1.301658
C	-1.040730	-0.764452	0.626283
C	-0.682882	-0.780084	-0.843444
C	0.407676	-0.157172	-1.311868
C	-1.474865	0.937207	2.423386
C	-2.572682	-0.940685	0.646461
C	-3.325748	0.425678	0.683630
C	-2.890809	1.264051	1.902799
C	-4.790343	0.026209	0.760936
C	-4.179345	-1.036844	-1.248986
C	-2.936034	-1.696937	-0.646480
H	-1.526806	0.096130	3.134735
H	0.898355	2.011479	1.371459
H	-3.617881	1.116909	2.712403
H	-4.668778	-1.653459	-2.010886
O	2.102382	1.423646	-0.899585
C	0.775042	-0.065282	-2.758645
H	1.842610	-0.271706	-2.907773
H	0.184649	-0.760909	-3.367199
H	0.595065	0.955442	-3.130516
C	-1.825560	-1.339610	-1.663048
H	-1.527830	-2.179548	-2.307696
H	-1.054301	1.798209	2.959531
H	-2.930191	2.329554	1.641638
O	-5.580967	0.311792	1.619153

O	-5.174237	-0.732992	-0.280078
C	-3.191471	1.184491	-0.686594
H	-2.143699	1.500203	-0.801921
C	-3.518265	0.209333	-1.848878
H	-4.129453	0.702812	-2.615719
H	-3.048288	-2.775402	-0.491338
H	-2.855793	-1.501516	1.549002
C	-4.082478	2.422865	-0.769653
H	-3.899066	3.127882	0.052483
H	-3.887981	2.955482	-1.712273
H	-5.152457	2.158715	-0.756135
O	-2.386868	-0.324864	-2.498311
O	-0.369859	-1.775810	1.375020
C	-0.570094	-3.113828	0.991170
H	0.126344	-3.720515	1.585854
H	-1.594819	-3.464108	1.202743
H	-0.349701	-3.280708	-0.078809
C	2.430727	-0.983157	0.436919
H	1.773408	-1.819395	0.168259
B	2.935195	2.448522	-0.174179
F	2.212623	3.630678	-0.072001
F	4.078695	2.620640	-0.939493
F	3.253931	1.959202	1.095626
N	2.232206	-0.525536	2.826340
N	2.344586	-0.796869	1.748382
Si	4.240687	-1.199999	-0.208039
C	4.359090	-0.690611	-1.998894
H	3.731919	-1.321370	-2.647518
H	4.082309	0.363917	-2.134390
H	5.405889	-0.817900	-2.322896
C	5.419831	-0.226948	0.865892
H	5.224367	-0.370310	1.940824
H	6.447018	-0.573368	0.663682
H	5.365190	0.847251	0.644467
C	4.560369	-3.041448	-0.032253
H	5.575547	-3.287456	-0.386167
H	4.482062	-3.363973	1.018868
H	3.843906	-3.631717	-0.626541

65

TS1a-B_4 Eopt -1992.376885

C	1.291117	0.957458	-0.414844
C	0.846028	0.650766	0.964569
C	-0.194550	-0.155660	1.193919
C	-0.824635	-0.904360	0.047911
C	-0.700827	-0.156218	-1.261701
C	0.320220	0.677802	-1.514984
C	-0.868670	-0.390324	2.502801
C	-2.343334	-1.092179	0.235436
C	-2.982874	0.056997	1.074741
C	-2.305545	0.172679	2.456776
C	-4.442594	-0.353682	1.186688
C	-4.223332	-0.249031	-1.155376
C	-2.951838	-1.101768	-1.180309
H	-0.899987	-1.478280	2.679216
H	1.345585	1.184767	1.770105
H	-2.924853	-0.344930	3.201418
H	-4.865546	-0.407196	-2.028796
O	2.012032	2.017339	-0.624582
C	0.498758	1.437390	-2.791814
H	1.525794	1.315947	-3.170085
H	-0.204130	1.097275	-3.561855
H	0.345949	2.514908	-2.629538
C	-1.987662	-0.263426	-2.054187
H	-1.846425	-0.663232	-3.068671
H	-0.309283	0.064734	3.331113
H	-2.275352	1.228268	2.756974

O	-5.070133	-0.555156	2.191153
O	-5.033756	-0.491130	-0.013039
C	-3.008566	1.404457	0.265036
H	-1.971721	1.754007	0.151233
C	-3.576339	1.140427	-1.155714
H	-4.261205	1.942106	-1.460674
H	-3.114842	-2.113442	-1.566847
H	-2.528904	-2.032609	0.773228
C	-3.814308	2.499578	0.962382
H	-4.884731	2.244194	1.024252
H	-3.456054	2.698512	1.981667
H	-3.733120	3.437676	0.393658
O	-2.599177	1.022956	-2.166179
O	-0.116675	-2.142487	0.025826
C	-0.598226	-3.176328	-0.798783
H	-1.560121	-3.582712	-0.443044
H	-0.707798	-2.866062	-1.852218
H	0.147450	-3.982171	-0.756636
C	2.767950	-0.555907	-0.796557
H	3.437272	0.155428	-1.297459
B	3.121878	2.621162	0.188520
F	3.428110	3.816943	-0.434764
F	4.215429	1.745455	0.148972
F	2.733689	2.835746	1.509990
N	1.689950	-1.988740	-2.452274
N	2.238649	-1.356791	-1.710372
Si	3.629441	-1.491864	0.666130
C	3.432928	-0.630612	2.312058
H	3.601120	0.452386	2.233484
H	2.445883	-0.809928	2.761913
H	4.195067	-1.045677	2.993595
C	2.892767	-3.210818	0.727932
H	3.012882	-3.752329	-0.224629
H	3.398098	-3.798257	1.512330
H	1.820174	-3.154828	0.968738
C	5.435545	-1.530784	0.170275
H	6.027432	-2.082047	0.919754
H	5.576144	-2.023903	-0.805147
H	5.838002	-0.507834	0.102281

65

TS1a-B_5 Eopt -1992.371641

C	-1.284657	0.876896	0.118318
C	-0.660974	0.869250	-1.223964
C	0.386811	0.085668	-1.497736
C	0.807701	-0.991831	-0.532654
C	0.404324	-0.694495	0.889781
C	-0.661701	0.057190	1.201834
C	1.309098	0.220159	-2.661850
C	2.338579	-1.154601	-0.470880
C	3.076174	0.186794	-0.762890
C	2.669061	0.743803	-2.145783
C	4.546969	-0.192480	-0.708637
C	3.875490	-0.839739	1.454692
C	2.661629	-1.630836	0.958245
H	1.438563	-0.770066	-3.127192
H	-0.997768	1.624291	-1.933787
H	3.454334	0.499208	-2.873615
H	4.345168	-1.280944	2.340741
O	-1.758317	1.995245	0.573000
C	-1.108370	0.332179	2.604460
H	-0.820114	1.345824	2.919243
H	-2.204381	0.269943	2.680205
H	-0.672324	-0.388333	3.307524
C	1.509713	-1.098739	1.845741
H	1.189794	-1.799739	2.630294
H	0.907219	0.901490	-3.423516

H	2.619247	1.839546	-2.093869
O	5.366177	-0.071503	-1.579009
O	4.900232	-0.719944	0.476719
C	2.883643	1.204573	0.421288
H	1.831144	1.523694	0.432233
C	3.178557	0.488382	1.768476
H	3.755172	1.138348	2.439249
H	2.794462	-2.715699	1.025938
H	2.657472	-1.881686	-1.231157
C	3.760700	2.447313	0.278297
H	4.832842	2.203373	0.354998
H	3.599186	2.963490	-0.678057
H	3.527080	3.160033	1.082938
O	2.035018	0.069070	2.481506
O	0.163298	-2.159213	-1.031321
C	0.456185	-3.396713	-0.431365
H	-0.263657	-4.120277	-0.838422
H	1.473024	-3.750813	-0.673068
H	0.337429	-3.377011	0.665799
C	-3.158197	-0.147244	-0.384587
H	-3.215300	0.247574	-1.408652
B	-2.159537	3.206803	-0.244872
F	-2.833740	4.037221	0.627984
F	-2.993910	2.783474	-1.284092
F	-1.014398	3.810007	-0.745160
N	-4.484264	1.253816	1.109173
N	-3.945413	0.591786	0.388134
Si	-3.475405	-2.043604	-0.259154
C	-5.336012	-2.246582	-0.111175
H	-5.596524	-3.317301	-0.063074
H	-5.726532	-1.768702	0.802667
H	-5.859011	-1.810036	-0.977444
C	-2.861619	-2.752519	-1.871734

H	-2.967947	-3.849823	-1.870249
H	-3.456565	-2.357121	-2.710932
H	-1.803836	-2.499188	-2.032849
C	-2.668205	-2.743663	1.274456
H	-3.112124	-2.300014	2.180462
H	-2.841064	-3.831967	1.317502
H	-1.586120	-2.562997	1.297696

65

TS1a-B_6 Eopt -1992.376392

C	1.223944	0.698497	-0.351116
C	0.516607	1.259783	0.822115
C	-0.585168	0.670463	1.307860
C	-1.012192	-0.669071	0.751598
C	-0.623059	-0.842980	-0.694620
C	0.464994	-0.262591	-1.215789
C	-1.552175	1.282216	2.270178
C	-2.535810	-0.842931	0.755675
C	-3.300161	0.495661	0.514157
C	-2.883164	1.572394	1.541039
C	-4.754972	0.079096	0.653765
C	-4.083100	-1.336462	-1.112563
C	-2.830065	-1.832682	-0.383804
H	-1.736126	0.583791	3.101112
H	0.861892	2.221809	1.201542
H	-3.683223	1.690813	2.284237
H	-4.549079	-2.098972	-1.746412
O	2.074108	1.420368	-0.992844
C	0.885794	-0.381484	-2.647238
H	0.676202	0.555627	-3.186134
H	1.966716	-0.559470	-2.731429
H	0.350838	-1.194967	-3.152441
C	-1.705160	-1.610422	-1.424849
H	-1.335860	-2.537642	-1.885426

H	-1.151027	2.209512	2.700797
H	-2.785306	2.538198	1.027773
O	-5.566717	0.497853	1.434028
O	-5.098848	-0.879239	-0.225847
C	-3.171668	0.981865	-0.974447
H	-2.133697	1.307640	-1.137989
C	-3.448739	-0.205261	-1.932871
H	-4.060603	0.110922	-2.787536
H	-2.896334	-2.868877	-0.035817
H	-2.861566	-1.233932	1.729191
C	-4.102251	2.151632	-1.292552
H	-5.162619	1.854280	-1.249978
H	-3.961460	2.997065	-0.605051
H	-3.904627	2.515251	-2.311786
O	-2.287057	-0.810739	-2.453636
O	-0.389489	-1.770799	1.419529
C	-0.432978	-1.812074	2.827537
H	0.042152	-2.756071	3.125899
H	0.127190	-0.978404	3.285656
H	-1.464704	-1.808469	3.219642
C	2.456887	-0.824399	0.710491
H	1.619281	-1.522383	0.862220
B	2.937386	2.538913	-0.447682
F	3.774767	2.900779	-1.483967
F	3.650455	2.041379	0.641697
F	2.131125	3.605407	-0.061098
N	2.920257	0.322995	2.814361
N	2.724387	-0.238624	1.866395
Si	3.950228	-1.533265	-0.261392
C	5.210106	-2.071064	1.019605
H	6.087745	-2.521077	0.526405
H	5.566446	-1.210366	1.609835
H	4.791816	-2.815974	1.715655
C	3.231235	-3.003460	-1.172298
H	2.418190	-2.708307	-1.853510
H	4.012799	-3.495580	-1.774299
H	2.830785	-3.746322	-0.463695
C	4.734686	-0.296155	-1.418624
H	4.024168	0.133891	-2.137347
H	5.187530	0.533276	-0.856345
H	5.535220	-0.805775	-1.981175

65

TS1a-B_7 Eopt -1992.376114

C	1.233140	0.712411	-0.311698
C	0.507076	1.268026	0.850356
C	-0.584774	0.659379	1.331499
C	-1.003627	-0.682687	0.774983
C	-0.596750	-0.854049	-0.669995
C	0.498141	-0.268101	-1.175536
C	-1.507449	1.178969	2.379834
C	-2.534137	-0.867463	0.765413
C	-3.291386	0.489538	0.630409
C	-2.896657	1.457487	1.765309
C	-4.756095	0.090130	0.703014
C	-4.075073	-1.179305	-1.160184
C	-2.851712	-1.762952	-0.448209
H	-1.595177	0.406240	3.161369
H	0.860185	2.214109	1.260967
H	-3.658456	1.410249	2.554786
H	-4.536973	-1.877908	-1.866477
O	2.076836	1.433899	-0.960066
C	0.913872	-0.354622	-2.611515
H	0.673254	0.583459	-3.135306
H	1.999158	-0.498889	-2.703348
H	0.401429	-1.176302	-3.126546
C	-1.707852	-1.514123	-1.459287

H	-1.385202	-2.417557	-1.997059
H	-1.110131	2.087033	2.852219
H	-2.908442	2.486401	1.382591
O	-5.575474	0.459718	1.499884
O	-5.102427	-0.778608	-0.262966
C	-3.114991	1.096748	-0.809205
H	-2.066298	1.409917	-0.922195
C	-3.396111	-0.001834	-1.868907
H	-3.979941	0.400569	-2.706738
H	-2.967982	-2.818365	-0.180017
H	-2.844323	-1.329560	1.713600
C	-4.011595	2.309528	-1.054490
H	-5.079325	2.035962	-1.051024
H	-3.863199	3.098907	-0.305084
H	-3.786862	2.742089	-2.040583
O	-2.240856	-0.597230	-2.416747
O	-0.360250	-1.619519	1.640124
C	-0.543606	-2.991487	1.375778
H	0.129305	-3.533939	2.053495
H	-1.575538	-3.322313	1.580570
H	-0.277447	-3.256688	0.337145
C	2.484327	-0.745845	0.827430
H	1.633648	-1.395953	1.077375
B	2.908265	2.599174	-0.468874
F	3.686378	2.972136	-1.547477
F	3.688033	2.154886	0.595686
F	2.071556	3.636093	-0.069517
N	3.065633	0.539928	2.819978
N	2.815883	-0.079171	1.922190
Si	3.904723	-1.551435	-0.168148
C	5.160893	-2.172733	1.078820
H	5.998751	-2.670896	0.563087
H	5.580992	-1.340266	1.667598
H	4.713377	-2.896702	1.778789
C	3.078727	-2.980771	-1.054213
H	2.259574	-2.650045	-1.710487
H	3.814047	-3.515421	-1.677850
H	2.667621	-3.699978	-0.327323
C	4.726934	-0.343086	-1.331209
H	4.022276	0.125649	-2.031565
H	5.221278	0.461313	-0.766173
H	5.498242	-0.875259	-1.912991

65

TS1a-B_8 Eopt -1992.375453

C	-1.283069	1.065033	0.281736
C	-0.809083	0.675110	-1.067245
C	0.228929	-0.150576	-1.220675
C	0.811655	-0.835630	-0.011892
C	0.691807	0.013597	1.235531
C	-0.314229	0.885365	1.405275
C	0.919324	-0.493335	-2.496058
C	2.326966	-1.084955	-0.153598
C	3.020153	-0.019561	-1.060375
C	2.374201	0.019540	-2.461289
C	4.466804	-0.484754	-1.112608
C	4.209923	-0.202445	1.210559
C	2.909375	-1.008159	1.271341
H	0.915690	-1.591740	-2.597079
H	-1.300759	1.145435	-1.916863
H	2.987798	-0.572318	-3.153404
H	4.830656	-0.317699	2.105962
O	-2.019986	2.130228	0.397366
C	-0.497911	1.752091	2.610794
H	0.187218	1.466427	3.418206
H	-0.320825	2.809049	2.360465
H	-1.533051	1.683804	2.979548

C	1.958762	-0.074400	2.059986
H	1.782416	-0.392559	3.097703
H	0.388282	-0.080326	-3.364120
H	2.389679	1.050968	-2.837142
O	5.104726	-0.778868	-2.087195
O	5.031796	-0.554028	0.105359
C	3.078162	1.381729	-0.350067
H	2.052040	1.772488	-0.282028
C	3.612091	1.204264	1.096755
H	4.319361	2.002695	1.355834
H	3.030292	-1.992718	1.735613
H	2.493694	-2.066961	-0.618591
C	3.932814	2.396470	-1.108189
H	4.995424	2.104493	-1.126808
H	3.601963	2.530365	-2.147100
H	3.870569	3.376127	-0.611725
O	2.613501	1.194882	2.092429
O	0.054857	-2.044121	0.087358
C	0.499660	-3.031894	0.986163
H	-0.269405	-3.815875	1.001372
H	1.447900	-3.495130	0.665462
H	0.618518	-2.650354	2.014992
C	-2.736701	-0.426848	0.764361
H	-3.449368	0.317880	1.148928
B	-3.265814	2.496377	-0.352837
F	-4.333050	2.272813	0.524399
F	-3.427309	1.693532	-1.486329
F	-3.171536	3.832113	-0.695353
N	-1.699434	-1.607261	2.632875
N	-2.228094	-1.085453	1.797763
Si	-3.539322	-1.592411	-0.568045
C	-5.296322	-0.967059	-0.707450
H	-5.307807	0.075669	-1.057602
H	-5.861006	-1.581320	-1.428172
H	-5.816442	-1.013344	0.262704
C	-2.694583	-1.612897	-2.230916
H	-1.657556	-1.968555	-2.151081
H	-3.253860	-2.318952	-2.868888
H	-2.705641	-0.629277	-2.720008
C	-3.491323	-3.316835	0.168540
H	-2.457273	-3.680128	0.279967
H	-3.987278	-3.367426	1.151494
H	-4.015480	-4.013830	-0.506340

65

TS1a-B_9 Eopt -1992.374356

C	-1.232773	1.053987	0.160821
C	-0.794496	0.460002	-1.124260
C	0.255180	-0.369053	-1.181473
C	0.864509	-0.868696	0.103110
C	0.741797	0.130484	1.227522
C	-0.264198	1.012360	1.294504
C	0.996851	-0.767375	-2.416893
C	2.375828	-1.106618	-0.017380
C	3.067658	-0.104631	-0.992315
C	2.406916	-0.136083	-2.389416
C	4.505712	-0.594570	-1.011223
C	4.246099	-0.154222	1.293696
C	2.929767	-0.930541	1.405845
H	1.088554	-1.864195	-2.454299
H	-1.285490	0.828797	-2.023665
H	3.061659	-0.679043	-3.084401
H	4.867451	-0.217153	2.193789
O	-1.947246	2.136554	0.137280
C	-0.463215	1.998123	2.402357
H	0.235342	1.818189	3.228396
H	-0.319223	3.027217	2.040377

H	-1.493588	1.938818	2.786721
C	1.981640	0.080357	2.095761
H	1.762192	-0.159014	3.145672
H	0.461968	-0.450630	-3.322662
H	2.327218	0.890687	-2.770888
O	5.142881	-0.964794	-1.960109
O	5.062478	-0.594199	0.213564
C	3.151542	1.339298	-0.379044
H	2.133061	1.753662	-0.345703
C	3.673952	1.253355	1.079298
H	4.394204	2.054956	1.288242
H	3.015119	-1.879227	1.946240
H	2.567825	-2.116500	-0.403013
C	4.031211	2.277392	-1.204266
H	5.089274	1.969565	-1.183086
H	3.716013	2.328206	-2.255580
H	3.977521	3.295523	-0.791194
O	2.666524	1.331994	2.062420
O	0.203442	-2.037145	0.591941
C	0.136121	-3.169767	-0.239118
H	-0.277814	-3.981636	0.374089
H	-0.525199	-3.015169	-1.107871
H	1.125507	-3.495696	-0.603097
C	-2.717118	-0.336883	0.909434
H	-3.331377	0.487704	1.298884
B	-3.199341	2.465290	-0.619248
F	-4.256735	2.309155	0.283214
F	-3.382766	1.603208	-1.702166
F	-3.090645	3.777946	-1.039227
N	-1.549945	-1.456532	2.738073
N	-2.140871	-0.956538	1.932095
Si	-3.717945	-1.512526	-0.265038
C	-5.450431	-0.809868	-0.244709
H	-5.449446	0.219407	-0.633003
H	-6.115705	-1.420559	-0.877095
H	-5.865759	-0.793530	0.775611
C	-3.086212	-1.609022	-2.020080
H	-2.051833	-1.972244	-2.092231
H	-3.735240	-2.319525	-2.560632
H	-3.155579	-0.633425	-2.519671
C	-3.659210	-3.207205	0.537420
H	-2.632798	-3.601228	0.602134
H	-4.086528	-3.192940	1.553327
H	-4.249779	-3.917612	-0.064501

65

TS1b-Al'_1 Eopt -3290.914735

C	1.249668	0.029256	-0.334687
C	0.700869	-0.122457	1.038506
C	-0.410567	-0.839562	1.245766
C	-1.005696	-1.598039	0.090535
C	-0.794247	-0.892841	-1.224500
C	0.301172	-0.162997	-1.483921
C	-1.173664	-0.999442	2.519392
C	-2.515359	-1.798257	0.200580
C	-3.225359	-0.641905	0.950944
C	-2.617061	-0.458548	2.359172
C	-4.677811	-1.083199	0.993898
C	-4.313330	-1.086304	-1.339694
C	-3.019357	-1.900738	-1.250261
H	-1.211743	-2.075574	2.755412
H	1.243187	0.343696	1.865302
H	-3.266332	-0.958359	3.090480
H	-4.901745	-1.297723	-2.239307
O	2.489050	-0.223231	-0.563216
C	0.665135	0.380542	-2.831621
H	0.399090	1.443504	-2.932141

H	1.749284	0.286076	-2.993237
H	0.146954	-0.166087	-3.630046
C	-2.020471	-1.074955	-2.096298
H	-1.803105	-1.505789	-3.083759
H	-0.672382	-0.496018	3.357202
H	-2.626838	0.607436	2.622027
O	-5.366108	-1.246201	1.964736
O	-5.184570	-1.297461	-0.234167
C	-3.234244	0.661790	0.080662
H	-2.197899	1.017350	0.012457
C	-3.704317	0.320442	-1.359591
H	-4.387946	1.088773	-1.743018
H	-3.117767	-2.936964	-1.590081
H	-2.692638	-2.726751	0.759198
C	-4.092730	1.775096	0.674895
H	-3.975458	2.693390	0.080562
H	-5.163038	1.512115	0.664963
H	-3.814989	2.010519	1.711339
O	-2.656759	0.190879	-2.298308
O	-0.506188	-2.937193	0.031188
C	0.870653	-3.148184	-0.148920
H	1.009219	-4.235262	-0.222526
H	1.253074	-2.692888	-1.077823
H	1.466404	-2.784249	0.705777
C	1.398177	2.218645	-0.382998
H	1.857307	2.267299	-1.380035
N	3.105718	2.304066	1.361139
N	2.354290	2.318835	0.532713
Si	-0.077376	3.417149	-0.067056
C	-1.241698	3.241190	-1.514680
H	-1.546672	2.206160	-1.725480
H	-2.149440	3.838019	-1.325424
H	-0.766428	3.648948	-2.421835
C	-0.788508	3.000139	1.611056
H	-0.023251	3.141016	2.392510
H	-1.626455	3.677468	1.843124
H	-1.148200	1.964755	1.674244
C	0.644806	5.146027	-0.027785
H	1.136322	5.396618	-0.981541
H	-0.153517	5.886734	0.146831
H	1.385786	5.255422	0.780833
Al	4.037803	-0.888396	0.052950
Cl	4.396051	-2.677932	-1.076065
Cl	5.489693	0.635690	-0.370046
Cl	3.866681	-1.295959	2.155105

65

TS1b-Al'_2 Eopt -3290.915455

C	-1.038298	-0.270286	0.403577
C	-0.495859	-0.056426	-0.960063
C	0.691294	-0.556204	-1.321382
C	1.396139	-1.506297	-0.395099
C	1.077176	-1.255886	1.051394
C	-0.104565	-0.777467	1.462839
C	1.460447	-0.269333	-2.567419
C	2.918963	-1.419746	-0.486586
C	3.401652	0.008435	-0.858290
C	2.775446	0.460133	-2.196839
C	4.912064	-0.131766	-0.944052
C	4.557679	-0.843088	1.276773
C	3.443788	-1.825826	0.903456
H	1.694261	-1.227466	-3.058850
H	-1.105265	0.524464	-1.651685
H	3.509157	0.308835	-2.999857
H	5.174940	-1.182995	2.115746
O	-2.291850	-0.515276	0.566395
C	-0.518307	-0.705422	2.903003

H	-1.592244	-0.918666	3.004051
H	0.033873	-1.441262	3.501771
H	-0.318964	0.283679	3.345311
C	2.313904	-1.466737	1.896152
H	2.186868	-2.188004	2.715488
H	0.879554	0.334521	-3.278196
H	2.577496	1.539196	-2.156874
O	5.616996	0.106935	-1.886996
O	5.452916	-0.578905	0.203382
C	3.163454	1.004637	0.332867
H	2.076780	1.150283	0.440047
C	3.690106	0.366271	1.649367
H	4.218189	1.110505	2.259655
H	3.742052	-2.878389	0.951387
H	3.256868	-2.111944	-1.269182
C	3.820544	2.365030	0.105579
H	3.534065	2.814115	-0.855527
H	3.524904	3.062354	0.903667
H	4.920031	2.293069	0.124777
O	2.694954	-0.209332	2.466744
O	1.098999	-2.862597	-0.741612
C	-0.240235	-3.289910	-0.695458
H	-0.875568	-2.772886	-1.434815
H	-0.230047	-4.361095	-0.938032
H	-0.691238	-3.170143	0.304714
C	-0.918893	1.816584	1.006487
H	0.034924	1.857707	1.550658
N	-2.893870	1.616564	2.421615
N	-1.945462	1.774940	1.851338
Si	-1.188294	3.192451	-0.327889
C	0.313003	3.235433	-1.435522
H	0.360117	2.379752	-2.122501
H	0.276839	4.155491	-2.042324
H	1.239490	3.266068	-0.841534
C	-2.795256	2.823786	-1.208469
H	-3.066840	3.685142	-1.841177
H	-2.750730	1.935733	-1.856243
H	-3.615244	2.672749	-0.487651
C	-1.335081	4.791555	0.637138
H	-2.184276	4.760275	1.339311
H	-0.418693	4.999032	1.212807
H	-1.501021	5.636131	-0.052266
Al	-3.789709	-1.047704	-0.260862
Cl	-4.098272	-3.066435	0.403166
Cl	-3.539591	-0.969400	-2.397440
Cl	-5.340004	0.280403	0.404173

65

TS1b-Al'_3 Eopt -3290.908135

C	1.254806	-0.131118	0.346682
C	0.413969	-0.026512	1.570958
C	-0.781217	-0.621815	1.625941
C	-1.167681	-1.508321	0.468566
C	-0.652402	-0.979766	-0.847573
C	0.544277	-0.385357	-0.955496
C	-1.804106	-0.509805	2.711409
C	-2.682749	-1.639350	0.280780
C	-3.452135	-0.350881	0.696687
C	-3.147140	0.028026	2.161258
C	-4.909460	-0.723644	0.485566
C	-4.072471	-1.078453	-1.691936
C	-2.878554	-1.915428	-1.220998
H	-1.970463	-1.510564	3.140000
H	0.817803	0.528744	2.422026
H	-3.963589	-0.340160	2.796666
H	-4.473504	-1.397558	-2.660221
O	2.461983	-0.532052	0.523292

C	1.193203	-0.079819	-2.272257
H	1.538783	0.958592	-2.354468
H	2.062716	-0.735745	-2.433529
H	0.491387	-0.258067	-3.095860
C	-1.677746	-1.243338	-1.931333
H	-1.278980	-1.823834	-2.774184
H	-1.445092	0.133939	3.526225
H	-3.157440	1.121057	2.259081
O	-5.792384	-0.718458	1.300347
O	-5.165497	-1.097033	-0.781062
C	-3.186011	0.811860	-0.322341
H	-2.131842	1.103198	-0.220052
C	-3.385046	0.289543	-1.770381
H	-3.928904	1.019425	-2.383748
H	-2.965525	-2.983506	-1.446071
H	-3.064153	-2.464880	0.897536
C	-4.052739	2.044157	-0.075667
H	-3.958484	2.427411	0.949375
H	-3.752028	2.850528	-0.761044
H	-5.119107	1.835989	-0.260901
O	-2.181725	-0.007971	-2.444755
O	-0.564577	-2.797910	0.553105
C	-0.683130	-3.509055	1.758823
H	-0.233155	-4.495805	1.584958
H	-0.135282	-3.020807	2.584028
H	-1.732384	-3.662085	2.068809
C	1.662595	2.011316	0.098656
H	2.321791	1.916456	-0.776879
N	3.039017	2.121507	2.111960
N	2.437644	2.132037	1.169081
Si	0.268232	3.325264	-0.072978
C	-0.860106	3.199037	1.412123
H	-1.351982	2.220598	1.485408
H	-0.294383	3.367642	2.343313
H	-1.638721	3.977093	1.351868
C	1.113020	4.997641	-0.080097
H	1.835712	5.078058	-0.907884
H	0.366512	5.800342	-0.201912
H	1.650319	5.180125	0.864838
C	-0.551093	3.003579	-1.719372
H	-1.393709	3.703621	-1.847143
H	0.162713	3.198054	-2.536341
H	-0.933867	1.979444	-1.838955
Al	4.051719	-0.998949	-0.157956
Cl	3.794804	-2.723566	-1.405959
Cl	5.300463	-1.400577	1.534221
Cl	4.752951	0.717257	-1.260879

65

TS1b-Al'_4 Eopt -3290.910722

C	1.277777	0.052679	0.387880
C	0.412409	0.261217	1.577293
C	-0.739557	-0.404185	1.689298
C	-1.058839	-1.436312	0.635051
C	-0.555879	-1.020802	-0.728331
C	0.605134	-0.371120	-0.887644
C	-1.741911	-0.330862	2.791247
C	-2.568037	-1.671252	0.439362
C	-3.405638	-0.406497	0.797842
C	-3.143866	0.034221	2.251413
C	-4.845145	-0.841997	0.584853
C	-3.973530	-1.240168	-1.566968
C	-2.759258	-2.017702	-1.052092
H	-1.779971	-1.329191	3.257824
H	0.787819	0.913672	2.369884
H	-3.906692	-0.419548	2.897868
H	-4.348134	-1.610406	-2.527650

O	2.494296	-0.273325	0.632267
C	1.200688	-0.063775	-2.230068
H	2.288015	0.082487	-2.178450
H	1.004089	-0.881212	-2.936690
H	0.762565	0.853247	-2.652312
C	-1.577001	-1.347614	-1.793131
H	-1.168552	-1.944538	-2.621400
H	-1.431757	0.380969	3.568174
H	-3.286333	1.119796	2.329764
O	-5.735825	-0.840164	1.391034
O	-5.076265	-1.264839	-0.670562
C	-3.167116	0.727530	-0.260709
H	-2.124277	1.057370	-0.157485
C	-3.330845	0.144744	-1.690328
H	-3.891431	0.832609	-2.336382
H	-2.823187	-3.095933	-1.233194
H	-2.892549	-2.489247	1.098418
C	-4.075882	1.939232	-0.070920
H	-3.805824	2.719823	-0.797517
H	-5.135348	1.686055	-0.238947
H	-3.989117	2.377433	0.932426
O	-2.111590	-0.139252	-2.343288
O	-0.383385	-2.595286	1.109303
C	-0.427703	-3.751190	0.311982
H	0.256871	-4.476969	0.771186
H	-1.434626	-4.203450	0.276248
H	-0.081179	-3.563537	-0.719860
C	1.581946	2.179480	-0.133019
H	2.107301	2.041558	-1.088779
N	3.219799	2.449740	1.654954
N	2.492052	2.394521	0.807950
Si	0.131310	3.442825	-0.218517
C	0.921963	5.139989	-0.304390
H	1.594537	5.226006	-1.172832
H	0.145229	5.917441	-0.397239
H	1.505688	5.356858	0.605377
C	-0.774182	3.057925	-1.805904
H	-1.107690	2.012170	-1.881202
H	-1.658859	3.710707	-1.890346
H	-0.124462	3.272256	-2.670218
C	-0.926927	3.332483	1.319689
H	-0.321016	3.496578	2.225781
H	-1.689072	4.128752	1.283658
H	-1.440464	2.367387	1.420545
Al	3.980588	-1.036786	-0.024158
Cl	3.397150	-2.759930	-1.162528
Cl	5.165337	-1.551570	1.681551
Cl	4.928692	0.484233	-1.220678

65

TS1b-Al'_5 Eopt -3290.901970

C	1.246016	0.173448	-0.335853
C	0.663415	0.119465	1.033924
C	-0.469784	-0.568659	1.209596
C	-1.021881	-1.297165	0.004699
C	-0.850404	-0.480542	-1.259539
C	0.265289	0.227638	-1.476101
C	-1.180768	-0.861447	2.487931
C	-2.529569	-1.597647	0.082375
C	-3.285844	-0.582080	0.988163
C	-2.698232	-0.571953	2.410595
C	-4.725694	-1.062154	0.969061
C	-4.375375	-0.720809	-1.335971
C	-3.061001	-1.504389	-1.363958
H	-1.027917	-1.937623	2.677002
H	1.229192	0.531904	1.872832
H	-3.235233	-1.316754	3.012739

H	-4.957779	-0.820934	-2.258561
O	2.382096	-0.367907	-0.572067
C	0.595260	0.942696	-2.747552
H	-0.039920	0.594592	-3.572252
H	0.445259	2.029445	-2.649454
H	1.646871	0.769556	-3.022827
C	-2.100733	-0.543357	-2.105179
H	-1.905207	-0.827121	-3.149324
H	-0.723177	-0.322238	3.328222
H	-2.905409	0.399445	2.877260
O	-5.402017	-1.380934	1.909519
O	-5.237511	-1.108815	-0.273975
C	-3.320022	0.828930	0.308766
H	-2.287584	1.195177	0.271503
C	-3.802853	0.689023	-1.158137
H	-4.510995	1.485508	-1.420776
H	-3.145643	-2.482706	-1.849232
H	-2.670172	-2.600589	0.510593
C	-4.165597	1.854898	1.057941
H	-3.833035	1.998554	2.095132
H	-4.094255	2.828956	0.551244
H	-5.230792	1.572044	1.079740
O	-2.759797	0.726663	-2.109178
O	-0.236084	-2.483779	-0.027926
C	-0.447834	-3.380800	-1.087851
H	0.320528	-4.158987	-0.996902
H	-1.439512	-3.864300	-1.040269
H	-0.330530	-2.897599	-2.074605
C	1.981971	2.289967	-0.354702
H	2.402236	2.196662	-1.364276
N	3.692789	1.822921	1.320135
N	2.944561	2.083923	0.529115
Si	0.879757	3.829548	-0.017300
C	1.238517	5.022160	-1.413266
H	0.646422	5.943852	-1.288706
H	2.304140	5.300785	-1.437692
H	0.975220	4.583105	-2.389264
C	-0.917187	3.334272	0.011472
H	-1.544685	4.240322	0.047798
H	-1.211105	2.750050	-0.873694
H	-1.127639	2.743191	0.915174
C	1.401070	4.501819	1.650562
H	0.783441	5.377702	1.908888
H	1.262578	3.751985	2.447082
H	2.455734	4.821397	1.656683
Al	3.585752	-1.542587	0.080950
Cl	3.311457	-1.826213	2.188871
Cl	3.312935	-3.356350	-1.033540
Cl	5.478555	-0.626557	-0.360730

65

TS1b-Al'_6 Eopt -3290.907474

C	-1.135857	-0.160250	0.501037
C	-0.595977	-0.047371	-0.881788
C	0.600542	-0.559578	-1.191958
C	1.283013	-1.430822	-0.166120
C	1.013694	-0.980131	1.245874
C	-0.168439	-0.472471	1.609256
C	1.370209	-0.363069	-2.460147
C	2.809850	-1.443977	-0.292030
C	3.377145	-0.092694	-0.815430
C	2.752311	0.276635	-2.177197
C	4.870308	-0.354776	-0.915361
C	4.524848	-0.799088	1.375866
C	3.331249	-1.726749	1.129762
H	1.524007	-1.344180	-2.935387
H	-1.207571	0.483953	-1.613633

H	3.448034	-0.017240	-2.974384
H	5.137956	-1.096454	2.233806
O	-2.347785	-0.562163	0.691904
C	-0.599052	-0.250924	3.028605
H	-0.483064	0.796510	3.348844
H	-1.658405	-0.524869	3.144955
H	-0.007884	-0.867974	3.717504
C	2.252305	-1.170091	2.090097
H	2.085731	-1.788240	2.983585
H	0.807346	0.255415	-3.173179
H	2.659539	1.368205	-2.242700
O	5.563965	-0.277425	-1.893474
O	5.408411	-0.716437	0.263124
C	3.246817	1.037434	0.267384
H	2.178552	1.274813	0.384338
C	3.761003	0.506347	1.632746
H	4.359678	1.267564	2.150000
H	3.548224	-2.787140	1.296498
H	3.117442	-2.226508	-0.999739
C	3.989692	2.315478	-0.118695
H	3.675745	2.705468	-1.096730
H	3.797152	3.099083	0.629426
H	5.080040	2.157978	-0.152500
O	2.742574	0.103530	2.520087
O	0.775435	-2.764047	-0.179097
C	0.711243	-3.435972	-1.410781
H	0.370856	-4.457330	-1.192541
H	-0.015846	-2.972848	-2.099227
H	1.692958	-3.502011	-1.914659
C	-1.256294	1.943113	0.885843
H	-0.469299	2.083350	1.640186
N	-3.480771	1.685744	1.845337
N	-2.447646	1.902175	1.480932

Si	-1.139448	3.329714	-0.467898
C	-2.444855	3.095008	-1.779496
H	-3.442290	2.961921	-1.333249
H	-2.473615	4.004131	-2.403485
H	-2.251708	2.240224	-2.443944
C	-1.454590	4.921518	0.469308
H	-0.728127	5.059372	1.286107
H	-1.365665	5.783567	-0.212728
H	-2.468200	4.940652	0.902233
C	0.603170	3.285918	-1.126916
H	0.811811	2.358175	-1.675580
H	0.747233	4.131745	-1.819393
H	1.335624	3.390573	-0.312324
Al	-3.763075	-1.235436	-0.183173
Cl	-4.805521	0.386609	-1.135092
Cl	-3.042480	-2.656476	-1.624023
Cl	-4.953757	-2.158207	1.341840

65

TS1b-Al'_7 Eopt -3290.906434

C	-1.157438	-0.348951	0.474059
C	-0.630570	-0.241048	-0.910470
C	0.567680	-0.724314	-1.250352
C	1.315431	-1.585867	-0.265280
C	1.000804	-1.230093	1.167014
C	-0.207954	-0.800572	1.551241
C	1.278615	-0.550036	-2.550958
C	2.847140	-1.455077	-0.376517
C	3.261962	-0.066680	-0.942322
C	2.646187	0.146115	-2.338825
C	4.779494	-0.122126	-1.003942
C	4.449131	-0.538168	1.288191
C	3.404730	-1.632143	1.051486
H	1.444277	-1.554297	-2.973347

H	-1.265115	0.277466	-1.630879
H	3.356630	-0.227130	-3.088189
H	5.074474	-0.722359	2.168845
O	-2.410245	-0.646942	0.657218
C	-0.633547	-0.665039	2.983969
H	-0.531150	0.362141	3.369101
H	-1.688614	-0.959211	3.088327
H	-0.030533	-1.313385	3.632592
C	2.257495	-1.227388	2.004654
H	2.200120	-1.830105	2.922119
H	0.668541	0.012783	-3.270028
H	2.545624	1.221944	-2.532240
O	5.475630	0.024630	-1.972110
O	5.338780	-0.367011	0.193675
C	2.943110	1.066059	0.099844
H	1.848802	1.143380	0.193383
C	3.503728	0.650882	1.490163
H	3.978433	1.505034	1.990767
H	3.781928	-2.643524	1.237986
H	3.225998	-2.224012	-1.064910
C	3.488807	2.431102	-0.313714
H	4.588820	2.429844	-0.380597
H	3.093001	2.763437	-1.283426
H	3.210733	3.187160	0.435386
O	2.541986	0.128669	2.381523
O	0.866595	-2.898638	-0.584221
C	1.367023	-3.964061	0.180353
H	1.195320	-3.823746	1.263025
H	0.821018	-4.863267	-0.135927
H	2.444154	-4.141843	0.011281
C	-1.025168	1.649445	0.962680
H	-0.138172	1.645239	1.612834
N	-3.138071	1.603104	2.182562
N	-2.133083	1.710597	1.711785
Si	-0.948424	3.195687	-0.238550
C	0.072267	4.425876	0.736105
H	0.158173	5.373043	0.178134
H	-0.389009	4.647573	1.711847
H	1.091188	4.046753	0.914519
C	-0.103414	2.799888	-1.852355
H	0.827174	2.237886	-1.697308
H	-0.747278	2.241054	-2.546244
H	0.156453	3.759070	-2.331484
C	-2.700439	3.793876	-0.477571
H	-3.344942	3.003236	-0.892778
H	-3.146871	4.151548	0.464205
H	-2.699730	4.637670	-1.187220
Al	-3.845482	-1.035830	-0.344274
Cl	-5.487175	-1.076497	1.033006
Cl	-3.483139	-2.935419	-1.272327
Cl	-4.112898	0.505513	-1.835876

65

TS1b-Al'_8 Eopt -3290.906893

C	-1.125569	-0.360652	0.566472
C	-0.634905	-0.320372	-0.838382
C	0.575726	-0.780958	-1.167644
C	1.350985	-1.557102	-0.136285
C	1.076030	-1.104298	1.272247
C	-0.122531	-0.650121	1.652606
C	1.315833	-0.540664	-2.445809
C	2.864787	-1.414172	-0.300586
C	3.278347	-0.004452	-0.822938
C	2.550377	0.339686	-2.143668
C	4.782156	-0.139946	-0.985602
C	4.562684	-0.629641	1.317505
C	3.446073	-1.655615	1.102700

H	1.647236	-1.499131	-2.872735
H	-1.288188	0.150790	-1.572307
H	3.261933	0.265590	-2.977317
H	5.233970	-0.880392	2.146190
O	-2.338456	-0.762678	0.808669
C	-0.520335	-0.411354	3.079492
H	-1.565760	-0.716777	3.234682
H	0.111615	-0.992753	3.763192
H	-0.428511	0.645933	3.376355
C	2.344987	-1.199053	2.091793
H	2.249451	-1.838061	2.980987
H	0.679183	-0.050534	-3.195076
H	2.220741	1.387805	-2.106365
O	5.429344	-0.000330	-1.988044
O	5.391633	-0.462462	0.170668
C	3.106964	1.103651	0.276879
H	2.028752	1.272652	0.426525
C	3.698303	0.602275	1.619576
H	4.244413	1.404644	2.132789
H	3.758304	-2.693796	1.256793
H	3.231559	-2.154326	-1.025028
C	3.759635	2.425510	-0.127156
H	3.403442	2.788802	-1.101363
H	3.535983	3.199067	0.622328
H	4.856535	2.336548	-0.183598
O	2.741227	0.104606	2.526761
O	0.982626	-2.935076	-0.110787
C	0.955483	-3.637098	-1.327172
H	0.732705	-4.683942	-1.079712
H	0.164134	-3.267744	-2.002361
H	1.922703	-3.611020	-1.861488
C	-1.183015	1.690794	0.853809
H	-0.322233	1.819635	1.524516
N	-3.298220	1.575045	2.064958
N	-2.305012	1.723059	1.581397
Si	-1.152969	3.135110	-0.476797
C	0.352020	4.123200	0.029230
H	1.266472	3.516039	-0.053170
H	0.469988	4.997967	-0.631159
H	0.270639	4.486268	1.066118
C	-0.983022	2.597223	-2.255344
H	-0.049188	2.048798	-2.445766
H	-1.837369	1.999571	-2.605619
H	-0.955058	3.522337	-2.856792
C	-2.732125	4.104481	-0.223519
H	-2.740351	4.962925	-0.915313
H	-3.624211	3.494736	-0.436579
H	-2.814635	4.499938	0.801695
Al	-3.787139	-1.117488	-0.193460
Cl	-4.249000	0.711527	-1.252402
Cl	-5.333031	-1.648429	1.186439
Cl	-3.302178	-2.704447	-1.553574

65

TS1b-Al-1 Eopt -3290.919162

C	1.075125	-0.129602	0.033885
C	0.426203	-0.065520	1.358356
C	-0.818701	-0.526391	1.537502
C	-1.454875	-1.324517	0.424742
C	-0.982623	-0.910737	-0.947955
C	0.243418	-0.412303	-1.160170
C	-1.724211	-0.219678	2.684386
C	-2.975712	-1.137041	0.369698
C	-3.411243	0.305008	0.778844
C	-2.875024	0.678814	2.179414
C	-4.927576	0.228118	0.729897
C	-4.408242	-0.362535	-1.495570

C	-3.364848	-1.410586	-1.092891
H	-2.139696	-1.155347	3.089542
H	0.960519	0.491383	2.132472
H	-3.701111	0.649508	2.902616
H	-4.963383	-0.627432	-2.402108
O	2.107156	0.623582	-0.147916
C	0.793405	-0.020148	-2.495002
H	1.828802	-0.374591	-2.605493
H	0.186454	-0.429289	-3.312064
H	0.811569	1.076232	-2.597331
C	-2.126224	-1.011465	-1.933929
H	-1.911500	-1.695142	-2.767150
H	-1.183888	0.284451	3.496879
H	-2.509075	1.713813	2.157958
O	-5.702092	0.437779	1.624014
O	-5.382870	-0.128225	-0.484889
C	-3.031843	1.358261	-0.323986
H	-1.936592	1.462331	-0.335102
C	-3.471693	0.832992	-1.715305
H	-3.918222	1.634324	-2.317809
H	-3.682960	-2.445126	-1.259219
H	-3.463163	-1.839273	1.060091
C	-3.637652	2.735618	-0.058846
H	-4.736777	2.723251	-0.140745
H	-3.377349	3.122787	0.935908
H	-3.259142	3.452857	-0.802025
O	-2.427459	0.271025	-2.478577
O	-1.110602	-2.707655	0.496566
C	-1.303062	-3.380621	1.717091
H	-1.062428	-4.435955	1.531140
H	-0.633855	-3.004738	2.511070
H	-2.345654	-3.323950	2.075050
C	2.040793	-2.174049	0.258666
H	1.609959	-2.450977	1.229630
N	0.975006	-3.390867	-1.562889
N	1.485200	-2.891191	-0.699148
Si	3.945957	-1.967192	0.178881
C	4.671204	-3.692421	0.322118
H	4.352502	-4.332324	-0.517118
H	5.773119	-3.644992	0.308258
H	4.365123	-4.180572	1.261442
C	4.413531	-1.216943	-1.467486
H	4.074447	-0.175098	-1.554355
H	5.510571	-1.231978	-1.578714
H	3.989754	-1.799941	-2.301857
C	4.413073	-0.939387	1.665400
H	5.508794	-0.824319	1.707187
H	3.967363	0.064851	1.638797
H	4.093059	-1.435920	2.595963
Al	2.432352	2.374608	0.056077
Cl	3.987019	2.864426	-1.332589
Cl	3.012990	2.749371	2.093836
Cl	0.556164	3.337680	-0.398232

65

TS1b-Al-2 Eopt -3290.919779

C	-1.078962	0.150334	0.018827
C	-0.431137	0.031391	1.339483
C	0.805487	0.499279	1.543178
C	1.460823	1.325733	0.462186
C	0.996949	0.948562	-0.927577
C	-0.236504	0.473336	-1.161436
C	1.670135	0.281893	2.735177
C	2.989162	1.130473	0.417978
C	3.407856	-0.293339	0.901702
C	2.898421	-0.562890	2.333440
C	4.926268	-0.265381	0.834304

C	4.406761	0.218352	-1.410209
C	3.405781	1.321326	-1.053969
H	1.999134	1.269985	3.097431
H	-0.980279	-0.522524	2.104623
H	3.714895	-0.377342	3.043814
H	4.957882	0.419853	-2.335365
O	-2.107468	-0.599248	-0.201714
C	-0.769500	0.101053	-2.509151
H	-0.760877	-0.992521	-2.637301
H	-1.812200	0.434784	-2.618541
H	-0.166249	0.544481	-3.311218
C	2.161500	0.942494	-1.893384
H	2.002494	1.583005	-2.773019
H	1.120506	-0.207325	3.550081
H	2.636109	-1.624545	2.429755
O	5.700469	-0.451694	1.733768
O	5.383776	0.005221	-0.400320
C	2.981606	-1.390781	-0.141039
H	1.883509	-1.459462	-0.141671
C	3.426953	-0.950651	-1.561478
H	3.838785	-1.797866	-2.124608
H	3.777446	2.329851	-1.263827
H	3.469190	1.862547	1.083144
C	3.543713	-2.773136	0.185991
H	4.642785	-2.799038	0.106736
H	3.269367	-3.107997	1.195599
H	3.143537	-3.509942	-0.525898
O	2.400970	-0.388650	-2.350006
O	1.077475	2.658716	0.798662
C	1.640103	3.713070	0.054116
H	1.126247	4.630574	0.371376
H	2.718310	3.836939	0.251705
H	1.489305	3.591247	-1.033344

C	-2.024791	2.158016	0.354806
H	-1.494670	2.427786	1.277056
N	-1.154881	3.405530	-1.549117
N	-1.567948	2.888725	-0.643595
Si	-3.930652	1.948624	0.443530
C	-4.529915	1.285077	-1.197109
H	-5.632389	1.291025	-1.217984
H	-4.181309	1.919249	-2.029142
H	-4.189573	0.254642	-1.371239
C	-4.633697	3.662641	0.740702
H	-5.732876	3.615243	0.819038
H	-4.246168	4.099630	1.675103
H	-4.385404	4.346462	-0.087589
C	-4.260589	0.842939	1.909918
H	-3.823497	1.273807	2.825460
H	-5.348011	0.753442	2.068424
H	-3.853673	-0.168931	1.774894
Al	-2.444925	-2.355072	-0.081667
Cl	-4.088343	-2.738551	-1.400883
Cl	-2.910653	-2.857448	1.958551
Cl	-0.618045	-3.317835	-0.704970

65

TS1b-Al-3 Eopt -3290.915016

C	-1.028269	-0.149201	-0.067832
C	-0.334205	-0.521264	1.179153
C	0.872781	-0.022846	1.478730
C	1.495363	1.021923	0.580090
C	1.015370	0.918433	-0.844146
C	-0.212666	0.477104	-1.154140
C	1.757986	-0.442070	2.602079
C	3.027094	0.866401	0.482098
C	3.482089	-0.613099	0.675434
C	2.998041	-1.165807	2.032767

C	4.998071	-0.536258	0.597886
C	4.432810	0.360347	-1.504958
C	3.417293	1.350634	-0.927846
H	2.070806	0.464218	3.145183
H	-0.835763	-1.234184	1.838386
H	3.820300	-1.102281	2.757815
H	4.965115	0.749162	-2.379996
O	-1.990110	-0.893190	-0.499531
C	-0.706384	0.313654	-2.559038
H	-0.070160	0.855442	-3.269417
H	-0.711104	-0.749981	-2.842207
H	-1.737294	0.676461	-2.665838
C	2.166232	1.119070	-1.807922
H	1.986389	1.914004	-2.546413
H	1.230912	-1.092850	3.312293
H	2.758577	-2.231745	1.924401
O	5.790416	-0.870232	1.436780
O	5.430074	-0.023673	-0.567698
C	3.062761	-1.497217	-0.555668
H	1.966381	-1.590752	-0.552269
C	3.472383	-0.777303	-1.868797
H	3.890278	-1.488405	-2.593032
H	3.767505	2.388076	-0.940279
H	3.498568	1.468225	1.272411
C	3.665760	-2.900363	-0.511220
H	4.763485	-2.877311	-0.607371
H	3.419565	-3.433664	0.417098
H	3.275236	-3.496900	-1.348880
O	2.421314	-0.095004	-2.517024
O	1.116065	2.259284	1.172217
C	1.560705	3.449652	0.570606
H	2.648323	3.599683	0.683222
H	1.301103	3.506164	-0.501163
H	1.050775	4.271233	1.092147
C	-2.287508	1.423882	0.813849
H	-2.997873	0.664313	1.176177
N	-1.090436	2.320985	2.744152
N	-1.681246	1.941085	1.875114
Si	-3.076386	2.661723	-0.419330
C	-1.745804	3.600848	-1.339547
H	-2.218956	4.368921	-1.974036
H	-1.087366	4.121151	-0.625507
H	-1.124432	2.964330	-1.984494
C	-4.193739	1.608298	-1.485220
H	-4.623268	2.211463	-2.301742
H	-3.678068	0.744082	-1.927430
H	-5.027883	1.219396	-0.878741
C	-4.082769	3.870160	0.602159
H	-4.602399	4.587016	-0.055434
H	-4.844224	3.347752	1.203321
H	-3.441035	4.448924	1.286917
Al	-3.002303	-2.282206	0.004105
Cl	-3.866173	-1.783752	1.918994
Cl	-4.497141	-2.484628	-1.515790
Cl	-1.698734	-3.981574	0.140220

65

TS1b-Al-4 Eopt -3290.913796

C	-1.109353	-0.275232	-0.172876
C	-0.467641	-0.751350	1.070886
C	0.752030	-0.325698	1.432810
C	1.411862	0.767137	0.620166
C	0.997090	0.739214	-0.827635
C	-0.210928	0.318057	-1.214213
C	1.627689	-0.950560	2.470438
C	2.935553	0.608747	0.568088
C	3.388447	-0.885491	0.559729

C	2.806656	-1.657288	1.766029
C	4.903220	-0.768411	0.608778
C	4.455209	0.429202	-1.376310
C	3.367578	1.298208	-0.735473
H	2.017408	-0.172841	3.144879
H	-0.993054	-1.517428	1.646457
H	3.605934	-1.842461	2.496315
H	5.038086	0.953881	-2.141165
O	-2.102410	-0.957740	-0.647796
C	-0.684387	0.260813	-2.631822
H	-0.005812	0.800499	-3.303539
H	-0.746760	-0.784354	-2.972192
H	-1.695488	0.682567	-2.727574
C	2.167762	1.133070	-1.701464
H	1.960752	2.023892	-2.312037
H	1.070934	-1.672999	3.082427
H	2.454938	-2.640508	1.425691
O	5.646876	-1.208087	1.443497
O	5.395609	-0.071635	-0.431633
C	3.094857	-1.585190	-0.815458
H	2.006057	-1.712687	-0.907223
C	3.562996	-0.667358	-1.974415
H	4.055124	-1.248510	-2.764969
H	3.661510	2.342728	-0.584970
H	3.396554	1.074213	1.448557
C	3.755415	-2.959273	-0.925057
H	4.854597	-2.883675	-0.952508
H	3.484548	-3.621618	-0.091198
H	3.437218	-3.450013	-1.856711
O	2.528118	0.070841	-2.582898
O	1.023566	2.070109	1.066307
C	1.499641	2.550648	2.302382
H	1.255251	1.883103	3.144767

H	2.586918	2.734544	2.294936
H	0.996578	3.512562	2.471495
C	-2.167716	1.463738	0.524095
H	-3.138626	0.947759	0.532380
N	-1.389347	1.659707	2.830245
N	-1.792641	1.608057	1.790016
Si	-2.253303	3.114830	-0.486890
C	-0.671385	3.574514	-1.367450
H	-0.760401	4.625606	-1.690780
H	0.190818	3.485423	-0.689993
H	-0.486156	2.962041	-2.260881
C	-3.678486	2.828851	-1.663859
H	-3.796791	3.695881	-2.334169
H	-3.523759	1.936161	-2.289978
H	-4.622042	2.694416	-1.111064
C	-2.659523	4.437212	0.778909
H	-2.811064	5.406321	0.275277
H	-3.578439	4.197869	1.337927
H	-1.838642	4.565522	1.504023
Al	-3.371275	-2.083322	-0.073516
Cl	-2.485585	-4.021553	0.172041
Cl	-4.070456	-1.294531	1.812745
Cl	-4.904137	-2.043089	-1.569569

65

TS1b-Al-5 Eopt -3290.916840

C	-0.952460	-0.260715	-0.450329
C	-0.426547	0.026589	0.899219
C	0.777459	0.583038	1.067172
C	1.539428	1.066055	-0.143137
C	1.240248	0.242251	-1.375262
C	0.038624	-0.316043	-1.577332
C	1.494761	0.799008	2.354946
C	3.066900	0.950976	0.033894

C	3.463000	-0.206475	1.002687
C	2.802425	-0.021476	2.384068
C	4.978132	-0.109344	1.078643
C	4.701822	-0.425882	-1.238126
C	3.643368	0.672752	-1.368423
H	1.730027	1.874209	2.426841
H	-1.033918	-0.295062	1.746435
H	3.520269	0.462289	3.059593
H	5.348232	-0.508260	-2.118830
O	-1.941809	-1.076309	-0.571512
C	-0.329146	-1.147714	-2.766847
H	-1.244086	-0.767385	-3.246480
H	0.476109	-1.158286	-3.510983
H	-0.540025	-2.184563	-2.465112
C	2.510109	-0.028807	-2.153641
H	2.449836	0.272494	-3.209426
H	0.862925	0.540389	3.215136
H	2.586650	-1.007366	2.815836
O	5.650657	0.064537	2.058792
O	5.564814	-0.238393	-0.124201
C	3.184811	-1.609915	0.350295
H	2.095162	-1.739603	0.268934
C	3.770845	-1.634156	-1.086738
H	4.262037	-2.593594	-1.294529
H	4.014452	1.576621	-1.863067
H	3.449172	1.889730	0.460335
C	3.749402	-2.769067	1.170204
H	4.850789	-2.746469	1.206222
H	3.378172	-2.772435	2.204090
H	3.455948	-3.724169	0.710088
O	2.823062	-1.422454	-2.109442
O	1.112355	2.418385	-0.286193
C	1.640520	3.164889	-1.355207
H	1.095105	4.118045	-1.373842
H	2.713212	3.387787	-1.223704
H	1.494989	2.663603	-2.328812
C	-1.876998	1.673047	-0.901457
H	-1.071694	2.086876	-1.519172
N	-3.653263	0.596437	-2.172064
N	-2.841945	1.171819	-1.658793
Si	-2.510719	2.868109	0.491584
C	-4.292782	3.248712	0.051784
H	-4.921579	2.344461	0.079643
H	-4.706675	3.964143	0.781554
H	-4.379097	3.701358	-0.949487
C	-2.414479	2.154109	2.215961
H	-1.380266	2.067004	2.578326
H	-2.949682	2.848151	2.886817
H	-2.908091	1.173724	2.288011
C	-1.433500	4.390304	0.358536
H	-1.773294	5.150170	1.081741
H	-0.387828	4.134204	0.586303
H	-1.479129	4.834696	-0.648507
Al	-3.220704	-1.978260	0.302408
Cl	-3.931097	-3.426783	-1.105074
Cl	-4.750221	-0.563154	0.850796
Cl	-2.345173	-2.893925	2.037428

65

TS1b-B'_1 Eopt -1992.376669

C	-1.754837	-0.654584	0.195215
C	-1.108799	-0.838691	-1.135938
C	0.164643	-1.244909	-1.209404
C	0.873356	-1.660572	0.051065
C	0.409438	-0.878553	1.251085
C	-0.851474	-0.440403	1.376105
C	1.023141	-1.337848	-2.428897

C	2.384972	-1.455198	0.000350
C	2.804504	-0.243746	-0.871667
C	2.240308	-0.387554	-2.303203
C	4.321883	-0.287843	-0.828206
C	3.845510	-0.117863	1.479806
C	2.814420	-1.250180	1.463549
H	1.378194	-2.377574	-2.516386
H	-1.698925	-0.635288	-2.030871
H	3.041018	-0.746123	-2.964139
H	4.419750	-0.062361	2.411151
O	-2.890960	-1.200125	0.479229
C	-1.421306	0.158197	2.625137
H	-1.627221	1.232653	2.511124
H	-2.368117	-0.339209	2.884442
H	-0.728547	0.045708	3.468460
C	1.587501	-0.625505	2.171730
H	1.431846	-0.985232	3.198478
H	0.457137	-1.104908	-3.341105
H	1.953571	0.603253	-2.679810
O	5.080960	-0.375225	-1.755038
O	4.799861	-0.216965	0.428119
C	2.429598	1.107690	-0.170614
H	1.334469	1.186191	-0.173185
C	2.891766	1.070597	1.310987
H	3.329655	2.031149	1.611876
H	3.158724	-2.176252	1.935459
H	2.834077	-2.358867	-0.432464
C	3.008191	2.328586	-0.881059
H	2.748148	2.353439	-1.948170
H	2.618935	3.248047	-0.419078
H	4.107011	2.361178	-0.801967
O	1.863975	0.776317	2.233829
O	0.746637	-3.067481	0.274221

C	-0.520710	-3.606012	0.558230
H	-0.377169	-4.691658	0.646014
H	-0.933188	-3.234300	1.512160
H	-1.255369	-3.417252	-0.240508
C	-2.453951	1.380786	-0.241144
H	-2.864665	1.090579	-1.218405
B	-3.895793	-1.725187	-0.530859
F	-3.340960	-2.801142	-1.210207
F	-4.209045	-0.694262	-1.421675
F	-4.997399	-2.099316	0.209993
N	-4.302336	1.323275	1.352326
N	-3.479459	1.416375	0.602525
Si	-1.345157	2.941239	-0.386097
C	-0.301778	3.205910	1.140613
H	0.393490	4.042142	0.954856
H	-0.939579	3.499930	1.990502
H	0.287175	2.328241	1.443751
C	-0.382779	2.633649	-1.959531
H	0.157787	1.677056	-1.939070
H	-1.073444	2.611756	-2.818213
H	0.344140	3.442882	-2.132902
C	-2.503343	4.399464	-0.605732
H	-1.924275	5.327185	-0.749092
H	-3.155070	4.266680	-1.484071
H	-3.144494	4.539768	0.280110

65

TS1b-B'_2 Eopt -1992.365178

C	1.625721	-0.709731	-0.230595
C	0.935070	-0.925071	1.080991
C	-0.373462	-1.200078	1.081311
C	-1.046627	-1.386764	-0.256839
C	-0.511159	-0.428740	-1.292549
C	0.790848	-0.126222	-1.339017

C	-1.264850	-1.393641	2.267630
C	-2.558473	-1.144736	-0.228422
C	-2.986136	-0.075903	0.820286
C	-2.485364	-0.445045	2.232370
C	-4.499409	-0.061048	0.710702
C	-3.914728	0.482051	-1.513911
C	-2.911871	-0.666975	-1.649907
H	-1.623093	-2.435288	2.254575
H	1.518816	-0.897745	2.001819
H	-3.310520	-0.904346	2.793251
H	-4.445195	0.711676	-2.444702
O	2.559687	-1.506873	-0.633245
C	1.456527	0.615458	-2.454094
H	1.787605	1.616269	-2.140298
H	2.343167	0.063236	-2.801760
H	0.777032	0.746576	-3.305623
C	-1.639531	0.037312	-2.185620
H	-1.447377	-0.144930	-3.252277
H	-0.708447	-1.259352	3.205393
H	-2.226126	0.476252	2.769850
O	-5.301594	-0.279352	1.578247
O	-4.918230	0.237845	-0.533270
C	-2.541837	1.357232	0.372385
H	-1.447339	1.382103	0.420659
C	-2.942077	1.594957	-1.105206
H	-3.347251	2.604744	-1.250177
H	-3.252071	-1.483392	-2.295689
H	-3.075675	-2.081033	0.024425
C	-3.091056	2.472271	1.259000
H	-2.836740	2.334727	2.319016
H	-2.669199	3.437111	0.939585
H	-4.187901	2.551635	1.183706
O	-1.876297	1.436993	-2.016211
O	-0.767045	-2.666949	-0.821847
C	-0.944417	-3.799686	-0.010185
H	-1.972102	-3.885539	0.387856
H	-0.752576	-4.673604	-0.647435
H	-0.229687	-3.823609	0.829480
C	2.843640	0.978978	0.428008
H	3.064277	0.538386	1.409640
B	3.353117	-2.433173	0.273333
F	3.909684	-1.666999	1.302408
F	4.333801	-2.980532	-0.528317
F	2.506104	-3.402432	0.788065
N	4.687377	0.436465	-1.077477
N	3.894125	0.733718	-0.350746
Si	2.355921	2.844705	0.529820
C	2.933573	3.403918	2.220329
H	2.439311	2.825752	3.017978
H	4.022908	3.282246	2.332339
H	2.691391	4.468454	2.375010
C	3.278160	3.754915	-0.823037
H	4.368706	3.628802	-0.724164
H	2.985421	3.437502	-1.835909
H	3.060524	4.832667	-0.739686
C	0.508126	3.034207	0.386241
H	0.099989	2.609939	-0.543176
H	0.016981	2.552894	1.245607
H	0.253693	4.106964	0.416092

65

TS1b-B'_3 Eopt -1992.364231

C	1.621601	-0.758040	-0.134210
C	0.917999	-0.853019	1.183651
C	-0.390149	-1.127385	1.201940
C	-1.067019	-1.430020	-0.113843
C	-0.514931	-0.573590	-1.231441

C	0.790764	-0.284845	-1.297723
C	-1.270850	-1.319294	2.392445
C	-2.582980	-1.165433	-0.118695
C	-2.996744	-0.068413	0.905290
C	-2.547413	-0.448729	2.328738
C	-4.507053	0.007135	0.776838
C	-3.879333	0.491098	-1.444078
C	-2.930934	-0.705227	-1.550331
H	-1.559497	-2.383915	2.392398
H	1.502124	-0.777540	2.101533
H	-3.369755	-0.981838	2.824312
H	-4.386741	0.726731	-2.386192
O	2.558340	-1.592127	-0.452377
C	1.457212	0.382161	-2.458535
H	0.786635	0.436736	-3.325237
H	1.767896	1.408839	-2.216722
H	2.356593	-0.179776	-2.753976
C	-1.635253	-0.075693	-2.116662
H	-1.473226	-0.277405	-3.185357
H	-0.724135	-1.134656	3.326988
H	-2.386011	0.467898	2.910641
O	-5.324967	-0.166472	1.639668
O	-4.902650	0.309001	-0.473366
C	-2.481112	1.335191	0.439071
H	-1.386795	1.310766	0.497823
C	-2.858771	1.562850	-1.047945
H	-3.213685	2.588188	-1.213678
H	-3.321065	-1.516600	-2.174228
H	-3.107611	-2.092111	0.154993
C	-2.984308	2.491829	1.298907
H	-4.078525	2.606761	1.231920
H	-2.722113	2.373823	2.359239
H	-2.533291	3.432570	0.949274

O	-1.800206	1.337032	-1.955180
O	-0.767503	-2.807597	-0.313356
C	-1.217221	-3.415542	-1.495561
H	-0.877025	-2.880722	-2.400901
H	-0.783586	-4.424599	-1.514604
H	-2.316649	-3.516477	-1.532312
C	2.824536	0.976091	0.361378
H	3.044968	0.620576	1.377204
B	3.383079	-2.400849	0.535857
F	4.383430	-2.984212	-0.217082
F	2.579519	-3.345476	1.147049
F	3.918027	-1.520058	1.483899
N	4.681399	0.319566	-1.083034
N	3.882275	0.671428	-0.388509
Si	2.354079	2.850146	0.314760
C	2.971755	3.539497	1.942291
H	2.743362	4.615860	2.014065
H	2.488273	3.034064	2.794109
H	4.061893	3.415156	2.043071
C	3.259823	3.642075	-1.121465
H	4.351055	3.514581	-1.031675
H	2.947882	3.253381	-2.103277
H	3.051954	4.725058	-1.114986
C	0.506717	3.046216	0.192278
H	0.032396	2.659622	1.106980
H	0.264519	4.119885	0.121251
H	0.071038	2.535947	-0.679694

65

TS1b-B'_4 Eopt -1992.365247

C	-1.657851	-0.711103	0.283604
C	-0.937847	-0.850077	-1.021205
C	0.388785	-1.030637	-1.031088
C	1.027241	-1.288263	0.325807

C	0.456709	-0.324119	1.348623
C	-0.859628	-0.095515	1.402513
C	1.223906	-1.018084	-2.289397
C	2.552661	-1.109219	0.411039
C	3.106518	-0.230141	-0.734845
C	2.741619	-0.899514	-2.058416
C	4.603340	-0.193422	-0.511690
C	3.850959	0.671855	1.542981
C	2.845176	-0.459118	1.779069
H	1.019582	-1.930733	-2.872168
H	-1.511057	-0.789135	-1.947228
H	3.197735	-1.901008	-2.045128
H	4.300715	1.051302	2.467266
O	-2.572727	-1.541118	0.659352
C	-1.561582	0.632633	2.504222
H	-1.928379	1.616920	2.179121
H	-2.428164	0.049350	2.851515
H	-0.896321	0.796541	3.361262
C	1.561118	0.294705	2.168153
H	1.357490	0.309849	3.247680
H	0.870219	-0.181293	-2.910833
H	3.204722	-0.370136	-2.902307
O	5.462044	-0.543721	-1.275835
O	4.931621	0.296519	0.697457
C	2.614316	1.238539	-0.561324
H	1.523125	1.238561	-0.672932
C	2.906881	1.693385	0.894566
H	3.293124	2.720956	0.918317
H	3.162011	-1.186302	2.534750
H	3.036077	-2.092014	0.332913
C	3.192999	2.199163	-1.593591
H	4.288349	2.288120	-1.507107
H	2.960064	1.879908	-2.619782
H	2.766639	3.203664	-1.450869
O	1.776025	1.649851	1.741950
O	0.664779	-2.579369	0.809284
C	0.873816	-3.654867	-0.070472
H	0.208225	-3.604431	-0.948176
H	1.920820	-3.722447	-0.419511
H	0.638855	-4.571385	0.487288
C	-2.905302	0.937268	-0.382441
H	-3.148585	0.450427	-1.336433
B	-3.314082	-2.481543	-0.273097
F	-4.277238	-3.089473	0.506198
F	-2.420221	-3.401659	-0.799733
F	-3.896179	-1.720877	-1.293128
N	-4.726426	0.456491	1.170726
N	-3.940286	0.727701	0.425850
Si	-2.372690	2.778095	-0.603007
C	-3.089105	3.287015	-2.256830
H	-2.821446	4.332014	-2.485274
H	-2.698628	2.652902	-3.069390
H	-4.188249	3.208878	-2.259130
C	-3.122622	3.797296	0.777911
H	-2.672783	3.593070	1.761533
H	-2.955720	4.864588	0.556153
H	-4.211302	3.642788	0.855657
C	-0.512941	2.877323	-0.638998
H	-0.050557	2.511844	0.290088
H	-0.119419	2.295176	-1.486766
H	-0.208283	3.927225	-0.785775

65

TS1b-B_1 Eopt -1992.375951

C	-1.226471	0.951870	0.147954
C	-0.771283	0.210991	-1.050849
C	0.284551	-0.610022	-0.992301

C	0.916937	-0.908619	0.346486
C	0.791513	0.242426	1.310088
C	-0.247861	1.084243	1.281174
C	1.012380	-1.182193	-2.166659
C	2.430491	-1.142848	0.236549
C	3.094059	-0.293267	-0.891454
C	2.417259	-0.546733	-2.257103
C	4.539991	-0.759166	-0.857503
C	4.304220	0.026868	1.355310
C	3.002509	-0.741473	1.606154
H	1.111184	-2.271800	-2.042470
H	-1.263391	0.438255	-1.995954
H	3.068001	-1.187298	-2.867645
H	4.938187	0.111720	2.244709
O	-1.987867	1.990737	0.021907
C	-0.413622	2.250291	2.207818
H	-0.265215	3.195546	1.663671
H	-1.426060	2.287799	2.634089
H	0.311284	2.209426	3.029690
C	2.048267	0.350256	2.148237
H	1.853357	0.273944	3.227313
H	0.461363	-1.005683	-3.100462
H	2.324039	0.407627	-2.792099
O	5.170527	-1.259931	-1.749233
O	5.113439	-0.563042	0.343573
C	3.162921	1.229195	-0.509772
H	2.138431	1.629757	-0.524152
C	3.706534	1.376410	0.935353
H	4.416756	2.210410	1.007245
H	3.111107	-1.594135	2.284817
H	2.631898	-2.198230	0.008243
C	4.015387	2.041342	-1.484030
H	5.078597	1.755132	-1.433788
H	3.684818	1.927282	-2.525699
H	3.951436	3.109825	-1.230300
O	2.710841	1.591819	1.909387
O	0.286990	-2.000257	1.021887
C	0.189988	-3.228340	0.342935
H	-0.194572	-3.958995	1.066956
H	-0.504845	-3.178391	-0.512454
H	1.165803	-3.594006	-0.019736
C	-2.539814	-0.546434	0.998667
H	-1.791648	-1.038606	1.634410
B	-2.897172	2.316319	-1.139125
F	-3.669176	1.188037	-1.431188
F	-2.123263	2.671781	-2.236804
F	-3.688180	3.364258	-0.713138
N	-3.880204	1.166489	2.093342
N	-3.276092	0.319237	1.681369
Si	-3.638299	-1.755251	-0.051702
C	-5.388351	-1.093731	-0.005145
H	-6.043738	-1.772238	-0.576142
H	-5.780487	-1.042462	1.023734
H	-5.454643	-0.094494	-0.459476
C	-3.554655	-3.383317	0.875966
H	-4.182233	-4.135302	0.368865
H	-2.529344	-3.779386	0.925873
H	-3.929065	-3.270356	1.906265
C	-3.038476	-1.969785	-1.806826
H	-2.004500	-2.338739	-1.864982
H	-3.691822	-2.715122	-2.291888
H	-3.113320	-1.028720	-2.368450

65

TS1b-B_2 Eopt -1992.373987

C	-1.280865	0.609560	0.426473
C	-0.751478	0.640493	-0.955656

C	0.332412	-0.068579	-1.292996
C	0.934129	-1.035227	-0.299809
C	0.721333	-0.588721	1.128740
C	-0.349692	0.127540	1.499717
C	1.065129	-0.018166	-2.589677
C	2.465202	-1.153053	-0.438518
C	3.107059	0.152138	-1.002839
C	2.494580	0.523033	-2.369241
C	4.582952	-0.193313	-1.114666
C	4.248897	-0.557078	1.187662
C	3.007744	-1.428823	0.977716
H	1.114983	-1.046407	-2.984540
H	-1.248568	1.294175	-1.671179
H	3.151270	0.149586	-3.166201
H	4.853871	-0.867833	2.046564
O	-2.119023	1.505666	0.822949
C	-0.568655	0.667596	2.880414
H	-0.264734	1.725363	2.923454
H	-1.629946	0.630577	3.160771
H	0.019416	0.113125	3.622672
C	1.976547	-0.805148	1.946829
H	1.805971	-1.397350	2.857777
H	0.533678	0.596272	-3.328384
H	2.474042	1.615864	-2.470259
O	5.263800	-0.177092	-2.104237
O	5.120584	-0.550563	0.064743
C	3.047888	1.314572	0.054474
H	1.995722	1.611687	0.178677
C	3.554649	0.789106	1.424095
H	4.195588	1.531293	1.917254
H	3.187091	-2.492840	1.165404
H	2.697319	-1.969591	-1.137343
C	3.849084	2.544388	-0.370156
H	4.929017	2.330898	-0.425082
H	3.531801	2.934591	-1.346792
H	3.710284	3.348145	0.367873
O	2.533160	0.451829	2.336185
O	0.277278	-2.267328	-0.603970
C	0.582506	-3.389565	0.191575
H	-0.111034	-4.186269	-0.109403
H	1.611621	-3.750372	0.025822
H	0.436975	-3.189755	1.267930
C	-2.483965	-1.215161	0.225183
H	-1.674079	-1.823293	-0.209794
B	-2.592434	2.733175	0.072673
F	-3.196970	2.370144	-1.131324
F	-1.483142	3.534110	-0.166097
F	-3.503675	3.345081	0.912392
N	-2.857701	-1.771319	2.573407
N	-2.683517	-1.563431	1.485812
Si	-4.100514	-1.063531	-0.801996
C	-5.262333	0.076006	0.114938
H	-6.230340	0.124148	-0.410543
H	-5.454920	-0.298666	1.133833
H	-4.863038	1.096459	0.188497
C	-4.817649	-2.797534	-0.865286
H	-5.759175	-2.800031	-1.439926
H	-4.122433	-3.500489	-1.352245
H	-5.040433	-3.178362	0.144998
C	-3.625679	-0.497067	-2.514872
H	-2.805206	-1.109643	-2.921933
H	-4.494462	-0.608773	-3.184863
H	-3.323484	0.558189	-2.517699

65

TS1b-B_3 Eopt -1992.374367

C	-1.313679	0.850797	0.141378

C	-0.552182	1.162799	-1.085024
C	0.508910	0.427553	-1.443923
C	0.879859	-0.802747	-0.647776
C	0.445173	-0.706434	0.790854
C	-0.642687	-0.016400	1.162124
C	1.464674	0.726513	-2.548722
C	2.406151	-1.013382	-0.574416
C	3.192152	0.330974	-0.657929
C	2.840790	1.095061	-1.952230
C	4.648724	-0.101267	-0.625723
C	3.901801	-1.019167	1.410673
C	2.678817	-1.692610	0.781674
H	1.558078	-0.177279	-3.172305
H	-0.869032	2.025559	-1.671704
H	3.622933	0.908828	-2.700248
H	4.334151	-1.594396	2.236747
O	-2.087704	1.741667	0.676382
C	-1.073160	0.145405	2.588126
H	-0.575824	-0.584300	3.238669
H	-0.825126	1.154833	2.949908
H	-2.159725	0.028195	2.695408
C	1.521197	-1.249626	1.708083
H	1.163801	-2.040898	2.383333
H	1.099382	1.539143	-3.190612
H	2.853543	2.174039	-1.749528
O	5.493231	0.112625	-1.452942
O	4.955453	-0.799090	0.482012
C	2.999581	1.179863	0.651671
H	1.955147	1.524123	0.685168
C	3.237227	0.275612	1.890865
H	3.814932	0.805361	2.659361
H	2.776332	-2.780365	0.702726
H	2.722177	-1.638492	-1.422059

C	3.914721	2.402138	0.705467
H	4.976885	2.115208	0.771406
H	3.795534	3.054127	-0.170572
H	3.680189	3.000604	1.598161
O	2.059465	-0.195091	2.508337
O	0.223667	-1.868624	-1.326230
C	0.402221	-3.175665	-0.841525
H	1.425008	-3.553968	-1.012561
H	0.162659	-3.266258	0.232513
H	-0.292238	-3.814617	-1.404283
C	-2.863989	-0.361009	-0.791697
H	-3.408057	0.558745	-1.053118
B	-2.655716	2.979703	0.033457
F	-3.605632	3.454483	0.918620
F	-3.244532	2.638882	-1.192873
F	-1.632117	3.896937	-0.164813
N	-1.936902	-1.295232	-2.848700
N	-2.409235	-0.888460	-1.921751
Si	-3.872242	-1.514339	0.359612
C	-2.766576	-2.760581	1.210152
H	-3.395622	-3.452721	1.794926
H	-2.219241	-3.359686	0.465407
H	-2.038019	-2.305851	1.895203
C	-4.759750	-0.338881	1.512079
H	-5.245272	-0.891584	2.332715
H	-4.086647	0.413871	1.947669
H	-5.542958	0.203576	0.957992
C	-5.091787	-2.429681	-0.732212
H	-5.745314	-3.072828	-0.119292
H	-5.732591	-1.728720	-1.290811
H	-4.573652	-3.076387	-1.459600

65

TS1b-B_4 Eopt -1992.374123

C	1.392531	0.994642	-0.268458
C	0.653438	1.379127	0.954976
C	-0.399179	0.665324	1.381431
C	-0.753745	-0.619545	0.665595
C	-0.380574	-0.582343	-0.793010
C	0.664567	0.118166	-1.243151
C	-1.397743	1.111332	2.401611
C	-2.265194	-0.875075	0.650374
C	-3.100326	0.440572	0.555978
C	-2.733891	1.422184	1.692132
C	-4.528501	-0.069647	0.657609
C	-3.800522	-1.245719	-1.254948
C	-2.512860	-1.746019	-0.591133
H	-1.553402	0.312821	3.143113
H	0.945161	2.306039	1.449640
H	-3.544668	1.433834	2.433163
H	-4.230558	-1.960198	-1.965315
O	2.167218	1.867974	-0.841993
C	1.084712	0.192515	-2.677118
H	0.551751	-0.546068	-3.288181
H	0.877426	1.193284	-3.085979
H	2.167248	0.028874	-2.781401
C	-1.412265	-1.337321	-1.602578
H	-0.981184	-2.183251	-2.157254
H	-1.046303	2.000936	2.941627
H	-2.668128	2.437877	1.279704
O	-5.352525	0.215974	1.483844
O	-4.829803	-0.945303	-0.318225
C	-3.016835	1.091454	-0.871009
H	-2.000456	1.490510	-1.003715
C	-3.240723	0.001686	-1.951819
H	-3.880637	0.373997	-2.762257
H	-2.514960	-2.817634	-0.363550
H	-2.575885	-1.380465	1.573990
C	-4.015625	2.234023	-1.052361
H	-5.056961	1.873368	-1.035311
H	-3.914032	3.005862	-0.276925
H	-3.852120	2.717992	-2.026595
O	-2.055643	-0.479192	-2.543348
O	-0.027454	-1.740809	1.179532
C	-0.352171	-2.256853	2.449517
H	-0.317348	-1.494508	3.244705
H	-1.338984	-2.749023	2.466550
H	0.406820	-3.018875	2.673987
C	2.859243	-0.348001	0.499707
H	3.651385	0.410402	0.436777
B	2.875010	3.009169	-0.165236
F	3.818886	3.462245	-1.069448
F	3.489568	2.534392	1.001241
F	1.956312	4.006347	0.148639
N	2.189659	-0.570493	2.837383
N	2.554241	-0.490115	1.784977
Si	3.366968	-1.995476	-0.384752
C	1.958169	-2.919572	-1.191009
H	2.321057	-3.929288	-1.447984
H	1.107968	-3.013997	-0.499579
H	1.611316	-2.440035	-2.117042
C	4.662137	-1.454501	-1.620818
H	5.012399	-2.322144	-2.203798
H	4.268631	-0.708569	-2.328874
H	5.532917	-1.012232	-1.111032
C	4.115680	-3.061140	0.964738
H	4.515288	-3.991834	0.528876
H	4.942691	-2.545174	1.478694
H	3.364510	-3.345160	1.720267

65

TS1b-B_5 Eopt -1992.377879

C	-1.263761	0.981644	0.205929
C	-0.784221	0.377202	-1.054811
C	0.274089	-0.440082	-1.062955
C	0.886062	-0.872737	0.246379
C	0.755463	0.191415	1.311766
C	-0.292470	1.025704	1.350516
C	0.950783	-1.014499	-2.260132
C	2.404149	-1.122533	0.139537
C	3.067734	-0.254956	-0.974785
C	2.410652	-0.519191	-2.344765
C	4.524478	-0.688006	-0.950135
C	4.280104	0.056850	1.269737
C	2.999742	-0.747343	1.510958
H	0.938809	-2.112181	-2.151029
H	-1.286292	0.669669	-1.975725
H	3.014103	-1.256418	-2.890807
H	4.911077	0.140601	2.161437
O	-2.065214	1.996992	0.178309
C	-0.478580	2.111007	2.365362
H	-1.497528	2.094073	2.779509
H	0.239095	2.013668	3.188925
H	-0.342355	3.099789	1.901444
C	2.036509	0.305195	2.109863
H	1.886412	0.203165	3.194561
H	0.409380	-0.768680	-3.183442
H	2.434749	0.402361	-2.940657
O	5.161659	-1.158851	-1.853339
O	5.101025	-0.495230	0.249013
C	3.096148	1.263419	-0.566246
H	2.060915	1.635920	-0.563455
C	3.647098	1.396923	0.878906
H	4.337190	2.246842	0.959736
H	3.148569	-1.615543	2.161669
H	2.575500	-2.179274	-0.111882
C	3.915816	2.123713	-1.526740
H	4.984319	1.853780	-1.508833
H	3.564812	2.044316	-2.564602
H	3.839666	3.180779	-1.231965
O	2.657330	1.569553	1.868421
O	0.168008	-2.060417	0.577283
C	0.494907	-2.701721	1.786001
H	-0.241623	-3.503491	1.930123
H	1.498312	-3.159956	1.760428
H	0.435956	-2.018617	2.652403
C	-2.543995	-0.639808	0.918305
H	-1.845501	-1.086474	1.634364
B	-3.063340	2.335777	-0.907913
F	-3.830286	3.365567	-0.401994
F	-3.841312	1.199481	-1.153631
F	-2.387240	2.722043	-2.058094
N	-4.093683	0.940896	1.933656
N	-3.406387	0.149475	1.542841
Si	-3.388869	-1.909844	-0.278641
C	-5.234215	-1.634516	-0.098142
H	-5.518384	-0.623321	-0.428558
H	-5.777156	-2.359607	-0.726621
H	-5.574994	-1.771337	0.941195
C	-2.894439	-1.769982	-2.074496
H	-1.835958	-2.014022	-2.241789
H	-3.502305	-2.501526	-2.634575
H	-3.105269	-0.770148	-2.477638
C	-2.885367	-3.591566	0.375831
H	-3.429211	-4.382573	-0.167093
H	-1.807231	-3.750223	0.219198
H	-3.106727	-3.702229	1.449412

65

TS1b-B_6 Eopt -1992.374266

C	-1.332612	0.466112	0.138436
C	-0.684423	0.685149	-1.171808
C	0.446004	0.046923	-1.492299
C	0.970750	-1.032595	-0.576793
C	0.600547	-0.801113	0.870556
C	-0.530760	-0.170317	1.219803
C	1.311049	0.292497	-2.679683
C	2.511061	-1.097551	-0.569398
C	3.153562	0.295036	-0.860022
C	2.673095	0.852538	-2.216381
C	4.646850	0.009861	-0.860891
C	4.092930	-0.710637	1.310176
C	2.916072	-1.570983	0.840761
H	1.459903	-0.670052	-3.196161
H	-1.139301	1.426015	-1.829891
H	3.433786	0.638662	-2.978877
H	4.618340	-1.133824	2.173366
O	-2.208127	1.329083	0.550872
C	-0.967742	0.085811	2.627263
H	-2.038975	-0.137749	2.744114
H	-0.396109	-0.521614	3.339814
H	-0.832916	1.147389	2.884949
C	1.767387	-1.125012	1.777709
H	1.522042	-1.855361	2.562170
H	0.836606	0.983044	-3.389363
H	2.595735	1.945597	-2.151140
O	5.424980	0.196388	-1.757017
O	5.074738	-0.510641	0.302223
C	2.933672	1.280081	0.346562
H	1.862201	1.525618	0.399373
C	3.324230	0.566083	1.669225
H	3.882447	1.241667	2.330126
H	3.117149	-2.646737	0.886589
H	2.850186	-1.790186	-1.353058
C	3.717038	2.583519	0.198676
H	4.805656	2.412938	0.227907
H	3.482110	3.109596	-0.736586
H	3.467280	3.259738	1.029509
O	2.234038	0.066546	2.411952
O	0.364084	-2.217932	-1.092585
C	0.754356	-3.451137	-0.537667
H	0.084042	-4.212321	-0.959588
H	1.790378	-3.722262	-0.802675
H	0.652384	-3.473121	0.561980
C	-2.596669	-1.246167	-0.439211
H	-2.151652	-1.455958	-1.420618
B	-2.236156	2.802537	0.224262
F	-3.172535	3.350444	1.081661
F	-2.598446	3.018918	-1.108498
F	-0.957368	3.304248	0.458842
N	-1.928804	-2.931261	1.192209
N	-2.257520	-2.206795	0.403402
Si	-4.456832	-0.751856	-0.414284
C	-5.411593	-2.318111	-0.809470
H	-5.241843	-3.097161	-0.048031
H	-6.493708	-2.106281	-0.835863
H	-5.122676	-2.727664	-1.790844
C	-4.903613	-0.138376	1.292895
H	-4.411317	0.818126	1.515938
H	-5.995052	0.005914	1.356144
H	-4.622046	-0.872114	2.066104
C	-4.654350	0.514134	-1.767336
H	-5.711611	0.817603	-1.841781
H	-4.051739	1.411827	-1.571521

H	-4.354210	0.093883	-2.740916

65

TS1b-B_7 Eopt -1992.373530

C	-1.321134	0.507832	0.099232
C	-0.659339	0.682239	-1.215210
C	0.490250	0.055457	-1.494462
C	0.993548	-0.996708	-0.537609
C	0.611347	-0.716145	0.894575
C	-0.516056	-0.067145	1.213117
C	1.419346	0.368092	-2.622294
C	2.524221	-1.081271	-0.510174
C	3.203559	0.306115	-0.734692
C	2.727786	0.958571	-2.052123
C	4.682834	-0.040029	-0.750004
C	4.090744	-0.834491	1.392236
C	2.875563	-1.619816	0.887155
H	1.648498	-0.553606	-3.178752
H	-1.097319	1.416130	-1.892345
H	3.522705	0.871097	-2.805055
H	4.599752	-1.314885	2.234960
O	-2.208069	1.377886	0.467624
C	-0.980826	0.208973	2.607478
H	-2.033156	-0.089877	2.728345
H	-0.371114	-0.326398	3.345538
H	-0.929477	1.286399	2.826203
C	1.734930	-1.136847	1.817180
H	1.420922	-1.882161	2.561277
H	0.965092	1.076616	-3.328036
H	2.570070	2.032063	-1.883359
O	5.472304	0.151625	-1.635088
O	5.081302	-0.633679	0.389720
C	3.032421	1.246968	0.512617
H	1.974614	1.543972	0.574204
C	3.381282	0.462506	1.804295
H	3.967765	1.080941	2.495997
H	3.009650	-2.706610	0.899499
H	2.874710	-1.753744	-1.305493
C	3.884047	2.512097	0.413580
H	4.961538	2.283926	0.454146
H	3.693191	3.073472	-0.511354
H	3.656944	3.178018	1.259133
O	2.260046	-0.009566	2.516363
O	0.419084	-2.277402	-0.804150
C	0.453601	-2.765643	-2.122942
H	0.035566	-3.780897	-2.089317
H	-0.161817	-2.157583	-2.809464
H	1.478298	-2.830833	-2.528770
C	-2.560847	-1.248820	-0.361755
H	-2.231066	-1.439848	-1.391940
B	-2.388250	2.789876	-0.030914
F	-3.382641	3.332717	0.762647
F	-2.767032	2.807266	-1.377921
F	-1.173952	3.450007	0.131378
N	-1.689273	-2.934347	1.171392
N	-2.113570	-2.213656	0.428642
Si	-4.436418	-0.852368	-0.155297
C	-5.323609	-2.500014	-0.302018
H	-5.023067	-3.191480	0.502672
H	-6.413695	-2.351281	-0.223773
H	-5.116981	-2.986803	-1.268758
C	-4.756212	-0.113954	1.530683
H	-4.304005	0.882225	1.628141
H	-5.844750	-0.022807	1.682893
H	-4.364470	-0.763620	2.330439
C	-4.859553	0.271846	-1.580073
H	-5.916912	0.576828	-1.515906

H -4.238256 1.178379 -1.574943
H -4.714354 -0.247546 -2.541140
53
TS2'-A_1 Eopt -1583.885118
C 1.934536 -0.210095 0.257562
C 1.072357 -1.040714 1.237113
C -0.177374 -1.433031 0.949600
C -0.708724 -1.273665 -0.447635
C -0.102777 -0.104600 -1.168105
C 1.129939 0.349098 -0.923273
C -1.170773 -2.030917 1.887109
C -2.215252 -1.024476 -0.481036
C -2.713786 -0.254839 0.773298
C -2.327554 -1.014790 2.064234
C -4.216810 -0.182330 0.564727
C -3.472698 0.894372 -1.400341
C -2.469826 -0.206104 -1.758087
H -1.556727 -2.963710 1.447515
H 1.534837 -1.321699 2.188513
H -3.212203 -1.541693 2.446626
H -3.933575 1.366642 -2.274821
O 3.065513 -0.883060 -0.116286
C 1.753026 1.475789 -1.694961
H 1.536388 2.451752 -1.230638
H 2.842892 1.348150 -1.750515
H 1.355096 1.518394 -2.717791
C -1.159641 0.575998 -2.015542
H -0.893462 0.664200 -3.078488
H -0.727873 -2.272680 2.862489
H -2.035108 -0.291373 2.837607
O -5.078291 -0.600536 1.290236
O -4.545164 0.430258 -0.586671
C -2.238861 1.242435 0.744666
H -1.149085 1.257408 0.898034
C -2.523700 1.845478 -0.659371
H -2.912296 2.868712 -0.572828
H -2.773326 -0.830637 -2.604996
H -2.725432 -1.996398 -0.510657
C -2.895183 2.089912 1.833141
H -3.979439 2.197449 1.667978
H -2.749350 1.667102 2.837091
H -2.463295 3.101887 1.830446
O -1.405025 1.895404 -1.514826
O -0.538569 -2.500467 -1.156649
C 0.764132 -2.887117 -1.521229
H 0.673857 -3.887712 -1.965594
H 1.209732 -2.208891 -2.268119
H 1.450455 -2.946336 -0.660026
C 2.040485 0.830889 1.329742
H 1.161286 1.462507 1.491861
H 2.670629 0.608160 2.194047
B 4.441814 -0.457189 0.101765
F 5.269313 -1.513592 -0.257962
F 4.653771 -0.112817 1.460488
F 4.751377 0.683283 -0.675422
N 3.794037 3.146149 0.709459
N 3.148813 2.274179 0.851026
53
TS2'-A_2 Eopt -1583.888311
C -1.975902 0.253488 -0.104654
C -1.309199 -0.891473 -0.892777
C -0.096883 -1.373485 -0.573579
C 0.536537 -0.998540 0.736422
C 0.137394 0.372024 1.200304
C -1.039392 0.928402 0.901088
C 0.752233 -2.279163 -1.397462

C	2.062020	-0.965578	0.666879
C	2.580519	-0.549969	-0.738232
C	2.016894	-1.493863	-1.826052
C	4.089739	-0.651532	-0.599052
C	3.618072	0.902948	1.114222
C	2.500054	0.056450	1.729397
H	1.041272	-3.145472	-0.782153
H	-1.849251	-1.299292	-1.750766
H	2.798534	-2.206699	-2.121419
H	4.190544	1.474377	1.853203
O	-3.204333	0.019369	0.454609
C	-1.470778	2.265043	1.429577
H	-1.277092	3.074790	0.706952
H	-2.545967	2.255652	1.661434
H	-0.926308	2.521924	2.347557
C	1.324204	1.055236	1.851719
H	1.135225	1.396445	2.879454
H	0.220557	-2.653432	-2.282350
H	1.770552	-0.909167	-2.722994
O	4.841732	-1.327304	-1.248013
O	4.566831	0.131068	0.385196
C	2.312086	0.973130	-1.014447
H	1.226434	1.111758	-1.127497
C	2.762500	1.807108	0.217095
H	3.279303	2.723145	-0.098098
H	2.767147	-0.414357	2.681441
H	2.439991	-1.972699	0.887480
C	3.002073	1.473550	-2.282270
H	4.099489	1.457928	-2.182876
H	2.737258	0.875679	-3.165647
H	2.707451	2.514557	-2.482310
O	1.714444	2.194093	1.076502
O	0.243280	-2.007790	1.700730
C	-1.069859	-2.089065	2.204531
H	-1.106973	-3.002490	2.813737
H	-1.323672	-1.229091	2.847088
H	-1.832172	-2.167657	1.412471
C	-2.005614	1.050221	-1.374082
H	-1.116365	1.616355	-1.674293
H	-2.684407	0.713108	-2.161504
B	-4.207664	-0.850834	-0.155448
F	-5.411769	-0.618849	0.504862
F	-3.847883	-2.205052	-0.041427
F	-4.352459	-0.549148	-1.536586
N	-3.961085	3.275765	-0.893733
N	-3.161779	2.542973	-1.041588

53

TS2'-A_3 Eopt -1583.880849

C	2.060595	0.355084	0.144354
C	1.369254	-0.745544	0.982043
C	0.157557	-1.226039	0.663934
C	-0.437509	-0.865312	-0.671692
C	-0.082393	0.534130	-1.090618
C	1.092521	1.088673	-0.789313
C	-0.697551	-2.107879	1.512432
C	-1.969722	-0.892412	-0.659177
C	-2.566804	-0.479022	0.722227
C	-2.006246	-1.360394	1.860497
C	-4.059669	-0.663252	0.511631
C	-3.586530	0.905974	-1.189292
C	-2.396722	0.114507	-1.740314
H	-0.942576	-3.023064	0.952779
H	1.896045	-1.139639	1.855274
H	-2.768361	-2.094049	2.156022
H	-4.151168	1.439830	-1.961558
O	3.220128	0.035471	-0.512653

C	1.522529	2.433128	-1.297394
H	1.429136	3.216637	-0.526996
H	2.572004	2.397613	-1.626767
H	0.909470	2.749333	-2.151198
C	-1.263213	1.168921	-1.795105
H	-1.027239	1.510869	-2.812726
H	-0.178092	-2.409735	2.431753
H	-1.818115	-0.732744	2.741858
O	-4.804450	-1.379663	1.124506
O	-4.528767	0.089805	-0.499557
C	-2.398443	1.058550	0.997746
H	-1.330522	1.258848	1.170314
C	-2.826300	1.860882	-0.258885
H	-3.405014	2.751241	0.020160
H	-2.586907	-0.362009	-2.707825
H	-2.326438	-1.907741	-0.882477
C	-3.186078	1.525235	2.221693
H	-4.273559	1.446629	2.061354
H	-2.937700	0.950977	3.125080
H	-2.962488	2.582819	2.426657
O	-1.751906	2.293824	-1.059772
O	0.075116	-1.683361	-1.716872
C	0.110268	-3.075348	-1.529605
H	0.463785	-3.507763	-2.475434
H	0.816776	-3.369394	-0.734496
H	-0.883401	-3.506154	-1.308469
C	2.264012	1.119983	1.409437
H	1.456198	1.742383	1.810397
H	3.007989	0.731678	2.110066
B	4.126846	-1.003828	-0.028729
F	5.314627	-0.884609	-0.745692
F	3.577834	-2.283819	-0.206695
F	4.387377	-0.822883	1.358270

N	4.308831	3.268323	0.760415
N	3.492470	2.569760	0.968745

53

TS2'-B_1 Eopt -1583.880558

C	1.976093	0.466692	-0.053318
C	1.100526	1.354570	0.815500
C	-0.070934	0.988060	1.331067
C	-0.541345	-0.424650	1.145056
C	-0.020047	-1.037648	-0.125796
C	1.167145	-0.753440	-0.684337
C	-1.075192	1.864316	2.010149
C	-2.061190	-0.533527	1.031364
C	-2.703587	0.706902	0.349338
C	-2.316523	2.003271	1.099263
C	-4.192064	0.408592	0.417846
C	-3.445392	-1.480707	-0.786022
C	-2.318510	-1.801733	0.200661
H	-1.362592	1.397849	2.966008
H	1.493561	2.364171	0.976146
H	-3.174106	2.338820	1.698167
H	-3.898400	-2.370714	-1.236343
O	3.143331	0.196092	0.599004
C	1.680183	-1.537097	-1.872453
H	1.253562	-1.158680	-2.815070
H	2.771855	-1.507809	-1.946322
H	1.381806	-2.590007	-1.780617
C	-1.079633	-1.951966	-0.713329
H	-0.743083	-2.989293	-0.850080
H	-0.663336	2.857367	2.236933
H	-2.117685	2.797294	0.366025
O	-5.049590	1.069755	0.937825
O	-4.511652	-0.746519	-0.194114
C	-2.382165	0.742935	-1.188024

H	-1.310665	0.963267	-1.304694
C	-2.642630	-0.659105	-1.802663
H	-3.132464	-0.574384	-2.781412
H	-2.505125	-2.683485	0.822148
H	-2.473218	-0.619121	2.045615
C	-3.181360	1.810893	-1.933288
H	-4.259225	1.581906	-1.942211
H	-3.054838	2.809700	-1.492559
H	-2.849508	1.862700	-2.980979
O	-1.482146	-1.441333	-1.986020
O	-0.222724	-1.259517	2.254058
C	1.131812	-1.422897	2.602762
H	1.149649	-2.148577	3.427219
H	1.741668	-1.816370	1.773469
H	1.590906	-0.482445	2.949753
C	2.097069	1.079071	-1.384247
H	2.887927	0.743273	-2.060795
H	1.235412	1.604781	-1.810634
B	4.226189	-0.667151	0.137698
F	5.316850	-0.437327	0.970879
F	4.581668	-0.351328	-1.203176
F	3.853887	-2.020456	0.192404
N	3.855829	3.616718	-0.750001
N	3.199031	2.776782	-0.997487

53

TS2'-B_2 Eopt -1583.873840

C	2.049965	0.426121	-0.205027
C	1.169319	1.529275	0.359005
C	-0.016157	1.316476	0.925808
C	-0.472912	-0.106094	1.105258
C	0.012940	-1.001733	-0.003583
C	1.194179	-0.864036	-0.618642
C	-1.024472	2.353023	1.317545
C	-1.998851	-0.246483	1.065329
C	-2.676521	0.765402	0.090845
C	-2.280890	2.220401	0.429904
C	-4.156006	0.489209	0.295861
C	-3.423503	-1.647283	-0.396499
C	-2.254376	-1.686810	0.592834
H	-1.304734	2.208808	2.372496
H	1.561119	2.545514	0.243149
H	-3.128356	2.717534	0.921325
H	-3.878375	-2.627934	-0.574447
O	3.121360	0.216659	0.611441
C	1.700881	-1.889395	-1.606328
H	1.401733	-1.644342	-2.638479
H	2.792826	-1.977315	-1.574726
H	1.280013	-2.875966	-1.371790
C	-1.044188	-2.047244	-0.303434
H	-0.680585	-3.075887	-0.169921
H	-0.609281	3.366536	1.227854
H	-2.098362	2.767090	-0.505343
O	-5.003469	1.258301	0.661459
O	-4.479262	-0.788270	0.022428
C	-2.416067	0.393650	-1.413507
H	-1.354663	0.584360	-1.631130
C	-2.674930	-1.120137	-1.627890
H	-3.203796	-1.301128	-2.572595
H	-2.395178	-2.385759	1.423658
H	-2.412224	-0.067655	2.067760
C	-3.266775	1.220650	-2.377068
H	-4.338682	0.983947	-2.280296
H	-3.146969	2.301641	-2.219779
H	-2.976163	0.999307	-3.414829
O	-1.506318	-1.909396	-1.646718
O	0.042942	-0.722472	2.277512

C	-0.048931	-0.012109	3.484853
H	0.345798	-0.672377	4.268864
H	0.559494	0.909269	3.471770
H	-1.089054	0.250719	3.750824
C	2.332877	0.713620	-1.614335
H	3.145877	0.185063	-2.117727
H	1.557763	1.185895	-2.228245
B	4.186341	-0.765842	0.417801
F	5.218750	-0.425090	1.286976
F	4.667390	-0.725500	-0.922462
F	3.737411	-2.065691	0.684512
N	4.271550	3.205182	-1.404252
N	3.555965	2.383508	-1.509059

53

TS2'-B_3 Eopt -1583.882707

C	-1.974459	-0.155528	0.169011
C	-1.332197	0.603196	-0.983104
C	-0.109060	0.352396	-1.451734
C	0.623133	-0.867221	-0.978576
C	0.243677	-1.272924	0.418423
C	-0.975525	-1.087420	0.947022
C	0.712812	1.220742	-2.351237
C	2.136810	-0.684113	-0.930511
C	2.543205	0.768100	-0.557740
C	1.907768	1.779377	-1.542385
C	4.059784	0.736005	-0.638155
C	3.693964	-0.955893	0.970129
C	2.634458	-1.678837	0.132574
H	1.082159	0.616233	-3.195031
H	-1.914533	1.452672	-1.351103
H	2.682109	2.136580	-2.234999
H	4.311570	-1.631717	1.571802
O	-3.121159	-0.838647	-0.119693
C	-1.437083	-1.755408	2.217938
H	-1.191541	-1.171756	3.119118
H	-2.525455	-1.907794	2.187822
H	-0.957591	-2.737545	2.325781
C	1.456154	-1.849863	1.122301
H	1.316685	-2.876461	1.489105
H	0.124086	2.049208	-2.768584
H	1.561368	2.658788	-0.981507
O	4.773075	1.414522	-1.326321
O	4.596110	-0.185397	0.183499
C	2.238614	1.073123	0.953187
H	1.146395	1.113998	1.075395
C	2.764657	-0.093784	1.833798
H	3.242660	0.286603	2.745807
H	2.975296	-2.624768	-0.300433
H	2.544472	-0.911663	-1.924410
C	2.828479	2.403815	1.417023
H	3.930279	2.383940	1.415869
H	2.507754	3.244015	0.785353
H	2.506741	2.616926	2.447337
O	1.774077	-1.015112	2.238579
O	0.442301	-1.983920	-1.847987
C	-0.867984	-2.420539	-2.117535
H	-0.769400	-3.341777	-2.707632
H	-1.438788	-2.650784	-1.202272
H	-1.443091	-1.685407	-2.704799
C	-1.999943	0.749851	1.342407
H	-2.675422	0.535858	2.175273
H	-1.104297	1.342232	1.551387
B	-4.415020	-0.168411	-0.212281
F	-5.343426	-1.095809	-0.666060
F	-4.348923	0.936135	-1.087705
F	-4.797548	0.317094	1.063879

N	-3.551320	3.338222	0.667051
N	-3.023836	2.405474	0.888586
53			
TS2'-C_1		Eopt	-1583.891433
C	2.007633	0.461924	0.640642
C	1.093235	0.069917	1.781022
C	0.000446	-0.674638	1.618112
C	-0.301311	-1.266794	0.266998
C	0.263963	-0.434761	-0.855799
C	1.353589	0.332564	-0.737575
C	-1.071818	-0.932130	2.627384
C	-1.805054	-1.326599	-0.016878
C	-2.598378	-0.177757	0.668345
C	-2.348963	-0.176660	2.194630
C	-4.042794	-0.489179	0.314128
C	-3.117333	-0.306352	-1.848758
C	-1.939596	-1.236895	-1.545517
H	-1.270269	-2.014995	2.664836
H	1.365137	0.457635	2.768196
H	-3.222071	-0.612605	2.699022
H	-3.477931	-0.379380	-2.880743
O	3.285438	-0.105282	0.835592
C	1.860265	1.184596	-1.868810
H	1.477166	2.215915	-1.793986
H	2.955807	1.232831	-1.883118
H	1.526608	0.789754	-2.836507
C	-0.712242	-0.422142	-2.017968
H	-0.277002	-0.780460	-2.961718
H	-0.770716	-0.614276	3.635240
H	-2.270606	0.860861	2.548157
O	-4.957053	-0.682683	1.069596
O	-4.246345	-0.541729	-1.014078
C	-2.307685	1.199675	-0.027041
H	-1.262992	1.465986	0.186342
C	-2.433941	1.037434	-1.566917
H	-2.956843	1.894513	-2.011433
H	-2.012831	-2.220938	-2.020340
H	-2.185173	-2.281084	0.370777
C	-3.209513	2.326141	0.472742
H	-4.263585	2.157341	0.198621
H	-3.162255	2.447165	1.564022
H	-2.901644	3.279754	0.018132
O	-1.202360	0.905026	-2.238384
O	0.114585	-2.627614	0.197783
C	1.492036	-2.897854	0.294970
H	1.601922	-3.985035	0.180388
H	2.076277	-2.397130	-0.492250
H	1.909933	-2.605853	1.273975
C	2.601115	1.818183	0.905770
H	2.901456	2.020558	1.937138
H	3.212508	2.279870	0.126533
B	4.335535	-0.359838	-0.188274
F	5.340607	-1.052521	0.472686
F	4.837836	0.863791	-0.680632
F	3.836289	-1.109928	-1.255946
N	0.279702	3.572404	0.866507
N	1.226128	3.022294	0.879803
53			
TS2'-C_2		Eopt	-1583.887059
C	-2.128140	0.560359	0.205405
C	-1.541722	0.105849	-1.112752
C	-0.426406	-0.617484	-1.196880
C	0.186510	-1.178839	0.059332
C	-0.057445	-0.286989	1.253707
C	-1.150594	0.472295	1.375645
C	0.339343	-0.958262	-2.432556

C	1.722563	-1.289876	-0.024366
C	2.339885	-0.208167	-0.964306
C	1.734369	-0.299125	-2.381726
C	3.825731	-0.525403	-0.953268
C	3.464969	-0.166046	1.346932
C	2.245805	-1.089544	1.410916
H	0.446590	-2.054310	-2.472197
H	-2.054282	0.446404	-2.015646
H	2.426134	-0.856634	-3.027482
H	4.065529	-0.177885	2.263158
O	-3.398605	0.068192	0.571905
C	-1.435552	1.304801	2.591509
H	-1.229167	2.374543	2.420999
H	-2.494358	1.207038	2.878405
H	-0.821880	0.988652	3.444104
C	1.182226	-0.218897	2.123019
H	1.005152	-0.504546	3.170108
H	-0.195438	-0.646441	-3.340107
H	1.660635	0.710702	-2.807376
O	4.523654	-0.793372	-1.894035
O	4.352912	-0.484789	0.283034
C	2.233624	1.222175	-0.320234
H	1.169811	1.496243	-0.290392
C	2.738778	1.168527	1.147527
H	3.358976	2.043310	1.382972
H	2.443615	-2.034709	1.927466
H	1.990088	-2.277024	-0.428119
C	2.988355	2.292353	-1.106335
H	4.075917	2.113397	-1.100246
H	2.663258	2.351394	-2.154184
H	2.816510	3.278540	-0.649518
O	1.715131	1.109799	2.114870
O	-0.436012	-2.449330	0.189053

C	-0.107243	-3.236857	1.301930
H	-0.786537	-4.100242	1.289776
H	0.928818	-3.618814	1.263811
H	-0.255223	-2.701804	2.257693
C	-2.751698	1.925809	0.053042
H	-3.379362	2.046736	-0.834362
H	-3.073102	2.460576	0.949537
B	-4.348513	-0.560759	-0.391451
F	-5.563115	-0.651340	0.278978
F	-3.896516	-1.818691	-0.774048
F	-4.489775	0.256309	-1.534255
N	-0.538146	3.595104	-0.743138
N	-1.454890	3.078908	-0.440446

53

TS2'-C_3 Eopt -1583.888429

C	-2.116215	0.685377	0.049770
C	-1.551633	-0.172005	-1.062823
C	-0.451532	-0.909977	-0.914175
C	0.161916	-1.031779	0.456984
C	-0.032756	0.213970	1.280153
C	-1.110344	0.991363	1.159504
C	0.334470	-1.562891	-2.008943
C	1.683535	-1.218111	0.401223
C	2.345675	-0.460379	-0.791772
C	1.706960	-0.868754	-2.138597
C	3.804412	-0.867539	-0.679357
C	3.484377	0.129401	1.437679
C	2.202916	-0.655406	1.734694
H	0.483390	-2.626990	-1.769705
H	-2.049527	-0.106176	-2.033841
H	2.399941	-1.526557	-2.680806
H	4.092853	0.326217	2.327301
O	-3.364655	0.320801	0.597460

C	-1.381153	2.164468	2.055494
H	-1.206887	3.128306	1.548561
H	-2.428588	2.148277	2.395712
H	-0.735596	2.144874	2.942269
C	1.194300	0.449823	2.137324
H	0.973581	0.476326	3.213786
H	-0.200985	-1.515518	-2.967441
H	1.577734	0.028662	-2.759313
O	4.474897	-1.433692	-1.500261
O	4.339695	-0.528413	0.507825
C	2.356588	1.094323	-0.565312
H	1.320712	1.454861	-0.642772
C	2.853601	1.408092	0.870115
H	3.534337	2.269594	0.872756
H	2.320508	-1.428559	2.501186
H	1.923141	-2.285065	0.288301
C	3.205885	1.833373	-1.598802
H	4.276634	1.594718	-1.493309
H	2.910150	1.597379	-2.630591
H	3.097830	2.919658	-1.460963
O	1.823672	1.685861	1.790021
O	-0.428394	-2.060060	1.244286
C	-0.627988	-3.312659	0.643343
H	-1.042833	-3.971320	1.418571
H	-1.351112	-3.262893	-0.188074
H	0.311078	-3.766029	0.275884
C	-2.773820	1.916715	-0.520128
H	-3.428746	1.739051	-1.377812
H	-3.076125	2.711322	0.165635
B	-4.300003	-0.624028	-0.076857
F	-5.483745	-0.570149	0.648176
F	-3.776663	-1.916163	-0.077655
F	-4.526637	-0.211316	-1.407101
N	-0.584420	3.221675	-1.863032
N	-1.503431	2.850933	-1.397642

53

TS2-A_1 Eopt -1583.899753

C	2.269884	0.280146	-0.265222
C	1.402204	0.171037	-1.533865
C	0.141723	0.619553	-1.560384
C	-0.414257	1.329212	-0.345491
C	0.177491	0.803416	0.939040
C	1.418508	0.318300	1.013393
C	-0.852257	0.350229	-2.645290
C	-1.923043	1.088833	-0.181744
C	-2.372565	-0.330240	-0.660909
C	-1.945813	-0.599772	-2.119503
C	-3.881225	-0.273220	-0.490596
C	-3.192696	0.146646	1.726553
C	-2.191922	1.234675	1.324484
H	-1.310805	1.301157	-2.957817
H	1.869382	-0.342965	-2.375862
H	-2.826297	-0.536914	-2.773184
H	-3.676494	0.335306	2.691271
O	3.302558	-0.615950	-0.222259
C	2.026988	-0.261293	2.251782
H	1.479204	0.048174	3.150996
H	1.998188	-1.359710	2.193168
H	3.083950	0.021211	2.358182
C	-0.883534	0.784150	2.020312
H	-0.612611	1.401502	2.888951
H	-0.366691	-0.089623	-3.526774
H	-1.565969	-1.626958	-2.197445
O	-4.721849	-0.416655	-1.337346
O	-4.243350	-0.021786	0.780164
C	-1.907660	-1.464110	0.322720

H	-0.815999	-1.565342	0.236287
C	-2.234031	-1.051308	1.779989
H	-2.627326	-1.900764	2.353503
H	-2.500138	2.248717	1.601007
H	-2.487973	1.825656	-0.769335
C	-2.547170	-2.814394	0.000209
H	-3.635073	-2.805797	0.177847
H	-2.378009	-3.120684	-1.041315
H	-2.114321	-3.590893	0.648064
O	-1.134911	-0.537380	2.494099
O	-0.116868	2.729145	-0.353863
C	-0.456280	3.470950	-1.501400
H	-0.238068	4.522666	-1.271651
H	0.142723	3.175418	-2.381251
H	-1.526335	3.392379	-1.759392
C	2.777181	1.621410	-0.654212
H	3.491659	1.657229	-1.482942
H	2.114745	2.482587	-0.509719
B	3.058860	-2.043086	-0.459408
F	3.997612	-2.764629	0.278276
F	3.204261	-2.342464	-1.828606
F	1.751266	-2.399502	-0.062447
N	4.755814	2.296894	1.404489
N	4.026460	2.082837	0.617495

53

TS2-A_2 Eopt -1583.898747

C	-2.050231	-0.111266	-0.089042
C	-1.261155	-1.262111	0.583270
C	-0.033853	-1.072635	1.084014
C	0.544905	0.325871	1.102645
C	0.082506	1.140464	-0.081408
C	-1.113554	0.967269	-0.648739
C	0.904833	-2.149746	1.528753
C	2.075666	0.322945	0.976614
C	2.620539	-0.842702	0.088948
C	2.125820	-2.215855	0.590103
C	4.125954	-0.676572	0.215544
C	3.546327	1.426368	-0.684763
C	2.427114	1.673287	0.331894
H	1.242945	-1.921031	2.551589
H	-1.759314	-2.232461	0.558725
H	2.948237	-2.731561	1.103888
H	4.072189	2.339646	-0.984075
O	-2.889967	-0.564131	-1.064810
C	-1.600613	1.714837	-1.849942
H	-1.607340	1.049636	-2.726767
H	-2.636204	2.061751	-1.718731
H	-0.959771	2.576573	-2.074469
C	1.212394	2.030497	-0.559621
H	0.947621	3.097245	-0.534548
H	0.401359	-3.125312	1.559019
H	1.857282	-2.837124	-0.274502
O	4.918862	-1.473570	0.639470
O	4.544714	0.529467	-0.208011
C	2.329134	-0.614586	-1.437771
H	1.247578	-0.733000	-1.598757
C	2.706080	0.837187	-1.826367
H	3.210225	0.868541	-2.801069
H	2.659576	2.445900	1.072477
H	2.532958	0.214298	1.969894
C	3.067214	-1.611994	-2.330458
H	4.158652	-1.462789	-2.294969
H	2.861089	-2.655307	-2.054435
H	2.752086	-1.476302	-3.375626
O	1.608538	1.716352	-1.893561
O	0.134606	1.068386	2.255519

C	0.336844	0.489955	3.524189
H	0.050977	1.249707	4.263883
H	-0.294355	-0.402826	3.680129
H	1.390415	0.216730	3.704733
C	-2.660611	0.290846	1.210620
H	-3.457766	-0.335015	1.622563
H	-2.000929	0.809292	1.915032
B	-4.194178	-1.160734	-0.797355
F	-4.104632	-2.152974	0.207338
F	-4.660216	-1.713549	-1.983145
F	-5.094990	-0.171934	-0.334785
N	-4.347772	2.657959	0.688235
N	-3.759938	1.763298	0.914387

53

TS2-A_4 Eopt -1583.900325

C	-2.265240	0.193458	0.378099
C	-1.376984	-0.210843	1.568255
C	-0.125151	0.242765	1.688877
C	0.418276	1.219297	0.670284
C	-0.190015	1.005752	-0.697515
C	-1.434025	0.545314	-0.864236
C	0.861854	-0.156953	2.733413
C	1.934206	1.033032	0.439121
C	2.396919	-0.441948	0.674647
C	2.037257	-0.920728	2.094822
C	3.900890	-0.381280	0.460927
C	3.155251	0.388799	-1.633077
C	2.193596	1.416781	-1.030794
H	1.231699	0.767397	3.207175
H	-1.833617	-0.891392	2.288806
H	2.922283	-0.831216	2.738586
H	3.608654	0.722656	-2.572947
O	-3.295150	-0.671483	0.125794
C	-2.045606	0.241097	-2.195630
H	-1.519057	0.759475	-3.007070
H	-1.988437	-0.841140	-2.384091
H	-3.110235	0.512514	-2.224412
C	0.867335	1.127939	-1.774650
H	0.619699	1.879275	-2.539066
H	0.391407	-0.767194	3.515581
H	1.781570	-1.987597	2.062112
O	4.762736	-0.670105	1.247104
O	4.231597	0.056160	-0.765863
C	1.867489	-1.395950	-0.458694
H	0.775414	-1.479999	-0.357266
C	2.167466	-0.764477	-1.843835
H	2.524789	-1.522310	-2.553113
H	2.536629	2.450468	-1.146489
H	2.488269	1.673989	1.140288
C	2.466932	-2.799318	-0.380115
H	3.552358	-2.794294	-0.572835
H	2.299233	-3.273909	0.596267
H	1.999811	-3.440513	-1.142140
O	1.068544	-0.117854	-2.442578
O	0.103212	2.496904	1.229415
C	0.496483	3.639333	0.509238
H	0.052114	4.504303	1.020238
H	1.591536	3.773646	0.501130
H	0.128271	3.625340	-0.532245
C	-2.771669	1.389613	1.102663
H	-3.481584	1.204761	1.915250
H	-2.109607	2.258580	1.193362
B	-3.044016	-2.113270	0.028118
F	-3.981072	-2.652508	-0.853998
F	-3.181758	-2.720253	1.291796
F	-1.736143	-2.361061	-0.444930

N -4.754862 2.543413 -0.716624

N -4.023633 2.154993 -0.001274

53

TS2-A_5 Eopt -1583.899317

C 2.049674 -0.165201 -0.003211

C 1.237866 -1.012897 -1.010935

C 0.017793 -0.640749 -1.414623

C -0.550159 0.681934 -0.948827

C -0.068961 1.038969 0.440416

C 1.133436 0.672518 0.896966

C -0.903027 -1.420884 -2.291909

C -2.087987 0.647236 -0.817909

C -2.633298 -0.778192 -0.480764

C -2.187239 -1.810170 -1.534612

C -4.141318 -0.585606 -0.473548

C -3.519713 0.954253 1.194823

C -2.440265 1.611816 0.331239

H -1.159270 -0.772184 -3.146181

H 1.730047 -1.925993 -1.348705

H -3.001901 -1.960196 -2.255412

H -4.025128 1.659396 1.864067

O 2.890505 -0.923718 0.759278

C 1.626421 0.932524 2.285510

H 1.612830 -0.000153 2.869202

H 2.670455 1.279264 2.280966

H 1.003164 1.673534 2.801474

C -1.203805 1.615874 1.261443

H -0.971946 2.606161 1.680259

H -0.409819 -2.316210 -2.692678

H -2.023165 -2.778992 -1.045318

O -4.951940 -1.143732 -1.162652

O -4.540547 0.327616 0.428187

C -2.279762 -1.198769 0.992258

H -1.192831 -1.358167 1.050824

C -2.638593 -0.040439 1.959337

H -3.105140 -0.423326 2.876271

H -2.717941 2.610843 -0.021171

H -2.535904 0.957642 -1.773227

C -2.985078 -2.483250 1.426631

H -4.077849 -2.350521 1.482849

H -2.782699 -3.325149 0.750698

H -2.638295 -2.773317 2.429473

O -1.542659 0.754012 2.349072

O -0.113646 1.606272 -1.947866

C -0.481102 2.956521 -1.797392

H 0.062778 3.519550 -2.567921

H -1.560896 3.116827 -1.956605

H -0.198720 3.359478 -0.808369

C 2.658065 0.625220 -1.114131

H 3.445074 0.147719 -1.705802

H 2.002073 1.337362 -1.626911

B 4.188030 -1.405884 0.300494

F 4.087997 -2.005547 -0.977307

F 4.653653 -2.327018 1.230098

F 5.095252 -0.324751 0.191225

N 4.349552 2.673649 0.162225

N 3.772080 1.911185 -0.369244

53

TS2-B_1 Eopt -1583.899207

C 2.265649 0.209205 0.297752

C 1.574424 0.595035 -0.998156

C 0.339682 1.094482 -1.027251

C -0.386390 1.338085 0.277069

C 0.019746 0.351420 1.347210

C 1.241070 -0.194314 1.409319

C -0.489468 1.317480 -2.256453

C	-1.903889	1.122845	0.138989
C	-2.273002	0.017403	-0.903902
C	-1.655942	0.312521	-2.287598
C	-3.792112	0.062764	-0.920830
C	-3.416399	-0.422549	1.355316
C	-2.386364	0.697366	1.535834
H	-0.888625	2.343598	-2.251234
H	2.141360	0.390197	-1.909104
H	-2.441316	0.680495	-2.961807
H	-4.027083	-0.598950	2.247702
O	3.290449	-0.680201	0.143534
C	1.678870	-1.224143	2.401372
H	1.059283	-1.197609	3.307224
H	1.587145	-2.222098	1.948236
H	2.733221	-1.092088	2.679946
C	-1.185482	-0.036736	2.178148
H	-1.041705	0.193091	3.244010
H	0.119136	1.209423	-3.164780
H	-1.284332	-0.625951	-2.719462
O	-4.509276	0.285349	-1.858908
O	-4.326960	-0.176457	0.290255
C	-1.925354	-1.422207	-0.377337
H	-0.829883	-1.520110	-0.347969
C	-2.456637	-1.585763	1.068854
H	-2.910888	-2.574019	1.216260
H	-2.745144	1.541411	2.134504
H	-2.380499	2.056997	-0.189288
C	-2.484035	-2.527275	-1.273297
H	-3.586176	-2.546573	-1.261611
H	-2.159280	-2.424808	-2.317710
H	-2.133723	-3.505636	-0.912623
O	-1.471849	-1.426815	2.065201
O	-0.105459	2.623322	0.839635

C	-0.256872	3.751979	0.012378
H	-0.085472	4.631860	0.647137
H	0.479405	3.765380	-0.810487
H	-1.270768	3.831166	-0.416680
C	2.722465	1.380584	1.084005
H	2.080186	2.267531	1.120144
H	3.369275	1.186280	1.946490
B	3.093869	-1.910257	-0.632955
F	3.917306	-2.895785	-0.086617
F	3.442773	-1.698660	-1.980533
F	1.744156	-2.322551	-0.578095
N	4.831635	2.453423	-0.681204
N	4.084567	2.115721	0.043607

53

TS2-B_2 Eopt -1583.896522

C	-2.035374	-0.127399	0.130656
C	-1.248739	0.783435	1.057976
C	-0.046273	1.266793	0.745454
C	0.541500	0.942796	-0.611158
C	0.110607	-0.421049	-1.101573
C	-1.083483	-0.952316	-0.809091
C	0.868266	2.026894	1.659119
C	2.076290	0.855207	-0.572360
C	2.632010	0.337545	0.794079
C	2.123789	1.191718	1.974697
C	4.136717	0.437890	0.603421
C	3.588916	-1.017273	-1.170414
C	2.457546	-0.126486	-1.693169
H	1.169110	2.966847	1.171261
H	-1.733320	0.984154	2.017116
H	2.930870	1.856398	2.311530
H	4.125537	-1.548607	-1.964089
O	-2.847926	-0.957357	0.845773

C	-1.550919	-2.315778	-1.206069
H	-1.418848	-3.012049	-0.363887
H	-2.621394	-2.312680	-1.449156
H	-0.979864	-2.699351	-2.061664
C	1.265263	-1.105834	-1.803507
H	1.010323	-1.381558	-2.837090
H	0.354337	2.295940	2.592178
H	1.891344	0.529913	2.819410
O	4.917531	1.076615	1.255826
O	4.572110	-0.295334	-0.437228
C	2.364671	-1.197587	0.994400
H	1.283906	-1.337122	1.147721
C	2.760283	-1.964790	-0.291684
H	3.277257	-2.902996	-0.051996
H	2.687247	0.379209	-2.637121
H	2.505451	1.852652	-0.740935
C	3.106193	-1.771378	2.201580
H	4.198393	-1.755767	2.054463
H	2.883819	-1.227383	3.129817
H	2.811063	-2.820317	2.352259
O	1.668851	-2.289858	-1.122278
O	0.117142	1.849637	-1.634387
C	0.232710	3.231093	-1.386647
H	-0.062440	3.740943	-2.313608
H	-0.437324	3.564378	-0.574718
H	1.266021	3.532903	-1.143696
C	-2.606023	0.601943	-1.035598
H	-1.932053	1.295858	-1.550125
H	-3.382621	0.105203	-1.625919
B	-4.291091	-1.074780	0.692680
F	-4.918547	0.155661	0.986981
F	-4.730449	-2.060971	1.567062
F	-4.641191	-1.422354	-0.638954

N	-4.198385	2.884834	-0.030314
N	-3.708049	1.985757	-0.415867

53

TS2-B_3 Eopt -1583.897974

C	-2.023820	-0.315324	-0.090616
C	-1.347875	0.584326	0.931442
C	-0.158944	1.147099	0.713742
C	0.519234	0.935009	-0.622397
C	0.192931	-0.417803	-1.212757
C	-0.981911	-1.029939	-1.014373
C	0.663768	1.895822	1.719722
C	2.052229	0.925231	-0.500369
C	2.560158	0.352709	0.862703
C	1.940691	1.104002	2.060224
C	4.065228	0.542703	0.765350
C	3.696038	-0.825555	-1.120280
C	2.547495	0.036611	-1.653613
H	0.943065	2.876427	1.304187
H	-1.894514	0.704815	1.868892
H	2.690950	1.783706	2.486479
H	4.303158	-1.278798	-1.911594
O	-2.833743	-1.253942	0.479397
C	-1.351286	-2.387224	-1.521806
H	-1.302750	-3.118064	-0.701031
H	-2.382433	-2.405052	-1.901709
H	-0.668580	-2.712651	-2.317173
C	1.419004	-0.994484	-1.890521
H	1.234427	-1.216208	-2.951631
H	0.086359	2.084332	2.635215
H	1.694001	0.378380	2.846427
O	4.772978	1.180021	1.497519
O	4.596558	-0.100877	-0.290231
C	2.366566	-1.203591	0.955407

H	1.288344	-1.407615	1.036517
C	2.874572	-1.867467	-0.348619
H	3.428552	-2.790777	-0.135617
H	2.800011	0.611269	-2.551076
H	2.434247	1.951954	-0.584906
C	3.067945	-1.813401	2.168892
H	4.164189	-1.728996	2.092379
H	2.758990	-1.343847	3.112872
H	2.825664	-2.884349	2.235943
O	1.850002	-2.196408	-1.259698
O	0.101697	1.872757	-1.619839
C	0.126286	3.242382	-1.292439
H	-0.147648	3.787608	-2.205663
H	-0.603084	3.492701	-0.502181
H	1.126376	3.584907	-0.975780
C	-2.568578	0.434007	-1.258567
H	-1.931949	1.219870	-1.680254
H	-3.252034	-0.089402	-1.934103
B	-4.208286	-0.970848	0.869041
F	-4.691659	-2.073456	1.562859
F	-5.001888	-0.736995	-0.281718
F	-4.276566	0.187154	1.675975
N	-4.519088	2.457433	-0.280147
N	-3.863941	1.668368	-0.661194

53

TS2-B_4 Eopt -1583.899639

C	2.270238	0.322363	0.162023
C	1.551955	0.335660	-1.175449
C	0.313216	0.800520	-1.313687
C	-0.406688	1.379159	-0.116598
C	0.031005	0.719742	1.175375
C	1.268894	0.240074	1.362545
C	-0.508696	0.801081	-2.562551
C	-1.929864	1.116196	-0.169230
C	-2.281582	-0.183027	-0.964371
C	-1.729829	-0.123207	-2.402360
C	-3.801473	-0.217865	-0.945179
C	-3.362932	-0.225197	1.365076
C	-2.395492	0.959427	1.291933
H	-0.845473	1.835026	-2.744317
H	2.120063	-0.063798	-2.018187
H	-2.531539	0.204957	-3.077406
H	-3.945161	-0.248043	2.292870
O	3.296067	-0.575041	0.245769
C	1.738309	-0.481889	2.585014
H	1.135913	-0.213575	3.462811
H	1.650601	-1.565550	2.421328
H	2.796249	-0.270351	2.792413
C	-1.161303	0.441529	2.066264
H	-1.048583	0.879727	3.069123
H	0.088875	0.493057	-3.431129
H	-1.449229	-1.134893	-2.722720
O	-4.543790	-0.223783	-1.890094
O	-4.304267	-0.242699	0.301248
C	-1.840222	-1.468799	-0.172357
H	-0.740848	-1.499172	-0.146737
C	-2.344222	-1.368248	1.291080
H	-2.739554	-2.330640	1.640458
H	-2.808679	1.884128	1.708838
H	-2.421822	1.960377	-0.673994
C	-2.338946	-2.762973	-0.813275
H	-3.438641	-2.836371	-0.789806
H	-2.015041	-2.865492	-1.857754
H	-1.938456	-3.624561	-0.259019
O	-1.362509	-0.961314	2.219134
O	-0.088379	2.770259	-0.153688

C	-0.648982	3.594863	0.839283
H	-0.198654	4.589177	0.716713
H	-1.741705	3.699768	0.730293
H	-0.423099	3.235987	1.859724
C	2.734417	1.668972	0.569720
H	2.094650	2.526400	0.332233
H	3.382635	1.737826	1.449452
B	3.094848	-1.972614	-0.157479
F	3.938755	-2.765218	0.621841
F	3.414626	-2.140733	-1.517457
F	1.750739	-2.358894	0.039365
N	4.854130	2.146177	-1.428498
N	4.100498	2.057113	-0.640075

53

TS2-B_5 Eopt -1583.896812

C	2.041160	-0.065862	-0.153901
C	1.225013	0.758990	-1.134684
C	0.022204	1.247452	-0.839156
C	-0.556769	1.014304	0.539550
C	-0.094520	-0.306865	1.120536
C	1.112711	-0.828907	0.860450
C	-0.875515	2.035514	-1.739069
C	-2.097733	0.895871	0.516846
C	-2.643381	0.373359	-0.851159
C	-2.187073	1.275380	-2.013991
C	-4.152862	0.401828	-0.668904
C	-3.553618	-1.040849	1.090921
C	-2.472473	-0.101229	1.630883
H	-1.099576	2.987933	-1.230763
H	1.709570	0.933158	-2.098891
H	-2.986065	1.993602	-2.241591
H	-4.067530	-1.603067	1.878420
O	2.855228	-0.938754	-0.813670
C	1.595687	-2.164674	1.326883
H	1.497378	-2.898291	0.512668
H	2.659572	-2.128120	1.594152
H	1.012066	-2.521317	2.185312
C	-1.248670	-1.032885	1.779674
H	-1.027676	-1.314085	2.820023
H	-0.372682	2.280369	-2.684393
H	-2.057962	0.662098	-2.915299
O	-4.956038	1.010434	-1.322888
O	-4.564556	-0.362528	0.357703
C	-2.297794	-1.145841	-1.061753
H	-1.209885	-1.231949	-1.201688
C	-2.671459	-1.939854	0.216421
H	-3.140531	-2.899431	-0.036864
H	-2.753213	0.392894	2.567172
H	-2.533170	1.891139	0.687239
C	-2.992469	-1.751656	-2.280636
H	-4.087523	-1.776274	-2.157644
H	-2.769316	-1.205716	-3.207289
H	-2.654928	-2.789468	-2.418921
O	-1.579473	-2.221241	1.063377
O	-0.109261	2.134010	1.304262
C	-0.521368	2.214519	2.648710
H	0.021504	3.058496	3.095113
H	-1.601919	2.414609	2.744876
H	-0.271863	1.301162	3.218337
C	2.620437	0.778704	0.924282
H	1.949871	1.532204	1.351932
H	3.404650	0.353673	1.558430
B	4.295587	-1.069222	-0.636388
F	4.950420	0.115994	-1.035476
F	4.720640	-2.138723	-1.414662
F	4.631162	-1.300803	0.723851

N 4.211631 2.932981 -0.350326

N 3.717663 2.087751 0.138399

53

TS2-B_6 Eopt -1583.898016

C	-2.038714	-0.223668	-0.144800
C	-1.337846	0.326991	1.086615
C	-0.144345	0.914199	1.028171
C	0.530611	1.097804	-0.313438
C	0.174937	-0.021079	-1.272309
C	-1.016130	-0.637224	-1.256064
C	0.654803	1.447107	2.174709
C	2.071481	1.029283	-0.212876
C	2.559425	0.161853	0.991929
C	1.986122	0.685774	2.322842
C	4.073531	0.291802	0.934340
C	3.658303	-0.619775	-1.194892
C	2.565441	0.398445	-1.529894
H	0.858372	2.511110	1.969084
H	-1.894087	0.214515	2.019334
H	2.730624	1.338471	2.797946
H	4.247515	-0.921291	-2.068040
O	-2.847464	-1.288210	0.128829
C	-1.403040	-1.793430	-2.122083
H	-1.326611	-2.730704	-1.551588
H	-2.446653	-1.708656	-2.455836
H	-0.749284	-1.867116	-3.000773
C	1.403190	-0.492798	-2.022159
H	1.260545	-0.477445	-3.112848
H	0.083275	1.399712	3.111442
H	1.836369	-0.159621	3.007003
O	4.802583	0.722874	1.786229
O	4.586219	-0.141928	-0.230677
C	2.281952	-1.366157	0.746555
H	1.193895	-1.525333	0.779380
C	2.772042	-1.756260	-0.671611
H	3.272786	-2.732953	-0.662484
H	2.878937	1.143868	-2.268556
H	2.464186	2.046145	-0.067906
C	2.933179	-2.266122	1.796160
H	4.033428	-2.220596	1.749670
H	2.627867	-2.007311	2.819030
H	2.640461	-3.311424	1.618059
O	1.749179	-1.823025	-1.641026
O	0.071537	2.371363	-0.766836
C	0.568077	2.847266	-1.995629
H	0.012599	3.766349	-2.225825
H	1.640448	3.100496	-1.944708
H	0.406635	2.129151	-2.819906
C	-2.598078	0.850703	-1.010431
H	-1.961656	1.728786	-1.163154
H	-3.297623	0.568829	-1.802691
B	-4.215608	-1.131461	0.603129
F	-4.700503	-2.391016	0.933546
F	-5.018260	-0.553618	-0.412271
F	-4.266839	-0.275363	1.726000
N	-4.507499	2.491278	0.578044
N	-3.868168	1.848069	-0.033861

53

TS2-C_1 Eopt -1583.893992

C	2.211781	0.235359	0.225039
C	1.560792	-0.060246	-1.105701
C	0.322123	0.334886	-1.394413
C	-0.445132	1.191051	-0.418495
C	-0.027794	0.930200	1.008976
C	1.208499	0.538061	1.334795
C	-0.462093	-0.013532	-2.618065

C	-1.957809	0.885545	-0.433370
C	-2.259266	-0.589774	-0.845610
C	-1.653305	-0.913131	-2.227790
C	-3.777660	-0.651030	-0.858113
C	-3.410496	-0.007246	1.377001
C	-2.462784	1.134787	1.000939
H	-0.831475	0.921663	-3.069287
H	2.158483	-0.616105	-1.828776
H	-2.440665	-0.832144	-2.989373
H	-4.022532	0.212095	2.258968
O	3.124628	-0.734258	0.688461
C	1.637894	0.221676	2.735112
H	1.715028	-0.866681	2.874517
H	2.632404	0.640174	2.953348
H	0.924479	0.614412	3.470243
C	-1.240629	0.892028	1.918167
H	-1.178429	1.590878	2.765358
H	0.162732	-0.519260	-3.366739
H	-1.317197	-1.958435	-2.236904
O	-4.491624	-0.928071	-1.784268
O	-4.317133	-0.343664	0.334723
C	-1.827266	-1.595311	0.284115
H	-0.728691	-1.597619	0.336669
C	-2.368655	-1.097614	1.651175
H	-2.757007	-1.933663	2.247331
H	-2.908307	2.127025	1.129747
H	-2.450299	1.542116	-1.165239
C	-2.300580	-3.023633	0.017631
H	-3.399463	-3.104691	0.048779
H	-1.961980	-3.401933	-0.956576
H	-1.899591	-3.695959	0.790525
O	-1.423086	-0.422578	2.449618
O	-0.163982	2.524128	-0.842538
C	-0.748831	3.577279	-0.120732
H	-0.316630	4.510376	-0.508686
H	-1.842670	3.631134	-0.258918
H	-0.529162	3.519386	0.961181
C	3.387472	1.169210	0.131986
H	3.809283	1.541140	1.069790
H	4.085469	0.963275	-0.684402
B	3.669280	-1.853137	-0.131148
F	4.276613	-1.344112	-1.295937
F	2.641269	-2.729698	-0.478823
F	4.613969	-2.485970	0.664762
N	2.415740	3.690023	-0.926953
N	2.843569	2.781502	-0.493001

53

TS2-C_2 Eopt -1583.893330

C	2.196838	0.150033	0.303896
C	1.564751	0.073042	-1.067444
C	0.327494	0.508589	-1.309720
C	-0.438006	1.214953	-0.218592
C	-0.045561	0.731388	1.152844
C	1.176299	0.272317	1.433296
C	-0.469314	0.233801	-2.549678
C	-1.946360	0.938691	-0.296567
C	-2.275900	-0.490340	-0.831737
C	-1.614207	-0.742330	-2.205463
C	-3.793426	-0.479517	-0.900973
C	-3.477597	-0.024993	1.393568
C	-2.459617	1.092205	1.144025
H	-0.892439	1.171617	-2.940811
H	2.154870	-0.406673	-1.848897
H	-2.382917	-0.696078	-2.989044
H	-4.113675	0.151688	2.268025
O	3.103760	-0.887296	0.603723

C	1.589985	-0.232599	2.781747
H	1.723882	-1.324142	2.756371
H	2.553969	0.197327	3.095307
H	0.839544	0.005808	3.545370
C	-1.268034	0.694655	2.049642
H	-1.165207	1.323900	2.945228
H	0.161090	-0.197639	-3.339494
H	-1.206585	-1.761968	-2.223647
O	-4.486553	-0.654138	-1.867053
O	-4.359169	-0.231195	0.294555
C	-1.938930	-1.603198	0.225013
H	-0.844538	-1.666844	0.312559
C	-2.502758	-1.190519	1.609587
H	-2.954821	-2.048839	2.123678
H	-2.841224	2.097771	1.351011
H	-2.423920	1.658503	-0.975640
C	-2.474730	-2.976221	-0.179304
H	-3.576728	-3.001959	-0.180226
H	-2.130040	-3.282725	-1.176561
H	-2.129325	-3.734733	0.538723
O	-1.547019	-0.636620	2.484256
O	-0.191952	2.622748	-0.193882
C	-0.357377	3.343288	-1.388890
H	-0.228815	4.405818	-1.139597
H	0.395990	3.070461	-2.150055
H	-1.361722	3.220074	-1.830556
C	3.366973	1.089738	0.402343
H	3.787784	1.260732	1.397239
H	4.067145	1.061808	-0.437629
B	3.728034	-1.805135	-0.390432
F	4.324528	-1.058941	-1.426981
F	2.763094	-2.660066	-0.922451
F	4.695759	-2.518148	0.303470
N	2.430596	3.817096	0.044354
N	2.821032	2.801515	0.156521

65

TS2a-Al''_1 Eopt -3290.915014

C	-1.265531	0.344870	-0.472837
C	-0.702712	0.476680	0.923052
C	0.527244	0.926607	1.172197
C	1.347891	1.471271	0.029912
C	1.024492	0.791688	-1.277060
C	-0.180460	0.283938	-1.549787
C	1.263347	0.808104	2.471850
C	2.848654	1.215240	0.222254
C	3.148292	-0.130608	0.954679
C	2.408591	-0.212857	2.309222
C	4.658881	-0.097301	1.104768
C	4.468679	0.051789	-1.244324
C	3.434128	1.180873	-1.198194
H	1.679389	1.786612	2.756642
H	-1.332212	0.075601	1.722354
H	3.132970	-0.085401	3.125379
H	5.152354	0.119938	-2.097844
O	-2.160340	-0.725592	-0.557522
C	-0.519473	-0.430791	-2.822508
H	-0.490481	-1.518971	-2.659939
H	-1.532589	-0.184393	-3.174483
H	0.194757	-0.190187	-3.620439
C	2.290553	0.651745	-2.100788
H	2.225061	1.160398	-3.073529
H	0.592230	0.486968	3.280667
H	1.980231	-1.218211	2.419985
O	5.298884	-0.138583	2.121371
O	5.288611	-0.001151	-0.080809
C	2.870755	-1.372298	0.033856

H	1.784065	-1.456407	-0.108905
C	3.511186	-1.141942	-1.358360
H	3.993178	-2.055918	-1.729213
H	3.820660	2.154193	-1.518824
H	3.289882	2.021579	0.824926
C	3.373897	-2.679706	0.643085
H	4.473307	-2.700601	0.722209
H	2.959777	-2.859854	1.644834
H	3.073362	-3.523786	0.005172
O	2.603456	-0.717556	-2.349321
O	1.114144	2.859770	-0.208143
C	1.171263	3.734110	0.889098
H	2.139520	3.694420	1.419001
H	1.045536	4.751133	0.491979
H	0.363587	3.542428	1.618543
C	-2.420917	1.270475	-0.808811
H	-2.730210	1.153283	-1.856650
N	-1.260984	3.951281	-1.396681
N	-1.657034	2.955885	-1.172347
Si	-3.852903	1.827026	0.406977
C	-4.801830	0.296286	0.877172
H	-5.119768	-0.270939	-0.010447
H	-4.209039	-0.368194	1.522071
H	-5.704767	0.600378	1.432834
C	-3.102731	2.710587	1.871841
H	-2.525799	2.034154	2.519072
H	-2.449767	3.542850	1.564205
H	-3.920598	3.138291	2.475925
C	-4.889464	2.992537	-0.625389
H	-5.254313	2.500966	-1.541440
H	-5.770257	3.304089	-0.039712
H	-4.344679	3.906027	-0.912132
Al	-2.206327	-2.394619	0.035413
Cl	-2.491350	-2.469349	2.182229
Cl	-3.855849	-3.321630	-0.986383
Cl	-0.324143	-3.350303	-0.434156

65

TS2a-Al''_2 Eopt -3290.914829

C	-1.263446	0.380047	-0.425343
C	-0.690867	0.386221	0.972100
C	0.540268	0.810532	1.247115
C	1.362905	1.438942	0.151819
C	1.032107	0.859566	-1.206185
C	-0.181595	0.383093	-1.507254
C	1.254117	0.716637	2.555658
C	2.871529	1.163397	0.320275
C	3.150081	-0.191511	1.046102
C	2.458761	-0.236837	2.425274
C	4.664807	-0.225393	1.164699
C	4.438710	-0.061594	-1.173275
C	3.460946	1.114265	-1.102405
H	1.603542	1.726191	2.827526
H	-1.332732	-0.036245	1.749655
H	3.198059	-0.004093	3.203511
H	5.105166	-0.017536	-2.041862
O	-2.185621	-0.655749	-0.595307
C	-0.529719	-0.259500	-2.814637
H	-1.536641	0.030267	-3.152171
H	0.192711	0.005105	-3.597356
H	-0.526952	-1.354670	-2.706603
C	2.297599	0.658017	-2.016911
H	2.275860	1.168846	-2.991019
H	0.584128	0.371771	3.355153
H	2.113270	-1.260667	2.620201
O	5.319325	-0.299464	2.170081
O	5.278449	-0.157477	-0.029828

C	2.792952	-1.413742	0.123704
H	1.701207	-1.442038	-0.003077
C	3.424083	-1.205278	-1.278358
H	3.855518	-2.140098	-1.659146
H	3.905817	2.065823	-1.412322
H	3.319682	1.963290	0.927523
C	3.238243	-2.751754	0.710740
H	2.815312	-2.931782	1.708509
H	2.900279	-3.570145	0.058395
H	4.335658	-2.822726	0.788934
O	2.531218	-0.729640	-2.260240
O	1.051290	2.828526	0.253427
C	1.722770	3.714379	-0.603204
H	1.276716	4.707515	-0.451141
H	2.799208	3.795257	-0.373221
H	1.607580	3.441535	-1.668987
C	-2.385833	1.371633	-0.671694
H	-2.721637	1.330095	-1.717510
N	-1.295650	4.064949	-1.232167
N	-1.584093	3.043803	-0.962025
Si	-3.763829	1.892667	0.620949
C	-4.780564	0.372033	0.966646
H	-4.208675	-0.381092	1.527312
H	-5.653311	0.664803	1.574266
H	-5.149057	-0.088493	0.037665
C	-2.927967	2.598531	2.134960
H	-2.114681	3.293150	1.870945
H	-3.672418	3.159664	2.724087
H	-2.510028	1.811540	2.779567
C	-4.769339	3.192497	-0.273726
H	-5.644805	3.448838	0.345928
H	-4.210487	4.124510	-0.452115
H	-5.144159	2.818068	-1.239799
Al	-2.297410	-2.356660	-0.117076
Cl	-0.451999	-3.350066	-0.649495
Cl	-2.599689	-2.576700	2.017270
Cl	-3.976934	-3.148139	-1.204625

65

TS2a-Al''_3 Eopt -3290.908458

C	-1.026872	0.203690	-0.063014
C	-0.272017	0.682647	1.160646
C	1.000322	1.077064	1.126192
C	1.681166	1.179844	-0.214648
C	1.162844	0.155235	-1.191948
C	-0.093010	-0.299766	-1.168198
C	1.893580	1.317368	2.305173
C	3.185811	0.892452	-0.131202
C	3.545179	-0.166487	0.958726
C	3.001161	0.243793	2.345400
C	5.062974	-0.197081	0.904910
C	4.553987	-0.794105	-1.321902
C	3.572241	0.364539	-1.522423
H	2.354012	2.313945	2.220126
H	-0.822829	0.596015	2.101365
H	3.833595	0.599822	2.967717
H	5.114400	-1.053403	-2.226981
O	-1.985245	-0.711640	0.357865
C	-0.624122	-1.301938	-2.145493
H	-0.632890	-2.308858	-1.703439
H	-1.658484	-1.076904	-2.448363
H	-0.005892	-1.340338	-3.051535
C	2.301450	-0.337022	-2.063007
H	2.125396	-0.166504	-3.135026
H	1.323653	1.299958	3.244688
H	2.594656	-0.645712	2.845186
O	5.834904	0.044386	1.793771

O	5.525186	-0.533404	-0.313214
C	3.109214	-1.615995	0.536511
H	2.010909	-1.662457	0.566561
C	3.552909	-1.887333	-0.923997
H	3.950537	-2.904746	-1.033703
H	3.943004	1.153964	-2.184977
H	3.727645	1.815579	0.118100
C	3.668143	-2.690325	1.468979
H	4.765464	-2.764402	1.396324
H	3.412788	-2.505950	2.521682
H	3.254337	-3.671744	1.193660
O	2.528130	-1.734294	-1.878200
O	1.450819	2.436880	-0.852407
C	1.690191	3.606731	-0.112700
H	1.499352	4.452615	-0.788041
H	1.015947	3.696859	0.756598
H	2.733562	3.684195	0.241819
C	-2.105595	1.147804	-0.568826
H	-2.642258	0.715271	-1.424820
N	-0.884037	3.110742	-2.459320
N	-1.271045	2.403334	-1.719027
Si	-3.143878	2.391900	0.530797
C	-4.414110	3.083807	-0.651381
H	-5.034183	2.277718	-1.075228
H	-5.082637	3.775136	-0.112283
H	-3.955699	3.643986	-1.481944
C	-3.928216	1.420594	1.909006
H	-4.643724	0.678070	1.527857
H	-3.185993	0.897187	2.527923
H	-4.479653	2.127090	2.552121
C	-1.991126	3.716817	1.171042
H	-2.590167	4.444710	1.743968
H	-1.228087	3.303526	1.847185
H	-1.485891	4.269788	0.363940
Al	-3.179003	-1.988075	0.168670
Cl	-4.257283	-2.176465	2.025380
Cl	-4.527334	-1.382637	-1.423504
Cl	-2.260733	-3.894664	-0.258084

65

TS2a-Al''_4 Eopt -3290.914918

C	-1.204487	0.442544	-0.409271
C	-0.618202	0.424121	0.982440
C	0.621514	0.832148	1.249010
C	1.453338	1.450037	0.152907
C	1.097895	0.884347	-1.204552
C	-0.126175	0.428709	-1.495213
C	1.342783	0.715840	2.552482
C	2.956261	1.133606	0.308732
C	3.211191	-0.227990	1.029471
C	2.526617	-0.262399	2.412118
C	4.725548	-0.293049	1.136349
C	4.483922	-0.121071	-1.200471
C	3.532904	1.075489	-1.118778
H	1.715410	1.716532	2.826223
H	-1.252212	0.002112	1.765203
H	3.274644	-0.047510	3.187220
H	5.144485	-0.089379	-2.074089
O	-2.146653	-0.570982	-0.608218
C	-0.480035	-0.239146	-2.788919
H	-1.498860	0.015381	-3.116293
H	0.225436	0.028451	-3.586013
H	-0.446083	-1.332518	-2.663077
C	2.351986	0.648648	-2.024096
H	2.336193	1.159390	-2.998418
H	0.670962	0.381965	3.355092
H	2.160437	-1.279206	2.606025

O	5.386753	-0.381033	2.136190
O	5.330446	-0.237744	-0.063612
C	2.823866	-1.441173	0.108618
H	1.730722	-1.449505	-0.006124
C	3.443704	-1.241976	-1.298708
H	3.850660	-2.184496	-1.687423
H	3.996200	2.017803	-1.429775
H	3.427191	1.922058	0.913802
C	3.250790	-2.788111	0.688984
H	4.347370	-2.877017	0.760911
H	2.830408	-2.965105	1.688380
H	2.897129	-3.598973	0.035435
O	2.548563	-0.744905	-2.268309
O	1.187466	2.846501	0.267836
C	1.864260	3.715607	-0.602092
H	2.947523	3.765735	-0.396698
H	1.715876	3.449569	-1.665263
H	1.450242	4.720631	-0.438155
C	-2.324175	1.438154	-0.651482
H	-2.571663	1.496489	-1.722172
N	-1.237897	4.182534	-0.748388
N	-1.549228	3.134821	-0.688800
Si	-3.890217	1.728472	0.493218
C	-5.133900	0.432350	-0.011556
H	-5.184937	0.305627	-1.103561
H	-4.897470	-0.539469	0.444959
H	-6.130812	0.741292	0.344241
C	-3.434402	1.643031	2.297782
H	-2.548698	2.253515	2.532945
H	-4.281275	2.036131	2.885260
H	-3.254552	0.607391	2.622060
C	-4.506491	3.437590	0.032117
H	-5.522034	3.556932	0.445011
H	-3.887049	4.244431	0.453963
H	-4.575634	3.577633	-1.058925
Al	-2.345862	-2.252961	-0.094431
Cl	-3.777641	-3.124155	-1.440552
Cl	-0.429100	-3.238728	-0.215808
Cl	-3.075926	-2.318648	1.943172

65

TS2a-Al'_1 Eopt -3290.918518

C	-1.285323	0.244990	0.307331
C	-0.410287	-0.199504	1.474693
C	0.829763	0.252763	1.645786
C	1.391418	1.260703	0.665224
C	0.826429	1.051456	-0.725596
C	-0.408140	0.574437	-0.936951
C	1.785616	-0.126561	2.730940
C	2.917925	1.101384	0.473293
C	3.407712	-0.360428	0.734793
C	3.023337	-0.834706	2.149496
C	4.915115	-0.270128	0.559046
C	4.210522	0.468979	-1.559765
C	3.214023	1.482446	-0.990537
H	2.094303	0.801735	3.239837
H	-0.878707	-0.891771	2.181063
H	3.880625	-0.686493	2.819722
H	4.681118	0.804395	-2.490614
O	-2.297634	-0.625952	0.034236
C	-0.938914	0.172662	-2.277078
H	-0.443428	0.723785	-3.087004
H	-0.737627	-0.898759	-2.432360
H	-2.024582	0.309111	-2.354006
C	1.917001	1.164723	-1.770035
H	1.674565	1.903706	-2.548423
H	1.300253	-0.765085	3.481412

H	2.832234	-1.915304	2.126070
O	5.762159	-0.534522	1.369330
O	5.269636	0.164086	-0.662873
C	2.926875	-1.331512	-0.404057
H	1.833663	-1.431070	-0.331116
C	3.251576	-0.705517	-1.784732
H	3.639995	-1.460243	-2.480594
H	3.539694	2.522208	-1.102185
H	3.436578	1.758783	1.186333
C	3.537036	-2.728040	-0.299555
H	4.629983	-2.712373	-0.442913
H	3.328967	-3.205281	0.667623
H	3.111468	-3.373248	-1.082187
O	2.151569	-0.086270	-2.413552
O	1.046129	2.527196	1.226437
C	1.432878	3.678634	0.516257
H	0.956244	4.534207	1.013968
H	2.524657	3.836334	0.535802
H	1.093841	3.655516	-0.535172
C	-1.826581	1.631122	0.533463
H	-1.092042	2.301132	1.009082
N	-3.425780	0.992039	2.977229
N	-2.818786	1.319690	2.126770
Si	-3.199097	2.548548	-0.506446
C	-3.873892	3.852094	0.655723
H	-4.497126	3.425380	1.457150
H	-3.067873	4.446316	1.115625
H	-4.510861	4.543451	0.079115
C	-2.257570	3.379098	-1.893163
H	-2.952502	4.056828	-2.417007
H	-1.430510	3.989056	-1.496503
H	-1.854288	2.674076	-2.632606
C	-4.527595	1.360713	-1.043861

H	-4.139875	0.532824	-1.652930
H	-5.025016	0.920823	-0.165132
H	-5.288948	1.904766	-1.627113
Al	-2.420006	-2.366673	-0.105620
Cl	-0.495960	-3.204220	-0.663201
Cl	-3.044355	-3.182500	1.799857
Cl	-3.898734	-2.770631	-1.624452

65

TS2a-Al'_2 Eopt -3290.917496

C	1.296318	-0.262340	0.277653
C	0.432774	0.092935	1.483565
C	-0.810017	-0.364926	1.630103
C	-1.369520	-1.305650	0.583436
C	-0.812043	-1.012731	-0.790364
C	0.411001	-0.502594	-0.981190
C	-1.794875	0.070057	2.673447
C	-2.886936	-1.124000	0.403830
C	-3.364408	0.348262	0.633561
C	-2.922795	0.883848	2.012795
C	-4.873777	0.231511	0.503372
C	-4.210391	-0.562605	-1.616586
C	-3.178473	-1.541105	-1.046492
H	-2.221837	-0.815196	3.169970
H	0.892678	0.768940	2.211298
H	-3.792489	0.921169	2.682884
H	-4.703695	-0.929738	-2.523322
O	2.304469	0.632084	0.069935
C	0.932451	-0.021396	-2.299054
H	0.447945	-0.538513	-3.137537
H	0.707957	1.052423	-2.397590
H	2.020706	-0.127828	-2.383511
C	-1.893383	-1.193819	-1.836440
H	-1.613353	-1.950914	-2.583618

H	-1.304755	0.674882	3.448860
H	-2.568905	1.916690	1.895983
O	-5.705505	0.505834	1.326199
O	-5.249673	-0.249604	-0.695781
C	-2.937348	1.294274	-0.545319
H	-1.845496	1.420815	-0.501573
C	-3.281387	0.626090	-1.898766
H	-3.701899	1.351887	-2.606710
H	-3.465796	-2.594443	-1.133124
H	-3.422127	-1.756725	1.125486
C	-3.579906	2.677674	-0.456798
H	-4.672790	2.633196	-0.594306
H	-3.380295	3.172480	0.503513
H	-3.173989	3.322312	-1.250391
O	-2.178725	0.016169	-2.530660
O	-1.052041	-2.676850	0.841016
C	-1.331542	-3.191156	2.120894
H	-1.095878	-4.263520	2.086551
H	-0.709178	-2.721080	2.902698
H	-2.393906	-3.083360	2.400063
C	1.838487	-1.664475	0.377564
H	1.102591	-2.380480	0.776142
N	3.442583	-1.245139	2.854338
N	2.827367	-1.501984	1.985550
Si	3.205842	-2.500774	-0.739184
C	4.548398	-1.280153	-1.155547
H	4.166802	-0.386917	-1.668987
H	5.055956	-0.943423	-0.237655
H	5.300218	-1.766685	-1.798762
C	3.856983	-3.914885	0.301842
H	4.500580	-4.548184	-0.331454
H	4.468137	-3.577365	1.153289
H	3.040574	-4.548637	0.683996
C	2.263821	-3.197476	-2.197086
H	1.927391	-2.431675	-2.908329
H	2.934303	-3.887531	-2.736291
H	1.388340	-3.774256	-1.858814
Al	2.382471	2.381937	0.026857
Cl	0.415030	3.199223	-0.395826
Cl	3.061769	3.099172	1.952700
Cl	3.783192	2.921258	-1.523035

65

TS2a-Al'_3 Eopt -3290.913696

C	-1.029919	0.076528	0.016198
C	-0.253451	-0.580873	1.152942
C	0.983642	-0.204399	1.478478
C	1.632140	0.925938	0.704749
C	1.171838	0.946273	-0.736040
C	-0.049009	0.539102	-1.106427
C	1.877450	-0.874032	2.479727
C	3.157546	0.754320	0.601760
C	3.618642	-0.739346	0.566420
C	3.080527	-1.530528	1.776955
C	5.134087	-0.619543	0.570025
C	4.625233	0.590241	-1.390197
C	3.557250	1.451383	-0.709088
H	2.241292	-0.122829	3.197862
H	-0.763937	-1.400863	1.666064
H	3.892994	-1.677269	2.501618
H	5.182236	1.122879	-2.168858
O	-2.015623	-0.708219	-0.488888
C	-0.501915	0.364433	-2.520518
H	-0.557166	-0.710635	-2.751261
H	-1.508704	0.765894	-2.690962
H	0.195428	0.833847	-3.225058
C	2.334038	1.296876	-1.643517

H	2.123513	2.190289	-2.249251
H	1.323992	-1.629371	3.054514
H	2.776475	-2.530995	1.441940
O	5.903581	-1.064510	1.378285
O	5.595408	0.083794	-0.480248
C	3.284529	-1.428959	-0.804843
H	2.193039	-1.554059	-0.865427
C	3.716974	-0.503679	-1.968831
H	4.185081	-1.076710	-2.779644
H	3.854074	2.494785	-0.558340
H	3.636491	1.222442	1.473150
C	3.940480	-2.802209	-0.949148
H	3.599132	-3.278400	-1.880162
H	5.038800	-2.727078	-1.002336
H	3.689312	-3.477865	-0.120089
O	2.658830	0.234079	-2.535386
O	1.315550	2.220383	1.228319
C	1.480353	2.434395	2.610018
H	2.509936	2.230393	2.950579
H	1.265423	3.496288	2.790400
H	0.779979	1.827610	3.210762
C	-1.506831	1.458641	0.391447
H	-0.731619	2.046989	0.908076
N	-2.896924	0.930421	2.991697
N	-2.434077	1.139086	2.021644
Si	-2.767663	2.590591	-0.578930
C	-4.030492	1.627718	-1.548538
H	-3.588451	0.924555	-2.267398
H	-4.682841	1.055936	-0.871570
H	-4.659261	2.342565	-2.105597
C	-3.589258	3.639463	0.734533
H	-4.140298	4.454776	0.236910
H	-4.318492	3.064118	1.326514
H	-2.858860	4.099752	1.419081
C	-1.628240	3.644821	-1.627071
H	-1.127278	3.078928	-2.425404
H	-2.228346	4.438490	-2.103004
H	-0.857747	4.128683	-1.006042
Al	-3.247061	-1.888147	-0.154318
Cl	-2.433048	-3.565611	0.951203
Cl	-4.842410	-0.996278	1.028252
Cl	-4.011822	-2.541972	-2.060642

65

TS2a-Al_1 Eopt -3290.912363

C	1.362368	0.187208	0.174766
C	0.683156	0.086409	-1.199616
C	-0.556167	0.518723	-1.440770
C	-1.279566	1.367482	-0.431210
C	-0.817130	1.103492	0.979676
C	0.414676	0.677622	1.280790
C	-1.395228	0.142037	-2.616620
C	-2.790342	1.065982	-0.399816
C	-3.102837	-0.422162	-0.755068
C	-2.520679	-0.797360	-2.135412
C	-4.621249	-0.471021	-0.735098
C	-4.199852	0.238027	1.471931
C	-3.251575	1.360171	1.039295
H	-1.825503	1.059331	-3.049639
H	1.225117	-0.504388	-1.943595
H	-3.330352	-0.809990	-2.877346
H	-4.791750	0.490719	2.358600
O	1.855064	-1.034981	0.565621
C	0.892125	0.475208	2.687702
H	1.735190	1.151225	2.908028
H	0.101996	0.684162	3.418100
H	1.247337	-0.553571	2.835001

C	-2.004356	1.131268	1.928047
H	-1.906469	1.862836	2.743075
H	-0.800775	-0.352794	-3.396198
H	-2.114233	-1.816191	-2.089660
O	-5.356877	-0.768872	-1.637405
O	-5.131406	-0.122296	0.459340
C	-2.656552	-1.396334	0.396396
H	-1.557317	-1.417699	0.421254
C	-3.160648	-0.851437	1.759078
H	-3.541049	-1.665119	2.390096
H	-3.686156	2.358840	1.152438
H	-3.303861	1.698845	-1.137915
C	-3.159583	-2.823102	0.182063
H	-4.256797	-2.887103	0.268388
H	-2.873263	-3.224496	-0.799266
H	-2.728877	-3.485464	0.947168
O	-2.194715	-0.156995	2.516143
O	-1.004735	2.700570	-0.853793
C	-1.620572	3.764685	-0.174551
H	-1.138136	4.685590	-0.530815
H	-2.699882	3.836485	-0.394018
H	-1.482130	3.706095	0.919642
C	2.484101	1.091229	-0.240973
H	3.165208	0.545752	-0.914146
N	4.484230	0.544543	1.952435
N	3.734858	0.784946	1.191392
Si	2.595447	3.025958	-0.464546
C	4.435672	3.372223	-0.493011
H	4.958296	2.739636	-1.228413
H	4.588847	4.423184	-0.790611
H	4.913665	3.237422	0.489953
C	1.847785	3.362045	-2.138984
H	2.320099	2.738523	-2.914547

H	0.765475	3.173890	-2.127776
H	2.027223	4.419210	-2.397618
C	1.780581	3.868301	0.983458
H	2.340041	3.670250	1.911726
H	1.777912	4.958841	0.821468
H	0.743871	3.536166	1.122549
Al	2.210953	-2.595748	-0.138954
Cl	3.229173	-3.715271	1.393477
Cl	3.513229	-2.283750	-1.855056
Cl	0.409986	-3.614442	-0.770442

65

TS2a-B''_1 Eopt -1992.370425

C	-1.524503	0.403642	0.450582
C	-0.993266	0.108123	-0.933392
C	0.109130	-0.608820	-1.147547
C	0.808588	-1.249601	0.025364
C	0.627078	-0.458643	1.296494
C	-0.452340	0.290424	1.533941
C	0.845392	-0.711651	-2.449359
C	2.331814	-1.295071	-0.169866
C	2.879462	-0.072437	-0.973535
C	2.171299	0.068263	-2.339754
C	4.357800	-0.393874	-1.107230
C	4.146467	-0.374505	1.243034
C	2.920523	-1.293039	1.250206
H	1.057830	-1.766831	-2.680178
H	-1.517274	0.599751	-1.755802
H	2.855981	-0.254693	-3.136050
H	4.806034	-0.520205	2.105801
O	-2.216091	1.622218	0.517491
C	-0.639399	1.126885	2.761424
H	-0.464073	2.185897	2.520003
H	-1.665772	1.053489	3.152525

H	0.059404	0.837230	3.556250
C	1.900279	-0.513535	2.117340
H	1.750130	-0.947057	3.116653
H	0.244972	-0.308627	-3.276918
H	1.954395	1.129618	-2.520163
O	4.992865	-0.528859	-2.118976
O	4.960939	-0.539662	0.086810
C	2.836060	1.249300	-0.124551
H	1.783096	1.538702	0.001439
C	3.427397	0.980687	1.283242
H	4.072636	1.808337	1.605725
H	3.121570	-2.302505	1.624774
H	2.609330	-2.203190	-0.723412
C	3.574475	2.404592	-0.799359
H	4.659594	2.219776	-0.860517
H	3.207407	2.597408	-1.816890
H	3.429091	3.325908	-0.216029
O	2.461849	0.787442	2.290746
O	0.312374	-2.554952	0.327028
C	0.167255	-3.467255	-0.729923
H	1.113976	-3.661454	-1.265122
H	-0.169400	-4.415374	-0.287350
H	-0.589282	-3.138434	-1.465166
C	-2.843327	-0.272219	0.778386
H	-3.160496	-0.053554	1.807168
B	-1.755439	2.855035	-0.172472
F	-2.368631	3.923039	0.470352
F	-2.117276	2.844386	-1.531040
F	-0.358798	2.937681	-0.068683
N	-2.242370	-3.099497	1.582606
N	-2.407355	-2.064228	1.267912
Si	-4.294631	-0.631377	-0.486396
C	-4.860653	1.030019	-1.104491

H	-5.216860	1.656926	-0.272705
H	-4.047320	1.568699	-1.609989
H	-5.694149	0.886620	-1.812002
C	-3.671352	-1.759378	-1.839100
H	-2.936823	-1.267184	-2.492653
H	-3.221064	-2.679689	-1.434478
H	-4.531171	-2.059030	-2.461807
C	-5.599796	-1.486150	0.545300
H	-5.895093	-0.870698	1.410097
H	-6.498842	-1.648569	-0.072165
H	-5.271172	-2.471088	0.913524

65

TS2a-B''_2 Eopt -1992.370595

C	1.542248	0.334514	-0.474349
C	0.991249	0.253928	0.929626
C	-0.111166	-0.425698	1.231820
C	-0.802770	-1.234195	0.164721
C	-0.608137	-0.636708	-1.211582
C	0.479864	0.068965	-1.540706
C	-0.807703	-0.458003	2.553258
C	-2.335581	-1.254139	0.351830
C	-2.863009	0.033306	1.062075
C	-2.181904	0.230159	2.432841
C	-4.355294	-0.218187	1.199735
C	-4.126145	-0.379529	-1.137102
C	-2.941467	-1.346316	-1.062142
H	-0.940912	-1.512152	2.846755
H	1.528921	0.828486	1.686167
H	-2.851427	-0.141078	3.220490
H	-4.780558	-0.564235	-1.996373
O	2.247799	1.522021	-0.718742
C	0.672725	0.746513	-2.862125
H	1.712256	0.657675	-3.212672

H	0.005670	0.333188	-3.629361
H	0.458805	1.821106	-2.763713
C	-1.899512	-0.694569	-2.003138
H	-1.797460	-1.207826	-2.970883
H	-0.208628	0.029217	3.334831
H	-2.049678	1.304328	2.617227
O	-5.002517	-0.248032	2.212267
O	-4.957198	-0.425121	0.015420
C	-2.752277	1.285315	0.116406
H	-1.686710	1.516035	-0.023584
C	-3.349073	0.934763	-1.272818
H	-3.955989	1.763468	-1.660445
H	-3.199344	-2.371637	-1.348791
H	-2.612657	-2.115310	0.977236
C	-3.437938	2.525738	0.687072
H	-3.058489	2.794196	1.682422
H	-3.254291	3.383816	0.023578
H	-4.529877	2.394728	0.764556
O	-2.394108	0.621240	-2.260893
O	-0.225638	-2.533763	0.295820
C	-0.716479	-3.553928	-0.532431
H	-0.086073	-4.438203	-0.360941
H	-1.756245	-3.835366	-0.290821
H	-0.661257	-3.291706	-1.605584
C	2.851000	-0.407526	-0.667436
H	3.194903	-0.345211	-1.709347
B	1.799940	2.842362	-0.208786
F	0.402803	2.921567	-0.316054
F	2.414184	3.805509	-0.999944
F	2.171444	3.022277	1.135380
N	2.342573	-3.303438	-1.154993
N	2.403948	-2.237535	-0.912179
Si	4.263195	-0.599155	0.676419
C	4.897042	1.121510	0.994007
H	4.101725	1.775486	1.377368
H	5.713487	1.073365	1.733628
H	5.293200	1.571098	0.070485
C	3.557453	-1.437685	2.189736
H	2.919800	-2.296579	1.926365
H	4.391210	-1.813548	2.806065
H	2.964005	-0.747423	2.806539
C	5.546444	-1.677921	-0.154694
H	6.438524	-1.733275	0.491213
H	5.195391	-2.709070	-0.318210
H	5.861607	-1.257194	-1.122972

65

TS2a-B''_3 Eopt -1992.362243

C	1.441431	0.453211	0.097781
C	0.763692	-0.357163	1.187270
C	-0.392958	-0.995443	1.019680
C	-1.003555	-1.005673	-0.356039
C	-0.691796	0.258083	-1.116844
C	0.436636	0.957246	-0.948854
C	-1.242373	-1.608074	2.091636
C	-2.535689	-1.055693	-0.315592
C	-3.141390	-0.293764	0.905583
C	-2.552259	-0.805999	2.238991
C	-4.627258	-0.577100	0.766446
C	-4.207527	0.480244	-1.303064
C	-2.991699	-0.394113	-1.625219
H	-1.480259	-2.649086	1.822655
H	1.261811	-0.314470	2.159302
H	-3.303660	-1.422111	2.751725
H	-4.789445	0.763150	-2.187248
O	2.232335	1.393746	0.778393
C	0.744148	2.188204	-1.743207

H	0.729033	3.078455	-1.100474
H	1.749610	2.138187	-2.183601
H	0.012955	2.332514	-2.547207
C	-1.889822	0.639524	-1.966978
H	-1.654912	0.691170	-3.039826
H	-0.710993	-1.632743	3.053381
H	-2.352051	0.053500	2.892790
O	-5.349136	-1.123496	1.556915
O	-5.122067	-0.146169	-0.408389
C	-3.023298	1.264717	0.745710
H	-1.962720	1.536293	0.849068
C	-3.479290	1.677510	-0.677196
H	-4.086193	2.591950	-0.648154
H	-3.164117	-1.120222	-2.426825
H	-2.872789	-2.099438	-0.247522
C	-3.826965	2.023906	1.801060
H	-4.911708	1.874431	1.674753
H	-3.563295	1.724176	2.824822
H	-3.631966	3.102774	1.710023
O	-2.422369	1.906287	-1.579232
O	-0.495551	-2.059481	-1.176233
C	-0.507853	-3.363622	-0.655512
H	-0.119155	-4.023481	-1.443882
H	0.138554	-3.465640	0.233216
H	-1.523345	-3.710617	-0.393580
C	2.682099	-0.196082	-0.487652
H	3.165406	0.476390	-1.208984
B	3.008699	2.610337	0.432997
F	3.616371	2.508921	-0.835162
F	4.005069	2.711815	1.406850
F	2.190648	3.740971	0.481958
N	1.962441	-1.975999	-2.746530
N	2.160698	-1.343949	-1.875041

Si	3.902131	-1.377107	0.483487
C	5.337387	-1.583211	-0.695490
H	5.816504	-0.612993	-0.903353
H	6.096505	-2.247304	-0.250607
H	5.024996	-2.027852	-1.654236
C	4.397335	-0.515991	2.058077
H	4.759217	0.503203	1.863558
H	3.556090	-0.448736	2.763405
H	5.203317	-1.097627	2.536054
C	3.041433	-3.004773	0.810557
H	3.755237	-3.677397	1.315594
H	2.172203	-2.879216	1.473253
H	2.712813	-3.507925	-0.112218

65

TS2a-B'_1 Eopt -1992.375430

C	1.553176	0.534188	0.243613
C	0.614075	0.841241	1.403988
C	-0.488910	0.132002	1.628558
C	-0.826219	-1.028679	0.717097
C	-0.330343	-0.786928	-0.694240
C	0.763877	-0.060058	-0.960164
C	-1.497410	0.357127	2.709628
C	-2.353449	-1.209532	0.545534
C	-3.139428	0.129460	0.730523
C	-2.857955	0.756590	2.109606
C	-4.595566	-0.283684	0.586957
C	-3.763455	-0.987399	-1.496490
C	-2.572170	-1.732360	-0.887891
H	-1.602178	-0.585750	3.271715
H	0.916776	1.668348	2.050771
H	-3.663684	0.472982	2.799969
H	-4.157654	-1.469917	-2.397753
O	2.370283	1.579042	-0.098231

C	1.185409	0.356796	-2.333845
H	0.837193	-0.352469	-3.096213
H	0.740755	1.338631	-2.551224
H	2.272483	0.477057	-2.414572
C	-1.376513	-1.196651	-1.709278
H	-0.987104	-1.914816	-2.446604
H	-1.155053	1.124029	3.417630
H	-2.896771	1.849929	2.021254
O	-5.475920	-0.149623	1.393945
O	-4.857918	-0.857783	-0.599850
C	-2.880124	1.110059	-0.471930
H	-1.832396	1.441685	-0.425019
C	-3.074947	0.346336	-1.807827
H	-3.618569	0.959754	-2.537967
H	-2.673169	-2.822162	-0.933244
H	-2.717195	-1.916815	1.305179
C	-3.776929	2.346538	-0.436714
H	-4.840613	2.087092	-0.565291
H	-3.677340	2.910644	0.500485
H	-3.500882	3.023373	-1.258732
O	-1.875593	-0.067075	-2.422980
O	-0.213072	-2.157298	1.342903
C	-0.344811	-3.406040	0.709209
H	0.305210	-4.107553	1.249953
H	-1.377123	-3.793237	0.754876
H	-0.020905	-3.376805	-0.346794
C	2.386691	-0.680828	0.537660
H	1.817810	-1.459961	1.070318
B	1.840750	2.927702	-0.299489
F	1.944165	3.677416	0.888604
F	0.481789	2.886792	-0.686312
F	2.599788	3.539517	-1.301864
N	3.842297	0.407978	2.902910
N	3.304323	-0.068652	2.076881
Si	3.905637	-1.367606	-0.477543
C	4.844569	-2.427782	0.747427
H	5.361509	-1.834561	1.517882
H	4.183888	-3.153800	1.248124
H	5.614613	-2.998703	0.202075
C	3.139571	-2.464684	-1.784410
H	3.950724	-3.031597	-2.271542
H	2.447265	-3.191808	-1.331082
H	2.604585	-1.905715	-2.563771
C	4.955270	0.027338	-1.123938
H	4.392315	0.712078	-1.773160
H	5.361589	0.618785	-0.288578
H	5.804535	-0.385960	-1.692762

65

TS2a-B'_2 Eopt -1992.373283

C	1.400713	0.489850	-0.350343
C	0.538617	1.455626	0.457692
C	-0.588525	1.070734	1.056910
C	-1.020575	-0.376677	0.947858
C	-0.601984	-0.980666	-0.373324
C	0.507165	-0.601292	-1.020933
C	-1.571662	1.969175	1.747856
C	-2.551405	-0.524541	0.953655
C	-3.299024	0.686259	0.305951
C	-2.896229	2.022808	0.963761
C	-4.759650	0.334571	0.536230
C	-4.072847	-1.540341	-0.720271
C	-2.838361	-1.802042	0.147874
H	-1.763885	1.584753	2.761626
H	0.900013	2.485350	0.493043
H	-3.704550	2.354759	1.629545
H	-4.533755	-2.455054	-1.108910

O	2.148843	1.102758	-1.319491
C	0.878628	-1.052476	-2.397854
H	0.689875	-0.235895	-3.111110
H	1.943652	-1.300053	-2.485229
H	0.283693	-1.921895	-2.703961
C	-1.693861	-1.896777	-0.889106
H	-1.326026	-2.920726	-1.049323
H	-1.162709	2.982924	1.858795
H	-2.800236	2.788710	0.182778
O	-5.579910	0.967371	1.144782
O	-5.100138	-0.837994	-0.029249
C	-3.143401	0.709650	-1.256864
H	-2.100139	0.970029	-1.490127
C	-3.414136	-0.704563	-1.826529
H	-4.007147	-0.654981	-2.748970
H	-2.918609	-2.691503	0.781833
H	-2.905976	-0.609263	1.990490
C	-4.062288	1.733106	-1.923586
H	-3.845249	1.778445	-3.001068
H	-5.124658	1.460496	-1.814065
H	-3.930842	2.744610	-1.515496
O	-2.245958	-1.435957	-2.119341
O	-0.441567	-1.219046	1.951699
C	-0.505043	-0.791945	3.291584
H	-1.540436	-0.617813	3.632329
H	-0.077024	-1.600809	3.899035
H	0.085147	0.125659	3.462789
C	2.155723	-0.464497	0.545167
H	1.493274	-0.928665	1.291217
B	3.197943	2.076920	-1.065439
F	2.679837	3.305815	-0.605756
F	3.880389	2.274836	-2.264397
F	4.103291	1.600435	-0.076537

N	3.213820	1.225580	2.790299
N	2.891003	0.623966	1.934377
Si	3.641890	-1.629254	0.050391
C	4.598361	-1.087458	-1.452358
H	3.951591	-0.737770	-2.268013
H	5.283113	-0.267792	-1.195960
H	5.191872	-1.942557	-1.816583
C	4.740851	-1.729302	1.560072
H	5.486942	-2.524835	1.396851
H	5.289835	-0.789202	1.726060
H	4.178580	-1.981382	2.473244
C	2.748492	-3.258498	-0.210643
H	2.077425	-3.243277	-1.082292
H	3.499458	-4.048475	-0.379871
H	2.157664	-3.536715	0.676533

65

TS2a-B'_3 Eopt -1992.374368

C	1.558223	0.491502	0.279521
C	0.636191	0.646868	1.483510
C	-0.474772	-0.075092	1.623992
C	-0.821706	-1.100911	0.565305
C	-0.337665	-0.680351	-0.802949
C	0.748612	0.080267	-0.985565
C	-1.525997	0.122223	2.675411
C	-2.343987	-1.242776	0.383196
C	-3.121735	0.090164	0.637942
C	-2.812870	0.672475	2.033397
C	-4.574325	-0.332343	0.491984
C	-3.752514	-0.929633	-1.635033
C	-2.542014	-1.684516	-1.076201
H	-1.743496	-0.839777	3.164790
H	0.931998	1.409190	2.208562
H	-3.666626	0.485303	2.698913

H	-4.154459	-1.371998	-2.553264
O	2.388569	1.562691	0.082355
C	1.164093	0.663929	-2.298691
H	0.791178	0.068475	-3.142124
H	0.740212	1.675573	-2.379809
H	2.252162	0.773434	-2.379026
C	-1.356473	-1.065472	-1.854930
H	-0.927912	-1.734056	-2.615984
H	-1.174822	0.811772	3.455502
H	-2.712426	1.762561	1.949803
O	-5.449100	-0.247523	1.311561
O	-4.838057	-0.857774	-0.717903
C	-2.895927	1.130432	-0.518459
H	-1.855247	1.482396	-0.460677
C	-3.089055	0.431268	-1.887772
H	-3.649865	1.068602	-2.583609
H	-2.606308	-2.772663	-1.183185
H	-2.733453	-1.988821	1.090213
C	-3.821871	2.341445	-0.409535
H	-4.878384	2.064263	-0.558177
H	-3.739053	2.847044	0.562258
H	-3.561514	3.074901	-1.186911
O	-1.885303	0.073916	-2.526688
O	-0.222255	-2.378896	0.806507
C	-0.375224	-2.949684	2.083344
H	0.079051	-3.948834	2.037657
H	0.142122	-2.365174	2.864708
H	-1.433669	-3.068328	2.372575
C	2.374376	-0.768558	0.367632
H	1.796238	-1.622696	0.753375
B	1.880408	2.933570	0.083922
F	0.520737	2.971322	-0.300743
F	2.649156	3.676478	-0.816962
F	1.994967	3.496285	1.371299
N	3.823450	-0.118077	2.882218
N	3.297990	-0.445210	1.978936
Si	3.873482	-1.308203	-0.762579
C	4.926157	0.157135	-1.220567
H	4.363141	0.931439	-1.759587
H	5.347992	0.621503	-0.315431
H	5.764600	-0.178387	-1.853184
C	4.827537	-2.533818	0.283760
H	5.573337	-3.033442	-0.356899
H	5.375641	-2.054242	1.109763
H	4.172198	-3.314978	0.701486
C	3.077495	-2.209132	-2.195695
H	2.569274	-1.540661	-2.903430
H	3.869767	-2.744110	-2.745770
H	2.353592	-2.956881	-1.834709

65

TS2a-B'_4 Eopt -1992.373650

C	1.401875	0.555488	-0.239514
C	0.520467	1.368487	0.705331
C	-0.601486	0.877142	1.228405
C	-1.027462	-0.535105	0.887300
C	-0.592754	-0.911258	-0.515137
C	0.526410	-0.431198	-1.075740
C	-1.540745	1.577870	2.158487
C	-2.564837	-0.694841	0.859549
C	-3.309709	0.637963	0.526630
C	-2.933430	1.750763	1.523164
C	-4.779040	0.258742	0.625624
C	-4.076918	-1.248220	-1.039726
C	-2.872160	-1.738114	-0.232311
H	-1.624182	0.959659	3.067914
H	0.890070	2.367520	0.944802

H	-3.695017	1.792670	2.313345
H	-4.530376	-2.032793	-1.655663
O	2.141497	1.317704	-1.103737
C	0.907512	-0.642101	-2.506522
H	0.758146	0.292171	-3.067539
H	1.966232	-0.907637	-2.618982
H	0.295835	-1.426429	-2.968746
C	-1.709876	-1.629554	-1.244384
H	-1.387875	-2.598563	-1.653833
H	-1.139038	2.553278	2.464678
H	-2.969820	2.719927	1.009062
O	-5.608910	0.733790	1.352970
O	-5.117651	-0.726527	-0.224565
C	-3.105079	1.047961	-0.977057
H	-2.051773	1.336246	-1.110695
C	-3.376443	-0.178505	-1.885685
H	-3.941834	0.110127	-2.781274
H	-3.000749	-2.749262	0.168288
H	-2.903692	-1.022932	1.853053
C	-3.986674	2.222282	-1.400756
H	-5.057229	1.961003	-1.376094
H	-3.841204	3.108545	-0.768429
H	-3.743812	2.510637	-2.434206
O	-2.213284	-0.843732	-2.324101
O	-0.424832	-1.346929	1.897552
C	-0.646943	-2.735692	1.835611
H	0.017751	-3.196996	2.578781
H	-1.685621	-3.005221	2.092830
H	-0.403487	-3.154662	0.842686
C	2.170293	-0.498661	0.517263
H	1.515807	-1.046611	1.211026
B	3.180475	2.254988	-0.707144
F	2.652443	3.380448	-0.041515
F	3.838201	2.659612	-1.867368
F	4.111593	1.637255	0.173766
N	3.240629	0.897249	2.960676
N	2.905261	0.415270	2.036654
Si	3.664078	-1.572059	-0.128529
C	4.770178	-1.862769	1.350343
H	5.323754	-0.952448	1.628620
H	4.209998	-2.222679	2.228086
H	5.511960	-2.635038	1.086743
C	2.780212	-3.161913	-0.591591
H	3.533025	-3.903059	-0.908917
H	2.239913	-3.580120	0.272679
H	2.068276	-3.033971	-1.420565
C	4.605157	-0.830947	-1.554273
H	5.297100	-0.056802	-1.195780
H	5.190048	-1.628816	-2.041435
H	3.951114	-0.368691	-2.305572

65

TS2a-B_1 Eopt -1992.368199

C	-1.520379	0.644768	0.127751
C	-0.826941	0.527642	-1.237822
C	0.264364	-0.211668	-1.445843
C	0.770325	-1.157250	-0.391701
C	0.378437	-0.726284	0.998320
C	-0.716809	-0.005115	1.263908
C	1.162981	-0.109577	-2.634054
C	2.310499	-1.208367	-0.355997
C	2.954066	0.148343	-0.784508
C	2.478550	0.565533	-2.193406
C	4.445381	-0.140447	-0.742405
C	3.867387	-0.622907	1.489508
C	2.690706	-1.523372	1.102282
H	1.365664	-1.124162	-3.012713

H	-1.203397	1.196559	-2.012307
H	3.269134	0.340788	-2.921886
H	4.384476	-0.954029	2.396902
O	-1.720317	1.960954	0.507873
C	-1.114376	0.379131	2.658058
H	-0.467838	-0.094884	3.405939
H	-1.069192	1.468482	2.789618
H	-2.149330	0.066904	2.869719
C	1.526845	-0.972425	1.962294
H	1.265861	-1.617944	2.813346
H	0.697800	0.466855	-3.444724
H	2.326889	1.652800	-2.215546
O	5.233645	-0.056037	-1.645530
O	4.859465	-0.535532	0.474299
C	2.730273	1.257509	0.308641
H	1.661846	1.517090	0.319293
C	3.100795	0.686809	1.704580
H	3.655504	1.426932	2.295985
H	2.886353	-2.587543	1.271262
H	2.666074	-1.980939	-1.052826
C	3.529703	2.528847	0.027308
H	4.616061	2.355235	0.096932
H	3.316476	2.943892	-0.967361
H	3.270956	3.299244	0.768613
O	2.003677	0.269579	2.485396
O	0.200109	-2.411662	-0.755874
C	0.565510	-3.555750	-0.028020
H	-0.100852	-4.364449	-0.359178
H	1.605948	-3.866549	-0.226027
H	0.433352	-3.427205	1.061175
C	-2.812349	-0.001887	-0.281777
H	-3.337379	0.663041	-0.984797
B	-1.922682	3.087269	-0.398695

F	-2.424275	4.140818	0.364960
F	-2.860153	2.754348	-1.416288
F	-0.720873	3.472622	-1.015863
N	-4.719030	1.024400	1.816908
N	-3.995433	0.615142	1.104404
Si	-3.356178	-1.864870	-0.450663
C	-5.227644	-1.786309	-0.508538
H	-5.610925	-2.783299	-0.783567
H	-5.678731	-1.518126	0.459556
H	-5.581704	-1.073372	-1.270540
C	-2.694129	-2.430419	-2.099502
H	-3.119258	-3.423185	-2.324081
H	-2.997831	-1.741843	-2.903811
H	-1.598570	-2.506011	-2.076795
C	-2.775570	-2.819108	1.040898
H	-3.306490	-2.481063	1.945297
H	-2.993488	-3.891321	0.905880
H	-1.696924	-2.701537	1.205776

65

TS2a-B_2 Eopt -1992.367001

C	-1.471759	0.654740	0.010282
C	-0.768628	0.427362	-1.341705
C	0.363785	-0.267912	-1.468336
C	0.890875	-1.097993	-0.329254
C	0.475937	-0.549663	1.013188
C	-0.645829	0.153302	1.206874
C	1.278883	-0.228234	-2.648833
C	2.431220	-1.105092	-0.277590
C	3.048919	0.223797	-0.814668
C	2.577337	0.507379	-2.257438
C	4.545608	-0.029348	-0.738331
C	3.958996	-0.331777	1.522914
C	2.803947	-1.285752	1.205254

H	1.508827	-1.264343	-2.945979
H	-1.168020	1.028480	-2.159447
H	3.378604	0.232993	-2.956739
H	4.474634	-0.574202	2.458690
O	-1.632336	2.013540	0.200968
C	-1.083045	0.626188	2.564343
H	-1.747427	-0.119577	3.033236
H	-0.220314	0.767924	3.226874
H	-1.623067	1.578606	2.496215
C	1.619982	-0.687985	2.005295
H	1.360582	-1.264311	2.905020
H	0.808125	0.269694	-3.507070
H	2.410816	1.586080	-2.377883
O	5.339132	-0.006736	-1.640461
O	4.957483	-0.310442	0.510426
C	2.793493	1.416244	0.179104
H	1.718907	1.648748	0.164109
C	3.164920	0.974947	1.620694
H	3.698573	1.774181	2.151183
H	3.020446	-2.326597	1.467009
H	2.812065	-1.925040	-0.903401
C	3.567435	2.677108	-0.202885
H	4.656381	2.534983	-0.107749
H	3.357997	3.000677	-1.231704
H	3.283948	3.501916	0.467480
O	2.069976	0.601644	2.427141
O	0.361728	-2.398311	-0.575865
C	0.770656	-3.463855	0.242386
H	0.136610	-4.321957	-0.020341
H	1.822026	-3.750434	0.067583
H	0.633023	-3.251401	1.317522
C	-2.731946	-0.089127	-0.305038
H	-3.361763	0.501065	-0.988568

B	-2.603467	2.883872	-0.456324
F	-2.097658	4.180361	-0.432067
F	-3.840086	2.840885	0.223089
F	-2.846652	2.487424	-1.797211
N	-4.522568	0.541355	2.049062
N	-3.877028	0.350833	1.185820
Si	-3.141692	-1.995991	-0.430579
C	-5.011608	-2.036443	-0.550348
H	-5.377657	-1.389326	-1.363690
H	-5.321948	-3.068914	-0.783647
H	-5.518620	-1.746366	0.382594
C	-2.420333	-2.589956	-2.043403
H	-2.726346	-1.934843	-2.874361
H	-1.324786	-2.636592	-1.996339
H	-2.817339	-3.599207	-2.245088
C	-2.562636	-2.842820	1.126258
H	-3.193109	-2.543576	1.979038
H	-2.650029	-3.935920	1.015483
H	-1.518472	-2.599774	1.360985

65

TS2b-Al''_1 Eopt -3290.912090

C	1.268525	-0.268116	0.292683
C	0.395719	0.083646	1.478400
C	-0.849386	-0.370324	1.611740
C	-1.411283	-1.320030	0.578868
C	-0.804441	-1.082071	-0.784829
C	0.439571	-0.616295	-0.944982
C	-1.824142	-0.015352	2.687341
C	-2.926140	-1.111912	0.359630
C	-3.378660	0.354110	0.652081
C	-3.001823	0.778159	2.086826
C	-4.883138	0.310019	0.444872
C	-4.153874	-0.393491	-1.680720

C	-3.197234	-1.448315	-1.119085
H	-2.194501	-0.952515	3.134308
H	0.856370	0.726455	2.235167
H	-3.883398	0.674105	2.733694
H	-4.615974	-0.690086	-2.628936
O	2.220311	0.706384	-0.008077
C	0.979239	-0.185914	-2.276901
H	0.794836	0.891701	-2.410747
H	2.062418	-0.333173	-2.361462
H	0.481873	-0.712666	-3.101702
C	-1.871828	-1.149361	-1.859386
H	-1.638032	-1.873250	-2.654247
H	-1.345931	0.566300	3.487238
H	-2.738517	1.844085	2.087830
O	-5.740782	0.575740	1.243936
O	-5.222607	-0.082773	-0.795625
C	-2.850160	1.340745	-0.451207
H	-1.757408	1.411887	-0.350810
C	-3.157943	0.758384	-1.855059
H	-3.510325	1.541830	-2.538611
H	-3.550115	-2.475011	-1.264755
H	-3.478639	-1.773625	1.042511
C	-3.432090	2.747034	-0.321608
H	-4.520655	2.759985	-0.495999
H	-3.242329	3.189366	0.665906
H	-2.970568	3.406336	-1.071405
O	-2.062984	0.124962	-2.476943
O	-1.125913	-2.612077	1.106054
C	-1.500080	-3.726918	0.339953
H	-1.070783	-4.612262	0.830450
H	-2.594599	-3.864095	0.297663
H	-1.106136	-3.679124	-0.691825
C	2.412863	-1.193892	0.684913
H	2.844970	-0.853846	1.637020
N	1.393700	-3.607079	2.037558
N	1.687335	-2.697903	1.503480
Si	3.697883	-2.077626	-0.501409
C	4.725189	-0.738001	-1.289593
H	4.129230	-0.012839	-1.858992
H	5.277559	-0.182314	-0.515878
H	5.461285	-1.204630	-1.965227
C	4.754597	-3.109916	0.648802
H	5.598660	-3.524563	0.072673
H	5.176999	-2.501830	1.464776
H	4.210765	-3.959859	1.089597
C	2.767858	-3.184310	-1.685741
H	2.108151	-3.881867	-1.144755
H	2.162309	-2.633424	-2.418402
H	3.500719	-3.790763	-2.243887
Al	2.319171	2.464462	0.161623
Cl	3.877006	3.114088	-1.172101
Cl	2.807496	2.954333	2.209137
Cl	0.404480	3.298531	-0.393893

65

TS2b-Al''_2 Eopt -3290.910800

C	1.262546	-0.306597	0.263805
C	0.399607	-0.031215	1.477688
C	-0.852547	-0.476335	1.592634
C	-1.421254	-1.364684	0.508698
C	-0.811614	-1.070197	-0.838473
C	0.427771	-0.593391	-0.984854
C	-1.846998	-0.034335	2.623877
C	-2.922665	-1.112248	0.295411
C	-3.340214	0.372580	0.542296
C	-2.906094	0.857411	1.943942
C	-4.848956	0.322181	0.374759

C	-4.166380	-0.464212	-1.744287
C	-3.192645	-1.496451	-1.167109
H	-2.337575	-0.910015	3.075714
H	0.855115	0.600379	2.247453
H	-3.791238	0.935762	2.590131
H	-4.653940	-0.792675	-2.668882
O	2.197660	0.699754	0.021915
C	0.970344	-0.122087	-2.301841
H	2.055828	-0.250101	-2.384186
H	0.485440	-0.632363	-3.144243
H	0.773123	0.956519	-2.408844
C	-1.871083	-1.198095	-1.917055
H	-1.613720	-1.952080	-2.675112
H	-1.357834	0.523886	3.434260
H	-2.486486	1.868510	1.855927
O	-5.689411	0.615680	1.182306
O	-5.214120	-0.121396	-0.842307
C	-2.846908	1.320110	-0.608337
H	-1.753392	1.405095	-0.532992
C	-3.178987	0.686117	-1.981904
H	-3.548758	1.440857	-2.688261
H	-3.525735	-2.534215	-1.275920
H	-3.507659	-1.730088	0.989975
C	-3.442912	2.723442	-0.510179
H	-4.531903	2.720167	-0.682056
H	-3.257043	3.189329	0.467439
H	-2.991048	3.370737	-1.276254
O	-2.088965	0.038115	-2.595812
O	-1.177986	-2.752622	0.740515
C	-1.583346	-3.300835	1.968608
H	-1.394824	-4.382076	1.911712
H	-1.011473	-2.891632	2.821359
H	-2.659150	-3.158519	2.171360

C	2.420427	-1.243291	0.578909
H	2.841748	-0.980392	1.559851
N	1.438644	-3.810541	1.637813
N	1.706103	-2.824798	1.243745
Si	3.741600	-2.005631	-0.656446
C	4.725533	-0.584862	-1.352354
H	5.474761	-0.983429	-2.056506
H	4.107164	0.157989	-1.872714
H	5.261625	-0.065494	-0.542886
C	4.830787	-3.062287	0.440196
H	5.692249	-3.410244	-0.154140
H	5.227164	-2.484832	1.290784
H	4.320828	-3.956681	0.830192
C	2.860391	-3.070517	-1.913293
H	2.250980	-2.495747	-2.623963
H	3.618038	-3.625517	-2.491462
H	2.210182	-3.812926	-1.423299
Al	2.288671	2.446099	0.285764
Cl	0.363751	3.306177	-0.189489
Cl	2.809519	2.822099	2.349871
Cl	3.820433	3.181680	-1.033312

65

TS2b-Al'_1 Eopt -3290.921124

C	1.243043	-0.343673	-0.339169
C	0.587109	-0.285941	1.051350
C	-0.640055	-0.766971	1.269228
C	-1.396199	-1.415516	0.131128
C	-1.037844	-0.787799	-1.199156
C	0.175397	-0.282717	-1.446190
C	-1.397293	-0.712750	2.554160
C	-2.922457	-1.202380	0.247563
C	-3.291369	0.115147	1.003485
C	-2.669946	0.143403	2.412810

C	-4.810399	0.084079	1.055581
C	-4.477233	0.012409	-1.271811
C	-3.455470	-1.125118	-1.196908
H	-1.675041	-1.749605	2.807526
H	1.180479	0.171659	1.845284
H	-3.419193	-0.195518	3.140434
H	-5.101698	-0.027670	-2.171157
O	2.230769	0.583108	-0.516028
C	0.538413	0.459642	-2.695266
H	-0.118755	0.188092	-3.531735
H	0.428462	1.541531	-2.518863
H	1.585193	0.291509	-2.983715
C	-2.277583	-0.588480	-2.044726
H	-2.198989	-1.058413	-3.036414
H	-0.768648	-0.331607	3.369963
H	-2.426416	1.179835	2.679165
O	-5.507959	0.096944	2.034172
O	-5.369551	0.034865	-0.165418
C	-2.944207	1.382788	0.141118
H	-1.849515	1.454935	0.061044
C	-3.508206	1.200118	-1.291938
H	-3.963214	2.129398	-1.658617
H	-3.845317	-2.082681	-1.558343
H	-3.359747	-2.041754	0.807868
C	-3.462646	2.683136	0.752587
H	-4.564035	2.709198	0.793639
H	-3.081699	2.850322	1.769186
H	-3.135796	3.533598	0.136323
O	-2.552436	0.798428	-2.245636
O	-1.035995	-2.796119	0.220638
C	-1.568663	-3.672432	-0.742192
H	-1.370199	-3.330976	-1.774018
H	-1.072450	-4.641838	-0.597550
H	-2.654407	-3.823191	-0.616911
C	1.830122	-1.717926	-0.161794
H	1.071614	-2.516021	-0.141458
N	2.826434	-2.378380	-2.865570
N	2.439175	-2.164538	-1.863773
Si	3.475607	-2.067567	0.844124
C	3.803502	-3.885393	0.540032
H	2.938081	-4.506074	0.822735
H	4.057152	-4.098976	-0.510765
H	4.661015	-4.205096	1.155132
C	4.868249	-0.998065	0.218047
H	5.818977	-1.376530	0.629349
H	4.948236	-1.004573	-0.879825
H	4.735796	0.041333	0.550958
C	3.084297	-1.742094	2.640578
H	3.030926	-0.663615	2.851148
H	2.147278	-2.221200	2.962639
H	3.906274	-2.161951	3.244925
Al	2.416080	2.251690	-0.020247
Cl	3.223476	2.302411	1.995978
Cl	3.788298	3.185593	-1.392411
Cl	0.483923	3.239154	-0.044155

65

TS2b-Al'_2 Eopt -3290.920869

C	-1.245379	-0.288331	0.403617
C	-0.608469	-0.387654	-0.993791
C	0.615255	-0.893453	-1.176932
C	1.373061	-1.432096	0.016511
C	1.035614	-0.678302	1.280297
C	-0.162826	-0.128259	1.488003
C	1.397162	-0.834936	-2.451614
C	2.890034	-1.234175	-0.134038
C	3.269169	0.070799	-0.909033

C	2.583664	0.133910	-2.290481
C	4.781924	-0.030880	-1.004681
C	4.506894	-0.067416	1.338591
C	3.427857	-1.154314	1.303598
H	1.773376	-1.841985	-2.689895
H	-1.196438	0.024507	-1.816661
H	3.326443	-0.063787	-3.075160
H	5.156718	-0.126692	2.218611
O	-2.217942	0.667634	0.485442
C	-0.505234	0.719151	2.674704
H	-0.387071	1.781452	2.406870
H	-1.548547	0.589887	2.992699
H	0.162182	0.513419	3.521733
C	2.277448	-0.535865	2.136909
H	2.156126	-0.996196	3.128268
H	0.762966	-0.513614	-3.289117
H	2.209893	1.152879	-2.456544
O	5.454930	-0.059854	-1.999899
O	5.367585	-0.099219	0.204515
C	3.015074	1.363547	-0.053766
H	1.928235	1.498303	0.045607
C	3.598532	1.169624	1.367394
H	4.107386	2.077877	1.715687
H	3.761058	-2.126138	1.683341
H	3.325846	-2.078289	-0.686306
C	3.595639	2.620154	-0.701125
H	3.304744	3.503904	-0.114201
H	4.697273	2.593783	-0.734506
H	3.230546	2.770581	-1.726477
O	2.638474	0.828331	2.339315
O	1.063574	-2.801327	0.294350
C	1.170334	-3.728827	-0.758673
H	0.979551	-4.720155	-0.325709
H	0.424914	-3.545833	-1.553185
H	2.175838	-3.741920	-1.213369
C	-1.852271	-1.664815	0.393303
H	-1.103541	-2.466180	0.493960
N	-2.910752	-1.995514	3.130596
N	-2.488346	-1.887409	2.126106
Si	-3.495407	-2.115724	-0.577579
C	-3.107735	-1.989852	-2.399773
H	-2.182641	-2.516840	-2.677788
H	-3.942337	-2.457163	-2.949702
H	-3.038791	-0.941257	-2.724918
C	-3.829136	-3.887988	-0.077660
H	-4.704981	-4.262848	-0.632704
H	-2.974539	-4.540871	-0.317821
H	-4.052526	-3.990380	0.996378
C	-4.880281	-0.970548	-0.082398
H	-4.981349	-0.863590	1.008399
H	-4.725340	0.027561	-0.516833
H	-5.829585	-1.375061	-0.471683
Al	-2.364104	2.280408	-0.181407
Cl	-3.228220	2.137626	-2.169789
Cl	-3.661171	3.407948	1.115421
Cl	-0.397881	3.189048	-0.321811

65

TS2b-Al'_3 Eopt -3290.913349

C	-0.963284	0.040476	-0.054454
C	-0.168612	0.593702	1.143037
C	1.080311	1.053288	1.015728
C	1.724545	1.073062	-0.352374
C	1.228436	-0.068963	-1.213675
C	-0.010880	-0.562765	-1.105604
C	1.959786	1.555598	2.111492
C	3.252429	0.863218	-0.285443

C	3.694483	0.023546	0.955180
C	3.215905	0.675397	2.266575
C	5.211225	0.007366	0.850176
C	4.644989	-0.980250	-1.209487
C	3.637291	0.112325	-1.575025
H	2.257802	2.581264	1.836589
H	-0.695358	0.574832	2.099725
H	4.032855	1.279381	2.682971
H	5.176069	-1.384962	-2.078071
O	-1.908723	-0.834558	0.360837
C	-0.510206	-1.742456	-1.875832
H	-0.630278	-2.608939	-1.209264
H	-1.502787	-1.554925	-2.313104
H	0.179375	-2.019102	-2.682573
C	2.379431	-0.693568	-1.975531
H	2.208433	-0.729534	-3.061538
H	1.415736	1.615486	3.063500
H	3.005482	-0.109451	3.004307
O	6.003217	0.414380	1.656937
O	5.643836	-0.534860	-0.300807
C	3.269123	-1.482907	0.809755
H	2.172530	-1.537150	0.877504
C	3.677749	-1.998770	-0.595298
H	4.091462	-3.013916	-0.536393
H	3.990910	0.781156	-2.367068
H	3.745254	1.843700	-0.213747
C	3.867542	-2.378087	1.894175
H	3.477761	-3.400810	1.783640
H	4.965664	-2.435438	1.818437
H	3.617077	-2.037994	2.908233
O	2.626739	-2.029656	-1.532366
O	1.384685	2.361133	-0.873068
C	1.848780	2.691025	-2.159881

H	2.941950	2.837850	-2.186054
H	1.569201	1.932396	-2.912543
H	1.371363	3.641876	-2.432941
C	-1.473107	1.402879	-0.459965
H	-0.690250	1.990171	-0.965289
N	-2.859592	0.931669	-3.031196
N	-2.382420	1.104481	-2.061099
Si	-2.659485	2.585687	0.555923
C	-3.587631	1.716379	1.912885
H	-4.050431	2.489109	2.550088
H	-4.391815	1.081093	1.516224
H	-2.937129	1.100395	2.549676
C	-1.451758	3.838444	1.248936
H	-0.814841	3.398047	2.031102
H	-0.798981	4.256468	0.466933
H	-2.019011	4.668989	1.701569
C	-3.810611	3.378908	-0.684762
H	-4.385512	4.166622	-0.169803
H	-3.268789	3.853753	-1.518345
H	-4.533029	2.654223	-1.091527
Al	-3.416506	-1.682647	0.372788
Cl	-3.232363	-3.652008	-0.505223
Cl	-4.075613	-1.918263	2.417031
Cl	-4.895136	-0.559813	-0.770118

65

TS2b-Al'_4 Eopt -3290.913638

C	0.966493	0.134168	-0.047683
C	0.140460	0.615428	-1.256359
C	-1.108388	1.072933	-1.123308
C	-1.722453	1.152525	0.257135
C	-1.201708	0.047515	1.153623
C	0.034334	-0.451513	1.033655
C	-2.018333	1.508246	-2.224154

C	-3.250481	0.929623	0.233722
C	-3.716464	0.028022	-0.954811
C	-3.269186	0.612593	-2.307944
C	-5.230549	0.015176	-0.814044
C	-4.614196	-0.872981	1.276120
C	-3.601724	0.237987	1.565128
H	-2.320993	2.544172	-1.996669
H	0.639583	0.537157	-2.222663
H	-4.099182	1.187301	-2.739812
H	-5.123809	-1.236269	2.175318
O	1.944481	-0.711164	-0.453381
C	0.542578	-1.616767	1.821581
H	0.598247	-2.509927	1.179578
H	1.559021	-1.444584	2.205683
H	-0.116112	-1.850332	2.666723
C	-2.331406	-0.545384	1.971088
H	-2.131756	-0.527710	3.052716
H	-1.494464	1.524184	-3.189196
H	-3.066734	-0.209208	-3.006740
O	-6.041873	0.383837	-1.620019
O	-5.635172	-0.473322	0.370761
C	-3.283829	-1.468977	-0.747193
H	-2.188887	-1.524235	-0.837610
C	-3.658654	-1.918234	0.689112
H	-4.070290	-2.935919	0.689331
H	-3.939007	0.941131	2.334042
H	-3.750520	1.903254	0.126653
C	-3.904142	-2.415880	-1.773932
H	-4.999914	-2.472828	-1.669277
H	-3.679134	-2.123262	-2.808462
H	-3.507633	-3.431062	-1.624695
O	-2.585377	-1.901604	1.600918
O	-1.381690	2.464296	0.713858

C	-1.785960	2.833814	2.010301
H	-2.878084	2.970083	2.085207
H	-1.460847	2.103949	2.772989
H	-1.307275	3.798359	2.227572
C	1.467929	1.510711	0.316002
H	0.659544	2.157875	0.689101
N	2.594479	1.206082	3.033691
N	2.201790	1.331558	2.019183
Si	2.908776	2.488162	-0.599768
C	2.794280	2.155043	-2.430348
H	3.614109	2.708040	-2.919394
H	2.937740	1.084558	-2.642064
H	1.845404	2.496053	-2.869878
C	2.510342	4.261519	-0.160555
H	1.509234	4.543910	-0.524658
H	2.536183	4.418513	0.930396
H	3.244961	4.945472	-0.615917
C	4.584931	1.987943	0.049736
H	4.685224	2.061612	1.142686
H	4.865615	0.974152	-0.269702
H	5.315985	2.685784	-0.393682
Al	3.305181	-1.766636	-0.297369
Cl	4.319928	-1.422506	1.594222
Cl	4.670391	-1.330857	-1.923783
Cl	2.679886	-3.830703	-0.445349

65

TS2b-Al_1 Eopt -3290.912316

C	1.574430	-0.018388	-0.169361
C	0.934302	-0.190929	1.191625
C	-0.203817	-0.842390	1.410709
C	-0.847958	-1.649829	0.314960
C	-0.487625	-1.135158	-1.058098
C	0.651115	-0.487748	-1.342730

C	-1.026837	-0.781306	2.657640
C	-2.388001	-1.551554	0.361078
C	-2.874761	-0.189083	0.947390
C	-2.295496	0.044282	2.360085
C	-4.387747	-0.330890	0.965026
C	-3.971560	-0.650386	-1.330464
C	-2.872114	-1.690345	-1.093493
H	-1.300495	-1.804425	2.961322
H	1.419390	0.373539	1.992547
H	-3.069573	-0.180422	3.106343
H	-4.561860	-0.844818	-2.232802
O	1.932143	1.293250	-0.363040
C	0.956983	0.075367	-2.700597
H	0.449258	-0.501469	-3.484844
H	0.604588	1.115006	-2.760364
H	2.033448	0.078598	-2.919896
C	-1.705301	-1.170242	-1.967552
H	-1.540354	-1.755495	-2.884549
H	-0.469646	-0.323996	3.486558
H	-2.043509	1.106721	2.473215
O	-5.119125	-0.258656	1.915780
O	-4.898700	-0.567453	-0.256257
C	-2.595327	0.990950	-0.053705
H	-1.508650	1.143415	-0.112461
C	-3.090118	0.595794	-1.470592
H	-3.593257	1.438782	-1.961136
H	-3.179066	-2.710876	-1.345301
H	-2.781736	-2.349353	1.006916
C	-3.236522	2.305593	0.385262
H	-4.337419	2.256058	0.355753
H	-2.936535	2.596881	1.400796
H	-2.918095	3.109597	-0.293610
O	-2.079529	0.150646	-2.348787
O	-0.386952	-2.978909	0.535755
C	-0.937545	-4.019797	-0.230540
H	-1.991299	-4.221904	0.027068
H	-0.868281	-3.831730	-1.317047
H	-0.352026	-4.921011	-0.001830
C	2.761362	-0.887806	-0.413491
H	3.344683	-0.510558	-1.268159
N	4.639495	0.571336	1.532247
N	4.014844	0.026125	0.817177
Si	3.161142	-2.754920	-0.030238
C	2.385169	-3.657357	-1.469807
H	2.570873	-4.738584	-1.358569
H	1.302128	-3.492166	-1.524171
H	2.838658	-3.331725	-2.419403
C	5.028174	-2.831625	-0.160732
H	5.395066	-2.384244	-1.098213
H	5.532315	-2.338415	0.685240
H	5.337008	-3.890485	-0.153426
C	2.591341	-3.249891	1.668742
H	1.494737	-3.259547	1.730930
H	2.972037	-4.261415	1.888730
H	2.994065	-2.566533	2.433496
Al	1.335443	2.918688	-0.095146
Cl	0.434884	3.172701	1.860988
Cl	3.080811	4.182286	-0.261224
Cl	-0.126820	3.484405	-1.592388

65

TS2b-Al_2 Eopt -3290.911513

C	1.576453	0.002842	-0.165671
C	0.939816	-0.206520	1.195154
C	-0.210469	-0.847056	1.402690
C	-0.844648	-1.626711	0.280446
C	-0.485056	-1.077912	-1.076150

C	0.642744	-0.411118	-1.348443
C	-1.079434	-0.689525	2.616143
C	-2.377059	-1.536809	0.317579
C	-2.889945	-0.156903	0.836513
C	-2.291350	0.180814	2.221365
C	-4.395193	-0.357980	0.875995
C	-3.977865	-0.778678	-1.409126
C	-2.832478	-1.758930	-1.131333
H	-1.427030	-1.668891	2.978216
H	1.415040	0.363871	1.998086
H	-3.075352	0.092440	2.985982
H	-4.568657	-1.037696	-2.294707
O	1.957592	1.315514	-0.300118
C	0.946808	0.178440	-2.696394
H	0.609026	1.224243	-2.729664
H	2.021078	0.174927	-2.925764
H	0.425731	-0.372321	-3.490371
C	-1.678729	-1.212909	-2.007333
H	-1.455453	-1.832432	-2.888108
H	-0.532576	-0.206648	3.437894
H	-1.959666	1.227584	2.219386
O	-5.124421	-0.269651	1.827005
O	-4.900267	-0.680971	-0.329155
C	-2.671251	0.982923	-0.220805
H	-1.593003	1.184793	-0.292900
C	-3.152279	0.498916	-1.612701
H	-3.694252	1.292741	-2.142525
H	-3.077959	-2.805217	-1.342960
H	-2.781552	-2.302461	0.992147
C	-3.371793	2.283601	0.166220
H	-4.469227	2.180397	0.150809
H	-3.077046	2.631828	1.165770
H	-3.099538	3.072580	-0.549357
O	-2.118379	0.061117	-2.466543
O	-0.417200	-2.987665	0.240039
C	-0.690758	-3.826787	1.334472
H	-0.255090	-3.453745	2.276468
H	-1.771078	-3.992155	1.486979
H	-0.227728	-4.796140	1.103589
C	2.739437	-0.875484	-0.485461
H	3.308602	-0.450113	-1.326150
N	4.689749	0.373777	1.521365
N	4.042732	-0.090475	0.769939
Si	3.120012	-2.771554	-0.248088
C	2.126046	-3.642101	-1.564313
H	2.457228	-4.693185	-1.611613
H	1.054362	-3.611348	-1.325616
H	2.296288	-3.189671	-2.554184
C	4.948071	-2.881855	-0.637028
H	5.570321	-2.331629	0.086228
H	5.257967	-3.939709	-0.599972
H	5.170107	-2.504307	-1.647974
C	2.788857	-3.256605	1.518680
H	3.524437	-2.782027	2.187963
H	1.784409	-2.959638	1.843661
H	2.885261	-4.348745	1.632256
Al	1.362957	2.934822	0.006365
Cl	0.494570	3.148569	1.983384
Cl	-0.125694	3.534411	-1.452046
Cl	3.102961	4.204664	-0.161046

65

TS2b-Al_3 Eopt -3290.908630

C	-1.135158	0.205622	0.179539
C	-0.314876	0.451495	1.430799
C	0.955903	0.843075	1.436209
C	1.634271	1.259318	0.156721

C	1.012784	0.603966	-1.053469
C	-0.267875	0.216281	-1.123645
C	1.879874	0.829879	2.612537
C	3.110551	0.805747	0.116064
C	3.356724	-0.490849	0.948958
C	2.919856	-0.292715	2.416794
C	4.855211	-0.715483	0.825610
C	4.245603	-0.758402	-1.447727
C	3.439621	0.540867	-1.364625
H	2.386520	1.806022	2.678408
H	-0.822322	0.164802	2.356863
H	3.808412	-0.084580	3.028091
H	4.749861	-0.892467	-2.411153
O	-1.725137	-1.020299	0.313298
C	-0.839007	-0.496397	-2.315568
H	-0.323485	-0.190964	-3.235495
H	-0.711426	-1.583300	-2.205234
H	-1.912985	-0.305721	-2.447637
C	2.093469	0.159743	-2.024566
H	1.961620	0.569441	-3.036928
H	1.333165	0.675517	3.552762
H	2.493084	-1.231264	2.794985
O	5.661430	-0.774842	1.714570
O	5.254555	-0.851365	-0.451026
C	2.706799	-1.742868	0.252776
H	1.613598	-1.637886	0.311663
C	3.101680	-1.758524	-1.248501
H	3.345785	-2.775837	-1.580635
H	3.933932	1.388847	-1.850229
H	3.741681	1.597300	0.545015
C	3.101213	-3.061113	0.916995
H	4.176956	-3.272531	0.802506
H	2.867316	-3.074852	1.990368
H	2.551059	-3.889876	0.447332
O	2.111676	-1.264182	-2.123828
O	1.511344	2.676896	0.129035
C	2.183662	3.386827	-0.879921
H	1.956937	3.003471	-1.890902
H	1.833128	4.426425	-0.820656
H	3.278077	3.385066	-0.738549
C	-2.115650	1.285009	-0.147002
H	-2.886221	0.918012	-0.844812
N	-3.989676	0.872253	2.240782
N	-3.365479	1.020651	1.353375
Si	-2.023033	3.230767	-0.176116
C	-3.824609	3.742979	-0.206131
H	-4.394269	3.202561	-0.978957
H	-4.323931	3.597599	0.764681
H	-3.877828	4.818884	-0.443282
C	-1.167163	3.903710	1.330904
H	-1.630253	3.512228	2.250927
H	-0.098335	3.651443	1.326751
H	-1.276931	5.001084	1.341471
C	-1.239966	3.595370	-1.831796
H	-0.231498	3.173667	-1.919831
H	-1.861761	3.191207	-2.646503
H	-1.177639	4.688345	-1.964424
Al	-3.024444	-2.123013	-0.033989
Cl	-2.249968	-3.875445	-1.020239
Cl	-3.959558	-2.645403	1.842758
Cl	-4.498967	-1.121823	-1.291363

65

TS2b-Al_4 Eopt -3290.912293

C	1.579535	0.005859	-0.169354
C	0.944968	-0.177895	1.192517
C	-0.181871	-0.847378	1.414160

C	-0.814472	-1.666776	0.320935
C	-0.460947	-1.152078	-1.053900
C	0.667066	-0.487399	-1.341396
C	-1.005636	-0.793527	2.660874
C	-2.355785	-1.588392	0.366458
C	-2.859460	-0.229696	0.947510
C	-2.283735	0.016103	2.359572
C	-4.370609	-0.389469	0.965223
C	-3.949913	-0.712787	-1.328785
C	-2.838067	-1.738581	-1.087580
H	-1.266948	-1.818874	2.967792
H	1.421216	0.396509	1.991704
H	-3.055467	-0.214679	3.106386
H	-4.537508	-0.917789	-2.230548
O	1.904534	1.325986	-0.365138
C	0.964692	0.071978	-2.702782
H	0.601572	1.107529	-2.768320
H	2.040608	0.085197	-2.924756
H	0.462115	-0.514754	-3.482974
C	-1.677598	-1.207847	-1.963608
H	-1.505113	-1.794994	-2.877983
H	-0.454455	-0.326624	3.488487
H	-2.043498	1.081819	2.467787
O	-5.103141	-0.322224	1.915460
O	-4.878343	-0.636843	-0.255275
C	-2.593998	0.949941	-0.058156
H	-1.509186	1.116135	-0.114902
C	-3.083496	0.543262	-1.473660
H	-3.596633	1.378324	-1.967400
H	-3.133002	-2.763539	-1.335562
H	-2.739393	-2.388679	1.015314
C	-3.252185	2.257761	0.375322
H	-4.352283	2.195563	0.339477
H	-2.959808	2.553935	1.391414
H	-2.939247	3.064262	-0.303277
O	-2.067963	0.106768	-2.350495
O	-0.335986	-2.988901	0.546231
C	-0.870389	-4.038413	-0.219669
H	-1.921840	-4.254768	0.035999
H	-0.801656	-3.850493	-1.306220
H	-0.272868	-4.931132	0.011276
C	2.785375	-0.836445	-0.411952
H	3.363284	-0.445256	-1.263870
N	4.632724	0.652960	1.540775
N	4.017361	0.099275	0.824180
Si	3.219180	-2.696653	-0.032030
C	2.455392	-3.612857	-1.469300
H	2.671059	-4.689130	-1.363866
H	1.367808	-3.476686	-1.512810
H	2.890949	-3.271763	-2.421845
C	5.086871	-2.739282	-0.165616
H	5.415578	-3.792098	-0.153845
H	5.443227	-2.289513	-1.105994
H	5.583324	-2.232425	0.676868
C	2.658687	-3.202899	1.666680
H	1.562332	-3.230129	1.728872
H	3.055109	-4.208712	1.885007
H	3.050645	-2.514513	2.432488
Al	1.260518	2.934328	-0.099668
Cl	-0.122434	3.501568	-1.670210
Cl	0.238026	3.138442	1.802484
Cl	2.986460	4.234763	-0.137128

65

TS2b-Al_5 Eopt -3290.911437

C	-1.607802	0.086333	0.105935
C	-0.965510	-0.178200	-1.242960

C	0.163471	-0.863513	-1.424342
C	0.751452	-1.645832	-0.279860
C	0.403564	-1.051618	1.060098
C	-0.703251	-0.342345	1.308782
C	1.053908	-0.765980	-2.628282
C	2.285893	-1.634114	-0.300375
C	2.870180	-0.290332	-0.835847
C	2.304298	0.049222	-2.234073
C	4.364569	-0.561102	-0.853134
C	3.901662	-0.924848	1.432860
C	2.712356	-1.852868	1.158032
H	1.355472	-1.767838	-2.970313
H	-1.408760	0.397054	-2.060677
H	3.091395	-0.094758	-2.986944
H	4.469546	-1.196960	2.329412
O	-1.934479	1.417999	0.194658
C	-1.039581	0.207246	2.667008
H	-0.141678	0.644625	3.121846
H	-1.803191	0.992263	2.605530
H	-1.407348	-0.592190	3.330513
C	1.576282	-1.235258	2.011377
H	1.307943	-1.828805	2.897156
H	0.540691	-0.270185	-3.463978
H	2.026654	1.111111	-2.256462
O	5.108757	-0.520815	-1.795862
O	4.839541	-0.889643	0.362670
C	2.689598	0.874496	0.201281
H	1.620080	1.119146	0.260341
C	3.137164	0.394203	1.605757
H	3.711521	1.171018	2.126344
H	2.903886	-2.905986	1.390402
H	2.660522	-2.430380	-0.956646
C	3.443174	2.141000	-0.198117
H	4.535680	1.996833	-0.166742
H	3.174142	2.483020	-1.206802
H	3.191423	2.951194	0.501176
O	2.079133	0.018683	2.460228
O	0.257667	-2.983443	-0.212483
C	0.501998	-3.860064	-1.284089
H	0.095924	-3.487793	-2.239552
H	1.574521	-4.081543	-1.419485
H	-0.010730	-4.800006	-1.037012
C	-2.808618	-0.729715	0.448229
H	-3.349948	-0.262416	1.285540
N	-4.706441	0.516476	-1.585159
N	-4.075297	0.064382	-0.813117
Si	-3.261287	-2.615892	0.254584
C	-2.308028	-3.504259	1.588572
H	-2.485936	-3.047394	2.574827
H	-2.665330	-4.546989	1.632010
H	-1.232232	-3.502293	1.367866
C	-5.092550	-2.651403	0.644209
H	-5.299513	-2.244083	1.646780
H	-5.697544	-2.096646	-0.089969
H	-5.438737	-3.698518	0.631615
C	-2.944106	-3.144972	-1.502488
H	-1.932463	-2.878364	-1.832107
H	-3.066851	-4.236335	-1.595500
H	-3.667304	-2.665542	-2.181715
Al	-1.163861	2.981171	-0.010085
Cl	0.238702	3.392341	1.585218
Cl	-0.120885	3.147653	-1.906055
Cl	-2.784707	4.408906	0.047442

65

TS2b-Al_6 Eopt -3290.907543

C	-1.132962	0.190214	0.175028

C	-0.316144	0.477450	1.422993
C	0.962634	0.853628	1.423643
C	1.633096	1.230882	0.126494
C	1.012731	0.538096	-1.059712
C	-0.259073	0.128315	-1.118357
C	1.898355	0.738293	2.591561
C	3.101597	0.777283	0.089492
C	3.348301	-0.551981	0.868645
C	2.860276	-0.440697	2.331487
C	4.854764	-0.725683	0.765069
C	4.283716	-0.680441	-1.523764
C	3.431909	0.587406	-1.398304
H	2.476473	1.666456	2.714944
H	-0.821736	0.189526	2.350083
H	3.731173	-0.358220	2.996070
H	4.810776	-0.758510	-2.481102
O	-1.749557	-1.013628	0.371228
C	-0.824899	-0.628304	-2.286032
H	-1.895425	-0.435718	-2.440778
H	-0.296515	-0.371174	-3.213292
H	-0.711334	-1.710787	-2.125596
C	2.095208	0.181315	-2.063104
H	1.927586	0.642266	-3.047259
H	1.348143	0.566359	3.527093
H	2.340232	-1.368369	2.606199
O	5.648831	-0.800390	1.663767
O	5.277802	-0.786184	-0.510706
C	2.755368	-1.792086	0.107508
H	1.658293	-1.727931	0.154985
C	3.171396	-1.728348	-1.385562
H	3.455850	-2.720295	-1.759624
H	3.885473	1.476894	-1.848689
H	3.741090	1.540859	0.551269
C	3.189132	-3.121176	0.724155
H	2.672599	-3.949980	0.217816
H	4.272559	-3.290300	0.612195
H	2.946832	-3.183518	1.794117
O	2.172304	-1.230364	-2.246481
O	1.526173	2.621039	-0.174386
C	2.106508	3.563572	0.692188
H	3.204922	3.475134	0.748426
H	1.866786	4.552199	0.277014
H	1.696154	3.511943	1.714534
C	-2.083736	1.263897	-0.249379
H	-2.836649	0.856502	-0.942566
N	-4.016547	1.078304	2.103688
N	-3.379275	1.140280	1.215303
Si	-1.967070	3.202151	-0.425729
C	-3.745624	3.697894	-0.737866
H	-4.404191	3.498183	0.121787
H	-3.782837	4.782298	-0.936117
H	-4.158884	3.182769	-1.619834
C	-1.342419	3.935666	1.168218
H	-2.108394	3.844743	1.955207
H	-0.428939	3.438398	1.516498
H	-1.126758	5.007826	1.031458
C	-0.947818	3.498529	-1.959298
H	-1.035970	4.563255	-2.233712
H	0.108262	3.257425	-1.779790
H	-1.323956	2.900567	-2.804627
Al	-3.075156	-2.100662	0.080314
Cl	-2.349854	-3.921422	-0.814801
Cl	-4.018918	-2.498043	1.983259
Cl	-4.527884	-1.134449	-1.229136

65

TS2b-B''_1 Eopt -1992.367994

C	-1.452556	0.483590	-0.109845
C	-0.556824	0.853821	-1.274545
C	0.582415	0.210900	-1.526886
C	0.997857	-0.956404	-0.662806
C	0.477490	-0.811864	0.747189
C	-0.663879	-0.171971	1.025908
C	1.567641	0.529474	-2.605364
C	2.530808	-1.045306	-0.496323
C	3.229484	0.344114	-0.630402
C	2.898797	1.002006	-1.985719
C	4.707153	0.012968	-0.502626
C	3.920039	-0.819122	1.554119
C	2.779294	-1.614013	0.913737
H	1.736372	-0.388132	-3.192540
H	-0.911920	1.651658	-1.928393
H	3.721279	0.804443	-2.686466
H	4.346148	-1.309422	2.436516
O	-2.220346	1.529501	0.428239
C	-1.096596	0.132609	2.429250
H	-0.804613	1.161601	2.689007
H	-2.184694	0.079919	2.553612
H	-0.624257	-0.546231	3.150738
C	1.547544	-1.195311	1.750096
H	1.217841	-1.964138	2.464598
H	1.177914	1.293390	-3.291940
H	2.857801	2.091381	-1.855371
O	5.576391	0.222968	-1.305596
O	5.004105	-0.585680	0.664225
C	2.915373	1.259554	0.608572
H	1.851112	1.534260	0.567339
C	3.146624	0.454181	1.914632
H	3.647893	1.070296	2.672610
H	2.952813	-2.695607	0.912242
H	2.936551	-1.698122	-1.282910
C	3.745727	2.542080	0.624605
H	4.820569	2.333339	0.752494
H	3.623554	3.132105	-0.293975
H	3.431530	3.175003	1.467747
O	1.972830	-0.056640	2.503552
O	0.475285	-2.094166	-1.342824
C	0.657847	-3.352384	-0.748788
H	0.068395	-4.071994	-1.334862
H	1.710850	-3.683561	-0.771070
H	0.297251	-3.380290	0.295757
C	-2.736313	-0.194059	-0.573004
H	-3.151984	0.380757	-1.413179
B	-2.491114	2.828422	-0.227704
F	-1.322323	3.590613	-0.289670
F	-3.454128	3.461210	0.551101
F	-2.988602	2.628288	-1.535033
N	-2.157349	-2.431920	-2.371934
N	-2.300051	-1.601443	-1.673385
Si	-4.111242	-1.048681	0.524829
C	-4.885276	0.286637	1.569762
H	-4.173936	0.765978	2.255180
H	-5.309364	1.070925	0.924123
H	-5.706122	-0.153932	2.159790
C	-5.356800	-1.693562	-0.716363
H	-6.245998	-2.049713	-0.169708
H	-5.687237	-0.899843	-1.405481
H	-4.977882	-2.539717	-1.310809
C	-3.349078	-2.471805	1.467181
H	-2.861941	-3.183165	0.780706
H	-2.612343	-2.156563	2.219104
H	-4.152094	-3.017318	1.990661

65

TS2b-B''_2 Eopt -1992.366258

C	1.447787	0.443376	0.143151
C	0.568602	0.674721	1.356492
C	-0.579874	0.022608	1.545953
C	-1.009452	-1.035382	0.556205
C	-0.488236	-0.749825	-0.827644
C	0.648225	-0.083990	-1.049242
C	-1.605603	0.352597	2.589275
C	-2.536103	-1.067514	0.378750
C	-3.205059	0.333405	0.546977
C	-2.841663	0.972848	1.905610
C	-4.685169	0.011903	0.426825
C	-3.916051	-0.783323	-1.657350
C	-2.767913	-1.592761	-1.046588
H	-1.904566	-0.559955	3.126785
H	0.916902	1.420961	2.072235
H	-3.707393	0.903917	2.578571
H	-4.355142	-1.250652	-2.545760
O	2.197843	1.555637	-0.274416
C	1.080525	0.347437	-2.419382
H	2.169178	0.331017	-2.544992
H	0.623503	-0.274900	-3.199310
H	0.766580	1.388699	-2.591103
C	-1.534581	-1.128007	-1.858254
H	-1.173079	-1.875309	-2.579384
H	-1.202452	1.053782	3.333212
H	-2.644783	2.042668	1.754683
O	-5.548371	0.208471	1.239693
O	-4.990709	-0.566941	-0.748738
C	-2.905906	1.275271	-0.673842
H	-1.841949	1.551283	-0.635578
C	-3.147309	0.501102	-1.995352
H	-3.655861	1.132575	-2.735646

H	-2.921031	-2.676942	-1.077032
H	-2.984539	-1.729843	1.131495
C	-3.741473	2.554560	-0.646499
H	-4.815050	2.345585	-0.783639
H	-3.624196	3.110809	0.293822
H	-3.429004	3.218841	-1.465848
O	-1.974628	0.009997	-2.600850
O	-0.518885	-2.336671	0.884936
C	-0.814645	-2.855384	2.156184
H	-0.438206	-3.887853	2.173504
H	-0.321168	-2.290509	2.967928
H	-1.897872	-2.889644	2.367391
C	2.738524	-0.283420	0.493447
H	3.158321	0.168237	1.404460
B	2.485810	2.760842	0.534305
F	1.320937	3.509745	0.720609
F	3.430702	3.490206	-0.179249
F	3.012059	2.394737	1.793751
N	2.251481	-2.794518	1.903207
N	2.316819	-1.841914	1.367480
Si	4.122568	-0.967107	-0.712357
C	4.872968	0.505264	-1.574040
H	5.691176	0.156969	-2.226094
H	4.148907	1.065100	-2.180467
H	5.296500	1.199882	-0.832585
C	5.383328	-1.739783	0.437671
H	6.272627	-2.015943	-0.153397
H	5.709114	-1.028880	1.213891
H	5.022035	-2.656002	0.930203
C	3.385040	-2.273934	-1.826678
H	2.685804	-1.871345	-2.572510
H	4.204195	-2.775353	-2.368860
H	2.857311	-3.043646	-1.240318

65

TS2b-B''_3 Eopt -1992.365805

C	-1.512621	0.346710	-0.383853
C	-0.596642	0.253069	-1.585646
C	0.533504	-0.454145	-1.580826
C	0.905455	-1.255708	-0.354423
C	0.341863	-0.652408	0.906661
C	-0.779213	0.073555	0.928187
C	1.606791	-0.379746	-2.626043
C	2.425489	-1.251987	-0.116085
C	3.113985	0.075749	-0.566941
C	2.807616	0.395703	-2.047380
C	4.585837	-0.207644	-0.320615
C	3.731978	-0.519787	1.856163
C	2.598258	-1.443200	1.398751
H	1.926945	-1.391887	-2.916473
H	-0.894815	0.848332	-2.452230
H	3.702610	0.195946	-2.652652
H	4.133229	-0.777125	2.842784
O	-2.241585	1.540833	-0.316773
C	-1.239856	0.824697	2.141657
H	-2.330838	0.914716	2.194183
H	-0.874551	0.361285	3.067165
H	-0.844261	1.851188	2.099646
C	1.341153	-0.803131	2.037471
H	0.940188	-1.371907	2.888946
H	1.243881	0.123462	-3.533114
H	2.593160	1.468580	-2.142831
O	5.482221	-0.190183	-1.121334
O	4.841751	-0.513580	0.964317
C	2.774206	1.266555	0.400397
H	1.715397	1.526487	0.259296
C	2.964567	0.809129	1.869546
H	3.453848	1.590093	2.466324
H	2.737476	-2.493057	1.678470
H	2.893906	-2.068152	-0.683003
C	3.615693	2.510659	0.118669
H	4.682604	2.344910	0.341306
H	3.536369	2.841037	-0.926236
H	3.271534	3.340145	0.754064
O	1.769293	0.466400	2.530687
O	0.411672	-2.595663	-0.388242
C	0.711751	-3.383528	-1.511015
H	1.796068	-3.470311	-1.700628
H	0.326806	-4.392029	-1.304294
H	0.227176	-3.007922	-2.430786
C	-2.837463	-0.358911	-0.630559
H	-3.153551	-0.197517	-1.671146
B	-1.718173	2.856352	-0.760862
F	-2.483128	3.821288	-0.114727
F	-1.839243	2.998048	-2.150301
F	-0.367819	2.959245	-0.391301
N	-2.380459	-3.236058	-1.139739
N	-2.446814	-2.158032	-0.961084
Si	-4.340220	-0.604171	0.607355
C	-4.984987	1.092142	1.030641
H	-4.237909	1.720022	1.533826
H	-5.300233	1.615905	0.115036
H	-5.864701	0.985168	1.686877
C	-5.601511	-1.547332	-0.405074
H	-5.844032	-1.019028	-1.341005
H	-5.277602	-2.570191	-0.653690
H	-6.532570	-1.631746	0.179973
C	-3.787733	-1.636413	2.063959
H	-3.069606	-1.123950	2.718534
H	-4.676877	-1.883393	2.668068

H -3.338629 -2.587660 1.736575

65

TS2b-B'_1 Eopt -1992.378183

C	-1.562293	0.440990	0.291307
C	-0.931725	0.058598	-1.054183
C	0.167169	-0.697372	-1.121799
C	0.799317	-1.210973	0.151746
C	0.587237	-0.254322	1.305169
C	-0.497634	0.522388	1.399187
C	0.902787	-1.076769	-2.363518
C	2.335981	-1.319887	0.032954
C	2.935236	-0.288008	-0.976693
C	2.309299	-0.448376	-2.375670
C	4.420260	-0.613986	-0.981760
C	4.114947	-0.122690	1.297923
C	2.895449	-1.037019	1.441116
H	0.986763	-2.176190	-2.371851
H	-1.434120	0.445411	-1.942337
H	2.976823	-1.059241	-2.997825
H	4.734275	-0.086409	2.200999
O	-2.367335	1.550798	0.243449
C	-0.693505	1.561147	2.459015
H	-0.076834	1.355544	3.343569
H	-0.402583	2.544253	2.059918
H	-1.746212	1.643787	2.761838
C	1.853145	-0.119773	2.124525
H	1.701919	-0.344178	3.190990
H	0.346185	-0.781042	-3.262752
H	2.248160	0.535192	-2.858739
O	5.092816	-0.941810	-1.922331
O	4.978526	-0.505986	0.235701
C	2.847432	1.178201	-0.413642
H	1.788139	1.472545	-0.381703
C	3.388349	1.202500	1.040573
H	4.016867	2.086146	1.211890
H	3.102334	-1.951375	2.007686
H	2.598639	-2.326735	-0.323673
C	3.601859	2.191394	-1.273385
H	4.687854	2.001871	-1.275403
H	3.257688	2.196803	-2.316539
H	3.444918	3.202892	-0.870540
O	2.390921	1.200656	2.034833
O	0.181201	-2.485226	0.353309
C	0.575965	-3.232350	1.477300
H	0.487802	-2.656011	2.415801
H	-0.100915	-4.095553	1.537542
H	1.607582	-3.614026	1.387090
C	-2.403551	-0.802929	0.379997
H	-1.830474	-1.686733	0.701103
B	-1.949944	2.756265	-0.466151
F	-2.327892	2.685438	-1.829066
F	-0.547521	2.918953	-0.409771
F	-2.584661	3.847050	0.130386
N	-3.823014	-0.327496	2.918408
N	-3.277248	-0.568936	2.000238
Si	-3.908870	-1.227074	-0.799190
C	-5.005186	-2.373334	0.196359
H	-4.432203	-3.198149	0.650084
H	-5.559512	-1.850252	0.991240
H	-5.750671	-2.820982	-0.481756
C	-4.784048	0.329573	-1.317428
H	-5.586089	0.079825	-2.031759
H	-5.239882	0.831934	-0.450324
H	-4.090146	1.039638	-1.789064
C	-3.133590	-2.160338	-2.223626
H	-2.585645	-1.494334	-2.905724

H -2.443774 -2.941870 -1.868261

H -3.936916 -2.650450 -2.799034

65

TS2b-B'_2 Eopt -1992.377606

C	-1.549908	0.505485	0.229408
C	-0.940696	-0.122065	-1.034055
C	0.161565	-0.877156	-0.981475
C	0.798002	-1.161058	0.361635
C	0.602182	-0.019432	1.328664
C	-0.471358	0.773129	1.295036
C	0.940882	-1.357626	-2.165648
C	2.325786	-1.295214	0.255000
C	2.948506	-0.381742	-0.850865
C	2.299045	-0.632419	-2.228296
C	4.416916	-0.771258	-0.815706
C	4.125256	-0.039107	1.407427
C	2.863783	-0.878328	1.633454
H	1.109322	-2.441513	-2.070399
H	-1.435915	0.130543	-1.973319
H	2.991709	-1.209569	-2.855748
H	4.747247	0.062148	2.303635
O	-2.343061	1.597932	-0.014666
C	-0.658492	1.967238	2.178946
H	-0.326138	2.867936	1.641140
H	-1.713407	2.130227	2.436547
H	-0.067573	1.883376	3.100537
C	1.845689	0.155335	2.175148
H	1.642286	0.050349	3.250862
H	0.385224	-1.190981	-3.098503
H	2.145334	0.331093	-2.731640
O	5.078419	-1.222296	-1.712100
O	4.972575	-0.567280	0.392012
C	2.932656	1.135333	-0.441565
H	1.889175	1.481509	-0.456488
C	3.461246	1.285924	1.007255
H	4.128282	2.153350	1.097033
H	3.010027	-1.731773	2.304305
H	2.594258	-2.332210	0.010819
C	3.746958	2.008197	-1.395976
H	3.624189	3.066601	-1.122572
H	4.823696	1.777320	-1.345417
H	3.427303	1.898193	-2.441385
O	2.451164	1.431765	1.976781
O	0.232563	-2.309387	1.004194
C	0.177479	-3.511667	0.274779
H	-0.216450	-4.276649	0.957736
H	-0.497152	-3.440407	-0.596165
H	1.170345	-3.844118	-0.075297
C	-2.404337	-0.685991	0.564157
H	-1.830087	-1.502213	1.030355
B	-1.913798	2.657091	-0.922482
F	-0.514233	2.836532	-0.866208
F	-2.566609	3.832708	-0.548638
F	-2.260158	2.338175	-2.258867
N	-3.719325	0.177246	3.058626
N	-3.218046	-0.173569	2.150515
Si	-3.951462	-1.266267	-0.490774
C	-3.261410	-2.555025	-1.657878
H	-2.763028	-3.368424	-1.107726
H	-4.101751	-2.996183	-2.220125
H	-2.552589	-2.132462	-2.384527
C	-5.122876	-2.076093	0.724454
H	-5.896735	-2.616326	0.153950
H	-4.611286	-2.810783	1.367160
H	-5.640347	-1.344942	1.365016
C	-4.728760	0.179583	-1.363765

H	-5.157151	0.889701	-0.640075
H	-3.998415	0.728571	-1.974499
H	-5.542291	-0.184682	-2.013092

65

TS2b-B'_3 Eopt -1992.371541

C	1.390300	-0.483390	-0.303490
C	0.657176	0.579442	-1.146750
C	-0.474089	1.162011	-0.738303
C	-1.042415	0.805618	0.616331
C	-0.731380	-0.629737	0.983158
C	0.384346	-1.251092	0.580245
C	-1.292941	2.147149	-1.504147
C	-2.583837	0.880851	0.643734
C	-3.229824	0.606892	-0.751981
C	-2.693711	1.584480	-1.815417
C	-4.716067	0.802397	-0.497984
C	-4.256903	-0.917456	1.041881
C	-3.045340	-0.198959	1.641204
H	-1.386862	3.045809	-0.871726
H	1.119695	0.807316	-2.108813
H	-3.402996	2.415417	-1.925569
H	-4.816708	-1.505811	1.777328
O	2.093714	-1.290078	-1.154887
C	0.677181	-2.690123	0.857809
H	0.655234	-3.265296	-0.078982
H	1.687174	-2.823222	1.269696
H	-0.053700	-3.122520	1.551392
C	-1.944767	-1.285338	1.612359
H	-1.735282	-1.722582	2.599660
H	-0.788070	2.449171	-2.431435
H	-2.659599	1.076600	-2.787865
O	-5.451420	1.587790	-1.033164
O	-5.192536	-0.028345	0.444809
C	-3.091105	-0.904340	-1.162987
H	-2.028603	-1.108880	-1.360945
C	-3.527571	-1.802378	0.024414
H	-4.127730	-2.652576	-0.325061
H	-3.230534	0.200446	2.644071
H	-2.885380	1.891264	0.956355
C	-3.894922	-1.257139	-2.413964
H	-3.699001	-2.302612	-2.694584
H	-4.979899	-1.160667	-2.245165
H	-3.630962	-0.629390	-3.275929
O	-2.461371	-2.326561	0.780409
O	-0.449201	1.759214	1.502728
C	-0.767229	1.669105	2.870344
H	-1.813238	1.952867	3.077014
H	-0.581551	0.658834	3.276674
H	-0.112523	2.378763	3.394237
C	2.158177	0.557244	0.475511
H	1.490064	1.087586	1.169587
B	3.392468	-1.928525	-1.001781
F	4.228388	-1.195634	-0.112737
F	3.995844	-1.975273	-2.263078
F	3.287649	-3.236130	-0.501786
N	3.380558	-0.839534	2.767375
N	2.966788	-0.329461	1.891387
Si	3.545193	1.751847	-0.219764
C	4.132208	1.315780	-1.932131
H	4.747395	2.155519	-2.297538
H	4.743001	0.404435	-1.934221
H	3.302828	1.175486	-2.640276
C	2.644539	3.397768	-0.237055
H	1.826118	3.406193	-0.973620
H	2.221004	3.644099	0.749519
H	3.353149	4.197347	-0.511065

C	4.936220	1.787880	1.027928
H	5.667208	2.550754	0.712234
H	4.586613	2.061913	2.036211
H	5.457996	0.820641	1.078066

65

TS2b-B'_4 Eopt -1992.371687

C	-1.382996	0.446817	-0.338368
C	-0.632218	-0.553498	-1.239964
C	0.501496	-1.148269	-0.855629
C	1.046087	-0.876051	0.528500
C	0.720330	0.532733	0.980487
C	-0.391558	1.172078	0.596991
C	1.345683	-2.066215	-1.676296
C	2.587343	-0.942394	0.577315
C	3.256423	-0.568780	-0.784780
C	2.743941	-1.469083	-1.925141
C	4.738715	-0.776125	-0.517272
C	4.242348	0.832159	1.128431
C	3.023315	0.070020	1.653701
H	1.441336	-3.006691	-1.108089
H	-1.075132	-0.710271	-2.223978
H	3.463300	-2.282594	-2.088114
H	4.785424	1.370569	1.912976
O	-2.080190	1.292097	-1.155747
C	-0.706638	2.591043	0.948895
H	-0.723539	3.207719	0.038365
H	-1.707319	2.674253	1.395773
H	0.034229	3.010220	1.639552
C	1.917276	1.151123	1.675689
H	1.684290	1.518320	2.685973
H	0.858210	-2.307343	-2.630394
H	2.716258	-0.889606	-2.856904
O	5.487958	-1.519014	-1.092221
O	5.193000	-0.011463	0.490357
C	3.119514	0.966444	-1.093865
H	2.060186	1.179765	-1.299805
C	3.528317	1.782543	0.160103
H	4.130623	2.657212	-0.118072
H	3.192157	-0.394640	2.630991
H	2.890696	-1.969892	0.826003
C	3.947757	1.405763	-2.301005
H	5.029377	1.303445	-2.114971
H	3.706556	0.835461	-3.208354
H	3.751359	2.466669	-2.515589
O	2.444513	2.249126	0.928617
O	0.446582	-1.889867	1.341387
C	0.710165	-1.865756	2.723498
H	1.755245	-2.132270	2.955122
H	0.480735	-0.884345	3.175540
H	0.056781	-2.619998	3.182818
C	-2.153028	-0.636835	0.378946
H	-1.468717	-1.264518	0.967674
B	-3.315451	2.010914	-0.893696
F	-3.709201	1.888813	0.461839
F	-4.348993	1.488646	-1.699499
F	-3.143016	3.363463	-1.206227
N	-3.082216	0.433573	2.971310
N	-2.814651	0.074228	1.972161
Si	-3.692677	-1.654850	-0.299969
C	-3.635527	-1.664670	-2.163678
H	-4.567355	-2.126387	-2.531072
H	-3.587394	-0.636393	-2.549244
H	-2.790652	-2.250177	-2.555457
C	-3.379682	-3.351080	0.426566
H	-2.402897	-3.745710	0.102617
H	-3.390087	-3.322723	1.528514

H	-4.158674	-4.059504	0.100288
C	-5.287557	-0.921501	0.330800
H	-5.343559	-0.885779	1.429554
H	-5.436990	0.088620	-0.073539
H	-6.112697	-1.562825	-0.022116

65

TS2b-B_1 Eopt -1992.369044

C	-1.629817	0.803115	0.178999
C	-0.994448	0.573416	-1.177667
C	0.006000	-0.275732	-1.397868
C	0.483514	-1.202214	-0.308542
C	0.234272	-0.629121	1.066520
C	-0.762543	0.223254	1.347181
C	0.824087	-0.372355	-2.646468
C	2.011858	-1.416602	-0.360076
C	2.766618	-0.184657	-0.947181
C	2.242311	0.161334	-2.357937
C	4.219260	-0.627906	-0.967921
C	3.750140	-0.864806	1.327858
C	2.459272	-1.654188	1.093590
H	0.874503	-1.428090	-2.958369
H	-1.361081	1.229536	-1.970095
H	2.944686	-0.230476	-3.106216
H	4.290620	-1.177784	2.228145
O	-1.926288	2.133768	0.399971
C	-0.942903	0.867951	2.689334
H	-0.559630	0.223211	3.491499
H	-0.384015	1.814292	2.712603
H	-1.994748	1.102955	2.900825
C	1.425561	-0.904764	1.970688
H	1.147524	-1.444906	2.888088
H	0.370265	0.196088	-3.469648
H	2.230167	1.252548	-2.478802
O	4.952475	-0.693574	-1.918104
O	4.672100	-0.971315	0.251051
C	2.729175	1.022931	0.056811
H	1.695034	1.384409	0.121951
C	3.145081	0.535334	1.469223
H	3.814571	1.256033	1.956116
H	2.550264	-2.716427	1.344112
H	2.233293	-2.277079	-1.007398
C	3.605311	2.194388	-0.381457
H	4.677191	1.935708	-0.376573
H	3.347988	2.553389	-1.387415
H	3.462866	3.035690	0.312654
O	2.067548	0.308978	2.353880
O	-0.228444	-2.415671	-0.531261
C	0.139527	-3.558210	0.198118
H	1.123978	-3.953423	-0.105064
H	0.149572	-3.382502	1.288650
H	-0.615948	-4.325963	-0.018660
C	-2.897224	0.047689	0.413047
H	-3.419551	0.468923	1.286730
B	-1.020777	3.247389	0.115384
F	-1.537160	4.361662	0.782474
F	-0.960201	3.517275	-1.265410
F	0.285651	2.981609	0.575689
N	-4.629435	1.690180	-1.503295
N	-4.043565	1.112104	-0.781123
Si	-3.563436	-1.736694	0.004674
C	-2.965940	-2.761832	1.447953
H	-3.217493	-3.820942	1.273547
H	-1.884795	-2.679757	1.609960
H	-3.480973	-2.439875	2.367142
C	-5.422909	-1.531703	0.143255
H	-5.715989	-1.006120	1.066102

H	-5.860453	-1.003102	-0.717988
H	-5.877848	-2.536178	0.175354
C	-3.112363	-2.281108	-1.715324
H	-2.032999	-2.451323	-1.815749
H	-3.648222	-3.218141	-1.941809
H	-3.432558	-1.527612	-2.453011

65

TS2b-B_2 Eopt -1992.368631

C	-1.620740	0.851026	0.102553
C	-1.012089	0.510106	-1.245489
C	-0.008694	-0.349401	-1.422939
C	0.449505	-1.213117	-0.276911
C	0.200100	-0.568483	1.062496
C	-0.769560	0.322747	1.302285
C	0.887485	-0.397977	-2.626000
C	1.967768	-1.438504	-0.298060
C	2.757524	-0.205900	-0.837081
C	2.243863	0.223920	-2.231431
C	4.188728	-0.711522	-0.858010
C	3.678463	-0.995203	1.430885
C	2.355129	-1.719451	1.160091
H	1.037493	-1.436336	-2.958756
H	-1.357486	1.146840	-2.062956
H	2.999669	-0.024318	-2.989418
H	4.198131	-1.354657	2.325974
O	-1.863155	2.206562	0.210625
C	-0.947248	1.009402	2.625788
H	-0.350909	1.932337	2.635178
H	-1.990517	1.293047	2.819031
H	-0.603168	0.369602	3.449489
C	1.333464	-0.924707	2.011061
H	0.978470	-1.465835	2.900384
H	0.456930	0.162070	-3.467708
H	2.122253	1.315556	-2.243292
O	4.930577	-0.786863	-1.800641
O	4.607220	-1.111382	0.357734
C	2.769530	0.972767	0.198325
H	1.755449	1.388692	0.254914
C	3.136459	0.429776	1.601592
H	3.830063	1.103760	2.120797
H	2.374256	-2.789185	1.395024
H	2.212142	-2.284752	-0.952859
C	3.715576	2.101670	-0.205792
H	4.772238	1.788318	-0.174232
H	3.504400	2.477109	-1.216830
H	3.598037	2.945463	0.490043
O	2.028275	0.236517	2.454238
O	-0.243033	-2.460355	-0.213503
C	-0.107861	-3.380103	-1.267884
H	-0.442968	-2.972320	-2.236266
H	0.924399	-3.753229	-1.380948
H	-0.750600	-4.234909	-1.015695
C	-2.898402	0.157117	0.439472
H	-3.389946	0.686845	1.269688
B	-0.880443	3.253167	-0.076076
F	0.344731	3.010502	0.582291
F	-1.432826	4.449853	0.388331
F	-0.621677	3.362130	-1.456277
N	-4.635188	1.561638	-1.629833
N	-4.055891	1.079195	-0.835752
Si	-3.588674	-1.658586	0.269411
C	-2.779626	-2.642493	1.631778
H	-3.299197	-3.612043	1.714672
H	-1.718173	-2.818293	1.413987
H	-2.877101	-2.124889	2.599420
C	-5.410187	-1.443275	0.654761

H	-5.940575	-0.835875	-0.095501
H	-5.886313	-2.438218	0.666588
H	-5.565197	-0.989209	1.646547
C	-3.364069	-2.248902	-1.482783
H	-4.053539	-1.708869	-2.151655
H	-2.340312	-2.089947	-1.842208
H	-3.598552	-3.323382	-1.554651

65

TS2b-B_3 Eopt -1992.364386

C	1.460079	0.636833	-0.219207
C	0.814196	0.583116	1.152322
C	-0.283618	-0.110137	1.441447
C	-0.866653	-1.079466	0.446487
C	-0.514703	-0.711023	-0.973796
C	0.593068	-0.047081	-1.327482
C	-1.121743	0.039485	2.671914
C	-2.410702	-1.070205	0.469038
C	-2.989464	0.312398	0.901684
C	-2.451528	0.722419	2.290032
C	-4.492127	0.082642	0.916879
C	-4.017292	-0.455622	-1.325328
C	-2.863745	-1.396445	-0.966221
H	-1.317631	-0.959731	3.092717
H	1.251467	1.282182	1.869738
H	-3.213936	0.497554	3.048131
H	-4.579046	-0.778787	-2.208771
O	1.552577	1.951932	-0.631112
C	0.895167	0.344406	-2.746109
H	0.467599	-0.384390	-3.448023
H	0.460370	1.328688	-2.968682
H	1.974404	0.414816	-2.937540
C	-1.716175	-0.908141	-1.882335
H	-1.511755	-1.573359	-2.734381
H	-0.605070	0.628595	3.441755
H	-2.301052	1.809958	2.311161
O	-5.243115	0.213846	1.845669
O	-4.966864	-0.313397	-0.277340
C	-2.763131	1.395476	-0.216262
H	-1.685490	1.606996	-0.274108
C	-3.208252	0.819310	-1.587428
H	-3.753285	1.571938	-2.171767
H	-3.107040	-2.455103	-1.106157
H	-2.765119	-1.816881	1.193962
C	-3.498111	2.703644	0.070978
H	-4.592372	2.572995	0.046150
H	-3.230928	3.127125	1.048866
H	-3.238317	3.449506	-0.694662
O	-2.155053	0.347947	-2.399483
O	-0.338379	-2.345827	0.824998
C	-0.814377	-3.499077	0.179850
H	-0.772048	-3.422301	-0.921075
H	-0.158295	-4.324910	0.488810
H	-1.847228	-3.752162	0.475226
C	2.716881	-0.159499	-0.355733
H	3.376033	0.283923	-1.118209
B	2.578878	2.909303	-0.250344
F	2.230294	4.136759	-0.806617
F	3.846430	2.505766	-0.745296
F	2.684823	3.028753	1.153894
N	4.495532	0.813048	1.966096
N	3.887593	0.545601	1.095765
Si	3.156647	-2.040419	-0.053653
C	5.015279	-2.093926	-0.282098
H	5.323883	-1.625362	-1.230288
H	5.567996	-1.614620	0.540549
H	5.330497	-3.150503	-0.315181

C	2.683670	-2.580511	1.661979
H	3.197644	-1.971314	2.422518
H	1.597971	-2.515616	1.814095
H	3.000808	-3.627422	1.802939
C	2.350872	-2.949035	-1.472594
H	1.256603	-2.890204	-1.447462
H	2.701211	-2.548397	-2.437144
H	2.650009	-4.009292	-1.421126

65

TS2b-B_4 Eopt -1992.369134

C	1.611875	0.843498	-0.097957
C	1.005175	0.523541	1.251673
C	0.024089	-0.352227	1.446347
C	-0.431759	-1.253125	0.330494
C	-0.174436	-0.651954	-1.029559
C	0.812812	0.216029	-1.291831
C	-0.811276	-0.479872	2.680153
C	-1.956944	-1.483393	0.360181
C	-2.720396	-0.255742	0.948038
C	-2.213743	0.081815	2.368464
C	-4.172757	-0.699173	0.947911
C	-3.675939	-0.919890	-1.343310
C	-2.384839	-1.708267	-1.101388
H	-0.880619	-1.541695	2.965859
H	1.357060	1.161875	2.065604
H	-2.937400	-0.292204	3.105508
H	-4.205541	-1.226717	-2.252118
O	1.759720	2.207659	-0.257707
C	1.071173	0.774619	-2.661836
H	0.123324	1.091728	-3.114504
H	1.734461	1.647020	-2.623184
H	1.526314	0.009487	-3.311418
C	-1.342688	-0.946225	-1.959642
H	-1.041073	-1.479729	-2.873184
H	-0.369810	0.065424	3.525542
H	-2.176112	1.172754	2.485282
O	-4.918020	-0.771091	1.888335
O	-4.609791	-1.036981	-0.278221
C	-2.667650	0.959020	-0.048266
H	-1.632500	1.317554	-0.102943
C	-3.072718	0.482588	-1.468246
H	-3.739283	1.206568	-1.954015
H	-2.469799	-2.768143	-1.363431
H	-2.181880	-2.351696	0.995738
C	-3.540242	2.133034	0.389039
H	-4.613721	1.881299	0.375532
H	-3.286156	2.486092	1.397782
H	-3.385338	2.975719	-0.300611
O	-1.992439	0.260304	-2.350225
O	0.295420	-2.462858	0.531353
C	-0.026936	-3.584463	-0.249872
H	-1.009086	-4.013475	0.012824
H	-0.013429	-3.368879	-1.333406
H	0.741447	-4.342438	-0.043420
C	2.940064	0.221331	-0.357294
H	3.425416	0.709885	-1.217330
B	0.660148	3.175687	-0.167924
F	1.227793	4.427075	-0.428343
F	0.066864	3.187373	1.109320
F	-0.345113	2.904634	-1.115164
N	4.543258	1.906404	1.606631
N	4.001144	1.310632	0.865045
Si	3.693775	-1.543322	-0.020175
C	3.113042	-2.548371	-1.483412
H	3.463932	-3.587589	-1.370575
H	2.020035	-2.556146	-1.573014

H	3.540600	-2.147202	-2.415942
C	5.540611	-1.255088	-0.152504
H	6.044084	-2.235967	-0.185948
H	5.809393	-0.712140	-1.072621
H	5.948738	-0.707560	0.711507
C	3.247598	-2.178297	1.669214
H	2.173746	-2.397263	1.739013
H	3.816249	-3.103650	1.861451
H	3.522822	-1.448438	2.447386

65

TS2b-B_5 Eopt -1992.364197

C	-1.466435	0.689193	0.169832
C	-0.824792	0.542468	-1.199493
C	0.281737	-0.159059	-1.445042
C	0.853731	-1.058695	-0.379294
C	0.510943	-0.585981	1.009845
C	-0.575670	0.127282	1.321900
C	1.170707	0.020437	-2.641539
C	2.391094	-1.059435	-0.395464
C	2.996490	0.313873	-0.821822
C	2.445892	0.765795	-2.193862
C	4.488310	0.026095	-0.854473
C	4.010238	-0.509816	1.393168
C	2.813888	-1.400401	1.040857
H	1.444850	-0.956625	-3.067220
H	-1.253723	1.212529	-1.948956
H	3.230072	0.643876	-2.953704
H	4.570006	-0.857463	2.268477
O	-1.611599	2.032420	0.452825
C	-0.860653	0.638295	2.705733
H	-1.937129	0.704275	2.914300
H	-0.400531	-0.008514	3.464442
H	-0.448362	1.650247	2.825594
C	1.683079	-0.845860	1.939765
H	1.418225	-1.502764	2.780955
H	0.664996	0.594258	-3.430490
H	2.212843	1.838241	-2.149178
O	5.236362	0.131052	-1.788985
O	4.954517	-0.399825	0.333775
C	2.828725	1.398967	0.301691
H	1.761748	1.657589	0.367204
C	3.258466	0.799753	1.666477
H	3.839819	1.524619	2.251013
H	2.995601	-2.470695	1.186161
H	2.757862	-1.806037	-1.111591
C	3.618655	2.673069	0.006350
H	3.394061	3.433979	0.768359
H	4.706236	2.495198	0.028983
H	3.367060	3.100921	-0.974064
O	2.186988	0.374494	2.478224
O	0.353518	-2.393560	-0.445500
C	0.560765	-3.139602	-1.618607
H	1.629580	-3.275122	-1.857109
H	0.127077	-4.132166	-1.436750
H	0.057988	-2.698260	-2.495562
C	-2.678089	-0.147714	0.426335
H	-3.329618	0.344105	1.164657
B	-2.697041	2.890842	0.007419
F	-2.856441	2.842725	-1.396995
F	-2.393129	4.189087	0.406443
F	-3.925357	2.487800	0.593228
N	-4.553801	0.464382	-1.917836
N	-3.919721	0.330508	-1.035440
Si	-3.032806	-2.072248	0.347787
C	-4.837274	-2.176591	0.841218
H	-4.999464	-1.771182	1.852875

H	-5.511502	-1.653022	0.145669
H	-5.139095	-3.237515	0.854828
C	-2.786901	-2.663749	-1.405605
H	-3.703356	-2.504334	-1.995410
H	-1.960086	-2.134851	-1.898302
H	-2.562931	-3.742046	-1.416886
C	-1.993493	-2.892551	1.661625
H	-2.396025	-3.907011	1.823060
H	-0.945984	-2.966083	1.342359
H	-2.051498	-2.350922	2.618727
65			
TS2b-B_6		Eopt	-1992.368863
C	-1.611464	0.866123	0.046280
C	-1.022777	0.447812	-1.287529
C	-0.029089	-0.427664	-1.434778
C	0.426447	-1.245850	-0.256949
C	0.178466	-0.556507	1.059087
C	-0.794041	0.337069	1.272622
C	0.873198	-0.516813	-2.630929
C	1.942340	-1.478077	-0.270355
C	2.730265	-0.257113	-0.838278
C	2.218435	0.134541	-2.244824
C	4.162861	-0.759285	-0.844694
C	3.649766	-0.990450	1.448769
C	2.326130	-1.721858	1.195186
H	1.036440	-1.565014	-2.924041
H	-1.364265	1.053931	-2.130142
H	2.982326	-0.118627	-2.992971
H	4.168835	-1.326826	2.353104
O	-1.764774	2.238560	0.087317
C	-1.055357	0.970990	2.609455
H	-0.103340	1.199316	3.104338
H	-1.617632	1.906393	2.502786
H	-1.625851	0.287956	3.260071
C	1.302544	-0.905086	2.024806
H	0.938678	-1.424964	2.922635
H	0.445017	0.008582	-3.495924
H	2.078585	1.222959	-2.279735
O	4.906616	-0.854589	-1.784045
O	4.579474	-1.132483	0.379975
C	2.734819	0.946778	0.169373
H	1.718396	1.358293	0.219075
C	3.107252	0.437783	1.584969
H	3.800772	1.125342	2.085878
H	2.345289	-2.785112	1.457596
H	2.187122	-2.341144	-0.902814
C	3.671958	2.072629	-0.262477
H	4.730787	1.766946	-0.229603
H	3.451809	2.425348	-1.279665
H	3.550599	2.929642	0.416271
O	2.004900	0.258890	2.448322
O	-0.271663	-2.486923	-0.139927
C	-0.157449	-3.438258	-1.167997
H	-0.495725	-3.053137	-2.144790
H	0.869314	-3.827434	-1.278887
H	-0.809596	-4.276922	-0.886907
C	-2.922798	0.259469	0.407714
H	-3.383025	0.829891	1.229690
B	-0.666990	3.199398	-0.070418
F	-0.025662	3.076367	-1.319178
F	0.305438	3.042511	0.936628
F	-1.247348	4.467267	0.031239
N	-4.602333	1.685074	-1.685111
N	-4.041595	1.195953	-0.881886
Si	-3.670445	-1.535888	0.269199
C	-2.896230	-2.519016	1.651497

H	-3.012435	-2.003923	2.618063
H	-3.420406	-3.487119	1.722031
H	-1.831541	-2.701177	1.453577
C	-5.489676	-1.262274	0.622885
H	-5.645492	-0.772307	1.597294
H	-5.992625	-0.664811	-0.153795
H	-5.992837	-2.243140	0.659252
C	-3.432290	-2.178456	-1.462148
H	-2.383283	-2.130402	-1.777864
H	-3.769471	-3.226263	-1.521840
H	-4.035934	-1.589915	-2.171851

65

TS2b-B_7 Eopt -1992.363603

C	1.462445	0.639778	0.112859
C	0.744389	0.256586	1.393240
C	-0.365972	-0.472196	1.453894
C	-0.907886	-1.147056	0.221285
C	-0.492832	-0.434672	-1.042926
C	0.634907	0.277134	-1.171729
C	-1.253881	-0.632229	2.647337
C	-2.451539	-1.121370	0.184845
C	-3.043830	0.111968	0.934443
C	-2.563674	0.145177	2.401753
C	-4.546188	-0.094860	0.830375
C	-3.979465	-0.046002	-1.454152
C	-2.845092	-1.063126	-1.302356
H	-1.471463	-1.703209	2.786858
H	1.151827	0.741606	2.285468
H	-3.357739	-0.256069	3.045932
H	-4.504829	-0.124831	-2.412378
O	1.590139	2.013127	0.128473
C	0.994797	1.017544	-2.427233
H	2.077935	1.043185	-2.599109
H	0.512458	0.560257	-3.300852
H	0.657993	2.061841	-2.358531
C	-1.658506	-0.373407	-2.016130
H	-1.424469	-0.801079	-3.002050
H	-0.768059	-0.269581	3.563408
H	-2.410611	1.188756	2.707643
O	-5.334733	-0.197441	1.731253
O	-4.971634	-0.164635	-0.443176
C	-2.768321	1.442202	0.141647
H	-1.688676	1.647363	0.182970
C	-3.156221	1.242290	-1.348191
H	-3.673773	2.126806	-1.741593
H	-3.086619	-2.046805	-1.718694
H	-2.838017	-2.023220	0.680538
C	-3.511540	2.642762	0.725053
H	-4.604176	2.537727	0.623835
H	-3.284731	2.797766	1.788798
H	-3.217720	3.556343	0.187401
O	-2.070539	0.979977	-2.209786
O	-0.401470	-2.475761	0.287188
C	-0.858641	-3.418504	-0.648356
H	-0.761247	-3.066082	-1.690587
H	-0.228006	-4.310572	-0.529137
H	-1.907487	-3.713260	-0.471903
C	2.708780	-0.136904	-0.140409
H	3.407351	0.432354	-0.773662
B	2.748805	2.860039	-0.126002
F	2.273987	4.144272	-0.380015
F	3.509866	2.411854	-1.240472
F	3.609255	2.880033	0.990434
N	4.279535	0.350099	2.448962
N	3.774555	0.267843	1.480962
Si	3.115728	-2.043971	-0.222233

C	4.985261	-2.070007	-0.339811
H	5.350336	-1.428668	-1.157823
H	5.481660	-1.761421	0.593342
H	5.307457	-3.102565	-0.555925
C	2.540449	-2.947880	1.298294
H	2.994332	-2.520348	2.206604
H	1.446334	-2.923015	1.388248
H	2.867725	-3.998794	1.226463
C	2.398313	-2.594739	-1.855843
H	1.305347	-2.516589	-1.883612
H	2.814121	-1.994444	-2.680634
H	2.683301	-3.646160	-2.027708

65

TS2b-B_8 Eopt -1992.362757

C	1.467739	0.664233	0.171209
C	0.757337	0.183806	1.426012
C	-0.361240	-0.540364	1.440949
C	-0.892766	-1.124365	0.156972
C	-0.485428	-0.317525	-1.048714
C	0.621723	0.428012	-1.126025
C	-1.299045	-0.658796	2.607246
C	-2.429018	-1.100436	0.113086
C	-3.042049	0.128132	0.853975
C	-2.546038	0.203164	2.316470
C	-4.535472	-0.133954	0.753129
C	-3.966641	-0.083532	-1.536534
C	-2.792037	-1.054810	-1.377965
H	-1.601077	-1.706536	2.754547
H	1.156473	0.630681	2.341927
H	-3.362979	-0.092268	2.989111
H	-4.491519	-0.185738	-2.492759
O	1.626354	2.026688	0.315296
C	0.966011	1.271239	-2.319505
H	2.045744	1.305113	-2.510232
H	0.460790	0.899611	-3.219891
H	0.642020	2.308548	-2.150891
C	-1.620281	-0.308050	-2.058580
H	-1.326052	-0.731938	-3.029645
H	-0.823343	-0.318346	3.537484
H	-2.299394	1.246261	2.556941
O	-5.321660	-0.259493	1.653148
O	-4.953765	-0.233512	-0.521842
C	-2.821061	1.462027	0.054208
H	-1.750743	1.711221	0.099208
C	-3.195574	1.239326	-1.434473
H	-3.747156	2.099344	-1.835927
H	-2.974028	-2.048933	-1.800225
H	-2.832598	-1.997547	0.599722
C	-3.616375	2.630871	0.633994
H	-3.352493	3.557961	0.103932
H	-4.702796	2.483998	0.519622
H	-3.408503	2.788185	1.701464
O	-2.092905	1.018316	-2.284343
O	-0.405063	-2.439778	-0.103727
C	-0.689432	-3.469145	0.809941
H	-1.771159	-3.653287	0.926421
H	-0.234387	-4.381274	0.400670
H	-0.254725	-3.284217	1.806528
C	2.682280	-0.111400	-0.211059
H	3.379839	0.514750	-0.789043
B	2.817588	2.848623	0.155428
F	3.712295	2.653105	1.228355
F	2.402440	4.177016	0.122278
F	3.520202	2.545442	-1.042966
N	4.336727	-0.050387	2.358153
N	3.810362	0.027034	1.401134

Si	3.028779	-2.005802	-0.552353
C	4.865612	-2.012167	-0.919708
H	5.474549	-1.660276	-0.071923
H	5.181148	-3.044717	-1.145214
H	5.102369	-1.390801	-1.798242
C	2.666859	-2.993596	0.989476
H	3.531529	-2.965922	1.671572
H	1.789805	-2.607878	1.525388
H	2.477940	-4.047400	0.730343
C	2.083802	-2.466510	-2.092893
H	2.486304	-3.423971	-2.464887
H	1.014606	-2.583981	-1.873999
H	2.217814	-1.713859	-2.885738

10. References

[1]Profitt, J. A., Jones, T. & Watt, D. S. A Convenient Synthesis of 3-Methoxyphthalic Anhydride. *Synth. Commun.* **5**, 457–460 (1975).

[2]Frébault, F., Oliveira, M. T., Wöstefeld, E. & Maulide, N. A Concise Access to 3-Substituted 2-Pyrones. *J. Org. Chem.* **75**, 7962–7965 (2010).

[3]Haider, M., Sennari, G., Eggert, A & Sarpong, R. Total Synthesis of the Cephalotaxus Norditerpenoids (±)-Cephanolides A–D. *J. Am. Chem. Soc.* **143**, 2710–2715 (2021).

[4]Sennari, G., Gardner, K. E., Wiesler, S., Haider, M., Eggert, A. & Sarpong, R. Unified Total Syntheses of Benzenoid Cephalotane-Type Norditerpenoids: Cephanolides and Ceforalides. *J. Am. Chem. Soc.* **144,** 19173–19185 (2022).

[5]Camps, P., González, A., Muñoz-Torrero, D., Simon, M., Zúñiga, A., Martins, M. A., Font-Bardia, M. & Solans, X. Synthesis of Polysubstituted Bicyclo[3.3.1]nonane-3,7-diones from Cyclohexa-2,5-dienones and Dimethyl 1,3-Acetonedicarboxylate. *Tetrahedron* **56**, 8141-8151 (2000).

[6]Buta, J. G., Flippen, J. L. & Lusby, W. R. Harringtonolide, a plant growth inhibitory tropone from *Cephalotaxus harringtonia* (Forbes) K. Koch. *J. Org. Chem.* **43**, 1002–1003 (1978).

[7]Sun, N.-J., Xue, Z., Liang, X.-T. & Huang, L. Studies on the structure of a new antitumor agent – hainanolide. *Acta Pharm. Sin.* **14**, 39–43 (1979).

[8]Zhang, M., Liu, N. & Tang, W. Stereoselective Total Synthesis of Hainanolidol and Harringtonolide via Oxidopyrylium-Based [5+2] Cycloaddition. *J. Am. Chem. Soc.* **135**, 12434–12438 (2013).

[9]Zhang, H.-J., Hu, L., Ma, Z., Li, R., Zhang, Z., Tao, C., Cheng, B., Li, Y., Wang, H. & Zhai, H. Total Synthesis of the Diterpenoid (+)-Harringtonolide. *Angew. Chem. Int. Ed.* **55**, 11638–11641 (2016).

[10]Chai, J.-D. & Head-Gordon, H. Long-range corrected hybrid density functionals with damped atom-atom dispersion corrections. *Phys. Chem. Chem. Phys.* **10**, 6615–6620 (2008).

[11]Weigend, F. & Ahlrichs, R. Balanced basis sets of split valence, triple zeta valence and quadruple zeta valence quality for H to Rn: Design and assessment of accuracy. *Phys. Chem. Chem. Phys.* **7**, 3297–3305 (2005).

[12]Mardirossian, N. & Head-Gordon, M. ωB97M-V: A combinatorially optimized, range-separated hybrid, meta-GGA density functional with VV10 nonlocal correlation. *J. Chem. Phys.* **144**, 214110 (2016).

[13]Vydrov, O. A. & Van Voorhis, T. J. Nonlocal can der Waals density functional: The simpler the better. *J. Chem. Phys.* **133**, 244103 (2010).

[14]Hujo, W. & Grimme, S. Performance of the van der Waals Density Functional VV10 and (hybrid)GGA Variants for Thermochemistry and Noncovalent Interactions. *J. Chem. Theory Comput.* **7**, 3866–3871 (2011).

[15]Goerigk, L., Hansen, A., Bauer, C., Ehrlich, S., Najibi, A. & Griime, S. A look at the density functional theory zoo with the advanced GMTKN55 database for general main group thermochemistry, kinetics and noncovalent interactions. *Phys. Chem. Chem. Phys.* **19**, 32184–32215 (2017).

[16]Marenich, A. V., Cramer, C. J. & Truhlar, D. G. Universal Solvation Model Based on Solute Electron Density and on a Continuum Model of the Solvent Defined by the Bulk Dielectric Constant and Atomic Surface Tensions. *J. Phys. Chem. B* **113**, 6378–6396 (2009).

[17]Frisch, M. J., et al. Gaussian 16, Revision C.01. (2016).

[18]Neese, F. The ORCA program system. *Wiley Interdiscip. Rev. Comput. Mol. Sci.* **2**, 73–78 (2012).

[19]Fukui, K. The path of chemical reactions – the IRC approach. *Acc. Chem. Res.* **14**, 363–368 (1981).

[20]Grimmer, S. Supramolecular Binding Thermodynamics by Dispersion-Corrected Density Functional Theory. *Chem. Eur. J.* **18**, 9955–9964 (2012).

[21] Luchini, G., Alegre-Requena, J. V., Funes-Ardoiz, I. & Paton, R. S. GoodVibes: automated thermochemistry for heterogeneous computational chemistry data. *F1000Research* **9**, 291 (2020).

[22]Bryantsev, V. S., Diallo, M. S. & Goddard III, W. A. Calculation of Solvation Free Energies of Charged Solutes Using Mixed Cluster/Continuum Models. *J. Phys. Chem. B* **112**, 9709 (2008).

[23]Contreras-Garcia, J., Johnson, E. R., Keinan, S., Chaudret, R., Piquemal, J.-P., Beratan, D. N. & Yang, W. NCIPLOT: A Program for Plotting Noncovalent Interaction Regions. *J. Chem. Theory Comput.* **7**, 625–632 (2011).

[24]https://github.com/zorkzou/Molden2AIM (Accessed September 13th 2023)
